# *Para*-(3-phenylpropiolamido)phenyl (PPAP) glycosides: Harnessing *ipso*-cyclization–driven glycosylation for strategic flexibility

**DOI:** 10.1126/sciadv.ady4274

**Published:** 2025-07-25

**Authors:** Meifang Yang, Yanli Qiu, Yan Tan, Li Song, Xi Xiang, Yitian Zhao, Xing Zheng, Xiangwei Zheng, Weiliang Gu, Guoqiang Lin, Houchao Tao

**Affiliations:** ^1^ Shanghai Frontiers Science Center of TCM Chemical Biology, Innovation Research Institute of Traditional Chinese Medicine, Shanghai University of Traditional Chinese Medicine, Shanghai 201203, China.; ^2^ Group of Lead Compound, Department of Pharmacy, University of South China, Hengyang, Hunan 421001, China.; ^3^ Department of Pharmaceutical Engineering, Zhejiang Pharmaceutical University, Ningbo 315100, China.; ^4^ School of Pharmacy, Shanghai University of Traditional Chinese Medicine, Shanghai 201203, China.

## Abstract

We herein report *para*-(3-phenylpropiolamido)phenyl (PPAP) glycosides as a novel class of glycosyl donors with distinct advantages for carbohydrate synthesis. These donors, featured by an intrinsically stable phenolic linkage, undergo glycosylation via a unique *ipso*-cyclization–mediated activation. Activated with *N*-iodosuccinimide (NIS)/trimethylsilyl trifluoromethanesulfonate (TMSOTf), PPAP donors support both O- and N-glycosylation across a broad range of substrates. Guided by density functional theory calculations, their design allows straightforward synthesis through a simple amide coupling reaction, facilitating diverse latent-active transformations and broadening their strategic utility. Furthermore, the distinct reactivity profile of PPAP donors—marked by a clear gap relative to other known donors and orthogonality to many standard activation methods—makes them well suited for modular, one-pot glycosylation strategies. Their ease of synthesis, robust performance in glycosylation, and compatibility with diverse assembly approaches collectively establish PPAP glycosides as powerful tools for the efficient construction of complex carbohydrates.

## Teaser

A distinct class of glycosyl donor aligns with strategies for complex sugar synthesis via *ipso*-cyclization–mediated activation.

## INTRODUCTION

Carbohydrates, as one major class of biomolecules, encompass broad and prominent biological roles, beyond their traditional consideration as energy sources or structural components (*1*). Once considered “dark matter,” carbohydrates have since been a focal point in modern biology, leading to the naissance of glycobiology (*2*) and driving notable advances in carbohydrate-based drug discovery (*3*). Despite this progress, carbohydrate chemistry continues to face formidable challenges, primarily due to the inherent structural heterogeneity of naturally occurring glycans, which are not directly templated by DNA (*4*). This complexity necessitates the synthesis of homogeneous glycosides with precisely defined structures, yet current synthetic methodologies often struggle to meet these demands (*5*). Unlike oligopeptides and oligonucleotides, the synthesis of glycosides is particularly challenging. Enzymatic methods, while offering high specificity, are limited by substrate scope, enzyme availability, and cost (*6*). In contrast, chemical synthesis provides a more versatile and cost-effective approach to assembling both natural and unnatural derivatives (*7*–*8*).

Central to chemical synthesis of carbohydrates is glycosylation, a process for which a plethora of glycosyl donors have been developed (*9*). Besides glycosyl hemiacetal used directly in the pioneering Fischer glycosylation (*10*), representative donors include glycosyl halides [bromides (*11*) and fluorides (*12*)], glycosyl trichloroacetimidates (TCAs) (*13*) and later modified glycosyl *N*-phenyl trifluoroacetimidates (PTFAs) (*14*), thioglycosides (*15*), phosphites (*16*), alkenyl glycosides (*17*), and, more recently, glycosyl *o*-alkynylbenzoates (ABzs) (*18*) and cyclopropyl donors (*19*). Each type of glycosyl donor offers distinct benefits in terms of preparation, stability, or activation conditions, thus broadening the toolkit available to carbohydrate chemists. When strategically organized, these donors can markedly improve the efficiency of synthesizing complex molecules.

**Fig. 1.**
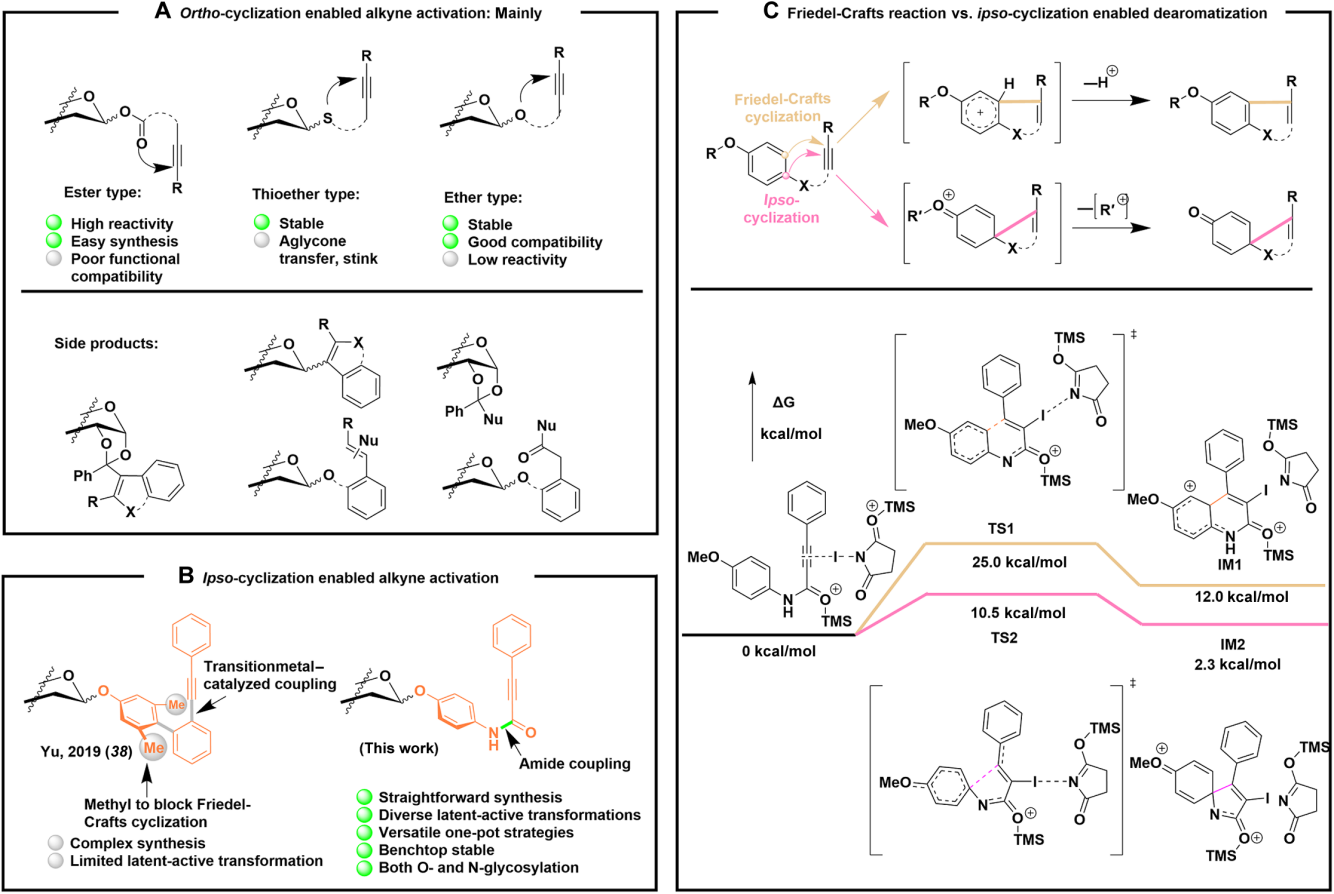
Distinct alkyne-tethering glycosyl donors. (**A**) *Ortho*-cyclization and (**B**) *ipso*-cyclization enable alkyne activation. (**C**) DFT calculations for the competition between *ipso*-cyclization and Friedel-Crafts reaction of amide-linked PPAP donors using B3LYP/def2-SVP//M06-2X/def2-TZVP. TMS, Trimethylsilyl; TS1, Transition state 1; TS2, Transition state 2; IM1, Intermediate 1; IM2, Intermediate 2.

In the past two decades, alkyne-tethered donors have emerged as an innovative class of reagents for glycosylation (*8*). Most of these donors rely on *ortho*-cyclization–mediated alkyne activation (Fig. 1A) and can be classified into ester (*18*, *20*–*24*), thioether (*25*–*30*) and ether types (*31*–*35*), each with distinct advantages and drawbacks. Ester-type donors are typically easy to synthesize and highly reactive; however, with the exception of the recently reported 8-alkynyl-1-naphthoate donor (*36*), they often suffer from poor functional compatibility in carbohydrate substrates. In contrast, thioether and ether types are more stable and amenable to “latent-active” strategies, although they are largely limited in transition metal–catalyzed transformations. Thioether-type donors share common drawbacks with thioglycosides, such as unpleasant odor and potential aglycone transfer. Ether-type donors, while stable, often exhibit lower reactivity, leading to unwanted side products, such as *C*-glycosides (*27*), or acceptor alkyne–conjugated by-products (*31*, *33*), further reducing their utility.

Recently, a new type of alkyne-tethered glycosyl donor, 3,5-dimethyl-4-(2′-phenylethynylphenyl)phenyl (EPP) glycosides, has emerged, which proceed via an unprecedented dearomative activation mechanism, involving *ipso*-cyclization via *para*-carbon attack on the alkyne (Fig. 1B) (*37*–*38*). EPP donors avoid many of the aforementioned side reactions. However, their complex synthesis, particularly the construction of the biaryl core with doubly *meta*-methylated phenyl for sugar attachment, has hindered widespread adoption. These methyl groups were introduced to block competing Friedel-Crafts reactions that prevent the leaving group departure and the subsequent glycosyl oxonium ion formation. Their omission yields 4-(2′-phenylethynylphenyl)phenyl (demethyl-EPP) glycosides, which exhibit limited efficiency in glycosylation (*39*).

In this context, we introduce *para*-(3-phenylpropiolamido)phenyl (PPAP) glycosides as a novel class of glycosyl donors (Fig. 1B). Density functional theory (DFT) calculations indicate that these donors, when treated with *N*-iodosuccinimide (NIS)/trimethylsilyl trifluoromethanesulfonate (TMSOTf), undergo exclusive *ipso*-cyclization to form spirocyclic products, even in the absence of methyl block (Fig. 1C). In contrast, the competing Friedel-Crafts pathway leading to fused products is disfavored due to substantially higher activation barriers and less stable intermediates. Notably, this pronounced selectivity arises from the dual activation by TMSOTf, which engages not only NIS but also the amide group. Although this activation increases both the overall barrier and the energy of intermediates along both pathways, it disproportionately raises the energy of the intermediate in the fusion pathway. In the absence of this amide effect, PPAP and demethyl-EPP donors exhibit comparable energy profiles (fig. S1). PPAP donors thus overcome existing limitations in synthetic accessibility and activation efficiency. Their preparation involves a straightforward amide coupling reaction, avoiding the challenges associated with transition metal–catalyzed alkyne installation and eliminating the multistep synthesis required for EPP donors. Furthermore, the electronic properties of PPAP glycosides can be finely tuned through substitutions on the phenylpropionic acid (PPA) moiety. The amide-based design enables multiple latent-active transformations by altering the protecting groups on the aniline component—an advantage over systems constrained to a single activation strategy. Together, the simplicity, versatility, and robustness of PPAP glycosides position them as powerful tools for the construction of complex carbohydrate architectures.

## RESULTS AND DISCUSSION

### Optimization of leaving group and activation conditions

**Fig. 2.**
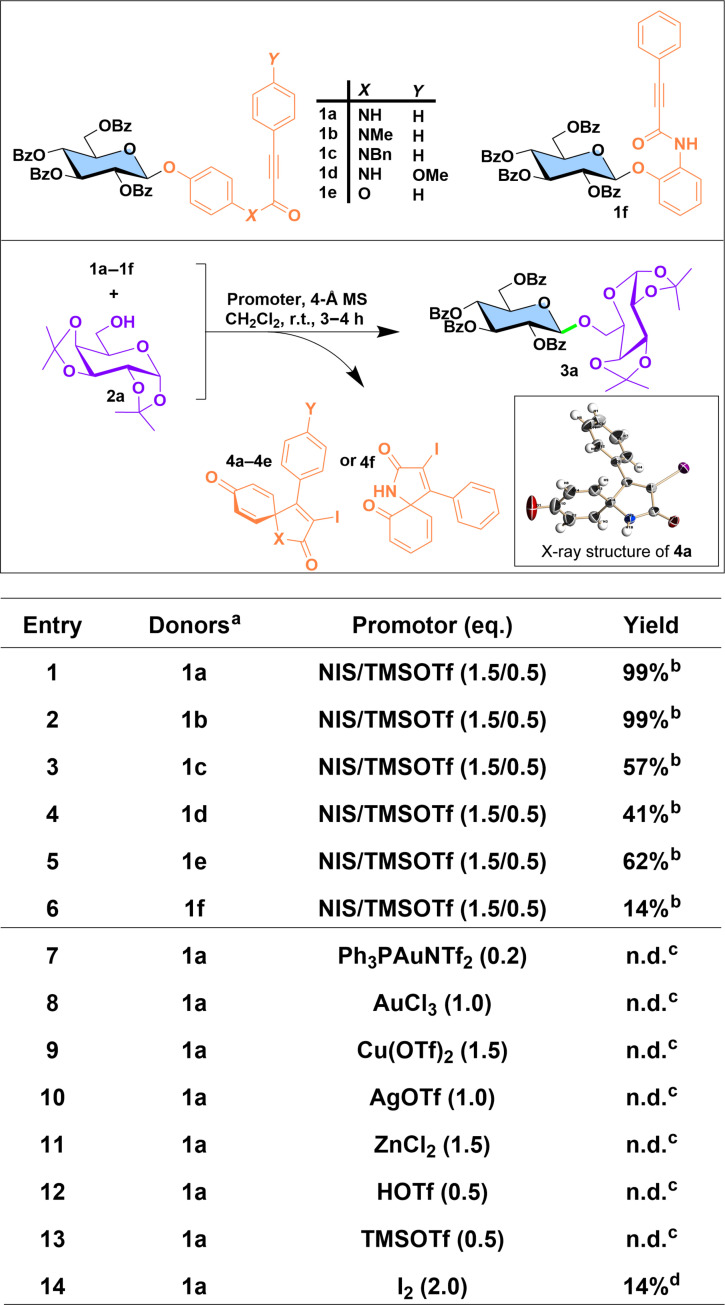
Optimization of leaving group and activation conditions for PPAP donors. ^a^Donor:acceptor = 1.2:1; CH_2_Cl_2_, 0°C to room temperature (r.t.) for 3 to 4 hours (h). ^b^Isolated yield. ^c^Not detectable by thin-layer chromatography (TLC). ^d^Yield was determined on the basis of ^1^H nuclear magnetic resonance (NMR) using 1,3,5-trimethybenzene as the internal standard.

Electrophile-triggered *ipso*-cyclization of alkynes via electron-rich carbons has been well documented for efficiently forming spirocycles, often accompanied by concurrent demethylation of *para*-methoxy groups (*40*–*42*). However, when the methyl group is replaced with a carbohydrate moiety, it is unclear whether a similar mechanism—specifically, the formation of an oxonium ion leading to glycosylation—can occur smoothly. To explore this, we designed and synthesized a series of glycosides containing either a phenylpropiolamide or ester (figs. S2 to S7), with the aim of identifying optimal glycosyl donors (Fig. 2).

We first evaluated the reactivity of a set of PPAP donors and derivatives (**1a** to **1f**) by glycosylation with a common acceptor, diacetone d-galactose (**2a**) (Fig. 2). As anticipated, the PPAP donor (**1a**), when activated with NIS [1.5 equivalent (eq.)] and TMSOTf (0.5 eq.), produced the desired β-linked disaccharide (**3a**) in nearly quantitatively (entry 1). The corresponding leaving fragment (**4a**) was also isolated in stoichiometric amounts and its structure was confirmed by x-ray diffraction [Cambridge Crystallographic Data Centre (CCDC): 2391061], thus verifying the dearomative *ipso*-cyclization mechanism. No ring-fused product was identified, indicating that no Friedel-Crafts reaction occurred. This outcome obviates the need for installing neighboring methyl groups to block potential side reactions, as required for EPP donor (*38*), thereby greatly simplifying the synthesis of this type of donors. The N-methylated analog (**1b**) gave comparable results under this promotion (entry 2), while the N-benzylated derivative (**1c**) showed a markedly reduced yield (entry 3). The *para*-methoxy substituted donor (**1d**), initially hypothesized to enhance electronic richness of the alkyne and thus promote *ipso*-cyclization, actually resulted in much lower yields (entry 4), likely due to the involvement of Friedel-Crafts pathway (*39*). The ester surrogate (**1e**) was similarly less effective than the amide donor, yielding considerably less product (entry 5), and the *ortho*-substituted analog (**1f**) exhibited minimal activity, providing a very low yield of product (entry 6). These findings suggest that the structurally simple *para*-linked phenylpropiolamide is the optimal donor for further investigation.

We next screened a variety of promoters for the selected PPAP donor (**1a**) to assess the orthogonality of the above NIS/TMSOTf system with respect to other activation conditions (Fig. 2). The PPAP donor stayed inert under the condition of Ph_3_PAuNTf_2_ (*18*) (entry 7), typically used in Yu glycosylation protocols. Similarly, other metal-based π-acids such as AuCl_3_ and Cu(OTf)_2_ (entries 8 and 9), which are commonly used for activating alkyne-containing donors such as propargyl glycoside (*43*) and the recently developed *ortho*-methoxycarbonylethynylphenyl thioglycosides (*28*), were inactive with the PPAP donor. Other acids, including AgOTf, ZnCl_2_, TMSOTf, and HOTf (entries 10 to 13), also failed to initiate glycosylation (*44*). We attempted I_2_ (*45*), a condition conducive to *ipso*-cyclization, but it also resulted in substantially lower yields (14%, entry 14).

### Scope of O-glycosylations

**Fig. 3.**
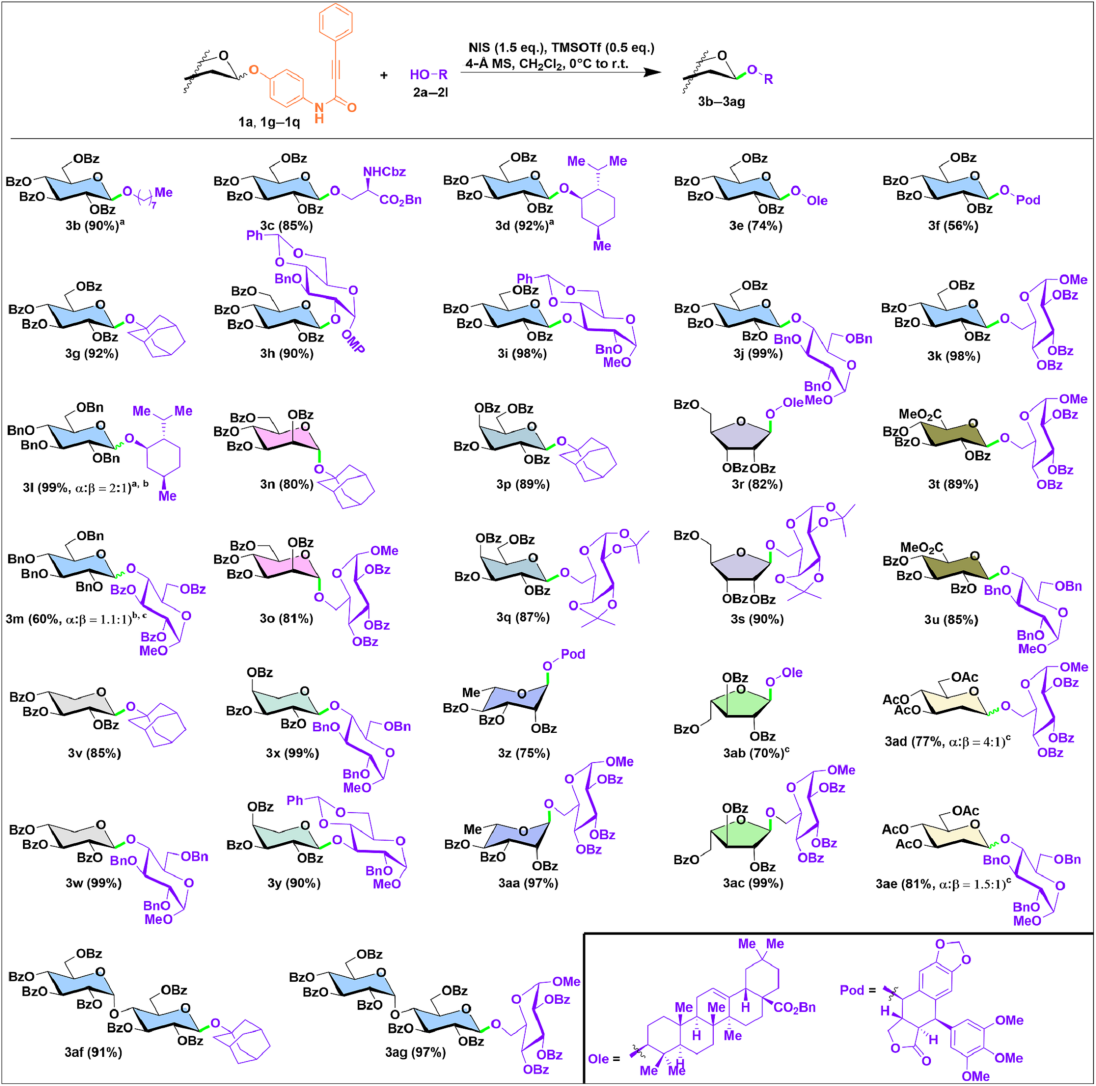
Scope of *O*-glycoside synthesis with glycosyl PPAP donors. ^a^The amount of donor and TMSOTf were increased to 1.5 and 1.0 eq., respectively. ^b^The α/β ratio was determined by ^1^H NMR analysis of the product mixture. ^c^The amount of TMSOTf was increased to 1.0 eq.

With the optimal leaving group and activation condition in hand, we investigated the substrate scope of PPAP donors for *O*-glycoside synthesis (Fig. 3). Using glucosyl PPAP (**1a**) as the donor, we successfully glycosylated a range of nonglycosyl acceptors, including *n*-octanol, protected serine, and menthol, affording the corresponding glycosides (**3b** to **3d**) with over 85% efficiency. However, sterically hindered acceptors, such as benzyl oleanolate and podophyllotoxin, showed decreased efficiency, yielding 56 to 74% yields (**3e** and **3f**). The glycosylation of 1-adamantanol, a bulky tertiary alcohol, still yielded high (**3g**, 92%), indicating its compatibility with this glycosylation process. Glycosyl acceptors spanning 2-, 3-, 4-, and 6-OH of properly protected sugars were consistently glycosylated to produce β-glucosides in excellent yields (**3h** to **3k**, 90 to 99%) as the model reaction (**3a**, 99%; Fig. 2).

We further examined the scope of PPAP donors. Beyond the perbenzoylated glucosyl donor (**1a**), we synthesized a diverse set of other PPAP donors with the same leaving group (figs. S8 to S18). These included the perbenzylated glucosyl donor (**1g**), and perbenzoylated mannosyl (**1h**), galactosyl (**1i**), ribofuranosyl (**1j**), glucuronate (**1k**), xylosyl (**1l**), arabinosyl (**1m**), rhamnosyl (**1n**), arabinofuranosyl (**1o**) donors, the peracetylated 2-deoxyglucosyl donor (**1p**), and the disaccharide maltosyl donor (**1q**). While the perbenzylated glucosyl donor (**1g**) glycosylated menthol smoothly (**3l**), yielding an expected anomeric mixture, and it also enabled disaccharide formation with a sterically hindered and electron-deficient acceptor (**3m**), although in attenuated yield. Other aldosyl donors, including pyranosyl and furanosyl, as well as disaccharide donors, generally performed well with various acceptors, such as 1-adamantanol, natural aglycones, and glycosyl alcohols at varied positions (**3n** to **3ag**, up to 99%). Even challenging glycosylations involving glucuronate and 2-deoxyglucsoyl donors proceeded efficiently (**3t** and **3u** and **3ad** and **3ae**, 77 to 89%).

### Scope of N-glycosylations

**Fig. 4.**
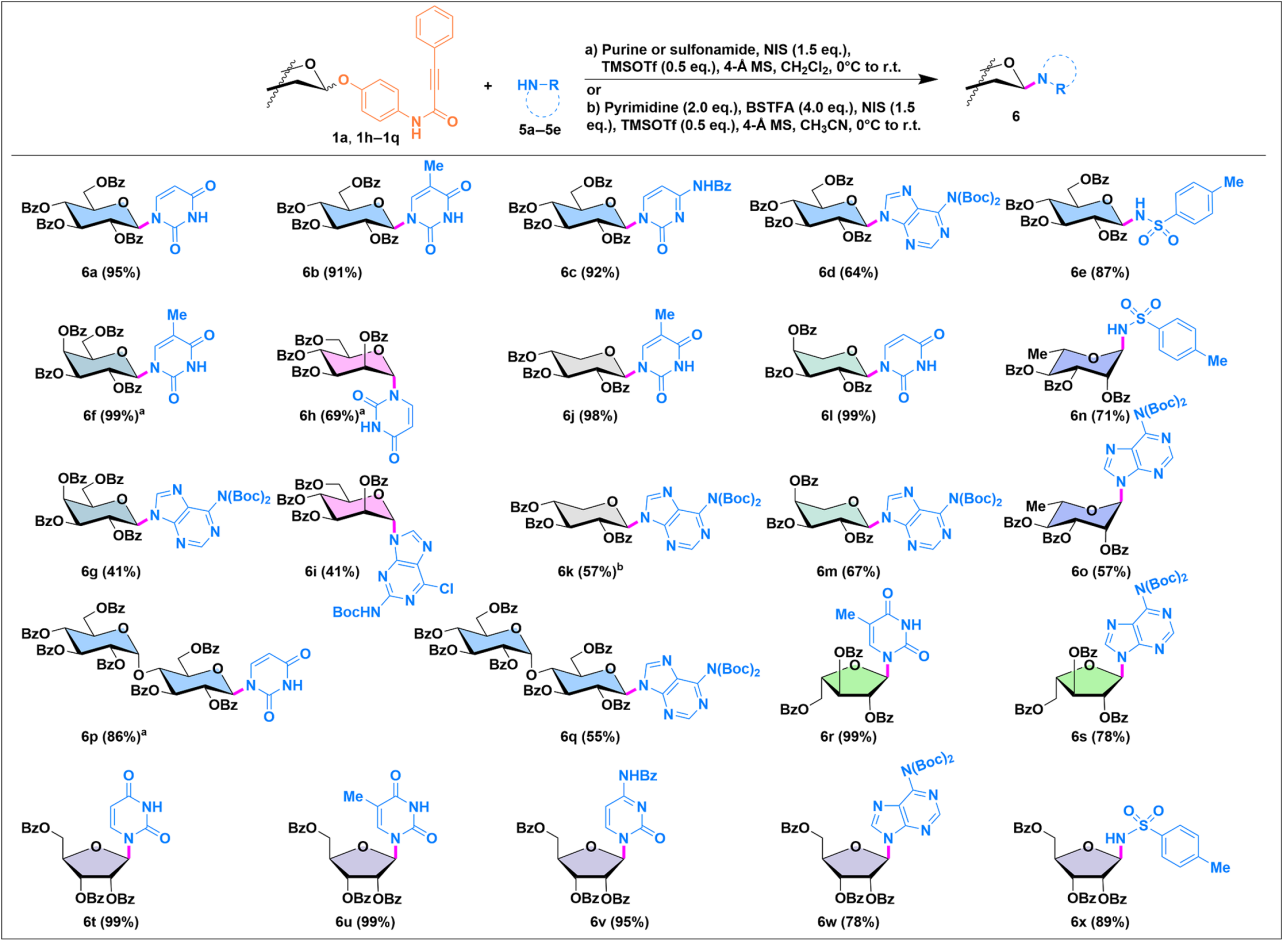
Scope of *N*-glycoside synthesis with glycosyl PPAP donors. ^a^The reaction was performed in a mixture of solvents (CH_2_Cl_2_:MeCN = 3:1). ^b^The reaction performed at −20°C to room temperature.

For *N*-glycosides, which are notoriously difficult to glycosylate because of the typically low nucleophilicity and poor solubility of the corresponding *N*-acceptors (*32*, *46*), PPAP donors also showed promise (Fig. 4). Glycosylation of the glucosyl PPAP donor (**1a**) with pyrimidines proceeded efficiently, yielding the desired *N*-glycosides (**6a** to **6c**) in excellent yields (>91%). The use of *N*,*O*-bis(trimethylsilyl)trifluoroacetamide as a pretreatment reagent enhanced the solubility of pyrimidines, thereby improving the glycosylation efficiency (*47*). In contrast, glycosylation with purines resulted in moderate yield (**6d**, 64%), consistent to prior observations, likely due to even lower nucleophilicity of purines, as well as potential challenges related to regioselectivity (*48*). An *N*-sulfonamide glycoside (**6e**) was obtained in high yield, demonstrating the versatility of this method. Further N-glycosylations were attempted on for the synthesis of other common pyranosides, including galactosides (**6f** and **6g**), mannosides (**6h** and **6i**), xyloside (**6j** and **6k**), arabinosides (**6l** and **6m**), rhamnosides (**6n** and **6o**), and maltosides (**6p** and **6q**). Consistent with the results observed for glucosylation, glycosylation with pyrimidines gave higher yields (up to 99%), compared to purines (41 to 67%) and sulfonamides (71%). Relatively higher yields were generally observed for *N*-furanosides (**6r** to **6x**), particularly with purines (**6s** and **6w**) and sulfonamide (**6x**), highlighting the potential of this approach for diverse *N*-glycosides.

### PPAP precursors as stable protective groups

**Fig. 5.**
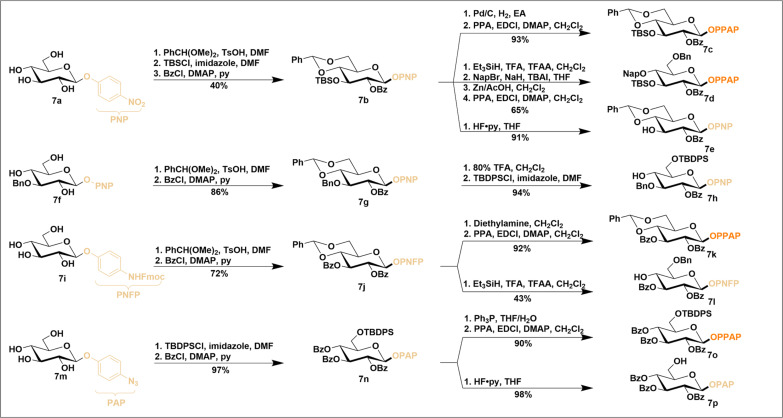
PNP, PNFP, and PAP groups as stable protective groups for the synthesis of active donors and latent acceptors. Fmoc, 9-fluorenylmethyloxycarbonyl; DMF, N,N′-dimethylformamide; DMAP, 4-dimethylaminopyridine; EA, ethyl acetate; TFA, trifluoroacetic acid; TFAA, trifluoroacetic anhydride; TBAI, tetrabutylammonium iodide; THF, tetrahydrofuran; TBDPSCl, tert-Butylchlorodiphenylsilane; EDCI, 1-(3-Dimethylaminopropyl)-3-ethylcarbodiimide hydrochloride; OPNP, O-p-nitrophenyl; OPNFP, O-p-NHFmoc; OPAP, O-p-N3; OPPAP, O-p-(3-phenylpropiolamido)phenyl.

The latent-active strategy is an appealing approach in the efficient synthesis of complex carbohydrate molecules (*49*–*50*). This iterative strategy requires intermediates to be capably prepared in two distinct forms: active donor and latent acceptor. The leaving group on the latent forms must remain inert during glycosylation but be readily convertible into active forms for subsequent steps. In addition, the latent leaving group must be stable enough to withstand various protecting group manipulations, allowing for the generation of either latent acceptors or active donors as needed. In this regard, PPAP donors are particularly well suited for the latent-active strategy. Structurally, PPAP donors are phenyl *O*-glycosides that feature an amide linkage between an aniline and an alkyne functionality. The stable nature of phenyl *O*-glycosides ensures the inertness of latent forms under most glycosylation conditions. Moreover, the amide linkage enables modular synthesis, facilitating the late-stage introduction of reactive functionality of alkynes, which allows for precise control over conversion between latent and active forms. Unlike traditional latent-active strategies, which typically rely on a single transformation method, PPAP donors offer multiple pathways for latent-active transformations. Depending on the aniline precursor and its protection, various options are available, including *para*-nitro, *para*-NHFmoc (9-fluorenylmethyloxycarbonyl), and *para*-azide (Fig. 5). Starting with *para*-nitrophenyl (PNP) glucoside (**7a**), which is commercially available in bulk, several manipulations can be performed. For example, global protection with benzoyl or benzyl groups under basic conditions (figs. S2 and S8), or benzylidenation under acidic condition followed by tris-buffered saline and benzoyl protection, affords fully protected latent form (**7b**). Subsequent manipulations, including a nitro reduction followed by acetylation with PPA, convert these intermediates into active donors (**7c**, Fig. 5). Notably, further manipulations, such as chemoselective reduction of benzylidene and sequential protection with Nap groups, convert the intermediate (**7b**) into an orthogonally protected active donor (**7d**) that will serve as a valuable building block for diverse saccharide synthesis. Alternatively, other manipulations lead to the preparation of a latent acceptor (**7e**). An orthogonally protected latent acceptor (**7h**) can also be prepared from 3-benzylated starting PNP glucoside (**7f**) in four steps. These processes highlight the versatility of the PNP group as a stable anomeric protecting group and its ease of conversion into active forms. The wide applicability of this approach was further demonstrated through the preparation of latent acceptors (**7l** and **7p**) and active donors (**7k** and **7o**) using *para*-NHFmoc phenyl (PNFP) and *para*-azidophenyl (PAP) glucosides (**7i** and **7m**). Notably, the two active forms (**7k** and **7o**) were transformed from their latent forms (**7j** and**7n**) via secondary amine-mediated deprotection and triphenylphosphine reduction, respectively, expanding the scope of this methodology.

### Latent-active strategies based on PPAP donors

**Fig. 6.**
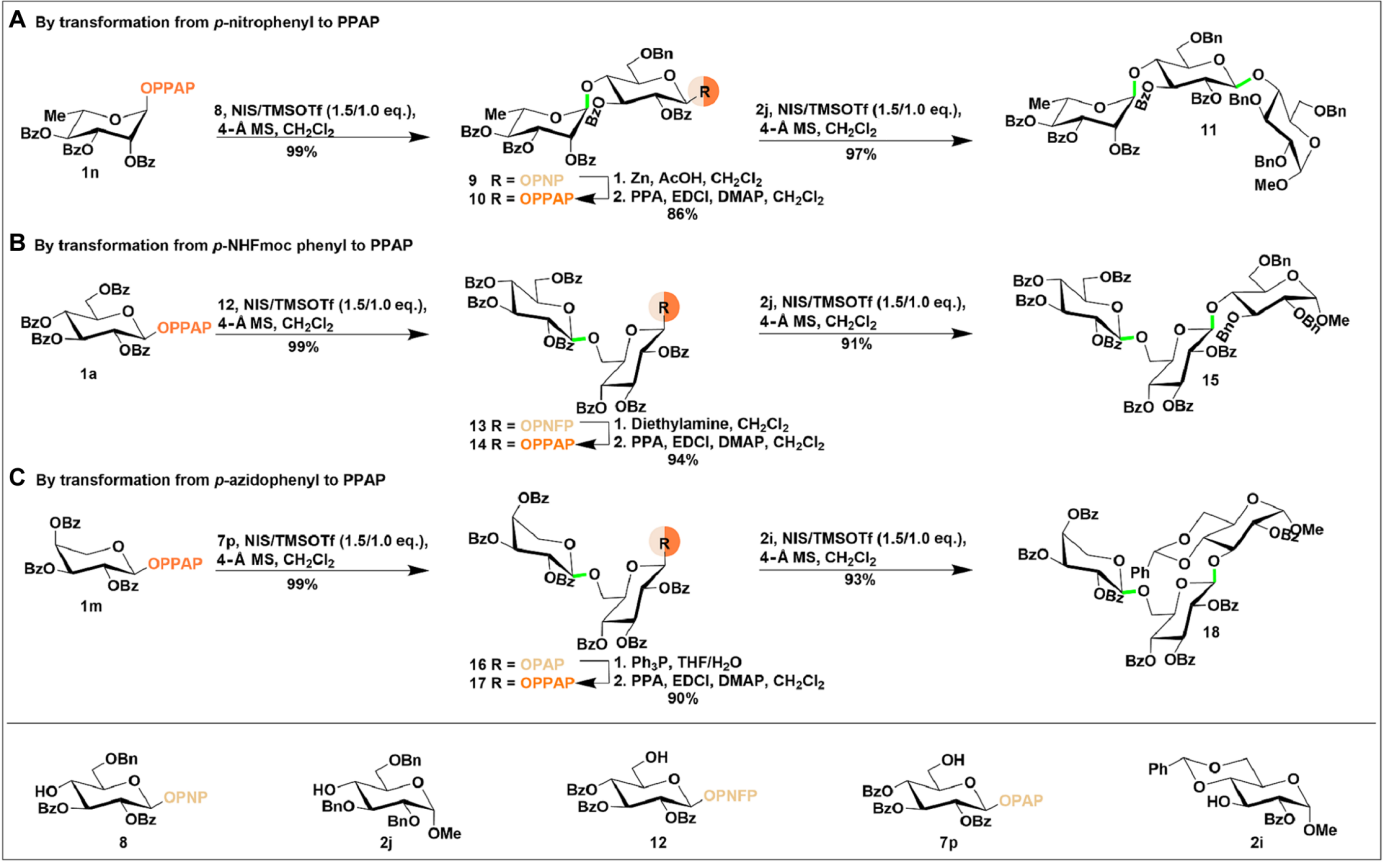
Synthesis of trisaccharides using latent-active strategies based on PPAP donors. (**A**) By transformation from *p*-nitrophenyl to PPAP; (**B**) By transformation from *p*-NHFmoc phenyl to PPAP; (**C**) By transformation from *p*-azidophenyl to PPAP.

Building on the demonstrated compatibility of protecting group manipulation and latent-active transformations, we applied the PPAP donor system to the synthesis of saccharides using latent-active strategies (Fig. 6). In the first example, an active rhamnosyl donor (**1n**) was glycosylated with a latent acceptor (**8**) under standard conditions, quantitatively yielding the latent disaccharide donor (**9**). This latent PNP donor (**9**) was then efficiently converted into an active disaccharide donor (**10**) in 86% yield using a two-step procedure. The newly activated donor was subsequently glycosylated with a second acceptor (**2j**), resulting in the formation of the trisaccharide (**11**) with two 1 → 4 linkages in quantitative yield (Fig. 6A). In the second example, an active glucosyl donor (**1a**) was smoothly coupled with a latent PNFP acceptor featuring a *para*-NHFmoc group (**12**), producing a disaccharide latent donor (**13**). After deprotection with diethylamine and functionalization with PPA, this disaccharide latent donor was converted to an active disaccharide donor (**14**) in 94%. The active donor was then glycosylated with a second acceptor (**2j**) to afford the corresponding trisaccharide (**15**) in 91%. In the third example, an active xylosyl donor (**1m**) was glycosylated with a latent PAP acceptor (**7p**) bearing a *para*-azide group, yielding the disaccharide latent donor (**16**) quantitatively. This latent donor was successfully converted to an active donor (**17**) through a reduction and acylation process in 80%. Last, the resulting active donor was glycosylated with a second acceptor (**2i**), producing the trisaccharide (**18**) in 93%. These examples demonstrate the versatility of the PPAP donor system, providing precise control over the reactivity of intermediates. The system’s wide tolerance for various reaction conditions, including protecting group manipulations and glycosylations, highlights its utility in the efficient construction of complex saccharide molecules.

### One-pot strategies with PPAP donors

**Fig. 7.**
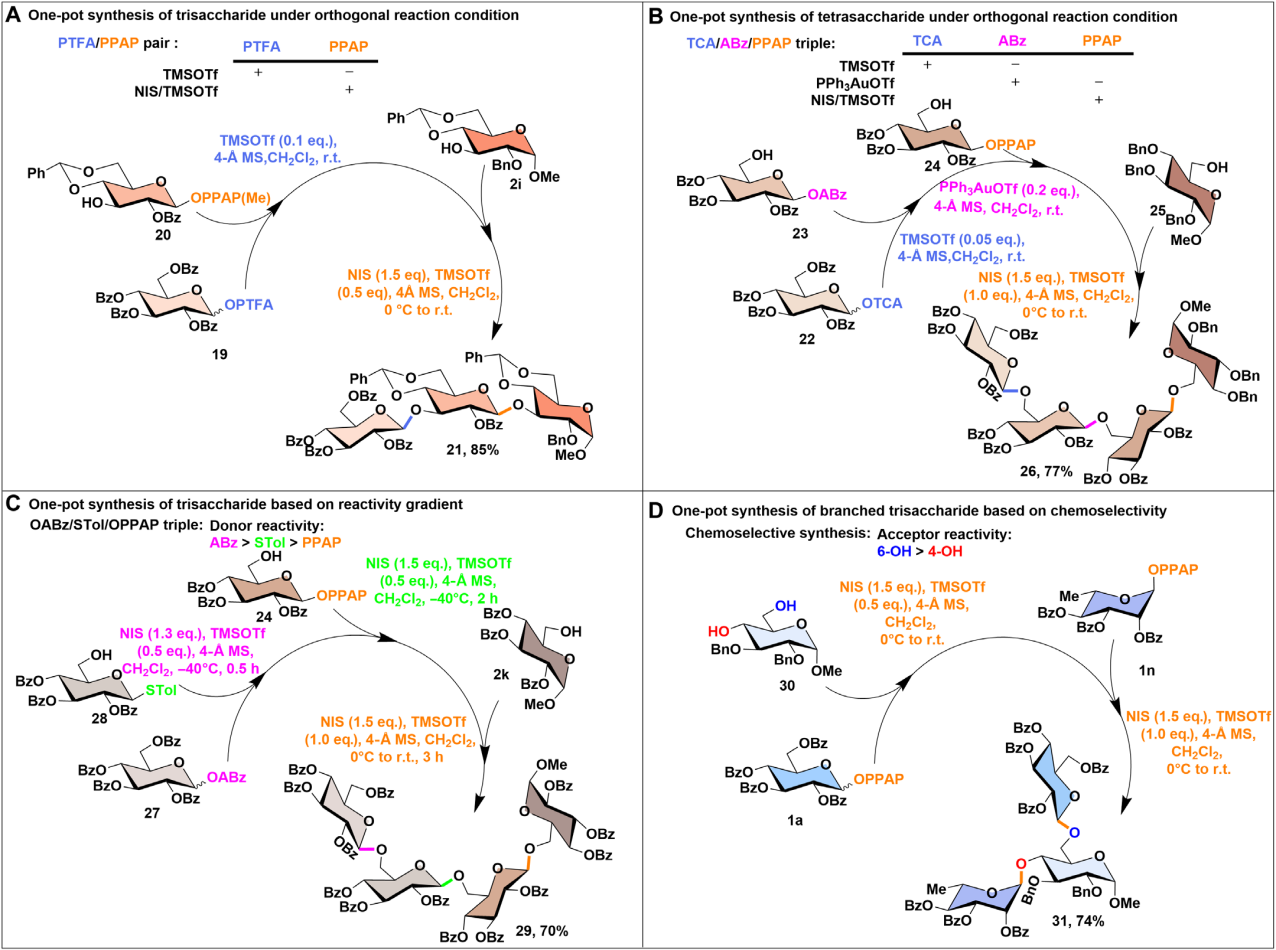
Synthesis of saccharides via various one-pot strategies by the combination of different glycosyl donors and promotors. (**A**) One-pot synthesis of trisaccharide under orthogonal reaction condition. (**B**) One-pot synthesis of tetrasaccharide under orthogonal reaction condition. (**C**) One-pot synthesis of trisaccharide based on reactivity gradient. Stol, thioglycoside. (**D**) One-pot synthesis of branched trisaccharide based on chemoselectivity.

The one-pot synthesis of carbohydrate molecules, a strategy in which multiple glycosylation reactions are performed sequentially in a single vessel without isolating intermediates, offers substantial advantages in efficiency, sustainability, and practicality (*51*–*54*). By streamlining the process, it saves time, reduces chemical waste, and accelerates overall synthesis. A crucial aspect of designing an effective one-pot approach is the exploitation of either orthogonal promotion conditions or reactivity differences between glycosyl donors in the reaction sequence. In this regard, PPAP donors are promising candidates for integration into various one-pot schemes. In our preliminary screening, we observed that PPAP donors were inert under TMSOTf promotion conditions, which are typically used for imidates. Accordingly, the first one-pot synthesis combined PTFA (*44*) and PPAP donors. Specifically, the coupling of a glucosyl PTFA donor (**19**) with a PPAP acceptor (**20**) under TMSOTf catalysis afforded a disaccharide intermediate, which was further glycosylated with an acceptor (**2i**) under NIS/TMSOTf promotion, yielding a trisaccharide (**21**) in 85% yield within a single vessel (Fig. 7A). Noted here is that an N-methylated PPAP acceptor (**20**) was used to increase the solubility, as its nonmethylated version displayed poor solubility. Nevertheless, the N-methylated PPAP acceptor underwent glycosylation with similar efficiency. Similarly, PPAP donors were inert under Au(I)-promoted conditions, typically used for glycosyl ABz, while ABz donors were inactive under TMSOTf promotion. This reactivity profile allowed for the design of a second one-pot synthesis combining TCA, ABz, and PPAP donors. In this case, a glucosyl TCA donor (**22**) was coupled with an ABz acceptor (**23**) under TMSOTf catalysis to afford a disaccharide intermediate. This intermediate was then glycosylated with a PPAP acceptor (**24**) using a catalytic amount of Ph_3_PAuOTf, providing the trisaccharide intermediate. Last, the trisaccharide intermediate was further glycosylated with an acceptor (**25**) under NIS/TMSOTf promotion, producing tetrasaccharide (**26**) in 77% yield, again achieved in one-pot (Fig. 7B).

The NIS/TMSOTf promotion condition has been widely used for activating various glycosyl donors, including classic thioglycosides and some alkyne-containing donors such as ABz donors (*15*, *55*). Competition experiments (*17*, *38*, *55*) revealed that the reactivity of these donors follows the order: ABz donors > thioglycosides > PPAP glucosides (figs. S37 and S38). On the basis of this reactivity hierarchy, we designed the third one-pot synthesis to sequentially activate glycosyl ABz, thioglycoside, and PPAP donors (Fig. 7C). Glycosylation of the ABz donor (**27**) with a thioglycoside acceptor (**28**) under NIS/TMSOTf activation (1.3/0.5 eq.) at −40°C furnished a disaccharide intermediate. This intermediate was subsequently coupled with the disarmed PPAP acceptor (**24**) using an additional portion of NIS/TMSOTf (1.5/0.5 eq.) at the same temperature, forming a trisaccharide PPAP donor. Last, this trisaccharide was glycosylated with acceptor (**2k**) using another portion of NIS/TMSOTf (1.5/1.0 eq.), yielding a tetrasaccharide (**29**) in 70% yield within the same reaction vessel. An additional stoichiometric amount of TMSOTf was required to drive the reaction complete, likely due to the accumulation of amide-containing by-products during the course of the reaction, such as succinimide and the leaving fragment, which may sequester or deactivate TMSOTf through coordination, thereby diminishing its effective concentration, as reported before (*55*).

In addition, during our exploration of the reaction scope, we observed that steric hindrance, as expected, could influence the reactivity of PPAP donors. This finding enabled the development of a fourth one-pot synthesis based on regioselective glycosylation of the 4,6-diol acceptor (**30**). The more reactive 6-OH of acceptor (**30**) was first glycosylated with PPAP donor (**1a**, 1.2 eq.), followed by glycosylation of 4-OH with a rhamnosyl donor (**1n**, 1.5 eq.). This approach furnished a branched trisaccharide (**31**) in an overall yield of 74% (Fig. 7D).

In conclusion, we have developed a novel glycosylation protocol using PPAP glycosides as donors, which proceeds via a dearomative activation mechanism. DFT calculations indicate that coordination of TMSOTf with the amide carbonyl group of the PPAP donor favors the *ipso*-cyclization pathway over the competing Friedel-Crafts pathway. This inherent selectivity eliminates the need for *meta*-methyl substitution to suppress the undesired pathway, thereby simplifying donor synthesis. This protocol, effective under mild conditions, demonstrates a broad reaction scope, including both O- and N-glycosylation of various glycosyl and nonglycosyl acceptors. The stability of PPAP glycosides and their precursors allows the use of various protecting groups and enables their application in the streamlined synthesis of glycans and glycoconjugates through versatile latent-active strategies. PPAP donors are compatible with the reaction conditions required for other glycosyl donors, such as TCA donors, PTFA donors, and ABz donors, making them viable donors for orthogonal one-pot synthesis strategies. On the other hand, although sharing the same promotion condition of NIS/TMSOTf, reactivity of PPAP donors is relatively lower than that of thioglycoside and ABz donors, facilitating other one-pot synthetic schemes using single promotion condition. Together, the PPAP donor–based glycosylation methods prospect the broad application in the synthesis of complex oligosaccharides and glycoconjugates, warranting further investigation.

## MATERIALS AND METHODS

### General information

All commercial reagents and solvents were used without further purification. Crushed 4-Å molecular sieves (MS) were activated through flame-drying under high vacuum immediately before use. All reactions were monitored by thin-layer chromatography (TLC). The TLC plates were visualized with ultraviolet light and/or by staining with methanol/H_2_SO_4_ (10%, v/v). Flash column chromatography was performed on Silica Gel 60 (200 to 300 mesh). Nuclear magnetic resonance (NMR) spectra were measured on Bruker AVANCE III 400 or 600-MHz NMR spectrometer. High-resolution mass spectra were recorded on an Agilent 6230 mass spectrometer (electrospray ionization, positive ion mode). Optical rotations were measured on an Autopol IV (serial no. 83493), using CHCl_3_ and CH_2_Cl_2_ as solvent. Single-crystal x-ray data were collected on Bruker D8 Venture x-ray single-crystal diffractometer using Cu Kα radiation (λ = 1.54178 Å).

### Typical procedure for glycosylations with PPAP donors

A solution of glycosyl PPAP donors (1.2 eq.) and acceptors (1.0 eq.) in dry CH_2_Cl_2_ (0.1 M) was stirred at room temperature for 30 min in the presence of activated 4-Å MS (3.0 g/mmol) under N_2_ atmosphere. Then, the vessel was chilled to 0°C, to which NIS (1.5 eq.) and TMSOTf (0.5 eq.) were added. The reaction mixture was stirred for 3 hours after the temperature gradually rise to room temperature. Then, Et_3_N was added to quench the reaction, and the solvent was removed under reduced pressure. The resulting residue was purified by silica gel column chromatography to afford the glycosylated product.

## Supplementary Materials

### This PDF file includes:

Supplementary Methods Figs. S1 to S40 X-ray structures DFT calculations NMR spectra References
