# Supplementary material for: Para-(3-phenylpropiolamido)phenyl (PPAP) glycosides: Harnessing ipso-cyclization–driven glycosylation for strategic flexibility
Source: Sci Adv. 2025 Jul 25;11(30):eady4274. doi: 10.1126/sciadv.ady4274 (PMC12292917; doi:10.1126/sciadv.ady4274)
Supplement: Supplementary file 1 — Supplementary Methods Figs. S1 to S40 X-ray structures DFT calculations NMR spectra References [file sciadv.ady4274_sm.pdf]

Science Advances
AAAS

Supplementary Materials for

Para-(3-phenylpropiolamido)phenyl (PPAP) glycosides: Harnessing *ipso*-cyclization–driven glycosylation for strategic flexibility

Meifang Yang *et al.*

Corresponding author: Houchao Tao, houchao@shutcm.edu.cn; Guoqiang Lin, lingq@sioc.ac.cn; Weiliang Gu, gwl22@shutcm.edu.cn; Xiangwei Zheng, zhengxhsh@hotmail.com

Sci. Adv. **11**, eady4274 (2025)
DOI: 10.1126/sciadv.ady4274

This PDF file includes:

Supplementary Methods
Figs. S1 to S40
X-ray structures
DFT calculations
NMR spectra
References

DFT calculation for amide-linked PPAP donors.

All density functional theory (DFT) calculations were performed using Gaussian09 rev.E.01. The B3LYP function is well-suited for optimization and vibrational analysis owing to its computational efficiency and stability(*56*), whereas M062X function is preferable for energy calculations given its superior ability to describe thermodynamic properties(*57*). This combination successfully achieves a balance between computational cost and accuracy in practical applications. Geometry optimizations were carried out with the B3LYP/def2-SVP, incorporating the D3 version of Grimme's dispersion corrections with zero-damping. Frequency analysis was conducted at the same level of theory as the geometry optimization, utilizing the harmonic oscillator model to confirm whether the optimized stationary points correspond to local minima or transition states. Single-point energy was evaluated with M06-2X/def2-TZVP basis set for all atoms. The 3D diagrams of the optimized structures were generated using GaussView 6.016 software.

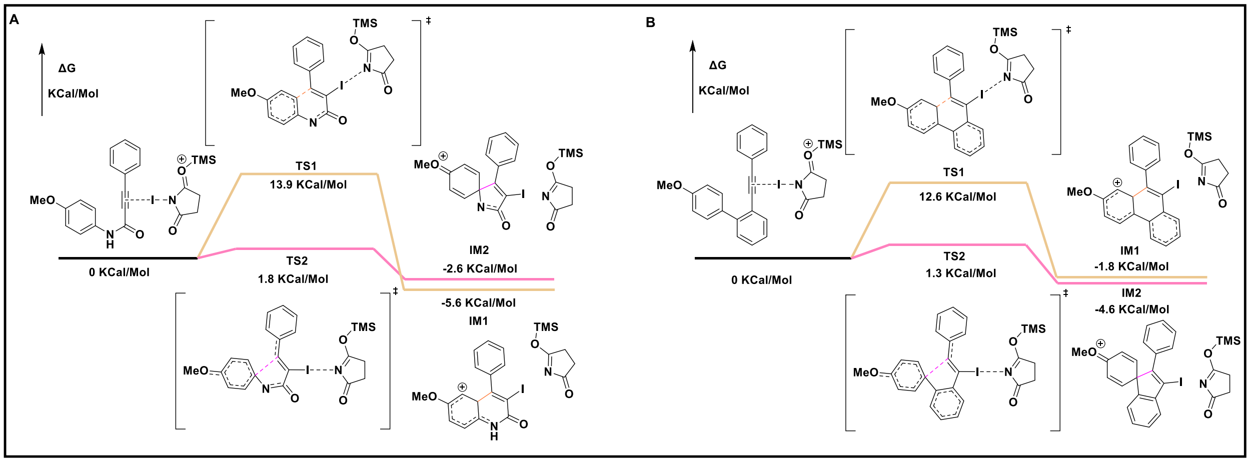

Fig. S1. DFT calculation for the competition between *ipso*-cyclization and Friedel-Crafts reaction of PPAP donors (A) and 4-(2'-phenylethynylphenyl)phenyl (demethyl-EPP) donors (B) in the absence of amide effect were performed with B3LYP/def2-SVP//M06-2X/def2-TZVP.

Cartesian coordinates of all optimized structures shown in Fig. 1C

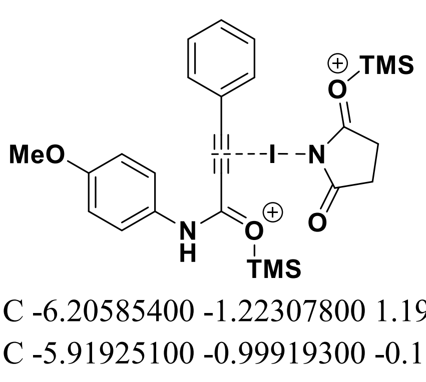

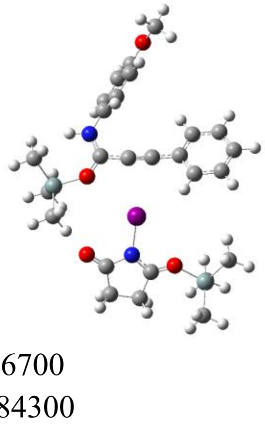

C -6.20585400 -1.22307800 1.19286700
C -5.91925100 -0.99919300 -0.16384300
C -4.98575700 -0.03004900 -0.52199100
C -4.60915300 0.48947800 1.81693500
C -5.53852400 -0.46880100 2.17821000
H -6.43032100 -1.55839300 -0.94562300

H -4.77674800 0.15772400 -1.57706900
H -5.78175100 -0.65948900 3.22349200
C -4.33308100 0.70540300 0.46264300
C -1.54937800 -1.06343800 -0.83900300
C -1.32421600 -2.45726400 -1.02069900
C -0.33675500 -2.91657700 -1.91239300
C -2.12622900 -3.36882200 -0.30763000
C -0.15823900 -4.28282500 -2.08486700
H 0.26663300 -2.20050400 -2.47266700
C -1.93228200 -4.73253400 -0.48848800
H -2.89080200 -2.99462600 0.37524300
C -0.95244100 -5.18719100 -1.37422200
H 0.59381200 -4.65019800 -2.78372800
H -2.54826500 -5.44628500 0.05905100
H -0.80979200 -6.25939100 -1.51741300
C -1.78759400 0.11972600 -0.65336100
O -7.08600200 -2.11766900 1.63519400
C -7.84011400 -2.87139900 0.70698900
H -8.44747200 -2.21386800 0.06616700
H -7.18573400 -3.49861700 0.08140700
H -8.50058200 -3.51333000 1.29748200
I 1.28697500 0.38306400 -0.14005100
C 4.94186900 2.59719000 0.78508900
C 5.49607900 1.17208900 0.70639200
H 5.40099300 3.29138200 0.06847000
H 5.04278600 3.05002300 1.78142400
H 6.22864000 1.03005800 -0.10249400
H 5.96942400 0.82309500 1.63678300
N 3.20033300 1.06165600 0.27715500
C 4.29165500 0.31867100 0.41400600
C 3.46777900 2.46914000 0.46535600
O 4.26112400 -0.93144600 0.29543100
O 2.62327500 3.29597800 0.37183800
Si 5.47590400 -2.26858000 0.51175400
C 7.01916900 -1.71041600 -0.35691600
H 7.58275500 -0.94598100 0.19601600
H 7.68363900 -2.58455000 -0.45259700
H 6.81761100 -1.35209800 -1.37759300
C 4.58870800 -3.65860400 -0.32034900
H 5.18742100 -4.57942800 -0.25277000
H 3.61755500 -3.84951400 0.15810300
H 4.42300600 -3.44285900 -1.38552800
C 5.62917000 -2.41591300 2.35279700
H 6.27020600 -3.27946300 2.59060200

H 6.09459900 -1.53391800 2.81588600
H 4.65149100 -2.59114800 2.82413100
H -4.09537200 1.07123000 2.58403200
N -3.37745700 1.71555600 0.08718700
H -3.63832200 2.69501700 0.21124100
C -2.20506500 1.47584900 -0.46571600
O -1.40705900 2.40135900 -0.83846200
Si -1.32300100 4.17974900 -0.63235800
C -3.00527700 4.83125800 -1.10292700
H -3.77299900 4.72946400 -0.32030300
H -2.91038700 5.91261900 -1.29125400
H -3.37651800 4.37789100 -2.03428600
C -0.89515600 4.38757900 1.16223900
H -1.64068200 3.93743500 1.83427700
H 0.08955500 3.94427400 1.36941300
H -0.83931500 5.45991400 1.40548400
C 0.02761900 4.62303700 -1.80985500
H -0.21506600 4.31411000 -2.83635600
H 0.18662800 5.71168600 -1.80999100
H 0.96612500 4.14280000 -1.49784200

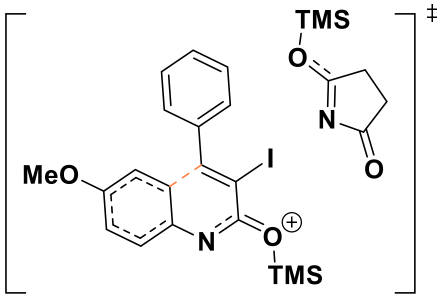

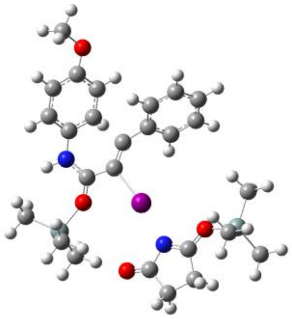

TS1

C -5.75135000 -1.56399000 -0.48916400
C -5.92217800 -0.38708300 -1.25690500
C -5.12336100 0.73547500 -1.04802900
C -3.95687600 -0.44966900 0.73777800
C -4.78085700 -1.57535100 0.51194900
H -6.67822500 -0.35002900 -2.04156500
H -5.25790200 1.61908200 -1.67395200
H -4.68198700 -2.46988800 1.12986500
C -4.15056700 0.71220500 -0.05922900
C -1.98996800 -0.78596900 0.50139300
C -1.71870700 -2.17448600 0.74552700
C -2.18863100 -3.18101300 -0.12025900
C -0.92272600 -2.50741400 1.86066400
C -1.84653600 -4.50277900 0.11921500
H -2.81201000 -2.91557300 -0.97627300

C -0.60056900 -3.83802900 2.09866100
H -0.56014100 -1.71860000 2.52227700
C -1.06020200 -4.83093400 1.23137100
H -2.19592400 -5.28657800 -0.55325800
H 0.00765200 -4.10342700 2.96383200
H -0.81019200 -5.87555900 1.42422600
C -1.36000000 0.35598300 0.39949900
O -6.46862200 -2.66715600 -0.64385800
C -7.53095500 -2.69918100 -1.58591200
H -8.28048600 -1.92708500 -1.35576900
H -7.14996100 -2.56755500 -2.60989700
H -7.98926100 -3.68774000 -1.49278600
I 0.88385600 0.43141300 0.11972200
C 5.10245600 2.31759100 -0.29269600
C 5.54198100 0.85366400 -0.30384600
H 5.32893300 2.85096300 -1.22607600
H 5.53229100 2.90639900 0.52808300
H 6.08479400 0.55304200 -1.21247500
H 6.16984400 0.57790700 0.55625700
N 3.19335700 0.89028400 -0.09969900
C 4.23155900 0.10617900 -0.21132900
C 3.59140300 2.25001500 -0.12532300
O 4.11204000 -1.16633200 -0.22261800
O 2.82083400 3.16142900 -0.03700800
Si 5.23538100 -2.50494100 -0.52829600
C 5.55394700 -2.46503900 -2.35911400
H 6.10846900 -1.56871900 -2.67279200
H 6.16297900 -3.33770500 -2.64180600
H 4.61506800 -2.51461800 -2.92883800
C 4.22124800 -3.95171500 0.03083000
H 4.76094700 -4.89251200 -0.15242400
H 4.00489200 -3.88834400 1.10682300
H 3.26889000 -3.99163700 -0.51706200
C 6.75725100 -2.22433100 0.50472300
H 7.34123000 -3.15823900 0.51989500
H 7.41612500 -1.44227700 0.10263700
H 6.50733000 -1.98348400 1.54887200
H -3.60240700 -0.30725200 1.76803400
N -3.28781900 1.79987500 0.14049200
H -3.63768000 2.74571900 -0.02308700
C -1.97815300 1.68129700 0.39613200
O -1.26102900 2.69427700 0.59074000
Si -0.96321300 4.42187400 0.15904600
C -0.15107800 4.27788300 -1.49647900

H 0.03877400 5.28392600 -1.90198600
H 0.81732500 3.76840300 -1.38205200
H -0.77104900 3.73493300 -2.22456000
C -2.66784100 5.17995100 0.12510900
H -3.27443900 4.83559400 -0.72816000
H -3.21557900 5.01967500 1.06641000
H -2.57063000 6.27042300 0.00202600
C 0.13256700 4.97190000 1.53400900
H 1.07466800 4.40556600 1.48964600
H 0.37200400 6.03950700 1.41624000
H -0.33477200 4.83395600 2.51888000

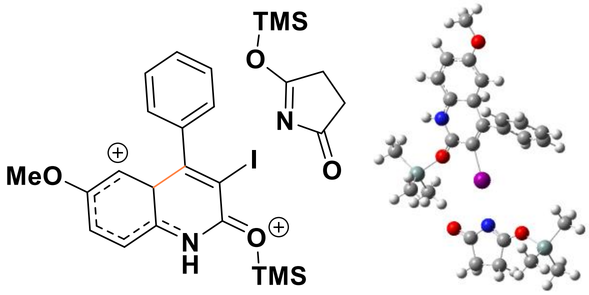

IM1
C 5.96962700 -1.66229000 0.03860600
C 6.46275600 -0.32728000 0.17228800
C 5.64782300 0.78781500 0.05535400
C 3.72132900 -0.70440700 -0.43108900
C 4.62473600 -1.84303600 -0.20421400
H 7.52436500 -0.16603900 0.37015200
H 6.07314600 1.78520200 0.17978500
H 4.24385500 -2.85684400 -0.33806900
C 4.28457500 0.63095400 -0.18946800
C 2.21327100 -0.84228800 -0.26625300
C 1.65977900 -2.20399400 -0.16728200
C 0.73711000 -2.67110000 -1.11445500
C 2.03553200 -3.02621700 0.90873100
C 0.21125200 -3.95431600 -0.99283700
H 0.43887200 -2.03114100 -1.94681400
C 1.48040800 -4.29372100 1.04198200
H 2.74113800 -2.66217600 1.66054600
C 0.57638200 -4.76144900 0.08509700
H -0.49084500 -4.32454000 -1.74057900
H 1.75497000 -4.92049900 1.89094000
H 0.15578100 -5.76343200 0.18157100
C 1.43753100 0.28293700 -0.30699200
O 6.72553600 -2.74141000 0.14659500
C 8.11245900 -2.62962600 0.44614200
H 8.64118500 -2.08280700 -0.34889600

H 8.26379700 -2.14125200 1.42053200
H 8.49190600 -3.65407300 0.49231400
I -0.69823600 0.38593400 -0.12861000
C -5.04364200 2.47841600 0.20208800
C -5.60497700 1.06321800 0.09160000
H -5.29635000 2.98119400 1.14549600
H -5.35124800 3.14642000 -0.61293000
H -6.23589900 0.75984000 0.94032700
H -6.18801100 0.89143400 -0.82495900
N -3.24042900 0.89713900 0.06430700
C -4.34869700 0.21939100 0.04619300
C -3.53371500 2.27023600 0.14385600
O -4.35017300 -1.06630500 -0.02208600
O -2.69042800 3.12780700 0.16782900
Si -5.61197000 -2.28598200 0.08334100
C -6.16013200 -2.31190200 1.86096200
H -6.66307300 -1.38188300 2.16296500
H -6.87922300 -3.13192900 2.01079800
H -5.31020500 -2.48485400 2.53651300
C -4.68874800 -3.81291200 -0.42639700
H -5.34457100 -4.69473300 -0.38086000
H -4.31628600 -3.71875600 -1.45661500
H -3.83266800 -3.98749600 0.24122400
C -6.97198300 -1.82788600 -1.10460100
H -7.61932700 -2.70665700 -1.25174900
H -7.61185100 -1.01564400 -0.73286200
H -6.57386400 -1.54765100 -2.09134600
H 3.78079500 -0.72448800 -1.56595100
N 3.43814700 1.68844200 -0.26803400
H 3.81587000 2.63745300 -0.21936800
C 2.07267200 1.58379400 -0.36188400
O 1.41507800 2.64073100 -0.46553300
Si 0.97812700 4.30293800 0.13643000
C -0.13354600 4.91290100 -1.19695000
H -1.05554400 4.31275400 -1.19409000
H -0.40999500 5.95907100 -0.99459900
H 0.34134100 4.87066600 -2.18679400
C 0.18575300 3.94447700 1.76589600
H -0.03081800 4.89461900 2.27922400
H -0.76831100 3.42428200 1.59480000
H 0.82877500 3.34498300 2.42637600
C 2.65076200 5.12208000 0.24753800
H 3.17367100 5.12919300 -0.72153200
H 2.52529600 6.17583100 0.54252200

H 3.28959300 4.66362600 1.01963500

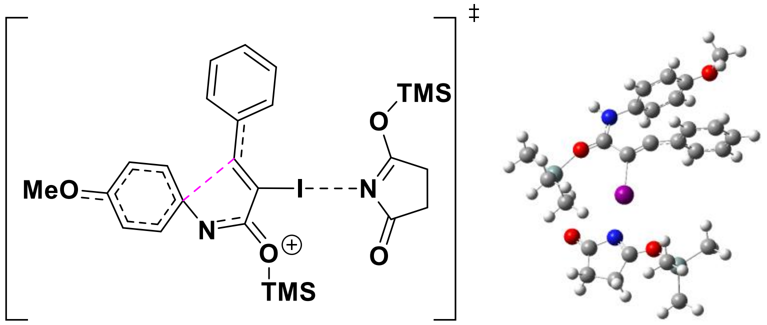

TS2

C -5.69032900 -0.95752000 0.10009000
C -5.27786000 -0.28210400 1.28170200
C -4.42836800 0.78996000 1.19792100
C -4.43733400 0.60467800 -1.24772200
C -5.29694700 -0.46905600 -1.17275900
H -5.66763100 -0.63930000 2.23537300
H -4.10975900 1.32421800 2.09543000
H -4.12452000 0.99725900 -2.21766600
H -5.67412800 -0.92638900 -2.08619500
C -3.90696900 1.17974200 -0.06633900
C -2.07656400 -0.08535800 0.03372400
C -2.42787700 -1.44600900 0.16020200
C -2.63513900 -2.23830900 -0.99271600
C -2.60688700 -2.00506800 1.44738400
C -2.99014900 -3.57249300 -0.85133100
H -2.49695700 -1.79716400 -1.98140700
C -2.96755200 -3.33843900 1.57209500
H -2.44705200 -1.38428800 2.33055700
C -3.16191600 -4.11690500 0.42624900
H -3.13112200 -4.19572300 -1.73524800
H -3.09794400 -3.77840700 2.56118700
H -3.44497300 -5.16587000 0.53078900
C -1.24535700 0.90804700 -0.03143000
O -6.44949900 -2.00923200 0.27744900
C -6.96985300 -2.72975400 -0.83602800
H -7.62450100 -2.08463800 -1.43856200
H -6.14850500 -3.12530000 -1.45119200
H -7.55160300 -3.55413200 -0.41519300
I 0.96373000 0.50019400 0.01270600
C 5.55016600 1.27630700 0.15280000
C 5.60961100 -0.25027300 0.17519900
H 5.99507200 1.71532200 -0.75113900
H 6.02689300 1.75899700 1.01547600
H 6.19000800 -0.69615700 -0.64585900

H 6.01262600 -0.65560000 1.11543100
N 3.33687500 0.37023100 0.07059100
C 4.15525000 -0.64725300 0.06272300
C 4.06069700 1.58657400 0.15610900
O 3.73118200 -1.85101500 -0.01508500
O 3.53114400 2.65801400 0.20979600
Si 4.54639600 -3.42109100 -0.16849300
C 5.20673100 -3.47238000 -1.90531600
H 6.00575100 -2.73769500 -2.08027900
H 5.63593500 -4.46798700 -2.09833800
H 4.40830100 -3.30498100 -2.64223200
C 3.13970800 -4.58916400 0.13287500
H 3.48477400 -5.62979800 0.04172500
H 2.72492100 -4.45649500 1.14221300
H 2.33662500 -4.43161500 -0.60091300
C 5.86614900 -3.50364700 1.14091100
H 6.18264400 -4.55347500 1.24609600
H 6.76425300 -2.92249300 0.88900300
H 5.49060900 -3.17967700 2.12312800
N -3.10434200 2.35429500 -0.15881100
H -3.53184900 3.27798600 -0.23331000
C -1.77327700 2.28403100 -0.13633900
O -1.09707500 3.33004600 -0.21028600
Si 0.36414900 4.41957900 -0.06005800
C 1.37168300 4.06422600 -1.56952400
H 0.80926200 4.31792800 -2.47952300
H 2.26792100 4.70348600 -1.53368300
H 1.71675700 3.02525200 -1.61846700
C 1.06751900 4.04750500 1.61039700
H 1.89765000 4.75210500 1.77964500
H 0.31896200 4.21807700 2.39773100
H 1.48081300 3.03637400 1.69045500
C -0.46774300 6.07493900 -0.12701600
H -0.99573700 6.22629400 -1.07931600
H -1.17851600 6.20922100 0.70084700
H 0.29157300 6.86770900 -0.04086000

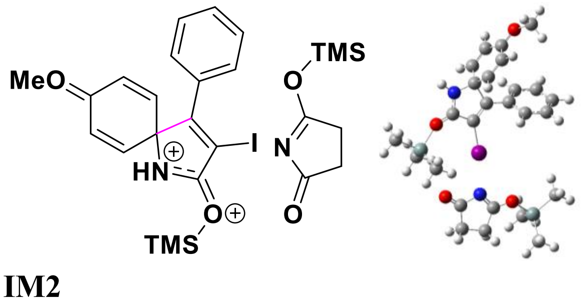

IM2

C 5.94995800 -1.18591800 -0.17020300

C 5.49771400 -0.60749600 -1.41718200
C 4.49285600 0.28678100 -1.40999200
C 4.45788000 0.20790100 1.09822500
C 5.44673300 -0.71142000 1.09654900
H 5.99655700 -0.93118300 -2.33161500
H 4.12320800 0.73246000 -2.33736600
H 4.06255500 0.60450100 2.03770400
H 5.87443400 -1.07209400 2.03111700
C 3.77144700 0.64411500 -0.15307800
C 2.38392900 -0.11043000 -0.12957900
C 2.30249000 -1.57946900 -0.07003500
C 2.66638900 -2.38939400 -1.15592900
C 1.83730300 -2.17337900 1.11379500
C 2.57275400 -3.77658200 -1.05211000
H 2.98176800 -1.93994600 -2.09859600
C 1.75655200 -3.55921800 1.21377800
H 1.53125300 -1.54360300 1.95211500
C 2.12755800 -4.36132300 0.13306800
H 2.83491600 -4.40167400 -1.90644200
H 1.39351200 -4.01501800 2.13559700
H 2.05563000 -5.44698800 0.21039600
C 1.39357200 0.80379700 -0.05916700
O 6.83546100 -2.09632900 -0.27811200
C 7.40960800 -2.79056100 0.85164300
H 7.97594100 -2.07881600 1.46483000
H 6.60877000 -3.27457000 1.42412900
H 8.07931700 -3.53711300 0.41877300
I -0.69489100 0.49632300 0.00432400
C -5.40043600 1.73329400 0.07941400
C -5.68883600 0.23585600 0.14077000
H -5.79871100 2.30373500 0.92822400
H -5.77060000 2.21399400 -0.83643200
H -6.17715100 -0.07786500 1.07502800
H -6.30314300 -0.14190200 -0.68989900
N -3.33364700 0.50466400 0.07269100
C -4.29899800 -0.36322400 0.08390900
C -3.87669800 1.80370200 0.08858400
O -4.06756400 -1.63124200 0.06470200
O -3.20447000 2.80028100 0.09815300
Si -5.10412700 -3.03933300 -0.09979200
C -6.48532000 -2.90868400 1.14553200
H -7.26655400 -2.19427300 0.85116300
H -6.96691900 -3.89491600 1.23655700
H -6.10937700 -2.63707500 2.14330100

C -3.92224700 -4.41927500 0.28064600
H -4.42127200 -5.39365100 0.17398900
H -3.06425500 -4.39686600 -0.40624200
H -3.54691600 -4.33991000 1.31095400
C -5.69708900 -3.04066500 -1.86486900
H -6.26763700 -3.96172300 -2.05977900
H -6.36102400 -2.19379200 -2.09044500
H -4.85041500 -3.01674400 -2.56588800
N 3.33871900 2.01949100 -0.11449600
H 3.96514800 2.82086300 -0.14171400
C 2.00405000 2.14934800 -0.06131400
O 1.47569300 3.27687600 -0.02492800
Si -0.01204600 4.36532700 0.03768900
C -0.81969800 3.98916300 1.65916200
H -0.12255000 4.15749600 2.49257800
H -1.65871100 4.69452200 1.77137800
H -1.24386800 2.98062000 1.70706800
C -0.91364100 4.03744900 -1.54382300
H -1.78729900 4.70830000 -1.56378600
H -0.28109100 4.27496400 -2.41113700
H -1.29796600 3.01429200 -1.61652500
C 0.84629100 6.01034600 0.03957900
H 1.49329100 6.13145700 0.91997700
H 1.44838200 6.15638500 -0.86844900
H 0.09326300 6.81303100 0.06960800

Cartesian coordinates of all optimized structures shown in Fig. S1. A-B

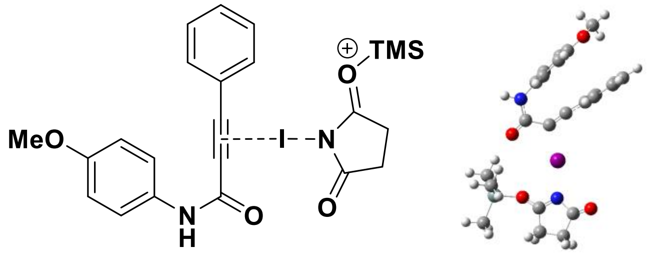

C -5.81803800 -1.49679000 -0.00667700
C -5.11476900 -1.34027000 1.20035400
C -3.78430000 -1.71449400 1.28772700
C -3.81406200 -2.38007800 -1.03058000
C -5.16004500 -2.02266900 -1.12590200
H -5.64942600 -0.93194900 2.05807500
H -3.24336000 -1.60583400 2.22936000
H -5.67908800 -2.15692600 -2.07334200
C -3.12224800 -2.23474400 0.16782100
C -1.63097100 0.76467600 0.30115200
C -2.41007700 1.96690800 0.26729600

C -1.90980500 3.19973900 0.71355300
C -3.72816200 1.86619600 -0.21231700
C -2.72815100 4.32344300 0.67910800
H -0.88697500 3.27209600 1.08676900
C -4.53565400 2.99747100 -0.23936600
H -4.10160800 0.89762700 -0.55187500
C -4.03708900 4.22337600 0.20467400
H -2.34416000 5.28318000 1.02611700
H -5.55952600 2.92204000 -0.60685800
H -4.67394400 5.10878500 0.18260400
C -1.10096900 -0.33555100 0.30471900
O -7.10494000 -1.11657700 0.00462200
C -7.87823000 -1.30167100 -1.15712300
H -7.48555800 -0.70810100 -1.99885800
H -7.91359000 -2.36392700 -1.44744300
H -8.88992800 -0.95962600 -0.91657900
I 1.35909400 1.08562500 -0.03869600
C 5.60779000 2.27082700 -0.50045300
C 5.69369600 0.74649100 -0.36904500
H 5.98550100 2.64922700 -1.45982400
H 6.13997200 2.81111300 0.29357700
H 6.13361500 0.24571500 -1.24372600
H 6.25614700 0.40759800 0.51339800
N 3.43190800 1.35123800 -0.25030200
C 4.25734400 0.31951900 -0.22731300
C 4.12656600 2.60213400 -0.40176000
O 3.81973200 -0.85060900 -0.10166800
O 3.57690000 3.64997700 -0.43276900
Si 4.18444000 -2.58188600 0.12299100
C 6.03531700 -2.73646400 -0.02138700
H 6.56572500 -2.19535400 0.77591700
H 6.30929800 -3.79888900 0.07429900
H 6.40713100 -2.39598700 -0.99910700
C 3.22252800 -3.35341200 -1.25406000
H 3.33527700 -4.44765000 -1.23048000
H 2.15917000 -3.10964600 -1.10964200
H 3.55103200 -2.99745400 -2.24063700
C 3.49382600 -2.90806600 1.80652800
H 3.60667400 -3.97241700 2.06217300
H 3.99510300 -2.31617500 2.58527400
H 2.42035500 -2.66541000 1.79310200
H -3.29530700 -2.77759200 -1.90440800
N -1.74407400 -2.60492900 0.24988900
H -1.48812300 -3.58908400 0.23145300

C -0.69167900 -1.75284500 0.32671600
O 0.46826800 -2.11323900 0.39488700

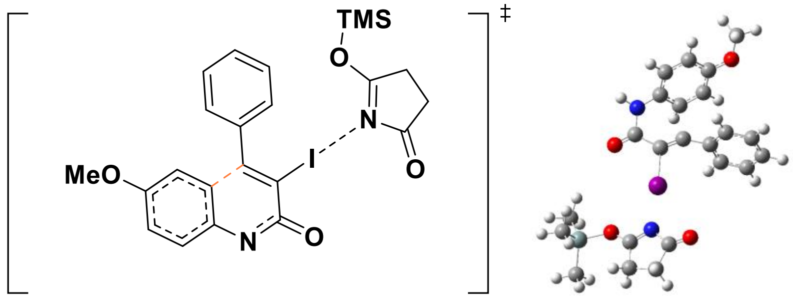

C -5.79839200 -1.05243500 0.21042000
C -5.42351700 -2.14996800 1.03678800
C -4.17859300 -2.75105400 0.91910000
C -3.62254700 -1.20266100 -0.88885900
C -4.90529600 -0.60962600 -0.75475100
H -6.11153300 -2.52210200 1.79593700
H -3.90529600 -3.57177400 1.58538500
H -5.20391800 0.20676600 -1.41324200
C -3.25956500 -2.29238300 -0.03844200
C -2.07491300 0.08485000 -0.40816800
C -2.56922300 1.42584800 -0.53439500
C -3.44441800 1.96332000 0.43216500
C -2.15615200 2.21750200 -1.62705800
C -3.87985900 3.27842400 0.31601600
H -3.76666100 1.34241300 1.26955800
C -2.60379700 3.53156500 -1.73744100
H -1.47347700 1.79726800 -2.36744600
C -3.46299600 4.06194900 -0.76900800
H -4.54555200 3.70063300 1.07127800
H -2.27773100 4.14746100 -2.57781300
H -3.81044300 5.09358700 -0.85792300
C -0.97560000 -0.61007800 -0.30335100
O -6.98043300 -0.42278100 0.29258500
C -7.97441200 -0.88621100 1.19321300
H -8.25980700 -1.92964900 0.97658400
H -7.64030600 -0.80540900 2.24171200
H -8.84662200 -0.23836200 1.04550900
I 0.90707600 0.50922000 0.05660300
C 5.09627700 2.80211300 0.99468400
C 5.54422000 1.39967300 0.56547200
H 5.49848200 3.60550100 0.36022200
H 5.35626700 3.05205900 2.03366200
H 6.21044600 1.38784300 -0.31135300
H 6.05242100 0.82974700 1.36004900
N 3.18416300 1.48768600 0.38162200

C 4.23192000 0.73136600 0.21727200
C 3.56692900 2.77244500 0.85504600
O 4.11744700 -0.47509300 -0.22015100
O 2.79658300 3.65747000 1.09672500
Si 5.01150400 -1.97599600 -0.25940100
C 6.64638800 -1.64399800 -1.10318300
H 7.30241800 -0.99515900 -0.50216200
H 7.18163400 -2.59466400 -1.26405800
H 6.49967800 -1.17650300 -2.09004600
C 3.86595600 -3.07130300 -1.23068000
H 4.21491600 -4.11651500 -1.21527700
H 2.84745400 -3.03237000 -0.81198000
H 3.81232000 -2.74650900 -2.28189200
C 5.20620200 -2.47897600 1.52958000
H 5.70102400 -3.46193400 1.59695800
H 5.82062300 -1.76105200 2.09620800
H 4.22429100 -2.56101200 2.02181900
H -3.15567000 -1.15638000 -1.87667200
N -1.99518600 -2.82546800 -0.14656200
H -1.82629300 -3.76561000 0.20457000
C -0.81264900 -2.09630500 -0.34794300
O 0.24723000 -2.66370600 -0.45193400

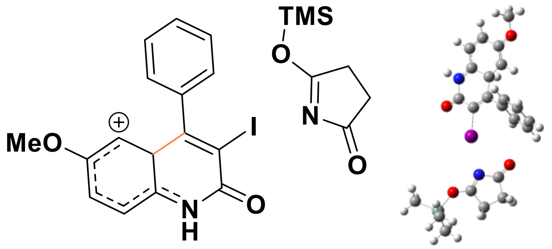

C 6.23183400 -0.22131600 -0.22110600
C 6.42235800 -1.63922400 -0.38286800
C 5.39252700 -2.54532000 -0.26343700
C 3.87261900 -0.68749000 0.44246600
C 4.99106900 0.24137100 0.12533200
H 7.41268700 -2.02018700 -0.63639200
H 5.56717200 -3.60471100 -0.46233000
H 4.83586600 1.30897600 0.27762500
C 4.08409200 -2.09719000 0.02743200
C 2.44292300 -0.16674300 0.31535200
C 2.26012800 1.28678900 0.54571300
C 2.70451200 1.86744700 1.74815200
C 1.62697900 2.09696800 -0.41112000
C 2.50330400 3.22599900 1.99282000
H 3.18236000 1.25126300 2.51513700
C 1.42587900 3.45469900 -0.16239200

H 1.27070900 1.66424500 -1.34533500
C 1.86198800 4.02117100 1.03761600
H 2.83586300 3.66151500 2.93747100
H 0.89964000 4.05822500 -0.90346200
H 1.69600000 5.08257900 1.23427600
C 1.41776600 -1.03771200 0.10234800
O 7.21387400 0.67498500 -0.39642500
C 8.49198400 0.26838100 -0.86441500
H 8.99407200 -0.39928800 -0.14370300
H 8.42399400 -0.22656300 -1.84812600
H 9.08252400 1.18629000 -0.96735700
I -0.61306900 -0.40739800 -0.07852300
C -4.20596100 2.90991100 -1.11732600
C -5.18498800 1.81832600 -0.67737300
H -4.25059200 3.82043900 -0.50197200
H -4.33335400 3.22257500 -2.16426000
H -5.78664000 2.08842200 0.20416600
H -5.89054200 1.50790700 -1.46378500
N -2.98791100 0.92793500 -0.48237000
C -4.25020400 0.67295700 -0.33055400
C -2.82677600 2.25204800 -0.95179600
O -4.66073900 -0.48400200 0.09657500
O -1.76171900 2.76531700 -1.18274700
Si -6.20481000 -1.25312800 0.34322500
C -7.19854700 -0.20349300 1.53176300
H -7.54951800 0.73568100 1.07737300
H -8.09095900 -0.76366800 1.85735500
H -6.61346300 0.04027700 2.43299400
C -5.71825600 -2.89467900 1.07335500
H -6.61029100 -3.51162600 1.26891200
H -5.06038200 -3.45048600 0.38707100
H -5.18174200 -2.75780400 2.02537500
C -7.01637800 -1.41219100 -1.33413700
H -7.93121300 -2.02208200 -1.25031000
H -7.30945000 -0.43691000 -1.75280400
H -6.34590600 -1.91157300 -2.05146200
H 4.00623600 -0.80302400 1.55486600
N 3.02655000 -2.89913800 -0.02288700
H 3.14800800 -3.89745000 -0.19921800
C 1.64393600 -2.48992500 -0.00296800
O 0.81505000 -3.35797200 -0.08313600

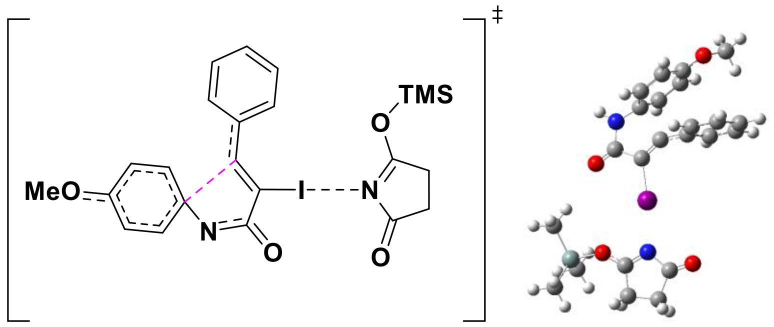

C -5.58290900 0.93282600 -0.06302100
C -4.99514700 1.18671800 1.20580400
C -3.76580800 1.79945400 1.26451200
C -3.73994300 2.02431900 -1.18412500
C -4.96629100 1.41946200 -1.25088100
H -5.50393400 0.89506900 2.12284200
H -3.28495900 2.00106300 2.22379500
H -3.23916900 2.39112500 -2.08208100
H -5.49427500 1.27907600 -2.19463700
C -3.03231000 2.10312800 0.06769200
C -2.04628800 0.35875500 -0.04308800
C -2.87587900 -0.80237500 -0.16815800
C -3.28553600 -1.25072700 -1.44240700
C -3.30990600 -1.49815900 0.98124500
C -4.09934800 -2.37409400 -1.56172800
H -2.94463700 -0.71748300 -2.33089000
C -4.11994800 -2.62389800 0.85385400
H -2.98689000 -1.15575700 1.96563300
C -4.52124300 -3.05715200 -0.41555400
H -4.40301800 -2.72331300 -2.55047200
H -4.43338600 -3.17226600 1.74471200
H -5.15753900 -3.93966300 -0.51286300
C -0.81843600 0.80911800 0.02713300
O -6.70891200 0.27287000 -0.23412000
C -7.41069300 -0.29218100 0.87682600
H -6.76602100 -1.00453000 1.41360500
H -7.76156900 0.49746000 1.55864400
H -8.27164900 -0.81812000 0.44997800
I 0.92479600 -0.46471100 -0.04019100
C 5.03376200 -3.20835600 0.26801500
C 5.55212200 -1.77628000 0.09422800
H 5.30869000 -3.66867500 1.22805900
H 5.36744300 -3.89397100 -0.52493200
H 6.09538300 -1.39016800 0.97202300
H 6.21129900 -1.63567600 -0.77644700
N 3.18411000 -1.70442800 -0.04244800

C 4.27065100 -0.99256000 -0.09916900
C 3.50481500 -3.06692400 0.19488800
O 4.22178400 0.28047300 -0.31761000
O 2.69150000 -3.93947700 0.31802900
Si 5.20295600 1.69811900 -0.05826200
C 5.47037300 1.79733400 1.78932700
H 6.05151300 0.94237400 2.17033500
H 6.02817600 2.71397000 2.04281300
H 4.50754600 1.82981100 2.32333200
C 4.11645900 3.04910400 -0.73092100
H 4.55855200 4.03956300 -0.53535700
H 3.99090900 2.94231300 -1.82013900
H 3.11692900 3.01327100 -0.26831600
C 6.79834300 1.47200800 -1.00769100
H 7.38588200 2.40468000 -0.97643100
H 7.42697800 0.67304400 -0.58451500
H 6.60201600 1.23894700 -2.06647500
N -1.86486400 2.90324500 0.15905400
H -1.91740300 3.91429700 0.23807100
C -0.62024200 2.29926000 0.15471100
O 0.43103200 2.88053400 0.23748600

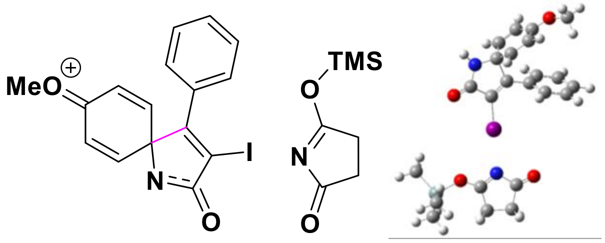

C -5.86321300 -0.73776400 -0.02336600
C -5.33907700 -1.36495800 -1.20823600
C -4.12245000 -1.94842500 -1.16089200
C -4.02185000 -1.52651300 1.29062400
C -5.22640700 -0.90277600 1.25051800
H -5.94080000 -1.31326600 -2.11561700
H -3.67064800 -2.40637500 -2.04430000
H -3.49503100 -1.68151500 2.23604500
H -5.70157000 -0.53972000 2.16008400
C -3.28653800 -1.89834700 0.06151300
C -2.29474200 -0.62982100 -0.10618400
C -2.81602800 0.74042700 -0.26258200
C -3.31618900 1.19937200 -1.48870400
C -2.79616500 1.60920500 0.83885800
C -3.79902100 2.50198200 -1.60701900
H -3.30374800 0.54223700 -2.36011800
C -3.27864700 2.90926200 0.71775100

H -2.37878100 1.26287000 1.78722500
C -3.78684200 3.35436800 -0.50403400
H -4.17114500 2.85637600 -2.56909000
H -3.24250600 3.58279800 1.57476800
H -4.15677200 4.37598600 -0.60124600
C -1.04173100 -1.08033800 0.01871900
O -6.94732500 -0.06262900 -0.18859500
C -7.55635700 0.67440000 0.88071800
H -7.93382100 -0.01781700 1.64434800
H -6.82707900 1.37705200 1.30497600
H -8.38691100 1.21782300 0.42371500
I 0.72716500 0.03234800 -0.02965600
C 4.61749700 3.28053200 0.36108100
C 5.39350200 1.98929400 0.11958200
H 4.81246500 4.06352600 -0.38299900
H 4.78946100 3.72122900 1.35207200
H 6.00738700 2.00466400 -0.79297400
H 6.05290800 1.69759000 0.95118600
N 3.08097200 1.45434000 0.02239100
C 4.27563100 0.97739600 -0.04293000
C 3.14987700 2.84636200 0.26099300
O 4.48998300 -0.28670600 -0.25343300
O 2.19297000 3.55314500 0.37315600
Si 5.87032200 -1.32266000 -0.10747100
C 7.29020900 -0.58302000 -1.06945000
H 7.74123700 0.28237500 -0.56415200
H 8.07859700 -1.34250600 -1.18791800
H 6.97293700 -0.27824500 -2.07798300
C 5.25434000 -2.90888900 -0.84708800
H 6.00428700 -3.70710700 -0.74699100
H 4.33313200 -3.23180900 -0.34058100
H 5.02959300 -2.77979200 -1.91545400
C 6.24077500 -1.46099800 1.71544200
H 7.04349600 -2.19546100 1.88131500
H 6.57318600 -0.50557400 2.14737400
H 5.35221600 -1.80067700 2.26701400
N -2.35134800 -2.96507400 0.21181300
H -2.62043000 -3.92965600 0.36810800
C -1.01916900 -2.57164100 0.22834400
O -0.07784300 -3.29027900 0.38089600

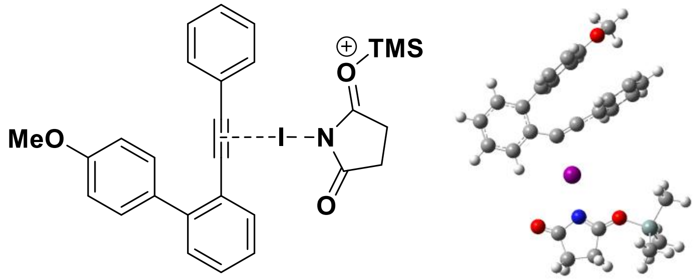

C -5.34346200 -1.22941800 -0.14583300
C -5.06217800 -0.61117200 1.08621200
C -4.38725300 0.60710100 1.10523900
C -4.33096700 0.63899900 -1.31241800
C -4.99533500 -0.57663000 -1.34639600
H -5.35059500 -1.07910400 2.02622900
H -4.15059500 1.07612000 2.06285600
H -4.05248400 1.13398100 -2.24525300
H -5.25785500 -1.05846500 -2.28922000
C -3.98048100 1.23840200 -0.08381000
C -1.64870700 -0.05327000 -0.02329400
C -1.99727000 -1.40600200 -0.05747600
C -2.23710900 -2.04913700 -1.30046400
C -2.21027000 -2.12426000 1.14820700
C -2.66536100 -3.36991100 -1.33026300
H -2.08319000 -1.48853300 -2.22282700
C -2.63137600 -3.44696300 1.10390100
H -2.03950600 -1.62028100 2.10037700
C -2.86631100 -4.06571900 -0.13196400
H -2.85592400 -3.86098400 -2.28616400
H -2.78680100 -4.00133600 2.03161800
H -3.21023400 -5.10218800 -0.16088300
C -1.17095700 1.13105200 -0.00778600
O -5.92416500 -2.43460800 -0.27491200
C -6.40196900 -3.10723900 0.87696500
H -5.58126500 -3.34332900 1.57487900
H -7.16504500 -2.50884500 1.40219700
H -6.85567200 -4.04118300 0.52339600
I 1.09641700 0.91962900 0.00555900
N 3.54869400 1.15378900 0.03323600
C 4.10193100 2.46300900 0.10313200
C 4.49136400 0.25641500 -0.00434400
C 5.62879000 2.34383300 0.12837200
O 3.44257400 3.46338100 0.13896900
C 5.89044900 0.83674300 0.02313100
O 4.21994300 -1.00376500 -0.06867800
H 6.00566400 2.79068100 1.06034400

H 6.05214600 2.92707900 -0.70206800
H 6.45535300 0.41820200 0.87060000
H 6.42508200 0.54435800 -0.89383300
Si 5.15100100 -2.49379900 -0.02107500
C 6.45609900 -2.40103200 -1.35530600
C 3.84337000 -3.77083600 -0.36718800
C 5.86515100 -2.60794000 1.70076400
H 7.25981400 -1.68961600 -1.11229500
H 6.92352400 -3.39277200 -1.47498600
H 6.02015500 -2.12269800 -2.32810200
H 4.27878000 -4.78291200 -0.34151800
H 3.03944700 -3.72460100 0.38398100
H 3.39552600 -3.61744800 -1.36157100
H 6.36104700 -3.58383600 1.83392700
H 6.61916800 -1.82930500 1.89578100
H 5.07562100 -2.52697300 2.46454500
C -3.20606500 2.50766100 -0.04550600
C -1.78661500 2.45934500 -0.00434800
C -3.09132100 4.93473300 -0.01182700
H -3.60286900 5.89967100 -0.01545900
C -1.69688400 4.88081800 0.02955600
H -1.11080300 5.80143000 0.05875800
C -1.04389900 3.64763400 0.03288100
C -3.83945400 3.75325500 -0.04896600
H -4.93004400 3.79489400 -0.08135200
H 0.04629100 3.60741100 0.06376200

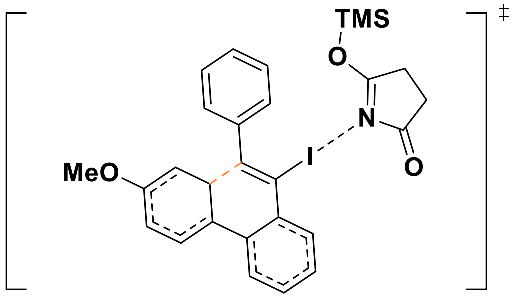

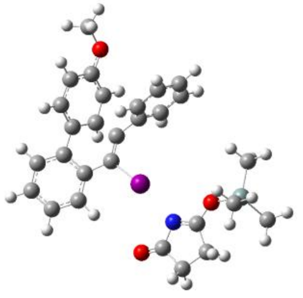

C -5.15792100 -1.77623000 -0.29423900
C -5.52890800 -0.76360900 -1.21744400
C -5.03742200 0.53445700 -1.10099700
C -3.75663000 -0.13813500 0.85077300
C -4.29793600 -1.44563900 0.74643200
H -6.19993200 -0.99722400 -2.04419200
H -5.32278200 1.28328800 -1.84184500
H -4.02885800 -2.21640200 1.46974900
C -4.15604300 0.88272800 -0.07000000
C -3.56292000 2.21517200 0.04497200
C -2.16575700 2.33393700 0.29900600

C -4.33415300 3.37406500 -0.12729000
C -1.60095100 3.61308600 0.39532900
C -3.75543400 4.63854700 -0.01501400
H -5.40590300 3.27955900 -0.31312800
C -2.38952100 4.75601600 0.25296200
H -0.52829200 3.71251900 0.56644500
H -4.37267700 5.53128400 -0.13378100
H -1.92853000 5.74194800 0.33831200
C -1.77090400 -0.12911800 0.48422900
C -1.28555300 -1.47699000 0.66219300
C -1.36088300 -2.40118000 -0.39840300
C -0.73887600 -1.87608100 1.89751900
C -0.87876200 -3.69601700 -0.22919000
H -1.79430000 -2.08731900 -1.34974000
C -0.26531100 -3.17668800 2.06213200
H -0.68040100 -1.15472100 2.71480200
C -0.33475600 -4.08608600 1.00132100
H -0.92875300 -4.40842800 -1.05530000
H 0.15995000 -3.48316900 3.02016600
H 0.03639500 -5.10484200 1.13288800
C -1.35856000 1.11059500 0.36325200
O -5.59418000 -3.04415000 -0.34991800
C -6.53947500 -3.42675600 -1.33611100
H -7.47200100 -2.84380900 -1.24834200
H -6.12985500 -3.31363700 -2.35443200
H -6.75997500 -4.48547100 -1.15469200
I 0.84114400 1.22477900 0.11445300
C 5.54827700 2.49086200 -0.39686500
C 5.67002700 0.96274600 -0.38793400
H 5.88180500 2.95302800 -1.33771200
H 6.09869200 2.98460100 0.41705000
H 6.09993900 0.54133400 -1.31020200
H 6.26412100 0.56916400 0.45150400
N 3.37377900 1.49765500 -0.15006600
C 4.22722100 0.52386400 -0.24001500
C 4.04467200 2.74785600 -0.22010300
O 3.83994500 -0.71221200 -0.19451700
O 3.49250300 3.80973600 -0.15044000
Si 4.57952500 -2.28526500 -0.34789900
C 5.19450500 -2.42881900 -2.10641600
H 6.03350500 -1.74623500 -2.31392300
H 5.55249600 -3.45459300 -2.29496900
H 4.38836000 -2.21697100 -2.82658600
C 3.14478200 -3.41397100 0.02274300

H 3.45299600 -4.47038700 -0.03625100
H 2.74937600 -3.22622400 1.03287300
H 2.32345300 -3.25357100 -0.69253600
C 5.94955400 -2.41110300 0.91840900
H 6.30177700 -3.45469000 0.97416400
H 6.81999100 -1.78651700 0.66471100
H 5.59453400 -2.12699500 1.92197100
H -3.43587600 0.17925200 1.84955700

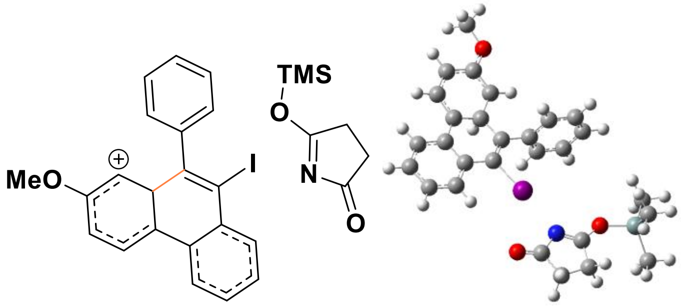

C -5.48122000 -1.85301600 -0.20682800
C -6.25565800 -0.66351200 -0.38321200
C -5.69829300 0.59691000 -0.27852500
C -3.52152600 -0.41029200 0.30482500
C -4.14259400 -1.72980400 0.08034800
H -7.31759500 -0.74057100 -0.61811800
H -6.34180000 1.45546500 -0.46448000
H -3.54143500 -2.62685200 0.22505300
C -4.33162100 0.79471400 0.00558600
C -3.70050400 2.07965700 0.05493400
C -2.26517700 2.16463700 0.12091200
C -4.46524100 3.27678900 0.00930300
C -1.67767600 3.44808200 0.13605100
C -3.85627800 4.51501200 0.02896500
H -5.55307000 3.22532600 -0.02067600
C -2.45189600 4.59751900 0.09194000
H -0.59196000 3.53172200 0.18201800
H -4.46036400 5.42377500 0.00284700
H -1.96358100 5.57424400 0.10834300
C -2.01779000 -0.28402300 0.22898500
C -1.24124100 -1.54704800 0.34246600
C -1.00208700 -2.32530500 -0.80094600
C -0.76355000 -1.98470100 1.58633300
C -0.28646200 -3.51962700 -0.70046500
H -1.36769100 -1.98435100 -1.77250400
C -0.05643200 -3.18484400 1.68551400
H -0.93527300 -1.37390800 2.47583600
C 0.18354300 -3.95309300 0.54317900

H -0.09658400 -4.11470700 -1.59654300
H 0.31231200 -3.51777500 2.65822700
H 0.73953000 -4.88968200 0.62127000
C -1.45063500 0.95406100 0.15564200
O -5.98190900 -3.09096500 -0.32119700
C -7.34329300 -3.28561500 -0.67704900
H -8.02327300 -2.85968600 0.07996300
H -7.56820100 -2.85045900 -1.66542500
H -7.49319800 -4.37074100 -0.72123300
I 0.69369700 1.11656300 0.05496600
C 5.46220900 2.74110200 -0.22888300
C 5.76281900 1.23979900 -0.17694100
H 5.78447900 3.22191100 -1.16444900
H 5.90836700 3.31348900 0.59723300
H 6.29515000 0.85635000 -1.06155000
H 6.34747600 0.93544600 0.70524000
N 3.40260700 1.49885500 -0.07124800
C 4.37039400 0.64023500 -0.09997100
C 3.92901000 2.81180500 -0.13041100
O 4.13645500 -0.63997200 -0.05463300
O 3.25569600 3.80728100 -0.10866200
Si 5.07762400 -2.09393400 -0.18537900
C 5.76802600 -2.15277700 -1.92249600
H 6.51609000 -1.36453000 -2.10136500
H 6.26364400 -3.12193900 -2.09842500
H 4.96593500 -2.04479200 -2.66982900
C 3.79912800 -3.41213700 0.13134300
H 4.24558800 -4.41698000 0.05792800
H 3.36308600 -3.30001300 1.13620500
H 2.97798100 -3.34147300 -0.59846100
C 6.41590800 -2.05636300 1.12140300
H 6.91373800 -3.03917400 1.16980900
H 7.19165800 -1.30644700 0.90373700
H 5.99641200 -1.84642500 2.11831400
H -3.61145700 -0.38173500 1.43582600

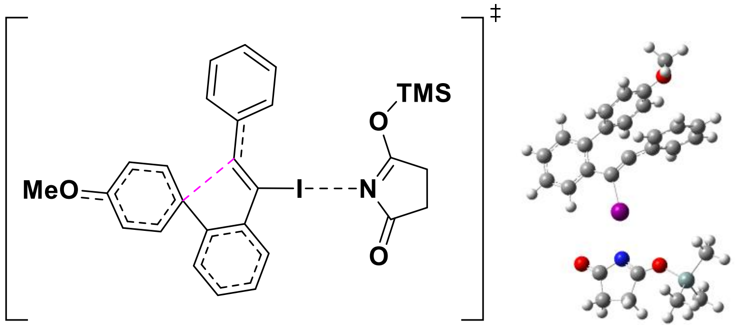

C -5.31965500 -1.40480900 0.12525000

C -5.01142400 -0.75967000 1.35412900
C -4.32899000 0.43016800 1.34947000
C -4.33867900 0.42439100 -1.10197800
C -5.02472000 -0.77095000 -1.10796800
H -5.31939500 -1.24846000 2.27897700
H -4.07331600 0.92898800 2.28637700
H -4.09171400 0.92182700 -2.04219100
H -5.32046400 -1.22399100 -2.05236900
C -3.86546200 1.01014400 0.11813000
C -3.31098400 2.39614100 0.10085300
C -1.90233000 2.46933100 0.03800400
C -4.07310300 3.56259600 0.13402200
C -1.25632100 3.70811900 0.00549400
C -3.42318600 4.80359300 0.10344600
H -5.16287100 3.51052700 0.18336500
C -2.02785200 4.87354900 0.03833000
H -0.16624200 3.75771500 -0.04321100
H -4.01330500 5.72203900 0.13064700
H -1.53339300 5.84673500 0.01421100
C -2.04785500 0.06945300 0.05100700
C -2.10204700 -1.36027000 0.06175900
C -2.14920200 -2.08512000 -1.14986300
C -2.15767400 -2.06587500 1.28377500
C -2.22870700 -3.47545900 -1.13619300
H -2.10778700 -1.54041300 -2.09439300
C -2.24279100 -3.45590300 1.29188300
H -2.12151300 -1.50758500 2.22012000
C -2.28386600 -4.16056800 0.08381500
H -2.24522800 -4.02967700 -2.07718900
H -2.27792000 -3.99397500 2.24125400
H -2.35253800 -5.25083900 0.09285100
C -1.28000300 1.14274400 0.02105800
O -5.89161200 -2.59176500 0.22851000
C -6.22729400 -3.34393600 -0.93711700
H -6.98621200 -2.81881700 -1.53774400
H -5.32908800 -3.53619400 -1.54390400
H -6.63964700 -4.29226300 -0.57477800
I 0.86745700 0.93379600 -0.03323800
C 5.52584600 2.51678800 -0.19879700
C 5.89361300 1.03034100 -0.19480700
H 5.87757600 3.06049500 -1.08743600
H 5.89614500 3.06377200 0.68097800
H 6.41532200 0.70282700 -1.10734700
H 6.51806000 0.72394000 0.65892000

N 3.52544800 1.17705400 -0.11984200
C 4.53306200 0.36128000 -0.11967800
C 3.99067400 2.51587900 -0.17645900
O 4.36426800 -0.92671200 -0.07111200
O 3.27081200 3.47769000 -0.19708700
Si 5.41907000 -2.30420000 0.10901700
C 6.72237800 -2.24301800 -1.23180100
H 7.46669800 -1.45045200 -1.06251900
H 7.26517200 -3.20275800 -1.25537800
H 6.27042100 -2.09429000 -2.22559400
C 4.24070700 -3.73022300 -0.10606200
H 4.77035700 -4.69019700 0.00529800
H 3.43740900 -3.69203300 0.64622300
H 3.77728800 -3.70956400 -1.10499200
C 6.14301000 -2.20863300 1.82987700
H 6.72665300 -3.11978400 2.04209300
H 6.82037500 -1.34910100 1.95312900
H 5.34820100 -2.13445200 2.58891900

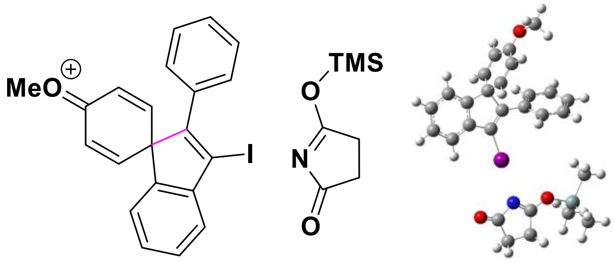

C -5.36122200 -1.55778300 0.08151000
C -4.88450800 -0.98513900 -1.13398700
C -4.02871300 0.07795700 -1.06618000
C -4.12081400 0.02656900 1.41715900
C -4.99638500 -1.00659300 1.35743300
H -5.20323300 -1.37887900 -2.09767000
H -3.65504800 0.54864800 -1.97827700
H -5.43672300 -1.45924600 2.24681400
C -3.49170800 0.59530300 0.20690800
C -3.37208500 2.11354800 0.21073900
C -2.01811200 2.48826700 0.09528900
C -4.38587300 3.06075100 0.29333000
C -1.66490300 3.83900300 0.06191800
C -4.02540900 4.41562700 0.26615700
H -5.43411500 2.76583100 0.38241900
C -2.68170100 4.79594700 0.15002400
H -0.61722700 4.13243200 -0.02978300
H -4.80056500 5.18132700 0.33785600
H -2.42400000 5.85691200 0.12935300
C -1.94771800 0.15241500 0.12195100

C -1.55446500 -1.26458100 0.03216100
C -0.96287600 -1.75037700 -1.14933700
C -1.74808100 -2.15374700 1.10352700
C -0.58049800 -3.08692700 -1.25531200
H -0.79721000 -1.06470900 -1.98247600
C -1.36138500 -3.49198200 0.99748400
H -2.17690200 -1.79013200 2.03856500
C -0.78093200 -3.96236000 -0.18305200
H -0.11919400 -3.44692300 -2.17767900
H -1.50577000 -4.16658500 1.84438500
H -0.47671400 -5.00816500 -0.26507800
C -1.19300100 1.27913500 0.03770800
O -6.15959700 -2.58656000 0.13302300
C -6.61630200 -3.26192600 -1.05112100
H -7.21300900 -2.57899000 -1.67306000
H -5.75981600 -3.65474500 -1.61802500
H -7.24223300 -4.08697300 -0.69540900
I 0.92595400 1.32634800 -0.08720500
C 5.95925300 2.15681100 -0.24248100
C 5.91705400 0.62835500 -0.15393600
H 6.46930300 2.53437500 -1.14068000
H 6.43623500 2.63742300 0.62432900
H 6.38918900 0.11890900 -1.00849200
H 6.38076700 0.22036600 0.75838300
N 3.66822900 1.40032900 -0.23061200
C 4.42461900 0.35348500 -0.15151400
C 4.47648900 2.56455300 -0.29202600
O 3.91344700 -0.84432100 -0.08858100
O 4.04407100 3.68196700 -0.36620100
Si 4.48594900 -2.44054300 0.27189700
C 5.83738000 -2.88402700 -0.94360200
H 6.76186700 -2.31172700 -0.77130800
H 6.08940100 -3.95308200 -0.84461200
H 5.51098000 -2.71396100 -1.98213700
C 2.94517800 -3.46296300 0.03400000
H 2.60125000 -3.40873400 -1.01061000
H 3.13149400 -4.52087800 0.28010900
H 2.12794800 -3.09540300 0.67379900
C 5.08535200 -2.42268900 2.04324400
H 5.34441900 -3.44488600 2.36540300
H 5.98472000 -1.79991800 2.17212200
H 4.30306100 -2.04395200 2.72004600
H -3.82051900 0.45199700 2.37740100

General Information.

All commercial reagents and solvents were used without further purification. Crushed 4Å molecular sieves were activated through flame-drying under high vacuum immediately prior to use. All reactions were monitored by thin-layer chromatography (TLC). The TLC plates were visualized with UV light and/or by staining with $MeOH/H_2SO_4$ (10%, v/v). Flash column chromatography was performed on Silica Gel 60 (200-300 mesh). NMR spectra were measured on Bruker AVANCE III 400 or 600 MHz NMR spectrometer. Chemical shifts are reported in parts per million (ppm) as values relative to the internal chloroform (7.26 ppm for ^{1}H NMR and 77.16 ppm for ^{13}C NMR). Multiplicities for ^{1}H NMR signals are described using the following abbreviations: s = singlet, d = doublet, t = triplet, q = quartet, m = multiplet; *J* = coupling constant in Hz, integration. High-resolution mass spectra (HRMS) were recorded on an Agilent 6230 mass spectrometer (ESI (electrospray ionization), positive ion mode). Optical rotations were measured on an Autopol IV (Serial #83493), using $CHCl_3$ and CH_2Cl_2 as solvent. Single crystal X-ray data were collected on Bruker D8 Venture X-ray Single Crystal Diffractometer using Cu Kα radiation (λ = 1.54178 Å).

Preparation of PPAP glycosides 1a-r.

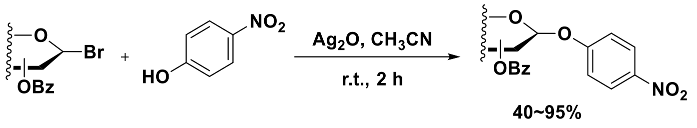

General procedure A. To a solution of perbenzoylated glycosyl bromide (1.0 eq.) and 4-nitrophenol (1.5 eq.) in anhydrous CH_3CN (0.2 M) was added Ag_2O (1.5 eq.). The mixture was stirred at room temperature for 2~3 h under N_2. The reaction was then filtered through a pad of celite, and the filtrate was concentrated under reduced pressure to give a residue. The residue was dissolved in EtOAc, washed with saturated $NaHCO_3$, and brine successively. The organic layer was dried over anhydrous Na_2SO_4, filtered, and concentrated in vacuo. The residue was purified by flash column chromatography on silica gel to afford 4-nitrophenyl perbenzoylated glycosides.

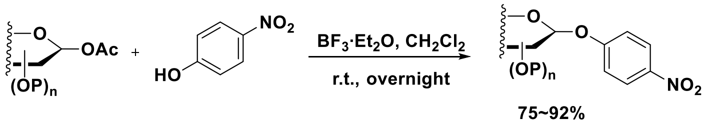

General Procedure B: A mixture of glycosyl acetate (1.0 eq.), 4-nitrophenol (1.5 eq.) and 4 Å molecular sieve in anhydrous CH_2Cl_2 was cooled at 0 °C under N_2. To the reaction, $BF_3{\cdot}Et_2O$ (2.5 eq.) was added dropwise over 20 min. The reaction was slowly warmed to room temperature and stirred overnight before being quenched by the addition of aqueous sat. $NaHCO_3$. The reaction was extracted three times with CH_2Cl_2. The combined organic layers were washed with brine, dried over Na_2SO_4 and filtered. The filtration was concentrated in vacuum and the crude product was purified by flash column chromatography on silica gel to afford the desired product.

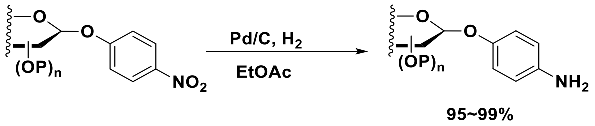

General Procedure C: A suspension of 4-nitrophenyl glycosides (1.0 eq.) and Pd/C (10%, 0.05 eq.) in EtOAc was degased and charged with H_2 at 1 atm for 3~6 h at room temperature until TLC-

analysis indicated the reaction to be complete. After filtration, the filtrate was evaporated under vacuum to afford the amine.

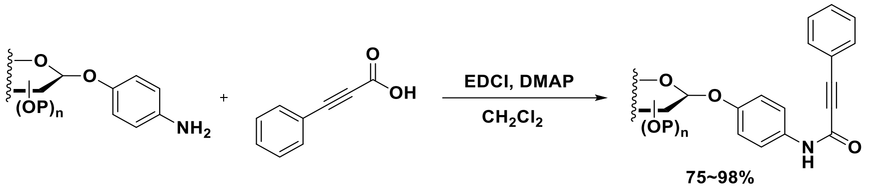

General procedure D. To a solution of amine (1.0 eq.) and phenylpropiolic acid (1.3 eq.) in anhydrous CH_2Cl_2 (0.1 M) was added 4-dimethylaminopyridine (DMAP) (0.1 eq.), *N*-(3-dimethylaminopropyl)-*N'*-ethylcarbodiimide hydrochloride (EDCI) (1.5 eq.). The resulting mixture was stirred at room temperature until TLC-analysis indicated the reaction to be complete. The reaction mixture was diluted with EtOAC and washed with brine. The organic layer was concentrated in *vacuo*, and the residue was purified by flash column chromatography on silica gel to afford the glycosyl PPAP donors.

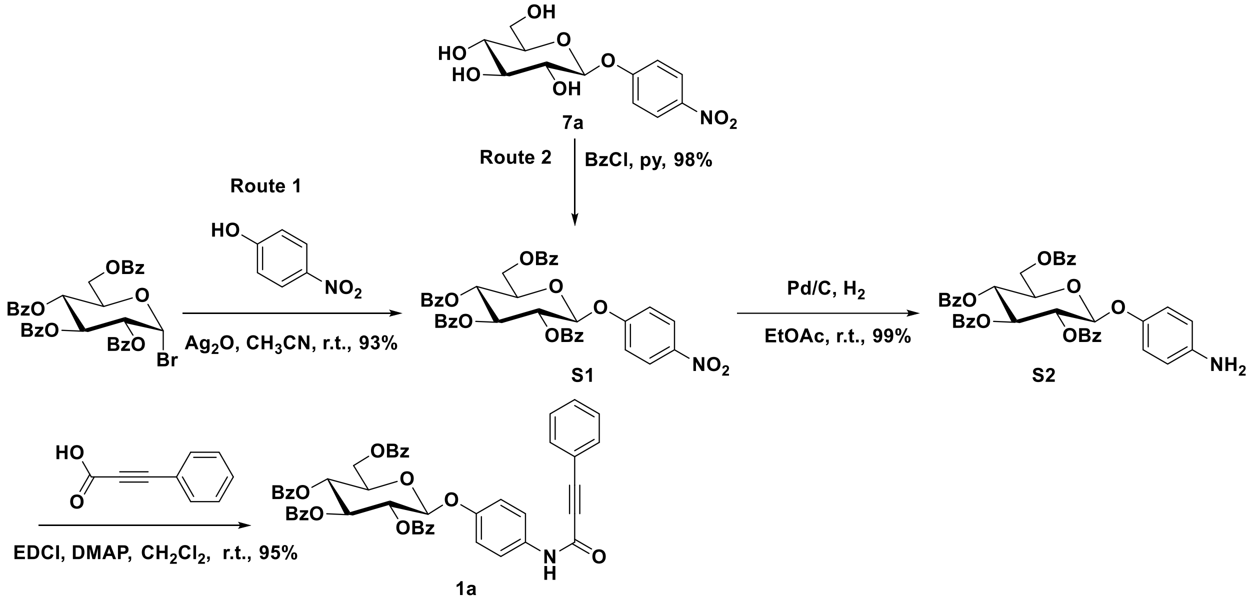

Fig. S2. Synthesis of the glycosyl PPAP donor **1a**.

4-nitrophenyl 2,3,4,6-tetra-*O*-benzoyl-β-D-glucopyranoside (S1) (*58*)

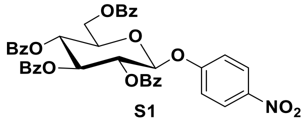

The one route of compound **S1**(*58*) was prepared according to **General procedure A**: To a solution of perbenzoylated glucosyl bromide (3.3 g, 5.0 mmol) and 4-nitrophenol (1.04 g, 7.5 mmol) in dry CH_3CN (25 mL) was added Ag_2O (1.74 g, 7.5 mmol). The mixture was stirred at room temperature for 2~3 hours under N_2. Filtration through a pad of Celite, and the filtrate was concentrated under reduced pressure to give a residue. The residue was washed with EtOAc, saturated $NaHCO_3$, and brine successively, and then dried over Na_2SO_4. Filtration was followed by concentration to yield a residue which was further purified by silica gel chromatography (Petroleum ether/EtOAc = 3:1) to afford **S1**(*58*) (3.3 g, 93%) as a white foam. The another route of compound **S1:** To a solution of **7a** (1.5 g, 5.0 mmol) in pyridine (20 mL) was added benzoyl chloride (3.5 mL, 30.0 mmol) at 0 °C. The reaction mixture was stirred at room temperature overnight and monitored by TLC. The mixture was cooled using an ice-bath, quenched by addition of MeOH (7.5 mL), and then

concentrated in *vacuo*. EtOAc was added to form a pyridine salt and filtered, then the resultant solution was washed with water, 1M HCl, saturated aqueous $NaHCO_3$, extracted with EtOAc. The organic phase was washed with brine, dried over Na_2SO_4, and evaporated to dryness. The residue was purified by silica gel column chromatography (Petroleum ether/EtOAc = 3:1) to afford **S1**(*58*) (3.5 g, 98%) as a white foam: $[\alpha]_D^{25}$= 21.4 (*c* 0.32, CH_2Cl_2); ^1H NMR (400 MHz, Chloroform-d) δ 8.03 – 7.93 (m, 8H), 7.87 (d, *J* = 8.0 Hz, 2H), 7.61 (t, *J* = 7.2 Hz, 1H), 7.57 – 7.50 (m, 2H), 7.49 – 7.29 (m, 9H), 7.05 (d, *J* = 9.2 Hz, 2H), 6.02 (t, *J* = 9.2 Hz, 1H), 5.83 (t, *J* = 8.0 Hz, 1H), 5.73 (t, *J* = 9.6 Hz, 1H), 5.54 (d, *J* = 7.2 Hz, 1H), 4.70 (dd, *J* = 12.0, 2.8 Hz, 1H), 4.59 – 4.50 (m, 1H), 4.46 – 4.37 (m, 1H). ^{13}C NMR (150 MHz, $CDCl_3$) δ 166.0, 165.8, 165.4, 165.1, 161.2, 143.3, 133.9, 133.73, 133.70, 133.6, 130.1, 130.0, 129.9, 129.8, 129.5, 128.9, 128.67, 128.66, 128.6, 125.8, 116.9, 98.5, 77.2, 73.2, 72.6, 71.6, 69.5, 63.1.

4-aminophenyl 2,3,4,6-tetra-*O*-benzoyl-β-D-glucopyranoside (S2)

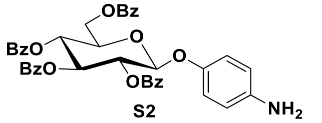

Compound **S2** was prepared according to **General procedure C**. A suspension of **S1** (3.0 g, 4.2 mmol) and 5% Pd/C (0.30 g) in EtOAc (16.0 mL) was degased and charged with H_2 at 1 atm for 3~6 h at room temperature. After the Pd/C was removed by filtration, the filtrate was evaporated under vacuum to afford **S2** (2.88 g, 99%) as a white foam: $[\alpha]_D^{25}$= 25.2 (*c* 0.57, CH_2Cl_2); ^1H NMR (600 MHz, Chloroform-*d*) δ 8.05 (dd, *J* = 8.3, 1.4 Hz, 2H), 7.99 (dd, *J* = 8.4, 1.4 Hz, 2H), 7.96 – 7.91 (m, 2H), 7.89 – 7.84 (m, 2H), 7.59 – 7.54 (m, 1H), 7.54 – 7.47 (m, 2H), 7.46 – 7.40 (m, 3H), 7.41 – 7.33 (m, 4H), 7.30 (t, *J* = 7.8 Hz, 2H), 6.85 (d, *J* = 8.8 Hz, 2H), 6.47 (d, *J* = 8.8 Hz, 2H), 5.98 (td, *J* = 9.5, 1.4 Hz, 1H), 5.78 (t, *J* = 8.8 Hz, 1H), 5.72 (t, *J* = 9.7 Hz, 1H), 5.25 (dd, *J* = 7.8, 1.2 Hz, 1H), 4.68 (dd, *J* = 12.0, 3.1 Hz, 1H), 4.55 (dd, *J* = 12.0, 6.4 Hz, 1H), 4.35 – 4.22 (m, 1H), 3.50 (s, 2H). ^{13}C NMR (150 MHz, $CDCl_3$) δ 166.2, 165.9, 165.4, 165.2, 150.0, 142.7, 133.6, 133.4, 133.3, 130.0, 129.9, 129.7, 129.3, 128.9, 128.8, 128.6, 128.5, 128.4, 119.3, 115.9, 101.3, 77.2, 73.0, 72.5, 71.9, 69.9, 63.4. HRMS (ESI) m/z calcd for $C_{40}H_{34}NO_{10}$ $[M+H]^+$ 688.2177, found 688.2172.

***Para*-(3-Phenylpropiolamido) phenyl 2,3,4,6-tetra-*O*-benzoyl-β-D-glucopyranoside (1a)**

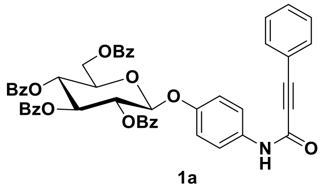

Compound **1a** was prepared according to **General procedure D**. The **S2** (2.8 g, 4.0 mmol), phenylpropiolic acid (0.76 g, 5.2 mmol), EDCI (1.15 g, 6.0 mmol), DMAP (49.0 mg, 0.4 mmol) were dissolved in CH_2Cl_2 (50 mL) and the mixture was stirred at room temperature 4~6 h. After concentrated in vacuo, the resulting residue was purified by silica gel column chromatography (Petroleum ether/EtOAc=3:1) to afford **1a** as a yellow foam (3.1 g, 95%): $[\alpha]_D^{25}$= 33.0 (*c* 0.27, $CHCl_3$); ^1H NMR (600 MHz, Chloroform-*d*) δ 8.04 (dd, *J* = 7.8, 1.2 Hz, 2H), 7.97 (dd, *J* = 7.8, 1.2 Hz, 2H), 7.94 (dd, *J* = 7.8, 1.2 Hz, 2H), 7.86 (dd, *J* = 7.8, 1.2 Hz, 2H), 7.69 (s, 1H), 7.61 – 7.56 (m, 1H), 7.55 – 7.49 (m, 4H), 7.48 – 7.34 (m, 12H), 7.30 (t, *J* = 7.8 Hz, 2H), 7.01 (d, *J* = 9.0 Hz, 1H), 6.00 (t, *J* = 9.6 Hz, 1H), 5.80 (dd, *J* = 9.6, 7.8 Hz, 1H), 5.71 (t, *J* = 9.6 Hz, 1H), 5.36 (d, *J* = 7.8 Hz, 1H), 4.69 (dd, *J* = 12.6, 3.0 Hz, 1H), 4.52 (dd, *J* = 12.0, 7.2 Hz, 1H), 4.36 – 4.27 (m, 1H). ^{13}C NMR (150 MHz, $CDCl_3$) δ 166.2, 165.9, 165.4, 165.2, 154.0, 151.0, 133.7, 133.54, 133.51, 133.48, 133.1,

132.7, 130.5, 130.0, 129.95, 129.93, 129.8, 129.6, 129.2, 128.8, 128.72, 128.65, 128.61, 128.59, 128.5, 121.5, 120.0, 118.1, 100.1, 85.9, 83.5, 77.2, 72.9, 72.8, 71.8, 69.7, 63.3. HRMS (ESI) m/z calcd for $C_{49}H_{41}N_2O_{11}$ $[M+NH_4]^+$ 833.2705, found 833.2709.

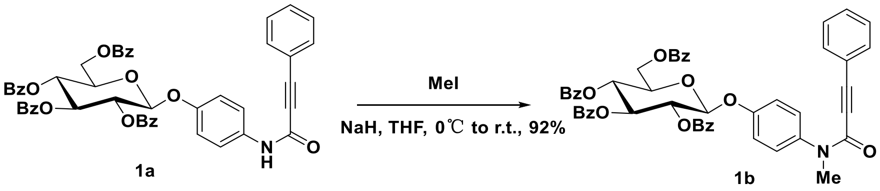

Fig. S3. Synthesis of the glycosyl PPAP donor **1b**.

To a solution of **1a** (0.5 g,0.61 mmol) in THF (5.0 mL) was added NaH (37 mg, 60% in mineral oil, 0.92 mmol) at 0 °C and stirred for 15 min then add CH_3I (0.1 mL, 1.5 mmol). The mixture was stirred for 1 h at room temperature and then poured into ice water, extracted with CH_2Cl_2, washed (brine), dried (Na_2SO_4) and evaporated the solvent. The residue was purified by column chromatography on silica gel (Petroleum ether/EtOAc=3:1) to afford **1b** as a yellow foam (0.46 g, 92%). $[\alpha]_D^{25}$=21.3 (*c* 0.36, $CHCl_3$); ^1H NMR (600 MHz, Chloroform-*d*) δ 8.01 – 7.92 (m, 6H), 7.87 (dd, *J* = 7.8, 1.2 Hz, 2H), 7.56 (t, *J* = 7.4 Hz, 1H), 7.52 (t, *J* = 7.2 Hz, 2H), 7.45 (t, *J* = 7.8 Hz, 1H), 7.42 – 7.34 (m, 7H), 7.29 (m, 3H), 7.18 (t, *J* = 7.8 Hz, 2H), 7.15 – 7.02 (m, 6H), 6.04 (t, *J* = 9.6 Hz, 1H), 5.85 (dd, *J* = 9.6, 7.8 Hz, 1H), 5.73 (t, *J* = 9.6 Hz, 1H), 5.45 (d, *J* = 7.8 Hz, 1H), 4.68 (dd, *J* = 12.0, 3.0 Hz, 1H), 4.56 (dd, *J* = 12.0, 6.6 Hz, 1H), 4.42 – 4.33 (m, 1H), 3.29 (s, 3H). ^{13}C NMR (150 MHz, $CDCl_3$) δ 166.0, 165.9, 165.4, 165.2, 156.2, 154.5, 138.6, 133.7, 133.55, 133.51, 133.45, 132.5, 130.1, 130.0, 129.9, 129.8, 129.5, 129.1, 128.8, 128.70, 128.67, 128.61, 128.56, 128.54, 128.48, 128.4, 120.3, 117.7, 99.6, 91.3, 82.6, 77.2, 72.9, 72.8, 71.8, 69.7, 63.3, 36.5. HRMS (ESI) calcd for $C_{50}H_{39}NO_{11}Na$ $[M+Na]^+$ 852.2415, found 852.2419.

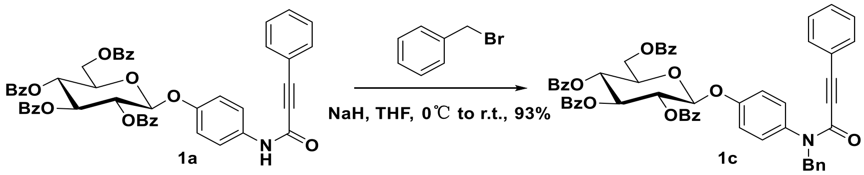

Fig. S4. Synthesis of the glycosyl PPAP donor **1c**.

To a solution of **1a** (0.3 g, 0.37 mmol) in THF (3.0 mL) was added NaH (29 mg, 60% in mineral oil, 0.92 mmol) at 0 °C and stirred for 15 min, then benzyl bromide (88 μL, 0.74 mmol) was added. The mixture was stirred for 3 h at room temperature and then poured into ice water, extracted with CH_2Cl_2, washed (brine), dried (Na_2SO_4) and evaporated the solvent. The residue was purified by column chromatography on silica gel (Petroleum ether/EtOAc = 3:1) to afford **1c** (310 mg, 93%) as a light yellow solid: $[\alpha]_D^{25}$= 20.5 (*c* 4.65, $CHCl_3$); ^1H NMR (400 MHz, Chloroform-*d*) δ 7.98 – 7.91 (m, 6H), 7.85 (d, *J* = 7.6 Hz, 2H), 7.56 – 7.48 (m, 3H), 7.47 – 7.26 (m, 12H), 7.26 – 7.14 (m, 5H), 7.08 – 6.89 (m, 6H), 6.02 (t, *J* = 9.6 Hz, 1H), 5.82 (dd, *J* = 9.6, 7.8 Hz, 1H), 5.71 (t, *J* = 9.6 Hz, 1H), 5.41 (d, *J* = 7.8 Hz, 1H), 5.00 – 4.81 (m, 2H), 4.65 (dd, *J* = 12.0, 3.0 Hz, 1H), 4.52 (dd, *J* = 12.0, 6.8 Hz, 1H), 4.38 – 4.28 (m, 1H). ^{13}C NMR (100 MHz, $CDCl_3$) δ 166.0, 165.9, 165.4, 165.1, 156.4, 154.6, 137.0, 136.7, 133.7, 133.53, 133.50, 133.4, 132.6, 132.5, 130.13, 130.00, 130.0, 129.97, 129.7, 129.4, 129.1, 128.8, 128.79, 128.71, 128.69, 128.63, 128.59, 128.55, 128.52, 128.4, 127.7, 120.3, 117.4, 99.4, 92.0, 82.6, 77.2, 72.84, 72.77, 71.8, 69.7, 63.3, 52.4. HRMS (ESI) calcd for $C_{56}H_{43}NO_{11}Na$ $[M+Na]^+$ 928.2728, found 928.2723.

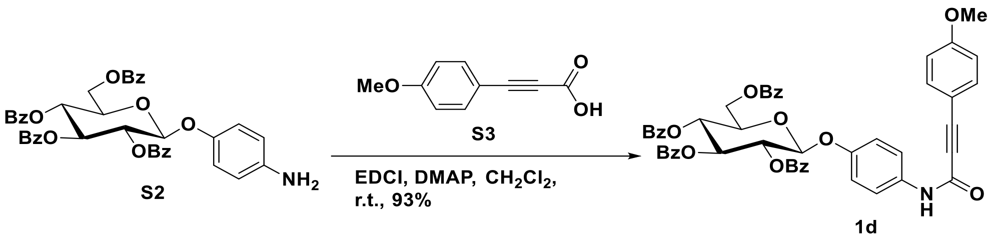

Fig. S5. Synthesis of the glycosyl PPAP donor **1d**.

Compound **1d** was prepared from **S2** (0.53 g, 0.77 mmol) and **S3**(*59*) (0.18 g, 1.0 mmol) using a similar procedure as that for the preparation of **1a** (**General procedure D)**. The crude product was purified by silica gel column chromatography (Petroleum ether/EtOAc = 4:1) to afford **1d** (0.60 g, 93%) as a light yellow solid: $[\alpha]_D^{25}$= 32.0 (*c* 1.06, $CHCl_3$); ^{1}H NMR (600 MHz, Chloroform-d) δ 8.04 (d, *J* = 7.7 Hz, 2H), 7.95 (dd, *J* = 18.1, 7.8 Hz, 4H), 7.86 (d, *J* = 7.7 Hz, 2H), 7.73 (s, 1H), 7.58 (t, *J* = 7.5 Hz, 1H), 7.53 – 7.34 (m, 13H), 7.29 (t, *J* = 7.7 Hz, 2H), 7.00 (d, *J* = 8.5 Hz, 2H), 6.85 (d, *J* = 8.3 Hz, 2H), 6.00 (t, *J* = 9.5 Hz, 1H), 5.80 (t, *J* = 8.7 Hz, 1H), 5.71 (t, *J* = 9.6 Hz, 1H), 5.36 (d, *J* = 7.8 Hz, 1H), 4.68 (dd, *J* = 12.1, 3.0 Hz, 1H), 4.52 (q, *J* = 12.1, 6.7 Hz, 1H), 4.34 – 4.28 (m, 1H), 3.81 (s, 3H). ^{13}C NMR (150 MHz, $CDCl_3$) δ 166.2, 165.9, 165.4, 165.3, 161.3, 153.9, 151.3, 134.5, 133.7, 133.52, 133.50, 133.47, 133.3, 130.0, 129.93, 129.91, 129.8, 129.6, 129.2, 128.8, 128.7, 128.64, 128.60, 128.57, 128.5, 121.4, 118.1, 114.4, 111.8, 100.1, 86.6, 82.8, 77.2, 72.9, 72.8, 71.8, 69.7, 63.3, 55.5. HRMS (ESI) calcd for $C_{50}H_{40}NO_{12}$ $[M+H]^+$ 846.2545, found 846.2544.

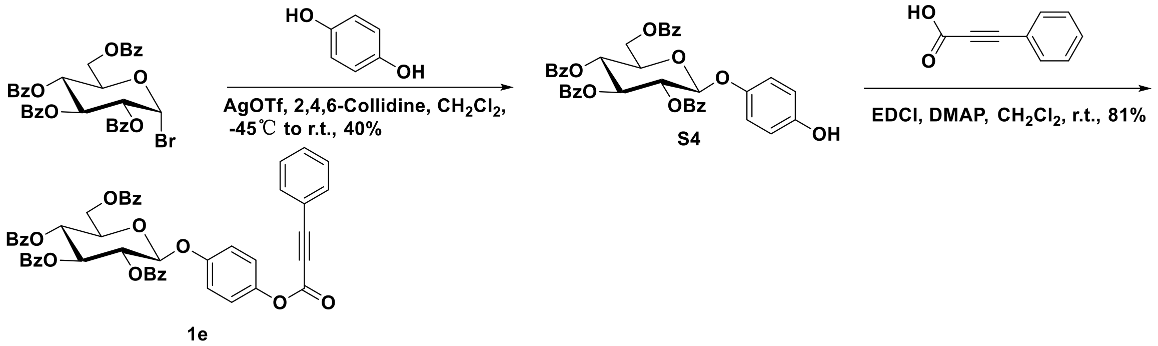

Fig. S6. Preparation of the glycosyl PPAP donor **1e**.

β-D-Glucopyranoside, 4-hydroxyphenyl, 2,3,4,6-tetrabenzoate (S4)(*60*)

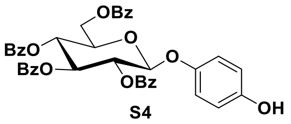

A mixture of hydroquinone (0.44 g, 4.0 mmol), AgOTf (0.52 g, 2.0 mmol), 2,4,6-collidine (0.12 mL, 0.9 mmol) in anhydrous CH_2Cl_2 (5.0 mL) was stirred at -45 °C. A solution of perbenzoylated glucosyl bromide (0.66 g, 1.0 mmol) in CH_2Cl_2 (5.0 mL) was added dropwise to this suspension. Stirring was continued for 0.5 h at -45 °C, and then the reaction mixture was allowed to warm to 0 °C and left stirring for 2.0 h. After completion of reaction (as detected by TLC), pyridine was added to the reaction mixture, and it was diluted with CH_2Cl_2 (20.0 mL) before being filtered over celite. The filtrate was washed successively with a 1 M aqueous $Na_2S_2O_3$ solution a 0.1 M aqueous HCl solution, and brine. Then the organic layer was dried with anhydrous Na_2SO_4. Filtration was followed by concentration to give the crude product which was further purified by silica gel chromatography (Petroleum ether/EtOAc = 3:1) to afford **S4** (275.0 mg, 40%) as a white foam: ^{1}H NMR (600 MHz, Chloroform-*d*) δ 8.02 (dd, *J* = 7.8, 1.2 Hz, 2H), 7.97 (dd, *J* = 7.8, 1.2 Hz, 2H),

7.92 (dd, J = 8.4, 1.2 Hz, 2H), 7.86 (dd, J = 7.8, 1.2 Hz, 2H), 7.59 – 7.52 (m, 1H), 7.55 – 7.47 (m, 2H), 7.47 – 7.39 (m, 3H), 7.37 (dt, J = 12.3, 7.8 Hz, 4H), 7.30 (t, J = 7.8 Hz, 2H), 6.89 (d, J = 9.0 Hz, 1H), 6.62 (d, J = 9.0 Hz, 2H), 5.97 (t, J = 9.6 Hz, 1H), 5.77 (dd, J = 9.6, 7.8 Hz, 1H), 5.71 (t, J = 9.6 Hz, 1H), 5.25 (d, J = 7.8 Hz, 1H), 5.07 (s, 1H), 4.67 (dd, J = 12.6, 3.0 Hz, 1H), 4.53 (dd, J = 12.0, 6.0 Hz, 1H), 4.30 – 4.22 (m, 1H). ^{13}C NMR (150 MHz, $CDCl_3$) δ 166.3, 166.0, 165.4, 165.3, 152.0, 151.1, 133.7, 133.51, 133.49, 133.4, 130.02, 129.97, 129.94, 129.91, 129.8, 129.7, 129.3, 128.9, 128.8, 128.61, 128.57, 128.5, 119.4, 116.1, 101.1, 77.2, 73.0, 72.6, 72.0, 69.9, 63.4.

Compound 1e

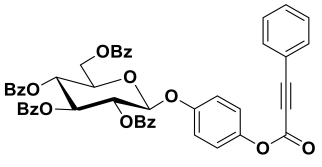

1e

Compound **1e** was prepared from **S4** (0.26 g, 0.38 mmol) using a similar procedure as that for the preparation of **1a** (**General procedure D)**. The crude product was purified by silica gel column chromatography (Petroleum ether/EtOAc = 4:1) to afford **1e** (0.25 g, 81%) as a light yellow solid: $[\alpha]_D^{25}$= 16.3 (c 1.14, $CHCl_3$); ^{1}H NMR (600 MHz, Chloroform-d) δ 8.04 (dd, J = 8.3, 1.4 Hz, 2H), 8.00 – 7.92 (m, 4H), 7.87 (dd, J = 8.4, 1.4 Hz, 2H), 7.64 – 7.61 (m, 2H), 7.60 – 7.57 (m, 1H), 7.54 – 7.47 (m, 3H), 7.47 – 7.43 (m, 3H), 7.42 – 7.35 (m, 6H), 7.31 (t, J = 8.3, 7.4 Hz, 2H), 7.06 (d, J = 9.0 Hz, 2H), 6.99 (d, J = 9.0 Hz, 2H), 6.01 (t, J = 9.5 Hz, 1H), 5.82 (dd, J = 9.7, 7.8 Hz, 1H), 5.73 (t, J = 9.7 Hz, 1H), 5.39 (d, J = 7.8 Hz, 1H), 4.70 (dd, J = 12.0, 3.0 Hz, 1H), 4.55 (dd, J = 12.0, 6.6 Hz, 1H), 4.38 – 4.31 (m, 1H). ^{13}C NMR (150 MHz, $CDCl_3$) δ 166.1, 165.9, 165.4, 165.2, 155.1, 152.5, 145.8, 133.7, 133.5, 133.55, 133.48, 133.3, 131.2, 130.0, 129.95, 129.93, 129.86, 129.6, 129.1, 128.84, 128.82, 128.76, 128.61, 128.58, 128.5, 122.5, 119.3, 118.4, 100.1, 89.0, 80.3, 77.2, 72.9, 72.8, 71.8, 69.7, 63.3. HRMS (ESI) calcd for $C_{49}H_{40}NO_{12}$ $[M+NH_4]^+$ 834.2545, found 834.2540.

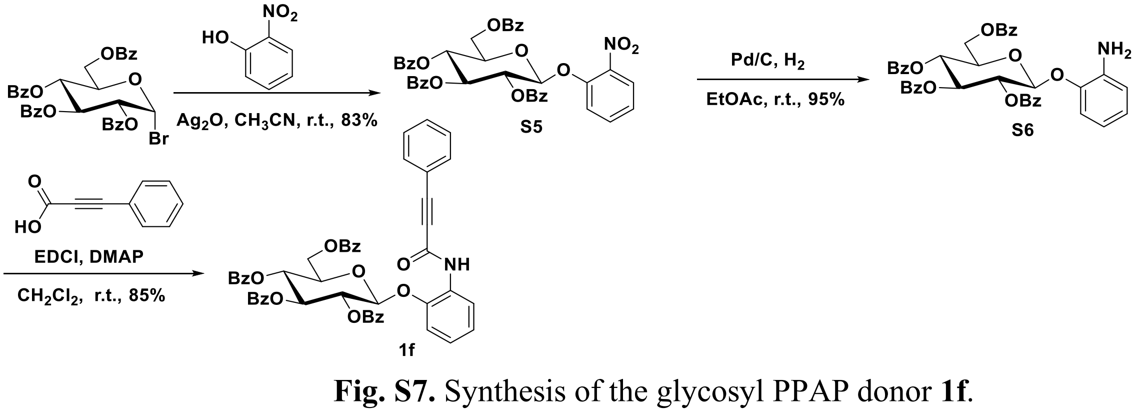

Fig. S7. Synthesis of the glycosyl PPAP donor **1f**.

2-nitrophenyl 2,3,4,6-tetra-*O*-benzoyl-β-D-glucopyranoside (S5)

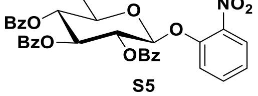

S5

Similar procedure as that used for the synthesis of **S1** (**General procedure A**) was applied to the preparation of **S5** (1.65 g, 83%) as a white foam: $[\alpha]_D^{25}$= 59.2 (c 0.78, $CHCl_3$); ^{1}H NMR (600 MHz, Chloroform-d) δ 8.04 – 7.99 (m, 4H), 7.95 (dd, J = 8.4, 1.2 Hz, 2H), 7.89 (dd, J = 8.4, 1.2 Hz, 2H), 7.69 (dd, J = 8.4, 1.8 Hz, 1H), 7.60 – 7.56 (m, 1H), 7.54 – 7.48 (m, 2H), 7.47 – 7.41 (m, 3H), 7.39

– 7.35 (m, 5H), 7.33 – 7.29 (m, 2H), 7.29 – 7.25 (m, 1H), 7.08 (td, *J* = 7.8, 1.2 Hz, 1H), 6.03 (t, *J* = 9.2 Hz, 1H), 5.87 (dd, *J* = 9.0, 7.2 Hz, 1H), 5.80 (t, *J* = 9.6 Hz, 1H), 5.54 (d, *J* = 7.2 Hz, 1H), 4.72 (dd, *J* = 12.0, 3.0 Hz, 1H), 4.56 (dd, *J* = 12.0, 6.0 Hz, 1H), 4.43 – 4.34 (m, 1H). ^{13}C NMR (150 MHz, $CDCl_3$) δ 166.0, 165.9, 165.3, 165.1, 149.2, 141.5, 133.74, 133.71, 133.5, 133.42, 133.36, 130.02, 129.97, 129.85, 129.6, 129.3, 128.75, 128.72, 128.61, 128.56, 128.5, 128.4, 125.3, 123.7, 119.6, 100.1, 77.2, 73.0, 72.7, 71.5, 69.5, 63.0. HRMS (ESI) calcd for $C_{40}H_{31}NO_{12}Na$ $[M+Na]^+$ 740.1738, found 740.1741.

2-aminophenyl 2,3,4,6-tetra-*O*-benzoyl-β-D-glucopyranoside (S6)

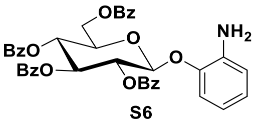

S6

Similar procedure as that used for the synthesis of **S2** (**General procedure C**) was applied to the preparation of **S6** (1.3 g, 95%) as a white foam:$[\alpha]_D^{20}$=22.9 (*c* 1.02, $CHCl_3$); ^{1}H NMR (600 MHz, Chloroform-d) δ 8.07 (dd, *J* = 8.4, 1.2 Hz, 2H), 7.99 (dd, *J* = 8.4, 1.2 Hz, 2H), 7.95 (dd, *J* = 7.8, 1.2 Hz, 2H), 7.88 (d, *J* = 7.2 Hz, 2H), 7.61 – 7.55 (m, 1H), 7.55 – 7.48 (m, 2H), 7.47 – 7.41 (m, 3H), 7.41 – 7.34 (m, 4H), 7.30 (t, *J* = 7.8 Hz, 2H), 7.03 (dd, *J* = 7.8, 1.2 Hz, 1H), 6.86 (td, *J* = 7.2, 1.8 Hz, 1H), 6.65 (dd, *J* = 7.8, 1.8 Hz, 1H), 6.52 (td, *J* = 7.8, 1.2 Hz, 1H), 6.05 (td, *J* = 9.6, 1.8 Hz, 1H), 5.75 (td, *J* = 9.6, 1.2 Hz, 1H), 5.33 (d, *J* = 7.8, 1H), 4.72 (dd, *J* = 12.0, 3.0 Hz, 1H), 4.55 (dd, *J* = 12.0, 6.6 Hz, 1H), 4.37 – 4.29 (m, 1H), 3.64 (s, 2H). ^{13}C NMR (150 MHz, CDC13) δ 166.2, 165.9, 165.6, 165.4, 144.5, 137.7, 133.7, 133.6, 133.5, 133.3, 129.98, 129.93, 129.88, 129.6, 129.1, 128.8, 128.7, 128.6, 128.54, 128.47, 124.4, 118.1, 116.8, 115.8, 100.9, 77.2, 72.75, 72.70, 72.1, 69.7, 63.2. HRMS (ESI) calcd for $C_{40}H_{33}NO_{10}Na$ $[M+Na]^+$ 710.1997, found 710.1999.

Compound 1f

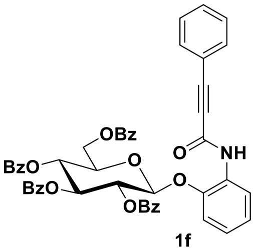

1f

Compound **1f** was prepared from **S6** (1.3 g, 1.89 mmol) according to the synthesis of **1a** (**General procedure D**) and purified by column chromatography (Petroleum ether/EtOAc = 4:1) to afford **1f** (1.3 g, 85%) as a light yellow solid: $[\alpha]_D^{25}$=-70.3 (*c* 0.37, $CHCl_3$); ^{1}H NMR (600 MHz, Chloroform-*d*) δ 8.43 (s, 1H), 8.39 (dd, *J* = 8.4 Hz, 1.8 Hz, 1H), 8.03 (d, *J* = 7.2 Hz, 2H), 7.96 (d, *J* = 7.8 Hz, 2H), 7.93 (d, *J* = 7.2 Hz, 2H), 7.88 (d, *J* = 7.2 Hz, 2H), 7.72 (d, *J* = 7.2 Hz, 2H), 7.56 (t, *J* = 7.4 Hz, 1H), 7.51 (t, *J* = 7.8 Hz, 1H), 7.47 – 7.39 (m, 5H), 7.36 (t, *J* = 7.2 Hz, 4H), 7.30 (t, *J* = 7.8 Hz, 2H), 7.24 (d, *J* = 7.2 Hz, 2H), 7.05 – 6.98 (m, 2H), 6.82 (td, *J* = 7.8, 1.2 Hz, 1H), 6.12 (t, *J* = 9.8 Hz, 1H), 5.84 (dd, *J* = 10.0, 7.8 Hz, 1H), 5.73 (t, *J* = 9.8 Hz, 1H), 5.37 (d, *J* = 7.8 Hz, 1H), 4.71 (dd, *J* = 12.2, 2.8 Hz, 1H), 4.55 (dd, *J* = 12.2, 6.6 Hz, 1H), 4.45 – 4.34 (m, 1H). ^{13}C NMR (150 MHz, $CDCl_3$) δ 166.4, 166.1, 165.8, 165.4, 151.2, 145.0, 133.8, 133.6, 133.5, 133.1, 130.3, 130.1, 130.0, 129.94, 129.88, 129.6, 128.8, 128.74, 128.71, 128.67, 128.61, 128.56, 128.4, 124.2, 123.7, 120.8, 120.5, 113.5, 100.0, 85.5, 83.9, 77.2, 72.9, 72.4, 72.2, 69.5, 63.2. HRMS (ESI) calcd for $C_{49}H_{37}NO_{11}Na$ $[M+Na]^+$ 838.2259, found 838.2261.

Compound 4a(*61*)

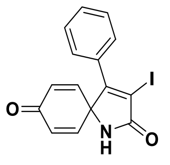

4a

white solid. ^{1}H NMR (600 MHz, Chloroform-d) δ 7.45 – 7.35 (m, 3H), 7.29 – 7.26 (m, 2H), 6.64 (d, *J* = 10.0 Hz, 2H), 6.34 (d, *J* = 10.0 Hz, 2H). ^{13}C NMR (150 MHz, CDCl3) δ 184.1, 170.0, 161.1, 144.3, 131.8, 130.4, 128.8, 127.8, 97.6, 77.2, 67.0.

Compound 4b(*62*)

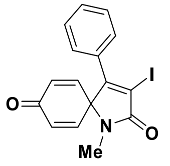

4b

white solid. ^{1}H NMR (600 MHz, Chloroform-*d*) δ 7.43 – 7.40 (m, 1H), 7.37 (t, *J* = 7.3 Hz, 2H), 7.29 (d, *J* = 7.1 Hz, 2H), 6.51 (d, *J* = 10.1 Hz, 2H), 6.46 (d, *J* = 10.2 Hz, 2H), 2.97 (s, 3H). ^{13}C NMR (150 MHz, $CDCl_3$) δ 167.6, 158.1, 144.2, 133.5, 132.0, 130.3, 128.9, 127.9, 98.3, 77.2, 70.5, 27.2.

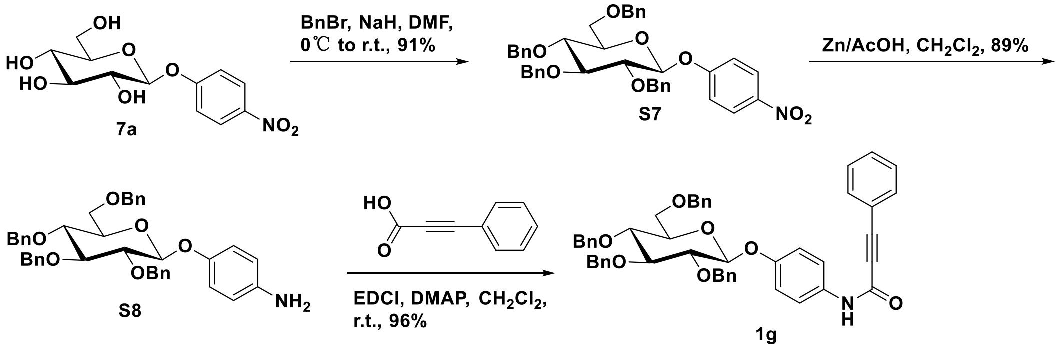

Fig. S8. Synthesis of the glycosyl PPAP donor **1g**.

***Para*-nitrophenyl 2,3,4,6-tetra-*O*-benzyl-β-D-glucopyranoside (S7)(*58*)**

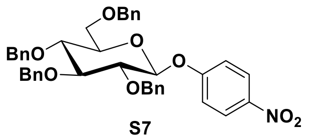

S7

To a solution of **7a** (0.6 g, 2.0 mmol) in THF (3.0 mL) was added NaH (0.48 g, 60% in oil, 12.0 mmol) at 0 °C and stirred for 15 min, then benzyl bromide (1.9 mL, 16.0 mmol) was added. The mixture was stirred for 3 h at room temperature and then poured into ice water, extracted with CH_2Cl_2, washed (brine), dried (Na_2SO_4) and evaporated the solvent. The residue was purified by column chromatography on silica gel (Petroleum ether/EtOAc = 5:1) to afford **S7** (1.2 g, 91%) as a white solid:^{1}H NMR (600 MHz, Chloroform-*d*) δ 8.18 (d, *J* = 9.0 Hz, 2H), 7.36 – 7.27 (m, 18H), 7.20 (dd, *J* = 7.8, 2.4 Hz, 2H), 7.09 (d, *J* = 9.0 Hz, 2H), 5.09 (d, *J* = 7.2 Hz, 1H), 4.96 (dd, *J* = 10.8, 1.8 Hz, 2H), 4.90 – 4.82 (m, 3H), 4.62 – 4.47 (m, 4H), 3.82 – 3.74 (m, 3H), 3.71 – 3.64 (m, 2H).

***Para*-aminophenyl 2,3,4,6-tetra-*O*-benzyl-β-D-glucopyranoside (S8)**(*63*)

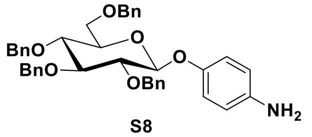

S8

To a solution of **S7** (1.0 g, 1.5 mmol) in CH_2Cl_2 (10.0 mL), zinc powder (1.47 g, 22.5 mmol) and acetic acid (4.28 mL, 75.0 mmol) were added sequentially at 0 °C. The reaction mixture was warmed to room temperature gradually, and the stirring was continued for 2 h. After the zinc powder was removed by filtration, the filtrate was washed successively with water, brine, and then dried over Na_2SO_4. Filtration was followed by concentration. The residue was purified by column chromatography on silica gel (Petroleum ether/EtOAc = 5:1) to afford **S8** (0.84 g, 89%) as a white solid: ^{1}H NMR (600 MHz, Chloroform-*d*) δ 7.38 – 7.33 (m, 2H), 7.35 – 7.25 (m, 16H), 7.21 – 7.16 (m, 2H), 6.94 (d, *J* = 9.0 Hz, 2H), 6.61 (d, *J* = 9.0 Hz, 1H), 5.06 (d, *J* = 10.8 Hz, 1H), 4.94 (d, *J* = 10.8 Hz, 1H), 4.88 – 4.78 (m, 4H), 4.64 – 4.51 (m, 3H), 3.78 (dd, *J* = 10.9, 2.0 Hz, 1H), 3.73 – 3.63 (m, 4H), 3.55 (ddd, *J* = 9.5, 5.2, 2.0 Hz, 1H).

***Para*-(3-phenylpropiolamido) phenyl 2,3,4,6-tetra-*O*-benzyl-β-D-glucopyranoside(1g)**

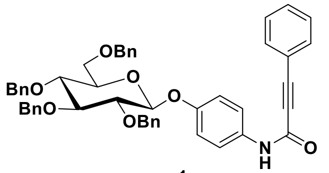

1g

Compound **1g** was prepared from **S8** (0.5 g, 0.79 mmol) according to the synthesis of **1a** (**General procedure D**) and purified by column chromatography (Petroleum ether/EtOAc = 4:1) to afford **1g** (0.58 g, 96%) as a light yellow solid: $[\alpha]_D^{20}$= -3.7 (*c* 0.99, $CHCl_3$); ^{1}H NMR (600 MHz, Chloroform-d) δ 7.60 – 7.57 (m, 2H), 7.54 (s, 1H), 7.50 – 7.42 (m, 3H), 7.39 (t, *J* = 7.6 Hz, 3H), 7.35 – 7.27 (m, 19H), 7.21 – 7.17 (m, 2H), 7.09 – 7.04 (m, 2H), 5.03 (d, *J* = 11.0 Hz, 1H), 4.98 – 4.93 (m, 2H), 4.84 (t, *J* = 11.6 Hz, 3H), 4.61 – 4.51 (m, 3H), 3.81 – 3.66 (m, 5H), 3.63 – 3.58 (m, 1H). ^{13}C NMR (150 MHz, CDCl3) δ 154.6, 151.0, 138.6, 138.3, 138.2, 138.1, 132.7, 132.3, 130.4, 128.7, 128.57, 128.55, 128.5, 128.44, 128.36, 128.1, 128.02, 127.96, 127.9, 127.83, 127.80, 121.6, 120.1, 117.7, 102.1, 85.8, 84.8, 83.6, 82.1, 77.8, 77.2, 75.9, 75.2, 75.2, 73.6, 68.9. HRMS (ESI) calcd for $C_{49}H_{46}NO_7$ $[M+H]^+$ 760.3269, found 760.3273.

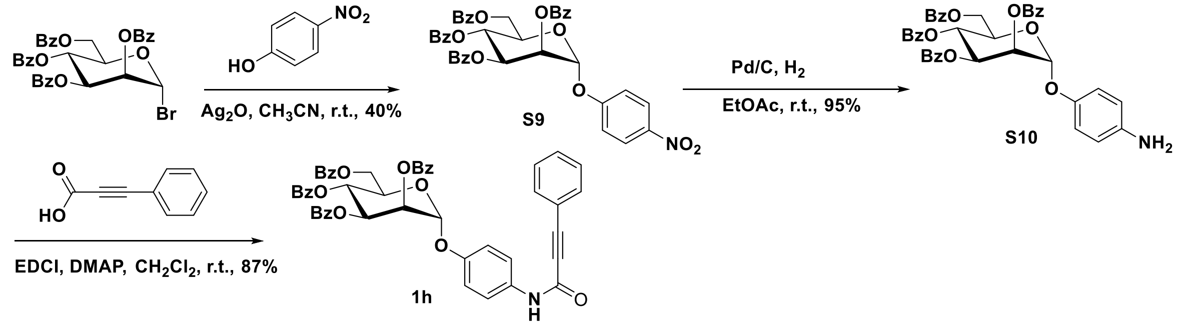

Fig. S9. Synthesis of the glycosyl PPAP donor **1h**.

***Para*-nitrophenyl 2,3,4,6-tetra-*O*-benzoyl-α-D-mannopyranoside (S9)**(*58*)

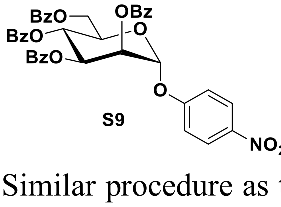

Similar procedure as that used for the synthesis of **S1** (**General procedure A**) was applied to the preparation of **S9** (1.4 g, 40%) as a white foam: $[\alpha]_D^{25}$= 17.4 (*c* 0.52, CH_2Cl_2); ^{1}H NMR (600 MHz, Chloroform-*d*) δ 8.17 – 8.12 (m, 2H), 8.13 – 8.08 (m, 2H), 8.01 – 7.93 (m, 4H), 7.91 – 7.86 (m, 2H), 7.67 – 7.61 (m, 1H), 7.61 – 7.56 (m, 1H), 7.55 – 7.50 (m, 1H), 7.50 – 7.43 (m, 3H), 7.42 –

7.35 (m, 4H), 7.33 – 7.27 (m, 4H), 6.19 – 6.07 (m, 2H), 5.93 (s, 2H), 4.69 – 4.57 (m, 1H), 4.56 – 4.44 (m, 2H).

***Para*-aminophenyl 2,3,4,6-tetra-*O*-benzoyl-α-D-mannopyranoside (S10)**

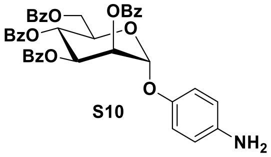

Similar procedure as that used for the synthesis of **S2** (**General procedure C**) was applied to the preparation of **S10** (1.1 g, 95%) as a white foam: $[\alpha]_D^{25}$= -12.7 (*c* 1.40, CH_2Cl_2); ^{1}H NMR (600 MHz, Chloroform-d) δ 8.10 (dd, *J* = 7.8, 1.2 Hz, 2H), 8.06 (dd, *J* = 7.8, 1.2 Hz, 2H), 7.98 (dd, *J* = 7.8, 1.2 Hz, 2H), 7.88 (dd, *J* = 8.4, 1.2 Hz, 2H), 7.64 – 7.56 (m, 2H), 7.53 – 7.49 (m, 1H), 7.47 – 7.41 (m, 5H), 7.38 (t, *J* = 8.4 Hz, 2H), 7.29 (t, *J* = 7.8 Hz, 2H), 7.04 (d, *J* = 9.0, 2H), 6.62 (d, *J* = 8.4, 2H), 6.21 – 6.12 (m, 2H), 5.91 (dd, *J* = 3.0, 1.8 Hz, 1H), 5.67 (d, *J* = 1.8 Hz, 1H), 4.67 (dd, *J* = 12.0, 2.4 Hz, 1H), 4.65 – 4.62 (m, 1H), 4.51 (dd, *J* = 12.0, 5.0 Hz, 1H), 3.52 (s, 2H). ^{13}C NMR (150 MHz, $CDCl_3$) δ 166.2, 165.7, 165.6, 165.5, 148.9, 142.1, 133.7, 133.6, 133.4, 133.1, 130.0, 129.93, 129.91, 129.87, 129.3, 129.1, 129.0, 128.7, 128.6, 128.5, 128.4, 118.2, 116.3, 97.0, 77.2, 70.5, 70.1, 69.4, 67.0, 63.0. HRMS (ESI) calcd for $C_{50}H_{39}NO_{11}Na$ $[M+Na]^+$ 710.1997, found 710.1998.

***Para*-(3-Phenylpropiolamido) phenyl 2,3,4,6-tetra-*O*-benzoyl-α-D-mannopyranoside (1h)**

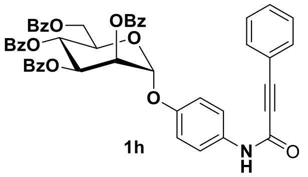

Compound **1h** was prepared from **S10** (1.1 g, 1.6 mmol) according to the synthesis of **1a** (**General procedure D**) and purified by column chromatography (Petroleum ether/EtOAc = 3: 1) to afford **1h** (1.1 g, 87%) as a light yellow solid: $[\alpha]_D^{25}$= 11.1 (*c* 2.23, CH_2Cl_2); ^{1}H NMR (600 MHz, Chloroform-*d*) δ 8.10 (dd, *J* = 7.8 Hz, 1.2 Hz, 2H), 8.04 (dd, *J* = 8.4 Hz, 1.2 Hz, 2H), 7.98 (dd, *J* = 8.4 Hz, 1.2 Hz, 2H), 7.90 (s, 1H), 7.88 (dd, *J* = 8.4 Hz, 1.2 Hz, 2H), 7.65 – 7.49 (m, 7H), 7.47 – 7.32 (m, 10H), 7.28 (t, *J* = 7.8 Hz, 2H), 7.19 (d, *J* = 9.0 Hz, 2H), 6.21 – 6.12 (m, 2H), 5.92 (dd, *J* = 3.0, 2.4 Hz, 1H), 5.76 (d, *J* = 1.8 Hz, 1H), 4.65 (dd, *J* = 12.6, 2.4 Hz, 1H), 4.59 – 4.54 (m, 1H), 4.50 (dd, *J* = 12.0, 5.4 Hz, 1H). ^{13}C NMR (150 MHz, $CDCl_3$) δ 166.2, 165.7, 165.6, 165.5, 152.7, 151.0, 133.8, 133.7, 133.4, 133.3, 132.9, 132.7, 130.4, 130.0, 129.95, 129.89, 129.85, 129.80, 129.2, 129.1, 128.9, 128.8, 128.7, 128.67, 128.61, 128.5, 121.6, 120.1, 117.3, 96.2, 85.9, 83.6, 77.2, 70.4, 70.0, 69.7, 66.9, 62.9. HRMS (ESI) calcd for $C_{49}H_{37}NO_{11}Na$ $[M+Na]^+$ 838.2259, found 838.2258.

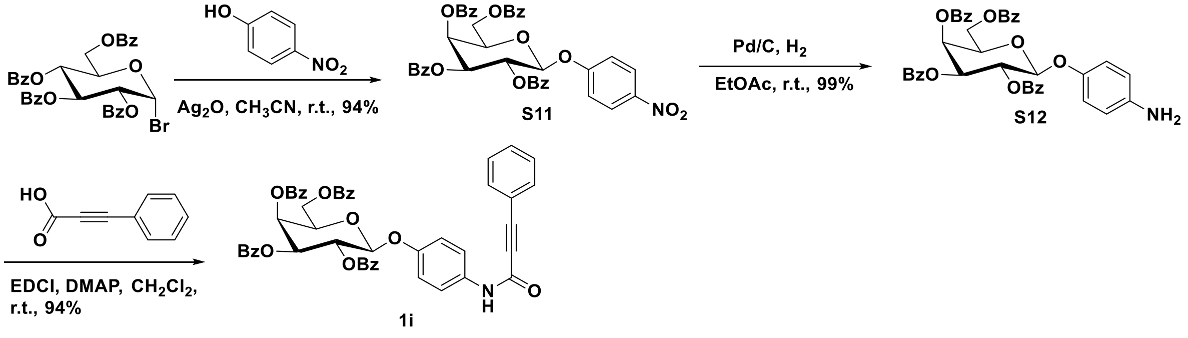

Fig. S10. Synthesis of the glycosyl PPAP donor **1i**.

***Para*-nitrophenyl 2,3,4,6-tetra-*O*-benzoyl-β-D-Galactopyranoside (S11)**(*58*)

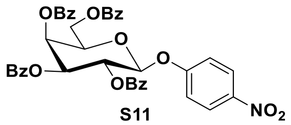

S11

Similar procedure as that used for the synthesis of **S1** (**General procedure A**) was applied to the preparation of **S11** (2.4 g, 94%) as a white foam: $[\alpha]_D^{25}$= 97.9 (*c* 0.44, CH_2Cl_2); ^{1}H NMR (600 MHz, Chloroform-*d*) δ 8.12 (dd, *J* = 8.4, 1.8 Hz, 2H), 8.05 – 7.99 (m, 4H), 7.95 (dd, *J* = 8.4, 1.8 Hz, 2H), 7.82 (dd, *J* = 7.8, 1.2 Hz, 2H), 7.67 – 7.61 (m, 2H), 7.53 – 7.43 (m, 6H), 7.39 – 7.34 (m, 2H), 7.30 – 7.24 (m, 2H), 7.10 (d, *J* = 9.0, 2H), 6.12 (dd, *J* = 10.2, 7.8 Hz, 1H), 6.08 (dd, *J* = 3.6, 1.2 Hz, 1H), 5.74 (dd, *J* = 10.2, 3.6 Hz, 1H), 5.52 (d, *J* = 7.8 Hz, 1H), 4.71 – 4.64 (m, 1H), 4.61 – 4.55 (m, 2H). ^{13}C NMR (150 MHz, $CDCl_3$) δ 166.1, 165.6, 165.3, 161.3, 143.3, 134.0, 133.8, 133.7, 133.6, 130.2, 129.93, 129.88, 129.8, 129.4, 129.0, 128.9, 128.8, 128.7, 128.6, 128.5, 125.8, 117.0, 99.0, 77.2, 72.4, 71.6, 69.3, 67.9, 62.4.

4-aminophenyl 2,3,4,6-tetra-*O*-benzoyl-β-D-Galactopyranoside (S12)

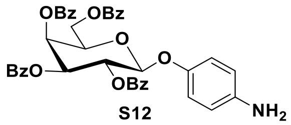

S12

Similar procedure as that used for the synthesis of **S2** (**General procedure C**) was applied to the preparation of **S12** (2.19 g, 99%) as a white foam: $[\alpha]_D^{25}$= 107.4 (*c* 0.6, CH_2Cl_2); ^{1}H NMR (600 MHz, Chloroform-*d*) δ 8.12 (dd, *J* = 7.8, 1.2 Hz, 2H), 8.05 (dd, *J* = 8.4, 1.2 Hz, 2H), 7.97 (dd, *J* = 8.4, 1.2 Hz, 2H), 7.82 (dd, *J* = 8.4, 1.8 Hz, 2H), 7.61 (t, *J* = 7.2 Hz, 1H), 7.57 (t, *J* = 7.2 Hz,1H), 7.51 – 7.42 (m, 6H), 7.36 (t, *J* = 7.8 Hz, 2H), 7.26 (t, *J* = 7.8 Hz, 2H), 6.88 (d, *J* = 9.0 Hz, 2H), 6.49 (d, *J* = 9.0 Hz, 2H), 6.08 – 6.01 (m, 2H), 5.67 (dd, *J* = 10.4, 3.6 Hz, 1H), 5.22 (d, *J* = 7.8 Hz, 1H), 4.69 (dd, *J* = 11.4, 7.4 Hz, 1H), 4.53 (dd, *J* = 11.4, 5.4 Hz, 1H), 4.44 (dd, *J* = 7.4, 6.0 Hz, 1H), 3.49 (s, 2H). ^{13}C NMR (150 MHz, $CDCl_3$) δ 166.1, 165.7, 165.4, 150.2, 142.7, 133.8, 133.43, 133.40, 133.38, 130.2, 129.9, 129.8, 129.5, 129.4, 129.0, 128.8, 128.7, 128.6, 128.5, 128.4, 119.2, 115.9, 101.7, 77.2, 71.9, 71.7, 69.8, 68.2, 62.4. HRMS (ESI) calcd for $C_{40}H_{34}NO_{10}$ $[M+H]^+$ 688.2177, found 688.2171.

***Para*-(3-Phenylpropiolamido) phenyl 2,3,4,6-tetra-*O*-benzoyl-β-D-Galactopyranoside (1i)**

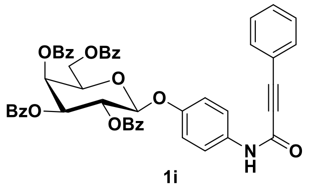

1i

Compound **1i** was prepared from **S12** (1.6 g, 2.3 mmol) according to the synthesis of **1a** (**General procedure D**) and purified by column chromatography (Petroleum ether/EtOAc = 3: 1) to afford **1i** (1.75 g, 94%) as a light yellow solid: $[\alpha]_D^{25}$= 106.4 (*c* 2.68, CH_2Cl_2); ^{1}H NMR (600 MHz, Chloroform-*d*) δ 8.12 (dd, *J* = 7.8, 1.2 Hz, 2H), 8.06 (dd, *J* = 7.8, 1.2 Hz, 2H), 7.97 (dd, *J* = 8.4, 1.8 Hz, 2H), 7.82 (dd, *J* = 7.8, 1.2 Hz, 2H), 7.66 – 7.55 (m, 2H), 7.56 – 7.45 (m, 7H), 7.47 – 7.39 (m, 4H), 7.40 – 7.31 (m, 4H), 7.27 (t, *J* = 7.8 Hz, 2H), 7.04 (d, *J* = 9.0 Hz, 2H), 6.11 – 6.03 (m, 2H), 5.70 (dd, *J* = 10.3, 3.2 Hz, 1H), 5.35 (d, *J* = 7.8 Hz, 1H), 4.65 (dd, *J* = 11.5, 7.7 Hz, 1H), 4.55 (dd, *J* = 11.5, 5.3 Hz, 1H), 4.51 – 4.45 (m, 1H). ^{13}C NMR (150 MHz, $CDCl_3$) δ 166.2, 165.7, 165.4, 154.1, 151.03, 151.00, 133.9, 133.6, 133.54, 133.52, 133.10, 133.06, 132.7, 130.4, 130.2, 129.92, 129.86, 129.4, 129.2, 128.9, 128.8, 128.7, 128.64, 128.59, 128.5, 121.5, 120.0, 118.1, 100.5, 85.9,

83.5, 77.2, 72.0, 71.8, 69.6, 68.1, 62.5. HRMS (ESI) calcd for $C_{49}H_{38}NO_{11}$ $[M+H]^+$ 816.2439, found 816.2435.

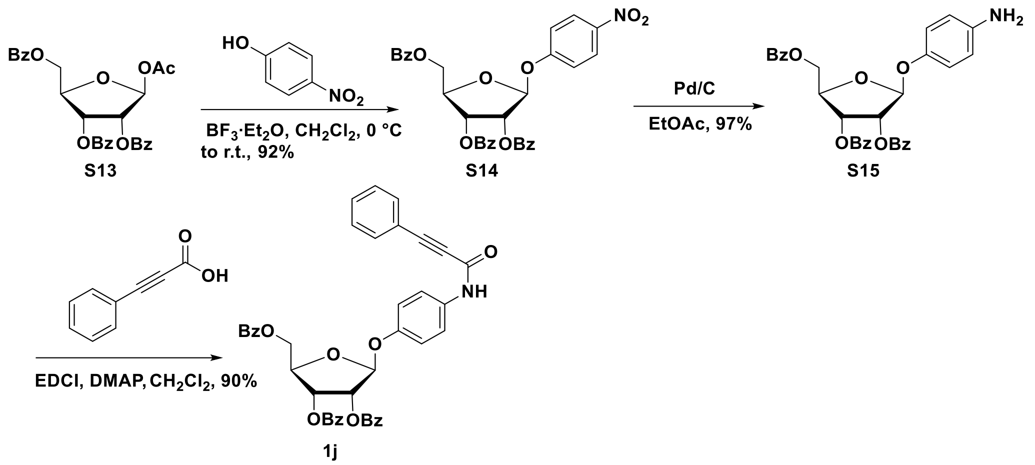

Fig. S11. Synthesis of the glycosyl PPAP donor **1j**.

***Para*-nitrophenyl 2,3,5-tri-*O*-benzoyl-β-D-ribofuranoside (S14)**

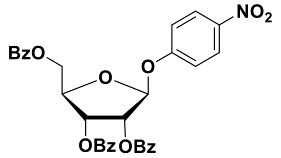

S14

Compound **S14** was prepared from **S13** (2.0 g, 4.0 mmol) and 4-nitrophenol (0.83 g, 6.0 mmol) according to **General procedure B** and purified by column chromatography (Petroleum ether/EtOAc = 4:1) to afford **S14** (2.14 g, 92%) as a white foam: $[\alpha]^{25}_{D}$= -59.5 (*c* 0.27, CH_2Cl_2); ^1H NMR (600 MHz, Chloroform-*d*) δ 8.10 – 8.07 (m, 2H), 8.04 (dd, *J* = 8.4, 1.2 Hz, 2H), 7.94 (dd, *J* = 8.4, 1.2 Hz, 2H), 7.89 (dd, *J* = 7.8, 1.2 Hz, 2H), 7.60 (tt, *J* = 7.6, 1.8 Hz, 1H), 7.57 – 7.50 (m, 2H), 7.47 – 7.43 (m, 2H), 7.39 – 7.31 (m, 4H), 7.02 (d, *J* = 9.0 Hz, 2H), 6.05 (dd, *J* = 6.6, 4.8 Hz, 1H), 5.96 (t, *J* = 2.3 Hz, 2H), 4.91 – 4.84 (m, 2H), 4.44 (dd, *J* = 12.0, 3.6 Hz, 1H). ^{13}C NMR (150 MHz, $CDCl_3$) δ 166.0, 165.6, 165.3, 160.8, 142.8, 134.0, 133.8, 133.4, 130.0, 129.9, 129.8, 129.5, 128.9, 128.8, 128.6, 128.4, 125.8, 116.4, 103.3, 80.5, 77.2, 75.7, 71.5, 63.0. HRMS (ESI) calcd for $C_{32}H_{29}N_2O_{10}$ $[M+NH_4]^+$ 601.1817, found 601.1820.

***Para*-aminophenyl 2,3,5-tri-*O*-benzoyl-β-D-ribofuranoside (S15)**

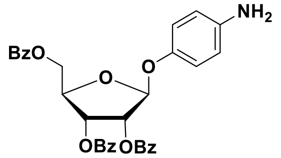

S15

Similar procedure as that used for the synthesis of **S2** (**General procedure C**) was applied to the preparation of **S15** (1.82 g, 97%) as a white foam: $[\alpha]^{20}_{D}$= -5.1 (*c* 1.77, $CHCl_3$); ^1H NMR (600 MHz, Chloroform-d) δ 8.02 (dd, *J* = 19.2, 7.8 Hz, 4H), 7.91 (d, *J* = 7.8 Hz, 2H), 7.58 (t, *J* = 7.2 Hz, 1H), 7.54 – 7.47 (m, 2H), 7.43 (t, *J* = 7.8 Hz, 2H), 7.33 (t, *J* = 7.8 Hz, 4H), 6.89 (d, *J* = 9.0 Hz, 2H), 6.59 (d, *J* = 9.0 Hz, 2H), 6.01 (dd, *J* = 7.2, 4.8 Hz, 1H), 5.90 (d, *J* = 4.8 Hz, 1H), 5.77 (s, 1H), 4.85 – 4.78 (m, 1H), 4.72 (dd, *J* = 12.0, 4.8 Hz, 1H), 4.58 (dd, *J* = 12.0, 4.8 Hz, 1H), δ 3.37 (s, 2H). ^{13}C NMR (150 MHz, $CDCl_3$) δ 166.3, 165.5, 165.3, 149.4, 141.9, 133.7, 133.5, 133.1, 129.94, 129.89,

1296, 129.7, 129.2, 129.0, 128.6, 128.5, 128.4, 118.3, 116.2, 104.9, 79.5, 77.2, 75.9, 72.4, 64.5. HRMS (ESI) calcd for $C_{32}H_{28}NO_8$ $[M+H]^+$ 554.1809, found 554.1812.

***Para*-(3-Phenylpropiolamido) phenyl 2,3,5-tri-*O*-benzoyl-β-D-ribofuranoside (1j)**

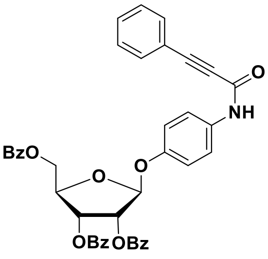

1j

Compound **1j** was prepared from **S15** (1.2 g, 2.17 mmol) according to the synthesis of **1a** (**General procedure D**) and purified by column chromatography (Petroleum ether/EtOAc = 5: 1) to afford **1j** (1.35 g, 90%) as a light yellow solid: $[\alpha]_D^{20}$= -15.4 (*c* 0.28, $CHCl_3$); ^{1}H NMR (600 MHz, Chloroform-*d*) δ 8.03 (dd, *J* = 8.4, 1.2 Hz, 2H), 7.96 (dd, *J* = 8.4, 1.8 Hz, 2H), 7.92 (dd, *J* = 8.4, 1.2 Hz, 2H), 7.62 (s, 1H), 7.60 – 7.48 (m, 6H), 7.48 – 7.41 (m, 5H), 7.39 – 7.31 (m, 7H), 7.01 (d, *J* = 9.0 Hz, 2H), 6.02 (dd, *J* = 7.2, 4.8 Hz, 1H), 5.92 (d, *J* = 4.8 Hz, 1H), 5.86 (s, 1H), 4.87 – 4.80 (m, 1H), 4.74 (dd, *J* = 12.0, 4.2 Hz, 1H), 4.52 (dd, *J* = 12.0, 4.8 Hz, 1H). ^{13}C NMR (150 MHz, $CDCl_3$) δ 166.3, 165.5, 165.4, 153.4, 151.0, 133.8, 133.6, 133.3, 132.7, 132.2, 130.4, 130.0, 129.96, 129.90, 129.8, 129.6, 129.1, 128.9, 128.72, 128.69, 128.6, 128.4, 121.5, 120.1, 117.1, 103.9, 85.8, 83.5, 79.8, 77.2, 75.9, 72.1, 64.1. HRMS (ESI) calcd for $C_{41}H_{31}NO_9Na$ $[M+Na]^+$ 704.1891, found 704.1890.

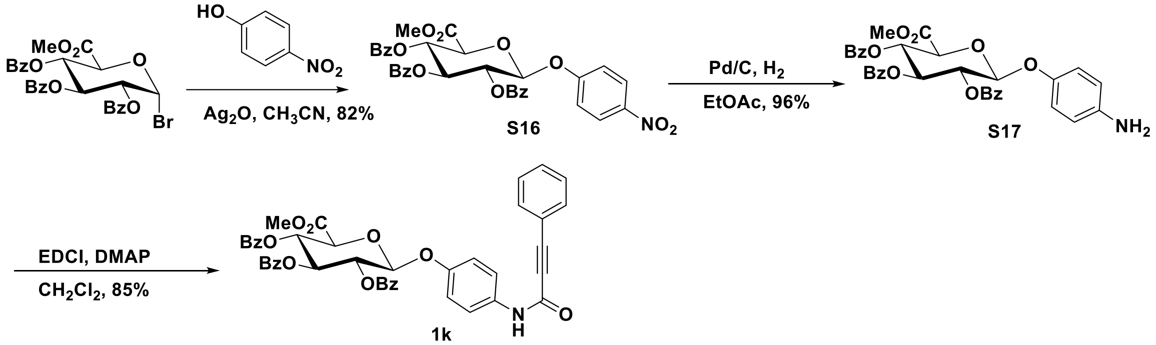

Fig. S12. Synthesis of the glycosyl PPAP donors **1k**.

Methyl-*para*-nitrophenyl 2,3,4-tri-*O*-benzoyl-β-D-glucuronate (S16)(*64*)

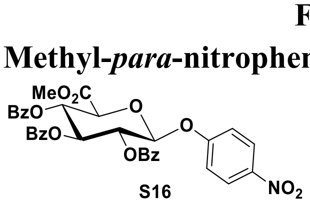

Similar procedure as that used for the synthesis of **S1** (**General procedure A**) was applied to the preparation of **S16** (1.52 g, 82%) as a white foam: $[\alpha]_D^{25}$= 5.3 (*c* 0.77, CH_2Cl_2); ^{1}H NMR (600 MHz, Chloroform-d) δ 8.20 (d, *J* = 9.0 Hz, 2H), 8.00 – 7.91 (m, 6H), 7.57 – 7.48 (m, 3H), 7.41 – 7.34 (m, 6H), 7.13 (d, *J* = 9.0 Hz, 2H), 6.01 (t, *J* = 8.4 Hz, 1H), 5.89 (t, *J* = 8.4 Hz, 1H), 5.79 (dd, *J* = 8.4, 6.0 Hz, 1H), 5.67 (d, *J* = 6.0 Hz, 1H), 4.65 (d, *J* = 7.8 Hz, 1H), 3.61 (s, 3H). ^{13}C NMR (150 MHz, CDCl3) δ 167.0, 165.4, 165.2, 165.0, 161.2, 143.3, 133.72, 133.71, 133.66, 129.9, 128.63, 128.56, 128.50, 128.47, 125.8, 116.8, 98.0, 73.0, 70.6, 70.5, 69.0, 53.0. HRMS (ESI) calcd for $C_{34}H_{31}N_2O_{12}$ $[M+NH_4]^+$ 659.1872, found 659.1876.

Methyl-*para*-aminophenyl 2,3,4-tri-*O*-benzoyl-β-D-glucuronate (S17)

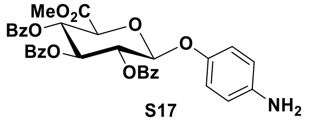

S17

Similar procedure as that used for the synthesis of **S2** (**General procedure C**) was applied to the preparation of **S17** (1.19 g, 96%) as a white foam: $[\alpha]_D^{25}$= 11.2 (*c* 0.18, CH_2Cl_2); ^{1}H NMR (600 MHz, Chloroform-*d*) δ 7.97 (d, *J* = 7.2 Hz, 2H), 7.94 (d, *J* = 7.2 Hz, 2H), 7.89 (d, *J* = 9.0 Hz, 2H), 7.53 (t, *J* = 7.8 Hz, 2H), 7.46 (t, *J* = 7.2 Hz, 1H), 7.38 (t, *J* = 7.2 Hz, 4H), 7.32 (t, *J* = 7.2 Hz, 2H), 6.86 (d, *J* = 9.0 Hz, 2H), 6.57 (d, *J* = 9.0 Hz, 2H), 5.95 (t, *J* = 9.6 Hz, 1H), 5.82 – 5.71 (m, 2H), 5.26 (d, *J* = 7.8 Hz, 1H), 4.42 (d, *J* = 9.0 Hz, 1H), 3.68 (s, 3H). ^{13}C NMR (150 MHz, $CDCl_3$) δ 167.3, 165.8, 165.3, 165.1, 150.0, 142.8, 133.6, 133.53, 133.51, 130.01, 130.98, 129.2, 128.8, 128.61, 128.57, 128.5, 119.5, 116.0, 101.2, 77.2, 73.1, 72.0, 71.5, 70.1, 53.1. HRMS (ESI) calcd for $C_{34}H_{29}NO_{10}Na$ $[M+Na]^+$ 634.1684, found 634.1682.

Compound 1k

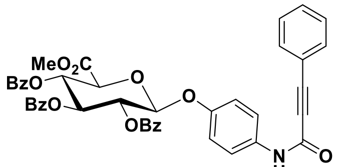

1k

Compound **1k** was prepared from **S17** (1.1 g, 1.8 mmol) according to the synthesis of **1a** (**General procedure D**) and purified by column chromatography (Petroleum ether/EtOAc = 3:1) to afford **1k** (1.13 g, 85%) as a light yellow solid: $[\alpha]_D^{25}$= 88.5 (*c* 1.12, CH_2Cl_2); ^{1}H NMR (600 MHz, Chloroform-*d*) δ 7.99 – 7.93 (m, 4H), 7.90 (dd, *J* = 8.4, 1.2 Hz, 2H), 7.68 (s, 1H), 7.57 – 7.50 (m, 4H), 7.49 – 7.45 (m, 3H), 7.45 – 7.41 (m, 1H), 7.40 – 7.31 (m, 8H), 7.01 (d, *J* = 9.0 Hz, 2H), 5.98 (t, *J* = 9.0 Hz, 1H), 5.82 (t, *J* = 9.0 Hz, 1H), 5.78 (dd, *J* = 9.0, 7.2 Hz, 1H), 5.42 (d, *J* = 7.2 Hz, 1H), 4.50 (d, *J* = 9.0 Hz, 1H), 3.66 (s, 3H). ^{13}C NMR (150 MHz, $CDCl_3$) δ 167.2, 165.7, 165.3, 165.1, 153.9, 151.0, 133.7, 133.63, 133.59, 133.1, 132.7, 130.5, 130.47, 130.02, 129.0, 128.83, 128.77, 128.7, 128.6, 128.5, 121.6, 120.0, 118.2, 99.9, 85.9, 83.4, 77.2, 73.1, 71.7, 71.3, 69.8, 53.1. HRMS (ESI) calcd for $C_{43}H_{37}N_2O_{11}$ $[M+NH_4]^+$ 757.2392, found 757.2395.

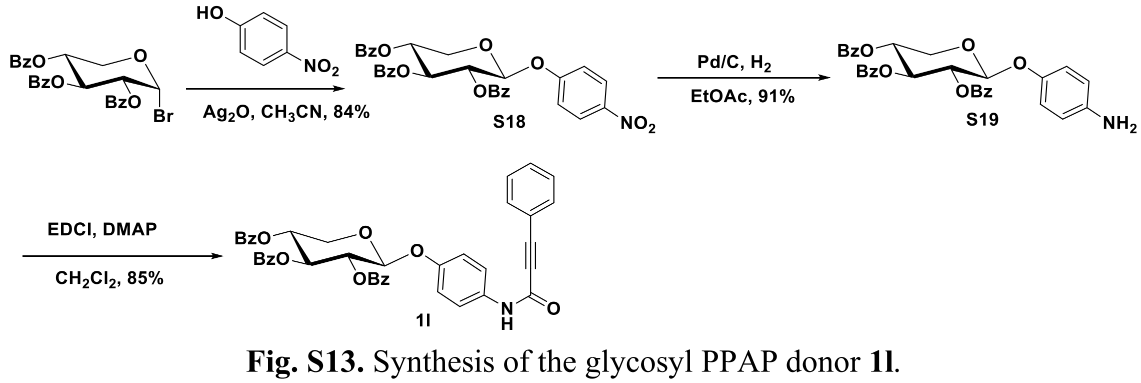

Fig. S13. Synthesis of the glycosyl PPAP donor **1l**.

***Para*-nitrophenyl 2,3,4-tri-*O*-benzoyl-β-D-xylopyranoside (S18)**

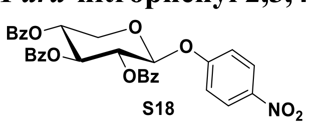

S18

Similar procedure as that used for the synthesis of **S1** (**General procedure A**) was applied to the preparation of **S18** (1.76 g, 84%) as a white foam: $[\alpha]_D^{25}$ = -31.8 (*c* 0.74, CH_2Cl_2); ^{1}H NMR (400 MHz, Chloroform-*d*) δ 8.21 (d, *J* = 9.2 Hz, 2H), 8.13 – 8.04 (m, 4H), 7.99 (dd, *J* = 8.4, 1.2 Hz, 2H),

7.65 – 7.50 (m, 3H), 7.47 (t, *J* = 8.0 Hz, 2H), 7.40 (t, *J* = 8.0 Hz, 2H), 7.32 (t, *J* = 8.0 Hz, 2H), 7.15 (d, *J* = 9.2 Hz, 2H), 5.81 (t, *J* = 5.2 Hz, 1H), 5.74 (d, *J* = 3.2 Hz, 1H), 5.58 (dd, *J* = 4.8, 3.2 Hz, 1H), 5.36 (q, *J* = 4.4 Hz, 1H), 4.53 (dd, *J* = 12.8, 3.2 Hz, 1H), 4.00 (dd, *J* = 12.8, 4.0 Hz, 1H). ^{13}C NMR (100 MHz, $CDCl_3$) δ 165.6, 165.3, 165.0, 161.1, 143.0, 133.85, 133.77, 133.7, 130.1, 130.0, 129.3, 129.1, 128.9, 128.7, 128.62, 128.57, 126.0, 116.6, 96.7, 77.2, 68.5, 68.2, 67.8, 60.8. HRMS (ESI) calcd for $C_{32}H_{29}N_2O_{10}$ $[M+NH_4]^+$ 601.1817, found 601.1821.

***Para*-aminophenyl 2,3,4-tri-*O*-benzoyl-β-D-xylopyranoside (S19)**

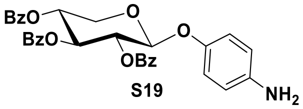

Similar procedure as that used for the synthesis of **S2** (**General procedure C**) was applied to the preparation of **S19** (1.0 g, 91%) as a white foam: $[\alpha]_D^{25}$=-10.1 (*c* 0.37, CH_2Cl_2); ^{1}H NMR (600 MHz, Chloroform-*d*) δ 8.10 – 7.96 (m, 6H), 7.59 – 7.50 (m, 3H), 7.45 – 7.31 (m, 6H), 6.89 (d, *J* = 9.0 Hz, 2H), 6.62 (d, *J* = 9.0 Hz, 2H), 5.81 (t, *J* = 6.6 Hz, 1H), 5.57 (dd, *J* = 6.6, 4.8 Hz, 1H), 5.37 (d, *J* = 5.0 Hz, 1H), 5.36 – 5.33 (m, 1H), 4.54 (dd, *J* = 12.4, 4.2 Hz, 1H), 3.82 (dd, *J* = 12.4, 6.0 Hz, 1H), 3.48 (s, 2H). ^{13}C NMR (150 MHz, $CDCl_3$) δ 165.7, 165.4, 165.3, 149.7, 142.2, 133.6, 133.5, 130.0, 129.34, 129.30, 129.26, 128.6, 128.5, 118.7, 116.2, 99.3, 77.2, 69.9, 69.7, 68.8, 61.1. HRMS (ESI) calcd for $C_{32}H_{31}N_2O_8$ $[M+NH_4]^+$ 571.2075, found 571.2071.

***Para*-(3-Phenylpropiolamido) phenyl 2,3,4-tri-*O*-benzoyl-β-D-xylopyranoside (1l)**

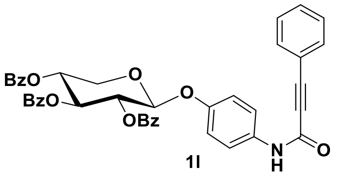

Compound **1l** was prepared from **S19** (0.94 g, 1.7 mmol) according to the synthesis of **1a** (**General procedure D**) and purified by column chromatography (Petroleum ether/EtOAc = 3:1) to afford **1l** (0.98 g, 85%) as a light yellow solid: $[\alpha]_D^{25}$ = -3.3 (*c* 1.77, CH_2Cl_2); ^{1}H NMR (600 MHz, Chloroform-*d*) δ 8.10 – 8.03 (m, 4H), 8.00 (dd, *J* = 7.8, 1.2 Hz, 2H), 7.95 – 7.88 (m, 1H), 7.59 – 7.49 (m, 7H), 7.45 – 7.36 (m, 5H), 7.36 – 7.30 (m, 4H), 7.03 (d, *J* = 9.0 Hz, 2H), 5.80 (t, *J* = 6.0 Hz, 1H), 5.57 (dd, *J* = 6.6, 4.2 Hz, 1H), 5.52 (d, *J* = 4.2 Hz, 1H), 5.38 – 5.32 (m, 1H), 4.52 (dd, *J* = 12.6, 3.6 Hz, 1H), 3.87 (dd, *J* = 12.6, 5.4 Hz, 1H). ^{13}C NMR (150 MHz, $CDCl_3$) δ 165.7, 165.4, 165.3, 149.7, 142.2, 133.6, 133.5, 130.0, 129.3, 129.3, 129.3, 128.6, 128.5, 118.7, 116.2, 99.3, 77.2, 69.9, 69.7, 68.8, 61.1. ^{13}C NMR (150 MHz, $CDCl_3$) δ 165.7, 165.31, 165.26, 153.6, 151.1, 133.7, 133.6, 132.7, 132.6, 130.4, 130.1, 130.0, 129.3, 129.2, 129.1, 128.7, 128.66, 128.62, 128.5, 121.8, 120.0, 117.5, 97.8, 85.9, 83.5, 77.2, 69.3, 69.1, 68.4, 60.8. HRMS (ESI) calcd for: $C_{41}H_{32}NO_9$ $[M+H]^+$ 682.2072, found 682.2075.

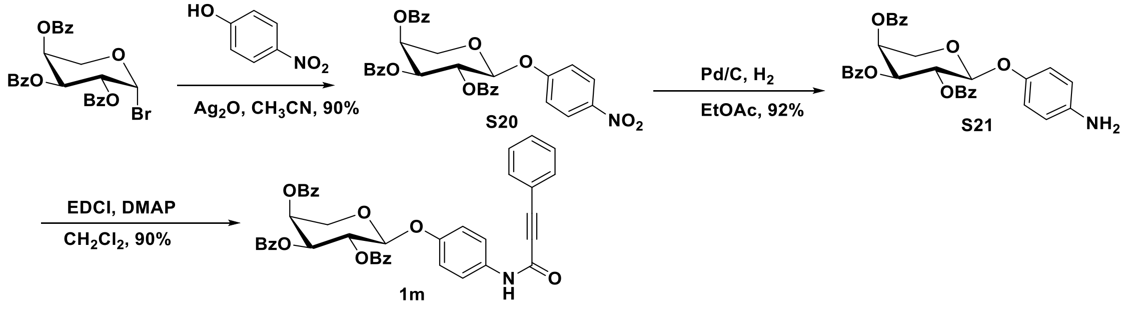

Fig. S14. Synthesis of the glycosyl PPAP donor **1m**.

***Para*-nitrophenyl 2,3,4-tri-*O*-benzoyl-β-L-arabinopyranoside (S20)**

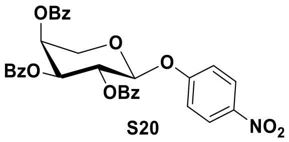

S20

Similar procedure as that used for the synthesis of **S1** (**General procedure A**) was applied to the preparation of **S20** (1.57 g, 90%) as a white foam： $[\alpha]_D^{25}$= 54.8 (*c* 0.30, CH_2Cl_2); ^{1}H NMR (600 MHz, Chloroform-*d*) δ 8.20 (d, *J* = 9.6 Hz, 2H), 8.08 – 8.00 (m, 6H), 7.61 – 7.55 (m, 3H), 7.48 – 7.40 (m, 6H), 7.13 (d, *J* = 9.0 Hz, 2H), 5.92 (dd, *J* = 6.0, 4.2 Hz, 1H), 5.84 – 5.78 (m, 2H), 5.61 (d, *J* = 4.8 Hz, 1H), 4.42 (dd, *J* = 12.2, 5.4 Hz, 1H), 4.10 (dd, *J* = 12.2, 3.0 Hz, 1H). ^{13}C NMR (150 MHz, $CDCl_3$) δ 165.6, 165.5, 165.2, 161.3, 143.1, 133.9, 133.8, 133.7, 130.02, 130.97, 129.2, 129.1, 128.8, 128.73, 128.67, 126.0, 116.7, 97.4, 77.2, 69.4, 69.3, 67.1, 61.5. HRMS (ESI) calcd for $C_{32}H_{29}N_2O_{10}$ $[M+NH_4]^+$ 601.1817, found 601.1816.

***Para*-aminophenyl 2,3,4-tri-*O*-benzoyl-β-L-arabinopyranoside (S21)**

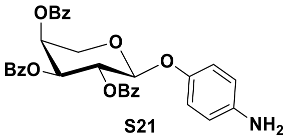

S21

Similar procedure as that used for the synthesis of **S2** (**General procedure C**) was applied to the preparation of **S21** (1.27 g, 92%) as a white foam: $[\alpha]_D^{25}$= 132.2 (*c* 0.73, CH_2Cl_2); ^{1}H NMR (400 MHz, Chloroform-*d*) δ 8.06 (td, *J* = 8.0, 1.2 Hz, 4H), 8.03 – 7.96 (m, 2H), 7.62 – 7.47 (m, 3H), 7.43 (q, *J* = 8.0 Hz, 4H), 7.36 (t, *J* = 7.6 Hz, 2H), 6.89 (d, *J* = 8.8 Hz, 2H), 6.60 (d, *J* = 8.8 Hz, 2H), 5.93 (dd, *J* = 8.0, 5.6 Hz, 1H), 5.76 (dt, *J* = 4.6, 2.1 Hz, 1H), 5.70 (dd, *J* = 8.3, 3.5 Hz, 1H), 5.26 (d, *J* = 6.0 Hz, 1H), 4.42 (dd, *J* = 12.8, 4.6 Hz, 1H), 3.98 (dd, *J* = 12.8, 2.4 Hz, 1H), 3.39 (s, 2H). ^{13}C NMR (100 MHz, $CDCl_3$) δ 165.8, 165.7, 165.3, 149.9, 142.4, 133.54, 133.52, 133.48, 130.00, 130.98, 129.93, 129.4, 129.3, 129.2, 128.60, 128.58, 128.5, 119.0, 116.1, 100.4, 77.2, 70.4, 70.0, 68.1, 62.4. HRMS (ESI) calcd for: $C_{32}H_{28}NO_8$ $[M+H]^+$ 554.1809, found 554.1806.

***Para*-(3-Phenylpropiolamido) phenyl 2,3,4-tri-*O*-benzoyl-β-L-arabinopyranoside (1m)**

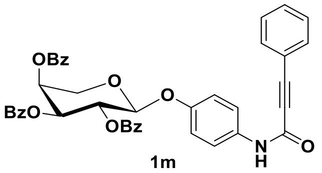

1m

Compound **1m** was prepared from **S21** (0.84 g, 1.52 mmol) according to the synthesis of **1a** (**General procedure D**) and purified by column chromatography (Petroleum ether/EtOAc = 4: 1) to afford **1m** (0.93 g, 90%) as a light yellow solid: $[\alpha]_D^{25}$= 85.1 (*c* 2.52, CH_2Cl_2); ^{1}H NMR (600 MHz, Chloroform-*d*) δ 8.06 – 8.03 (m, 4H), 8.01 (dd, *J* = 7.8, 1.2 Hz, 2H), 7.70 (s, 1H), 7.61 – 7.52 (m, 5H), 7.49 (d, *J* = 9.0 Hz, 2H), 7.46 – 7.41 (m, 5H), 7.40 – 7.34 (m, 4H), 7.04 (d, *J* = 9.0 Hz, 2H), 5.91 (dd, *J* = 7.8, 5.4 Hz, 1H), 5.80 – 5.76 (m, 1H), 5.73 (dd, *J* = 7.8, 3.6 Hz, 1H), 5.39 (d, *J* = 5.4 Hz, 1H), 4.41 (dd, *J* = 12.6, 5.4 Hz, 1H), 4.01 (dd, *J* = 12.6, 2.8 Hz, 1H). ^{13}C NMR (150 MHz, $CDCl_3$) δ 165.7, 165.6, 165.2, 153.8, 151.0, 133.6, 133.5, 132.62, 132.59, 130.4, 129.92, 129.90, 129.3, 129.1, 129.0, 128.62, 128.59, 128.56, 128.5, 121.6, 119.9, 117.7, 98.8, 85.8, 83.4, 69.9, 69.7, 67.7, 61.8. HRMS (ESI) calcd for: $C_{41}H_{32}NO_9$ $[M+H]^+$ 682.2072, found 682.2075.

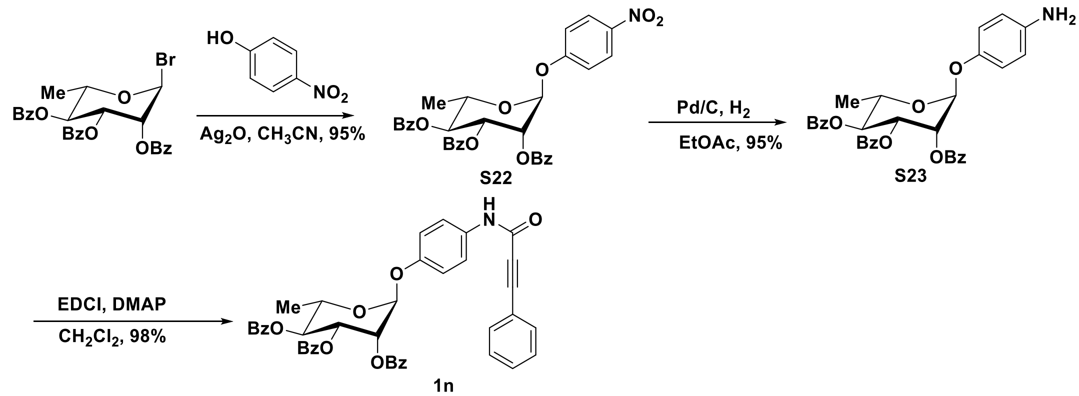

Fig. S15. Synthesis of the glycosyl PPAP donor **1n**.

***Para*-nitrophenyl 2,3,4-tri-*O*-benzoyl-α-L-rhamnopyranoside (S22)**

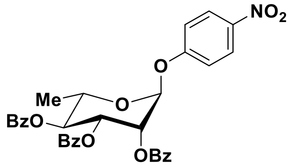

S22

Similar procedure as that used for the synthesis of **S1** (**General procedure A**) was applied to the preparation of **S22** (3.0 g, 95%) as a white foam: $[\alpha]_D^{20}$= 12.9 (*c* 0.85, $CHCl_3$); ^1H NMR (600 MHz, Chloroform-*d*) δ 8.26 (d, *J* = 9.6 Hz, 2H), 8.13 (d, *J* = 7.2 Hz, 2H), 7.97 (d, *J* = 7.8 Hz, 2H), 7.86 (d, *J* = 7.8 Hz, 2H), 7.64 (t, *J* = 7.2 Hz, 1H), 7.52 (t, *J* = 7.8 Hz, 3H), 7.44 (t, *J* = 7.4 Hz, 1H), 7.38 (t, *J* = 7.8 Hz, 2H), 7.33 – 7.26 (m, 4H), 6.02 (dd, *J* = 9.6, 3.0 Hz, 1H), 5.89 – 5.85 (m, 2H), 5.79 (t, *J* = 10.0 Hz, 1H), 4.28 – 4.20 (m, 1H), 1.36 (d, *J* = 6.2 Hz, 3H). ^{13}C NMR (150 MHz, $CDCl_3$) δ 165.7, 165.6, 165.6, 160.6, 143.0, 133.9, 133.5, 133.4, 130.0, 129.8, 129.05, 128.99, 128.9, 128.8, 128.5, 128.4, 126.0, 116.5, 95.8, 71.2, 70.2, 69.5, 68.2, 17.7. HRMS (ESI) calcd for: $C_{33}H_{31}N_2O_{10}$ $[M+NH_4]^+$ 615.1973, found 615.1975.

***Para*-aminophenyl 2,3,4-tri-*O*-benzoyl-α-L-rhamnopyranoside (S23)**

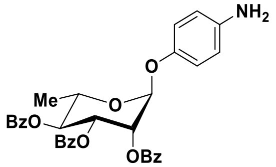

S23

Similar procedure as that used for the synthesis of **S2** (**General procedure C**) was applied to the preparation of **S23** (2.7 g, 95%) as a white foam:$[\alpha]_D^{20}$= 62.3 (*c* 0.47, $CHCl_3$); ^1H NMR (600 MHz, Chloroform-*d*) δ 8.13 (dd, *J* = 8.4, 1.2 Hz, 2H), 7.98 (dd, *J* = 8.4, 1.8 Hz, 2H), 7.85 (dd, *J* = 7.8, 1.2 Hz, 2H), 7.66 – 7.59 (m, 1H), 7.55 – 7.47 (m, 4H), 7.46 – 7.40 (m, 1H), 7.42 – 7.36 (m, 2H), 7.30 – 7.24 (m, 2H), 7.00 (d, *J* = 9.0 Hz, 2H), 6.67 (d, *J* = 9.0 Hz, 2H), 6.03 (dd, *J* = 10.2, 3.6 Hz, 1H), 5.84 (dd, *J* = 3.6, 1.8 Hz, 1H), 5.75 (t, *J* = 10.0 Hz, 1H), 5.58 (d, *J* = 1.8 Hz, 1H), 4.41 – 4.33 (m, 1H), 3.51 (s, 2H), 1.36 (d, *J* = 6.2 Hz, 3H). ^{13}C NMR (150 MHz, $CDCl_3$) δ 165.9, 165.7, 149.2, 142.0, 133.7, 133.5, 133.3, 130.1, 129.89, 129.87, 129.5, 129.4, 129.3, 128.8, 128.6, 128.4, 118.1, 116.3, 96.9, 77.2, 71.9, 70.9, 70.0, 67.4, 17.8. HRMS (ESI) calcd for $C_{33}H_{33}N_2O_8$ $[M+NH_4]^+$ 585.2231, found 585.2233.

***Para*-(3-Phenylpropiolamido) phenyl 2,3,4-tri-*O*-benzoyl-α-L-rhamnopyranoside (1n)**

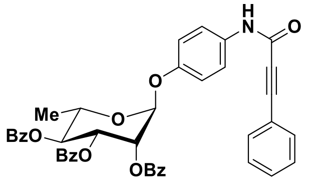

1n

Compound **1n** was prepared from **S23** (2.5 g, 4.4 mmol) according to the synthesis of **1a** (**General procedure D**) and purified by column chromatography (Petroleum ether/EtOAc = 3:1) to afford **1n** (3.0 g, 98%) as a light yellow solid: $[\alpha]_D^{25}$= 2.5 (*c* 1.51, CH_2Cl_2); ^{1}H NMR (600 MHz, Chloroform-*d*) δ 8.13 (d, *J* = 7.2 Hz, 2H), 7.98 (dd, *J* = 7.8, 1.2 Hz, 2H), 7.86 (dd, *J* = 7.8, 1.2 Hz, 2H), 7.63 (t, *J* = 7.2 Hz, 1H), 7.60 – 7.57 (m, 3H), 7.56 – 7.48 (m, 5H), 7.47 – 7.42 (m, 2H), 7.41 – 7.37 (m, 4H), 7.28 (t, *J* = 7.8 Hz, 2H), 7.18 (d, *J* = 9.0 Hz, 2H), 6.03 (dd, *J* = 10.2, 3.6 Hz, 1H), 5.85 (dd, *J* = 3.6, 1.8 Hz, 1H), 5.76 (t, *J* = 10.0 Hz, 1H), 5.71 (d, *J* = 1.8 Hz, 1H), 4.41 – 4.23 (m, 1H), 1.35 (d, *J* = 6.2 Hz, 3H). ^{13}C NMR (150 MHz, $CDCl_3$) δ 165.8, 165.64, 165.61, 153.0, 151.0, 133.7, 133.4, 133.2, 132.6, 132.4, 130.3, 130.0, 129.78, 129.77, 129.23, 129.20, 129.1, 128.7, 128.6, 128.5, 128.3, 121.7, 120.0, 117.1, 96.0, 85.8, 83.4, 71.6, 70.6, 69.8, 67.6, 17.7. HRMS (ESI) calcd for $C_{42}H_{37}N_2O_9$ $[M+NH_4]^+$ 713.2494, found 713.2495.

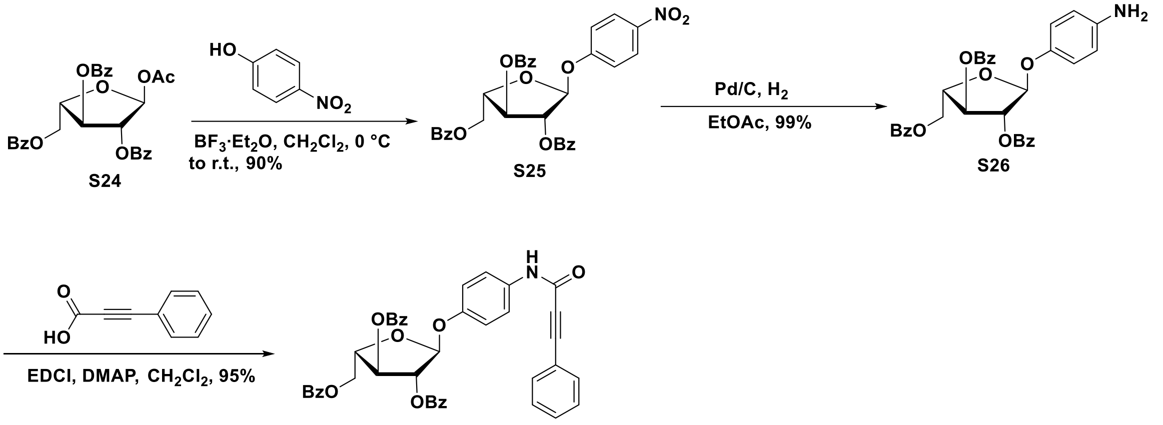

Fig. S16. Synthesis of the glycosyl PPAP donor **1o**.

***Para*-nitrophenyl 2,3,5-tri-*O*-benzoyl-L-arabinofuranoside (S25)**(*65*)

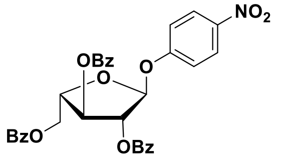

S25

Compound **S25** was prepared from **S24**(*66*) (2.0 g, 4.0 mmol) according to the synthesis of **S14** (**General procedure B**) to afford **S25** (2.1 g, 90%) as a white foam: $[\alpha]_D^{25}$= -60.7 (*c* 2.78, $CHCl_3$); ^{1}H NMR (400 MHz, Chloroform-*d*) δ 8.23 (d, *J* = 9.2 Hz, 2H), 8.11 (d, *J* = 7.6 Hz, 2H), 8.05 (t, *J* = 7.2 Hz, 4H), 7.67 – 7.58 (m, 2H), 7.57 – 7.47 (m, 3H), 7.42 (t, *J* = 7.6 Hz, 2H), 7.34 (t, *J* = 7.6 Hz, 2H), 7.22 (d, *J* = 9.2 Hz, 2H), 6.06 (s, 1H), 5.83 (d, *J* = 1.2 Hz, 1H), 5.72 (d, *J* = 4.0 Hz, 1H), 4.90 – 4.82 (m, 1H), 4.79 – 4.70 (m, 2H). ^{13}C NMR (150 MHz, $CDCl_3$) δ 166.3, 165.8, 165.5, 161.0, 142.9, 134.02, 133.96, 133.4, 130.10, 130.07, 129.9, 129.7, 129.0, 128.81, 128.78, 128.7, 128.6, 126.0, 116.7, 104.1, 83.0, 82.1, 77.6, 77.2, 63.5.

***Para*-aminophenyl 2,3,5-tri-*O*-benzoyl-L-arabinofuranoside (S26)**

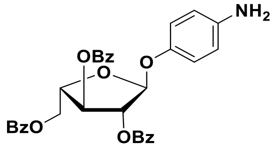

S26

Similar procedure as that used for the synthesis of **S2** (**General procedure C**) was applied to the preparation of **S26** (1.91 g, 99%) as a white foam: $[\alpha]_D^{20}$= -16.9 (*c* 0.42, $CHCl_3$); ^{1}H NMR (400 MHz, Chloroform-*d*) δ 8.16 – 8.08 (m, 2H), 8.08 – 7.99 (m, 4H), 7.60 (q, *J* = 7.8 Hz, 2H), 7.53 – 7.43 (m, 3H), 7.41 (t, *J* = 7.7 Hz, 2H), 7.29 (t, *J* = 7.7 Hz, 2H), 6.97 (d, *J* = 8.8 Hz, 2H), 6.67 (d, *J* = 8.8 Hz, 2H), 5.82 (s, 1H), 5.77 (d, *J* = 1.2 Hz, 1H), 5.68 (d, *J* = 4.5 Hz, 1H), 4.82 (dd, *J* = 11.0, 2.9 Hz, 1H), 4.78 – 4.69 (m, 2H). ^{13}C NMR (100 MHz, $CDCl_3$) δ 166.4, 165.9, 165.6, 149.3, 141.5, 133.7, 133.2, 130.13, 130.07, 129.92, 129.86, 129.3, 129.1, 128.70, 128.68, 128.5, 118.8, 116.5, 105.4, 82.4, 81.9, 77.9, 77.2, 63.8. HRMS (ESI) calcd for $C_{32}H_{27}NO_8Na$ $[M+Na]^+$ 576.1629, found 576.1629.

***Para*-(3-Phenylpropiolamido) phenyl 2,3,5-tri-*O*-benzoyl-L-arabinofuranoside (1o)**

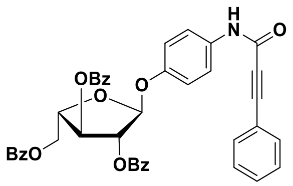

1o

Compound **1o** was prepared from **S26** (1.8 g, 3.2 mmol) according to the synthesis of **1a** (**General procedure D**) and purified by column chromatography (Petroleum ether/EtOAc = 3:1) to afford **1o** (2.1 g, 95%) as a light yellow solid: $[\alpha]_D^{20}$= -36.9 (*c* 2.01, $CHCl_3$); ^{1}H NMR (400 MHz, Chloroform-*d*) δ 8.12 (d, *J* = 7.6 Hz, 2H), 8.04 (t, *J* = 7.6 Hz, 4H), 7.71 (s, 1H), 7.66 – 7.46 (m, 9H), 7.45 – 7.26 (m, 7H), 7.12 (d, *J* = 8.8 Hz, 2H), 5.93 (s, 1H), 5.79 (s, 1H), 5.69 (d, *J* = 4.0 Hz, 1H), 4.88 – 4.77 (m, 1H), 4.78 – 4.67 (m, 2H). ^{13}C NMR (100 MHz, $CDCl_3$) δ 166.3, 165.9, 165.5, 153.1, 151.1, 133.80, 133.78, 133.2, 132.7, 132.5, 132.4, 130.3, 130.1, 130.0, 129.9, 129.7, 129.1, 128.9, 128.70, 128.68, 128.6, 128.5, 121.7, 120.1, 117.6, 104.5, 85.8, 83.6, 82.24, 82.18, 77.2, 63.7. HRMS (ESI) calcd for $C_{41}H_{31}NO_9Na$ $[M+Na]^+$ 704.1891, found 704.1890.

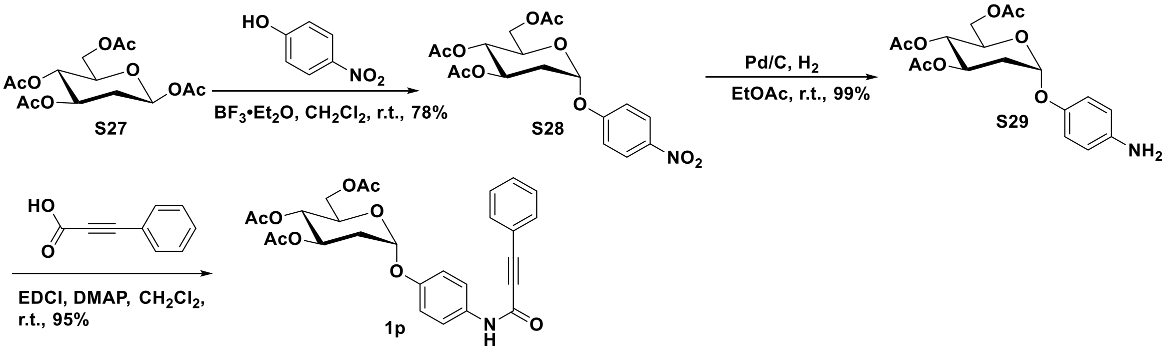

Fig. S17. Synthesis of the glycosyl PPAP donor **1p**.

***Para*-nitrophenyl 3,4,6-tri-*O*-acetyl-2-deoxyl-α-D-glucopyranoside (S28)**

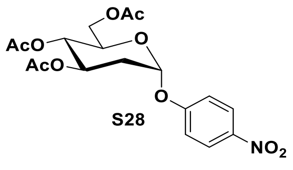

Compound **S28** was prepared from **S27** (2.0 g, 6.0 mmol) according to the synthesis of **S14** (**General procedure B**) to afford **S28** (1.9 g, 78%) as a white foam: $[\alpha]_D^{20}$ = 173.4 (*c* 0.12, $CHCl_3$); ^{1}H NMR (600 MHz, Chloroform-*d*) δ 8.19 (d, *J* = 9.0 Hz, 2H), 7.18 (d, *J* = 9.0 Hz, 2H), 5.77 (dd, *J* = 3.7, 1.3 Hz, 1H), 5.48 (ddd, *J* = 11.6, 9.4, 5.4 Hz, 1H), 5.10 (t, *J* = 9.9 Hz, 1H), 4.30 – 4.23 (m, 1H), 4.01 – 3.93 (m, 2H), 2.51 (ddd, *J* = 13.4, 5.5, 1.4 Hz, 1H), 2.05 (s, 3H), δ 2.03 – 2.02 (m, 1H), 2.01 (s, 3H). ^{13}C NMR (150 MHz, $CDCl_3$) δ 170.5, 170.3, 169.8, 160.9, 142.7, 125.8, 116.3, 95.5, 77.2, 69.3, 68.7, 68.4, 61.9, 34.7, 21.0, 20.7. HRMS (ESI) calcd for $C_{18}H_{25}N_2O_{10}$ $[M+NH_4]^+$ 429.1504, found 429.1502.

***Para*-aminophenyl 3,4,6-tri-*O*-acetyl-2-deoxyl-α-D-glucopyranoside (S29)**

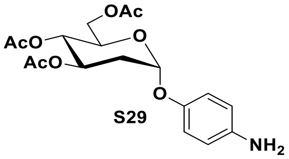

Similar procedure as that used for the synthesis of **S2** (**General procedure C**) was applied to the preparation of **S29** (1.5 g, 99%) as a white foam: $[\alpha]_D^{25}$ = 107.4 (c 0.32, $CHCl_3$); ^{1}H NMR (600 MHz, Chloroform-*d*) δ 6.89 (d, *J* = 8.4 Hz, 1H), 6.61 (d, *J* = 9.0 Hz, 2H), 5.54 – 5.46 (m, 2H), 5.06 (t, *J* = 10.2 Hz, 1H), 4.28 (dd, *J* = 12.0, 4.8 Hz, 1H), 4.10 (ddd, *J* = 10.2, 4.2, 1.8 Hz, 1H), 4.00 (dd, *J* = 12.0, 2.4 Hz, 1H), 3.47 (s, 2H), 2.43 (ddd, *J* = 13.2, 5.4, 1.8 Hz, 1H), 2.04 – 2.03 (m, 9H), 1.95 (ddd, *J* = 13.1, 11.6, 3.6 Hz, 1H). ^{13}C NMR (150 MHz, $CDCl_3$) δ 170.7, 170.3, 170.0, 149.1, 141.6, 117.8, 116.1, 96.1, 69.2, 69.0, 68.3, 62.2, 35.2, 21.0, 20.7. HRMS (ESI) calcd for $C_{18}H_{27}N_2O_8$ $[M+NH_4]^+$ 399.1762, found 399.1767.

***Para*-(3-Phenylpropiolamido) phenyl 3,4,6-tri-*O*-acetyl-2-deoxyl-α-D-glucopyranoside (1p)**

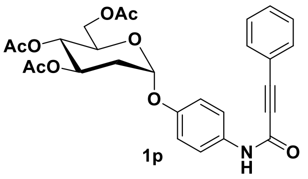

Compound **1p** was prepared from **S29** (1.3 g, 3.4 mmol) according to the synthesis of **1a** (**General procedure D**) and purified by column chromatography (Petroleum ether/EtOAc = 2: 1) to afford **1p** (1.64 g, 95%) as a light yellow solid: $[\alpha]_D^{25}$ = 105.8 (c 1.42, $CHCl_3$); ^{1}H NMR (600 MHz, Chloroform-*d*) δ 7.62 (s, 1H), 7.59 – 7.54 (m, 2H), 7.52 – 7.46 (m, 2H), 7.47 – 7.41 (m, 1H), 7.40 – 7.35 (m, 2H), 7.10 – 7.04 (m, 2H), 5.64 (d, *J* = 2.7 Hz, 2H), 5.51 (ddd, *J* = 11.5, 9.4, 5.4 Hz, 1H), 5.09 (t, *J* = 9.8 Hz, 1H), 4.29 (dd, *J* = 12.2, 4.8 Hz, 1H), 4.04 (ddd, *J* = 10.2, 4.7, 2.2 Hz, 1H), 3.99 (dd, *J* = 12.2, 2.3 Hz, 1H), 2.47 (ddd, *J* = 13.2, 5.5, 1.4 Hz, 1H), 2.07 – 2.03 (m, 9H), 1.99 (ddd, *J* = 13.1, 11.5, 3.6 Hz, 1H). ^{13}C NMR (150 MHz, $CDCl_3$) δ 170.8, 170.4, 170.1, 153.3, 151.0, 132.7, 132.1, 130.5, 128.7, 121.7, 120.1, 117.0, 95.6, 85.9, 83.5, 77.2, 69.2, 69.0, 68.8, 62.2, 35.1, 21.1, 20.9. HRMS (ESI) calcd for $C_{27}H_{31}N_2O_9$ $[M+NH_4]^+$ 527.2024, found 527.2024.

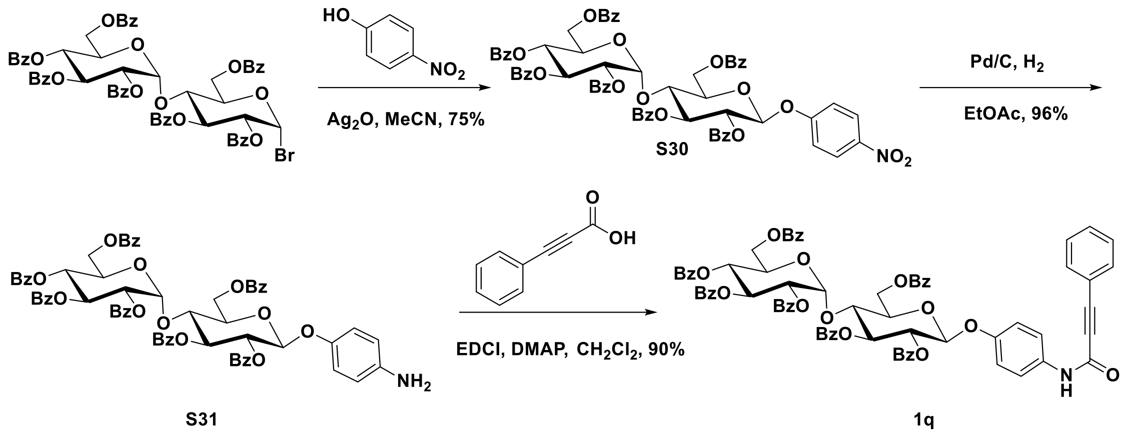

Fig. S18. Synthesis of the glycosyl PPAP donor **1q**.

***Para*-nitrophenyl 2,3,6,2',3',4',6'-hepta-*O*-benzoyl-β-D-maltoside (S30)**

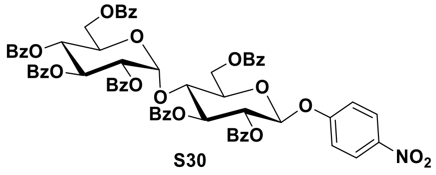

Similar procedure as that used for the synthesis of **S1** (**General procedure A**) was applied to the preparation of **S30** (1.78 g, 75%) as a white foam: $[\alpha]_D^{25}$= 55.3 (*c* 0.56, CH_2Cl_2); ^{1}H NMR (400 MHz, Chloroform-*d*) δ 8.04 – 7.92 (m, 6H), 7.94 – 7.84 (m, 4H), 7.80 – 7.66 (m, 6H), 7.58 (t, *J* = 7.5 Hz, 1H), 7.56 – 7.16 (m, 22H), 7.01 (d, *J* = 9.2 Hz, 2H), 6.14 (t, *J* = 10.0 Hz, 1H), 5.82 – 5.75 (m, 2H), 5.70 (t, *J* = 9.6 Hz, 1H), 5.62 – 5.51 (m, 2H), 5.31 (dd, *J* = 10.4, 3.6 Hz, 1H), 5.01 (dd, *J* = 12.0, 2.8 Hz, 1H), 4.70 (dd, *J* = 12.0, 5.6 Hz, 1H), 4.63 – 4.50 (m, 3H), 4.45 – 4.35 (m, 2H). ^{13}C NMR (150 MHz, $CDCl_3$) δ 166.0, 165.9, 165.8, 165.6, 165.2, 165.1, 165.0, 161.0, 143.0, 133.7, 133.62, 133.59, 133.56, 133.5, 133.3, 130.0, 129.9, 129.8, 129.74, 129.70, 129.5, 129.2, 128.9, 128.74, 128.66, 128.6, 128.5, 128.44, 128.39, 128.3, 128.2, 125.8, 116.7, 97.5, 97.1, 77.2, 74.8, 73.7, 73.4, 71.9, 71.0, 69.9, 69.5, 69.2, 63.2, 62.6. HRMS (ESI) calcd for $C_{67}H_{57}N_2O_{20}$ $[M+NH_4]^+$ 1209.3499, found 1209.3501.

***Para*-aminophenyl 2,3,6,2',3',4',6'-hepta-*O*-benzoyl-β-D-maltoside (S31)**

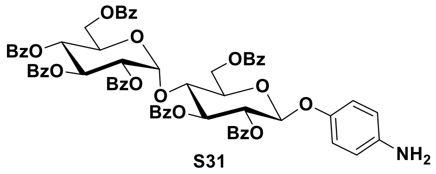

Similar procedure as that used for the synthesis of **S2** (**General procedure C**) was applied to the preparation of **S31** (1.39 g, 96%) as a white foam: $[\alpha]_D^{25}$= 62.4 (*c* 0.56, CH_2Cl_2); ^{1}H NMR (600 MHz, Chloroform-*d*) δ 8.08 (d, *J* = 7.2 Hz, 2H), 8.02 (d, *J* = 7.2 Hz, 2H), 7.87 (dd, *J* = 15.0, 7.2 Hz, 3H), 7.73 (t, *J* = 8.4 Hz, 4H), 7.66 (d, *J* = 7.2 Hz, 2H), 7.57 (t, *J* = 7.2 Hz, 1H), 7.52 (t, *J* = 7.2 Hz, 1H), 7.50 – 7.38 (m, 8H), 7.38 – 7.28 (m, 5H), 7.28 – 7.21 (m, 5H), 7.19 (t, *J* = 7.8 Hz, 2H), 6.80 (d, *J* = 8.4 Hz, 2H), 6.47 (d, *J* = 8.4 Hz, 2H), 6.10 (t, *J* = 10.2 Hz, 1H), 5.80 (t, *J* = 9.6 Hz, 1H), 5.76 (d, *J* = 4.2 Hz, 1H), 5.67 (t, *J* = 9.6 Hz, 1H), 5.53 (dd, *J* = 9.0, 7.8 Hz, 1H), 5.28 (dd, *J* = 10.2, 3.6 Hz, 1H), 5.19 (d, *J* = 7.2 Hz, 1H), 4.97 (dd, *J* = 12.0, 3.0 Hz, 1H), 4.74 (dd, *J* = 12.0, 5.4 Hz, 1H), 4.55 (t, *J* = 9.0 Hz, 1H), 4.52 – 4.48 (m, 1H), 4.45 (dd, *J* = 12.0, 3.0 Hz, 1H), 4.34 (dd, *J* = 12.6, 4.2 Hz, 1H), 4.24 – 4.18 (m, 1H), 3.46 (s, 2H). ^{13}C NMR (150 MHz, $CDCl_3$) δ 166.2, 166.0, 165.7, 165.5, 165.3, 165.2, 165.1, 149.9, 142.4, 133.5, 133.4, 133.34, 133.26, 133.22, 133.19,

130.04, 130.00, 129.93, 129.86, 129.8, 129.7, 129.6, 129.5, 129.2, 128.9, 128.8, 128.7, 128.65, 128.57, 128.5, 128.51, 128.48, 128.32, 128.29, 128.2, 119.2, 116.0, 100.6, 96.7, 77.2, 75.1, 73.5, 73.0, 72.3, 70.9, 70.0, 69.3, 69.1, 63.6, 62.6. HRMS (ESI) calcd for $C_{67}H_{55}NO_{18}Na$ $[M+Na]^+$ 1184.3311, found 1184.3314.

***Para*-(3-Phenylpropiolamido) phenyl 2,3,6,2',3',4',6'-hepta-*O*-benzoyl-β-D-maltoside (1q)**

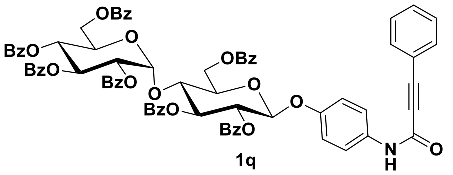

Compound **1q** was prepared from **S31** (1.2 g, 1.0 mmol) according to the synthesis of **1a** (**General procedure D**) and purified by column chromatography (Petroleum ether/EtOAc = 4: 1) to afford **1q** (1.2 g, 90%) as a light yellow solid: $[\alpha]_D^{25}$= 63.7 (*c* 1.73, CH_2Cl_2); ^1H NMR (600 MHz, Chloroform-*d*) δ 8.06 (d, *J* = 7.8 Hz, 2H), 8.03 (d, *J* = 7.8 Hz, 2H), 7.91 (d, *J* = 7.2 Hz, 2H), 7.89 – 7.84 (m, 2H), 7.80 – 7.71 (m, 5H), 7.68 (d, *J* = 7.8 Hz, 2H), 7.60 (t, *J* = 7.2 Hz, 1H), 7.56 – 7.51 (m, 3H), 7.52 – 7.29 (m, 19H), 7.28 – 7.23 (m, 5H), 7.21 (t, *J* = 7.8 Hz, 2H), 6.96 (d, *J* = 9.0 Hz, 2H), 6.12 (td, *J* = 10.2, 2.4 Hz, 1H), 5.81 (t, *J* = 8.4 Hz, 1H), 5.77 (d, *J* = 4.2 Hz, 1H), 5.69 (t, *J* = 10.2 Hz, 1H), 5.57 (t, *J* = 9.0 Hz, 1H), 5.32 (d, *J* = 7.2 Hz, 1H), 5.31 – 5.28 (m, 1H), 4.99 (dd, *J* = 12.0, 3.0 Hz, 1H), 4.70 (dd, *J* = 12.0, 6.0 Hz, 1H), 4.58 – 4.46 (m, 3H), 4.38 (dd, *J* = 12.0, 3.6 Hz, 1H), 4.28 – 4.21 (m, 1H). ^{13}C NMR (150 MHz, $CDCl_3$) δ 166.2, 166.0, 165.8, 165.6, 165.3, 165.2, 165.1, 153.8, 150.9, 133.7, 133.5, 133.4, 133.3, 133.2, 132.9, 132.7, 130.4, 130.0, 129.88, 129.86, 129.8, 129.7, 129.6, 129.4, 129.01, 128.95, 128.8, 128.77, 128.74, 128.70, 128.53, 128.50, 128.46, 128.4, 128.3, 128.2, 121.4, 120.0, 117.9, 99.3, 96.8, 85.8, 83.5, 77.2, 75.0, 73.7, 73.2, 72.2, 71.0, 70.0, 69.4, 69.2, 63.6, 62.6. HRMS (ESI) calcd for $C_{76}H_{59}NO_{19}Na$ $[M+Na]^+$1312.3573, found 1312.3575.

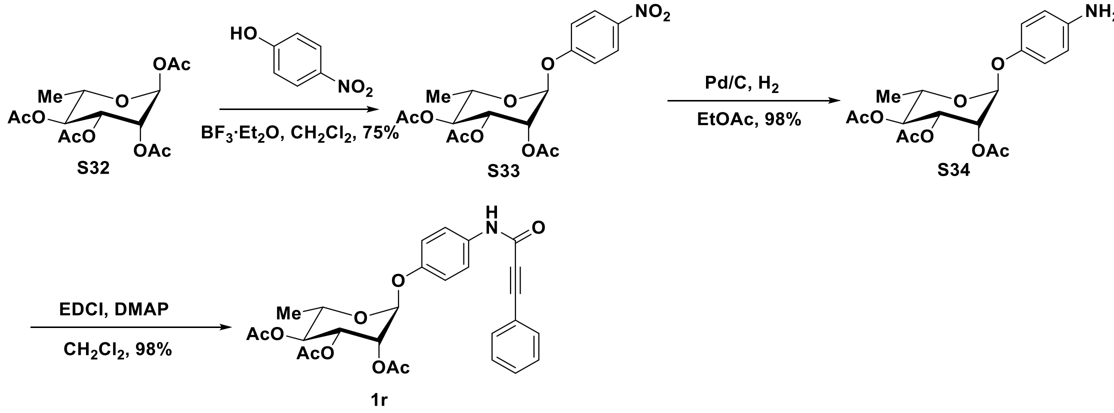

Fig. S19. Synthesis of the glycosyl PPAP donor **1r**.

***Para*-nitrophenyl 2,3,4-tri-*O*-acetyl-α-L-rhamnopyranoside (S33)**

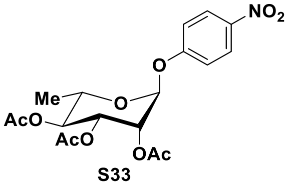

Compound **S33** was prepared from **S32** (1.67 g, 5.0 mmol) according to the synthesis of **S14** (**General procedure B**) to afford **S33** (1.54 g, 75%) as a white foam: $[\alpha]_D^{20}$= -91.4 (*c* 1.22, $CHCl_3$); ^1H NMR (600 MHz, Chloroform-*d*) δ 8.21 (d, *J* = 9.2 Hz, 2H), 7.18 (d, *J* = 9.3 Hz, 2H), 5.56 (d, *J*

= 1.8 Hz, 1H), 5.48 (dd, J = 10.1, 3.5 Hz, 1H), 5.44 (dd, J = 3.6, 1.9 Hz, 1H), 5.17 (t, J = 9.9 Hz, 1H), 3.94 – 3.86 (m, 1H), 2.20 (s, 3H), 2.05 (s, 3H), 2.03 (s, 3H), 1.21 (d, J = 6.2 Hz, 3H). ^{13}C NMR (150 MHz, $CDCl_3$) δ 170.1, 169.9, 160.6, 143.2, 126.0, 116.5, 95.9, 77.2, 70.7, 69.4, 68.8, 68.0, 20.9, 20.84, 20.79, 17.6. HRMS (ESI) calcd for $C_{18}H_{21}NO_{10}Na$ $[M+Na]^+$ 434.1058, found 434.1053.

***Para*-aminophenyl 2,3,4-tri-*O*-acetyl-α-L-rhamnopyranoside (S34)**

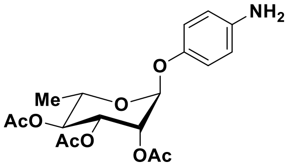

S34

Similar procedure as that used for the synthesis of **S2** (**General procedure C**) was applied to the preparation of **S34** (1.27 g, 98%) as a white foam: $[\alpha]_D^{20}$= -67.0 (*c* 0.28, $CHCl_3$); ^{1}H NMR (600 MHz, Chloroform-*d*) δ 6.88 (d, J = 8.8 Hz, 2H), 6.62 (d, J = 8.8 Hz, 2H), 5.49 (dd, J = 10.1, 3.5 Hz, 1H), 5.40 (dd, J = 3.5, 1.9 Hz, 1H), 5.29 (d, J = 1.8 Hz, 1H), 5.13 (t, J = 10.0 Hz, 1H), 4.07 – 3.99 (m, 1H), 3.50 (s, 2H), 2.17 (s, 3H), 2.05 (s, 3H), 2.02 (s, 3H), 1.20 (d, J = 6.3 Hz, 3H). ^{13}C NMR (100 MHz, $CDCl_3$) δ 170.3, 170.2, 149.0, 142.0, 118.0, 116.2, 96.8, 77.2, 71.2, 70.0, 69.1, 67.0, 21.1, 21.0, 20.9, 17.6. HRMS (ESI) calcd for $C_{18}H_{24}NO_8$ $[M+H]^+$ 382.1496, found 382.1499.

***Para*-(3-Phenylpropiolamido) phenyl 2,3,4-tri-*O*-acetyl-α-L-rhamnopyranoside (1r)**

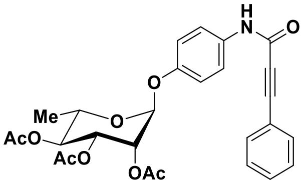

1r

Compound **1r** was prepared from **S34** (1.2 g, 3.1 mmol) according to the synthesis of **1a** (**General procedure D**) and purified by column chromatography (Petroleum ether/EtOAc = 3: 1) to afford **1r** (1.54 g, 98%) as a light yellow solid: $[\alpha]_D^{20}$= -71.9 (*c* 0.56, $CHCl_3$);^{1}H NMR (600 MHz, Chloroform-*d*) δ 7.69 (s, 1H), 7.58 – 7.54 (m, 2H), 7.50 (d, J = 9.0 Hz, 2H), 7.48 – 7.40 (m, 1H), 7.37 (t, J = 7.5 Hz, 2H), 7.05 (d, J = 9.1 Hz, 2H), 5.49 (dd, J = 10.1, 3.4 Hz, 1H), 5.44 – 5.39 (m, 2H), 5.15 (t, J = 10.0 Hz, 1H), 4.01 – 3.93 (m, 1H), 2.19 (s, 3H), 2.06 (s, 3H), 2.03 (s, 3H), 1.20 (d, J = 6.2 Hz, 3H). ^{13}C NMR (150 MHz, $CDCl_3$) δ 170.2, 170.2, 170.1, 153.0, 151.1, 132.7, 132.4, 130.5, 128.7, 121.7, 120.1, 117.1, 96.0, 85.9, 83.5, 77.2, 71.0, 69.8, 69.0, 67.3, 21.0, 20.93, 20.88, 17.6. HRMS (ESI) calcd for $C_{27}H_{27}NO_9Na$ $[M+Na]^+$ 532.1578, found 532.1573.

Glycosylation reactions with PPAP glycosides as donors.

acceptors (2a-2l, 5a-5f)

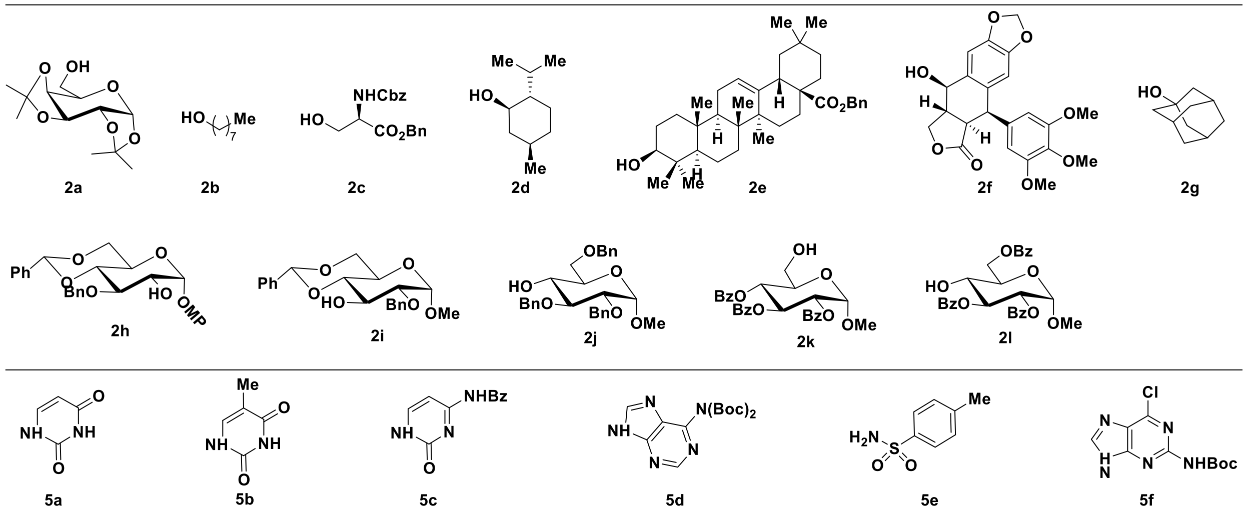

Compounds **2a-2d, 2f-2g, 5a-5e** are commercially available. Compounds **2e**(*67*), **2h**(*68*), **2i**(*69*), **2j**(*70*), **2k**(*71*), **2l**(*72*), and **5f**(*73*) were synthesized following literature procedures.

O- and *N*-Glycosylations with PPAP glycoside donors.

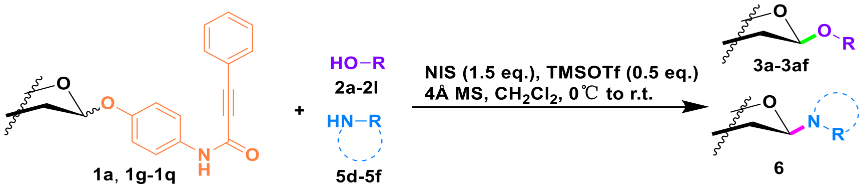

Note: (1) The amount of donor and TMSOTf were increased to 1.5 eq. and 1.0 eq., respectively for the synthesis of products **3a**, **3c**, **3k** and **3aa**. (2) The amount of TMSOTf was increased to 1.0 eq. for **3l**.

General procedure E: A solution of glycosyl PPAP donor **1a, 1g-1q** (1.2 eq.) and acceptor **2a-2l, 5d, 5e, 5f** (1.0 eq.) in dry CH_2Cl_2 (0.1 M) was stirred at room temperature for 30 min in the presence of activated 4 Å MS (3.0 g/mmol) under N_2 atmosphere. Then the vessel was chilled to 0 °C, to which NIS (1.5 eq.) and TMSOTf (0.5 eq.) were added. The reaction mixture was stirred for 3 h after the temperature gradually rise to room temperature. Then Et_3N was added to quench the reaction and the solvent was removed under reduced pressure. The resulting residue was purified by silica gel column chromatography to afford the glycosylated product.

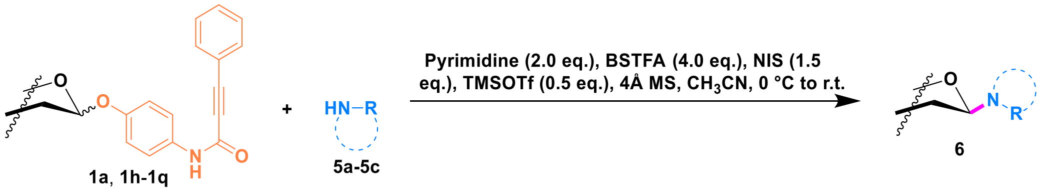

General procedure F: BSTFA (4.0 eq) was added to a stirred suspension of acceptor **5a-5c** (2.0 eq.) in dry CH_3CN (0.033 M) under N_2 atmosphere. After the mixture became clear, this solution was added to a solution of glycosyl donor **1** (1.0 eq) and activated 4Å MS (4.0 g/mmol) in dry

CH_3CN (0.05 M), which has been stirred at room temperature for 30 min under N_2 atmosphere. The stirring was continued for 30 min, then the vessel was chilled to 0 °C, which NIS (1.5 eq.) and TMSOTf (0.5 eq.) were added. The reaction mixture was warmed up and stirred for 3 h at room temperature. Et_3N was added to quench the reaction and the solvent was removed under reduced pressure. The resulting residue was purified by silica gel column chromatography to afford the glycosylated product.
Note: (1) The reaction of **6f**, **6h** and **6p** were performed in a mixture of solvents (CH_2Cl_2/MeCN = 3:1). (2) The reaction of **6k** was performed at −20 °C to r.t..

Compound 3b(*74*)

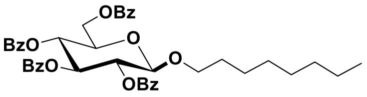

3b

Glycosylation of **1a** (122.0 mg, 0.15 mmol) with **2b** (13.0 mg, 0.10 mmol) according to **General procedure E** afforded **3b** (63.7 mg, 90%) as a white solid: ^{1}H NMR (600 MHz, Chloroform-*d*) δ 8.04 – 7.99 (m, 2H), 7.96 (dd, *J* = 8.1, 1.3 Hz, 2H), 7.90 (dd, *J* = 8.1, 1.3 Hz, 2H), 7.83 (dd, *J* = 8.0, 1.3 Hz, 2H), 7.57 – 7.46 (m, 3H), 7.45 – 7.31 (m, 7H), 7.28 (t, *J* = 7.7 Hz, 2H), 5.90 (t, *J* = 9.6 Hz, 1H), 5.67 (t, *J* = 9.7 Hz, 1H), 5.52 (dd, *J* = 9.8, 7.8 Hz, 1H), 4.83 (d, *J* = 7.8 Hz, 1H), 4.63 (dd, *J* = 12.0, 3.3 Hz, 1H), 4.51 (dd, *J* = 12.1, 5.2 Hz, 1H), 4.15 (ddd, *J* = 9.1, 5.2, 3.3 Hz, 1H), 3.92 (dt, *J* = 9.7, 6.2 Hz, 1H), 3.53 (dt, *J* = 9.7, 6.7 Hz, 1H), 1.58 – 1.44 (m, 2H), 1.23 – 1.00 (m, 10H), 0.82 (t, *J* = 7.3 Hz, 3H). ^{13}C NMR (150 MHz, $CDCl_3$) δ 166.3, 166.0, 165.4, 165.2, 133.6, 133.4, 133.3, 133.2, 130.0, 129.91, 129.88, 129.8, 129.5, 128.98, 128.96, 128.54, 128.47, 128.4, 101.4, 77.2, 73.1, 72.3, 72.0, 70.5, 70.0, 63.4, 31.8, 29.5, 29.34, 29.25, 25.9, 22.7, 14.2. HRMS (ESI) calcd for $C_{42}H_{44}O_{10}Na$ $[M+Na]^+$ 731.2827, found 731.2826.

Compound 3c(*75*)

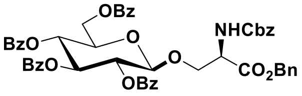

3c

Glycosylation of **1a** (98.0 mg, 0.12 mmol) with **2c** (32.9 mg, 0.10 mmol) according to **General procedure E** afforded **3c** (77.5 mg, 85%) as a white solid: ^{1}H NMR (400 MHz, Chloroform-*d*) δ 8.04 – 7.97 (m, 2H), 7.97 – 7.86 (m, 4H), 7.87 – 7.79 (m, 2H), 7.58 – 7.24 (m, 22H), 5.86 (t, *J* = 9.6 Hz, 1H), 5.64 (t, *J* = 9.7 Hz, 1H), 5.56 (d, *J* = 8.2 Hz, 1H), 5.45 (dd, *J* = 9.8, 7.8 Hz, 1H), 5.21 – 5.08 (m, 2H), 5.03 (d, *J* = 12.2 Hz, 1H), 4.95 (d, *J* = 12.3 Hz, 1H), 4.79 (d, *J* = 7.8 Hz, 1H), 4.61 (dd, *J* = 12.2, 3.2 Hz, 1H), 4.54 – 4.49 (m, 1H), 4.43 (dd, *J* = 12.2, 5.1 Hz, 1H), 4.38 (dd, *J* = 10.3, 2.9 Hz, 1H), 4.07 – 3.96 (m, 1H), 3.91 (dd, *J* = 10.3, 3.5 Hz, 1H).

Compound 3d(*24*)

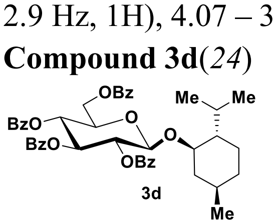

3d

Glycosylation of **1a** (122.0 mg, 0.15 mmol) with **2d** (15.6 mg, 0.10 mmol) according to **General procedure E** afforded **3d** (67.5 mg, 92%) as a white solid: ^{1}H NMR (400 MHz, Chloroform-*d*) δ 8.07 – 7.79 (m, 8H), 7.58 – 7.44 (m, 3H), 7.47 – 7.26 (m, 9H), 5.89 (t, *J* = 9.7 Hz, 1H), 5.63 (t, *J* = 9.7 Hz, 1H), 5.48 (dd, *J* = 9.8, 7.9 Hz, 1H), 4.93 (d, *J* = 7.9 Hz, 1H), 4.62 (dd, *J* = 12.0, 3.4 Hz,

1H), 4.48 (dd, $J = 12.0$, 5.7 Hz, 1H), 4.21 – 4.08 (m, 1H), 3.48 (td, $J = 10.7$, 4.3 Hz, 1H), 2.34 – 2.19 (m, 1H), 2.01 – 1.89 (m, 1H), 1.57 – 1.52 (m, 2H), 1.36 – 1.07 (m, 2H), 0.94 – 0.63 (m, 12H). ^{13}C NMR (100 MHz, $CDCl_3$) δ 166.3, 166.0, 165.5, 165.2, 133.5, 133.3, 133.2, 133.2, 130.0, 129.9, 129.9, 129.8, 129.7, 129.1, 129.0, 128.5, 128.4, 99.1, 79.2, 77.2, 73.4, 72.3, 72.2, 70.4, 63.6, 47.5, 40.9, 34.2, 31.5, 25.3, 23.2, 22.2, 20.9, 15.8.

Compound 3e(*38*)

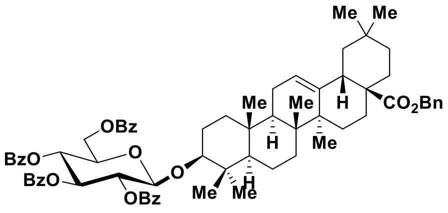

3e

Glycosylation of **1a** (98.0 mg, 0.12 mmol) with **2e** (54.7 mg, 0.10 mmol) according to **General procedure E** afforded **3e** (83.2 mg, 74%) as a white solid: ^{1}H NMR (600 MHz, Chloroform-*d*) δ 8.04 – 7.99 (m, 2H), 7.96 – 7.89 (m, 4H), 7.85 – 7.80 (m, 2H), 7.57 – 7.51 (m, 1H), 7.53 – 7.46 (m, 2H), 7.45 – 7.30 (m, 11H), 7.32 – 7.25 (m, 3H), 5.91 (t, $J = 9.7$ Hz, 1H), 5.63 – 5.53 (m, 2H), 5.29 (t, $J = 3.7$ Hz, 1H), 5.11 – 5.00 (m, 2H), 4.85 (d, $J = 8.0$ Hz, 1H), 4.62 – 4.50 (m, 2H), 4.14 (ddd, $J = 9.9$, 6.6, 3.4 Hz, 1H), 3.09 (dd, $J = 11.8$, 4.5 Hz, 1H), 2.91 (dd, $J = 14.0$, 4.5 Hz, 1H), 1.96 (td, $J = 13.6$, 4.1 Hz, 1H), 1.85–0.57 (m, 21H), 1.08 (s, 3H), 0.92 (d, $J = 12.0$ Hz, 6H), 0.80 (s, 3H), 0.68 (s, 3H), 0.62 (s, 3H), 0.55 (s, 3H).

Compound 3f(*76*)

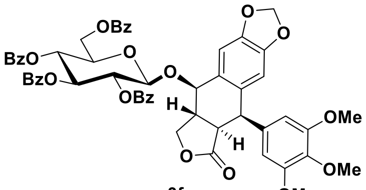

3f

Glycosylation of **1a** (98.0 mg, 0.12 mmol) with **2f** (41.4 mg, 0.10 mmol) according to **General procedure E** afforded **3f** (55.6 mg, 56%) as a white solid: ^{1}H NMR (600 MHz, Chloroform-*d*) δ 7.92 (td, $J = 8.2$, 1.4 Hz, 4H), 7.90 – 7.84 (m, 2H), 7.81 (dd, $J = 8.3$, 1.4 Hz, 2H), 7.55 – 7.47 (m, 3H), 7.45 – 7.39 (m, 1H), 7.39 – 7.31 (m, 6H), 7.26 (s, 2H), 7.12 (s, 1H), 6.48 (s, 1H), 6.34 (s, 2H), 5.97 – 5.90 (m, 3H), 5.70 – 5.59 (m, 2H), 4.99 (dd, $J = 11.8$, 8.9 Hz, 2H), 4.69 (dd, $J = 12.3$, 2.9 Hz, 1H), 4.55 – 4.44 (m, 3H), 4.19 (ddd, $J = 9.4$, 6.0, 2.9 Hz, 1H), 4.06 (dd, $J = 10.4$, 8.6 Hz, 1H), 3.73 (s, 6H), 3.67 (s, 3H), 2.97 – 2.87 (m, 1H), 2.72 (dd, $J = 14.4$, 4.7 Hz, 1H).

Compound 3g(*31*)

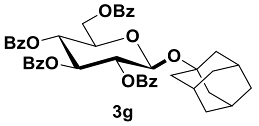

3g

Glycosylation of **1a** (98.0 mg, 0.12 mmol) with **2g** (15.2 mg, 0.10 mmol) according to **General procedure E** afforded **3g** (67.0 mg, 92%) as a white solid: ^{1}H NMR (600 MHz, Chloroform-*d*) δ 8.04 – 7.99 (m, 2H), 7.98 – 7.94 (m, 2H), 7.93 – 7.89 (m, 2H), 7.85 – 7.81 (m, 2H), 7.56 – 7.47 (m, 3H), 7.45 – 7.32 (m, 7H), 7.28 (t, $J = 7.8$ Hz, 2H), 5.92 (t, $J = 9.6$ Hz, 1H), 5.56 (t, $J = 9.7$ Hz, 1H), 5.49 (dd, $J = 9.8$, 8.0 Hz, 1H), 5.13 (d, $J = 8.0$ Hz, 1H), 4.58 (dd, $J = 11.9$, 3.1 Hz, 1H), 4.49 (dd, $J = 11.9$, 7.2 Hz, 1H), 4.22 – 4.15 (m, 1H), 2.05 – 1.98 (m, 3H), 1.85 – 1.79 (m, 3H), 1.69 – 1.62 (m, 3H), 1.59 – 1.52 (m, 3H), 1.52 – 1.45 (m, 3H).

Compound 3h

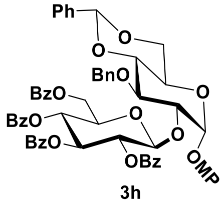

3h

Glycosylation of **1a** (98.0 mg, 0.12 mmol) with **2h** (46.4 mg, 0.10 mmol) according to **General procedure E** afforded **3h** (93.8 mg, 90%) as a white solid: $[\alpha]_D^{20}$= 20.0 (*c* 0.91, $CHCl_3$). ^{1}H NMR (600 MHz, Chloroform-*d*) δ 8.01 – 7.96 (m, 2H), 7.92 (ddd, *J* = 10.0, 8.4, 1.4 Hz, 4H), 7.84 – 7.79 (m, 2H), 7.59 – 7.52 (m, 1H), 7.53 – 7.47 (m, 1H), 7.46 – 7.38 (m, 6H), 7.37 – 7.32 (m, 5H), 7.30 – 7.23 (m, 5H), 7.22 – 7.15 (m, 3H), 7.10 – 7.00 (m, 4H), 6.73 (d, *J* = 9.0 Hz, 2H), 5.91 (t, *J* = 9.6 Hz, 1H), 5.69 – 5.62 (m, 2H), 5.59 (d, *J* = 3.6 Hz, 1H), 5.52 (s, 1H), 5.27 (d, *J* = 7.8 Hz, 1H), 4.63 (d, *J* = 11.5 Hz, 1H), 4.58 (dd, *J* = 12.1, 3.0 Hz, 1H), 4.48 (d, *J* = 11.4 Hz, 1H), 4.38 (dd, *J* = 12.1, 5.4 Hz, 1H), 4.25 (dd, *J* = 10.3, 4.9 Hz, 1H), 4.17 – 4.09 (m, 2H), 4.06 (td, *J* = 10.0, 5.0 Hz, 1H), 3.92 (dd, *J* = 9.3, 3.7 Hz, 1H), 3.74 (s, 3H), 3.69 (t, *J* = 10.4 Hz, 1H), 3.62 (t, *J* = 9.5 Hz, 1H). ^{13}C NMR (150 MHz, $CDCl_3$) δ 166.1, 165.9, 165.2, 165.1, 155.4, 151.1, 138.5, 137.3, 133.6, 133.34, 133.30, 133.2, 129.91, 129.86, 129.8, 129.5, 129.2, 129.0, 128.80, 128.78, 128.5, 128.4, 128.2, 127.52, 127.47, 126.1, 119.0, 114.6, 102.3, 101.5, 99.5, 82.0, 80.1, 77.6, 77.4, 77.2, 76.9, 75.0, 73.1, 72.4, 72.1, 69.6, 69.0, 63.0, 62.8, 60.5, 55.6, 14.3.HRMS (ESI) calcd for $C_{61}H_{58}NO_{16}$ $[M+NH_4]^+$ 1060.3750, found 1060.3752.

Compound 3i(*77*)

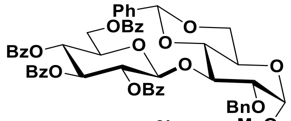

3i

Glycosylation of **1a** (98.0 mg, 0.12 mmol) with **2i** (37.2 mg, 0.10 mmol) according to **General procedure E** afforded **3i** (94.3 mg, 99%) as a white solid: ^{1}H NMR (600 MHz, Chloroform-*d*) δ 7.88 (ddd, *J* = 14.1, 8.3, 1.4 Hz, 4H), 7.80 – 7.75 (m, 2H), 7.74 – 7.69 (m, 2H), 7.47 – 7.34 (m, 5H), 7.34 – 7.28 (m, 1H), 7.24 (ddd, *J* = 15.7, 14.4, 7.2 Hz, 6H), 7.22 – 7.12 (m, 8H), 7.02 (dd, *J* = 7.4, 2.1 Hz, 2H), 5.80 (t, *J* = 9.6 Hz, 1H), 5.66 – 5.55 (m, 2H), 5.48 (s, 1H), 5.18 (d, *J* = 7.9 Hz, 1H), 4.51 (d, *J* = 12.5 Hz, 1H), 4.42 (dd, *J* = 12.0, 3.5 Hz, 1H), 4.26 – 4.17 (m, 3H), 4.17 – 4.08 (m, 2H), 3.88 (ddd, *J* = 9.9, 4.9, 3.5 Hz, 1H), 3.69 (td, *J* = 9.9, 4.6 Hz, 1H), 3.61 (t, *J* = 10.2 Hz, 1H), 3.53 (t, *J* = 9.3 Hz, 1H), 3.35 (dd, *J* = 9.2, 3.8 Hz, 1H), 3.18 (s, 3H).

Compound 3j(*78*)

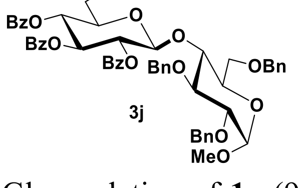

3j

Glycosylation of **1a** (98.0 mg, 0.12 mmol) with **2j** (46.4 mg, 0.10 mmol) according to **General procedure E** afforded **3j** (103.0 mg, 99%) as a white solid: ^{1}H NMR (600 MHz, Chloroform-*d*) δ 7.96 (dd, *J* = 8.3, 1.4 Hz, 2H), 7.88 (d, *J* = 8.4 Hz, 4H), 7.79 (dd, *J* = 8.3, 1.4 Hz, 2H), 7.55 – 7.45 (m, 5H), 7.47 – 7.30 (m, 13H), 7.29 – 7.23 (m, 6H), 7.22 – 7.15 (m, 3H), 5.62 (t, *J* = 9.6 Hz, 1H), 5.55 (t, *J* = 9.6 Hz, 1H), 5.46 (dd, *J* = 9.7, 8.0 Hz, 1H), 5.07 (d, *J* = 11.2 Hz, 1H), 4.83 – 4.71 (m, 4H), 4.58 (d, *J* = 12.3 Hz, 1H), 4.55 (d, *J* = 3.7 Hz, 1H), 4.40 (dd, *J* = 12.1, 3.4 Hz, 1H), 4.34 (d, *J*

= 12.1 Hz, 1H), 4.26 (dd, *J* = 12.0, 5.1 Hz, 1H), 3.97 (dd, *J* = 10.0, 8.9 Hz, 1H), 3.88 (t, *J* = 9.2 Hz, 1H), 3.76 – 3.67 (m, 2H), 3.50 (dt, *J* = 9.9, 2.4 Hz, 1H), 3.46 (dd, *J* = 9.5, 3.7 Hz, 1H), 3.43 (dd, *J* = 10.8, 2.0 Hz, 1H), 3.27 (s, 3H).

Compound 3k(*78*)

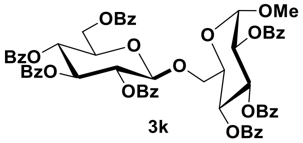

3k

Glycosylation of **1a** (98.0 mg, 0.12 mmol) with **2k** (50.6 mg, 0.10 mmol) according to **General procedure E** afforded **3k** (106.3 mg, 98%) as a white solid: ^{1}H NMR (400 MHz, Chloroform-*d*) δ 7.99 (t, *J* = 8.2 Hz, 4H), 7.94 (d, *J* = 7.8 Hz, 2H), 7.91 – 7.76 (m, 8H), 7.62 – 7.27 (m, 20H), 6.08 (t, *J* = 9.8 Hz, 1H), 5.93 (t, *J* = 9.6 Hz, 1H), 5.66 (t, *J* = 9.7 Hz, 1H), 5.57 (t, *J* = 8.8 Hz, 1H), 5.33 (t, *J* = 9.9 Hz, 1H), 5.10 (dd, *J* = 10.3, 3.5 Hz, 1H), 4.99 (d, *J* = 7.8 Hz, 1H), 4.95 (d, *J* = 3.6 Hz, 1H), 4.62 (dd, *J* = 12.2, 2.9 Hz, 1H), 4.46 (dd, *J* = 12.3, 5.1 Hz, 1H), 4.28 – 4.18 (m, 1H), 4.19 – 4.07 (m, 2H), 3.80 (dd, *J* = 11.4, 7.6 Hz, 1H), 3.11 (s, 3H).

Compound 3l(*79*)

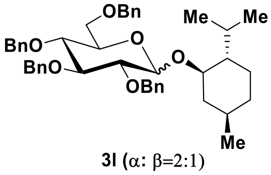

3l (α: β=2:1)

Glycosylation of **1g** (114.0 mg, 0.15 mmol) with **2d** (15.6 mg, 0.10 mmol) according to **General procedure E** afforded **3l** (106.3 mg, 99%, α:β=2:1) as a white solid: β: ^{1}H NMR (600 MHz, Chloroform-*d*) δ 7.38 – 7.25 (m, 18H), 7.20 (dd, *J* = 7.6, 1.8 Hz, 2H), 4.94 (dd, *J* = 16.1, 10.9 Hz, 2H), 4.81 (dd, *J* = 16.0, 10.9 Hz, 2H), 4.69 (d, *J* = 10.9 Hz, 1H), 4.64 – 4.52 (m, 3H), 4.48 (d, *J* = 7.9 Hz, 1H), 3.70 (d, *J* = 3.3 Hz, 2H), 3.67 – 3.57 (m, 2H), 3.50 (td, *J* = 10.7, 4.2 Hz, 1H), 3.45 – 3.39 (m, 2H), 2.41 – 2.31 (m, 1H), 2.14 (dt, *J* = 13.0, 3.2 Hz, 1H), 1.72 – 1.63 (m, 2H), 1.41 – 1.32 (m, 1H), 1.31 – 1.23 (m, 1H), 1.06 – 0.95 (m, 2H), 0.92 (dd, *J* = 12.2, 6.9 Hz, 6H), 0.89 – 0.84 (m, 1H), 0.83 (d, *J* = 6.9 Hz, 3H). ^{13}C NMR (150 MHz, $CDCl_3$) δ 139.0, 138.7, 138.5, 138.4, 128.53, 128.48, 128.4, 128.2, 127.93, 127.88, 127.79, 127.75, 127.7, 127.6, 100.9, 85.1, 82.4, 78.1, 77.9, 77.2, 75.7, 75.1, 75.0, 74.9, 73.8, 69.5, 48.3, 41.1, 34.6, 31.6, 25.4, 23.3, 22.4, 21.2, 16.1.

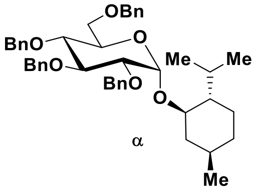

α

α:^{1}H NMR (600 MHz, Chloroform-*d*) δ 7.37 – 7.25 (m, 18H), 7.13 (dd, *J* = 7.5, 2.0 Hz, 2H), 5.02 (d, *J* = 3.7 Hz, 1H), 4.98 (d, *J* = 10.9 Hz, 1H), 4.83 (dd, *J* = 10.8, 7.3 Hz, 2H), 4.74 – 4.62 (m, 3H), 4.46 (dd, *J* = 11.4, 10.1 Hz, 2H), 4.01 (t, *J* = 9.3 Hz, 1H), 3.97 (ddd, *J* = 10.1, 3.9, 2.1 Hz, 1H), 3.75 (dd, *J* = 10.5, 3.8 Hz, 1H), 3.64 (ddd, *J* = 10.0, 5.3, 3.2 Hz, 2H), 3.55 (dd, *J* = 9.8, 3.6 Hz, 1H), 3.35 (td, *J* = 10.6, 4.4 Hz, 1H), 2.48 – 2.36 (m, 1H), 2.19 – 2.05 (m, 1H), 1.65 – 1.57 (m, 2H), 1.42 – 1.32 (m, 1H), 1.33 – 1.24 (m, 1H), 1.03 (q, *J* = 11.9 Hz, 1H), 0.94 (qd, *J* = 13.4, 12.7, 3.7 Hz, 1H), 0.85 (dd, *J* = 6.8, 3.0 Hz, 6H), 0.84 – 0.75 (m, 1H), 0.71 (d, *J* = 6.9 Hz, 3H). ^{13}C NMR (150 MHz, $CDCl_3$) δ 139.0, 138.5, 138.4, 138.2, 128.51, 128.49, 128.47, 128.4, 128.1, 128.0, 127.81, 127.79,

127.7, 127.6, 98.8, 82.1, 81.1, 80.7, 78.2, 77.2, 75.6, 75.2, 73.6, 73.3, 70.4, 68.8, 48.9, 43.2, 34.4, 31.9, 24.7, 23.1, 22.4, 21.3, 16.2.

Compound 3m(*33*)

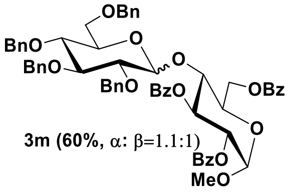

3m (60%, α: β=1.1:1)

Glycosylation of **1g** (92.0 mg, 0.12 mmol) with **2l** (50.6 mg, 0.10 mmol) according to **General procedure E** afforded **3m** (55.4 mg, 60%, α:β=1.1:1) as a white solid.

Compound 3n(*80*)

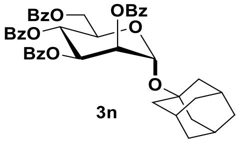

3n

Glycosylation of **1h** (98.0 mg, 0.12 mmol) with **2g** (15.2 mg, 0.10 mmol) according to **General procedure E** afforded **3n***78* (58.4 mg, 80%) as a white solid: ^{1}H NMR (600 MHz, Chloroform-*d*) δ 8.13 – 8.07 (m, 4H), 8.01 – 7.97 (m, 2H), 7.88 – 7.83 (m, 2H), 7.63 – 7.57 (m, 1H), 7.59 – 7.52 (m, 1H), 7.54 – 7.48 (m, 1H), 7.46 – 7.34 (m, 7H), 7.30 – 7.24 (m, 2H), 6.05 (t, *J* = 10.0 Hz, 1H), 5.99 (dd, *J* = 10.1, 3.1 Hz, 1H), 5.55 – 5.49 (m, 2H), 4.69 – 4.62 (m, 2H), 4.49 (dd, *J* = 12.1, 5.7 Hz, 1H), 2.13 (s, 3H), 2.01 – 1.86 (m, 6H), 1.62 (q, *J* = 12.5 Hz, 6H). ^{13}C NMR (150 MHz, $CDCl_3$) δ 166.3, 165.82, 165.77, 133.52, 133.50, 133.2, 133.1, 130.1, 129.99, 129.97, 129.8, 129.7, 129.4, 129.2, 128.7, 128.54, 128.48, 128.4, 91.1, 77.2, 76.2, 72.4, 70.4, 68.6, 67.6, 63.6, 42.5, 36.3, 30.8.

Compound 3o(*78*)

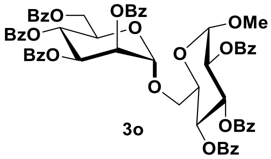

3o

Glycosylation of **1h** (98.0 mg, 0.12 mmol) with **2k** (50.6 mg, 0.10 mmol) according to **General procedure E** afforded **3o** (87.8 mg, 81%) as a white solid: ^{1}H NMR (600 MHz, Chloroform-*d*) δ 8.12 – 8.06 (m, 2H), 8.05 (dd, *J* = 8.3, 1.4 Hz, 2H), 8.03 – 7.94 (m, 6H), 7.91 (dd, *J* = 8.4, 1.4 Hz, 2H), 7.86 – 7.83 (m, 2H), 7.62 – 7.55 (m, 2H), 7.55 – 7.49 (m, 2H), 7.48 – 7.36 (m, 12H), 7.31 (td, *J* = 7.9, 2.8 Hz, 4H), 7.28 (d, *J* = 7.8 Hz, 2H), 6.19 (t, *J* = 9.8 Hz, 1H), 6.06 (t, *J* = 10.1 Hz, 1H), 5.96 (dd, *J* = 10.1, 3.4 Hz, 1H), 5.74 (dd, *J* = 3.3, 1.8 Hz, 1H), 5.55 (t, *J* = 9.9 Hz, 1H), 5.31 (d, J = 3.8 Hz, 1H), 5.24 (dd, *J* = 10.2, 3.7 Hz, 1H), 5.13 (d, *J* = 1.7 Hz, 1H), 4.60 (dd, J = 12.1, 2.3 Hz, 1H), 4.55 – 4.48 (m, 1H), 4.39 – 4.32 (m, 2H), 4.07 (dd, *J* = 10.8, 6.2 Hz, 1H), 3.75 (dd, *J* = 10.8, 2.2 Hz, 1H), 3.60 (s, 3H).

Compound 3p(*80*)

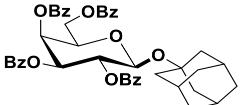

3p

Glycosylation of **1i** (98.0 mg, 0.12 mmol) with **2g** (15.2 mg, 0.10 mmol) according to **General procedure E** afforded **3p***78* (65.7 mg, 89%) as a white solid: ^{1}H NMR (600 MHz, Chloroform-*d*) δ 8.11 (dd, *J* = 8.1, 1.4 Hz, 2H), 8.06 – 8.01 (m, 2H), 7.96 (dd, *J* = 8.2, 1.4 Hz, 2H), 7.79 (dd, *J* = 8.1, 1.4 Hz, 2H), 7.64 – 7.58 (m, 1H), 7.59 – 7.53 (m, 1H), 7.49 (dt, *J* = 20.8, 7.5 Hz, 3H), 7.42 (t,

J = 7.7 Hz, 3H), 7.38 (t, J = 7.8 Hz, 2H), 7.24 (t, J = 7.8 Hz, 2H), 5.96 (d, J = 3.5 Hz, 1H), 5.77 (dd, J = 10.4, 7.9 Hz, 1H), 5.60 (dd, J = 10.4, 3.6 Hz, 1H), 5.09 (d, J = 8.0 Hz, 1H), 4.61 (dd, J = 11.4, 7.7 Hz, 1H), 4.46 (dd, J = 11.4, 5.5 Hz, 1H), 4.35 – 4.30 (m, 1H), 2.04 (s, 3H), 1.88 – 1.81 (m, 3H), 1.69 (dd, J = 11.6, 2.4 Hz, 3H), 1.57 (d, J = 12.7 Hz, 3H), 1.50 (d, J = 12.4 Hz, 3H).

Compound 3q(*75*)

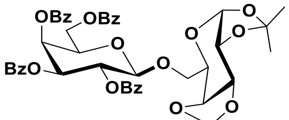

3q

Glycosylation of **1i** (98.0 mg, 0.12 mmol) with **2a** (26.0 mg, 0.10 mmol) according to **General procedure E** afforded **3q** (73.0 mg, 87%) as a white solid: ^{1}H NMR (600 MHz, Chloroform-*d*) δ 8.08 (d, J = 7.7 Hz, 2H), 8.03 (d, J = 7.7 Hz, 2H), 7.97 (d, J = 7.9 Hz, 2H), 7.78 (d, J = 7.8 Hz, 2H), 7.61 (t, J = 7.5 Hz, 1H), 7.56 (t, J = 7.4 Hz, 1H), 7.52 – 7.45 (m, 3H), 7.43 (t, J = 7.7 Hz, 3H), 7.36 (t, J = 7.7 Hz, 2H), 7.25 – 7.21 (m, 2H), 5.99 (d, J = 3.5 Hz, 1H), 5.80 (dd, J = 10.4, 8.0 Hz, 1H), 5.60 (dd, J = 10.4, 3.5 Hz, 1H), 5.41 (d, J = 5.0 Hz, 1H), 5.01 (d, J = 8.0 Hz, 1H), 4.67 (dd, J = 11.3, 6.6 Hz, 1H), 4.46 – 4.38 (m, 2H), 4.34 (t, J = 6.7 Hz, 1H), 4.21 (dd, J = 5.1, 2.4 Hz, 1H), 4.13 – 4.01 (m, 2H), 3.95 – 3.86 (m, 2H), 1.39 (s, 3H), 1.27 – 1.15 (m, 9H). ^{13}C NMR (150 MHz, $CDCl_3$) δ 166.2, 165.72, 165.69, 165.4, 133.7, 133.4, 133.2, 130.2, 130.1, 129.9, 129.6, 129.2, 128.9, 128.7, 128.6, 128.41, 128.35, 109.4, 108.6, 101.9, 96.3, 77.2, 71.9, 71.4, 71.1, 70.7, 70.5, 69.8, 68.6, 68.3, 67.6, 62.2, 26.0, 25.8, 25.0, 24.4.

Compound 3r

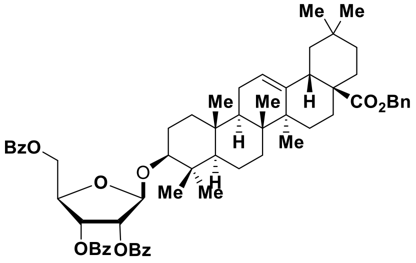

3r

Glycosylation of **1j** (82.0 mg, 0.12 mmol) with **2e** (54.7 mg, 0.10 mmol) according to **General procedure E** afforded **3r** (81.0 mg, 82%) as a white solid: $[\alpha]_D^{20}$= 36.6 (*c* 1.36, $CHCl_3$); ^{1}H NMR (600 MHz, Chloroform-*d*) δ 8.06 – 8.04 (m, 2H), 8.03 – 7.99 (m, 2H), 7.92 – 7.87 (m, 2H), 7.60 – 7.54 (m, 1H), 7.55 – 7.48 (m, 2H), 7.42 (t, J = 7.7 Hz, 2H), 7.41 – 7.27 (m, 9H), 5.83 (dd, J = 6.3, 4.8 Hz, 1H), 5.65 (d, J = 4.8 Hz, 1H), 5.32 (s, 1H), 5.30 – 5.25 (m, 1H), 5.07 (q, J = 12.6 Hz, 2H), 4.73 – 4.65 (m, 2H), 4.57 – 4.49 (m, 1H), 3.14 (dd, J = 11.7, 4.5 Hz, 1H), 2.89 (dd, J = 13.8, 4.5 Hz, 1H), 1.97 (td, J = 13.6, 4.1 Hz, 1H), 1.85 – 1.80 (m, 2H), 1.72 – 1.60 (m, 3H), 1.57 – 1.11 (m, 14H), 1.11 (s, 3H), 1.06 – 1.01 (m, 1H), 0.98 (s, 3H), 0.90 (d, J = 13.8 Hz, 6H), 0.80 (s, 3H), 0.77 (s, 3H), 0.75 – 0.65 (m, 1H), 0.58 (d, J = 11.5 Hz, 3H). ^{13}C NMR (150 MHz, $CDCl_3$) δ 177.6, 166.3, 165.6, 165.5, 143.8, 136.59, 133.55, 133.5, 133.2, 129.94, 129.93, 129.87, 129.5, 129.2, 128.61, 128.55, 128.50, 128.46, 128.1, 128.0, 122.7, 107.9, 89.8, 78.6, 77.2, 76.0, 72.9, 66.1, 65.4, 55.6, 47.7, 46.9, 46.0, 41.8, 41.5, 39.5, 38.9, 38.5, 36.9, 34.0, 33.2, 32.9, 32.5, 30.8, 29.8, 28.3, 27.8, 26.0, 25.7, 23.8, 23.6, 23.2, 18.4, 17.0, 16.6, 15.4. HRMS (ESI) calcd for $C_{63}H_{78}NO_{10}$ $[M+NH_4]^+$ 1008.5620, found 1008.5619.

Compound 3s(*75*)

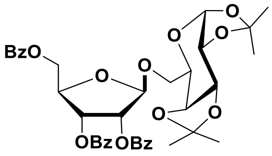

3s

Glycosylation of **1j** (82.0 mg, 0.12 mmol) with **2a** (26.0 mg, 0.10 mmol) according to **General procedure E** afforded **3s** (63.4 mg, 90%) as a white solid: ^{1}H NMR (400 MHz, Chloroform-*d*) δ 8.06 – 7.99 (m, 4H), 7.89 – 7.83 (m, 2H), 7.60 – 7.47 (m, 3H), 7.42 (t, *J* = 7.7 Hz, 2H), 7.36 (t, *J* = 7.7 Hz, 2H), 7.30 (t, *J* = 7.8 Hz, 2H), 5.84 (dd, *J* = 6.8, 4.8 Hz, 1H), 5.74 (d, *J* = 4.8 Hz, 1H), 5.52 (d, *J* = 5.0 Hz, 1H), 5.35 (s, 1H), 4.77 – 4.65 (m, 2H), 4.64 – 4.54 (m, 2H), 4.31 (dd, *J* = 5.0, 2.4 Hz, 1H), 4.19 (dd, *J* = 7.9, 1.5 Hz, 1H), 4.03 – 3.95 (m, 2H), 3.70 – 3.62 (m, 1H), 1.57 (s, 3H), 1.43 (s, 3H), 1.33 (d, *J* = 2.2 Hz, 6H). ^{13}C NMR (100 MHz, $CDCl_3$) δ 166.3, 165.5, 165.3, 133.6, 133.5, 133.2, 130.0, 129.93, 129.87, 129.8, 129.4, 129.1, 128.6, 128.48, 128.46, 109.5, 108.8, 105.7, 96.4, 79.1, 77.2, 75.6, 73.2, 71.0, 70.72, 70.69, 66.9, 66.6, 65.8, 26.3, 26.0, 25.1, 24.5.

Compound 3t(*28*)

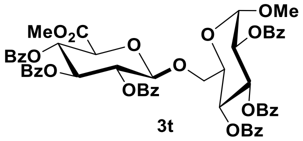

3t

Glycosylation of **1k** (89.0 mg, 0.12 mmol) with **2k** (50.6 mg, 0.10 mmol) according to **General procedure E** afforded **3t** (89.7 mg, 89%) as a white solid: ^{1}H NMR (400 MHz, Chloroform-*d*) δ 8.00 – 7.87 (m, 8H), 7.90 – 7.82 (m, 2H), 7.84 – 7.76 (m, 2H), 7.58 – 7.26 (m, 18H), 6.07 (t, *J* = 9.8 Hz, 1H), 5.92 (t, *J* = 9.4 Hz, 1H), 5.67 (t, *J* = 9.4 Hz, 1H), 5.57 (dd, *J* = 9.4, 7.5 Hz, 1H), 5.33 (t, *J* = 9.9 Hz, 1H), 5.09 (dd, *J* = 10.2, 3.6 Hz, 1H), 4.99 (d, *J* = 7.5 Hz, 1H), 4.91 (d, *J* = 3.5 Hz, 1H), 4.34 (d, *J* = 7.3 Hz, 1H), 4.27 – 4.17 (m, 1H), 4.14 (dd, *J* = 11.5, 1.8 Hz, 1H), 3.78 (dd, *J* = 11.3, 8.0 Hz, 1H), 3.66 (s, 3H), 3.09 (s, 3H).

Compound 3u(*81*)

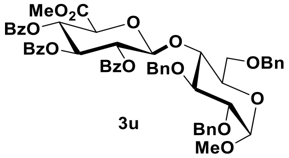

3u

Glycosylation of **1k** (89.0 mg, 0.12 mmol) with **2j** (46.4 mg, 0.10 mmol) according to **General procedure E** afforded **3u** (82.2 mg, 85%) as a white solid: ^{1}H NMR (600 MHz, Chloroform-*d*) δ 7.96 – 7.91 (m, 2H), 7.90 – 7.85 (m, 2H), 7.85 – 7.80 (m, 2H), 7.58 – 7.25 (m, 25H), 5.62 (d, *J* = 9.5 Hz, 1H), 5.57 (t, *J* = 9.5 Hz, 1H), 5.48 (dd, *J* = 9.6, 8.0 Hz, 1H), 5.15 (d, *J* = 11.4 Hz, 1H), 4.82 (d, *J* = 11.2 Hz, 1H), 4.78 (d, *J* = 8.0 Hz, 1H), 4.75 – 4.65 (m, 2H), 4.59 – 4.51 (m, 2H), 4.26 (d, *J* = 12.1 Hz, 1H), 3.98 – 3.88 (m, 3H), 3.69 – 3.61 (m, 2H), 3.52 (s, 3H), 3.51 – 3.48 (m, 1H), 3.40 (dd, *J* = 10.7, 2.1 Hz, 1H), 3.27 (s, 3H).

Compound 3v

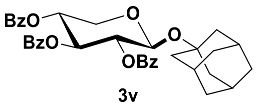

3v

Glycosylation of **1l** (82.0 mg, 0.12 mmol) with **2g** (15.2 mg, 0.10 mmol) according to **General procedure E** afforded **3v** (50.7 mg, 85%) as a white solid: $[\alpha]_D^{20}$= -6.5 (*c* 1.34, $CHCl_3$); ^{1}H NMR (600 MHz, Chloroform-*d*) δ 8.03 – 7.93 (m, 6H), 7.57 – 7.46 (m, 3H), 7.41 – 7.31 (m, 6H), 5.79 (t,

J = 7.9 Hz, 1H), 5.36 – 5.27 (m, 2H), 5.14 (d, *J* = 6.1 Hz, 1H), 4.45 (dd, *J* = 12.0, 4.6 Hz, 1H), 3.65 (dd, *J* = 12.1, 7.9 Hz, 1H), 2.12 (s, 3H), 1.84 (dd, *J* = 11.7, 2.4 Hz, 3H), 1.72 (dd, *J* = 11.8, 2.4 Hz, 3H), 1.64 – 1.58 (m, 3H), 1.59 – 1.53 (m, 3H). ^{13}C NMR (150 MHz, CDCl$_3$) δ 165.7, 165.2, 133.5, 133.4, 133.3, 130.02, 130.0, 129.9, 129.7, 129.40, 129.36, 128.55, 128.46, 93.7, 77.2, 75.6, 71.4, 71.2, 69.7, 61.5, 42.5, 36.2, 30.7. HRMS (ESI) calcd for $C_{36}H_{40}NO_8$ [M+NH$_4$]$^+$ 614.2748, found 614.2744.

Compound 3w

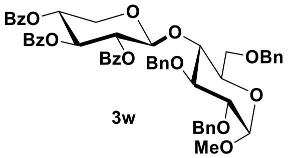

3w

Glycosylation of **1l** (82.0 mg, 0.12 mmol) with **2j** (46.4 mg, 0.10 mmol) according to **General procedure E** afforded **3w** (90.0 mg, 99%) as a white solid: $[\alpha]_D^{25}$= -14.4 (*c* 2.08, CHCl$_3$); ^{1}H NMR (600 MHz, Chloroform-*d*) δ 8.03 – 7.98 (m, 2H), 7.94 – 7.89 (m, 2H), 7.90 – 7.85 (m, 2H), 7.59 – 7.49 (m, 2H), 7.51 – 7.25 (m, 22H), 5.60 (t, *J* = 8.5 Hz, 1H), 5.38 (dd, *J* = 8.7, 6.8 Hz, 1H), 5.27 (td, *J* = 8.6, 5.0 Hz, 1H), 4.95 (d, *J* = 10.4 Hz, 1H), 4.89 (d, *J* = 10.3 Hz, 1H), 4.81 (d, *J* = 12.2 Hz, 1H), 4.71 (d, *J* = 6.7 Hz, 1H), 4.65 (t, *J* = 12.3 Hz, 2H), 4.54 (d, *J* = 3.6 Hz, 1H), 4.33 (d, *J* = 12.0 Hz, 1H), 4.28 (dd, *J* = 12.0, 5.0 Hz, 1H), 3.96 (t, *J* = 9.5 Hz, 1H), 3.88 (t, *J* = 9.3 Hz, 1H), 3.70 (dd, *J* = 10.7, 2.9 Hz, 1H), 3.57 – 3.48 (m, 2H), 3.43 (dd, *J* = 10.7, 1.9 Hz, 1H), 3.30 (s, 3H), 3.26 (dd, *J* = 12.0, 8.8 Hz, 1H). ^{13}C NMR (150 MHz, CDCl$_3$) δ 165.7, 165.6, 165.1, 138.9, 138.4, 138.0, 133.5, 133.44, 133.40, 130.0, 129.92, 129.90, 129.4, 129.31, 129.28, 128.8, 128.6, 128.54, 128.52, 128.46, 128.3, 128.2, 128.0, 127.7, 100.4, 98.6, 80.1, 79.5, 77.2, 76.8, 76.0, 73.8, 73.6, 71.8, 71.6, 69.9, 69.8, 67.8, 62.1, 55.5. HRMS (ESI) calcd for $C_{54}H_{56}NO_{13}$ [M+NH$_4$]$^+$ 926.3746, found 926.3742.

Compound 3x

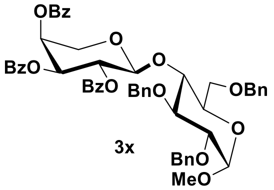

3x

Glycosylation of **1m** (82.0 mg, 0.12 mmol) with **2j** (46.4 mg, 0.10 mmol) according to **General procedure E** afforded **3x** (90.0 mg, 99%) as a white solid: $[\alpha]_D^{20}$= 84.8 (*c* 0.50, CHCl$_3$); ^{1}H NMR (600 MHz, Chloroform-*d*) δ 8.09 (d, *J* = 7.7 Hz, 2H), 7.92 (dd, *J* = 20.5, 7.8 Hz, 4H), 7.56 (q, *J* = 7.0 Hz, 2H), 7.50 – 7.26 (m, 20H), 7.18 – 7.08 (m, 2H), 5.77 (dd, *J* = 9.6, 7.2 Hz, 1H), 5.62 (s, 1H), 5.35 (dd, *J* = 9.6, 3.6 Hz, 1H), 5.02 (d, *J* = 10.0 Hz, 1H), 4.87 (dd, *J* = 11.2, 7.1 Hz, 2H), 4.74 – 4.65 (m, 3H), 4.56 (d, *J* = 3.7 Hz, 1H), 4.34 (d, *J* = 12.0 Hz, 1H), 4.21 (dd, *J* = 13.4, 3.1 Hz, 1H), 4.02 (t, *J* = 9.5 Hz, 1H), 3.92 (t, *J* = 9.3 Hz, 1H), 3.74 (dd, *J* = 10.7, 2.9 Hz, 1H), 3.58 (d, J = 9.8 Hz, 1H), 3.57 – 3.50 (m, 2H), 3.47 (dd, *J* = 10.9, 2.0 Hz, 1H), 3.33 (s, 3H). ^{13}C NMR (150 MHz, CDCl$_3$) δ 165.8, 165.6, 165.1, 138.6, 138.4, 137.9, 133.52, 133.47, 133.4, 129.9, 129.83, 129.79, 129.4, 129.2, 129.0, 128.7, 128.64, 128.57, 128.49, 128.45, 128.4, 128.3, 128.24, 128.19, 127.9, 127.5, 100.6, 98.6, 80.0, 79.1, 77.2, 76.8, 76.1, 73.8, 73.6, 71.4, 70.5, 69.8, 68.9, 67.7, 63.4, 55.5. HRMS (ESI) calcd for $C_{54}H_{52}O_{13}Na$ [M+Na]$^+$ 931.3300, found 931.3305.

Compound 3y

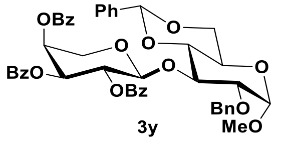

3y

Glycosylation of **1m** (82.0 mg, 0.12 mmol) with **2i** (37.2 mg, 0.10 mmol) according to **General procedure E** afforded **3y** (73.6 mg, 90%) as a white solid: $[\alpha]_D^{25}$= 19.8 (*c* 0.1, $CHCl_3$); ^{1}H NMR (600 MHz, Chloroform-*d*) δ 8.04 (dd, *J* = 8.1, 1.4 Hz, 4H), 7.95 – 7.88 (m, 2H), 7.60 – 7.50 (m, 3H), 7.48 – 7.43 (m, 2H), 7.45 – 7.35 (m, 7H), 7.25 (s, 3H), 7.26 – 7.14 (m, 4H), 5.71 – 5.64 (m, 2H), 5.63 (dt, *J* = 7.2, 3.5 Hz, 1H), 5.57 (s, 1H), 5.32 (d, *J* = 3.4 Hz, 1H), 4.77 (d, *J* = 12.4 Hz, 1H), 4.56 – 4.47 (m, 2H), 4.42 (d, *J* = 3.6 Hz, 1H), 4.35 (t, *J* = 9.4 Hz, 1H), 4.28 (dd, *J* = 10.2, 4.8 Hz, 1H), 3.85 (td, *J* = 10.0, 4.8 Hz, 1H), 3.71 (t, *J* = 10.3 Hz, 1H), 3.64 (dd, *J* = 12.0, 3.8 Hz, 1H), 3.59 (t, *J* = 9.5 Hz, 1H), 3.54 (dd, *J* = 9.3, 3.7 Hz, 1H), 3.33 (s, 3H). ^{13}C NMR (151 MHz, CDCl3) δ 165.5, 165.2, 138.0, 137.2, 133.5, 133.41, 133.37, 130.03, 129.96, 129.9, 129.8, 129.6, 129.3, 129.0, 128.6, 128.54, 128.51, 128.46, 128.3, 128.2, 128.1, 126.0, 101.6, 99.0, 80.4, 80.2, 77.2, 74.0, 70.0, 69.2, 67.1, 62.5, 55.5. HRMS (ESI) calcd for $C_{47}H_{45}O_{13}$ $[M+H]^+$ 817.2855, found 817.2859.

Compound 3z(*78*)

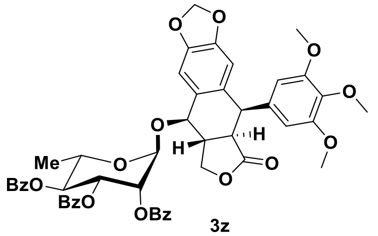

3z

Glycosylation of **1n** (83.4 mg, 0.12 mmol) with **2f** (41.4 mg, 0.10 mmol) according to **General procedure E** afforded **3z** (65.4 mg, 75%) as a white solid: ^{1}H NMR (400 MHz, Chloroform-*d*) δ 8.10 – 8.02 (m, 2H), 8.04 – 7.96 (m, 2H), 7.89 – 7.81 (m, 2H), 7.67 – 7.58 (m, 1H), 7.59 – 7.36 (m, 6H), 7.29 (d, *J* = 7.7 Hz, 2H), 7.14 (s, 1H), 6.56 (s, 1H), 6.39 (s, 2H), 6.04 (dd, *J* = 13.6, 1.4 Hz, 2H), 5.91 (dd, *J* = 10.2, 3.3 Hz, 1H), 5.74 (t, *J* = 10.0 Hz, 1H), 5.57 (dd, *J* = 3.3, 1.8 Hz, 1H), 5.22 – 5.11 (m, 3H), 5.09 (d, *J* = 9.8 Hz, 1H), 4.67 (dd, *J* = 8.7, 7.2 Hz, 1H), 4.61 (d, *J* = 4.6 Hz, 1H), 4.36 (dt, *J* = 9.5, 6.2 Hz, 1H), 4.20 (dd, *J* = 10.4, 8.8 Hz, 1H), 3.80 (s, 3H), 3.69 (s, 5H), 3.30 – 3.12 (m, 1H), 2.86 (dd, *J* = 14.4, 4.6 Hz, 1H), 1.39 (d, *J* = 6.2 Hz, 3H).

Compound 3aa(*28*)

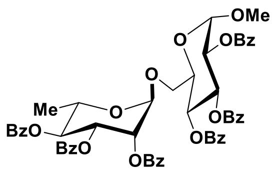

3aa

Glycosylation of **1n** (84.0 mg, 0.12 mmol) with **2k** (50.6 mg, 0.10 mmol) according to **General procedure E** afforded **3aa** (94.4 mg, 97%) as a white solid: ^{1}H NMR (600 MHz, Chloroform-*d*) δ 8.09 (dd, *J* = 8.0, 1.4 Hz, 2H), 8.03 – 7.99 (m, 4H), 7.98 – 7.95 (m, 2H), 7.92 – 7.87 (m, 2H), 7.81 (dd, *J* = 8.1, 1.4 Hz, 2H), 7.63 – 7.56 (m, 1H), 7.54 – 7.44 (m, 5H), 7.45 – 7.35 (m, 8H), 7.29 (t, *J* = 7.8 Hz, 2H), 7.28 – 7.22 (m, 3H), 6.22 (t, *J* = 9.4 Hz, 1H), 5.81 (dd, *J* = 10.1, 3.4 Hz, 1H), 5.73 (dd, *J* = 3.5, 1.8 Hz, 1H), 5.66 (t, *J* = 9.9 Hz, 1H), 5.53 (t, *J* = 9.9 Hz, 1H), 5.34 – 5.28 (m, 2H), 5.17 (d, *J* = 1.7 Hz, 1H), 4.43 – 4.36 (m, 1H), 4.21 – 4.13 (m, 1H), 3.95 (dd, *J* = 11.9, 2.2 Hz, 1H), 3.85 (dd, *J* = 11.8, 7.1 Hz, 1H), 3.60 (s, 3H), 1.29 (d, *J* = 6.2 Hz, 3H). ^{13}C NMR (150 MHz, $CDCl_3$)

δ 165.93, 165.89, 165.6, 165.5, 133.6, 133.54, 133.48, 133.4, 133.23, 133.17, 130.1, 130.0, 129.9, 129.8, 129.8, 129.5, 129.4, 129.34, 129.31, 129.2, 128.9, 128.7, 128.6, 128.5, 128.40, 128.36, 98.4, 97.0, 77.2, 72.2, 71.9, 70.7, 70.5, 69.94, 69.86, 69.7, 67.0, 66.9, 56.0, 17.7.

Compound 3ab

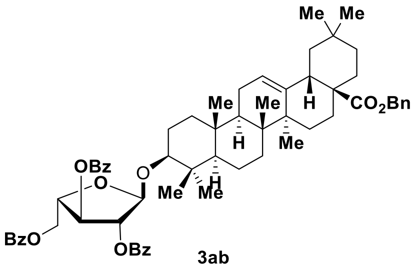

3ab

Glycosylation of **1o** (82.0 mg, 0.12 mmol) with **2e** (54.7 mg, 0.10 mmol) according to **General procedure E** afforded **3ab** (69.4 mg, 70%) as a white solid: $[\alpha]_D^{25}$= 18.5 (*c* 1.99, $CHCl_3$); ^1H NMR (600 MHz, Chloroform-*d*) δ 8.08 (dd, *J* = 8.3, 1.4 Hz, 2H), 8.07 – 8.02 (m, 2H), 8.00 (dd, *J* = 8.3, 1.4 Hz, 2H), 7.58 (dt, *J* = 13.6, 7.4 Hz, 2H), 7.51 (t, *J* = 7.5 Hz, 1H), 7.45 (t, *J* = 7.8 Hz, 2H), 7.39 (t, *J* = 7.8 Hz, 2H), 7.37 – 7.28 (m, 7H), 5.57 (dd, *J* = 4.8, 1.4 Hz, 1H), 5.50 (d, *J* = 1.3 Hz, 1H), 5.34 (s, 1H), 5.31 – 5.26 (m, 1H), 5.12 – 5.01 (m, 2H), 4.79 (dd, *J* = 11.7, 3.6 Hz, 1H), 4.67 (dd, *J* = 11.8, 5.1 Hz, 1H), 4.62 (td, *J* = 5.0, 3.6 Hz, 1H), 3.21 (dt, *J* = 10.6, 5.2 Hz, 1H), 2.90 (dd, *J* = 13.8, 4.5 Hz, 1H), 1.98 (td, *J* = 13.6, 4.1 Hz, 1H), 1.89 – 1.82 (m, 3H), 1.75 – 1.59 (m, 7H), 1.56 – 1.48 (m, 3H), 1.47 – 1.29 (m, 4H), 1.29 – 1.12 (m, 4H), 1.12 (s, 3H), 1.03 (s, 3H), 0.94 – 0.88 (m, 9H), 0.87 (s, 3H), 0.61 (s, 3H). ^{13}C NMR (150 MHz, $CDCl_3$) δ 177.6, 166.4, 165.9, 165.7, 143.8, 136.64, 133.59, 133.6, 133.2, 130.1, 130.0, 129.9, 129.4, 129.3, 128.64, 128.61, 128.55, 128.5, 128.1, 128.0, 122.6, 107.7, 87.9, 82.3, 80.9, 77.9, 77.2, 66.1, 64.1, 55.6, 47.7, 46.9, 46.0, 41.8, 41.5, 39.4, 39.0, 38.5, 36.9, 34.0, 33.2, 32.8, 32.5, 30.8, 28.3, 27.8, 26.0, 25.5, 23.8, 23.6, 23.2, 18.4, 17.0, 16.5, 15.5. HRMS (ESI) calcd for $C_{63}H_{74}O_{10}Na$ $[M+Na]^+$ 1013.5174, found 1013.5178.

Compound 3ac(*82*)

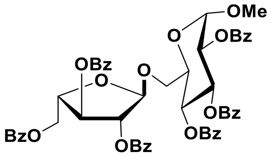

3ac

Glycosylation of **1o** (82.0 mg, 0.12 mmol) with **2k** (50.6 mg, 0.10 mmol) according to **General procedure E** afforded **3ac** (94.0 mg, 99%) as a white solid: ^1H NMR (400 MHz, Chloroform-*d*) δ 8.09 – 8.00 (m, 2H), 7.98 – 7.82 (m, 8H), 7.83 – 7.75 (m, 2H), 7.57 – 7.46 (m, 2H), 7.48 – 7.37 (m, 5H), 7.39 – 7.25 (m, 6H), 7.27 – 7.15 (m, 5H), 6.15 – 6.04 (m, 1H), 5.58 – 5.47 (m, 3H), 5.32 (s, 1H), 5.21 – 5.12 (m, 2H), 4.72 (dd, *J* = 11.8, 3.4 Hz, 1H), 4.64 – 4.49 (m, 2H), 4.24 (ddd, *J* = 10.0, 6.3, 2.1 Hz, 1H), 3.92 (dd, *J* = 11.6, 2.2 Hz, 1H), 3.78 (dd, *J* = 11.6, 6.3 Hz, 1H), 3.39 (s, 3H). ^{13}C NMR (100 MHz, $CDCl_3$) δ 166.3, 165.9, 165.9, 165.43, 165.37, 133.7, 133.6, 133.49, 133.46, 133.2, 133.1, 130.1, 130.04, 130.0, 129.97, 129.9, 129.83, 129.79, 129.4, 129.3, 129.2, 129.1, 128.64, 128.61, 128.5, 128.41, 128.38, 106.4, 97.0, 82.0, 81.5, 78.0, 77.2, 72.3, 70.6, 69.7, 69.3, 66.4, 63.9, 55.7.

Compound 3ad(*83*)

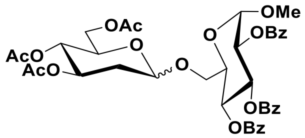

3ad (77%, α: β=4:1)

Glycosylation of **1p** (61.0 mg, 0.12 mmol) with **2k** (50.6 mg, 0.10 mmol) according to **General procedure E** afforded **3ad** (60.0 mg, 77%, α:β=4:1) as a white solid.

Compound 3ae(*84*)

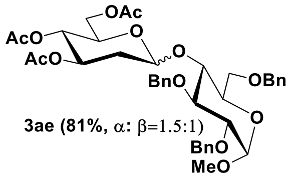

3ae (81%, α: β=1.5:1)

Glycosylation of **1p** (61.0 mg, 0.12 mmol) with **2j** (50.6 mg, 0.10 mmol) according to **General procedure E** afforded **3ae** (59.7 mg, 81%, α:β=1.5:1) as a white solid.

Compound 3af

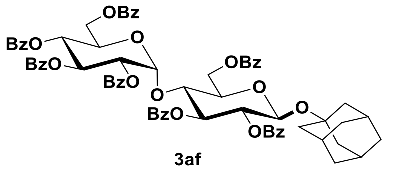

3af

Glycosylation of **1q** (78.0 mg, 0.06 mmol) with **2g** (7.6 mg, 0.05 mmol) according to **General procedure E** afforded **3af** (54.6 mg, 91%) as a white solid: $[\alpha]^{20}_{D}$= 50.9 (*c* 1.11, $CHCl_3$); ^{1}H NMR (400 MHz, Chloroform-*d*) δ 8.10 (d, *J* = 7.7 Hz, 2H), 8.03 (d, *J* = 7.8 Hz, 2H), 7.86 (dd, *J* = 19.0, 7.8 Hz, 4H), 7.73 (d, *J* = 7.8 Hz, 4H), 7.65 – 7.38 (m, 12H), 7.38 – 7.27 (m, 6H), 7.25 – 7.16 (m, 5H), 6.08 (t, *J* = 10.0 Hz, 1H), 5.85 – 5.61 (m, 3H), 5.35 – 5.22 (m, 2H), 5.06 (d, J = 7.8 Hz, 1H), 4.93 (dd, *J* = 11.8, 2.6 Hz, 1H), 4.66 (dd, *J* = 11.7, 6.2 Hz, 1H), 4.56 – 4.44 (m, 2H), 4.44 – 4.32 (m, 2H), 4.17 – 4.06 (m, 1H), 2.01 (s, 3H), 1.78 (d, *J* = 11.7 Hz, 3H), 1.68 – 1.42 (m, 9H). ^{13}C NMR (150 MHz, $CDCl_3$) δ 166.2, 166.1, 165.7, 165.5, 165.20, 165.16, 133.5, 133.4, 133.3, 133.22, 133.17, 133.0, 130.1, 130.0, 129.89, 129.87, 129.82, 129.76, 129.70, 129.68, 129.60, 129.57, 129.0, 128.9, 128.8, 128.6, 128.54, 128.48, 128.33, 128.28, 128.2, 96.7, 93.9, 77.2, 75.8, 75.5, 74.3, 72.6, 72.5, 70.9, 70.0, 69.3, 69.2, 64.1, 62.7, 42.4, 36.1, 30.6. HRMS (ESI) calcd for $C_{71}H_{64}O_{18}Na$ $[M+Na]^+$ 1227.3985, found 1227.3980.

Compound 3ag(*78*)

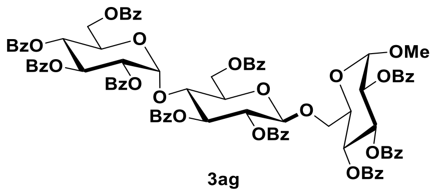

3ag

Glycosylation of **1q** (78.0 mg, 0.06 mmol) with **2k** (25.3 mg, 0.05 mmol) according to **General procedure E** afforded **3ag*86*** (75.6 mg, 97%) as a white solid: $[\alpha]^{20}_{D}$= 61.0 (*c* 2.69, $CHCl_3$); ^{1}H NMR (400 MHz, Chloroform-*d*) δ 8.11 – 8.04 (m, 2H), 8.02 – 7.95 (m, 2H), 7.96 – 7.88 (m, 2H), 7.90 – 7.81 (m, 6H), 7.81 – 7.70 (m, 6H), 7.68 – 7.60 (m, 2H), 7.61 – 7.15 (m, 30H), 6.06 (q, *J* = 10.3 Hz, 2H), 5.79 (t, *J* = 9.2 Hz, 1H), 5.72 (d, *J* = 3.9 Hz, 1H), 5.65 (t, *J* = 9.8 Hz, 1H), 5.41 – 5.20 (m, 3H), 5.09 (dd, *J* = 10.2, 3.6 Hz, 1H), 4.96 – 4.83 (m, 3H), 4.71 (dd, *J* = 12.1, 4.2 Hz, 1H), 4.53 – 4.33 (m, 3H), 4.29 – 4.13 (m, 2H), 4.15 – 4.02 (m, 2H), 3.76 (dd, *J* = 11.5, 7.6 Hz, 1H), 3.04 (s, 3H). ^{13}C NMR (150 MHz, $CDCl_3$) δ 166.2, 165.9, 165.80, 165.76, 165.7, 165.6, 165.5, 165.4, 165.1,

133.6, 133.54, 133.49, 133.42, 133.39, 133.3, 133.18, 133.15, 130.1, 130.0, 129.9, 129.8, 129.72, 129.68, 129.6, 129.4, 129.29, 129.27, 129.1, 129.0, 128.83, 128.80, 128.77, 128.7, 128.6, 128.52, 128.49, 128.33, 128.30, 128.2, 101.4, 96.49, 96.47, 77.2, 74.9, 73.1, 73.0, 72.4, 72.1, 71.0, 70.4, 70.0, 69.7, 69.2, 69.1, 68.9, 68.8, 63.5, 62.6, 55.1.

Compound 6a(*38*)

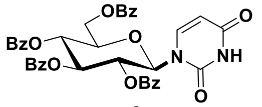

6a

Glycosylation of **1a** (81.5 mg, 0.10 mmol) with **5a** (22.4 mg, 0.2 mmol) according to **General procedure F** afforded **6a** (68.3 mg, 99%) as a white solid: ^{1}H NMR (600 MHz, Chloroform-*d*) δ 9.26 (d, *J* = 2.3 Hz, 1H), 8.06 – 8.01 (m, 2H), 7.95 – 7.88 (m, 2H), 7.91 – 7.84 (m, 2H), 7.82 – 7.77 (m, 2H), 7.60 – 7.48 (m, 4H), 7.46 – 7.41 (m, 3H), 7.39 – 7.33 (m, 4H), 7.30 – 7.26 (m, 3H), 6.29 (d, *J* = 9.4 Hz, 1H), 6.10 (t, *J* = 9.6 Hz, 1H), 5.84 (dd, *J* = 8.2, 2.2 Hz, 1H), 5.79 (t, *J* = 9.8 Hz, 1H), 5.69 (t, *J* = 9.5 Hz, 1H), 4.66 (dd, *J* = 12.4, 2.6 Hz, 1H), 4.50 (dd, *J* = 12.4, 5.0 Hz, 1H), 4.47 – 4.41 (m, 1H). ^{13}C NMR (150 MHz, $CDCl_3$) δ 166.2, 165.6, 165.4, 165.3, 162.1, 150.1, 139.2, 134.1, 133.9, 133.6, 133.5, 130.1, 130.0, 129.9, 129.9, 129.5, 128.7, 128.7, 128.6, 128.6, 128.5, 128.0, 104.0, 80.9, 77.2, 75.6, 73.0, 70.3, 68.9, 62.7.

Compound 6b(*85*)

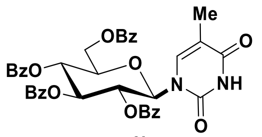

6b

Glycosylation of **1a** (81.5 mg, 0.10 mmol) with **5b** (25.2 mg, 0.2 mmol) according to **General procedure F** afforded **6b** (64.0 mg, 91%) as a white solid: ^{1}H NMR (600 MHz, Chloroform-*d*) δ 8.74 (s, 1H), 8.04 (dd, *J* = 8.3, 1.4 Hz, 2H), 7.92 (dd, *J* = 8.4, 1.4 Hz, 2H), 7.86 (dd, *J* = 8.4, 1.4 Hz, 2H), 7.80 (dd, *J* = 8.4, 1.3 Hz, 2H), 7.61 – 7.54 (m, 1H), 7.54 – 7.46 (m, 2H), 7.46 – 7.40 (m, 3H), 7.39 – 7.31 (m, 4H), 7.32 – 7.26 (m, 3H), 6.27 (d, *J* = 9.4 Hz, 1H), 6.08 (t, *J* = 9.6 Hz, 1H), 5.78 (t, *J* = 9.8 Hz, 1H), 5.68 (t, *J* = 9.5 Hz, 1H), 4.67 (dd, *J* = 12.5, 2.7 Hz, 1H), 4.49 (dd, *J* = 12.4, 5.1 Hz, 1H), 4.41 (ddd, *J* = 10.0, 5.1, 2.7 Hz, 1H), 1.93 (d, *J* = 1.3 Hz, 3H).

Compound 6c(*85*)

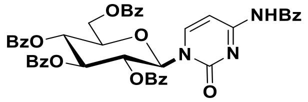

6c

Glycosylation of **1a** (81.5 mg, 0.10 mmol) with **5c** (43.0 mg, 0.2 mmol) according to **General procedure F** afforded **6c** (73.0 mg, 92%) as a white solid: ^{1}H NMR (600 MHz, Chloroform-*d*) δ 8.63 (s, 1H), 8.04 (d, *J* = 7.5 Hz, 2H), 7.93 (d, *J* = 7.9 Hz, 2H), 7.88 (d, *J* = 7.9 Hz, 2H), 7.81 (d, *J* = 7.9 Hz, 2H), 7.62 – 7.55 (m, 2H), 7.54 – 7.40 (m, 8H), 7.35 (dt, *J* = 24.3, 7.6 Hz, 4H), 7.28 (d, *J* = 7.6 Hz, 2H), 6.53 (d, *J* = 9.3 Hz, 1H), 6.10 (t, *J* = 9.4 Hz, 1H), 5.80 (t, *J* = 9.7 Hz, 1H), 5.70 (t, *J* = 9.4 Hz, 1H), 4.67 (dd, *J* = 12.4, 2.6 Hz, 1H), 4.52 (dd, *J* = 12.4, 5.0 Hz, 1H), 4.42 (ddd, *J* = 10.3, 5.1, 2.7 Hz, 1H).

Compound 6d(*17*)

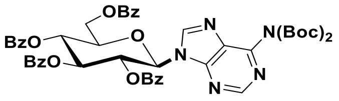

6d

Glycosylation of **1a** (98.0 mg, 0.12 mmol) with **5d** (33.6 mg, 0.1 mmol) according to **General procedure F** afforded **6d** (58.5 mg, 64%) as a white solid: ^{1}H NMR (600 MHz, Chloroform-*d*) δ 8.78 (s, 1H), 8.41 (s, 1H), 8.03 – 7.99 (m, 2H), 7.96 – 7.92 (m, 2H), 7.84 – 7.80 (m, 2H), 7.71 – 7.66 (m, 2H), 7.58 – 7.50 (m, 2H), 7.46 – 7.35 (m, 6H), 7.28 (t, *J* = 7.9 Hz, 2H), 7.27 – 7.22 (m, 2H), 6.34 (d, *J* = 8.8 Hz, 1H), 6.20 – 6.11 (m, 2H), 5.92 (t, *J* = 9.4 Hz, 1H), 4.74 – 4.67 (m, 1H), 4.56 – 4.48 (m, 2H), 1.32 (s, 18H). ^{13}C NMR (151 MHz, $CDCl_3$) δ 166.2, 165.7, 165.3, 164.9, 153.3, 152.6, 150.8, 150.2, 142.2, 133.9, 133.8, 133.6, 133.4, 130.1, 129.92, 129.89, 129.5, 128.7, 128.6, 128.52, 128.47, 127.7, 83.9, 81.2, 77.2, 75.8, 73.2, 71.2, 69.0, 62.6, 27.8.

Compound 6e(*19*)

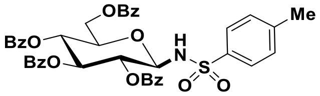

6e

Glycosylation of **1a** (98.0 mg, 0.12 mmol) with **5e** (17.1 mg, 0.1 mmol) according to **General procedure F** afforded **6e** (65.2 mg, 87%) as a white solid: ^{1}H NMR (600 MHz, Chloroform-*d*) δ 8.03 – 7.98 (m, 2H), 7.90 – 7.86 (m, 2H), 7.87 – 7.82 (m, 2H), 7.82 – 7.77 (m, 2H), 7.67 (d, *J* = 8.4 Hz, 2H), 7.58 – 7.46 (m, 4H), 7.44 – 7.32 (m, 8H), 6.93 (d, *J* = 8.1 Hz, 2H), 5.98 (t, *J* = 9.7 Hz, 1H), 5.62 (t, *J* = 9.8 Hz, 1H), 5.35 (t, *J* = 9.5 Hz, 1H), 5.16 (d, *J* = 9.1 Hz, 1H), 4.45 (dd, *J* = 12.2, 3.0 Hz, 1H), 4.36 (dd, *J* = 12.3, 5.0 Hz, 1H), 4.19 (ddd, *J* = 10.1, 4.9, 3.0 Hz, 1H), 2.17 (s, 3H).

Compound 6f(*85*)

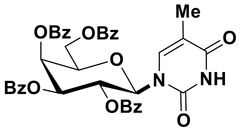

6f

Glycosylation of **1i** (81.5 mg, 0.10 mmol) with **5b** (25.2 mg, 0.2 mmol) according to **General procedure F** afforded **6f** (69.7 mg, 99%) as a white solid: ^{1}H NMR (600 MHz, Chloroform-*d*) δ 8.06 (dd, *J* = 8.3, 1.4 Hz, 2H), 8.00 (dd, *J* = 8.3, 1.4 Hz, 2H), 7.88 (dd, *J* = 8.4, 1.4 Hz, 2H), 7.79 (dd, *J* = 8.3, 1.3 Hz, 2H), 7.71 – 7.65 (m, 1H), 7.58 – 7.51 (m, 3H), 7.52 – 7.46 (m, 1H), 7.48 – 7.39 (m, 3H), 7.38 (d, *J* = 1.4 Hz, 1H), 7.37 – 7.31 (m, 2H), 7.29 – 7.23 (m, 2H), 6.25 (d, *J* = 9.3 Hz, 1H), 6.10 (dd, *J* = 3.3, 1.0 Hz, 1H), 5.93 (t, *J* = 9.7 Hz, 1H), 5.82 (dd, *J* = 10.1, 3.4 Hz, 1H), 4.67 – 4.55 (m, 2H), 4.47 (dd, *J* = 10.9, 5.1 Hz, 1H), 2.03 (d, *J* = 1.3 Hz, 3H).

Compound 6g(*17*)

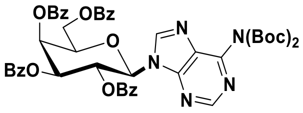

6g

Glycosylation of **1i** (98.0 mg, 0.12 mmol) with **5d** (33.6 mg, 0.1 mmol) according to **General procedure F** afforded **6g** (37.4 mg, 41%) as a white solid: ^{1}H NMR (600 MHz, Chloroform-*d*) δ 8.84 (s, 1H), 8.48 (s, 1H), 8.20 – 8.14 (m, 2H), 7.99 (dd, *J* = 8.3, 1.4 Hz, 2H), 7.80 – 7.77 (m, 2H), 7.71 – 7.67 (m, 3H), 7.60 – 7.53 (m, 3H), 7.47 – 7.37 (m, 5H), 7.28 – 7.23 (m, 3H), 6.47 (t, *J* = 9.7 Hz, 1H), 6.29 (d, *J* = 9.4 Hz, 1H), 6.18 (d, *J* = 3.2 Hz, 1H), 5.88 (dd, *J* = 10.1, 3.3 Hz, 1H), 4.70 – 4.62 (m, 2H), 4.51 (dd, *J* = 11.3, 5.9 Hz, 1H), 1.34 (s, 18H). ^{13}C NMR (150 MHz, $CDCl_3$) δ 166.1,

165.5, 165.4, 165.0, 153.3, 152.6, 150.8, 150.2, 142.5, 134.1, 133.9, 133.7, 133.6, 130.1, 130.0, 129.9, 129.8, 129.2, 129.02, 128.97, 128.8, 128.60, 128.57, 128.52, 128.49, 127.8, 83.9, 81.7, 77.2, 74.7, 72.1, 68.9, 68.1, 62.0, 27.8.

Compound 6h

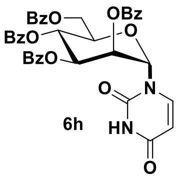

6h

Glycosylation of **1h** (81.5 mg, 0.10 mmol) with **5a** (22.4 mg, 0.20 mmol) according to **General procedure F** afforded **6h** (47.6 mg, 69%) as a white solid: $[\alpha]_D^{20}$= 82.3 (*c* 0.62, $CHCl_3$); ^{1}H NMR (600 MHz, Chloroform-*d*) δ 8.45 (d, *J* = 2.3 Hz, 1H), 8.17 – 8.12 (m, 2H), 8.12 – 8.07 (m, 2H), 8.05 – 8.00 (m, 2H), 7.88 – 7.82 (m, 2H), 7.69 – 7.62 (m, 2H), 7.59 – 7.48 (m, 7H), 7.42 (t, *J* = 7.7 Hz, 2H), 7.34 (t, *J* = 7.7 Hz, 2H), 6.62 (d, *J* = 9.3 Hz, 1H), 6.09 (t, *J* = 3.6 Hz, 1H), 5.91 (dd, *J* = 9.3, 3.4 Hz, 1H), 5.79 (dd, *J* = 8.2, 2.3 Hz, 1H), 5.61 (dd, *J* = 3.8, 1.9 Hz, 1H), 5.16 (dd, *J* = 12.0, 8.5 Hz, 1H), 4.86 – 4.80 (m, 1H), 4.69 (dd, *J* = 12.0, 5.1 Hz, 1H). ^{13}C NMR (150 MHz, $CDCl_3$) δ 166.2, 165.2, 165.1, 165.0, 162.4, 150.1, 140.0, 134.21, 134.18, 134.0, 133.5, 130.1, 130.03, 130.02, 129.9, 129.4, 129.1, 129.0, 128.80, 128.76, 128.6, 128.3, 103.6, 77.2, 76.4, 76.3, 69.3, 68.9, 67.2, 60.8. HRMS (ESI) calcd for $C_{38}H_{31}N_2O_{11}$ $[M+H]^+$ 691.1922, found 691.1926.

Compound 6i

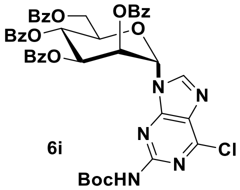

6i

Glycosylation of **1h** (98.0 mg, 0.12 mmol) with **5d** (27.0 mg, 0.10 mmol) according to **General procedure F** afforded **6i** (34.8 mg, 41%) as a white solid: $[\alpha]_D^{20}$= 21.0 (*c* 1.71, $CHCl_3$); ^{1}H NMR (600 MHz, Chloroform-*d*) δ 8.18 (s, 1H), 8.04 (ddd, *J* = 15.3, 8.3, 1.4 Hz, 5H), 8.00 – 7.94 (m, 6H), 7.62 – 7.52 (m, 5H), 7.44 – 7.36 (m, 8H), 6.85 (dd, *J* = 4.6, 3.4 Hz, 1H), 6.39 (dd, *J* = 7.1, 3.4 Hz, 1H), 6.33 (d, *J* = 4.6 Hz, 1H), 6.00 (t, *J* = 7.4 Hz, 1H), 4.84 – 4.75 (m, 2H), 4.66 (dd, *J* = 11.7, 2.2 Hz, 1H), 1.50 (s, 9H). ^{13}C NMR (150 MHz, $CDCl_3$) δ 166.2, 165.3, 165.2, 165.1, 152.8, 152.31, 152.28, 149.8, 143.3, 133.9, 133.8, 133.7, 133.4, 130.03, 129.95, 129.9, 129.8, 129.7, 129.4, 128.8, 128.7, 128.62, 128.60, 128.55, 128.5, 128.4, 128.1, 81.8, 81.5, 73.3, 70.2, 68.4, 67.7, 62.0, 28.1. HRMS (ESI) calcd for $C_{44}H_{39}N_5O_{11}Cl$ $[M+H]^+$ 848.2329, found 848.2333.

Compound 6j

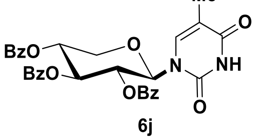

6j

Glycosylation of **1l** (68.1 mg, 0.10 mmol) with **5b** (25.2 mg, 0.20 mmol) according to **General procedure F** afforded **6j** (55.9 mg, 98%) as a white solid: $[\alpha]_D^{20}$= -0.73 (*c* 0.55, $CHCl_3$); ^{1}H NMR (600 MHz, Chloroform-*d*) δ 7.96 (dd, *J* = 8.4, 1.4 Hz, 2H), 7.90 – 7.85 (m, 4H), 7.58 – 7.51 (m, 1H), 7.53 – 7.47 (m, 1H), 7.49 – 7.43 (m, 1H), 7.41 (t, *J* = 7.9 Hz, 2H), 7.38 – 7.28 (m, 5H), 6.10 (d, *J* = 9.4 Hz, 1H), 6.05 (t, *J* = 9.6 Hz, 1H), 5.63 (t, *J* = 9.5 Hz, 1H), 5.52 – 5.44 (m, 1H), 4.51 (dd,

J = 11.6, 5.6 Hz, 1H), 3.78 (dd, J = 11.6, 10.6 Hz, 1H), 1.98 (d, J = 1.3 Hz, 3H). ^{13}C NMR (150 MHz, $CDCl_3$) δ 165.6, 165.5, 165.3, 163.0, 150.2, 134.7, 133.8, 133.7, 133.5, 130.0, 129.9, 129.7, 128.7, 128.558, 128.55, 128.4, 128.0, 112.1, 81.3, 72.6, 70.0, 69.6, 65.9, 12.6. HRMS (ESI) calcd for $C_{31}H_{27}N_2O_9$ $[M+H]^+$ 571.1711, found 571.1712.

Compound 6k(*17*)

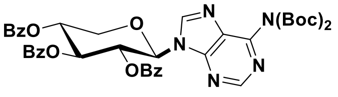

6k

Glycosylation of **1l** (82.0 mg, 0.12 mmol) with **5d** (33.6 mg, 0.10 mmol) according to **General procedure E** afforded **6k** (44.4 mg, 57%) as a white solid: ^1H NMR (600 MHz, Chloroform-*d*) δ 8.80 (s, 1H), 8.41 (s, 1H), 7.99 (dd, J = 8.4, 1.3 Hz, 2H), 7.89 (dd, J = 8.4, 1.4 Hz, 2H), 7.71 – 7.66 (m, 2H), 7.59 – 7.52 (m, 1H), 7.49 – 7.43 (m, 1H), 7.45 – 7.39 (m, 3H), 7.35 – 7.29 (m, 2H), 7.27 – 7.21 (m, 2H), 6.19 (d, J = 8.9 Hz, 1H), 6.17 – 6.07 (m, 2H), 5.64 (ddd, J = 10.4, 9.3, 5.7 Hz, 1H), 4.63 (dd, J = 11.6, 5.7 Hz, 1H), 3.90 (dd, J = 11.7, 10.4 Hz, 1H), 1.32 (s, 18H).

Compound 6l

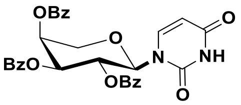

6l

Glycosylation of **1m** (68.1 mg, 0.10 mmol) with **5a** (22.4 mg, 0.20 mmol) according to **General procedure F** afforded **6l** (55.0 mg, 99%) as a white solid: $[\alpha]_D^{20}$= 173.6 (*c* 0.33, $CHCl_3$); ^1H NMR (600 MHz, Chloroform-*d*) δ 8.17 (s, 1H), 8.12 – 8.07 (m, 2H), 7.93 – 7.87 (m, 2H), 7.86 – 7.81 (m, 2H), 7.68 – 7.62 (m, 1H), 7.56 – 7.49 (m, 4H), 7.48 – 7.44 (m, 1H), 7.36 (dd, J = 8.3, 7.4 Hz, 2H), 7.31 – 7.27 (m, 2H), 6.07 (d, J = 9.4 Hz, 1H), 5.93 (t, J = 9.6 Hz, 1H), 5.84 (d, J = 8.2 Hz, 1H), 5.80 – 5.77 (m, 1H), 5.75 (dd, J = 10.0, 3.5 Hz, 1H), 4.42 (dd, J = 13.5, 2.0 Hz, 1H), 4.12 (dd, J = 13.6, 1.2 Hz, 1H). ^{13}C NMR (150 MHz, $CDCl_3$) δ 165.54, 165.49, 165.3, 161.9, 150.0, 139.2, 133.8, 133.7, 133.5, 129.9, 129.8, 129.3, 128.8, 128.64, 128.58, 128.4, 128.1, 103.7, 81.5, 71.5, 69.1, 68.2, 67.5. HRMS (ESI) calcd for $C_{30}H_{25}N_2O_9$ $[M+H]^+$ 557.1555, found 557.1552.

Compound 6m(*17*)

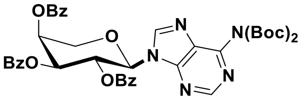

6m

Glycosylation of **1m** (82.0 mg, 0.12 mmol) with **5d** (33.6 mg, 0.10 mmol) according to **General procedure E** afforded **6m** (52.2 mg, 67%) as a white solid: ^1H NMR (600 MHz, Chloroform-*d*) δ 8.83 (s, 1H), 8.43 (s, 1H), 8.20 (d, J = 6.9 Hz, 2H), 7.85 (d, J = 6.9 Hz, 2H), 7.71 (d, J = 6.9 Hz, 2H), 7.68 (t, J = 7.4 Hz, 1H), 7.56 (t, J = 7.8 Hz, 2H), 7.46 (t, J = 7.4 Hz, 1H), 7.43 (t, J = 7.4 Hz, 1H), 7.29 (t, J = 7.8 Hz, 2H), 7.27 – 7.24 (m, 2H), 6.48 (t, J = 9.7 Hz, 1H), 6.16 (d, J = 9.4 Hz, 1H), 5.90 – 5.89 (m, 1H), 5.83 (dd, J = 10.0, 3.5 Hz, 1H), 4.52 (dd, J = 13.6, 2.1 Hz, 1H), 4.25 (d, J = 13.4 Hz, 1H), 1.31 (s, 18H). ^{13}C NMR (150 MHz, $CDCl_3$) δ 165.7, 165.6, 165.1, 153.2, 152.6, 150.8, 150.0, 142.5, 133.94, 133.88, 133.7, 130.1, 129.9, 129.8, 129.3, 128.9, 128.8, 128.64, 128.57, 128.5, 127.9, 127.8, 83.8, 82.1, 77.2, 71.9, 69.1, 69.0, 67.7, 27.7.

Compound 6n

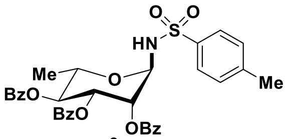

6n

Glycosylation of **1n** (84.0 mg, 0.12 mmol) with **5e** (17.1 mg, 0.10 mmol) according to **General procedure E** afforded **6n** (44.7 mg, 71%) as a white solid: $[\alpha]_D^{25}$= 106.9 (*c* 0.3, $CHCl_3$); ^{1}H NMR (600 MHz, Chloroform-*d*) δ 8.01 (d, *J* = 7.6 Hz, 2H), 7.92 (d, *J* = 7.8 Hz, 2H), 7.89 (d, *J* = 8.1 Hz, 2H), 7.79 (d, *J* = 7.7 Hz, 2H), 7.59 (t, *J* = 7.5 Hz, 1H), 7.51 – 7.39 (m, 4H), 7.34 (t, *J* = 7.7 Hz, 2H), 7.29 (d, *J* = 8.1 Hz, 2H), 7.24 (t, *J* = 7.7 Hz, 2H), 6.86 – 6.78 (m, 1H), 5.75 – 5.68 (m, 2H), 5.56 – 5.47 (m, 2H), 3.95 – 3.87 (m, 1H), 2.40 (s, 3H), 1.05 (d, *J* = 6.2 Hz, 3H). ^{13}C NMR (100 MHz, $CDCl_3$) δ 166.0, 165.7, 165.4, 143.9, 137.9, 133.7, 133.6, 130.1, 129.92, 129.87, 129.8, 129.2, 129.1, 128.9, 128.7, 128.6, 128.5, 127.5, 80.4, 77.2, 71.6, 70.2, 70.1, 68.0, 21.7, 17.0. HRMS (ESI) calcd for $C_{34}H_{35}N_2O_9S$ $[M+NH_4]^+$ 647.2058, found 647.2060.

Compound 6o(*17*)

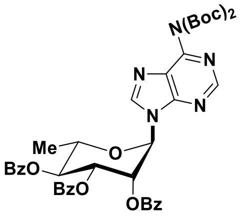

6o

Glycosylation of **1n** (83.4 mg, 0.12 mmol) with **5d** (33.6 mg, 0.10 mmol) according to **General procedure E** afforded **6o** (45.2 mg, 57%) as a white solid: ^{1}H NMR (600 MHz, Chloroform-*d*) δ 8.93 (s, 1H), 8.42 (s, 1H), 8.11 – 8.06 (m, 2H), 8.00 (dd, *J* = 8.3, 1.4 Hz, 2H), 7.89 (dd, *J* = 8.3, 1.4 Hz, 2H), 7.63 – 7.49 (m, 3H), 7.47 (t, *J* = 7.8 Hz, 2H), 7.44 – 7.41 (m, 2H), 7.40 – 7.34 (m, 2H), 6.61 (dd, *J* = 5.7, 3.5 Hz, 1H), 6.50 (d, *J* = 5.7 Hz, 1H), 6.16 (dd, *J* = 6.3, 3.6 Hz, 1H), 5.62 (t, *J* = 6.2 Hz, 1H), 4.42 (p, *J* = 6.5 Hz, 1H), 1.62 (s, 3H), 1.42 (s, 18H).

Compound 6p

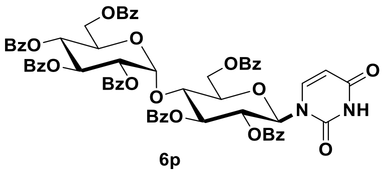

6p

Glycosylation of **1q** (64.4 mg, 0.05 mmol) with **5a** (11.2 mg, 0.10 mmol) according to **General procedure F** afforded **6p** (50.0 mg, 86%) as a white solid: $[\alpha]_D^{20}$= 57.5 (*c* 1.25, $CHCl_3$); ^{1}H NMR (600 MHz, Chloroform-*d*) δ 8.46 (s, 1H), 8.09 (dd, *J* = 8.0, 1.4 Hz, 2H), 8.02 – 7.97 (m, 2H), 7.90 – 7.85 (m, 2H), 7.77 – 7.70 (m, 6H), 7.63 – 7.58 (m, 2H), 7.56 – 7.50 (m, 1H), 7.51 – 7.44 (m, 4H), 7.46 – 7.35 (m, 6H), 7.37 – 7.27 (m, 6H), 7.27 – 7.18 (m, 6H), 6.16 (d, *J* = 9.4 Hz, 1H), 6.09 (t, *J* = 10.0 Hz, 1H), 5.96 (t, *J* = 9.3 Hz, 1H), 5.78 (d, *J* = 4.0 Hz, 1H), 5.74 (d, *J* = 8.3 Hz, 1H), 5.68 (t, *J* = 9.7 Hz, 1H), 5.43 (t, *J* = 9.4 Hz, 1H), 5.31 – 5.24 (m, 1H), 4.95 (dd, *J* = 12.4, 2.3 Hz, 1H), 4.76 (dd, *J* = 12.4, 4.2 Hz, 1H), 4.54 (t, *J* = 9.4 Hz, 1H), 4.51 – 4.44 (m, 2H), 4.35 – 4.26 (m, 2H). ^{13}C NMR (150 MHz, $CDCl_3$) δ 166.1, 166.0, 165.7, 165.6, 165.4, 165.2, 164.8, 162.2, 150.0, 139.0, 133.9, 133.8, 133.6, 133.5, 133.2, 130.1, 130.0, 129.91, 129.86, 129.8, 129.7, 129.6, 129.3, 129.0, 128.9, 128.8, 128.52, 128.47, 128.44, 128.41, 128.32, 128.29, 127.8, 103.7, 96.7, 80.4, 77.2, 76.2,

75.1, 72.9, 70.9, 70.5, 69.9, 69.5, 69.2, 63.1, 62.6. HRMS (ESI) calcd for $C_{65}H_{56}N_3O_{19}$ $[M+NH_4]^+$ 1182.3503, found 1182.3503.

Compound 6q

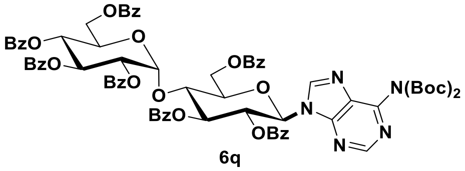

Glycosylation of **1q** (77.3 mg, 0.06 mmol) with **5d** (16.8 mg, 0.05 mmol) according to **General procedure E** afforded **6q** (38.1 mg, 55%) as a white solid: $[\alpha]_D^{20}$= 33.7 (*c* 0.70, $CHCl_3$); ^{1}H NMR (600 MHz, Chloroform-*d*) δ 8.74 (s, 1H), 8.27 (s, 1H), 8.08 (dd, *J* = 8.3, 1.4 Hz, 2H), 8.00 (dd, *J* = 8.3, 1.4 Hz, 2H), 7.89 (dd, *J* = 8.3, 1.4 Hz, 2H), 7.79 – 7.71 (m, 4H), 7.65 – 7.60 (m, 2H), 7.62 – 7.55 (m, 1H), 7.57 – 7.50 (m, 3H), 7.52 – 7.27 (m, 15H), 7.25 – 7.18 (m, 4H), 7.19 – 7.13 (m, 2H), 6.25 (d, *J* = 9.3 Hz, 1H), 6.12 (t, *J* = 9.9 Hz, 1H), 6.02 (t, *J* = 9.3 Hz, 1H), 5.93 (t, *J* = 9.4 Hz, 1H), 5.82 (d, *J* = 3.9 Hz, 1H), 5.68 (t, *J* = 9.7 Hz, 1H), 5.29 (dd, *J* = 10.4, 3.9 Hz, 1H), 4.98 (dd, *J* = 12.4, 2.4 Hz, 1H), 4.79 (dd, *J* = 12.3, 4.3 Hz, 1H), 4.70 (t, *J* = 9.2 Hz, 1H), 4.57 – 4.48 (m, 2H), 4.49 (dd, *J* = 12.2, 3.2 Hz, 1H), 4.42 (ddd, *J* = 9.6, 4.2, 2.4 Hz, 1H), 4.34 (dd, *J* = 12.2, 3.9 Hz, 1H), 1.30 (s, 18H). ^{13}C NMR (150 MHz, $CDCl_3$) δ 166.2, 166.0, 165.8, 165.6, 165.2, 165.0, 153.2, 152.5, 150.8, 150.1, 142.2, 133.72, 133.67, 133.6, 133.5, 133.3, 133.2, 130.2, 130.1, 130.0, 129.89, 129.85, 129.7, 129.4, 129.1, 128.9, 128.8, 128.7, 128.5, 128.4, 128.34, 128.31, 128.29, 127.7, 96.8, 83.8, 81.0, 77.2, 76.5, 75.3, 72.9, 71.5, 71.1, 70.0, 69.6, 69.4, 63.1, 62.7, 27.8. HRMS (ESI) calcd for $C_{76}H_{70}N_5O_{21}$ $[M+H]^+$ 1388.4558, found 1388.4563.

Compound 6r(*86*)

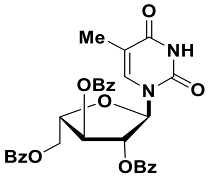

Glycosylation of **1o** (68.2 mg, 0.10 mmol) with **5b** (25.2 mg, 0.20 mmol) according to **General procedure F** afforded **6r** (56.5 mg, 99%) as a white solid: ^{1}H NMR (600 MHz, Chloroform-*d*) δ 8.70 (s, 1H), 8.12 – 8.07 (m, 2H), 8.06 – 7.99 (m, 4H), 7.61 (q, *J* = 7.4 Hz, 2H), 7.55 (t, *J* = 7.4 Hz, 1H), 7.50 – 7.43 (m, 4H), 7.40 (t, *J* = 7.7 Hz, 2H), 7.31 – 7.28 (m, 1H), 6.27 (d, *J* = 3.3 Hz, 1H), 5.93 (t, *J* = 3.0 Hz, 1H), 5.76 (t, *J* = 3.1 Hz, 1H), 5.01 – 4.95 (m, 1H), 4.75 (dd, *J* = 11.9, 5.9 Hz, 1H), 4.67 (dd, *J* = 11.9, 4.6 Hz, 1H), 1.92 (s, 3H).

Compound 6s(*87*)

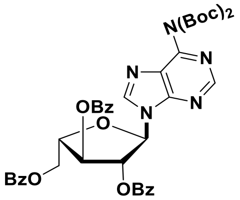

Glycosylation of **1o** (82.0 mg, 0.12 mmol) with **5d** (33.6 mg, 0.10 mmol) according to **General procedure E** afforded **6s** (60.8 mg, 78%) as a white solid: ^{1}H NMR (400 MHz, Chloroform-*d*) δ 8.88 (s, 1H), 8.35 (s, 1H), 8.13 – 8.05 (m, 2H), 8.07 – 8.00 (m, 2H), 7.96 – 7.89 (m, 2H), 7.66 – 7.50 (m, 3H), 7.46 (t, *J* = 7.7 Hz, 4H), 7.38 (t, *J* = 7.7 Hz, 2H), 6.58 – 6.50 (m, 2H), 5.92 (dd, *J* =

4.6, 2.8 Hz, 1H), 5.10 (q, J = 4.8 Hz, 1H), 4.80 (d, J = 4.9 Hz, 2H), 1.46 (s, 18H). ^{13}C NMR (100 MHz, $CDCl_3$) δ 166.3, 165.6, 165.4, 153.0, 152.6, 150.9, 150.5, 143.2, 134.2, 134.1, 133.5, 130.2, 130.0, 129.6, 129.5, 128.9, 128.8, 128.62, 128.58, 128.4, 89.4, 84.0, 83.5, 80.7, 77.2, 63.7, 28.0.

Compound 6t(*17*)

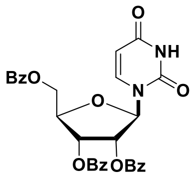

6t

Glycosylation of **1j** (68.2 mg, 0.10 mmol) with **5a** (22.4 mg, 0.20 mmol) according to **General procedure F** afforded **6t** (55.0 mg, 99%) as a white solid: ^{1}H NMR (400 MHz, Chloroform-*d*) δ 8.13 – 8.04 (m, 2H), 7.99 – 7.88 (m, 4H), 7.67 – 7.32 (m, 15H), 7.30 – 7.26 (m, 1H), 6.66 – 6.56 (m, 1H), 6.37 – 6.24 (m, 2H), 5.89 (dd, J = 6.0, 4.7 Hz, 1H), 5.81 – 5.72 (m, 1H), 5.62 (dd, J = 8.1, 1.8 Hz, 1H), 4.82 (dt, J = 11.8, 2.1 Hz, 1H), 4.75 – 4.63 (m, 2H).

Compound 6u(*17*)

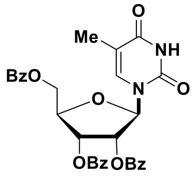

6u

Glycosylation of **1j** (68.2 mg, 0.10 mmol) with **5b** (25.2 mg, 0.20 mmol) according to **General procedure F** afforded **6u** (56.4 mg, 99%) as a white solid: ^{1}H NMR (400 MHz, Chloroform-*d*) δ 9.02 (s, 1H), 8.18 – 8.10 (m, 2H), 7.96 (ddd, J = 14.4, 8.3, 1.4 Hz, 4H), 7.66 – 7.45 (m, 5H), 7.44 – 7.31 (m, 4H), 7.17 (d, J = 1.4 Hz, 1H), 6.43 (d, J = 6.3 Hz, 1H), 5.91 (dd, J = 6.0, 3.8 Hz, 1H), 5.76 (t, J = 6.2 Hz, 1H), 4.88 (dd, J = 12.1, 2.6 Hz, 1H), 4.73 – 4.61 (m, 2H), 2.73 (s, 3H).

Compound 6v(*17*)

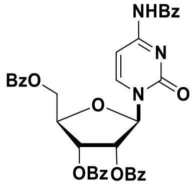

6v

Glycosylation of **1j** (68.2 mg, 0.10 mmol) with **5c** (43.0 mg, 0.20 mmol) according to **General procedure F** afforded **6v** (62.7 mg, 95%) as a white solid: ^{1}H NMR (600 MHz, Chloroform-*d*) δ 8.99 (d, J = 126.3 Hz, 1H), 8.12 – 8.08 (m, 2H), 8.00 – 7.89 (m, 7H), 7.65 – 7.47 (m, 9H), 7.39 – 7.32 (m, 4H), 6.46 (d, J = 4.5 Hz, 1H), 5.92 (t, J = 5.6 Hz, 1H), 5.87 (dd, J = 5.8, 4.4 Hz, 1H), 4.86 (dd, J = 12.2, 2.9 Hz, 1H), 4.82 – 4.76 (m, 1H), 4.73 (dd, J = 12.3, 4.1 Hz, 1H).

Compound 6w(*85*)

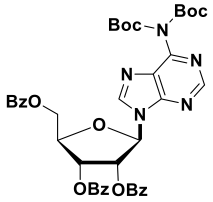

6w

Glycosylation of **1j** (82.0 mg, 0.12 mmol) with **5d** (33.6 mg, 0.10 mmol) according to **General procedure E** afforded **6w** (60.8 mg, 78%) as a white solid: ^{1}H NMR (600 MHz, Chloroform-*d*) δ 8.75 (s, 1H), 8.23 (s, 1H), 8.11 (dd, *J* = 8.3, 1.4 Hz, 2H), 8.01 (dd, *J* = 8.3, 1.4 Hz, 2H), 7.91 (dd, *J* = 8.3, 1.3 Hz, 2H), 7.63 – 7.50 (m, 3H), 7.49 – 7.43 (m, 2H), 7.45 – 7.39 (m, 2H), 7.39 – 7.32 (m, 2H), 6.49 (d, *J* = 5.3 Hz, 1H), 6.38 (t, *J* = 5.6 Hz, 1H), 6.26 (dd, *J* = 5.8, 4.7 Hz, 1H), 4.91 (dd, *J* = 12.2, 3.3 Hz, 1H), 4.87 – 4.82 (m, 1H), 4.72 (dd, *J* = 12.2, 4.3 Hz, 1H), 1.43 (s, 18H). ^{13}C NMR (151 MHz, $CDCl_3$) δ 166.3, 165.5, 165.2, 153.0, 152.6, 150.8, 150.4, 143.4, 134.0, 133.9, 133.6, 130.0, 129.9, 129.6, 129.4, 128.8, 128.72, 128.66, 128.4, 87.1, 84.0, 81.0, 77.2, 74.0, 71.6, 63.7, 27.9.

Compound 6x

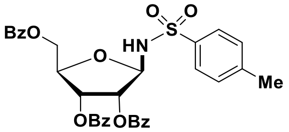

6x

Glycosylation of **1j** (82.0 mg, 0.12 mmol) with **5e** (17.1 mg, 0.10 mmol) according to **General procedure E** afforded **6x** (54.8 mg, 89%) as a white solid: $[\alpha]_D^{25}$= 2.8 (*c* 1.13, CH_2Cl_2); ^{1}H NMR (600 MHz, Chloroform-*d*) δ 8.05 – 8.00 (m, 2H), 7.94 – 7.86 (m, 4H), 7.73 (d, *J* = 8.3 Hz, 2H), 7.60 – 7.51 (m, 3H), 7.44 (t, *J* = 7.8 Hz, 2H), 7.39 – 7.34 (m, 4H), 7.07 (d, *J* = 8.1 Hz, 2H), 6.01 (d, *J* = 9.6 Hz, 1H), 5.72 (dd, *J* = 5.6, 3.0 Hz, 1H), 5.64 (dd, *J* = 9.6, 6.2 Hz, 1H), 5.57 (t, *J* = 5.9 Hz, 1H), 4.51 – 4.47 (m, 2H), 2.24 (s, 3H). ^{13}C NMR (150 MHz, $CDCl_3$) δ 166.3, 165.5, 165.4, 143.6, 138.2, 133.7, 133.5, 130.0, 129.9, 129.8, 129.6, 129.0, 128.7, 128.6, 128.5, 127.2, 86.6, 79.7, 77.2, 74.5, 71.6, 64.3, 21.5. HRMS (ESI) calcd for $C_{33}H_{33}N_2O_9S$ $[M+NH_4]^+$ 633.1901, found 633.1900.

Para-Nitrophenyl (PNP), *para*-NHFmoc phenyl (PNFP), *para*-azidophenyl (PAP) groups as stable protective groups for the synthesis of active donors and latent acceptors

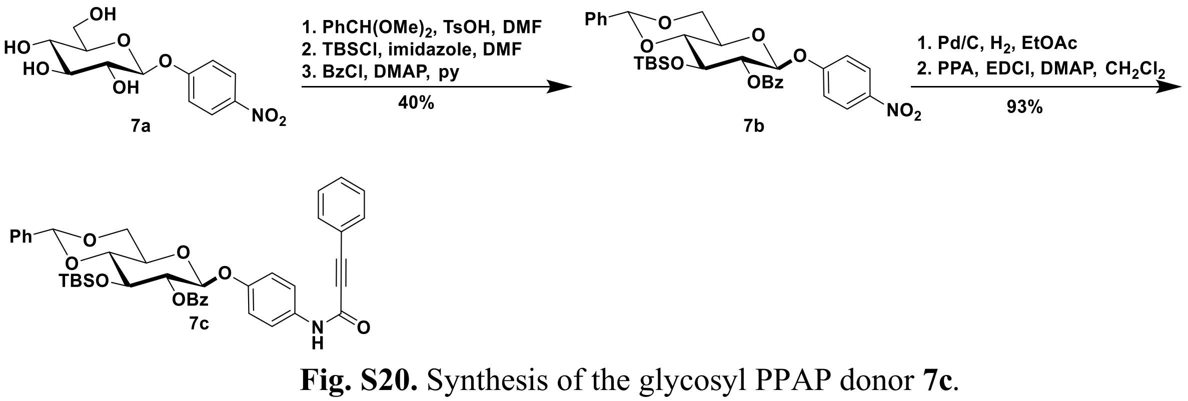

Fig. S20. Synthesis of the glycosyl PPAP donor **7c**.

Compound 7b

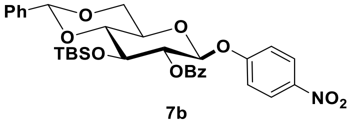

7b

To a solution of **7a** (6.0 g, 20.0 mmol) in dry DMF was added benzaldehyde dimethylacetal (11.6 mL, 80.0 mmol) followed by *p*-toluenesulfonic acid (1.72 g, 10.0 mmol). The solution was stirred at the 50 °C for 3 h, at which time TLC showed that all starting material disappeared. EtOAc was

added to dilute the reaction, and the resultant solution was washed with water, 1M HCl, saturated aqueous $NaHCO_3$, and brine, and then dried over Na_2SO_4. Filtration and concentration give the crude product which was used for the next step directly. The above obtained crude product was dissolved in DMF, and the imidazole (2.4 g, 40.0 mmol) and TBSCl (4. 5 g, 30.0 mmol) were added. The resulting mixture was stirred at room temperature for 2 h, at which time TLC showed that all starting material disappeared. EtOAc was added to dilute the reaction, and the resultant solution was washed with water, 1M HCl, saturated aqueous $NaHCO_3$, and brine, and then dried over Na_2SO_4. Filtration and concentration give the crude product. To a solution of the above the crude product in pyridine (80 mL) was added benzoyl chloride (7.0 mL, 60.0 mmol). The reaction mixture was stirred at room temperature and monitored by TLC. The mixture was cooled using an ice-bath, quenched by addition of MeOH (15 mL), and then concentrated in *vacuo*. EtOAc was added to form a pyridine salt and filtered, then the resultant solution was washed with water, 1M HCl, saturated aqueous $NaHCO_3$, extracted with EtOAc. The organic phase was washed with brine, dried over Na_2SO_4, and evaporated to dryness. The residue was purified by silica gel column chromatography (Petroleum ether/EtOAc = 10:1) to give **7b** (4.8 g, 40% for three steps) as a white solid: $[\alpha]_D^{20}$= -13.6 (*c* 0.39, $CHCl_3$); ^{1}H NMR (600 MHz, Chloroform-*d*) δ 8.13 – 8.07 (m, 2H), 7.60 – 7.53 (m, 1H), 7.45 (t, *J* = 7.8 Hz, 2H), 7.41 – 7.36 (m, 2H), 7.31 – 7.27 (m, 3H), 6.89 (d, *J* = 8.8 Hz, 2H), 6.66 (d, *J* = 8.8 Hz, 2H), 5.63 (t, *J* = 9.3 Hz, 1H), 5.49 (s, 1H), 5.04 (d, *J* = 7.4 Hz, 1H), 4.38 (dd, *J* = 10.5, 5.0 Hz, 1H), 4.03 (dd, *J* = 8.9, 7.4 Hz, 1H), 3.85 – 3.77 (m, 2H), 3.67 (td, *J* = 9.7, 5.0 Hz, 1H), 0.75 (s, 9H), 0.10 (s, 3H), -0.02 (s, 3H). ^{13}C NMR (150 MHz, $CDCl_3$) δ 165.6, 149.8, 137.0, 133.1, 130.2, 130.0, 129.0, 128.5, 128.4, 128.3, 126.2, 118.2, 116.3, 102.5, 101.4, 78.9, 77.2, 74.8, 73.9, 68.8, 66.4, 25.7, 18.0, -3.9, -4.7. HRMS (ESI) calcd for $C_{32}H_{38}NO_9Si$ $[M+H]^+$ 608.2310, found 608.2310.

Compound 7c

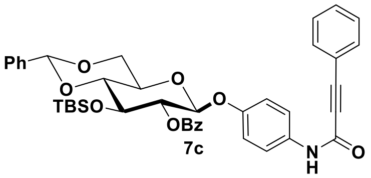

7c

A suspension of **7b** (1.2 g, 2.0 mmol) and 5% Pd/C (0.12 g) in EtOAc was degased and charged with H_2 at 1 atm for 3~6 h at room temperature until TLC-analysis indicated the reaction to be complete. After the Pd/C was removed by filtration, the filtrate was evaporated under vacuum to afford the amine, which was used for the next step directly. Similar procedure as that used for the synthesis of **1a** (**General procedure D**) was applied to the preparation of **7c** (1.3 g, 93% for two steps) as a white foam: $[\alpha]_D^{20}$= 19.6 (*c* 0.28, $CHCl_3$); ^{1}H NMR (600 MHz, Chloroform-*d*) δ 8.16 (s, 1H), 8.08 (dd, *J* = 8.2, 1.5 Hz, 2H), 7.60 – 7.54 (m, 1H), 7.55 – 7.50 (m, 2H), 7.49 – 7.33 (m, 10H), 7.32 – 7.24 (m, 2H), 6.87 (d, *J* = 9.0 Hz, 2H), 5.55 – 5.48 (m, 2H), 5.14 (d, *J* = 8.0 Hz, 1H), 4.38 (dd, *J* = 10.6, 4.8 Hz, 1H), 4.15 (t, *J* = 8.9 Hz, 1H), 3.84 (t, *J* = 10.2 Hz, 1H), 3.74 – 3.59 (m, 2H), 0.74 (s, 9H), 0.01 (s, 3H), -0.08 (s, 3H). ^{13}C NMR (150 MHz, $CDCl_3$) δ 165.3, 154.2, 151.3, 137.1, 133.3, 132.9, 132.6, 130.3, 129.9, 129.8, 129.2, 128.6, 128.5, 128.2, 126.4, 121.8, 120.0, 118.0, 101.9, 100.8, 85.9, 83.5, 81.3, 77.2, 74.9, 73.0, 68.6, 66.7, 25.6, 18.0, -4.1, -4.9. HRMS (ESI) calcd for $C_{41}H_{44}NO_8Si$ $[M+H]^+$ 706.2831, found 706.2832.

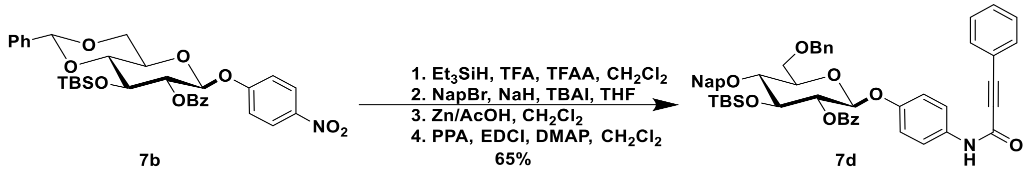

Fig. S21. Synthesis of the glycosyl PPAP donor **7d**.

Compound 7d

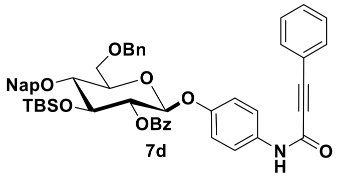

To a solution of **7b** (2.0 g, 3.36 mmol) in dry CH_2Cl_2 (25.0 mL), Et_3SiH (3.2 mL, 20.2 mmol) was added slowly, followed by TFA (1.03 mL, 13.4 mmol) and TFAA (0.63 mL, 3.7 mmol) at 0 °C under N_2 atmosphere. After the stirring was continued for 5 minutes, saturated aqueous $NaHCO_3$ was added to quench the reaction. The resultant mixture was washed successively with water, brine, dried over Na_2SO_4, and evaporated to afford crude product. To a solution of above crude product in dry THF was added 60% NaH (0.4 g, 10.0 mmol) at 0 °C. After stirring at the same temperature for 10 minutes, NapBr (1.1 g, 5.0 mmol) and TBAI (0.37 g, 1.0 mmol) were added. The reaction mixture was warmed to room temperature gradually, and the stirring was continued for another 3 h. EtOAc was added to dilute the reaction, the resultant mixture was washed successively with water, 1M HCl, saturated aqueous $NaHCO_3$, brine, and then dried over Na_2SO_4. Filtration was followed by concentration to give the crude product which was further dissolved in CH_2Cl_2, zinc powder and acetic acid were added sequentially at 0 °C. The reaction mixture was warmed to room temperature gradually, and the stirring was continued for another 2 h. After the zinc powder was removed by filtration, the filtrate was washed successively with water, brine, and then dried over Na_2SO_4. Filtration was followed by concentration to give the crude product which was used to prepare of **7d** (1.85 g, 65% for four steps) according to **General procedure D**. $[\alpha]_D^{20}$= 57.5 (*c* 2.4, $CHCl_3$); ^{1}H NMR (600 MHz, Chloroform-*d*) δ 7.92 – 7.87 (m, 2H), 7.70 – 7.65 (m, 1H), 7.62 – 7.56 (m, 5H), 7.55 – 7.48 (m, 3H), 7.49 – 7.36 (m, 6H), 7.36 – 7.30 (m, 6H), 7.32 – 7.26 (m, 1H), 7.17 (dd, *J* = 8.4, 1.6 Hz, 1H), 7.16 – 7.10 (m, 2H), 5.46 (t, *J* = 9.2 Hz, 1H), 5.15 (d, *J* = 7.7 Hz, 1H), 4.98 (d, *J* = 11.8 Hz, 1H), 4.84 (d, *J* = 11.8 Hz, 1H), 4.64 (d, *J* = 12.2 Hz, 1H), 4.54 (d, *J* = 12.1 Hz, 1H), 3.91 (t, *J* = 9.0 Hz, 1H), 3.84 – 3.74 (m, 2H), 3.71 – 3.64 (m, 2H), 0.73 (s, 9H), -0.03 (s, 3H), -0.23 (s, 3H). ^{13}C NMR (150 MHz, $CDCl_3$) δ 165.7, 154.4, 151.0, 138.2, 135.1, 133.2, 133.04, 133.01, 132.7, 132.6, 130.4, 130.3, 129.9, 128.7, 128.5, 128.3, 128.2, 128.1, 127.74, 127.71, 127.66, 127.4, 126.5, 126.0, 125.9, 121.7, 120.1, 117.8, 102.2, 85.8, 83.6, 78.6, 77.2, 76.5, 74.2, 73.5, 69.5, 68.8, 25.8, 18.0, -4.0, -4.6. HRMS (ESI) calcd for $C_{52}H_{53}NO_8SiNa$ $[M+Na]^+$ 870.3433, found 870.3433.

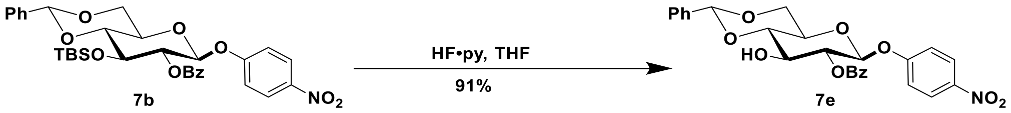

Fig. S22. Synthesis of the Compound **7e**.

Compound 7e

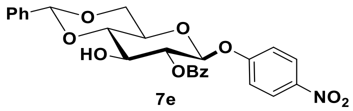

7e

To a solution of **7b** (0.5 g, 0.82 mmol) in THF (5 mL) was added HF·py (1.1 mL, 12.3 mmol) at 0 °C. After being stirred at room temperature for 8 h under nitrogen, the reaction mixture was evaporated under vacuum. The residue was dissolved in CH_2Cl_2 and washed with sat. $NaHCO_3$, H_2O, and brine, respectively. The organic layer was dried (Na_2SO_4) and concentrated. The residue was chromatographed on silica gel (Petroleum ether/EtOAc = 4:1) to afford **7e** (91%) as a white solid: $[\alpha]_D^{20}$= -15.5 (*c* 0.22, THF); ^{1}H NMR (600 MHz, Chloroform-*d*) δ 8.19 – 8.13 (m, 2H), 8.07 – 8.01 (m, 2H), 7.63 – 7.54 (m, 1H), 7.54 – 7.50 (m, 2H), 7.44 (t, *J* = 7.8 Hz, 2H), 7.42 – 7.38 (m, 3H), 7.08 – 7.02 (m, 2H), 5.61 (s, 1H), 5.51 (dd, *J* = 9.0, 7.7 Hz, 1H), 5.38 (d, *J* = 7.7 Hz, 1H), 4.45 (dd, *J* = 10.6, 4.9 Hz, 1H), 4.17 (t, *J* = 9.1 Hz, 1H), 3.87 (t, *J* = 10.3 Hz, 1H), 3.80 (t, *J* = 9.3 Hz, 1H), 3.74 (td, *J* = 9.6, 4.8 Hz, 1H), 2.91 (s, 1H). ^{13}C NMR (150 MHz, $CDCl_3$) δ 165.8, 161.4, 143.3, 136.7, 133.8, 130.1, 130.0, 129.6, 129.2, 128.7, 128.6, 128.4, 126.4, 126.2, 125.9, 116.9, 116.8, 102.2, 99.1, 80.5, 77.2, 74.3, 72.4, 68.5, 66.8. HRMS (ESI) calcd for $C_{26}H_{24}NO_9$ $[M+H]^+$ 494.1446, found 494.1450.

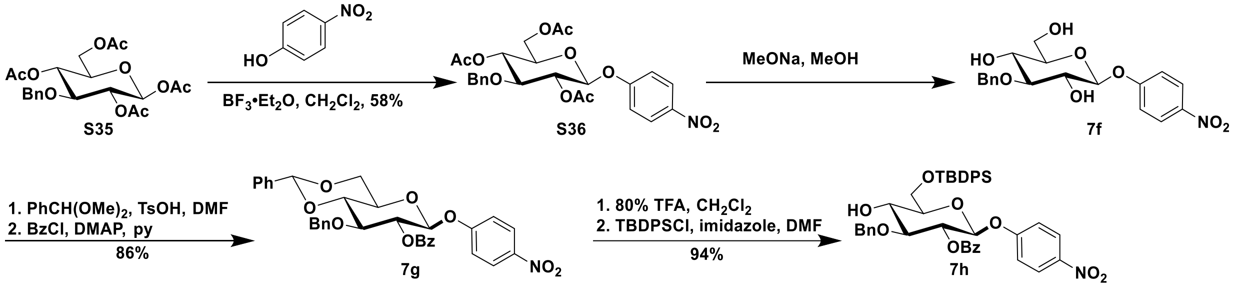

Fig. S23. Synthesis of the Compound **7h**.

Compound S36

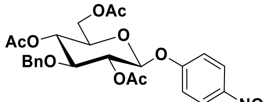

S36

Compound **S36** was prepared from **S35** (3.26 g, 7.4 mmol) according to the synthesis of **S14** (**General procedure B**) to afford **S36** (2.2 g, 58%) as a white foam: $[\alpha]_D^{20}$= -29.1 (*c* 3.54, $CHCl_3$); ^{1}H NMR (400 MHz, Chloroform-*d*) δ 8.15 (d, *J* = 9.2 Hz, 2H), 7.36 – 7.24 (m, 3H), 7.21 (dd, *J* = 8.0, 1.7 Hz, 2H), 7.02 (d, *J* = 9.2 Hz, 2H), 5.31 (dd, *J* = 9.0, 7.5 Hz, 1H), 5.16 (t, *J* = 9.5 Hz, 1H), 5.10 (d, *J* = 7.5 Hz, 1H), 4.61 (s, 2H), 4.19 (dd, *J* = 12.3, 5.8 Hz, 1H), 4.12 (dd, *J* = 12.4, 2.7 Hz, 1H), 3.85 – 3.74 (m, 2H), 2.02 (s, 3H), 1.98 (d, *J* = 1.3 Hz, 6H). ^{13}C NMR (100 MHz, $CDCl_3$) δ 170.6, 169.4, 169.2, 161.5, 143.2, 137.6, 128.6, 128.1, 127.9, 125.8, 116.7, 98.4, 79.6, 77.2, 74.2, 72.8, 72.1, 69.4, 62.3, 20.9, 20.83, 20.79. HRMS (ESI) calcd for $C_{25}H_{31}N_2O_{11}$ $[M+NH_4]^+$ 535.1922, found 535.1917.

Compound 7g

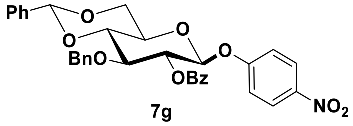

7g

To a solution of compound **S36** (2.0 g, 2.0 mmol) was added MeONa (0.2 mL, 5M), After the reaction mixture was stirred for 2 h at room temperature. The mixture was neutralized with Amberlite IR-120 (H) resin and then concentrated to give **7f**. To a solution of **7f** in dry DMF was

added benzaldehyde dimethylacetal (1.16 mL, 8.0 mmol) followed by *p*-toluenesulfonic acid (0.17 g, 1.0 mmol). The solution was stirred at the 50 °C for 3 h, at which time TLC showed that all starting material disappeared. EtOAc was added to dilute the reaction, and the resultant solution was washed with water, 1M HCl, saturated aqueous $NaHCO_3$, and brine, and then dried over Na_2SO_4. Filtration and concentration give the crude product which was used for the next step directly. The above obtained crude product was dissolved in pyridine (80 mL), and benzoyl chloride (0.7 mL, 6.0 mmol) was added in 0 °C. The reaction mixture was stirred at room temperature and monitored by TLC. The mixture was cooled using an ice-bath, quenched by addition of MeOH (1.5 mL), and then concentrated in *vacuo*. EtOAc was added to form a pyridine salt and filtered, then the resultant solution was washed with water, 1M HCl, saturated aqueous $NaHCO_3$, extracted with EtOAc. The organic phase was washed with brine, dried over Na_2SO_4, and evaporated to dryness. The residue was purified by silica gel column chromatography (Petroleum ether/EtOAc, 4:1) to give **7g** (1.0 g, 86% for three steps) as a white solid: $[\alpha]_D^{20}$= 34.7 (*c* 2.33, $CHCl_3$); ^{1}H NMR (400 MHz, Chloroform-*d*) δ 8.19 – 8.10 (m, 2H), 8.03 – 7.95 (m, 2H), 7.65 – 7.55 (m, 1H), 7.54 (dd, *J* = 7.4, 2.3 Hz, 2H), 7.51 – 7.38 (m, 5H), 7.21 – 7.06 (m, 5H), 7.08 – 6.99 (m, 2H), 5.66 (s, 1H), 5.65 – 5.56 (m, 1H), 5.35 (d, *J* = 7.6 Hz, 1H), 4.88 (d, *J* = 12.0 Hz, 1H), 4.75 (d, *J* = 11.9 Hz, 1H), 4.46 (dd, *J* = 10.5, 4.9 Hz, 1H), 4.06 – 3.96 (m, 2H), 3.89 (t, *J* = 10.3 Hz, 1H), 3.79 – 3.70 (m, 1H). ^{13}C NMR (150 MHz, $CDCl_3$) δ 165.1, 161.4, 143.2, 137.6, 137.0, 133.6, 130.0, 129.4, 129.3, 128.6, 128.5, 128.44, 128.38, 128.3, 127.9, 126.1, 125.9, 116.8, 101.6, 99.1, 81.3, 77.6, 77.2, 74.2, 72.9, 68.6, 66.8. HRMS (ESI) calcd for $C_{33}H_{30}NO_9$ $[M+H]^+$ 584.1915, found 584.1910.

Compound 7h

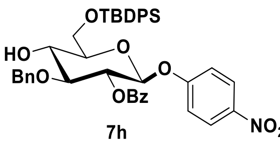

7h

To a solution of **7g** (0.9 g, 1.54 mmol) in CH_2Cl_2 (6.0 mL), 80% TFA (0.64 mL) was added. After the stirring was continued for 1 h, saturated aqueous $NaHCO_3$ was added to quench the reaction. The resultant mixture was washed successively with water, brine, dried over Na_2SO_4, and evaporated to afford crude product. To a solution of above crude product and imidazole (204 mg, 3.0 mmol) in DMF (5 mL), was added dropwise a solution of TBDPSCl (0.48 mL, 1.8 mmol). The resulting mixture was stirred at room temperature for 2 h, at which time TLC showed that all starting material disappeared. EtOAc was added to dilute the reaction, and the resultant solution was washed with water, 1M HCl, saturated aqueous $NaHCO_3$, and brine, and then dried over Na_2SO_4. Filtration and concentration give the crude product. The residue was chromatographed on silica gel (Petroleum ether/EtOAc = 4:1) to afford **7h** (1.06g, 94% for two steps) as a white solid: $[\alpha]_D^{25}$= -14.6 (*c* 1.91, $CHCl_3$); ^{1}H NMR (600 MHz, Chloroform-*d*) δ 8.09 – 8.01 (m, 4H), 7.68 – 7.63 (m, 4H), 7.62 – 7.57 (m, 1H), 7.46 (t, *J* = 7.8 Hz, 2H), 7.43 – 7.38 (m, 2H), 7.34 (t, *J* = 7.5 Hz, 2H), 7.29 (t, *J* = 7.5 Hz, 2H), 7.22 (s, 4H), 7.06 – 7.00 (m, 2H), 5.55 (dd, *J* = 9.4, 7.8 Hz, 1H), 5.24 (d, *J* = 7.8 Hz, 1H), 4.78 (d, *J* = 11.5 Hz, 1H), 4.71 (d, *J* = 11.5 Hz, 1H), 4.03 (dd, *J* = 11.1, 3.4 Hz, 1H), 3.96 – 3.85 (m, 2H), 3.80 (t, *J* = 9.1 Hz, 1H), 3.72 – 3.65 (m, 1H), 2.61 (s, 1H), 1.07 (s, 9H). ^{13}C NMR (150 MHz, $CDCl_3$) δ 165.3, 161.8, 143.0, 137.8, 135.8, 135.7, 133.6, 133.1, 132.8, 130.0, 129.9, 129.6, 128.7, 128.23, 128.19, 127.90, 127.88, 125.8, 116.9, 98.9, 82.3, 77.2, 76.5, 74.9, 73.1, 71.0, 63.8, 26.9, 19.4. HRMS (ESI) calcd for $C_{42}H_{47}N_2O_9Si$ $[M+NH_4]^+$ 751.3045, found 751.3042.

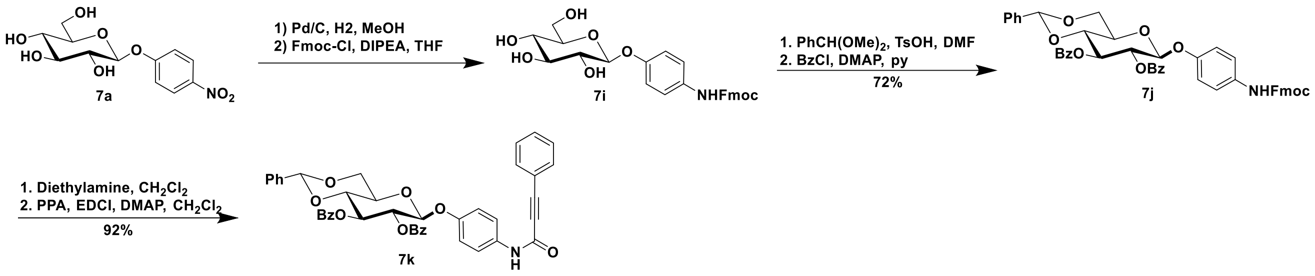

Fig. S24. Synthesis of the Compound **7k**.

Compound 7j

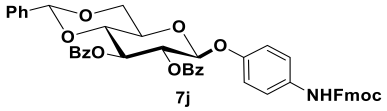

A suspension of **7a** (1.5 g, 5.0 mmol) and 5% Pd/C (0.15 g) in MeOH (100mL) was degased and charged with H_2 at 1 atm for 3~6 h at room temperature until TLC-analysis indicated the reaction to be complete. After the Pd/C was removed by filtration, the filtrate was evaporated under vacuum to afford the amine for the next step without purification. To a solution of amine in dry DMF was added DIPEA (1.74 mL, 10.0 mmol) followed by Fmoc-Cl (1.6 g, 6.0 mmol) at 0 °C, then the reaction was warmed to room temperature. After stirred at room temperature for 3 h, at which time TLC showed that all starting material disappeared. The mixture was concentrated to afford **7i**. To a solution of **7i** in dry DMF was added benzaldehyde dimethylacetal (2.9 mL, 20.0 mmol) followed by *p*-toluenesulfonic acid (0.43 g, 2.5 mmol). The solution was stirred at the 50 °C for 3 h, at which time TLC showed that all starting material disappeared. EtOAc was added to dilute the reaction, and the resultant solution was washed with water, 1M HCl, saturated aqueous $NaHCO_3$, and brine, and then dried over Na_2SO_4. Filtration and concentration give the crude product which was used for the next step directly. To a solution of the above the crude product in pyridine (20 mL) was added benzoyl chloride (3.5 mL, 30.0 mmol). The reaction mixture was stirred at room temperature and monitored by TLC. The mixture was cooled using an ice-bath, quenched by addition of MeOH (7 mL), and then concentrated in *vacuo*. EtOAc was added to form a pyridine salt and filtered, then the resultant solution was washed with water, 1M HCl, saturated aqueous $NaHCO_3$, extracted with EtOAc. The organic phase was washed with brine, dried over Na_2SO_4, and evaporated to dryness. The residue was purified by silica gel column chromatography (Petroleum ether/EtOAc = 10:1) to give **7j** (2.84 g, 72% for four steps) as a white solid: $[\alpha]^{25}_{D}$= 34.7 (*c* 0.58, $CHCl_3$); ^{1}H NMR (400 MHz, Chloroform-*d*) δ 7.98 (t, *J* = 7.6 Hz, 4H), 7.77 (d, *J* = 7.5 Hz, 2H), 7.59 (d, *J* = 7.3 Hz, 2H), 7.56 – 7.47 (m, 2H), 7.48 – 7.27 (m, 14H), 6.93 (d, *J* = 8.4 Hz, 2H), 6.59 (s, 1H), 5.85 (t, *J* = 9.4 Hz, 1H), 5.72 (t, *J* = 8.5 Hz, 1H), 5.57 (s, 1H), 5.30 (d, *J* = 7.5 Hz, 1H), 4.53 (d, *J* = 6.6 Hz, 2H), 4.47 (dd, *J* = 10.5, 4.8 Hz, 1H), 4.25 (t, *J* = 6.5 Hz, 1H), 4.04 (t, *J* = 9.4 Hz, 1H), 3.94 (d, *J* = 10.1 Hz, 1H), 3.87 – 3.77 (m, 1H). ^{13}C NMR (100 MHz, $CDCl_3$) δ 165.7, 165.4, 143.8, 141.5, 136.8, 133.5, 133.3, 130.0, 129.96, 129.5, 129.2, 128.6, 128.5, 128.4, 127.9, 127.3, 126.3, 125.1, 120.2, 118.4, 101.7, 100.9, 78.7, 77.2, 72.5, 72.2, 68.7, 67.0, 47.3. HRMS (ESI) calcd for $C_{48}H_{43}N_2O_{10}$ $[M+NH_4]^+$ 807.2912, found 807.2911.

Compound 7k

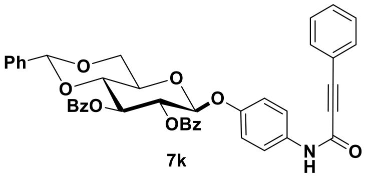

7k

Compound **7j** (1.58 g, 2.0 mmol) was dissolved in dichloromethane/diethylamine (12 mL, *v*:*v*=1:1). Afterwards, the reaction was stirred at room temperature for 2 h until TLC-analysis indicated the reaction to be complete. the mixture was concentrated to afford the amine. Similar procedure as that used for the synthesis of **1a** (**General procedure D**) was applied to the preparation of **7k** (1.28 g, 92% for two steps) as a white foam: $[\alpha]_D^{25}$= 57.9 (*c* 1.01, $CHCl_3$); ^{1}H NMR (600 MHz, Chloroform-*d*) δ 8.16 (s, 1H), 8.08 (dd, *J* = 8.2, 1.5 Hz, 2H), 7.60 – 7.54 (m, 1H), 7.53 (dd, *J* = 8.0, 1.8 Hz, 2H), 7.49 – 7.33 (m, 10H), 7.28 (t, *J* = 7.6 Hz, 2H), 6.87 (d, *J* = 9.0 Hz, 2H), 5.55 – 5.48 (m, 2H), 5.14 (d, *J* = 8.0 Hz, 1H), 4.38 (dd, *J* = 10.6, 4.8 Hz, 1H), 4.15 (t, *J* = 8.9 Hz, 1H), 3.84 (t, *J* = 10.2 Hz, 1H), 3.69 (t, *J* = 9.1 Hz, 1H), 3.66 – 3.61 (m, 1H), 0.74 (s, 10H), 0.01 (s, 3H), -0.08 (s, 3H). ^{13}C NMR (150 MHz, $CDCl_3$) δ 165.3, 154.2, 151.3, 137.1, 133.3, 132.9, 132.6, 130.3, 129.9, 129.8, 129.2, 128.6, 128.5, 128.2, 126.4, 121.8, 120.0, 118.0, 101.9, 100.8, 85.9, 83.5, 81.3, 77.2, 74.9, 73.0, 68.6, 66.7, 25.6, 18.0, -4.1, -4.9. HRMS (ESI) calcd for $C_{42}H_{33}NO_9Na$ $[M+Na]^+$ 718.2048, found 718.2049.

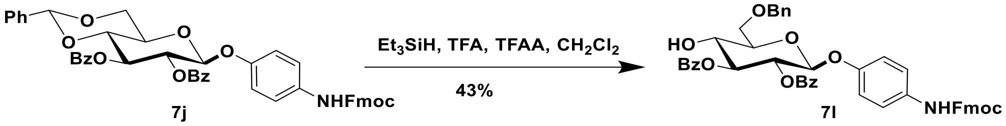

Fig. S25. Synthesis of the Compound **7l**.

Compound 7l

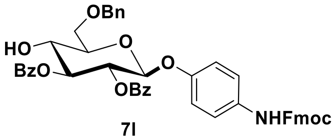

7l

To a solution of **7j** (1.18 g, 1.5 mmol) in dry CH_2Cl_2 (6.0 mL), Et_3SiH (1.43 mL, 9.0 mmol) was added slowly, followed by TFA (0.46 mL, 6.0 mmol) and TFAA (0.254 mL, 1.5 mmol) at 0 °C under N_2 atmosphere. After the stirring was continued for 5 minutes, saturated aqueous $NaHCO_3$ was added to quench the reaction. The resultant mixture was washed successively with water, brine, dried over Na_2SO_4, and evaporated. The residue was purified by silica gel column chromatography (Petroleum ether/EtOAc = 2:1) to give **7l** (0.51 g, 43%) as a white foam: $[\alpha]_D^{20}$= 52.6 (*c* 0.33, $CHCl_3$); ^{1}H NMR (400 MHz, Chloroform-*d*) δ 7.98 (dd, *J* = 15.7, 7.7 Hz, 4H), 7.77 (d, *J* = 7.5 Hz, 2H), 7.60 (d, *J* = 7.4 Hz, 2H), 7.51 (q, *J* = 7.8 Hz, 2H), 7.45 – 7.25 (m, 13H), 6.94 (d, *J* = 8.4 Hz, 2H), 6.65 – 6.52 (m, 1H), 5.68 (dd, *J* = 9.7, 7.9 Hz, 1H), 5.51 (t, *J* = 9.4 Hz, 1H), 5.18 (d, *J* = 7.9 Hz, 1H), 4.70 – 4.56 (m, 2H), 4.53 (d, *J* = 6.6 Hz, 2H), 4.25 (t, *J* = 6.6 Hz, 1H), 4.05 (t, *J* = 9.2 Hz, 1H), 3.96 – 3.77 (m, 3H), 3.32 (s, 1H). ^{13}C NMR (100 MHz, $CDCl_3$) δ 167.4, 165.4, 143.9, 141.5, 137.8, 133.7, 133.4, 130.1, 129.9, 129.4, 129.1, 128.64, 128.58, 128.5, 128.0, 127.91, 127.88, 127.2, 125.1, 120.2, 118.2, 100.3, 77.2, 75.2, 73.9, 71.5, 70.9, 69.9, 66.9, 47.3. HRMS (ESI) calcd for $C_{48}H_{45}N_2O_{10}$ $[M+NH_4]^+$ 809.3069, found 809.3065.

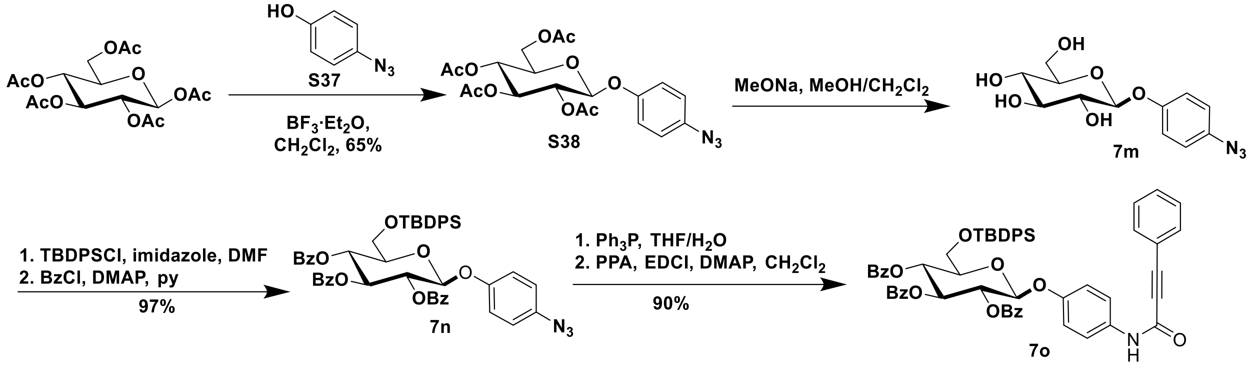

Fig. S26. Synthesis of the Compound **7o**.

Compound S38

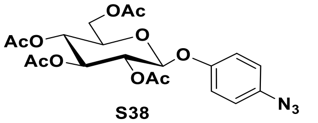

S38

Compound **S38** was prepared from peracetylated glucoside (3.9 g, 10.0 mmol) and **S37**(*88*) (2.0 g, 15.0 mmol) according to the synthesis of **S14** (**General procedure B**) to afford **S38** (3.0 g, 65%) as a white foam: $[\alpha]_D^{25}$= -15.5 (*c* 1.12, $CHCl_3$); ^1H NMR (600 MHz, Chloroform-*d*) δ 7.02 – 6.96 (m, 2H), 6.98 – 6.92 (m, 2H), 5.32 – 5.20 (m, 2H), 5.15 (dd, *J* = 10.0, 9.0 Hz, 1H), 5.02 (d, *J* = 7.8 Hz, 1H), 4.27 (dd, *J* = 12.3, 5.3 Hz, 1H), 4.17 (dd, *J* = 12.3, 2.5 Hz, 1H), 3.87 – 3.80 (m, 1H), 2.06 (d, *J* = 5.4 Hz, 6H), 2.03 (d, *J* = 7.2 Hz, 6H). ^{13}C NMR (150 MHz, $CDCl_3$) δ 170.6, 170.3, 169.5, 169.3, 154.2, 135.5, 120.2, 118.9, 99.8, 77.2, 72.9, 72.3, 71.4, 68.5, 62.1, 20.7. HRMS (ESI) calcd for $C_{20}H_{23}N_3O_{10}Na$ $[M+Na]^+$488.1276, found 488.1280.

Compound 7n

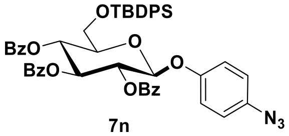

7n

To a solution of compound **S38** (2.9 g, 4.65 mmol) was added MeONa (0.47 mL, 5M), After the reaction mixture was stirred for 2 h at room temperature. The mixture was neutralized with Amberlite IR-120 (H) resin and then concentrated to give **7m**. To a solution of **7m** in dry DMF was added imidazole (1.4 g, 9.3 mmol) and TBDPSCl (1. 45 mL, 5.6 mmol). The solution was stirred at the room temperature for 3 h, at which time TLC showed that all starting material disappeared. EtOAc was added to dilute the reaction, and the resultant solution was washed with water, 1M HCl, saturated aqueous $NaHCO_3$, and brine, and then dried over Na_2SO_4. Filtration and concentration give the crude product which was used for the next step directly. The above obtained crude product was dissolved in pyridine (15 mL), and benzoyl chloride (2.7 mL, 23.0 mmol) was added in 0 °C. The reaction mixture was stirred at room temperature and monitored by TLC. The mixture was cooled using an ice-bath, quenched by addition of MeOH (5.0 mL), and then concentrated in *vacuo*. EtOAc was added to form a pyridine salt and filtered, then the resultant solution was washed with water, 1M HCl, saturated aqueous $NaHCO_3$, extracted with EtOAc. The organic phase was washed with brine, dried over Na_2SO_4, and evaporated to dryness. The residue was purified by silica gel column chromatography (Petroleum ether/EtOAc, 4:1) to give **7n** (3.8 g, 97% for three steps) as a white solid: $[\alpha]_D^{25}$=27.1 (*c* 0.73, $CHCl_3$); ^1H NMR (600 MHz, Chloroform-*d*) δ 7.98 – 7.93 (m, 2H), 7.84 (ddd, *J* = 8.5, 3.7, 1.3 Hz, 4H), 7.66 – 7.62 (m, 2H), 7.60 – 7.55 (m, 2H), 7.54 – 7.46 (m, 2H),

7.44 – 7.39 (m, 1H), 7.38 – 7.33 (m, 4H), 7.33 – 7.27 (m, 3H), 7.25 – 7.18 (m, 4H), 7.05 (d, *J* = 8.9 Hz, 2H), 6.84 (d, *J* = 8.9 Hz, 2H), 5.92 (t, *J* = 9.6 Hz, 1H), 5.74 (dd, *J* = 9.7, 7.8 Hz, 1H), 5.64 (t, *J* = 9.7 Hz, 1H), 5.31 (d, *J* = 7.8 Hz, 1H), 4.05 – 3.98 (m, 1H), 3.95 – 3.84 (m, 2H), 1.04 (s, 9H). ^{13}C NMR (150 MHz, $CDCl_3$) δ 166.0, 165.3, 165.2, 154.6, 135.7, 135.6, 135.2, 133.4, 133.3, 133.2, 132.9, 129.94, 129.92, 129.87, 129.8, 129.4, 129.2, 129.1, 128.54, 128.52, 128.4, 127.8, 120.1, 119.2, 100.4, 77.2, 76.0, 73.3, 72.1, 69.3, 63.0, 26.8, 19.3. HRMS (ESI) calcd for $C_{49}H_{49}N_4O_9Si$ $[M+NH_4]^+$ 865.3263, found 865.3264.

Compound 7o

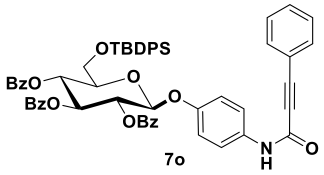

Compound **7n** (2.5 g, 2.94 mmol) was dissolved in THF/H_2O (20 mL, *v*:*v*=2:1). Afterwards, the reaction was stirred at room temperature for 24 h until TLC-analysis indicated the reaction to be complete. The resultant mixture was washed successively with water, brine, dried over Na_2SO_4, and evaporated to afford the amine. Similar procedure as that used for the synthesis of **1a** (**General procedure D**) was applied to the preparation of **7o** (2.5 g, 90% for two steps) as a white foam: $[\alpha]^{25}_{D}$= 45.2 (*c* 0.28, CH_2Cl_2); ^{1}H NMR (600 MHz, Chloroform-*d*) δ 8.11 (s, 1H), 7.99 – 7.93 (m, 2H), 7.88 – 7.82 (m, 4H), 7.68 – 7.63 (m, 2H), 7.60 – 7.55 (m, 2H), 7.51 – 7.42 (m, 6H), 7.40 – 7.27 (m, 8H), 7.25 – 7.16 (m, 8H), 7.03 (d, *J* = 9.0 Hz, 2H), 5.95 (t, *J* = 9.6 Hz, 1H), 5.78 (dd, *J* = 9.7, 7.8 Hz, 1H), 5.70 (t, *J* = 9.6 Hz, 1H), 4.00 (ddd, *J* = 10.0, 5.1, 2.6 Hz, 1H), 3.93 – 3.83 (m, 2H), 1.04 (s, 9H). ^{13}C NMR (150 MHz, $CDCl_3$) δ 166.0, 165.3, 165.1, 154.2, 151.2, 135.6, 135.5, 133.4, 133.3, 133.0, 132.9, 132.7, 132.6, 130.3, 129.84, 129.82, 129.7, 129.2, 129.1, 128.9, 128.6, 128.49, 128.45, 128.4, 127.8, 127.7, 121.6, 120.0, 118.1, 100.2, 85.9, 83.5, 77.2, 75.7, 73.3, 72.0, 69.1, 62.7, 26.7, 19.2. HRMS (ESI) calcd for $C_{58}H_{55}N_2O_{10}Si$ $[M+NH_4]^+$ 967.3620, found 967.3623.

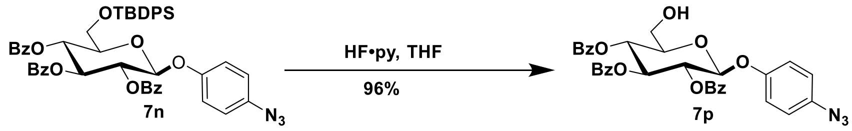

Fig. S27. Synthesis of the Compound **7p**.

Compound 7p

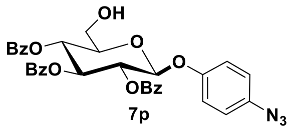

Similar procedure as that used for the synthesis of **7e** was applied to the preparation of **7p** (0.82 g, 96%) as a white foam: $[\alpha]^{20}_{D}$=24.1 (*c* 0.51, $CHCl_3$); ^{1}H NMR (600 MHz, Chloroform-*d*) δ 7.96 (d, *J* = 8.2 Hz, 4H), 7.90 – 7.83 (m, 2H), 7.57 – 7.48 (m, 2H), 7.44 (t, *J* = 7.5 Hz, 1H), 7.42 – 7.35 (m, 4H), 7.30 (t, *J* = 7.8 Hz, 2H), 7.01 (d, *J* = 9.0 Hz, 2H), 6.93 (d, *J* = 9.0 Hz, 2H), 6.01 (t, *J* = 9.6 Hz, 1H), 5.75 (dd, *J* = 9.8, 7.8 Hz, 1H), 5.58 (t, *J* = 9.7 Hz, 1H), 5.36 (d, *J* = 7.8 Hz, 1H), 4.00 – 3.86 (m, 2H), 3.84 – 3.75 (m, 1H), 2.49 (dd, *J* = 8.4, 5.6 Hz, 1H). ^{13}C NMR (150 MHz, $CDCl_3$) δ 166.1, 166.0, 165.2, 154.3, 135.5, 133.9, 133.51, 133.48, 130.1, 129.9, 129.3, 129.0, 128.74, 128.70, 128.6, 128.5, 120.3, 119.0, 100.3, 77.2, 75.3, 72.9, 72.0, 69.5, 61.6. HRMS (ESI) calcd for $C_{33}H_{31}N_4O_9$ $[M+NH_4]^+$ 627.2086, found 627.2081.

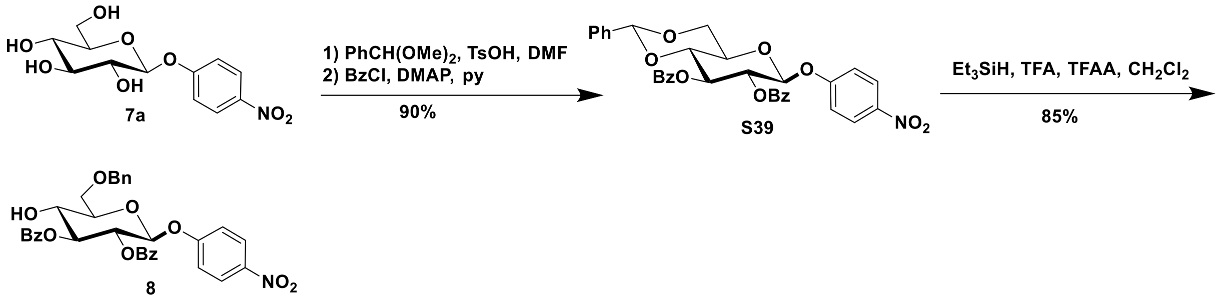

Fig. S28. Synthesis of the Compound **8**.

Compound S39

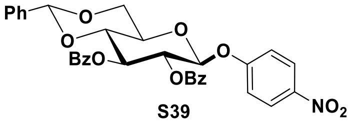

Similar procedure as that used for the synthesis of **7g** was applied to the preparation of **S39** (1.5 g, 90%) as a white foam: $[\alpha]_D^{25}$= 25.3 (*c* 0.26, $CHCl_3$); ^{1}H NMR (400 MHz, Chloroform-*d*) δ 8.18 (d, *J* = 9.0 Hz, 2H), 7.97 (dd, *J* = 12.6, 7.8 Hz, 4H), 7.52 (t, *J* = 7.5 Hz, 2H), 7.46 – 7.31 (m, 9H), 7.08 (d, *J* = 9.0 Hz, 2H), 5.90 (t, *J* = 9.3 Hz, 1H), 5.78 (t, *J* = 8.3 Hz, 1H), 5.60 (s, 1H), 5.54 (d, *J* = 7.4 Hz, 1H), 4.55 – 4.40 (m, 1H), 4.10 (t, *J* = 9.0 Hz, 1H), 4.02 – 3.86 (m, 2H). ^{13}C NMR (150 MHz, $CDCl_3$) δ 165.7, 165.2, 161.3, 143.4, 136.6, 133.7, 133.5, 130.0, 129.9, 129.33, 129.27, 128.9, 128.6, 128.5, 128.4, 126.3, 126.0, 116.9, 101.8, 99.2, 78.4, 77.2, 72.2, 71.9, 68.6, 67.2. HRMS (ESI) m/z calcd for $C_{33}H_{31}N_2O_{10}$ $[M+NH_4]^+$ 615.1973, found 615.1974.

Compound 8

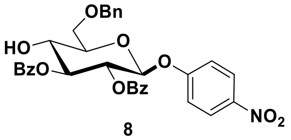

Similar procedure as that used for the synthesis of **7l** was applied to the preparation of **8** (1.2 g, 85%) as a white foam: $[\alpha]_D^{25}$= 49.1 (*c* 0.23, $CHCl_3$); ^{1}H NMR (600 MHz, Chloroform-*d*) δ 8.13 (d, *J* = 9.2 Hz, 2H), 8.02 – 7.97 (m, 2H), 7.97 – 7.92 (m, 2H), 7.57 – 7.47 (m, 2H), 7.43 – 7.28 (m, 9H), 7.08 (d, *J* = 9.2 Hz, 2H), 5.74 (dd, *J* = 9.7, 7.7 Hz, 1H), 5.57 (t, *J* = 9.4 Hz, 1H), 5.41 (d, *J* = 7.8 Hz, 1H), 4.66 – 4.56 (m, 2H), 4.07 (t, *J* = 9.2 Hz, 1H), 3.98 – 3.89 (m, 2H), 3.88 – 3.80 (m, 1H). ^{13}C NMR (150 MHz, $CDCl_3$) δ 167.3, 165.3, 161.6, 143.2, 137.7, 133.8, 133.6, 130.1, 129.9, 129.0, 128.9, 128.64, 128.62, 128.59, 128.1, 127.8, 125.9, 116.9, 98.7, 77.2, 76.7, 75.8, 73.9, 71.2, 70.4, 69.5. HRMS (ESI) m/z calcd for $C_{33}H_{29}NO_{10}Na$ $[M+Na]^+$ 622.1684, found 622.1683.

Synthesis of saccharides via latent-active strategies.

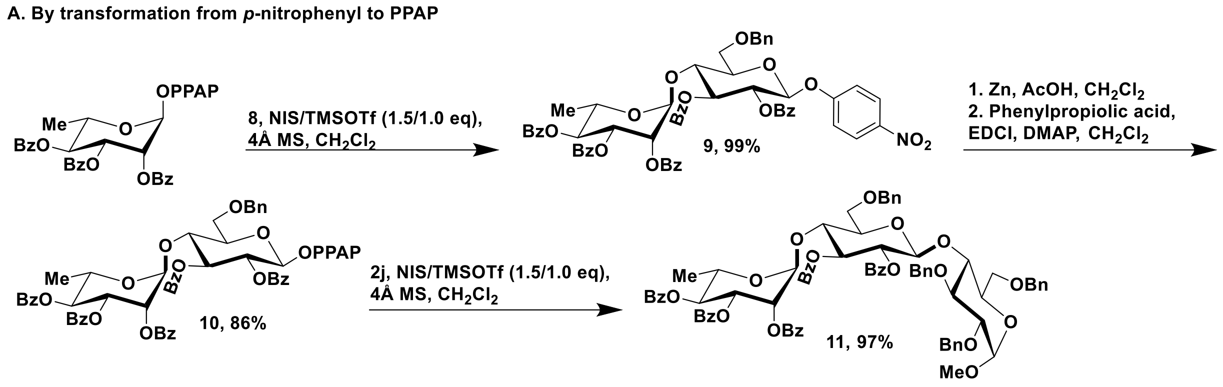

Fig. S29. Synthesis of trisaccharide **11**.

Compound 9

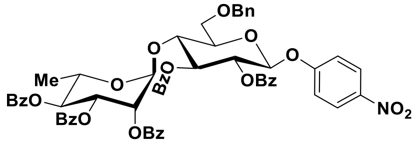

9

A solution of glycosyl PPAP donor **1n** (168.0 mg, 0.24 mmol) and acceptor **8** (120.0 mg, 0.2 mmol) in dry CH_2Cl_2 (0.033 M) was stirred at room temperature for 30 min in the presence of activated 4 Å MS (3.0 g/mmol) under N_2 atmosphere. Then the vessel was chilled to 0 °C, to which NIS (68.0 mg, 0.3 mmol), TMSOTf (400 μL, 0.5M in CH_2Cl_2) were added. The reaction mixture was stirred for 3 h after the temperature gradually rise to room temperature. Then Et_3N was added to quench the reaction and the solvent was removed under reduced pressure. The resulting residue was purified by silica gel column chromatography to afford the glycosylated product **9** (209.5 mg, 99%) as a white solid: $[\alpha]_D^{25}$=25.8 (*c* 0.66, $CHCl_3$); ^{1}H NMR (600 MHz, Chloroform-*d*) δ 8.16 (d, *J* = 9.2 Hz, 2H), 8.04 (ddd, *J* = 19.8, 8.4, 1.4 Hz, 4H), 7.95 (dd, *J* = 8.4, 1.4 Hz, 2H), 7.87 (ddd, *J* = 12.0, 8.4, 1.2 Hz, 4H), 7.63 – 7.57 (m, 1H), 7.56 – 7.41 (m, 7H), 7.42 – 7.34 (m, 4H), 7.34 – 7.24 (m, 6H), 7.25 – 7.16 (m, 3H), 7.10 (d, *J* = 9.2 Hz, 2H), 5.94 (t, *J* = 9.2 Hz, 1H), 5.77 – 5.66 (m, 2H), 5.63 (dd, *J* = 3.6, 1.8 Hz, 1H), 5.55 (t, *J* = 10.0 Hz, 1H), 5.48 (d, *J* = 7.8 Hz, 1H), 5.28 (d, *J* = 1.8 Hz, 1H), 4.69 (d, *J* = 12.0 Hz, 1H), 4.62 (d, *J* = 12.0 Hz, 1H), 4.51 (t, *J* = 9.2 Hz, 1H), 4.13 – 4.03 (m, 2H), 4.03 – 3.94 (m, 2H), 0.77 (d, *J* = 6.6 Hz, 3H). ^{13}C NMR (150 MHz, $CDCl_3$) δ 166.0, 165.8, 165.7, 165.2, 161.5, 143.2, 137.7, 133.7, 133.6, 133.5, 133.4, 133.3, 130.0, 129.91, 129.85, 129.8, 129.31, 129.26, 129.2, 129.0, 128.7, 128.6, 128.54, 128.49, 128.44, 128.40, 127.9, 127.8, 125.9, 117.0, 98.6, 77.2, 75.6, 75.4, 73.8, 73.5, 72.1, 71.4, 71.0, 69.9, 68.0, 67.7, 17.2. HRMS (ESI) m/z calcd for $C_{60}H_{55}N_2O_{17}$ $[M+NH_4]^+$ 1075.3495, found 1075.3490.

Compound 10

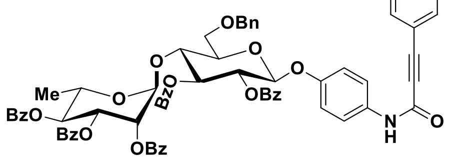

10

To a solution of compound **9** (180 mg, 0.17 mmol) in CH_2Cl_2 (3 mL) was added zinc powder (167 mg, 2.55 mmol) and acetic acid (0.48 mL, 8.5 mmol) were added sequentially at 0 °C. The reaction

mixture was warmed to room temperature gradually, and the stirring was continued for another 2 h. After the zinc powder was removed by filtration, the filtrate was washed successively with water, brine, and then dried over Na_2SO_4. Filtration was followed by concentration to give the crude product which was used to prepare of **10** (169 mg, 86% for four steps) according to **General procedure D**.$[\alpha]_D^{25}$=41.2 (*c* 0.72, $CHCl_3$); ^{1}H NMR (600 MHz, Chloroform-*d*) δ 8.03 (ddd, *J* = 19.8, 7.8, 1.2 Hz, 4H), 7.97 – 7.92 (m, 2H), 7.86 (ddd, *J* = 15.0, 8.4, 1.8 Hz, 4H), 7.72 (s, 1H), 7.63 – 7.56 (m, 1H), 7.57 – 7.31 (m, 20H), 7.32 – 7.25 (m, 4H), 7.23 (t, *J* = 7.4 Hz, 2H), 7.21 – 7.15 (m, 1H), 7.00 (d, J = 9.0 Hz, 1H), 5.89 (t, *J* = 9.4 Hz, 1H), 5.72 – 5.59 (m, 3H), 5.53 (t, *J* = 10.0 Hz, 1H), 5.31 – 5.25 (m, 2H), 4.73 – 4.62 (m, 2H), 4.48 (t, *J* = 9.4 Hz, 1H), 4.07 (dd, *J* = 11.4, 3.6 Hz, 1H), 4.01 – 3.90 (m, 3H), 0.74 (d, *J* = 6.6 Hz, 3H). ^{13}C NMR (150 MHz, $CDCl_3$) δ 166.1, 165.8, 165.71, 165.66, 165.4, 154.2, 151.0, 137.9, 133.6, 133.41, 133.38, 133.3, 132.9, 132.7, 130.4, 130.0, 129.9, 129.8, 129.43, 129.38, 129.34, 129.30, 128.7, 128.52, 128.49, 128.4, 127.9, 127.7, 121.6, 120.1, 118.3, 100.1, 98.5, 85.8, 83.5, 77.2, 75.6, 75.4, 74.0, 73.5, 72.4, 71.5, 71.0, 69.9, 68.1, 67.6, 17.2. HRMS (ESI) calcd for $C_{69}H_{61}N_2O_{16}$ $[M+NH_4]^+$ 1173.4016, found 1173.4011.

Compound 11

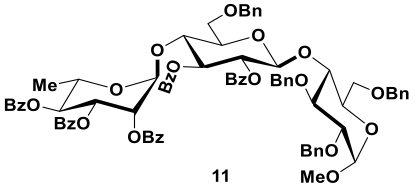

Glycosylation of **10** (138.0 mg, 0.12 mmol) with **2j** (26.0 mg, 0.10 mmol) according to **9** afforded **11** (134.0 mg, 97%) as a white solid: $[\alpha]_D^{25}$=39.6 (*c* 4.53, $CHCl_3$); ^{1}H NMR (600 MHz, Chloroform-*d*) δ 8.04 – 7.99 (m, 2H), 7.98 – 7.93 (m, 2H), 7.90 – 7.82 (m, 6H), 7.63 – 7.47 (m, 7H), 7.49 – 7.33 (m, 14H), 7.30 – 7.26 (m, 8H), 7.25 – 7.17 (m, 6H), 7.18 – 7.09 (m, 3H), 5.69 (dd, *J* = 10.2, 3.6 Hz, 1H), 5.64 – 5.55 (m, 2H), 5.50 (t, *J* = 10.0 Hz, 1H), 5.35 (dd, *J* = 9.8, 7.8 Hz, 1H), 5.19 (d, *J* = 1.8 Hz, 1H), 5.12 (d, *J* = 11.4 Hz, 1H), 4.80 (d, J = 11.8 Hz, 1H), 4.75 (d, *J* = 3.6 Hz, 1H), 4.73 (s, 1H), 4.70 (d, *J* = 12.0 Hz, 1H), 4.60 (d, *J* = 12.0 Hz, 1H), 4.55 (d, *J* = 3.6 Hz, 1H), 4.50 (d, *J* = 12.0 Hz, 1H), 4.41 (d, *J* = 12.0 Hz, 1H), 4.37 – 4.29 (m, 2H), 4.00 – 3.85 (m, 3H), 3.83 – 3.73 (m, 2H), 3.67 (dd, *J* = 10.8, 3.6 Hz, 1H), 3.54 – 3.47 (m, 2H), 3.45 (dd, *J* = 10.8, 2.0 Hz, 1H), 3.37 – 3.31 (m, 1H), 3.27 (s, 3H), 0.70 (d, *J* = 6.6 Hz, 3H). ^{13}C NMR (150 MHz, $CDCl_3$) δ 166.1, 165.7, 165.6, 165.1, 139.7, 138.5, 138.1, 137.9, 133.6, 133.4, 133.27, 133.25, 133.2, 130.0, 129.9, 129.8, 129.6, 129.43, 129.37, 129.0, 128.7, 128.6, 128.51, 128.47, 128.42, 128.36, 128.29, 128.27, 128.2, 127.9, 127.7, 127.6, 127.4, 127.2, 100.4, 98.5, 98.2, 80.4, 79.1, 77.2, 75.5, 75.4, 75.0, 74.3, 73.8, 73.7, 73.2, 73.1, 71.6, 71.0, 69.9, 69.6, 68.2, 68.0, 67.5, 55.4, 17.1. HRMS (ESI) calcd for $C_{82}H_{82}NO_{20}$ $[M+NH_4]^+$ 1400.5425, found 1400.5420.

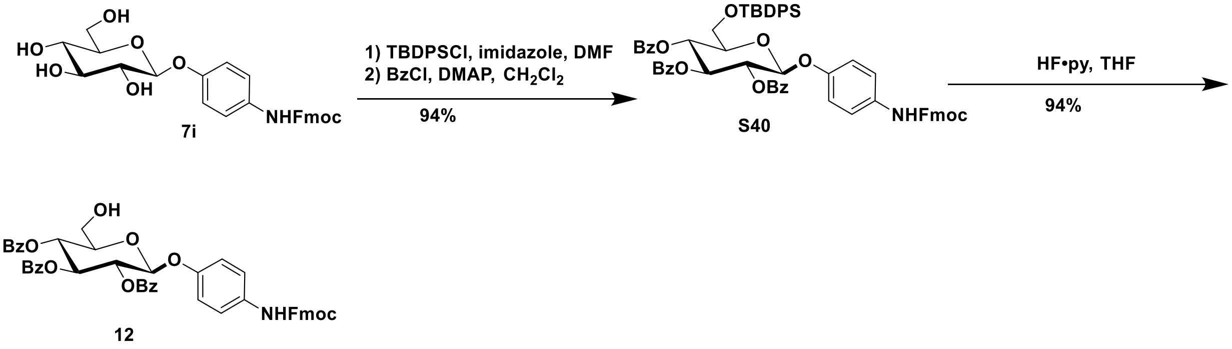

Fig. S30. Synthesis of compound **12**.

Compound S40

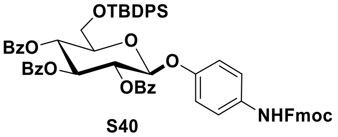

S40

Similar procedure as that used for the synthesis of **7n** was applied to get **S40** (2.0 g, 94%) as a white solid: $[\alpha]_D^{25}$= 22.7 (*c* 0.38, CH_2Cl_2); ^1H NMR (400 MHz, Chloroform-*d*) δ 7.99 (d, *J* = 7.6 Hz, 2H), 7.87 (d, *J* = 7.6 Hz, 4H), 7.78 (d, *J* = 7.6 Hz, 2H), 7.67 (d, *J* = 7.6 Hz, 2H), 7.63 – 7.58 (m, 4H), 7.57 – 7.50 (m, 2H), 7.47 – 7.28 (m, 14H), 7.25 – 7.19 (m, 5H), 7.03 (d, *J* = 8.5 Hz, 2H), 6.60 (s, 1H), 5.94 (t, *J* = 9.6 Hz, 1H), 5.77 (dd, *J* = 9.8, 7.8 Hz, 1H), 5.69 (t, *J* = 9.6 Hz, 1H), 5.31 (d, *J* = 7.8 Hz, 1H), 4.54 (d, *J* = 6.6 Hz, 2H), 4.27 (t, *J* = 6.6 Hz, 1H), 4.06 – 3.97 (m, 1H), 3.97 – 3.83 (m, 2H), 1.06 (s, 9H). ^{13}C NMR (100 MHz, $CDCl_3$) δ 166.0, 165.3, 165.2, 153.7, 143.9, 141.5, 135.7, 135.6, 133.4, 133.3, 133.1, 132.9, 129.94, 129.91, 129.83, 129.77, 129.4, 129.2, 129.0, 128.54, 128.50, 128.4, 127.9, 127.80, 127.78, 127.2, 125.1, 120.2, 118.4, 100.6, 77.4, 77.2, 75.8, 73.3, 72.1, 69.2, 66.9, 62.8, 47.3, 26.8, 19.3. HRMS (ESI) m/z calcd for $C_{64}H_{61}N_2O_{11}Si$ $[M+NH_4]^+$ 1061.4039, found 1061.4040.

Compound 12

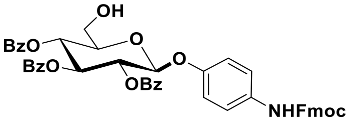

12

Similar procedure as that used for the synthesis of **7e** was applied to get **12** (0.8 g, 94%) as a white solid: $[\alpha]_D^{25}$= 25.0 (*c* 0.5, CH_2Cl_2); ^1H NMR (600 MHz, Chloroform-*d*) δ 7.96 (dd, *J* = 7.8, 1.2 Hz, 4H), 7.89 – 7.84 (m, 2H), 7.77 (d, *J* = 7.8 Hz, 2H), 7.60 (d, *J* = 7.2 Hz, 2H), 7.57 – 7.48 (m, 2H), 7.47 – 7.34 (m, 8H), 7.35 – 7.26 (m, 5H), 6.95 (d, *J* = 8.4 Hz, 2H), 6.68 (s, 1H), 6.01 (t, *J* = 9.6 Hz, 1H), 5.76 (dd, *J* = 10.2, 7.8 Hz, 1H), 5.58 (t, *J* = 9.6 Hz, 1H), 5.34 (d, *J* = 7.8 Hz, 1H), 4.52 (d, *J* = 6.6 Hz, 2H), 4.25 (t, *J* = 6.6 Hz, 1H), 4.00 – 3.86 (m, 2H), 3.78 (dd, *J* = 12.8, 4.8 Hz, 1H), 2.62 (s, 1H). ^{13}C NMR (100 MHz, $CDCl_3$) δ 166.1, 166.0, 165.2, 153.7, 153.3, 143.9, 141.5, 133.9, 133.49, 133.46, 130.1, 129.9, 129.2, 128.9, 128.7, 128.6, 128.5, 127.9, 127.3, 125.1, 120.2, 118.1, 100.3, 77.4, 77.2, 75.1, 72.8, 71.9, 69.5, 67.0, 61.5, 47.3. HRMS (ESI) calcd for $C_{48}H_{43}N_2O_{11}$ $[M+NH_4]^+$ 823.2861, found 823.2866.

B. By transformation from *p*-NHFmoc phenyl to PPAP

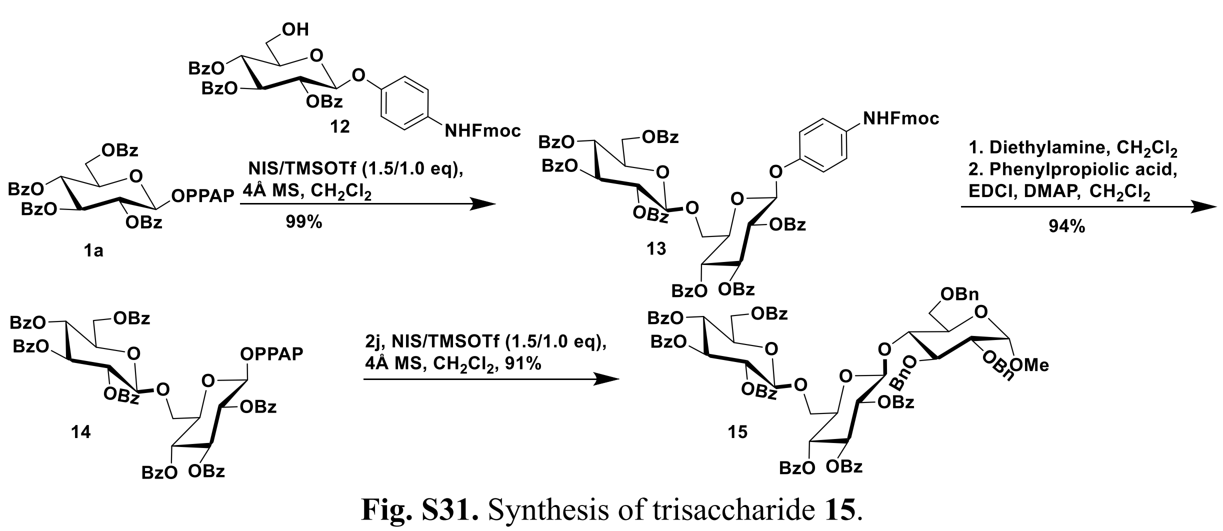

Fig. S31. Synthesis of trisaccharide **15**.

Compound 13

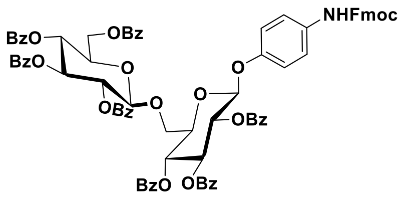

13

Glycosylation of **1a** (196.0 mg, 0.24 mmol) with **12** (161.0 mg, 0.20 mmol) according to **9** afforded **13** (274.0 mg, 99%) as a white solid: $[\alpha]_D^{25}$= 5.7 (*c* 0.31, $CHCl_3$); ^{1}H NMR (600 MHz, Chloroform-*d*) δ 8.10 – 8.04 (m, 2H), 7.96 (d, *J* = 7.6 Hz, 2H), 7.93 – 7.79 (m, 10H), 7.72 (t, *J* = 6.6 Hz, 2H), 7.62 – 7.55 (m, 2H), 7.54 – 7.47 (m, 8H), 7.46 – 7.42 (m, 3H), 7.41 – 7.32 (m, 9H), 7.31 – 7.24 (m, 8H), 7.19 – 7.13 (m, 1H), 7.10 – 7.01 (m, 3H), 5.88 (t, *J* = 9.6 Hz, 1H), 5.73 (t, *J* = 9.6 Hz, 1H), 5.63 (dd, *J* = 9.8, 7.8 Hz, 1H), 5.57 (t, *J* = 9.6 Hz, 1H), 5.47 (dd, *J* = 9.6, 7.8 Hz, 1H), 5.36 (d, *J* = 9.0 Hz, 1H), 5.29 (t, *J* = 9.6 Hz, 1H), 5.06 (d, *J* = 8.0 Hz, 1H), 4.56 (dd, *J* = 12.0, 3.0 Hz, 1H), 4.48 – 4.37 (m, 2H), 4.29 (s, 1H), 4.14 (t, *J* = 7.2 Hz, 1H), 4.11 – 4.06 (m, 2H), 3.92 – 3.84 (m, 1H), 3.77 – 3.71 (m, 1H). ^{13}C NMR (151 MHz, $CDCl_3$) δ 166.5, 166.2, 165.8, 165.4, 165.3, 165.2, 143.9, 143.8, 141.4, 141.3, 133.72, 133.66, 133.5, 133.43, 133.37, 130.01, 129.96, 129.9, 129.8, 129.77, 129.7, 129.3, 129.2, 128.8, 128.7, 128.63, 128.61, 128.58, 128.54, 128.52, 128.4, 128.3, 127.80, 127.76, 127.2, 125.2, 125.1, 121.1, 120.03, 119.98, 118.2, 100.1, 98.9, 77.2, 76.1, 73.4, 72.7, 72.4, 72.0, 71.7, 69.6, 69.4, 67.1, 66.5, 62.9, 47.1. HRMS (ESI) calcd for $C_{82}H_{65}NO_{20}Na$ $[M+Na]^+$ 1406.3992, found 1406.3993.

Compound 14

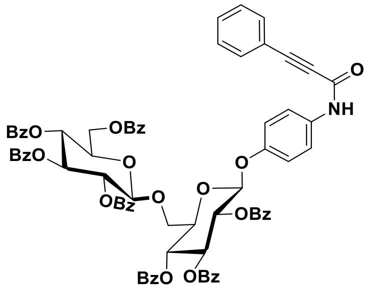

14

Similar procedure as that used for the synthesis of **7k** was applied to the preparation of **14** (182 mg, 94%) as a white foam: $[\alpha]_D^{25}$=28.3 (*c* 0.34, $CHCl_3$); ^{1}H NMR (600 MHz, Chloroform-*d*) δ 8.65 (s, 1H), 8.08 (dd, *J* = 8.4, 1.8 Hz, 2H), 7.96 (dd, *J* = 8.4, 1.2 Hz, 2H), 7.91 – 7.75 (m, 12H), 7.64 – 7.56 (m, 2H), 7.54 – 7.47 (m, 4H), 7.47 – 7.40 (m, 4H), 7.43 – 7.32 (m, 10H), 7.31 – 7.28 (m, 2H), 7.27 (s, 1H), 7.26 – 7.23 (m, 1H), 7.17 – 7.10 (m, 4H), 5.88 (t, *J* = 9.6 Hz, 1H), 5.69 (t, *J* = 9.7 Hz, 1H), 5.63 (dd, *J* = 9.8, 7.8 Hz, 1H), 5.56 (t, *J* = 9.7 Hz, 1H), 5.48 – 5.41 (m, 2H), 5.26 (t, *J* = 9.7 Hz, 1H), 5.06 (d, *J* = 8.0 Hz, 1H), 4.55 (dd, *J* = 12.1, 3.0 Hz, 1H), 4.42 (dd, *J* = 12.1, 5.4 Hz, 1H), 4.15 – 4.03 (m, 2H), 3.82 (dd, *J* = 12.9, 1.1 Hz, 1H), 3.67 – 3.60 (m, 1H). ^{13}C NMR (151 MHz, $CDCl_3$) δ 166.5, 166.1, 165.7, 165.4, 165.2, 165.1, 143.8, 143.7, 141.3, 141.2, 133.64, 133.58, 133.43, 133.35, 133.3, 129.93, 129.88, 129.85, 129.8, 129.7, 129.6, 129.2, 129.1, 128.7, 128.57, 128.55, 128.53, 128.50, 128.46, 128.44, 128.37, 128.2, 127.72, 127.68, 127.1, 125.1, 125.0, 121.0, 119.94, 119.90, 118.1, 100.0, 98.9, 76.0, 73.3, 72.6, 72.3, 71.9, 71.6, 69.5, 69.3, 67.0, 66.4, 62.8, 47.0. HRMS (ESI) calcd for $C_{76}H_{63}N_2O_{19}$ $[M+NH_4]^+$ 1307.4020, found 1307.4019.

Compound 15(*31*)

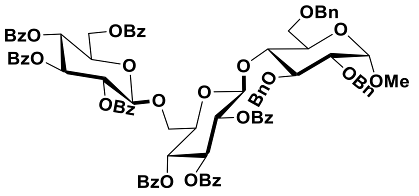

15

Glycosylation of **14** (154.7 mg, 0.12 mmol) with **2j** (46.4 mg, 0.10 mmol) according to **9** afforded **15** (138.0 mg, 91%) as a white solid: $[\alpha]_D^{20}$=99.3 (*c* 0.81, $CHCl_3$); ^{1}H NMR (600 MHz, Chloroform-*d*) δ 8.04 – 7.98 (m, 4H), 7.95 – 7.90 (m, 2H), 7.85 – 7.80 (m, 2H), 7.82 – 7.77 (m, 2H), 7.75 (ddd, *J* = 12.6, 8.4, 1.8 Hz, 4H), 7.66 – 7.62 (m, 2H), 7.58 – 7.50 (m, 2H), 7.52 – 7.46 (m, 1H), 7.49 – 7.32 (m, 20H), 7.32 – 7.27 (m, 6H), 7.22 (t, *J* = 7.8 Hz, 2H), 7.19 – 7.13 (m, 4H), 5.83 (t, *J* = 9.8 Hz, 1H), 5.56 (t, *J* = 9.6 Hz, 1H), 5.51 (t, *J* = 9.6 Hz, 1H), 5.45 – 5.36 (m, 2H), 5.32 (t, *J* = 9.8 Hz, 1H), 5.25 (d, *J* = 7.8 Hz, 1H), 5.16 (d, *J* = 11.7 Hz, 1H), 5.11 (d, *J* = 11.6 Hz, 1H), 4.93 (d, *J* = 11.7 Hz, 1H), 4.88 (d, *J* = 11.7 Hz, 1H), 4.74 (d, *J* = 3.5 Hz, 1H), 4.69 (d, *J* = 8.1 Hz, 1H), 4.18 (dd, *J* = 12.1, 3.9 Hz, 2H), 4.04 – 3.83 (m, 7H), 3.73 – 3.66 (m, 1H), 3.62 – 3.56 (m, 1H), 3.51 (dd, *J* = 10.5, 4.0 Hz, 1H), 3.35 (dd, *J* = 10.5, 1.9 Hz, 1H), 3.32 (s, 3H), 3.30 – 3.26 (m, 1H). ^{13}C NMR (150 MHz, $CDCl_3$) δ 166.2, 165.78, 165.76, 165.7, 165.4, 165.1, 164.8, 139.9, 139.1, 137.9, 133.6, 133.4, 133.33, 133.28, 133.2, 133.14, 133.11, 130.3, 130.0, 129.9, 129.83, 129.76, 129.3, 129.2, 129.04, 129.00, 128.95, 128.9, 128.7, 128.6, 128.51, 128.48, 128.42, 128.40, 128.35, 128.3, 128.2, 127.84, 127.75, 100.2, 100.0, 98.5, 81.6, 80.3, 77.2, 76.6, 74.8, 73.9, 73.3, 73.2, 73.1, 72.5, 72.4, 71.3, 70.2, 69.7, 69.4, 68.0, 66.8, 62.8, 55.4.

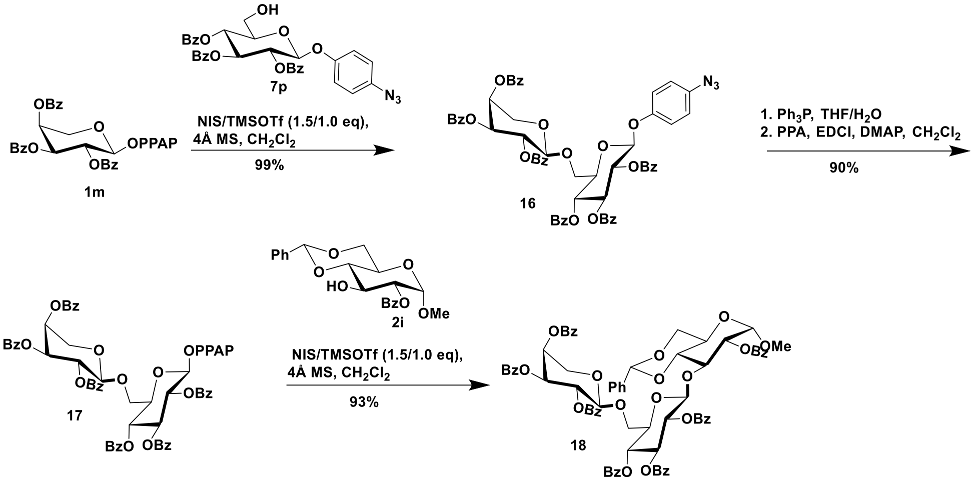

Fig. S32. Synthesis of trisaccharide **18**.

Compound 16

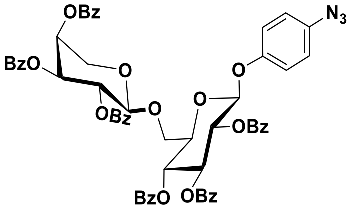

16

Glycosylation of **1m** (164.0 mg, 0.24 mmol) with **7p** (122.0 mg, 0.20 mmol) according to **9** afforded **16** (208.7 mg, 99%) as a white solid: $[\alpha]_D^{20}$= 79.8 (*c* 2.84, $CHCl_3$); ^{1}H NMR (600 MHz, Chloroform-*d*) δ 8.03 (dd, *J* = 7.8, 1.2 Hz, 2H), 7.96 (d, *J* = 7.2 Hz, 2H), 7.94 – 7.89 (m, 6H), 7.81 (dd, *J* = 8.4, 1.4 Hz, 2H), 7.60 – 7.54 (m, 1H), 7.54 – 7.45 (m, 4H), 7.47 – 7.40 (m, 3H), 7.39 – 7.30 (m, 8H),

7.31 – 7.26 (m, 2H), 6.92 (d, *J* = 9.0 Hz, 2H), 6.82 (d, *J* = 9.0 Hz, 2H), 5.90 (t, *J* = 9.6 Hz, 1H), 5.77 – 5.70 (m, 1H), 5.70 – 5.62 (m, 2H), 5.57 (dd, *J* = 8.4, 3.5 Hz, 1H), 5.50 (t, *J* = 9.7 Hz, 1H), 5.20 (d, *J* = 7.8 Hz, 1H), 4.84 (d, *J* = 6.0 Hz, 1H), 4.25 (dd, *J* = 12.8, 4.4 Hz, 1H), 4.22 – 4.15 (m, 1H), 4.12 (dd, *J* = 11.4, 2.0 Hz, 1H), 3.90 (dd, *J* = 11.4, 7.2 Hz, 1H), 3.83 (dd, *J* = 12.6, 2.4 Hz, 1H). ^{13}C NMR (150 MHz, $CDCl_3$) δ 165.9, 165.8, 165.7, 165.5, 165.4, 165.2, 154.4, 135.2, 133.7, 133.6, 133.5, 133.44, 133.39, 130.0, 129.91, 129.86, 129.5, 129.4, 129.2, 128.84, 128.78, 128.64, 128.61, 128.59, 128.56, 128.5, 128.4, 120.2, 118.8, 100.7, 100.4, 77.2, 74.4, 72.8, 71.8, 70.6, 69.8, 69.5, 68.4, 68.1. HRMS (ESI) calcd for $C_{59}H_{51}N_4O_{16}$ $[M+NH_4]^+$ 1071.3295, found 1071.3298.

Compound 17

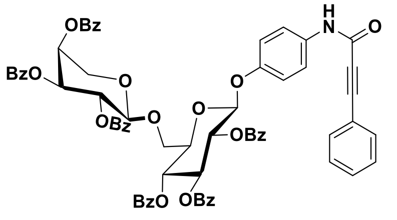

17

Similar procedure as that used for the synthesis of **7o** was applied to the preparation of **17** (173.4 mg, 90%) as a white foam: $[\alpha]_D^{25}$= 96.2 (*c* 0.12, $CHCl_3$); ^{1}H NMR (600 MHz, Chloroform-*d*) δ 8.07 – 8.02 (m, 2H), 7.98 (s, 1H), 7.97 – 7.92 (m, 2H), 7.94 – 7.87 (m, 6H), 7.84 – 7.78 (m, 2H), 7.61 – 7.48 (m, 8H), 7.49 – 7.40 (m, 5H), 7.43 – 7.33 (m, 8H), 7.28 (t, *J* = 7.8 Hz, 2H), 7.24 (t, *J* = 7.8 Hz, 2H), 7.02 (d, *J* = 9.0 Hz, 2H), 5.90 (t, *J* = 9.6 Hz, 1H), 5.74 – 5.67 (m, 2H), 5.64 – 5.59 (m, 1H), 5.53 (dd, *J* = 9.0, 3.5 Hz, 1H), 5.45 (t, *J* = 9.7 Hz, 1H), 5.28 (d, *J* = 7.8 Hz, 1H), 4.91 (d, *J* = 6.5 Hz, 1H), 4.26 (dd, *J* = 12.9, 3.8 Hz, 1H), 4.10 (d, *J* = 9.8, 7.4, 2.0 Hz, 1H), 4.06 – 3.95 (m, 2H), 3.73 (dd, *J* = 13.0, 2.1 Hz, 1H). ^{13}C NMR (150 MHz, $CDCl_3$) δ 166.1, 165.9, 165.8, 165.50, 165.47, 165.2, 153.6, 151.0, 133.74, 133.69, 133.61, 133.58, 133.53, 133.46, 133.2, 132.7, 130.4, 130.04, 130.01, 129.93, 129.90, 129.8, 129.5, 129.4, 129.2, 128.9, 128.8, 128.74, 128.68, 128.61, 128.58, 128.56, 128.5, 122.1, 120.2, 118.3, 100.6, 99.8, 85.6, 83.7, 77.2, 75.6, 72.8, 71.7, 70.7, 70.0, 69.4, 68.7, 67.1. HRMS (ESI) calcd for $C_{68}H_{57}N_2O_{17}$ $[M+NH_4]^+$ 1173.3652, found 1173.3656.

Compound 18

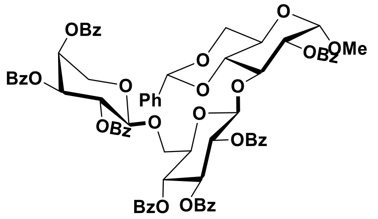

18

Glycosylation of **17** (138.0 mg, 0.12 mmol) with **2i** (38.6 mg, 0.10 mmol) according to **9** afforded **18** (121.4 mg, 93%) as a white solid: $[\alpha]_D^{25}$= 39.0 (*c* 3.8, $CHCl_3$); ^{1}H NMR (600 MHz, Chloroform-*d*) δ 8.19 – 8.15 (m, 2H), 8.02 – 7.97 (m, 2H), 7.94 – 7.89 (m, 2H), 7.89 – 7.82 (m, 4H), 7.81 – 7.75 (m, 2H), 7.71 – 7.66 (m, 2H), 7.57 – 7.31 (m, 17H), 7.31 – 7.26 (m, 5H), 7.26 – 7.23 (m, 3H), 7.09 – 7.02 (m, 2H), 5.77 (t, *J* = 9.6 Hz, 1H), 5.72 (dd, *J* = 9.8, 7.3 Hz, 1H), 5.66 (s, 1H), 5.60 (dd, *J* = 9.8, 7.8 Hz, 1H), 5.51 – 5.40 (m, 3H), 5.14 (dd, *J* = 11.9, 7.5 Hz, 2H), 4.60 (d, *J* = 12.4 Hz, 1H), 4.48 – 4.39 (m, 3H), 4.24 (d, *J* = 12.5 Hz, 1H), 4.08 – 3.93 (m, 2H), 3.90 – 3.80 (m, 2H), 3.78 (t, *J* = 10.2 Hz, 1H), 3.65 (t, *J* = 9.6 Hz, 1H), 3.46 – 3.37 (m, 2H), 3.14 (s, 3H), 3.14 – 3.06 (m, 1H). ^{13}C NMR (150 MHz, $CDCl_3$) δ 165.9, 165.9, 165.8, 165.5, 165.4, 165.3, 138.2, 137.8, 133.6, 133.34,

133.31, 133.26, 130.25, 130.0, 129.9, 129.84, 129.82, 129.7, 129.4, 129.34, 129.28, 129.1, 129.0, 128.82, 128.79, 128.54, 128.51, 128.46, 128.43, 128.39, 128.0, 127.7, 126.2, 101.1, 100.8, 100.7, 99.2, 80.5, 80.0, 77.2, 76.7, 75.8, 74.2, 73.1, 72.5, 71.1, 69.9, 69.6, 69.2, 69.1, 67.1, 62.9, 55.4. HRMS (ESI) calcd for $C_{74}H_{64}O_{22}Na$ $[M+Na]^+$ 1327.3781, found 1327.3780.

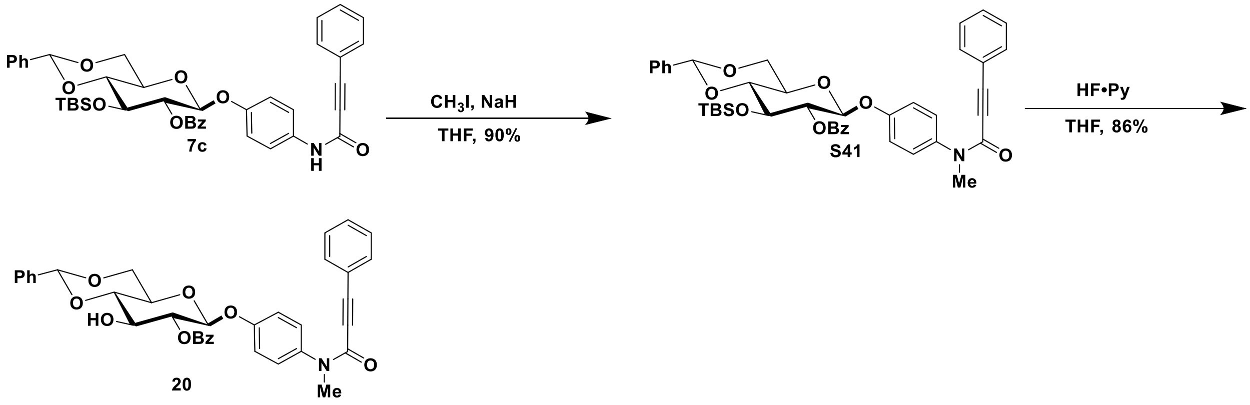

Fig. S33. Synthesis of compound **20**.

Compound S41

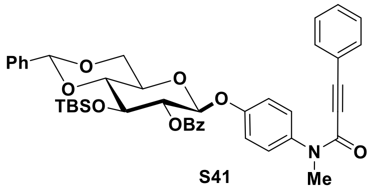

Similar procedure as that used for the synthesis of **1c** was applied to the preparation of **S41** (1.2 g, 90%) as a white foam: $[\alpha]_D^{20}$= 5.1 (*c* 1.02, $CHCl_3$); ^{1}H NMR (600 MHz, Chloroform-*d*) δ 8.16 (dd, *J* = 8.1, 1.5 Hz, 2H), 7.70 – 7.63 (m, 1H), 7.61 (dd, *J* = 7.7, 2.1 Hz, 2H), 7.56 – 7.49 (m, 2H), 7.51 – 7.44 (m, 3H), 7.42 – 7.36 (m, 1H), 7.34 – 7.29 (m, 2H), 7.27 (t, *J* = 7.8 Hz, 2H), 7.24 – 7.19 (m, 2H), 7.14 – 7.09 (m, 2H), 5.68 (s, 1H), 5.64 (t, *J* = 8.4 Hz, 1H), 5.35 (d, *J* = 8.0 Hz, 1H), 4.50 (dd, *J* = 10.5, 4.9 Hz, 1H), 4.25 (t, *J* = 8.9 Hz, 1H), 3.97 (t, *J* = 10.3 Hz, 1H), 3.85 (t, *J* = 9.2 Hz, 1H), 3.81 – 3.73 (m, 1H), 3.40 (s, 3H), 0.82 (s, 9H), 0.09 (s, 3H), 0.00 (s, 3H). ^{13}C NMR (150 MHz, $CDCl_3$) δ 165.2, 156.5, 154.4, 138.4, 137.0, 133.3, 132.50, 132.47, 130.0, 129.9, 129.8, 129.2, 128.7, 128.5, 128.4, 128.3, 126.3, 120.4, 117.7, 102.0, 100.3, 91.1, 82.6, 81.3, 77.2, 74.8, 73.0, 68.7, 66.8, 36.5, 25.6, 18.0, -4.1, -4.9. HRMS (ESI) calcd for $C_{42}H_{46}NO_8Si$ $[M+H]^+$ 720.2987, found 720.2983.

Compound 20

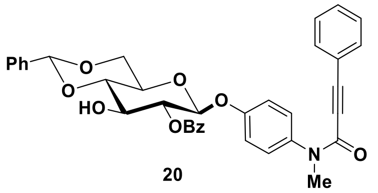

Similar procedure as that used for the synthesis of **7e** was applied to the preparation of **20** (0.6 g, 86%) as a white solid: $[\alpha]_D^{25}$= -1.2 (*c* 1.1, $CHCl_3$); ^{1}H NMR (600 MHz, Chloroform-*d*) δ 8.07 (d, *J* = 7.7 Hz, 1H), 7.58 (t, *J* = 7.4 Hz, 2H), 7.54 – 7.49 (m, 2H), 7.47 – 7.36 (m, 6H), 7.31 (t, *J* = 7.6 Hz, 1H), 77.26 – 7.22 (m, 2H), 7.19 (t, *J* = 7.7 Hz, 2H), 7.13 (d, *J* = 7.7 Hz, 2H), 7.04 (d, *J* = 8.7 Hz, 2H), 5.62 (s, 1H), 5.50 (t, *J* = 8.5 Hz, 1H), 5.30 (d, *J* = 7.8 Hz, 1H), 4.43 (dd, *J* = 10.6, 5.0 Hz, 1H), 4.17 (t, *J* = 9.2 Hz, 1H), 3.89 (t, *J* = 10.3 Hz, 1H), 3.81 (t, *J* = 9.4 Hz, 1H), 3.70 (td, *J* = 9.7, 4.9 Hz, 1H), 3.32 (s, 3H), 2.88 (s, 1H). ^{13}C NMR (150 MHz, $CDCl_3$) δ 165.9, 156.4, 154.5, 138.6,

136.9, 133.5, 132.5, 130.04, 129.97, 129.6, 129.4, 128.8, 128.6, 128.44, 128.40, 126.4, 120.5, 117.8, 102.1, 100.1, 91.2, 82.7, 80.7, 77.2, 74.7, 72.4, 68.6, 66.7, 36.5. HRMS (ESI) calcd for $C_{36}H_{32}NO_8$ $[M+H]^+$ 606.2122, found 606.2122.

Synthesis of saccharides via various one-pot strategies

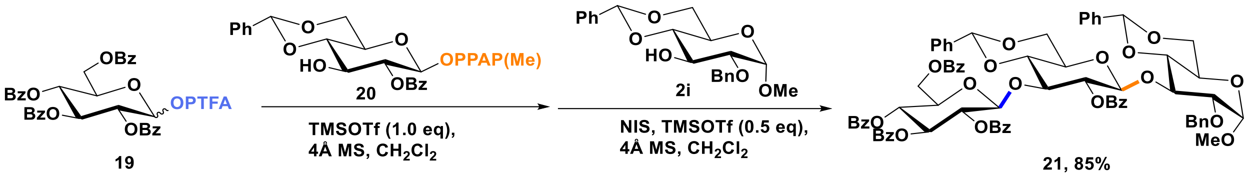

Fig. S34. One-pot synthesis of trisaccharide **21**.

A solution of glycosyl donor **19**(*14*) (74.0 mg, 0.096 mmol) and acceptor **20** (39.0 mg, 0.064mmol) in dry CH_2Cl_2 (1.5 mL) was stirred at room temperature for 30 min in the presence of activated 4Å MS (150 mg) under N_2 atmosphere. Then the vessel was chilled to 0 °C, to which TMSOTf (320 μL, 0.032 mmol) were added. The reaction mixture was stirred for 1 h at room temperature. Acceptor **2i** (19.8 mg, 0.053 mmol) was added to the reaction mixture, to which NIS (18.0 mg, 0.08 mol) and TMSOTf (53.0 μL, 0.027 mmol) were added at 0 ℃. The reaction mixture was warmed gradually to room temperature and stirred for 3h. Et_3N was added to quench the reaction and the solvent was removed under reduced pressure. The resulting residue was purified by silica gel column chromatography (Petroleum ether/EtOAc = 3:1 to 2:1) to afford trisaccharide **21** (58.8 mg, 85%) as a white solid: $[\alpha]_D^{25}$= 27.2 (*c* 1.3, CH_2Cl_2); ^{1}H NMR (600 MHz, Chloroform-*d*) δ 7.94 – 7.84 (m, 8H), 7.79 (dd, *J* = 8.1, 1.3 Hz, 2H), 7.58 – 7.50 (m, 3H), 7.51 – 7.36 (m, 5H), 7.37 – 7.17 (m, 21H), 7.14 (t, *J* = 7.5 Hz, 1H), 7.12 – 7.07 (m, 2H), 5.93 (t, *J* = 9.6 Hz, 1H), 5.70 (t, *J* = 9.7 Hz, 1H), 5.64 (dd, *J* = 9.7, 7.9 Hz, 1H), 5.49 (s, 1H), 5.38 (d, *J* = 7.9 Hz, 1H), 5.20 (d, *J* = 4.1 Hz, 1H), 5.14 (t, *J* = 3.7 Hz, 1H), 4.61 (s, 1H), 4.56 (dd, *J* = 12.0, 3.4 Hz, 1H), 4.41 (dd, *J* = 12.1, 4.8 Hz, 1H), 4.31 (d, *J* = 3.6 Hz, 1H), 4.28 – 4.15 (m, 4H), 4.17 – 4.06 (m, 4H), 3.71 (td, *J* = 10.0, 4.8 Hz, 1H), 3.63 – 3.56 (m, 2H), 3.50 (t, *J* = 10.1 Hz, 1H), 3.27 (s, 3H), 3.15 (t, *J* = 9.5 Hz, 1H), 2.79 (dd, *J* = 9.2, 3.7 Hz, 1H). ^{13}C NMR (150 MHz, $CDCl_3$) δ 166.2, 165.9, 165.3, 165.2, 164.8, 138.0, 137.52, 137.48, 133.5, 133.3, 133.1, 133.0, 130.1, 129.94, 129.90, 129.84, 129.81, 129.70, 129.66, 129.4, 129.3, 129.1, 129.0, 128.7, 128.6, 128.52, 128.49, 128.44, 128.38, 128.3, 128.1, 128.0, 127.9, 126.6, 126.1, 102.3, 100.4, 98.9, 98.4, 98.2, 81.0, 79.6, 77.9, 77.7, 77.2, 73.3, 73.2, 72.7, 72.1, 70.1, 69.1, 69.0, 65.2, 63.1, 62.5, 55.3. HRMS (ESI) calcd for $C_{75}H_{72}NO_{21}$ $[M+NH_4]^+$ 1322.4591, found 1322.4598.

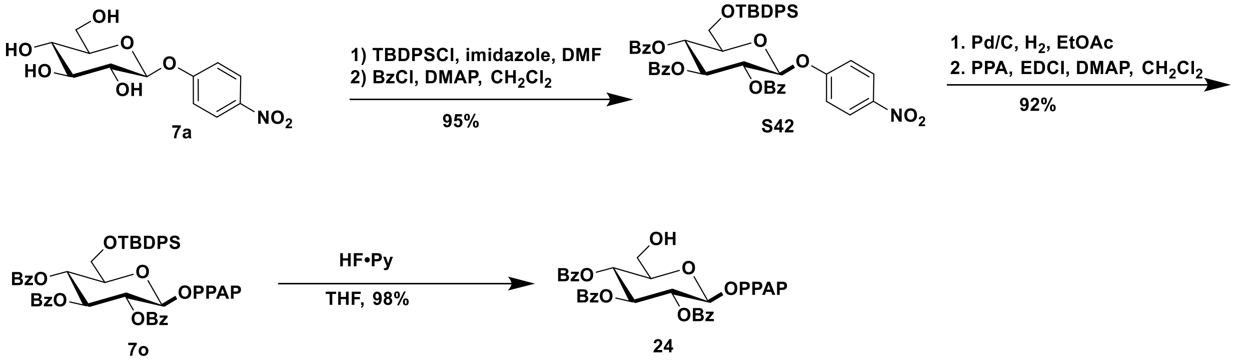

Fig. S35. Synthesis of compound **24**.

Compound S42

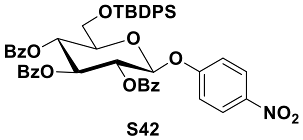

Similar procedure as that used for the synthesis of **7n** was applied to the preparation of **S42** (2.3 g, 95% for two steps) as a white solid: $[\alpha]_D^{25}$= 15.6 (*c* 1.3, CH_2Cl_2); ^{1}H NMR (600 MHz, Chloroform-*d*) δ 8.11 (d, *J* = 9.3 Hz, 2H), 7.99 – 7.94 (m, 2H), 7.87 (td, *J* = 8.1, 1.4 Hz, 4H), 7.65 – 7.60 (m, 2H), 7.60 – 7.55 (m, 2H), 7.53 (dd, *J* = 15.7, 7.5 Hz, 2H), 7.48 – 7.42 (m, 1H), 7.42 – 7.28 (m, 8H), 7.22 (dt, *J* = 15.0, 7.5 Hz, 4H), 7.15 (d, *J* = 9.3 Hz, 2H), 5.99 (t, *J* = 9.5 Hz, 1H), 5.82 (dd, *J* = 9.6, 7.6 Hz, 1H), 5.68 (t, *J* = 9.7 Hz, 1H), 5.52 (d, *J* = 7.7 Hz, 1H), 4.17 – 4.10 (m, 1H), 3.98 – 3.86 (m, 2H), 1.06 (s, 9H). ^{13}C NMR (150 MHz, $CDCl_3$) δ 165.9, 165.2, 165.1, 161.6, 143.3, 135.7, 135.5, 133.62, 133.58, 133.5, 133.0, 132.6, 129.93, 129.91, 129.03, 128.98, 128.8, 128.60, 128.56, 128.5, 127.81, 127.79, 125.9, 117.1, 98.9, 77.2, 76.3, 73.0, 71.8, 69.0, 62.8, 26.8, 19.3. HRMS (ESI) calcd for $C_{49}H_{49}N_2O_{11}Si$ $[M+NH_4]^+$ 869.3100, found 869.3105.

Compound 24

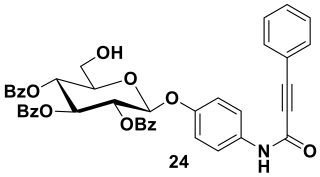

Similar procedure as that used for the synthesis of **7e** was applied to get **24** (0.8 g, 98%) as a white solid: $[\alpha]_D^{20}$= 43.7 (*c* 1.63, $CHCl_3$); ^{1}H NMR (600 MHz, Chloroform-*d*) δ 8.04 (s, 1H), 7.94 (d, *J* = 7.8 Hz, 4H), 7.84 (d, *J* = 7.7 Hz, 2H), 7.55 – 7.44 (m, 6H), 7.44 – 7.24 (m, 10H), 6.96 (d, *J* = 8.6 Hz, 2H), 6.00 (t, *J* = 9.7 Hz, 1H), 5.75 (dd, *J* = 9.8, 7.8 Hz, 1H), 5.56 (t, *J* = 9.7 Hz, 1H), 5.35 (d, *J* = 7.9 Hz, 1H), 3.96 – 3.86 (m, 2H), 3.78 (dd, *J* = 12.8, 4.8 Hz, 1H), 2.86 (s, 1H). ^{13}C NMR (150 MHz, $CDCl_3$) δ 166.1, 165.9, 165.3, 154.0, 151.2, 133.9, 133.51, 133.45, 133.1, 132.7, 130.4, 130.1, 129.90, 129.88, 129.1, 128.9, 128.7, 128.61, 128.57, 128.5, 121.8, 120.1, 117.9, 100.0, 85.9, 83.5, 77.2, 75.1, 72.8, 71.9, 69.5, 61.4. HRMS (ESI) calcd for $C_{42}H_{34}NO_{10}$ $[M+H]^+$ 712.2177, found 712.2175.

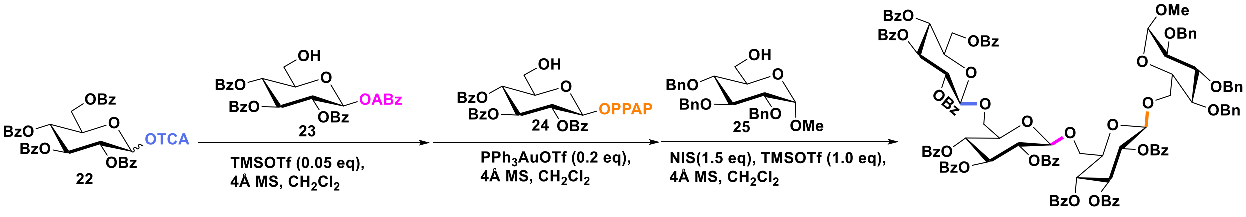

Fig. S36. One-pot synthesis of tetrasaccharide **26**.

A suspension of glucosyl trichloroacetimidate **22**(*13*) (66.6 mg, 0.09 mmol), glucosyl ABz acceptor **23**(*23*) (40.5 mg, 0.06 mmol), and activated 4Å MS (200 mg) in dry CH_2Cl_2 (2.0 mL) was stirred at room temperature for 30 min and was then cooled to 0 °C. A solution of TMSOTf in CH_2Cl_2 (60.0 μL, 0.05 M in CH_2Cl_2) was added to the mixture dropwise. After being stirred at room temperature for another 0.5 h, the reaction mixture was warmed to room temperature, to which glucosyl acceptor **24** (38.4 mg, 0.054 mmol) and a freshly prepared solution of PPh_3AuOTf in CH_2Cl_2 (0.22 mL, 0.05 M) were added successively. The resulting mixture was stirred at room temperature for another 1.5 h, then acceptor **25** (21 mg, 0.045 mmol), NIS (16 mg, 0.0675 mmol)

and TMSOTf (0.45 mL, 0.05 M, 0.025 mmol) were added successively at 0 °C. The resulting mixture was stirred for 3 h, then was quenched with Et_3N, filtered with Celite and concentrated in vacuo. The residue was purified by flash column chromatography (Petroleum ether/EtOAc = 2:1) to give **26***17* (64.5 mg, 72 %) as a white foam: ^{1}H NMR (600 MHz, Chloroform-*d*) δ 8.03 – 7.98 (m, 4H), 7.98 – 7.89 (m, 4H), 7.90 – 7.84 (m, 6H), 7.83 – 7.76 (m, 4H), 7.76 – 7.71 (m, 2H), 7.56 – 7.46 (m, 4H), 7.47 – 7.27 (m, 29H), 7.26 – 7.22 (m, 4H), 7.20 – 7.13 (m, 6H), 7.04 – 6.98 (m, 2H), 6.09 (t, *J* = 9.7 Hz, 1H), 5.80 (t, *J* = 9.6 Hz, 1H), 5.69 – 5.59 (m, 2H), 5.56 (dd, *J* = 9.9, 7.9 Hz, 1H), 5.52 – 5.45 (m, 2H), 5.21 (dd, *J* = 9.8, 7.8 Hz, 1H), 5.12 – 5.04 (m, 2H), 4.88 (d, *J* = 11.0 Hz, 1H), 4.73 – 4.51 (m, 7H), 4.47 – 4.41 (m, 2H), 4.29 – 4.21 (m, 2H), 4.04 – 3.93 (m, 3H), 3.89 – 3.73 (m, 4H), 3.65 (dd, *J* = 11.3, 6.3 Hz, 1H), 3.53 (dt, *J* = 10.1, 2.6 Hz, 1H), 3.45 (dd, *J* = 10.9, 3.5 Hz, 1H), 3.40 – 3.32 (m, 2H), 3.29 (s, 3H). ^{13}C NMR (150 MHz, $CDCl_3$) δ 166.3, 165.94, 165.87, 165.7, 165.6, 165.42, 165.37, 165.1, 165.0, 139.1, 138.5, 138.3, 133.6, 133.41, 133.37, 133.30, 133.28, 133.2, 133.1, 130.1, 130.03, 129.97, 129.94, 129.92, 129.89, 129.87, 129.84, 129.82, 129.78, 129.5, 129.4, 129.2, 129.1, 129.04, 129.00, 128.97, 128.9, 128.8, 128.7, 128.6, 128.5, 128.45, 128.42, 128.39, 128.36, 128.3, 128.2, 128.00, 127.95, 127.7, 127.54, 127.51, 101.4, 101.2, 101.0, 98.2, 82.0, 80.0, 77.3, 77.2, 75.5, 74.6, 74.3, 73.9, 73.5, 73.0, 72.9, 72.7, 72.4, 72.2, 72.1, 72.0, 70.7, 69.8, 69.72, 69.65, 68.8, 68.2, 67.9, 63.4, 55.4.

Comparison of the donor reactivity of the Abz glycosides and thioglycosides, PPAP glycosides and thioglycosides under the action of NIS/TMSOTf and a one-pot synthesis of trisaccharide 29

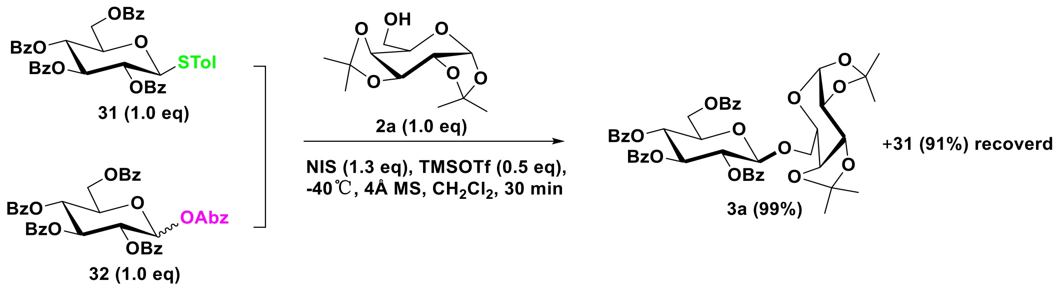

Fig. S37. Comparison of the donor reactivity of glycosyl Abz and thioglycoside.

A solution of glycosyl donor **31**(*15*) (70.3 mg, 0.1 mmol), **32**(*23*) (78.1 mg, 0.1 mmol), and acceptor **2a** (26.0 mg, 0.1 mmol) in dry CH_2Cl_2 (2.0 mL) was stirred at room temperature for 30 min in the presence of activated 4Å MS (120 mg) under N_2 atmosphere. Then the vessel was chilled to -40 °C, to which NIS (29.3 mg, 0.13 mmol) and TMSOTf (100 µL, 0.5 M in CH_2Cl_2) were added. The reaction mixture was stirred for 0.5 h at -40 °C. Then, Et_3N was added to quench the reaction. After the solvent was removed under reduced pressure, the resulting residue was purified by silica gel column chromatography (Petroleum ether/EtOAc = 4:1 to 3:1) to afford **3a**(*17*) (83.0 mg, 91%), and recover **31** (63.9 mg, 91%). Compound **3a:** ^{1}H NMR (400 MHz, Chloroform-*d*) δ 8.03 (d, *J* = 7.6 Hz, 2H), 7.97 (d, *J* = 7.7 Hz, 2H), 7.90 (d, *J* = 7.8 Hz, 2H), 7.83 (d, *J* = 7.6 Hz, 2H), 7.56 – 7.43 (m, 3H), 7.44 – 7.26 (m, 9H), 5.91 (t, *J* = 9.5 Hz, 1H), 5.69 (t, *J* = 9.7 Hz, 1H), 5.55 (dd, *J* = 9.7, 7.7 Hz, 1H), 5.42 (d, *J* = 4.9 Hz, 1H), 5.06 (d, *J* = 7.8 Hz, 1H), 4.65 (dd, *J* = 12.1, 3.1 Hz, 1H), 4.50 (dd, *J* = 12.1, 5.2 Hz, 1H), 4.44 (dd, *J* = 7.9, 2.3 Hz, 1H), 4.25 – 4.15 (m, 2H), 4.10 (dd, *J* = 7.9, 1.6 Hz, 1H), 4.03 (dd, *J* = 10.5, 3.5 Hz, 1H), 3.94 – 3.81 (m, 2H), 1.37 (s, 3H), 1.24 (s, 3H), 1.21 (s, 6H).

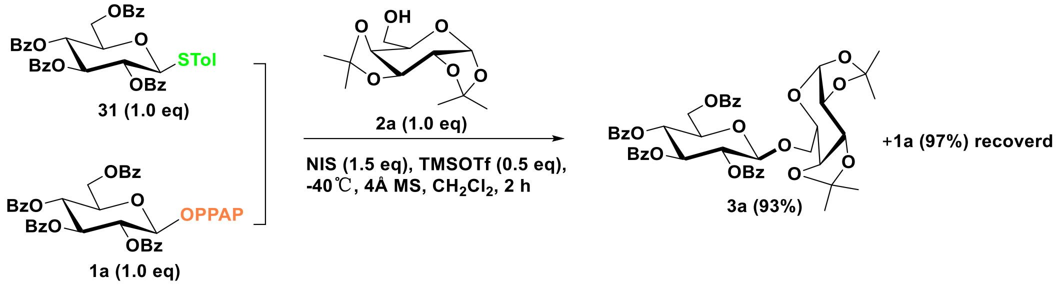

Fig. S38. Comparison of the donor reactivity of glycosyl glycosyl PPAP and thioglycoside.

A solution of glycosyl donor **31**(*15*) (70.3 mg, 0.1 mmol), **1a** (81.5 mg, 0.1 mmol), and acceptor **2a** (26.0 mg, 0.1 mmol) in dry CH_2Cl_2 (2.0 mL) was stirred at room temperature for 30 min in the presence of activated 4Å MS (120 mg) under N_2 atmosphere. Then the vessel was chilled to -40 °C, to which NIS (33.8 mg, 0.15 mmol) and TMSOTf (100 μL, 0.5 M in CH_2Cl_2) were added. The reaction mixture was stirred for 2.0 h at -40 °C. Then, Et_3N was added to quench the reaction. After the solvent was removed under reduced pressure, the resulting residue was purified by silica gel column chromatography (Petroleum ether/EtOAc = 4:1 to 3:1) to afford **3a***17* (77.9 mg, 93%), and recover **1a** (79.0 mg, 97%).

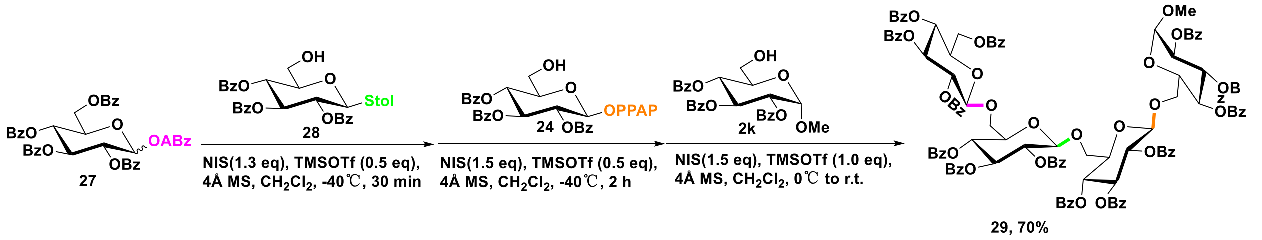

Fig. S39. One-pot synthesis of tetrasaccharide **29**.

A solution of glycosyl donor **27**(*23*) (86.0 mg, 0.11 mmol) and acceptor **28**(*89*) (59.8 mg, 0.1mmol) in dry CH_2Cl_2 (2.0 mL) was stirred at room temperature for 30 min in the presence of activated 4Å MS (120 mg) under N_2 atmosphere. Then the vessel was chilled to -40 °C, to which NIS (29.3 mg, 0.13 mmol) and TMSOTf (100 μL, 0.5 M in CH_2Cl_2) were added. The reaction mixture was stirred for 0.5 h at -40 °C. Acceptor **24** (56.9 mg, 0.08 mmol) was added to the reaction mixture, to which another portion of NIS (27.0 mg, 0.12 mmol) and TMSOTf (80 μL, 0.5 M in CH_2Cl_2) were added. The reaction mixture was stirred for 2.0 h at -40 °C. Acceptor **2k** (30.4 mg, 0.06 mmol) was added to the reaction mixture, to which another portion of NIS (20.3 mg, 0.09 mmol) and TMSOTf (120 μL, 0.5 M in CH_2Cl_2) were added. The reaction mixture was warmed gradually to room temperature and stirred for 3 h. Et_3N was added to quench the reaction and the solvent was removed under reduced pressure. The resulting residue was purified by silica gel column chromatography (Petroleum ether/EtOAc = 5:1 to 2.5:1) to afford tetrasaccharide **29:** $[\alpha]_D^{20}$= 3.2 (*c* 2.1, $CHCl_3$); ^{1}H NMR (600 MHz, Chloroform-*d*) δ 8.03 – 7.74 (m, 25H), 7.72 (d, *J* = 7.8 Hz, 2H), 7.57 – 7.30 (m, 28H), 7.25 – 7.22 (m, 4H), 7.17 (t, *J* = 7.7 Hz, 2H), 7.13 (t, *J* = 7.7 Hz, 2H), 7.08 (t, *J* = 7.7 Hz, 2H), 6.17 (t, *J* = 9.7 Hz, 1H), 6.10 (t, *J* = 9.9 Hz, 1H), 5.79 (dt, *J* = 24.6, 9.6 Hz, 2H), 5.66 (t, *J* = 9.7 Hz, 1H), 5.56 – 5.42 (m, 4H), 5.24 – 5.17 (m, 2H), 5.13 (d, *J* = 7.7 Hz, 1H), 5.09 (t, *J* = 9.7 Hz, 1H), 5.04 (d, *J* = 3.6 Hz, 1H), 4.83 (d, *J* = 7.9 Hz, 1H), 4.66 – 4.58 (m, 2H), 4.45 (dd, *J* = 12.1, 5.4 Hz, 1H), 4.36 (dt, *J* = 9.0, 4.0 Hz, 1H), 4.13 (t, *J* = 8.8 Hz, 1H), 4.03 (dd, *J* = 26.0, 11.8 Hz, 2H), 3.97 – 3.88 (m, 2H), 3.83 (dd, *J* = 11.9, 7.7 Hz, 1H), 3.75 – 3.66 (m, 2H), 3.49 (dd, *J* = 11.2, 5.1 Hz, 1H), 3.10 (s, 3H). ^{13}C NMR (150 MHz, $CDCl_3$) δ 166.3, 165.92, 165.88, 165.86, 165.7, 165.6, 165.42, 165.40, 165.36, 165.2, 165.1, 133.6, 133.49, 133.46, 133.42, 133.37, 133.3, 133.2, 133.0,

130.09, 130.06, 130.04, 130.01, 130.0, 129.93, 129.90, 129.84, 129.77, 129.7, 129.6, 129.5, 129.3, 129.1, 129.03, 129.00, 128.9, 128.8, 128.71, 128.65, 128.60, 128.55, 128.51, 128.49, 128.42, 128.38, 128.3, 128.2, 101.53, 101.49, 100.7, 96.7, 77.2, 74.3, 73.6, 73.0, 72.9, 72.7, 72.4, 72.3, 72.2, 72.1, 72.0, 70.6, 70.4, 70.0, 69.8, 69.6, 69.3, 68.6, 68.3, 68.1, 63.4, 55.2. HRMS (ESI) calcd for $C_{116}H_{96}O_{34}K$ $[M+K]^+$ 2071.5415, found 2071.5410.

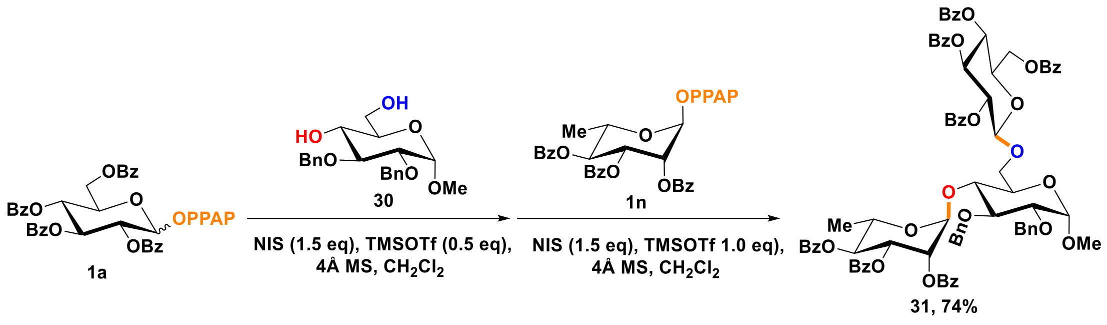

Fig. S40. One-pot synthesis of trisaccharide **31**.

A solution of glycosyl PPAP donor **1a** (49 mg, 0.06 mmol) and acceptor **30**(*90*) (18.7 mg, 0.05 mmol) in dry CH_2Cl_2 (0.05 M) was stirred at room temperature for 20 min in the presence of activated 4Å MS (150 mg) under N_2 atmosphere. Then NIS (17.0 mg, 0.075 mmol) and TMSOTf (50 μL, 0.05 M in CH_2Cl_2) was added at 0 °C. The reaction mixture was stirred for 3 h at room temperature. After the acceptor **30** was consumed completely (monitored by TLC), at 0 °C donor **1n** (52 mg, 0.075 mmol) and NIS (17.0 mg, 0.075 mmol) and TMSOTf (100 μL, 0.05 M in CH_2Cl_2) was added in sequence. The reaction mixture was warm to room temperature and stirred for 3 h. Then Et_3N was added to quench the reaction and the solvents were removed under reduced pressure. The resulting residue was purified by silica gel column chromatography (Petroleum ether/EtOAc = 4:1) to afford the glycosylated product **31** (52.8 mg, 74%) as a white solid: $[\alpha]_D^{25}$= 40.7 (*c* 0.51, CH_2Cl_2); ^1H NMR (600 MHz, Chloroform-*d*) δ 8.23 – 8.18 (m, 2H), 8.04 – 7.99 (m, 2H), 7.95 – 7.86 (m, 6H), 7.75 – 7.70 (m, 2H), 7.71 – 7.64 (m, 1H), 7.63 – 7.57 (m, 4H), 7.53 – 7.47 (m, 3H), 7.46 – 7.41 (m, 1H), 7.39 – 7.27 (m, 17H), 7.19 (t, *J* = 7.8 Hz, 2H), 7.16 – 7.12 (m, 3H), 6.94 (t, *J* = 7.8 Hz, 2H), 5.90 (t, *J* = 9.6 Hz, 1H), 5.74 – 5.67 (m, 2H), 5.63 (dd, *J* = 9.7, 7.8 Hz, 1H), 5.44 (dd, J = 3.5, 1.8 Hz, 1H), 5.40 (t, J = 10.1 Hz, 1H), 5.20 (d, *J* = 7.8 Hz, 1H), 5.11 (d, *J* = 11.0 Hz, 1H), 5.03 (d, *J* = 1.8 Hz, 1H), 4.73 (dd, *J* = 11.5, 6.6 Hz, 2H), 4.68 (dd, J = 12.0, 3.5 Hz, 1H), 4.64 – 4.57 (m, 3H), 4.39 – 4.30 (m, 2H), 4.20 – 4.11 (m, 1H), 4.05 (dd, J = 11.1, 3.1 Hz, 1H), 3.91 (t, J = 9.4 Hz, 1H), 3.85 (dt, J = 9.9, 2.6 Hz, 1H), 3.74 (t, J = 9.5 Hz, 1H), 3.59 (dd, J = 9.6, 3.5 Hz, 1H), 3.34 (s, 3H), 0.53 (d, J = 6.2 Hz, 3H). ^{13}C NMR (150 MHz, CDCl3) δ 166.3, 165.89, 165.88, 165.6, 165.4, 165.3, 138.8, 138.1, 133.8, 133.44, 133.37, 133.3, 133.14, 133.10, 132.9, 130.1, 130.0, 129.9, 129.84, 129.77, 129.7, 129.6, 129.5, 129.3, 129.1, 129.02, 128.99, 128.96, 128.7, 128.6, 128.5, 128.42, 128.38, 128.3, 128.2, 128.1, 128.0, 127.7, 127.4, 100.7, 98.0, 96.8, 80.4, 79.7, 77.2, 75.4, 75.0, 73.6, 73.2, 72.4, 71.8, 71.74, 71.69, 70.3, 70.2, 69.4, 67.1, 66.8, 63.6, 55.4. 29.8, 17.0. HRMS (ESI) calcd for $C_{82}H_{74}O_{22}Na$ $[M+Na]^+$ 1433.4564, Found: 1433.4567.

X-ray crystal structure data of compounds 4a and1r.

Single crystals suitable for X-ray studies were grown by slowly evaporating a solution of compound **4a** in a mixture of EtOAc and cyclohexane at room temperature. The X-ray data of **4a** is deposited in the Cambridge Crystallographic Data Center with a number of CCDC 2391061.

Crystal data and structure refinement of 4a

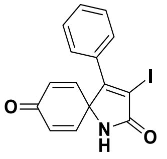

4a

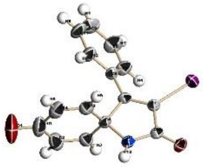

**Chemical formula**	$C_{15}H_{10}INO_2$
**Formula weight**	365.14 g/mol
**Temperature**	303(2) K
**Wavelength**	0.71073 Å
**Crystal system**	monoclinic
**Space group**	P 1 21/c 1
**Unit cell dimensions**	a = 13.9782(4) Å α = 90°<br>b = 10.7362(4) Å β = 100.6600(10)°<br>c = 9.5379(3) Å γ = 90°
**Volume**	1406.68(8) $Å^3$
**Z**	4
**Density (calculated)**	1.724 g/cm^3
**Absorption coefficient**	2.275 mm^{-1}
**F(000)**	708
**Diffractometer**	d8 venture
**Theta range for data collection**	2.41 to 28.37°
**Index ranges**	-18 < = h < = 18, -14 < = k < = 14, -12 < = l < = 12
**Reflections collected**	35907
**Independent reflections**	3512 [R(int) = 0.0838]
**Coverage of independent reflections**	99.8%
**Absorption correction**	Multi-Scan
**Structure solution technique**	direct methods
**Structure solution program**	SHELXT 2018/2 (Sheldrick, 2018)
**Refinement method**	Full-matrix least-squares on F^2
**Refinement program**	SHELXL-2018/3 (Sheldrick, 2018)
**Function minimized**	$\Sigma w(F_o^2 - F_c^2)^2$
**Data / restraints / parameters**	3512 / 0 / 175
**Goodness-of-fit on F^2**	1.070
**Δ/σ_{max}**	0.001
**Final R indices**	2774 data; I>2σ(I) R1 = 0.0351, wR2 = 0.0685<br>all data R1 = 0.0542, wR2 = 0.0757
**Weighting scheme**	$w=1/[\sigma^2(F_o^2)+(0.0203P)^2+2.1410P]$<br>where $P=(F_o^2+2F_c^2)/3$
**Largest diff. peak and hole**	0.713 and -0.737 $eÅ^{-3}$
**R.M.S. deviation from mean**	0.092 $eÅ^{-3}$

Crystal data and structure refinement of 1r

Single crystals suitable for X-ray studies were grown by slowly evaporating a solution of compound **1r** in a mixture of EtOAc and cycloPetroleum ether at room temperature. The X-ray data of **1r** is deposited in the Cambridge Crystallographic Data Center with a number of CCDC 2391062.

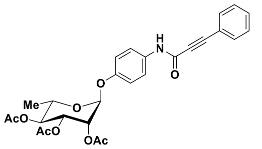

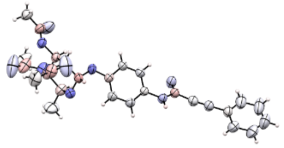

**Chemical formula**	$C_{27}H_{27}NO_9$
**Formula weight**	1528.48 g/mol
**Temperature**	303(2) K
**Wavelength**	0.71073 Å
**Crystal size**	0.100 x 0.150 x 0.200 mm
**Crystal habit**	clear light colourless block
**Crystal system**	monoclinic
**Space group**	P 1 21 1
**Unit cell dimensions**	a = 18.459(5) Å α = 90°<br>b = 9.761(2) Å β = 97.900(7)°<br>c = 22.245(7) Å γ = 90°
**Volume**	3970.0(19) $Å^3$
**Z**	2
**Density (calculated)**	1.279 g/cm^3
**Absorption coefficient**	0.097 mm^{-1}
**F(000)**	1608
**Diffractometer**	d8 venture
**Theta range for data collection**	0.92 to 24.91°
**Index ranges**	-21<=h<=21, -11<=k<=11, -26<=l<=25
**Reflections collected**	92531
**Independent reflections**	13573 [R(int) = 0.0913]
**Coverage of independent reflections**	98.9%
**Absorption correction**	Multi-Scan
**Max. and min. transmission**	0.9900 and 0.9810
**Structure solution technique**	direct methods
**Structure solution program**	SHELXT 2018/2 (Sheldrick, 2018)
**Refinement method**	Full-matrix least-squares on F^2
**Refinement program**	SHELXL-2018/3 (Sheldrick, 2018)
**Function minimized**	$\Sigma w(F_o^2 - F_c^2)^2$
**Final R indices**	8014 data; I>2σ(I) R1 = 0.0560, wR2 = 0.1233<br>all data R1 = 0.1118, wR2 = 0.1491
**Weighting scheme**	$w=1/[\sigma^2(F_o^2)+(0.0663P)^2]$<br>where $P=(F_o^2+2F_c^2)/3$
**Absolute structure parameter**	-1.1(7)
**Largest diff. peak and hole**	0.182 and -0.232 $eÅ^{-3}$
**R.M.S. deviation from mean**	0.048 $eÅ^{-3}$

NMR Spectra.

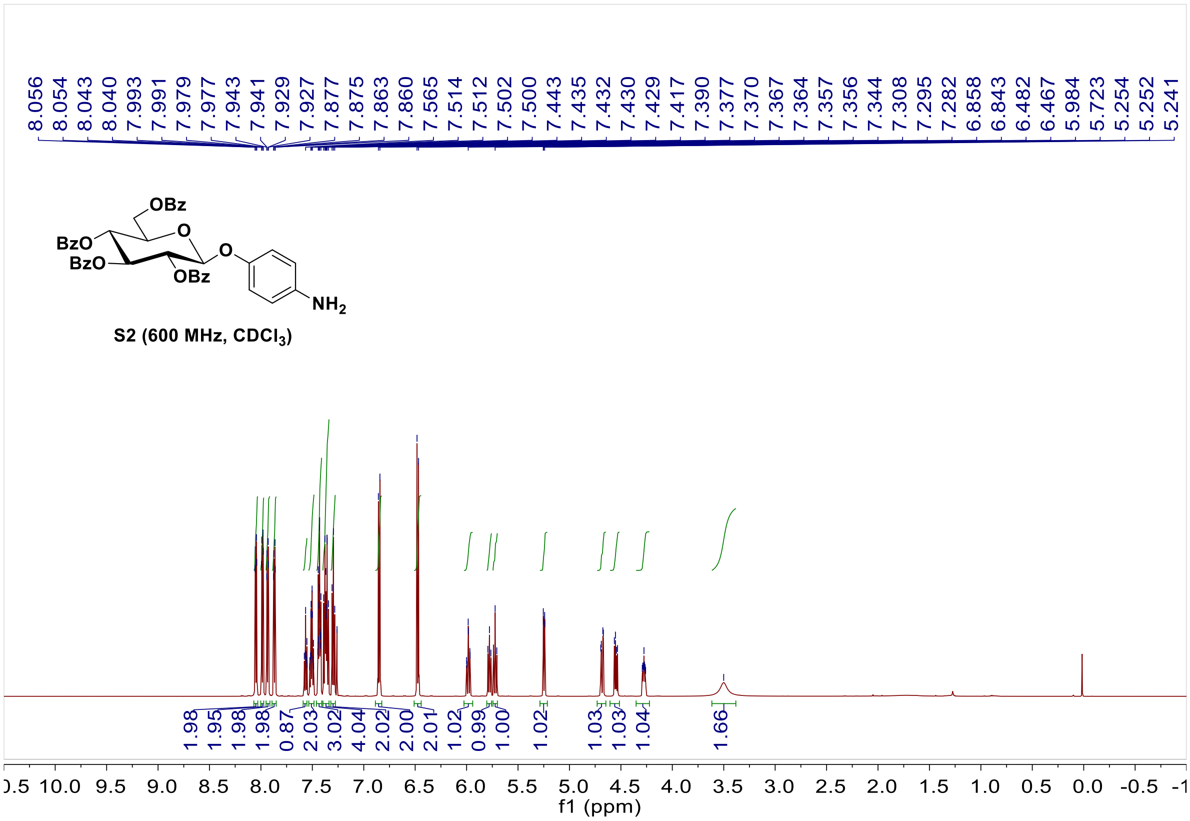
8.056
8.054
8.043
8.040
7.993
7.991
7.979
7.977
7.943
7.941
7.929
7.927
7.877
7.875
7.863
7.860
7.565
7.514
7.512
7.502
7.500
7.443
7.435
7.432
7.430
7.429
7.417
7.390
7.377
7.370
7.367
7.364
7.357
7.356
7.344
7.308
7.295
7.282
6.858
6.843
6.482
6.467
5.984
5.723
5.254
5.252
5.241
OBz
BzO
BzO
O
O
OBz
NH2
S2 (600 MHz, CDCl3)
1.98
1.95
1.98
1.98
0.87
2.03
3.02
4.04
2.02
2.00
2.01
1.02
0.99
1.00
1.02
1.03
1.03
1.04
1.66
f1 (ppm)

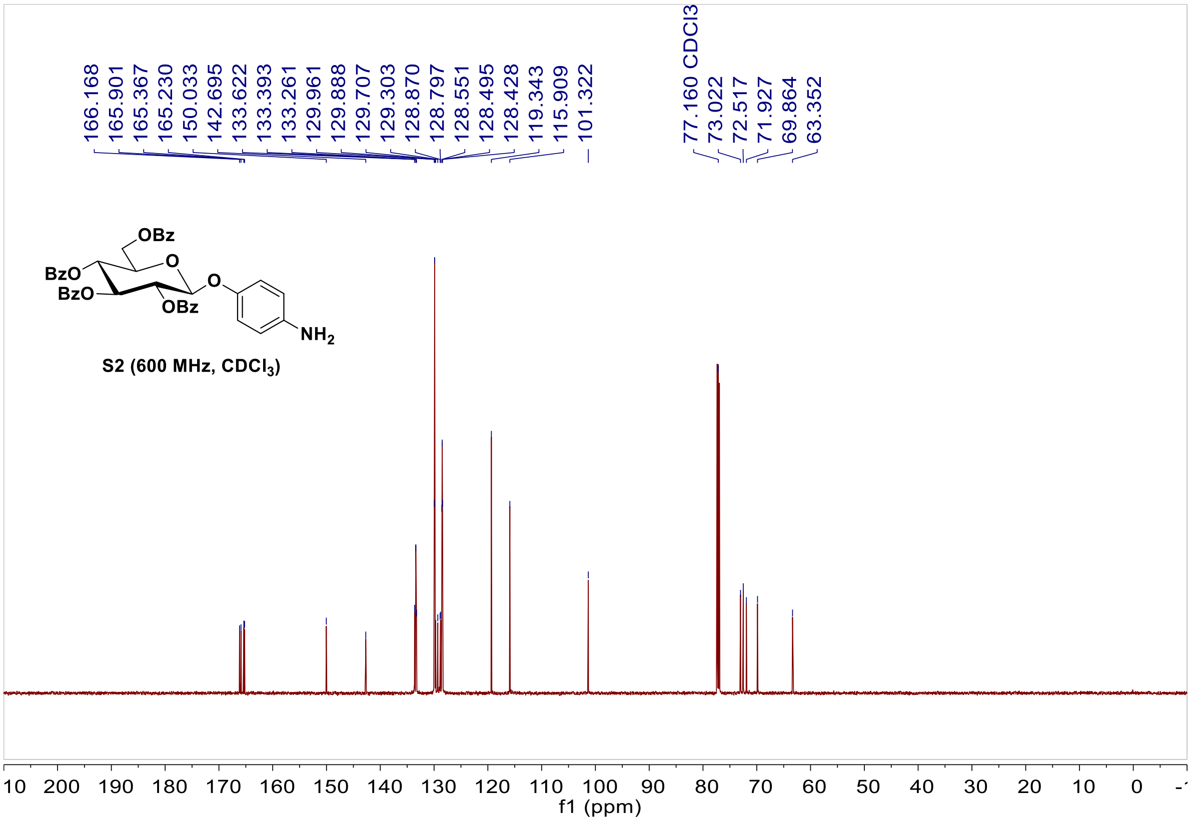
166.168
165.901
165.367
165.230
150.033
142.695
133.622
133.393
133.261
129.961
129.888
129.707
129.303
128.870
128.797
128.551
128.495
128.428
119.343
115.909
101.322
77.160 CDCl3
73.022
72.517
71.927
69.864
63.352
OBz
BzO
BzO
O
O
OBz
NH2
S2 (600 MHz, CDCl3)
f1 (ppm)

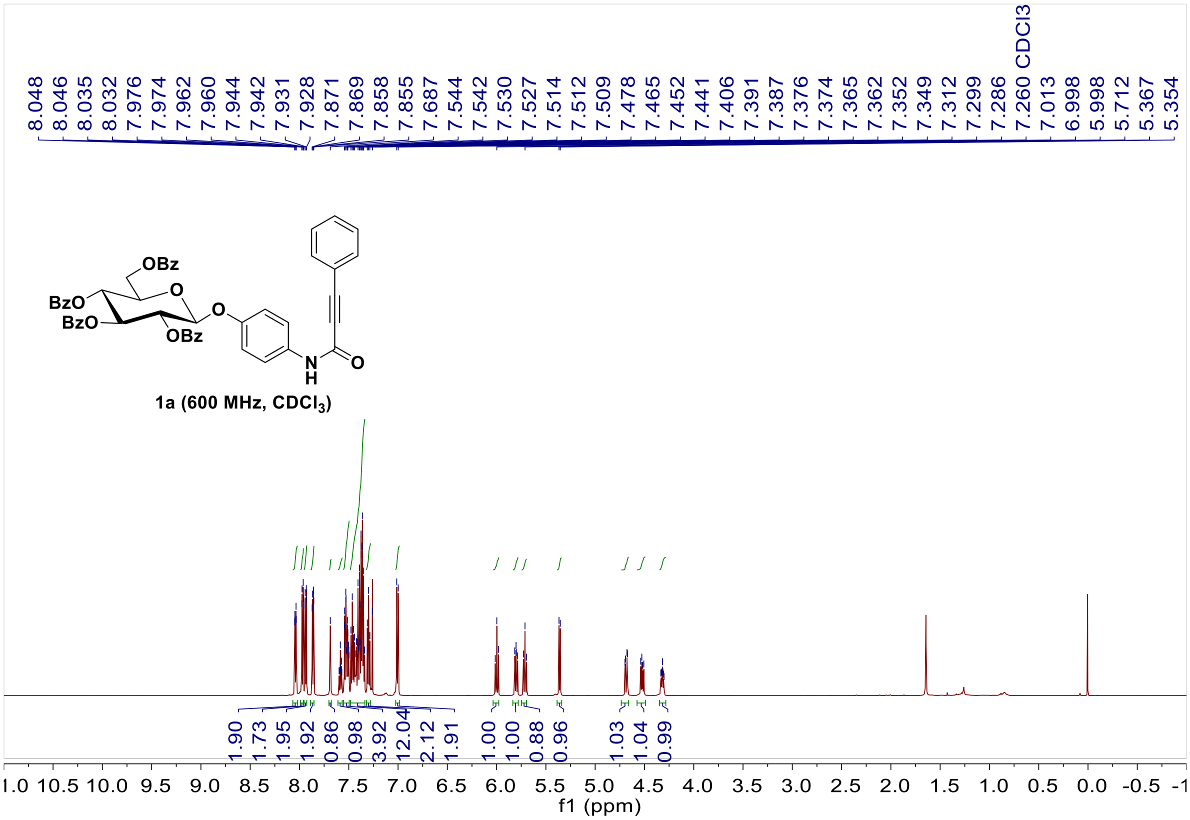
8.048 8.046 8.035 8.032 7.976 7.974 7.962 7.960 7.944 7.942 7.931 7.928 7.871 7.869 7.858 7.855 7.687 7.544 7.542 7.530 7.527 7.514 7.512 7.509 7.478 7.465 7.452 7.441 7.406 7.391 7.387 7.376 7.374 7.365 7.362 7.352 7.349 7.312 7.299 7.286 7.260 CDCl3 7.013 6.998 5.998 5.712 5.367 5.354
OBz
BzO
BzO
O
O
OBz
N
H
O
1a (600 MHz, CDCl3)
1.90 1.73 1.95 1.92 0.86 0.98 3.92 12.04 2.12 1.91 1.00 1.00 0.88 0.96 1.03 1.04 0.99
1.0 10.5 10.0 9.5 9.0 8.5 8.0 7.5 7.0 6.5 6.0 5.5 5.0 4.5 4.0 3.5 3.0 2.5 2.0 1.5 1.0 0.5 0.0 -0.5 -1
f1 (ppm)

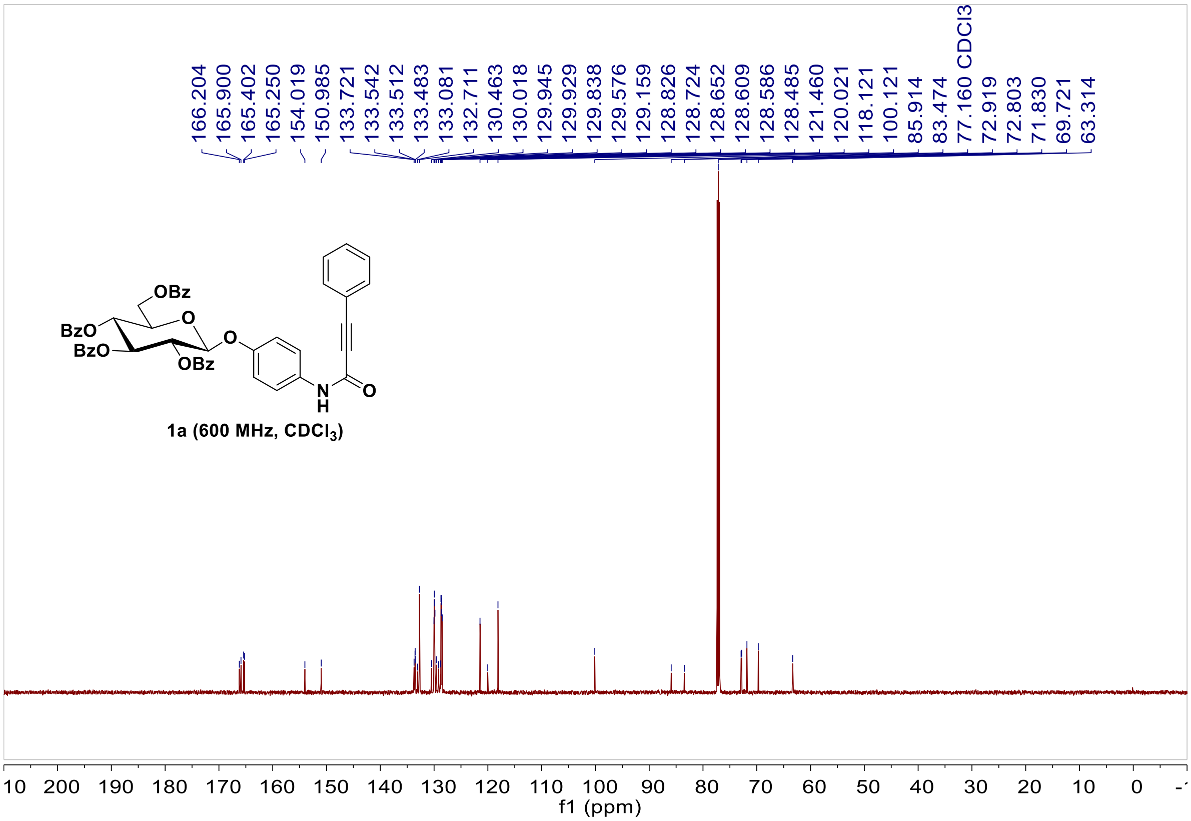
166.204 165.900 165.402 165.250 154.019 150.985 133.721 133.542 133.512 133.483 133.081 132.711 130.463 130.018 129.945 129.929 129.838 129.576 129.159 128.826 128.724 128.652 128.609 128.586 128.485 121.460 120.021 118.121 100.121 85.914 83.474 77.160 CDCl3 72.919 72.803 71.830 69.721 63.314
OBz
BzO
BzO
O
O
OBz
N
H
O
1a (600 MHz, CDCl3)
10 200 190 180 170 160 150 140 130 120 110 100 90 80 70 60 50 40 30 20 10 0
f1 (ppm)

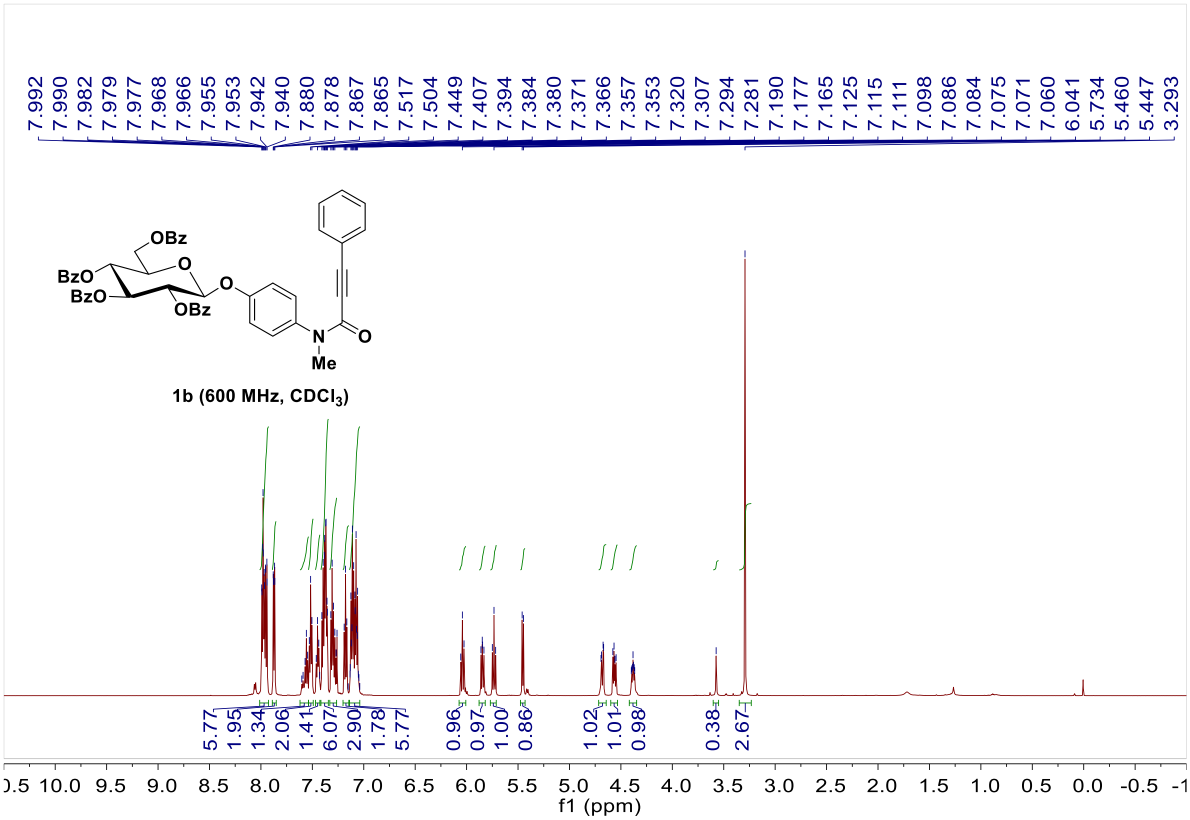

7.992 7.990 7.982 7.979 7.977 7.968 7.966 7.955 7.953 7.942 7.940 7.880 7.878 7.867 7.865 7.517 7.504 7.449 7.407 7.394 7.384 7.380 7.371 7.366 7.357 7.353 7.320 7.307 7.294 7.281 7.190 7.177 7.165 7.125 7.115 7.111 7.098 7.086 7.084 7.075 7.071 7.060 6.041 5.734 5.460 5.447 3.293
OBz
BzO
BzO
O
OBz
O
N
O
Me
1b (600 MHz, CDCl3)
5.77 1.95 1.34 2.06 1.41 6.07 2.90 1.78 5.77 0.96 0.97 1.00 0.86 1.02 1.01 0.98 0.38 2.67
f1 (ppm)

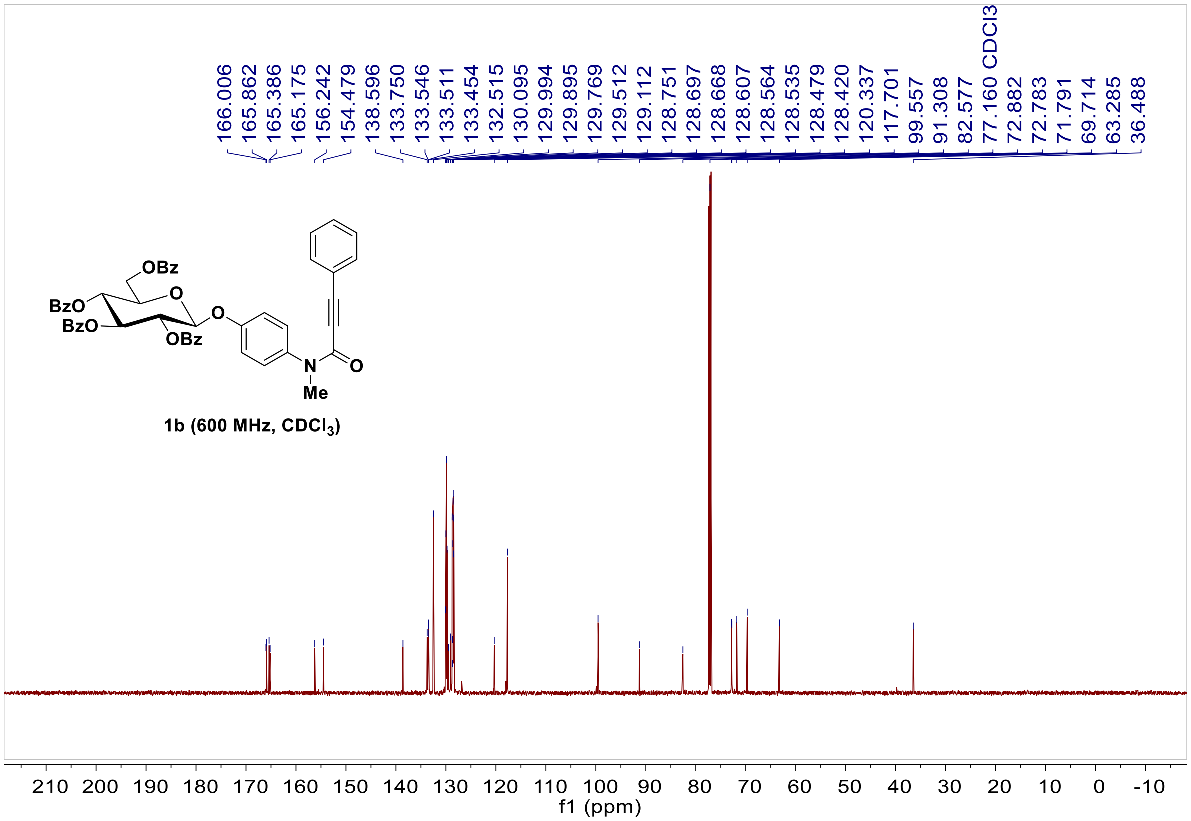

166.006 165.862 165.386 165.175 156.242 154.479 138.596 133.750 133.546 133.511 133.454 132.515 130.095 129.994 129.895 129.769 129.512 129.112 128.751 128.697 128.668 128.607 128.564 128.535 128.479 128.420 120.337 117.701 99.557 91.308 82.577 77.160 CDCl3 72.882 72.783 71.791 69.714 63.285 36.488
OBz
BzO
BzO
O
OBz
O
N
O
Me
1b (600 MHz, CDCl3)
f1 (ppm)

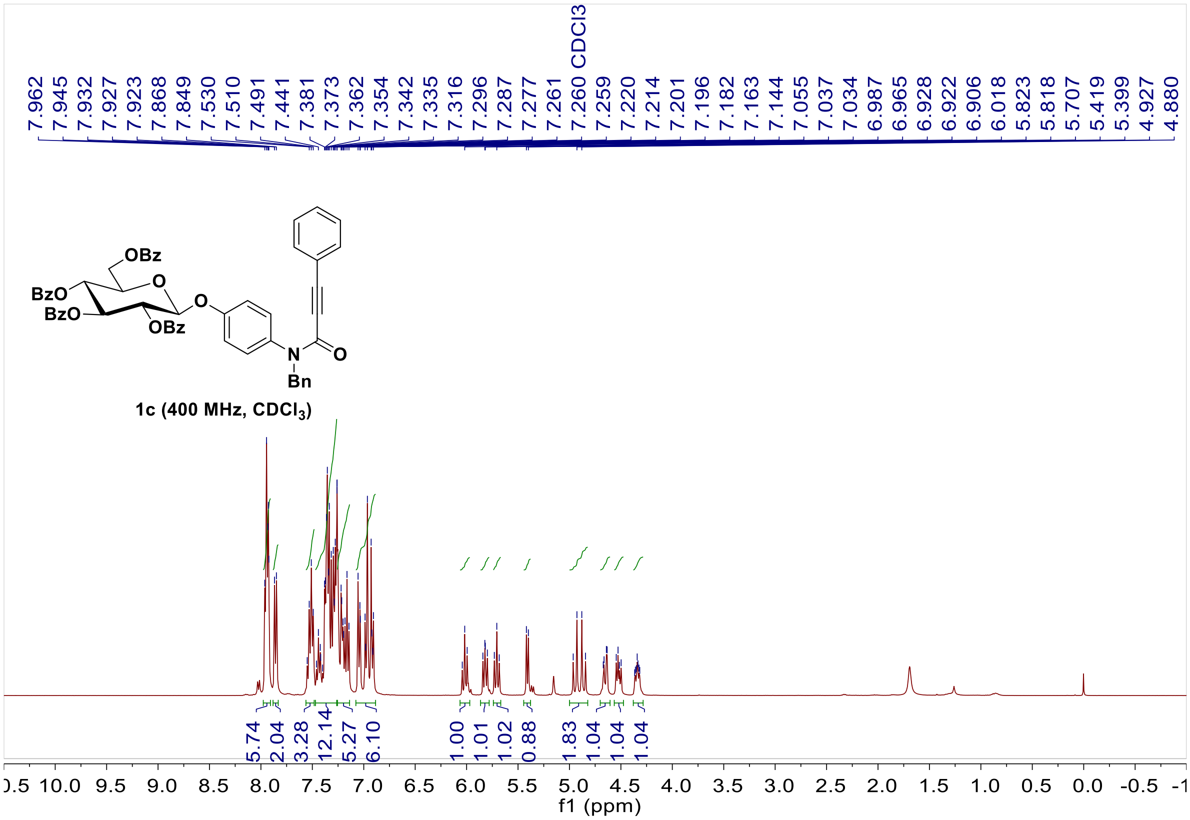
7.962 7.945 7.932 7.927 7.923 7.868 7.849 7.530 7.510 7.491 7.441 7.381 7.373 7.362 7.354 7.342 7.335 7.316 7.296 7.287 7.277 7.261 7.260 CDCl3 7.259 7.220 7.214 7.201 7.196 7.182 7.163 7.144 7.055 7.037 7.034 6.987 6.965 6.928 6.922 6.906 6.018 5.823 5.818 5.707 5.419 5.399 4.927 4.880
OBz
BzO
BzO
O
OBz
O
N
O
Bn
1c (400 MHz, CDCl3)
5.74 2.04 3.28 12.14 5.27 6.10 1.00 1.01 1.02 0.88 1.83 1.04 1.04 1.04
f1 (ppm)

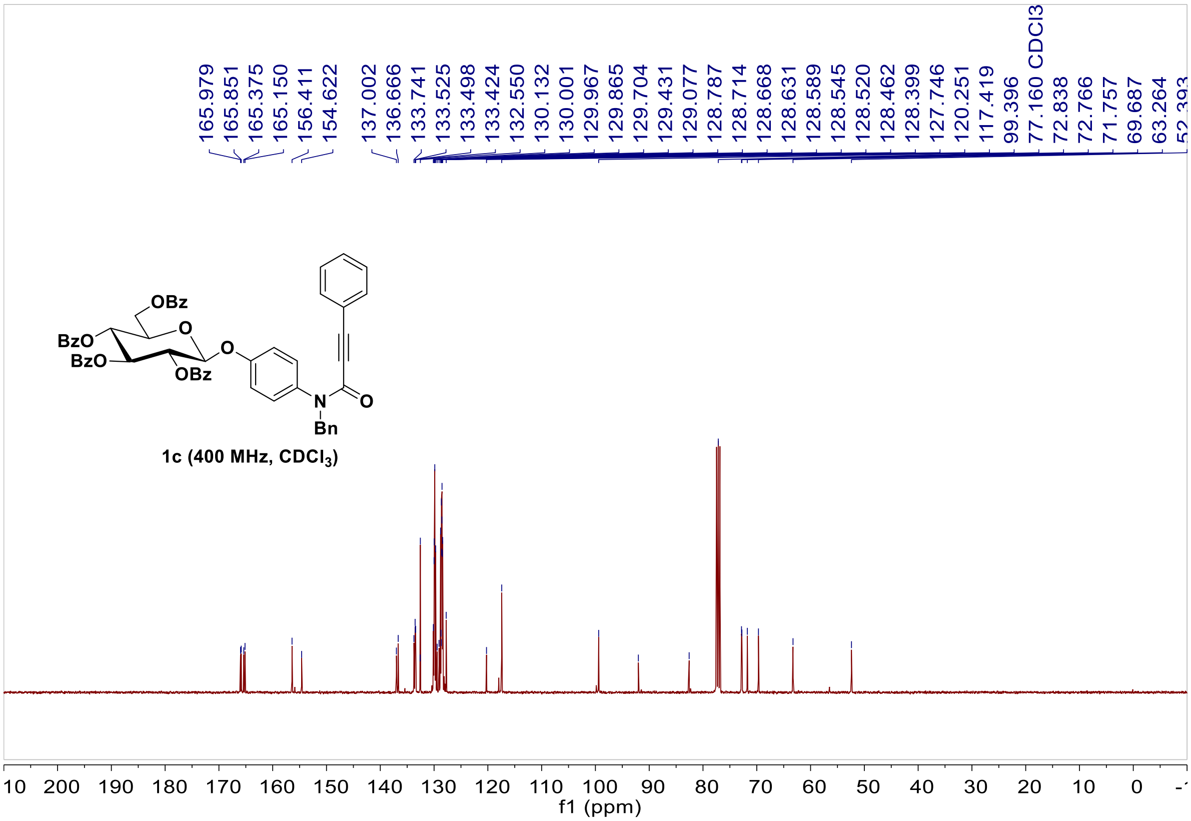
165.979 165.851 165.375 165.150 156.411 154.622 137.002 136.666 133.741 133.525 133.498 133.424 132.550 130.132 130.001 129.967 129.865 129.704 129.431 129.077 128.787 128.714 128.668 128.631 128.589 128.545 128.520 128.462 128.399 127.746 120.251 117.419 99.396 77.160 CDCl3 72.838 72.766 71.757 69.687 63.264
OBz
BzO
BzO
O
OBz
O
N
O
Bn
1c (400 MHz, CDCl3)
f1 (ppm)

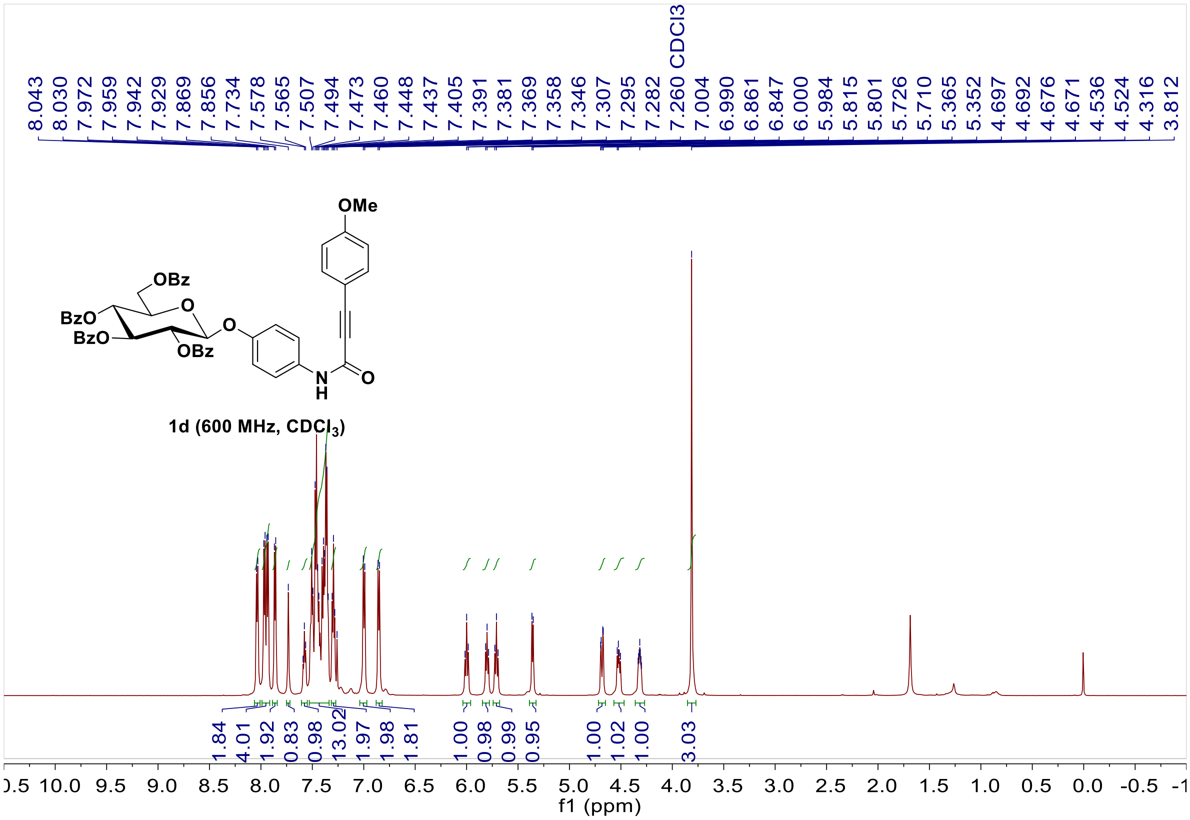
8.043 8.030 7.972 7.959 7.942 7.929 7.869 7.856 7.734 7.578 7.565 7.507 7.494 7.473 7.460 7.448 7.437 7.405 7.391 7.381 7.369 7.358 7.346 7.307 7.295 7.282 7.260 CDCl3 7.004 6.990 6.861 6.847 6.000 5.984 5.815 5.801 5.726 5.710 5.365 5.352 4.697 4.692 4.676 4.671 4.536 4.524 4.316 3.812
OMe
OBz
O
BzO
BzO
OBz
O
N
H
O
1d (600 MHz, CDCl$_3$)
1.84 4.01 1.92 0.83 0.98 13.02 1.97 1.98 1.81 1.00 0.98 0.99 0.95 1.00 1.02 1.00 3.03
f1 (ppm)

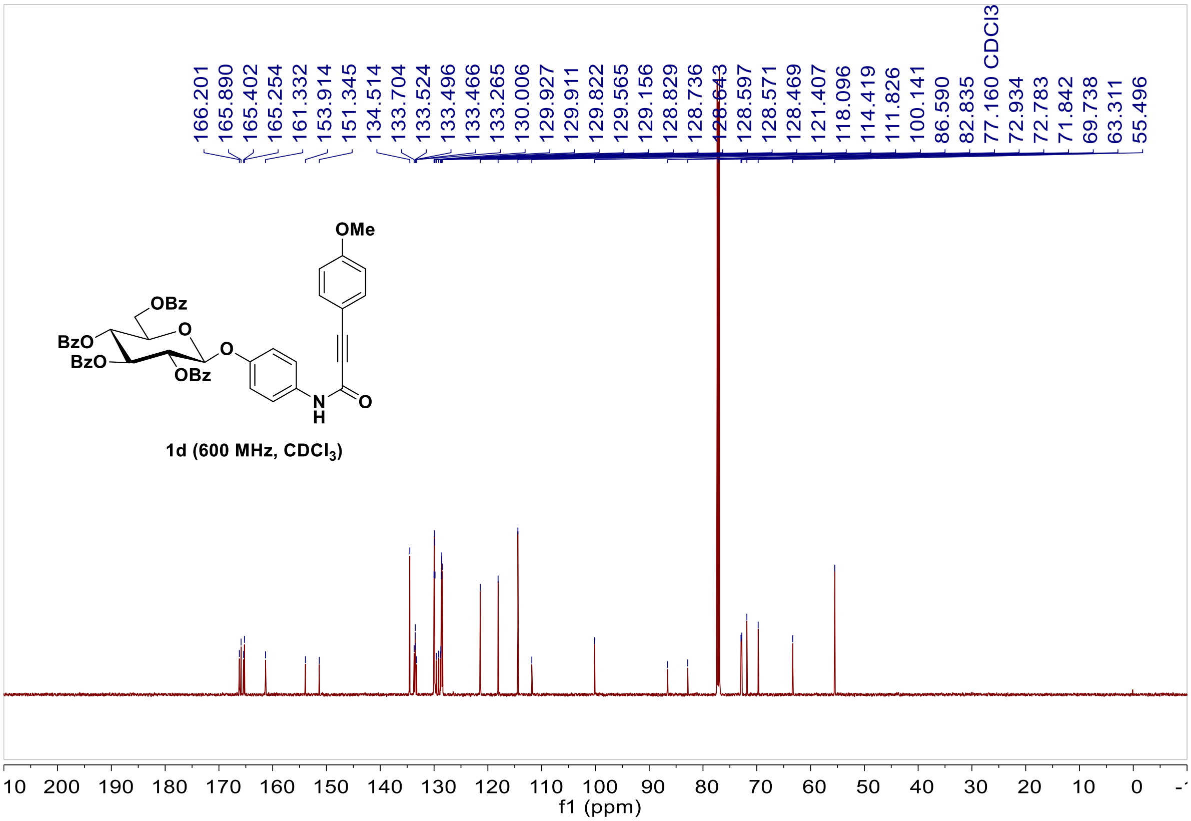
166.201 165.890 165.402 165.254 161.332 153.914 151.345 134.514 133.704 133.524 133.496 133.466 133.265 130.006 129.927 129.911 129.822 129.565 129.156 128.829 128.736 128.643 128.597 128.571 128.469 121.407 118.096 114.419 111.826 100.141 86.590 82.835 77.160 CDCl3 72.934 72.783 71.842 69.738 63.311 55.496
OMe
OBz
O
BzO
BzO
OBz
O
N
H
O
1d (600 MHz, CDCl$_3$)
f1 (ppm)

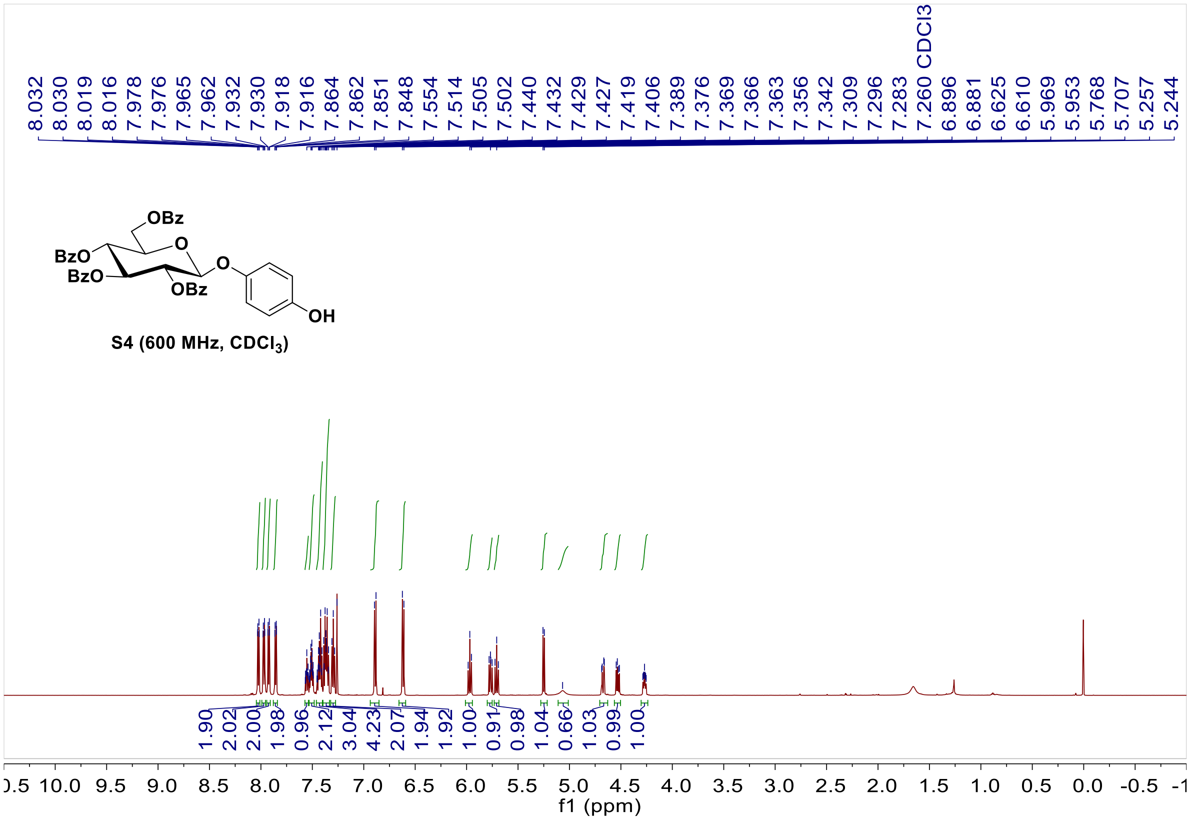
8.032 8.030 8.019 8.016 7.978 7.976 7.965 7.962 7.932 7.930 7.918 7.916 7.864 7.862 7.851 7.848 7.554 7.514 7.505 7.502 7.440 7.432 7.429 7.427 7.419 7.406 7.389 7.376 7.369 7.366 7.363 7.356 7.342 7.309 7.296 7.283 7.260 CDCl3 6.896 6.881 6.625 6.610 5.969 5.953 5.768 5.707 5.257 5.244
OBz
BzO
BzO
O
O
OBz
OH
S4 (600 MHz, CDCl3)
1.90 2.02 2.00 1.98 0.96 2.12 3.04 4.23 2.07 1.94 1.92 1.00 0.91 0.98 1.04 0.66 1.03 0.99 1.00
f1 (ppm)

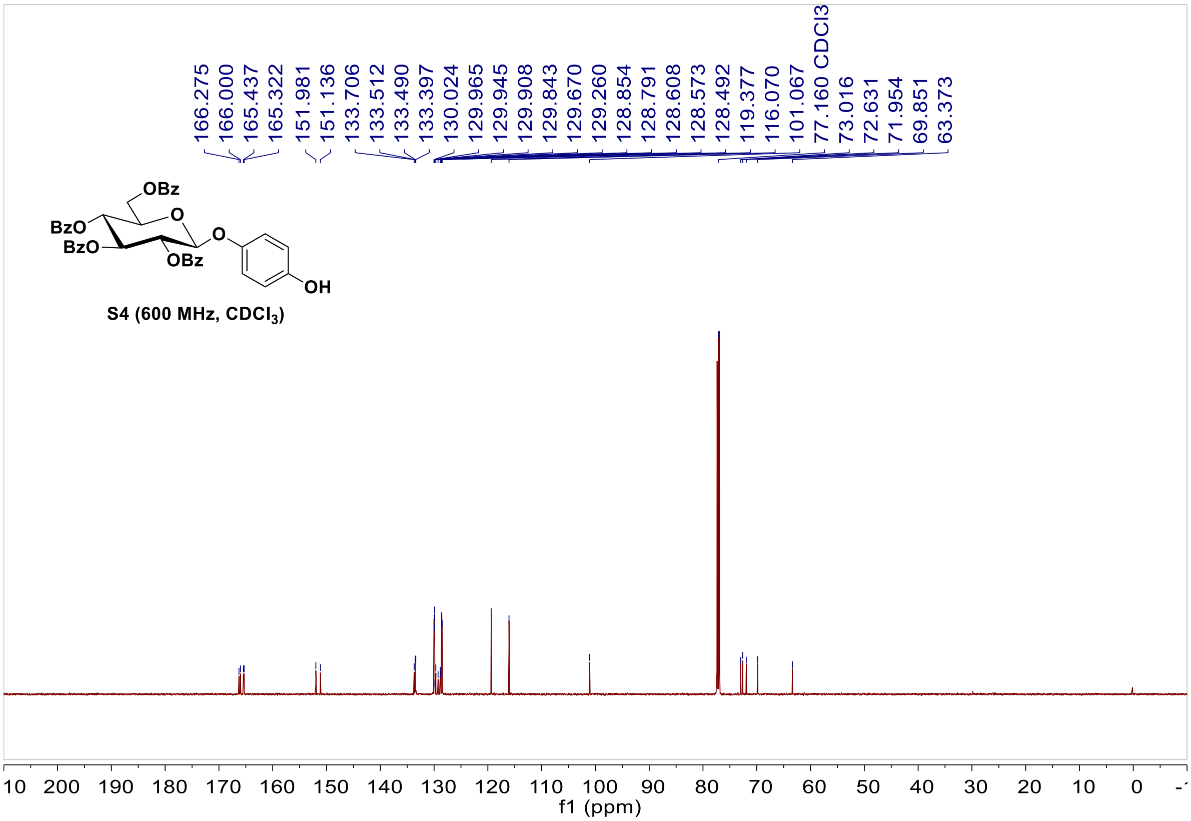
166.275 166.000 165.437 165.322 151.981 151.136 133.706 133.512 133.490 133.397 130.024 129.965 129.945 129.908 129.843 129.670 129.260 128.854 128.791 128.608 128.573 128.492 119.377 116.070 101.067 77.160 CDCl3 73.016 72.631 71.954 69.851 63.373
OBz
BzO
BzO
O
O
OBz
OH
S4 (600 MHz, CDCl3)
f1 (ppm)

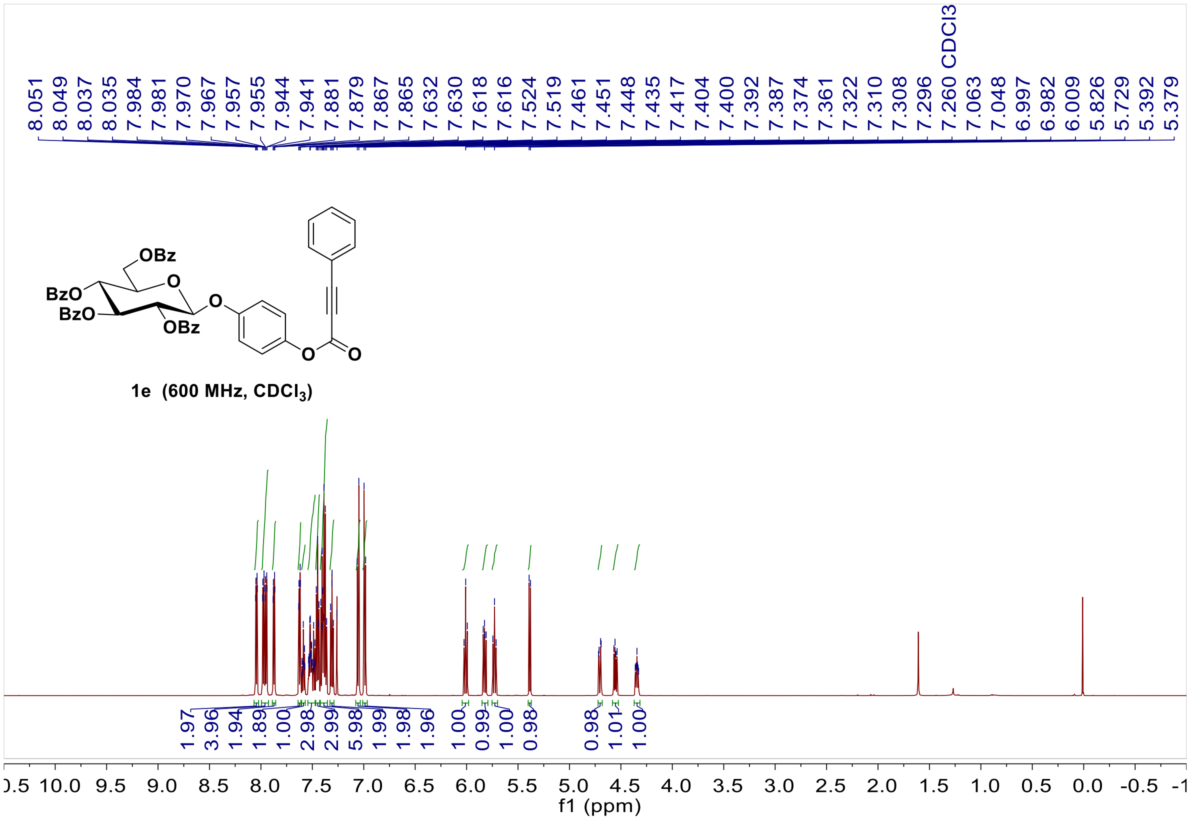
8.051 8.049 8.037 8.035 7.984 7.981 7.970 7.967 7.957 7.955 7.944 7.941 7.881 7.879 7.867 7.865 7.632 7.630 7.618 7.616 7.524 7.519 7.461 7.451 7.448 7.435 7.417 7.404 7.400 7.392 7.387 7.374 7.361 7.322 7.310 7.308 7.296 7.260 CDCl3 7.063 7.048 6.997 6.982 6.009 5.826 5.729 5.392 5.379
OBz
BzO
BzO
O
OBz
O
O
O
1e (600 MHz, $CDCl_3$)
1.97 3.96 1.94 1.89 1.00 2.98 2.99 5.98 1.99 1.98 1.96 1.00 0.99 1.00 0.98 0.98 1.01 1.00
f1 (ppm)

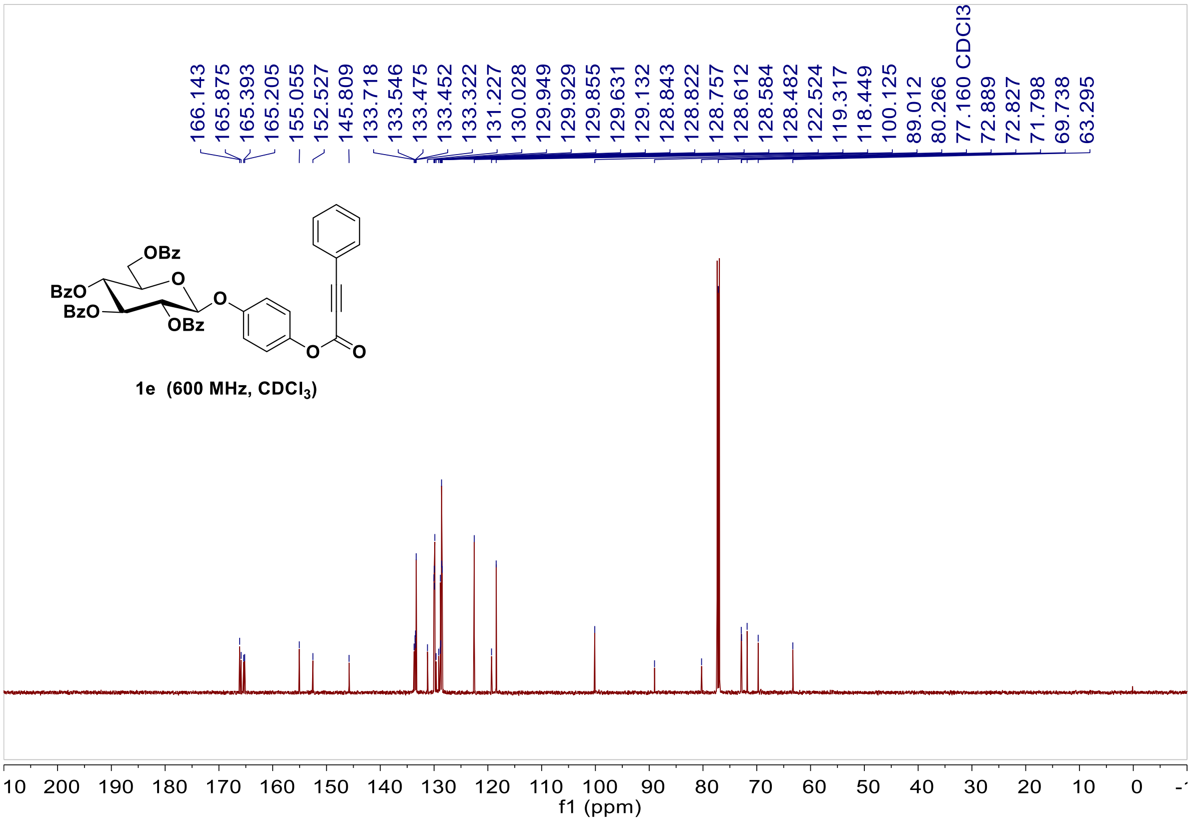
166.143 165.875 165.393 165.205 155.055 152.527 145.809 133.718 133.546 133.475 133.452 133.322 131.227 130.028 129.949 129.929 129.855 129.631 129.132 128.843 128.822 128.757 128.612 128.584 128.482 122.524 119.317 118.449 100.125 89.012 80.266 77.160 CDCl3 72.889 72.827 71.798 69.738 63.295
OBz
BzO
BzO
O
OBz
O
O
O
1e (600 MHz, $CDCl_3$)
f1 (ppm)

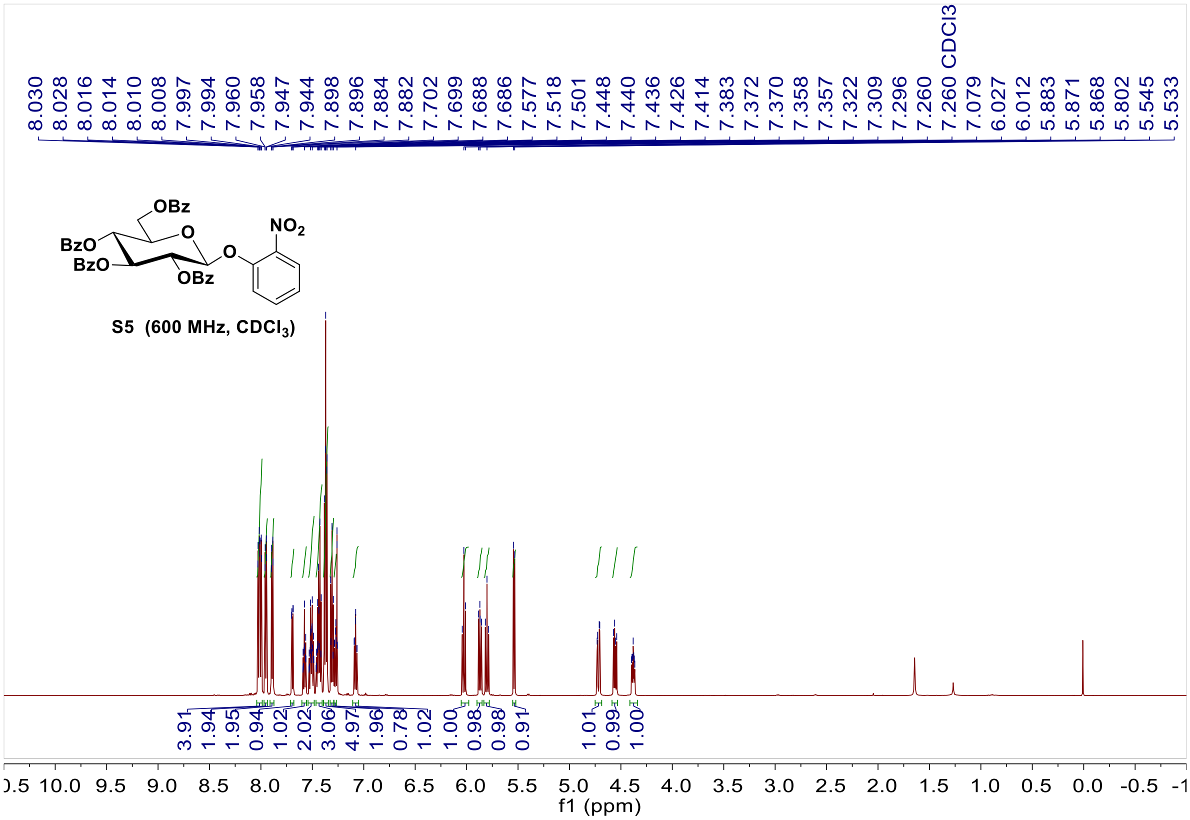
8.030 8.028 8.016 8.014 8.010 8.008 7.997 7.994 7.960 7.958 7.947 7.944 7.898 7.896 7.884 7.882 7.702 7.699 7.688 7.686 7.577 7.518 7.501 7.448 7.440 7.436 7.426 7.414 7.383 7.372 7.370 7.358 7.357 7.322 7.309 7.296 7.260 7.260 CDCl3 7.079 6.027 6.012 5.883 5.871 5.868 5.802 5.545 5.533
OBz
BzO
BzO
O
OBz
O
NO_2
S5 (600 MHz, $CDCl_3$)
3.91 1.94 1.95 0.94 1.02 2.02 3.06 4.97 1.96 0.78 1.02 1.00 0.98 0.98 0.91 1.01 0.99 1.00
f1 (ppm)

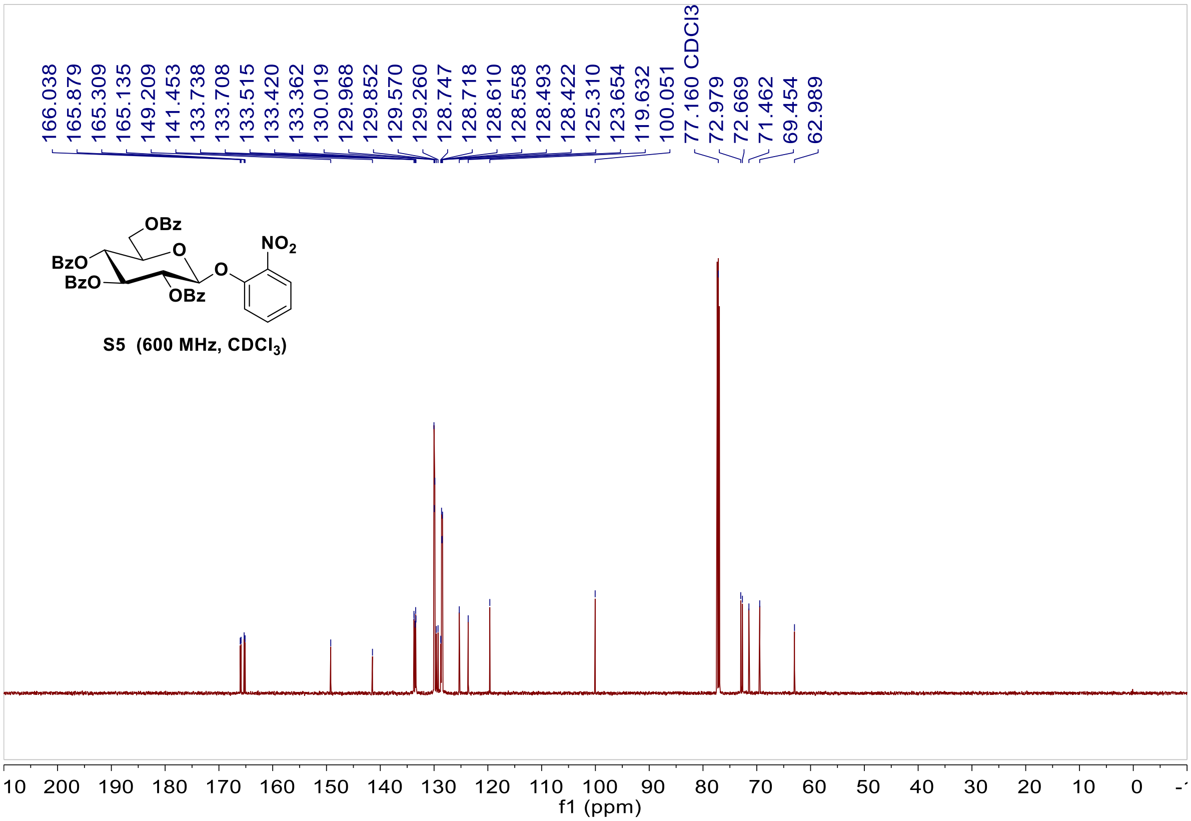
166.038 165.879 165.309 165.135 149.209 141.453 133.738 133.708 133.515 133.420 133.362 130.019 129.968 129.852 129.570 129.260 128.747 128.718 128.610 128.558 128.493 128.422 125.310 123.654 119.632 100.051 77.160 CDCl3 72.979 72.669 71.462 69.454 62.989
OBz
BzO
BzO
O
OBz
O
NO_2
S5 (600 MHz, $CDCl_3$)
f1 (ppm)

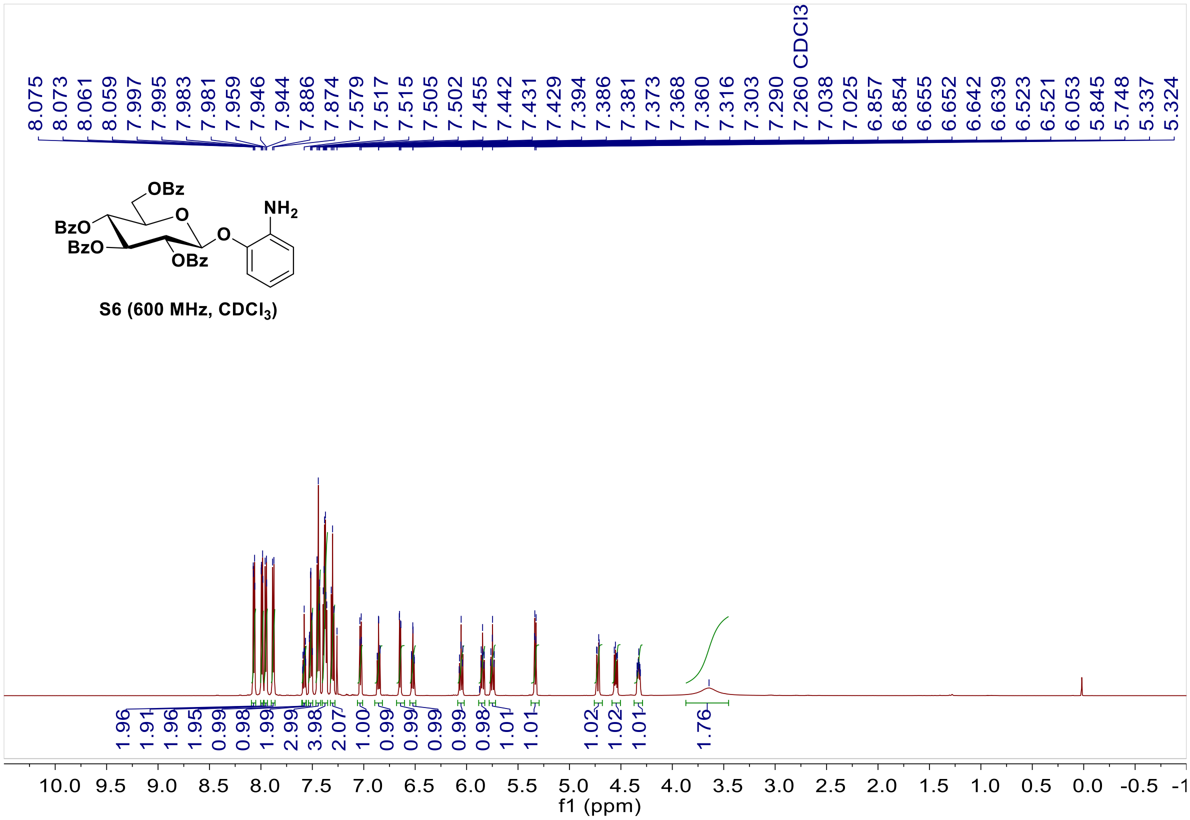
8.075 8.073 8.061 8.059 7.997 7.995 7.983 7.981 7.959 7.946 7.944 7.886 7.874 7.579 7.517 7.515 7.505 7.502 7.455 7.442 7.431 7.429 7.394 7.386 7.381 7.373 7.368 7.360 7.316 7.303 7.290 7.260 CDCl3 7.038 7.025 6.857 6.854 6.655 6.652 6.642 6.639 6.523 6.521 6.053 5.845 5.748 5.337 5.324
OBz
BzO
BzO
O
OBz
O
NH2
S6 (600 MHz, CDCl3)
1.96 1.91 1.96 1.95 0.99 0.98 1.99 2.99 3.98 2.07 1.00 0.99 0.99 0.99 0.99 0.98 1.01 1.01 1.02 1.02 1.01 1.76
10.0 9.5 9.0 8.5 8.0 7.5 7.0 6.5 6.0 5.5 5.0 4.5 4.0 3.5 3.0 2.5 2.0 1.5 1.0 0.5 0.0 -0.5 -1
f1 (ppm)
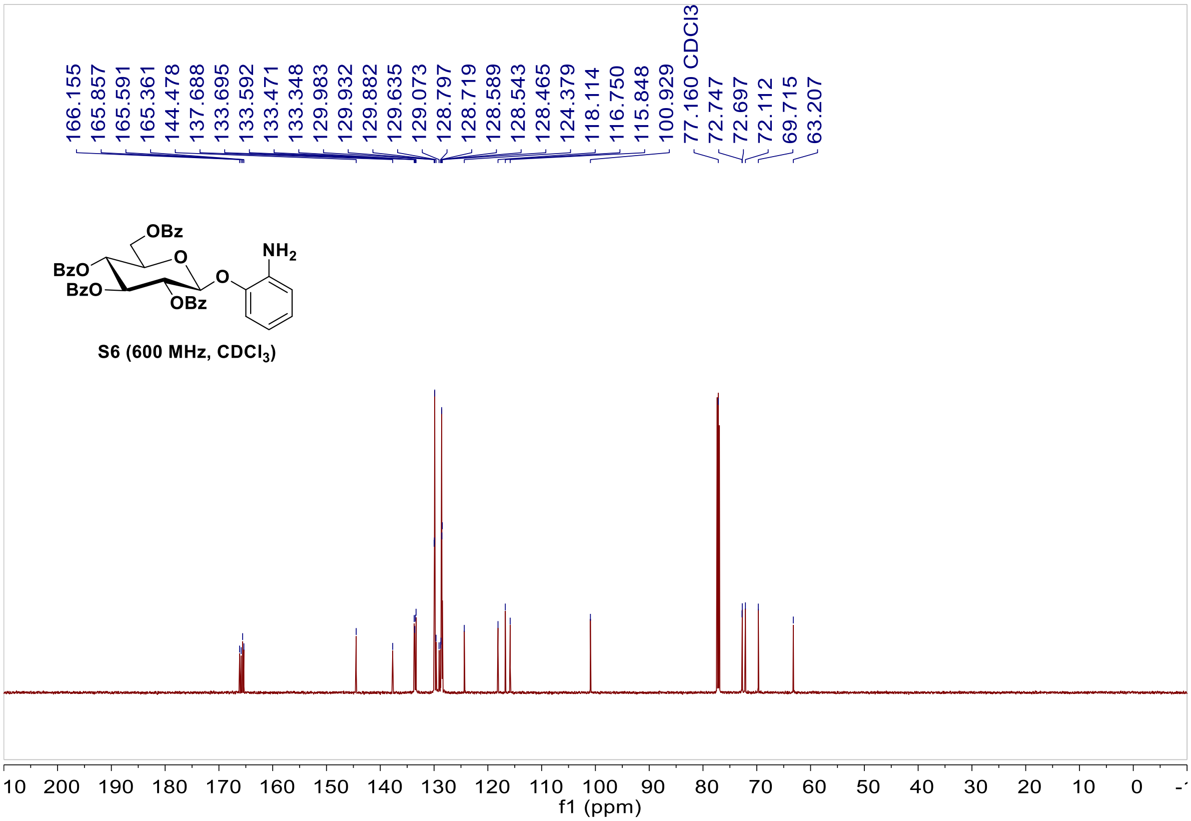
166.155 165.857 165.591 165.361 144.478 137.688 133.695 133.592 133.471 133.348 129.983 129.932 129.882 129.635 129.073 128.797 128.719 128.589 128.543 128.465 124.379 118.114 116.750 115.848 100.929 77.160 CDCl3 72.747 72.697 72.112 69.715 63.207
OBz
BzO
BzO
O
OBz
O
NH2
S6 (600 MHz, CDCl3)
10 200 190 180 170 160 150 140 130 120 110 100 90 80 70 60 50 40 30 20 10 0
f1 (ppm)

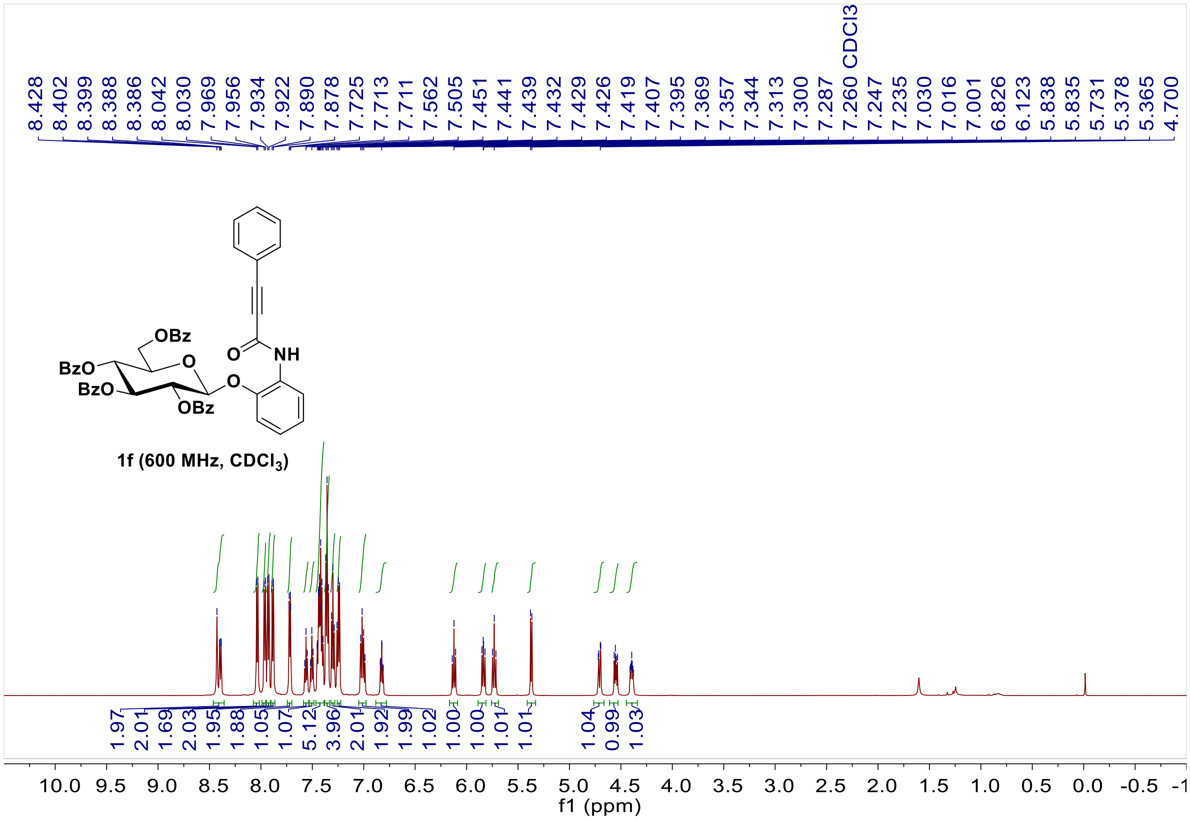

8.428 8.402 8.399 8.388 8.386 8.042 8.030 7.969 7.956 7.934 7.922 7.890 7.878 7.725 7.713 7.711 7.562 7.505 7.451 7.441 7.439 7.432 7.429 7.426 7.419 7.407 7.395 7.369 7.357 7.344 7.313 7.300 7.287 7.260 CDCl3 7.247 7.235 7.030 7.016 7.001 6.826 6.123 5.838 5.835 5.731 5.378 5.365 4.700
OBz
BzO
BzO
O
OBz
O
O
NH
1f (600 MHz, CDCl$_3$)
1.97 2.01 1.69 2.03 1.95 1.88 1.05 1.07 5.12 3.96 2.01 1.92 1.99 1.02 1.00 1.00 1.01 1.01 1.04 0.99 1.03
10.0 9.5 9.0 8.5 8.0 7.5 7.0 6.5 6.0 5.5 5.0 4.5 4.0 3.5 3.0 2.5 2.0 1.5 1.0 0.5 0.0 -0.5 -1
f1 (ppm)

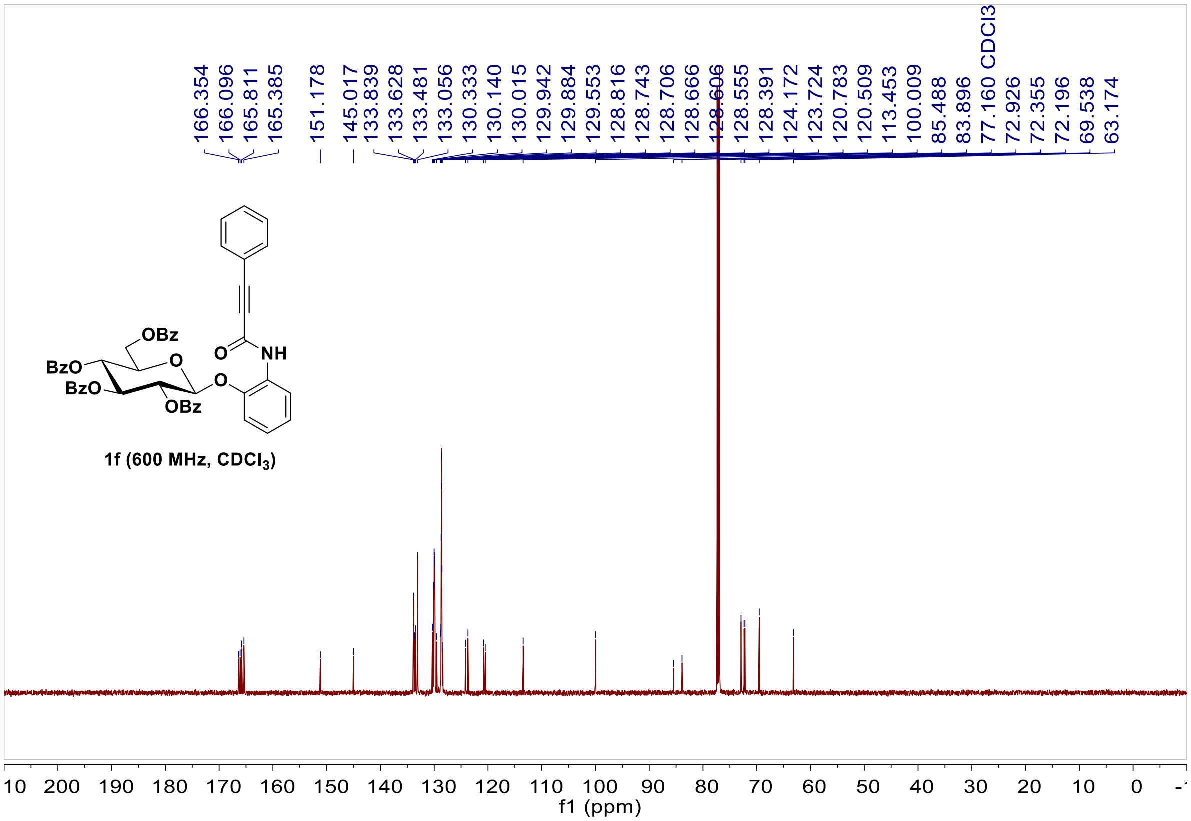

166.354 166.096 165.811 165.385
151.178
145.017 133.839 133.628 133.481 133.056 130.333 130.140 130.015 129.942 129.884 129.553 128.816 128.743 128.706 128.666 128.606 128.555 128.391 124.172 123.724 120.783 120.509 113.453 100.009 85.488 83.896 77.160 CDCl3 72.926 72.355 72.196 69.538 63.174
OBz
BzO
BzO
O
OBz
O
O
NH
1f (600 MHz, CDCl$_3$)
10 200 190 180 170 160 150 140 130 120 110 100 90 80 70 60 50 40 30 20 10 0
f1 (ppm)

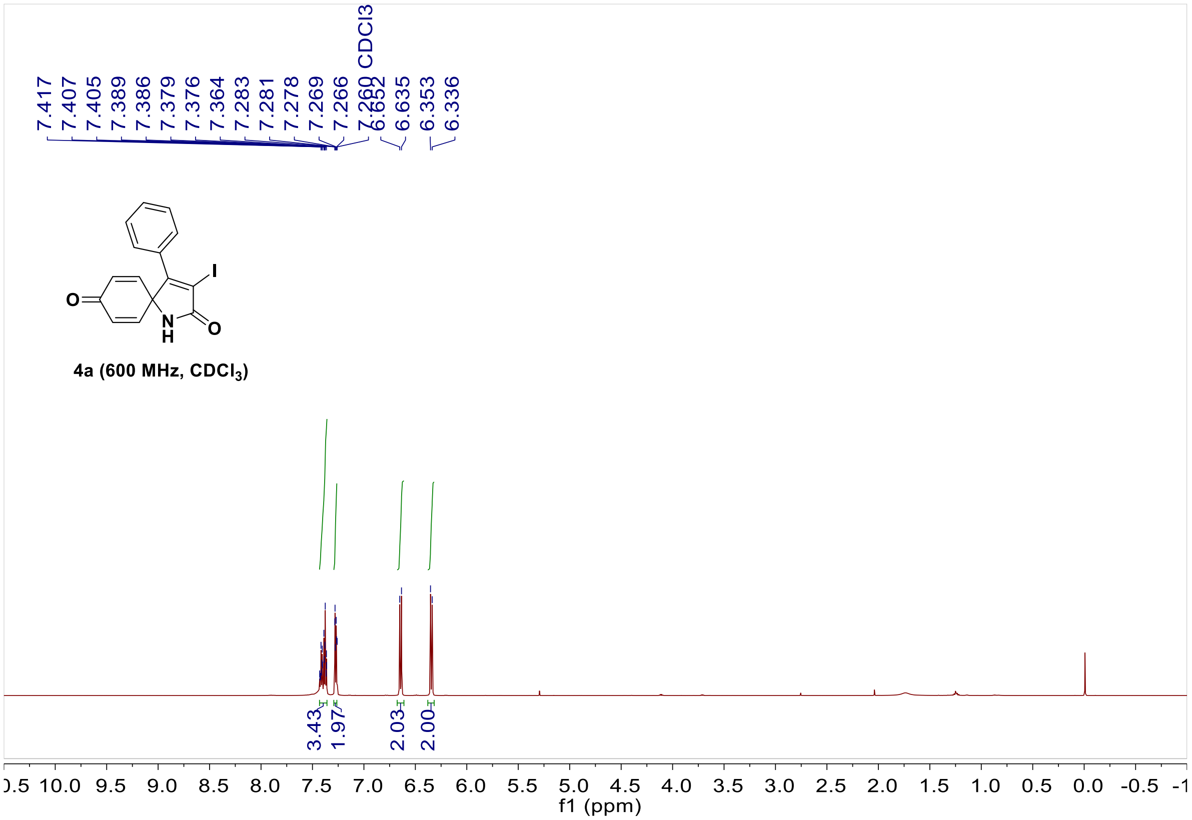
7.417
7.407
7.405
7.389
7.386
7.379
7.376
7.364
7.283
7.281
7.278
7.269
7.266
7.260 CDCl3
6.652
6.635
6.353
6.336
4a (600 MHz, CDCl3)
3.43
1.97
2.03
2.00
f1 (ppm)

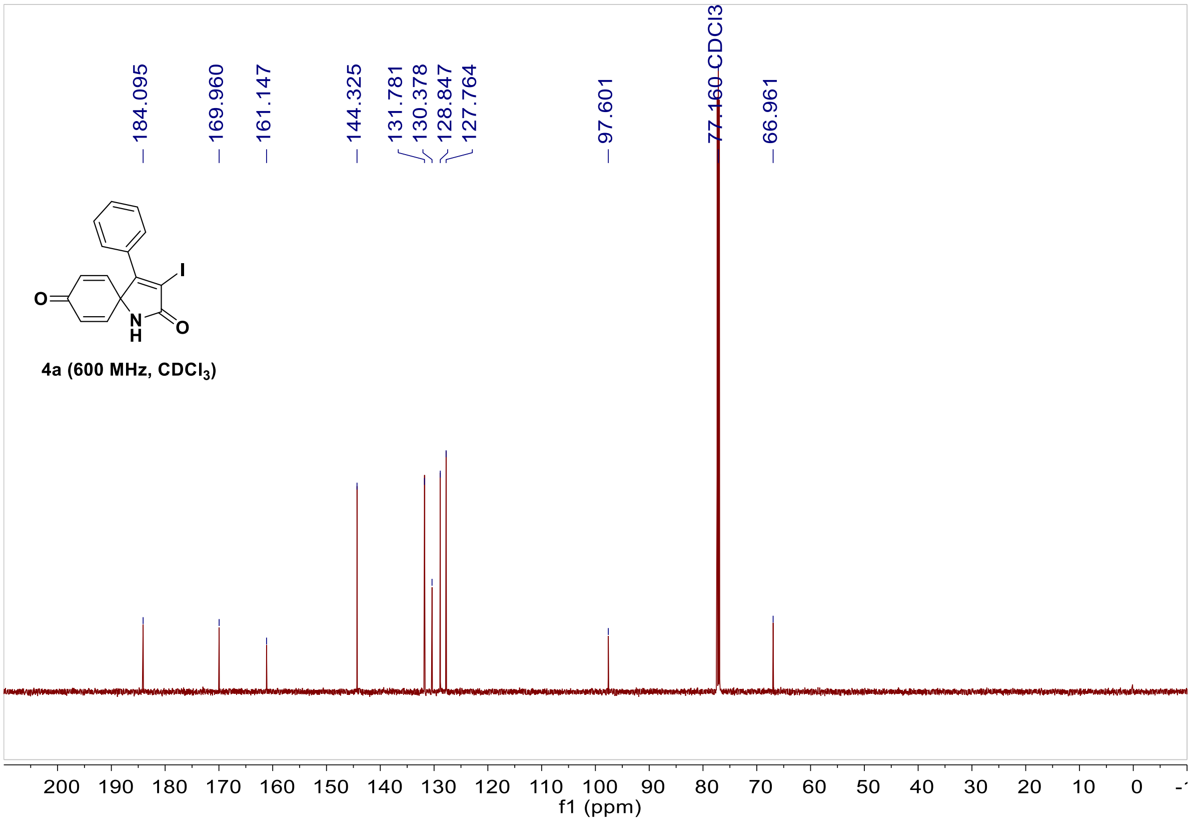
184.095
169.960
161.147
144.325
131.781
130.378
128.847
127.764
97.601
77.160 CDCl3
66.961
4a (600 MHz, CDCl3)
f1 (ppm)

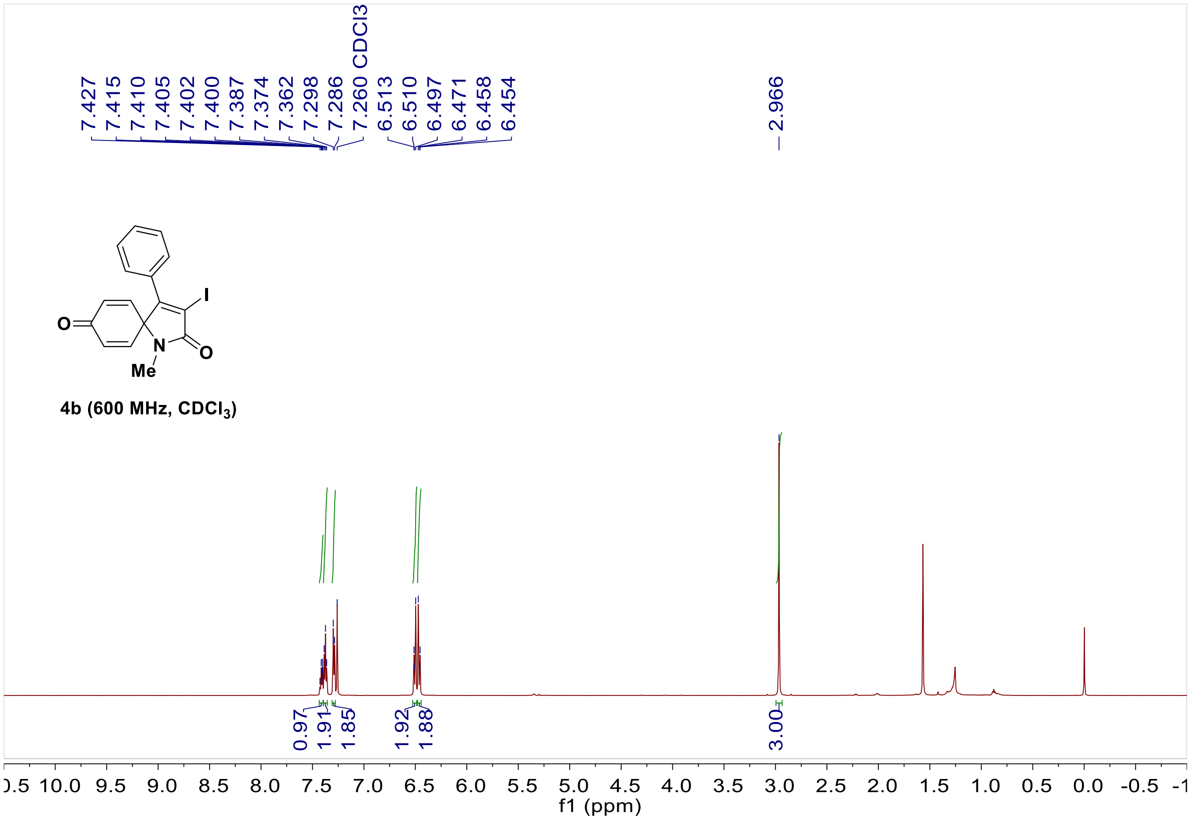

7.427
7.415
7.410
7.405
7.402
7.400
7.387
7.374
7.362
7.298
7.286
7.260 CDCl3
6.513
6.510
6.497
6.471
6.458
6.454
2.966
4b (600 MHz, CDCl$_3$)
0.97
1.91
1.85
1.92
1.88
3.00
f1 (ppm)

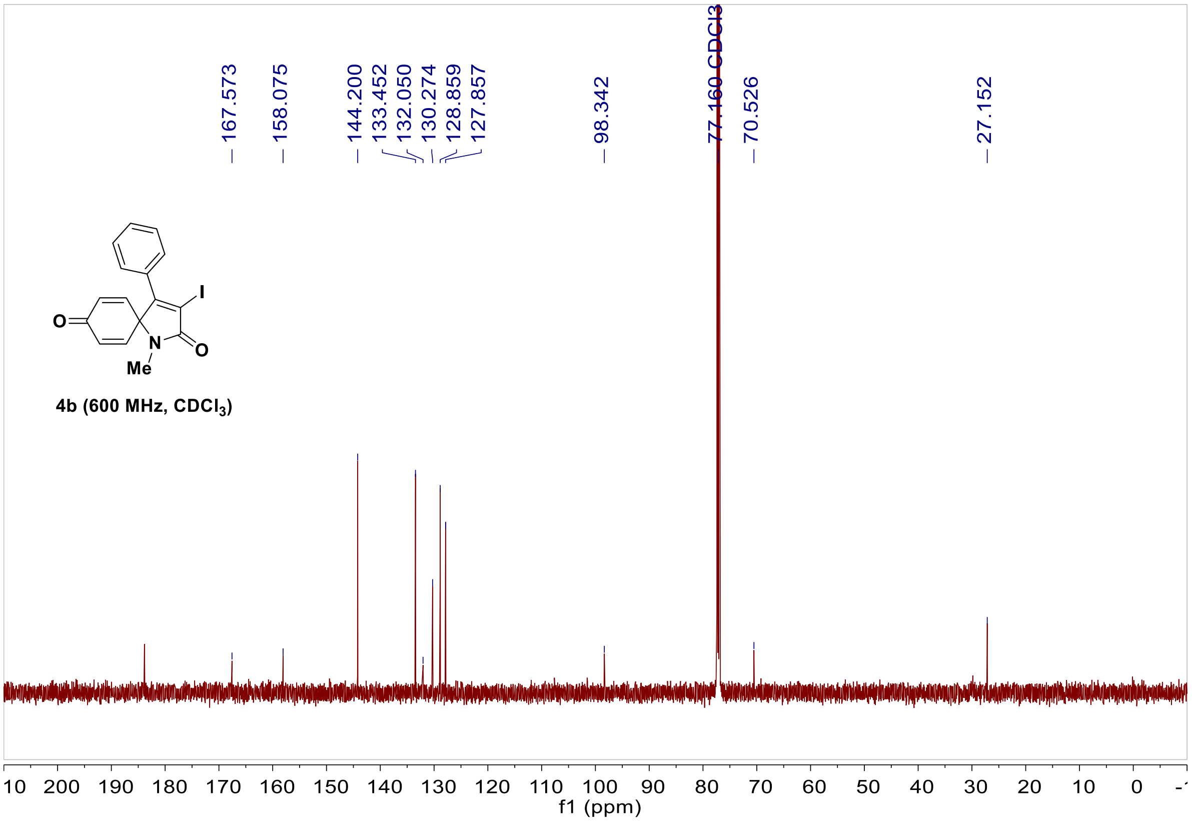

167.573
158.075
144.200
133.452
132.050
130.274
128.859
127.857
98.342
77.160 CDCl3
70.526
27.152
4b (600 MHz, CDCl$_3$)
f1 (ppm)

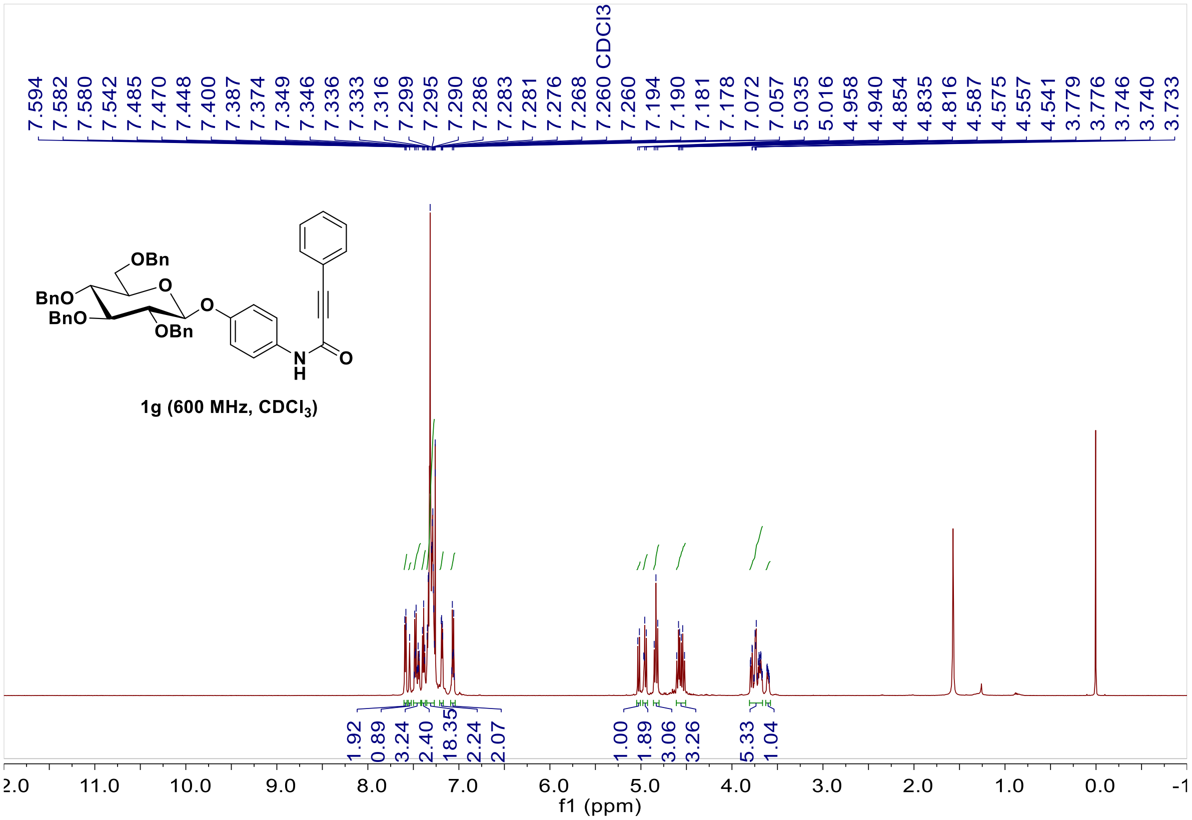
7.594 7.582 7.580 7.542 7.485 7.470 7.448 7.400 7.387 7.374 7.349 7.346 7.336 7.333 7.316 7.299 7.295 7.290 7.286 7.283 7.281 7.276 7.268 7.260 CDCl3 7.260 7.194 7.190 7.181 7.178 7.072 7.057 5.035 5.016 4.958 4.940 4.854 4.835 4.816 4.587 4.575 4.557 4.541 3.779 3.776 3.746 3.740 3.733
BnO
BnO
OBn
OBn
O
O
N
H
O
1g (600 MHz, CDCl3)
1.92 0.89 3.24 2.40 18.35 2.24 2.07
1.00 1.89 3.06 3.26
5.33 1.04
f1 (ppm)
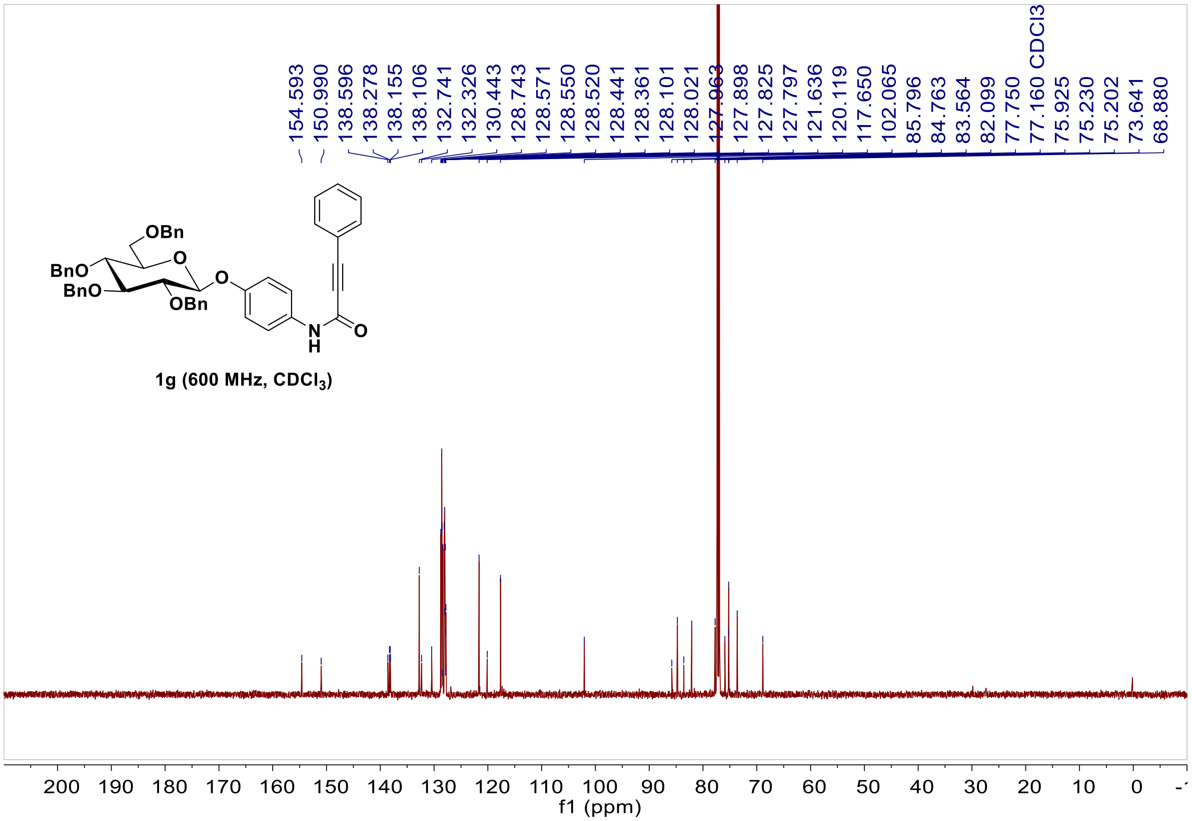
154.593 150.990 138.596 138.278 138.155 138.106 132.741 132.326 130.443 128.743 128.571 128.550 128.520 128.441 128.361 128.101 128.021 127.963 127.898 127.825 127.797 121.636 120.119 117.650 102.065 85.796 84.763 83.564 82.099 77.750 77.160 CDCl3 75.925 75.230 75.202 73.641 68.880
BnO
BnO
OBn
OBn
O
O
N
H
O
1g (600 MHz, CDCl3)
f1 (ppm)

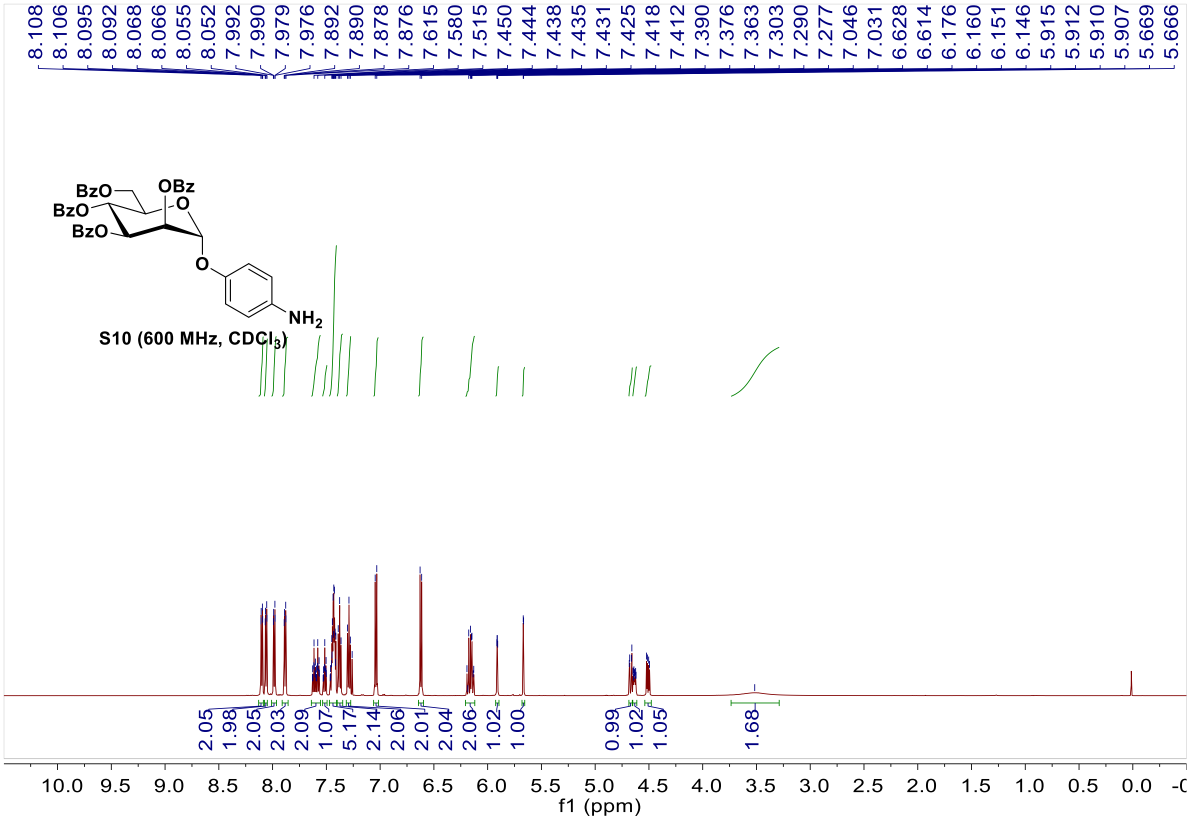
8.108 8.106 8.095 8.092 8.068 8.066 8.055 8.052 7.992 7.990 7.979 7.976 7.892 7.890 7.878 7.876 7.615 7.580 7.515 7.450 7.444 7.438 7.435 7.431 7.425 7.418 7.412 7.390 7.376 7.363 7.303 7.290 7.277 7.046 7.031 6.628 6.614 6.176 6.160 6.151 6.146 5.915 5.912 5.910 5.907 5.669 5.666
BzO
OBz
BzO
O
BzO
O
NH2
S10 (600 MHz, CDCl3)
2.05 1.98 2.05 2.03 2.09 1.07 5.17 2.14 2.06 2.01 2.04 2.06 1.02 1.00 0.99 1.02 1.05 1.68
10.0 9.5 9.0 8.5 8.0 7.5 7.0 6.5 6.0 5.5 5.0 4.5 4.0 3.5 3.0 2.5 2.0 1.5 1.0 0.5 0.0
f1 (ppm)

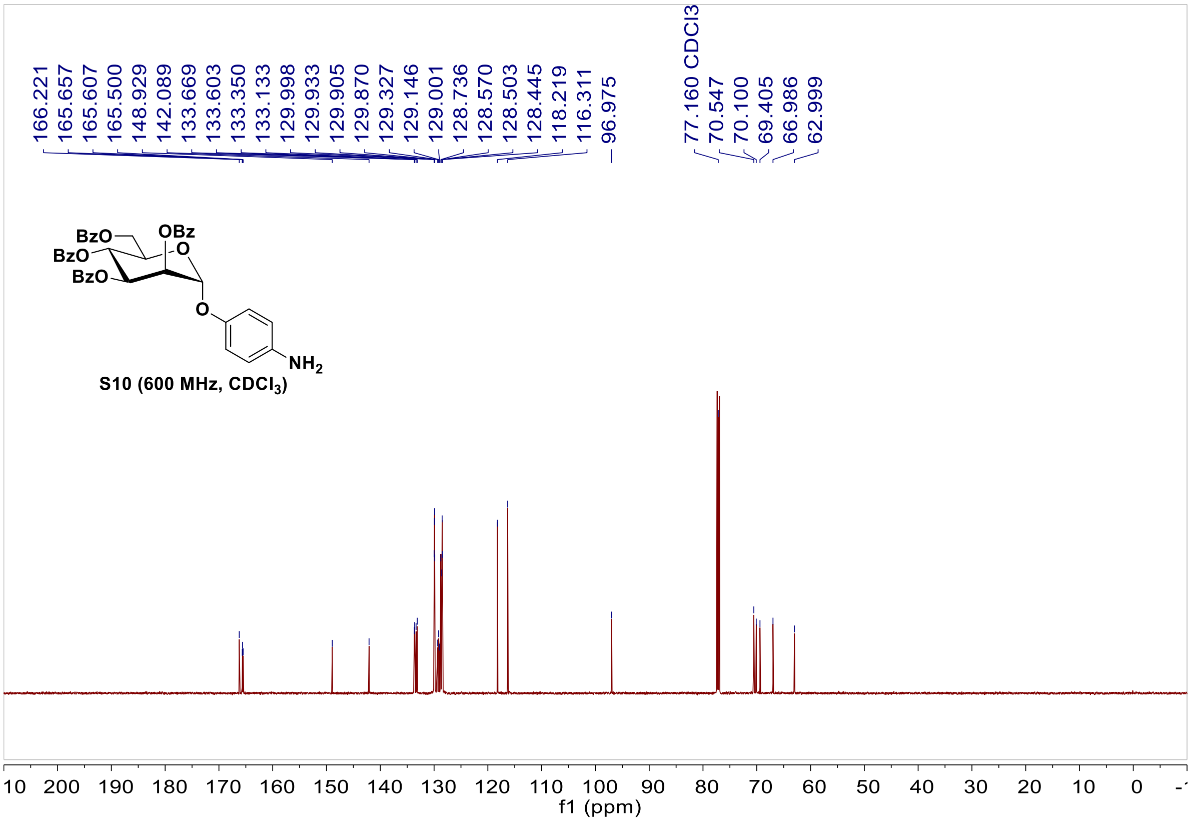
166.221 165.657 165.607 165.500 148.929 142.089 133.669 133.603 133.350 133.133 129.998 129.933 129.905 129.870 129.327 129.146 129.001 128.736 128.570 128.503 128.445 118.219 116.311 96.975
77.160 CDCl3 70.547 70.100 69.405 66.986 62.999
BzO
OBz
BzO
O
BzO
O
NH2
S10 (600 MHz, CDCl3)
200 190 180 170 160 150 140 130 120 110 100 90 80 70 60 50 40 30 20 10 0
f1 (ppm)

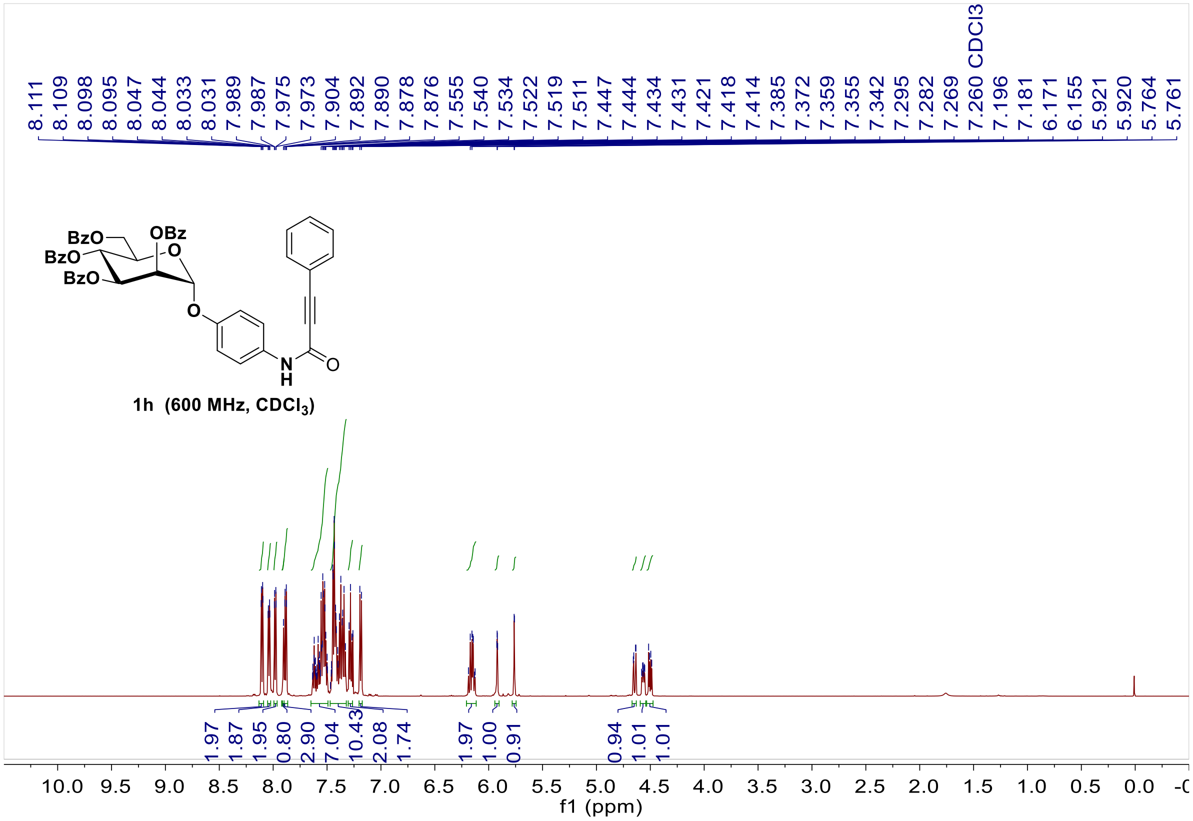
8.111 8.109 8.098 8.095 8.047 8.044 8.033 8.031 7.989 7.987 7.975 7.973 7.904 7.892 7.890 7.878 7.876 7.555 7.540 7.534 7.522 7.519 7.511 7.447 7.444 7.434 7.431 7.421 7.418 7.414 7.385 7.372 7.359 7.355 7.342 7.295 7.282 7.269 7.260 CDCl3 7.196 7.181 6.171 6.155 5.921 5.920 5.764 5.761
BzO OBz BzO BzO O O N H O
1h (600 MHz, CDCl$_3$)
1.97 1.87 1.95 0.80 2.90 7.04 10.43 2.08 1.74 1.97 1.00 0.91 0.94 1.01 1.01
10.0 9.5 9.0 8.5 8.0 7.5 7.0 6.5 6.0 5.5 5.0 4.5 4.0 3.5 3.0 2.5 2.0 1.5 1.0 0.5 0.0
f1 (ppm)

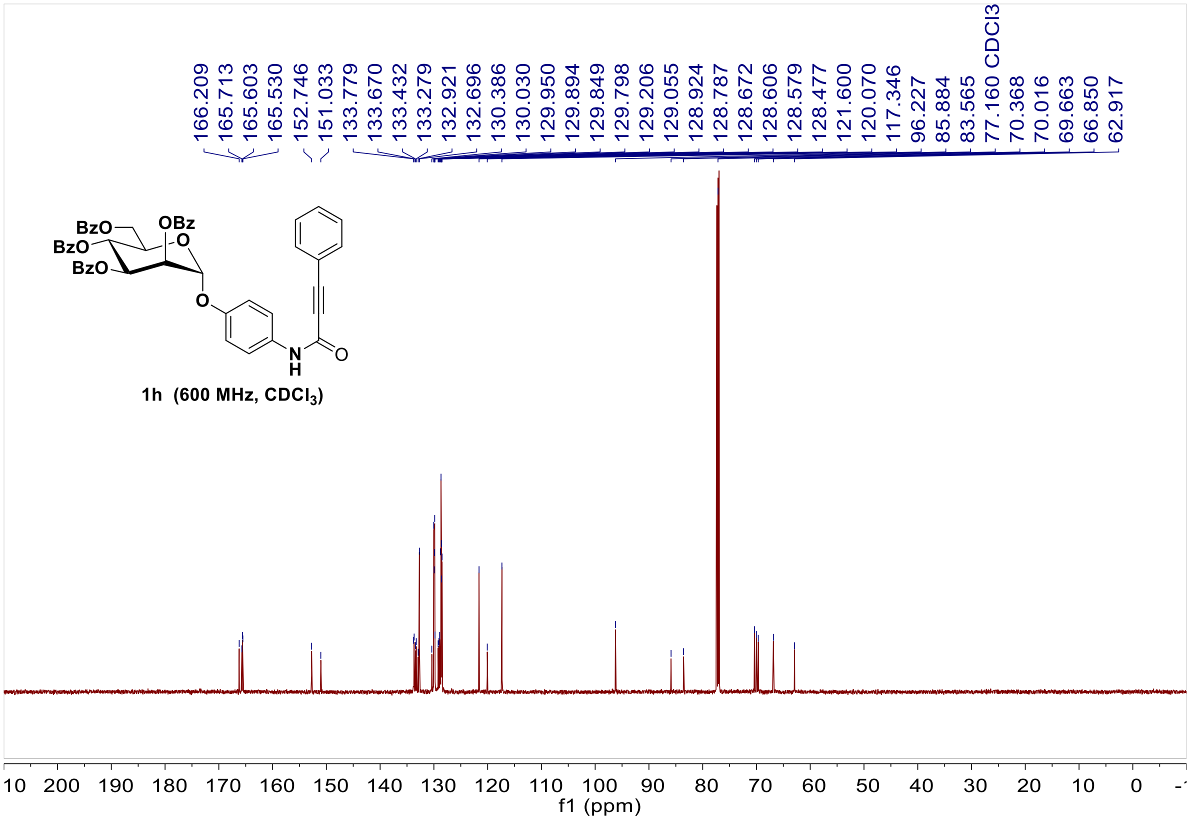
166.209 165.713 165.603 165.530 152.746 151.033 133.779 133.670 133.432 133.279 132.921 132.696 130.386 130.030 129.950 129.894 129.849 129.798 129.206 129.055 128.924 128.787 128.672 128.606 128.579 128.477 121.600 120.070 117.346 96.227 85.884 83.565 77.160 CDCl3 70.368 70.016 69.663 66.850 62.917
BzO OBz BzO BzO O O N H O
1h (600 MHz, CDCl$_3$)
10 200 190 180 170 160 150 140 130 120 110 100 90 80 70 60 50 40 30 20 10 0
f1 (ppm)

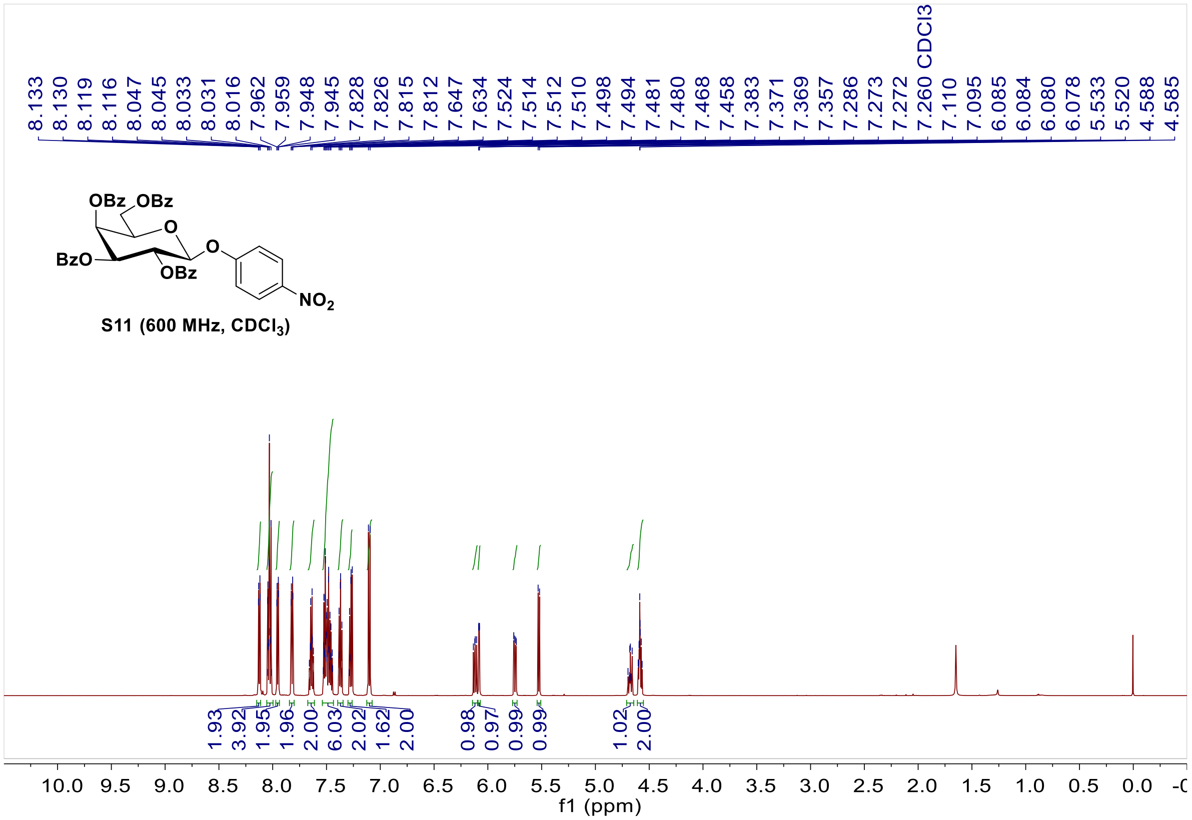
8.133 8.130 8.119 8.116 8.047 8.045 8.033 8.031 8.016 7.962 7.959 7.948 7.945 7.828 7.826 7.815 7.812 7.647 7.634 7.524 7.514 7.512 7.510 7.498 7.494 7.481 7.480 7.468 7.458 7.383 7.371 7.369 7.357 7.286 7.273 7.272 7.260 CDCl3 7.110 7.095 6.085 6.084 6.080 6.078 5.533 5.520 4.588 4.585
OBz OBz
BzO
OBz
NO2
S11 (600 MHz, CDCl3)
1.93 3.92 1.95 1.96 2.00 6.03 2.02 1.62 2.00 0.98 0.97 0.99 0.99 1.02 2.00
f1 (ppm)

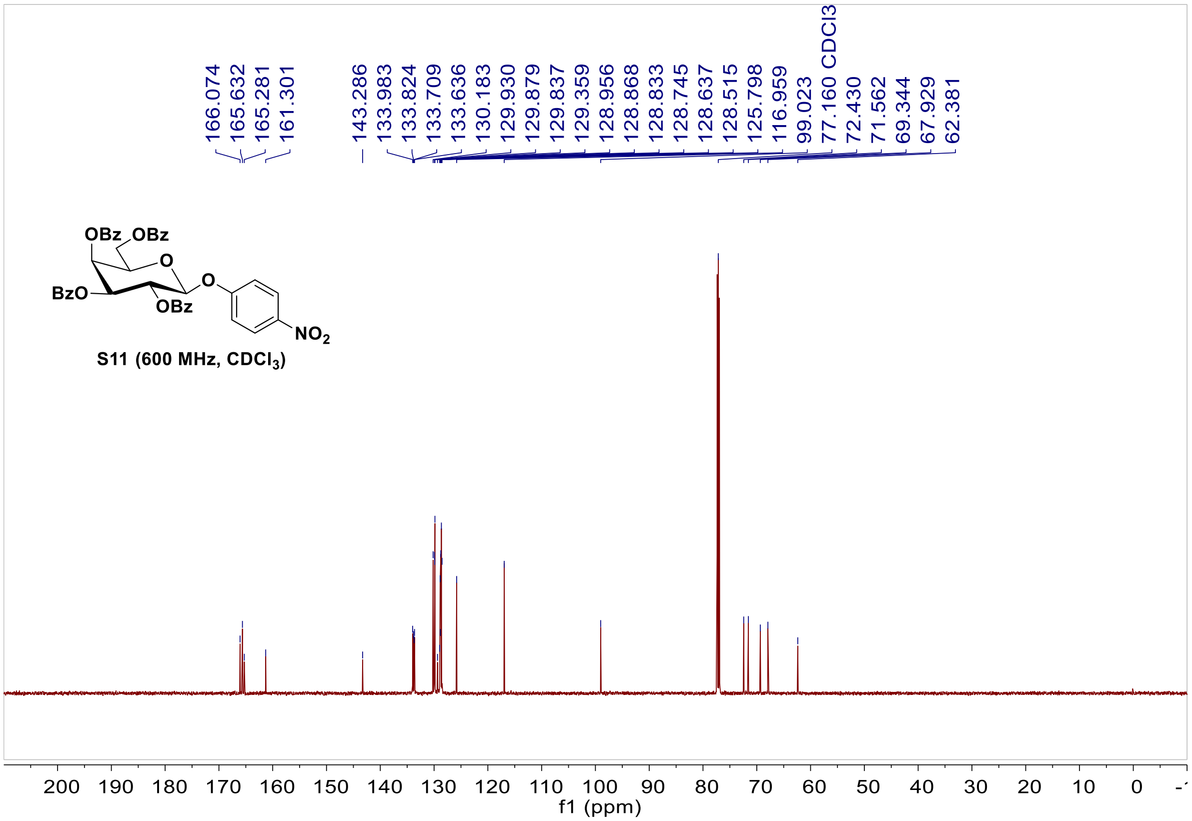
166.074 165.632 165.281 161.301 143.286 133.983 133.824 133.709 133.636 130.183 129.930 129.879 129.837 129.359 128.956 128.868 128.833 128.745 128.637 128.515 125.798 116.959 99.023 77.160 CDCl3 72.430 71.562 69.344 67.929 62.381
OBz OBz
BzO
OBz
NO2
S11 (600 MHz, CDCl3)
f1 (ppm)

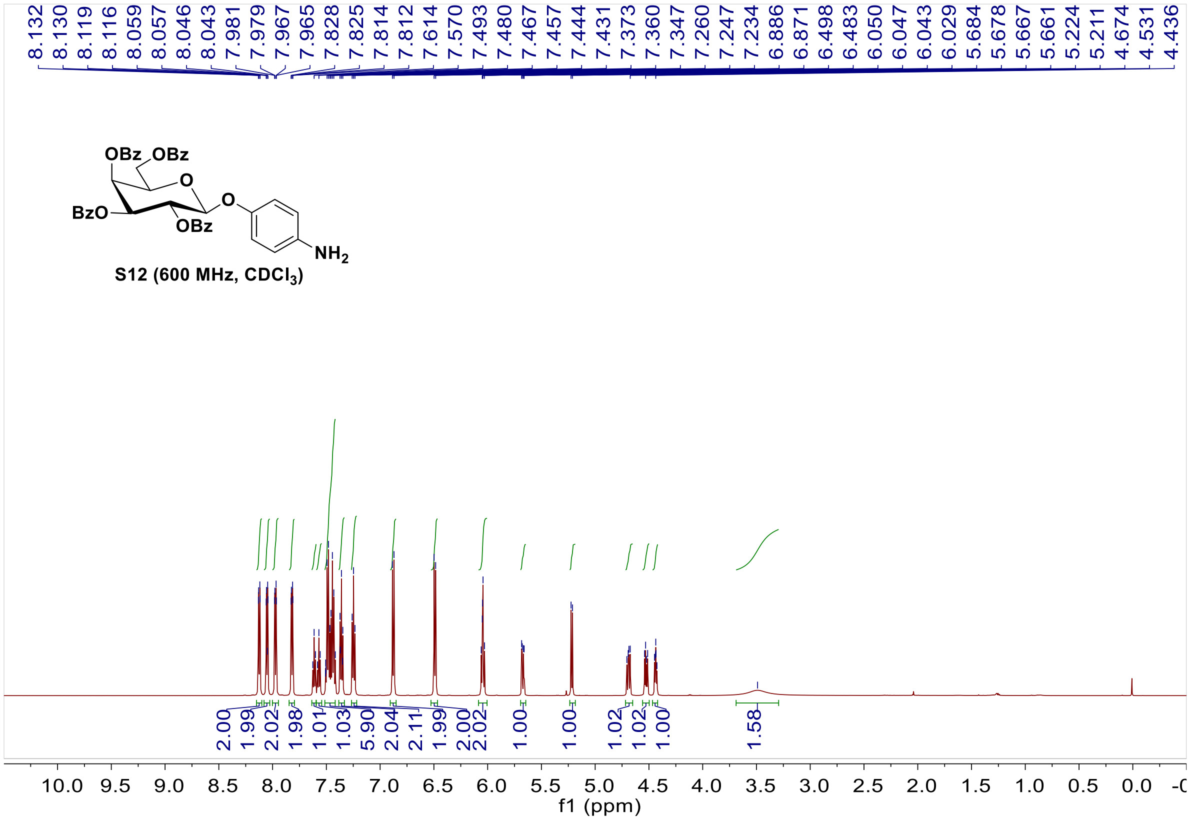
8.132
8.130
8.119
8.116
8.059
8.057
8.046
8.043
7.981
7.979
7.967
7.965
7.828
7.825
7.814
7.812
7.614
7.570
7.493
7.480
7.467
7.457
7.444
7.431
7.373
7.360
7.347
7.260
7.247
7.234
6.886
6.871
6.498
6.483
6.050
6.047
6.043
6.029
5.684
5.678
5.667
5.661
5.224
5.211
4.674
4.531
4.436
OBz OBz
O
BzO
OBz
O
NH2
S12 (600 MHz, CDCl3)
2.00
1.99
2.02
1.98
1.01
1.03
5.90
2.04
2.11
1.99
2.00
2.02
1.00
1.00
1.02
1.02
1.00
1.58
10.0 9.5 9.0 8.5 8.0 7.5 7.0 6.5 6.0 5.5 5.0 4.5 4.0 3.5 3.0 2.5 2.0 1.5 1.0 0.5 0.0
f1 (ppm)

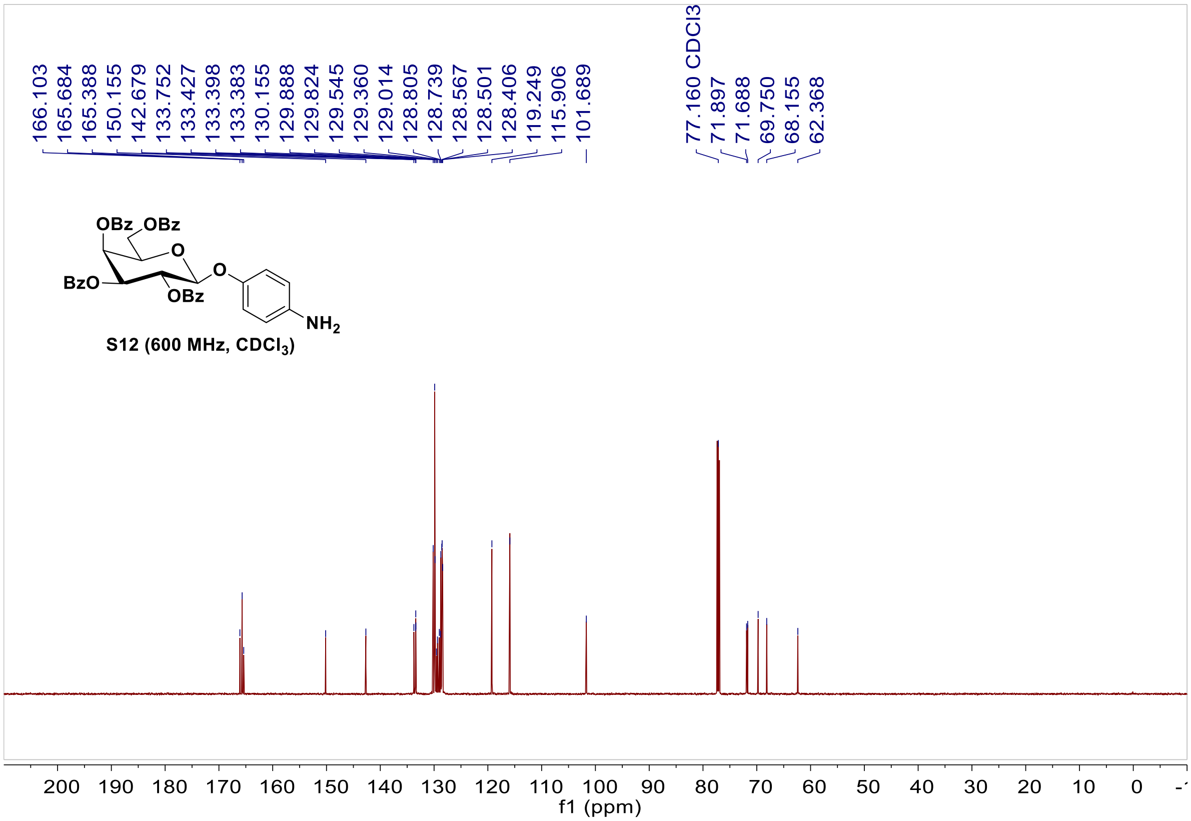
166.103
165.684
165.388
150.155
142.679
133.752
133.427
133.398
133.383
130.155
129.888
129.824
129.545
129.360
129.014
128.805
128.739
128.567
128.501
128.406
119.249
115.906
101.689
77.160 CDCl3
71.897
71.688
69.750
68.155
62.368
OBz OBz
O
BzO
OBz
O
NH2
S12 (600 MHz, CDCl3)
200 190 180 170 160 150 140 130 120 110 100 90 80 70 60 50 40 30 20 10 0
f1 (ppm)

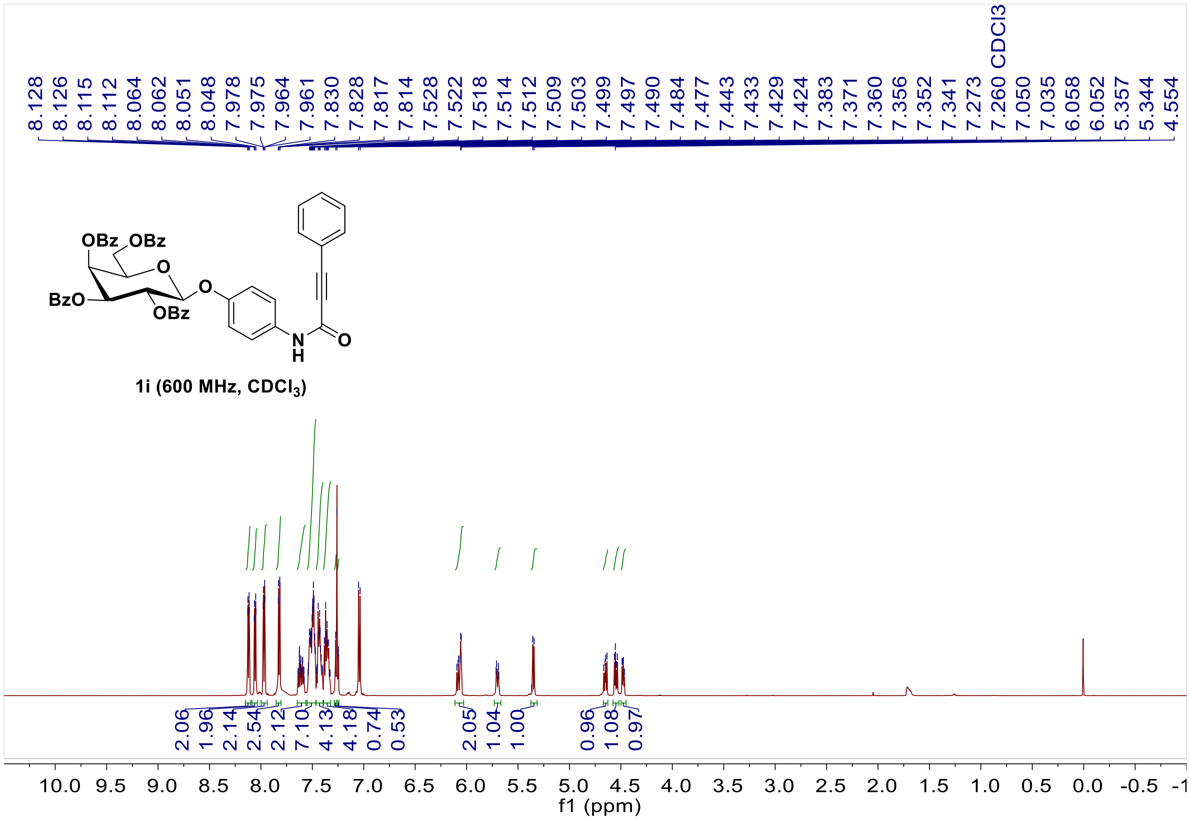
8.128 8.126 8.115 8.112 8.064 8.062 8.051 8.048 7.978 7.975 7.964 7.961 7.830 7.828 7.817 7.814 7.528 7.522 7.518 7.514 7.512 7.509 7.503 7.499 7.497 7.490 7.484 7.477 7.443 7.433 7.429 7.424 7.383 7.371 7.360 7.356 7.352 7.341 7.273 7.260 CDCl3 7.050 7.035 6.058 6.052 5.357 5.344 4.554
OBz OBz
O
BzO
OBz
O
N
H
O
1i (600 MHz, $CDCl_3$)
2.06 1.96 2.14 2.54 2.12 7.10 4.13 4.18 0.74 0.53 2.05 1.04 1.00 0.96 1.08 0.97
10.0 9.5 9.0 8.5 8.0 7.5 7.0 6.5 6.0 5.5 5.0 4.5 4.0 3.5 3.0 2.5 2.0 1.5 1.0 0.5 0.0 -0.5 -1
f1 (ppm)

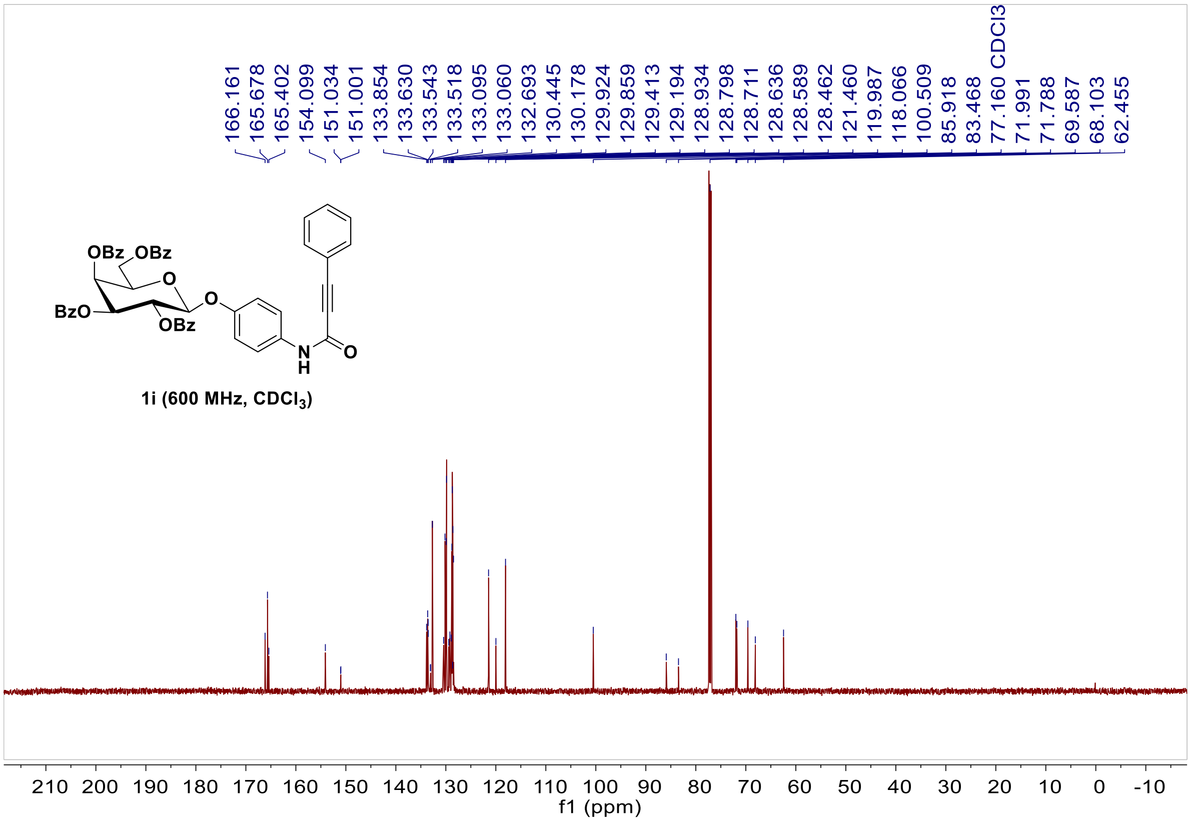
166.161 165.678 165.402 154.099 151.034 151.001 133.854 133.630 133.543 133.518 133.095 133.060 132.693 130.445 130.178 129.924 129.859 129.413 129.194 128.934 128.798 128.711 128.636 128.589 128.462 121.460 119.987 118.066 100.509 85.918 83.468 77.160 CDCl3 71.991 71.788 69.587 68.103 62.455
OBz OBz
O
BzO
OBz
O
N
H
O
1i (600 MHz, $CDCl_3$)
210 200 190 180 170 160 150 140 130 120 110 100 90 80 70 60 50 40 30 20 10 0 -10
f1 (ppm)

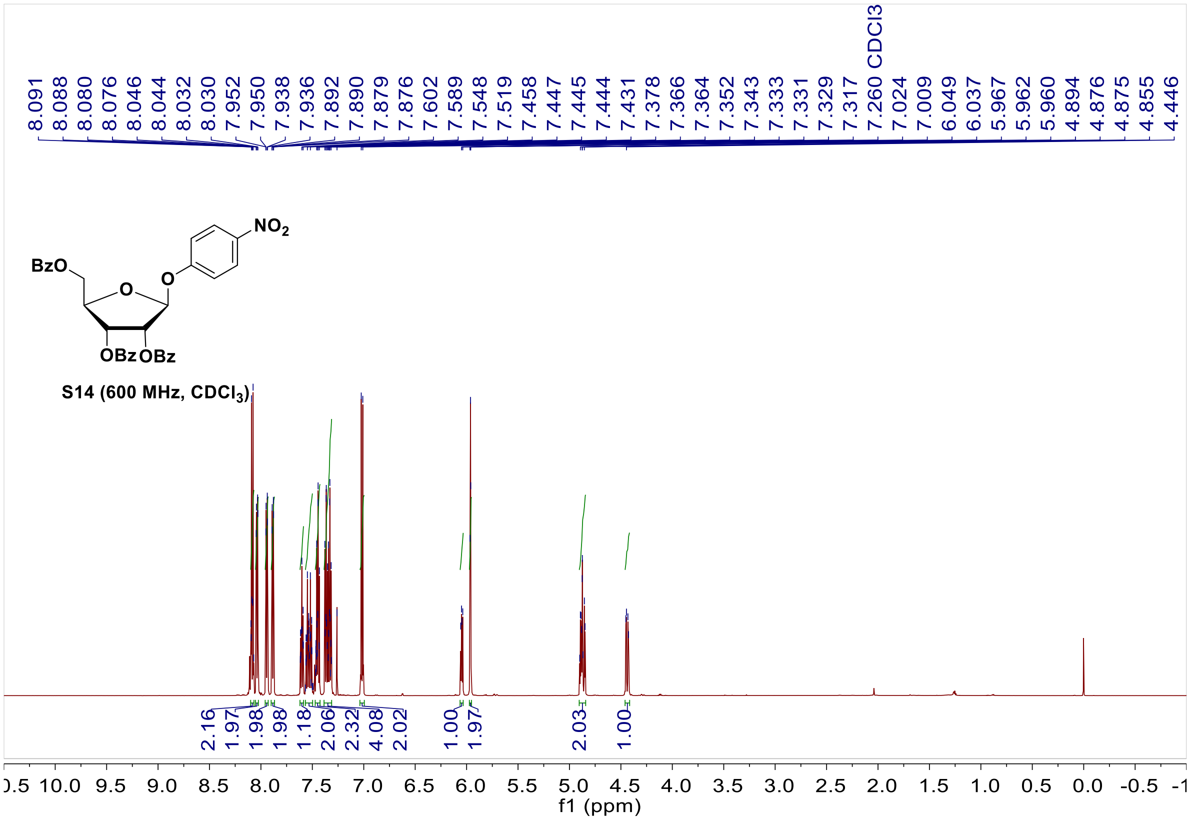
8.091
8.088
8.080
8.076
8.046
8.044
8.032
8.030
7.952
7.950
7.938
7.936
7.892
7.890
7.879
7.876
7.602
7.589
7.548
7.519
7.458
7.447
7.445
7.444
7.431
7.378
7.366
7.364
7.352
7.343
7.333
7.331
7.329
7.317
7.260 CDCl3
7.024
7.009
6.049
6.037
5.967
5.962
5.960
4.894
4.876
4.875
4.855
4.446
NO2
BzO
O
O
OBz OBz
S14 (600 MHz, CDCl3)
2.16
1.97
1.98
1.98
1.18
2.06
2.32
4.08
2.02
1.00
1.97
2.03
1.00
f1 (ppm)

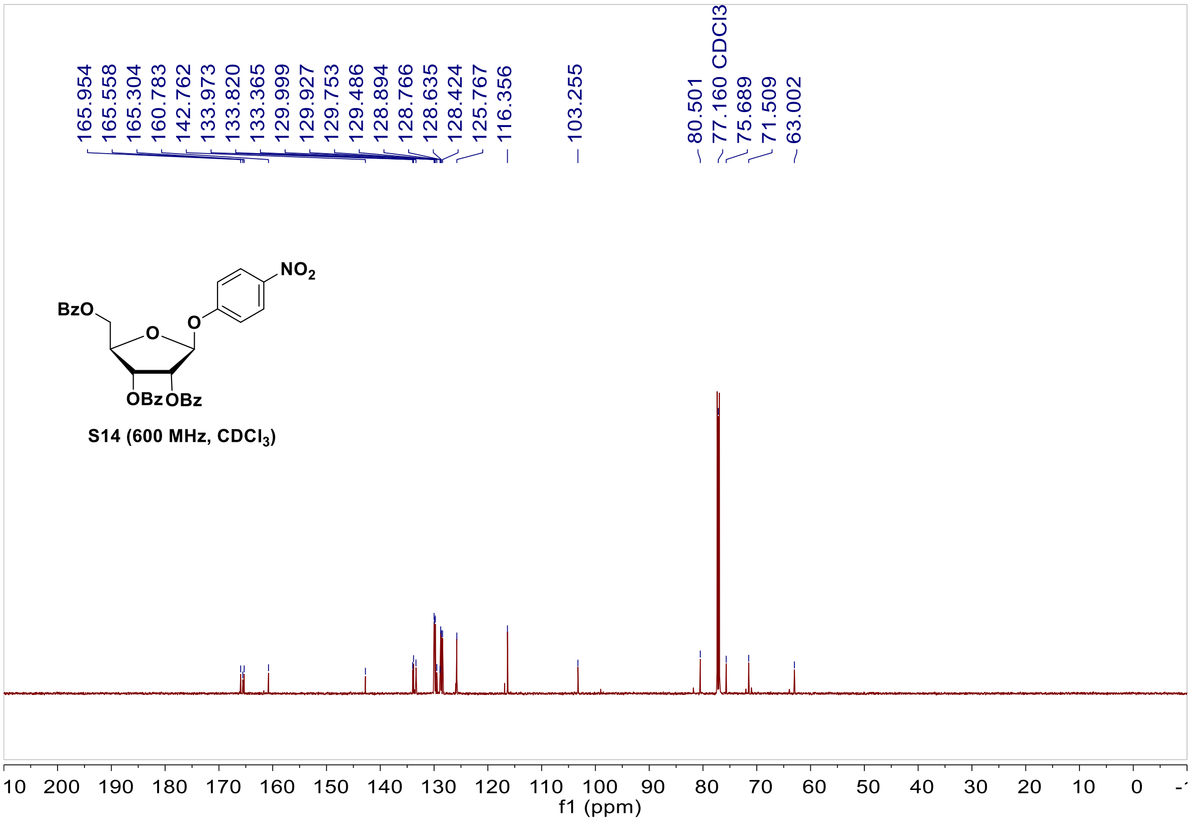
165.954
165.558
165.304
160.783
142.762
133.973
133.820
133.365
129.999
129.927
129.753
129.486
128.894
128.766
128.635
128.424
125.767
116.356
103.255
80.501
77.160 CDCl3
75.689
71.509
63.002
NO2
BzO
O
O
OBz OBz
S14 (600 MHz, CDCl3)
f1 (ppm)

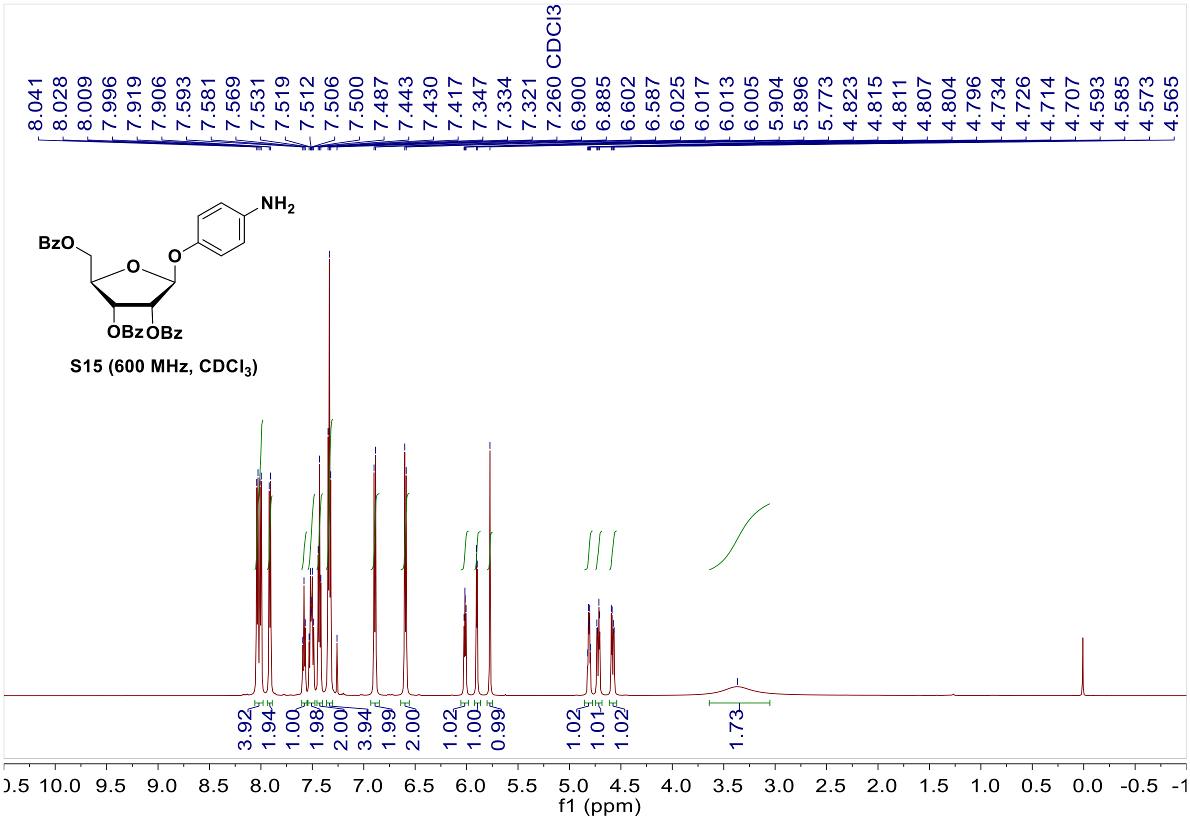
8.041
8.028
8.009
7.996
7.919
7.906
7.593
7.581
7.569
7.531
7.519
7.512
7.506
7.500
7.487
7.443
7.430
7.417
7.347
7.334
7.321
7.260 CDCl3
6.900
6.885
6.602
6.587
6.025
6.017
6.013
6.005
5.904
5.896
5.773
4.823
4.815
4.811
4.807
4.804
4.796
4.734
4.726
4.714
4.707
4.593
4.585
4.573
4.565
NH2
BzO
O
O
OBz OBz
S15 (600 MHz, CDCl3)
3.92
1.94
1.00
1.98
2.00
3.94
1.99
2.00
1.02
1.00
0.99
1.02
1.01
1.02
1.73
f1 (ppm)

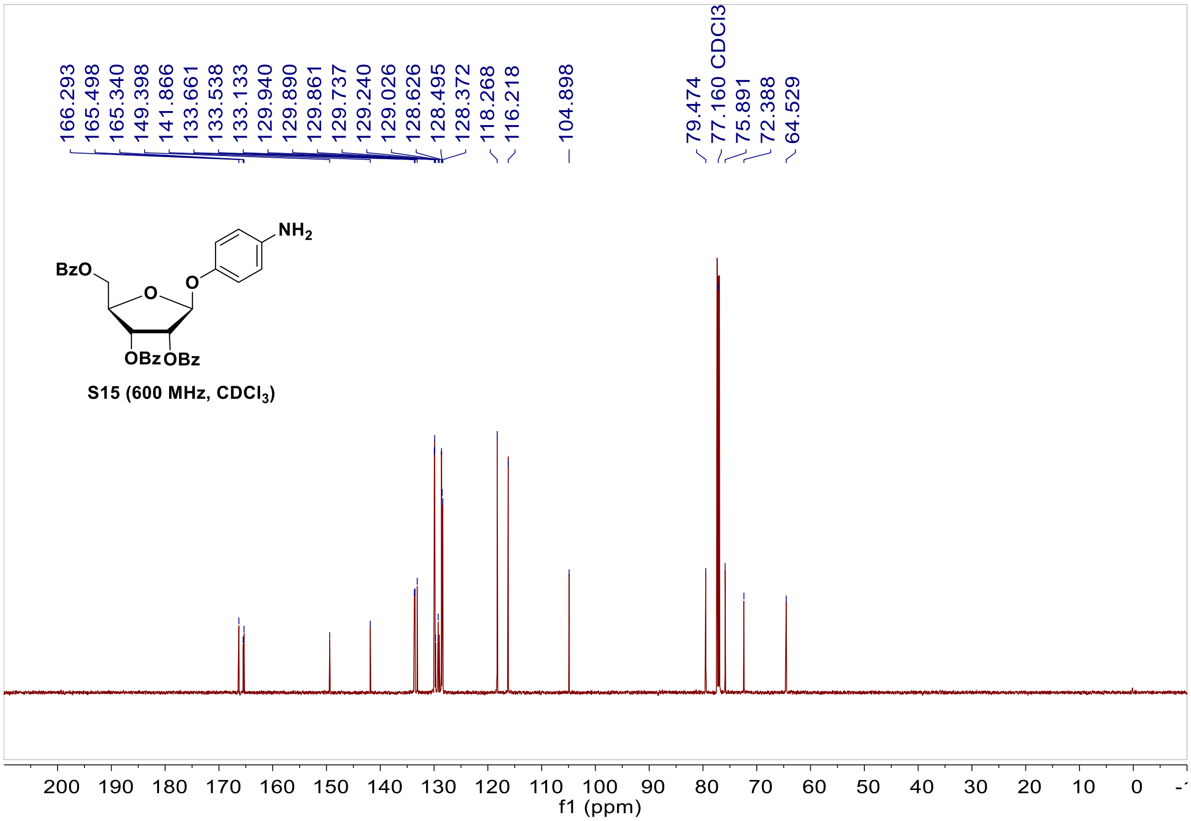
166.293
165.498
165.340
149.398
141.866
133.661
133.538
133.133
129.940
129.890
129.861
129.737
129.240
129.026
128.626
128.495
128.372
118.268
116.218
104.898
79.474
77.160 CDCl3
75.891
72.388
64.529
NH2
BzO
O
O
OBz OBz
S15 (600 MHz, CDCl3)
f1 (ppm)

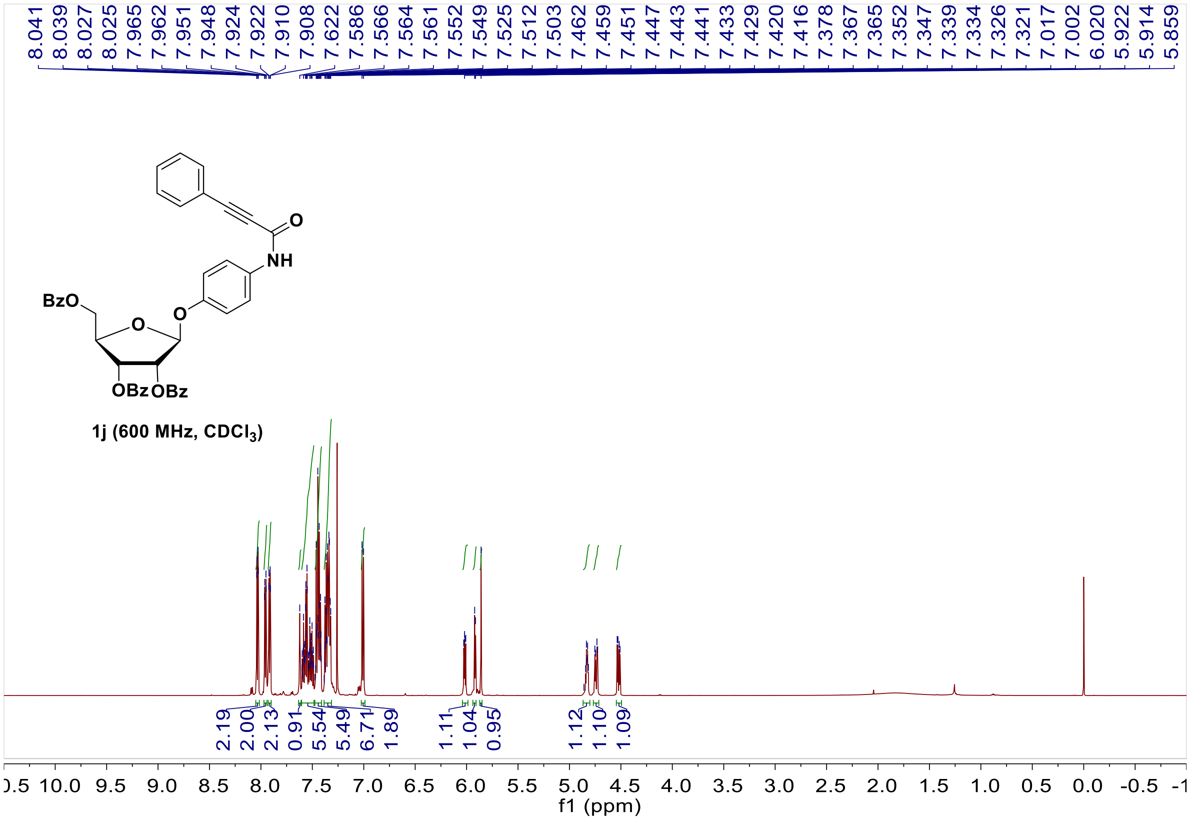
8.041 8.039 8.027 8.025 7.965 7.962 7.951 7.948 7.924 7.922 7.910 7.908 7.622 7.586 7.564 7.561 7.552 7.549 7.525 7.512 7.503 7.462 7.459 7.451 7.447 7.443 7.441 7.433 7.429 7.420 7.416 7.378 7.367 7.365 7.352 7.347 7.339 7.334 7.326 7.321 7.017 7.002 6.020 5.922 5.914 5.859
BzO
O
O
NH
O
OBz OBz
1j (600 MHz, CDCl3)
2.19 2.00 2.13 0.91 5.54 5.49 6.71 1.89 1.11 1.04 0.95 1.12 1.10 1.09
10.5 10.0 9.5 9.0 8.5 8.0 7.5 7.0 6.5 6.0 5.5 5.0 4.5 4.0 3.5 3.0 2.5 2.0 1.5 1.0 0.5 0.0 -0.5 -1
f1 (ppm)

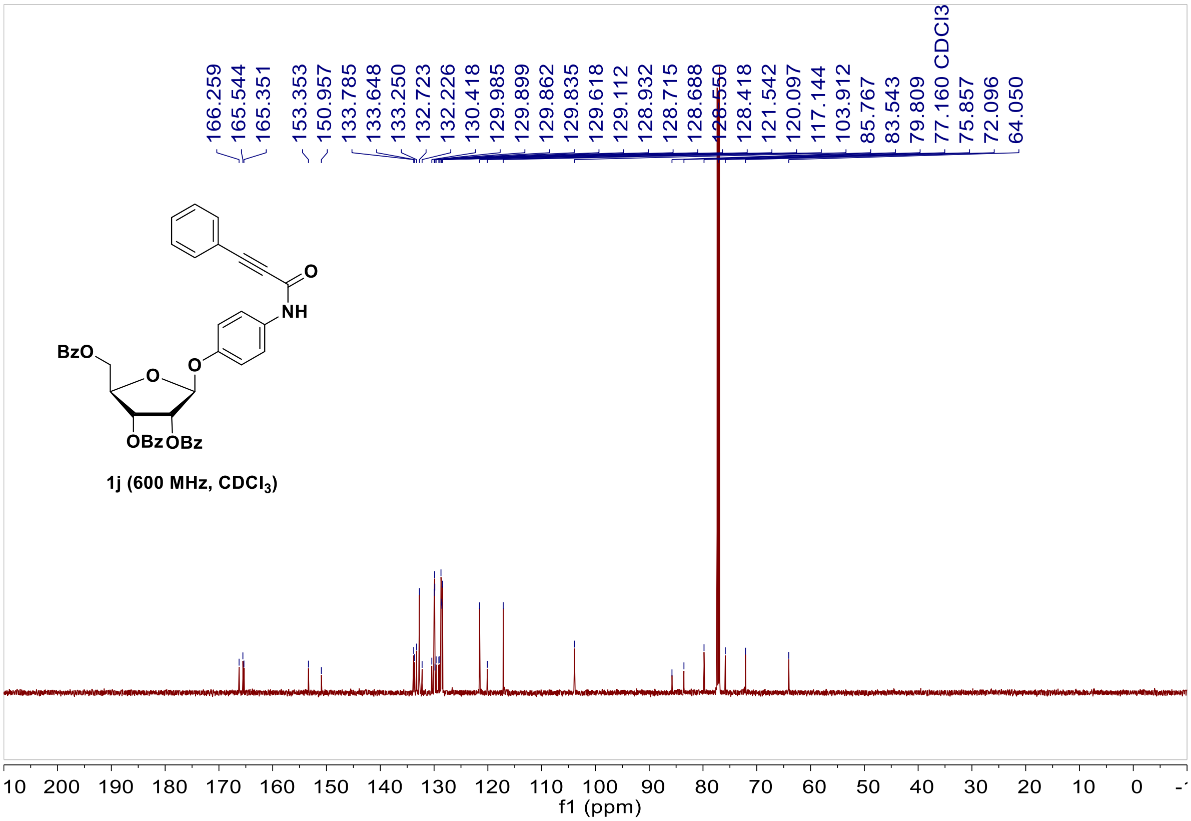
166.259 165.544 165.351 153.353 150.957 133.785 133.648 133.250 132.723 132.226 130.418 129.985 129.899 129.862 129.835 129.618 129.112 128.932 128.715 128.688 128.550 128.418 121.542 120.097 117.144 103.912 85.767 83.543 79.809 77.160 CDCl3 75.857 72.096 64.050
BzO
O
O
NH
O
OBz OBz
1j (600 MHz, CDCl3)
210 200 190 180 170 160 150 140 130 120 110 100 90 80 70 60 50 40 30 20 10 0
f1 (ppm)

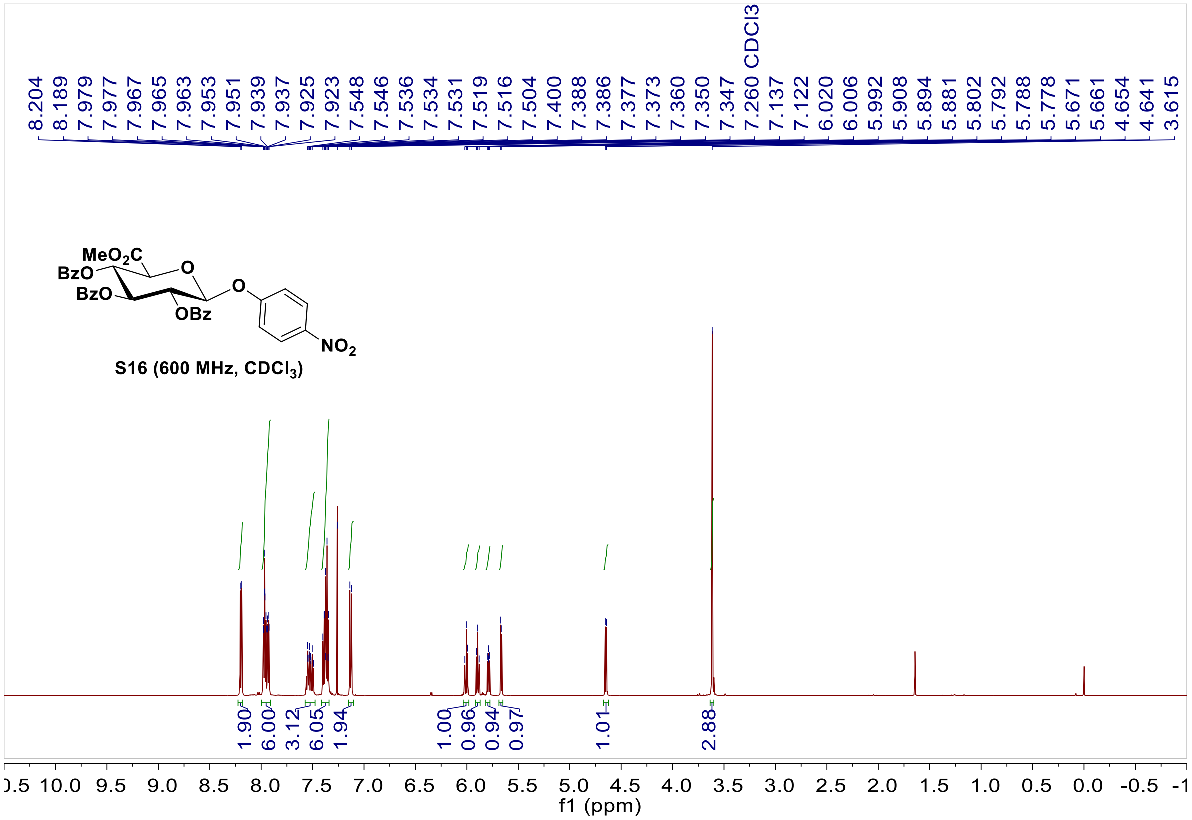
8.204 8.189 7.979 7.977 7.967 7.965 7.963 7.953 7.951 7.939 7.937 7.925 7.923 7.548 7.546 7.536 7.534 7.531 7.519 7.516 7.504 7.400 7.388 7.386 7.377 7.373 7.360 7.350 7.347 7.260 CDCl3 7.137 7.122 6.020 6.006 5.992 5.908 5.894 5.881 5.802 5.792 5.788 5.778 5.671 5.661 4.654 4.641 3.615
MeO2C
BzO
BzO
O
O
OBz
NO2
S16 (600 MHz, CDCl3)
1.90 6.00 3.12 6.05 1.94 1.00 0.96 0.94 0.97 1.01 2.88
f1 (ppm)

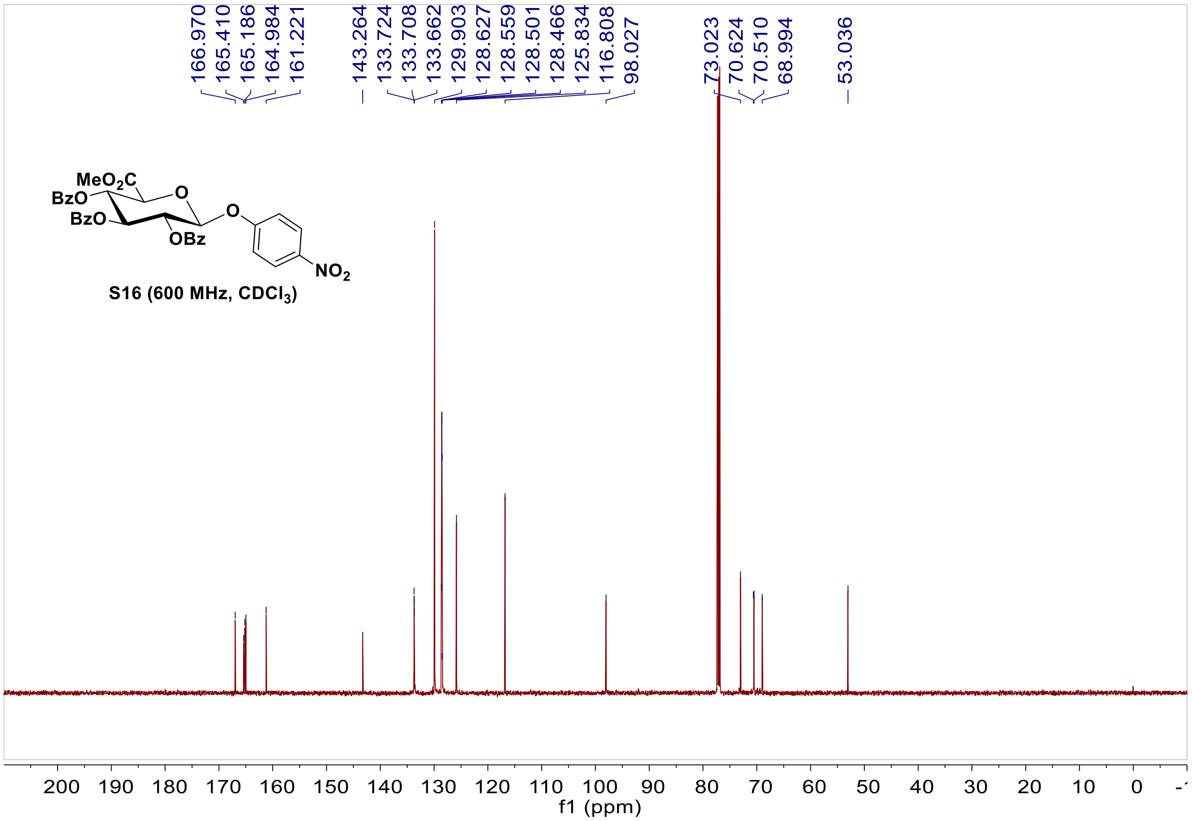
166.970 165.410 165.186 164.984 161.221 143.264 133.724 133.708 133.662 129.903 128.627 128.559 128.501 128.466 125.834 116.808 98.027 73.023 70.624 70.510 68.994 53.036
MeO2C
BzO
BzO
O
O
OBz
NO2
S16 (600 MHz, CDCl3)
f1 (ppm)

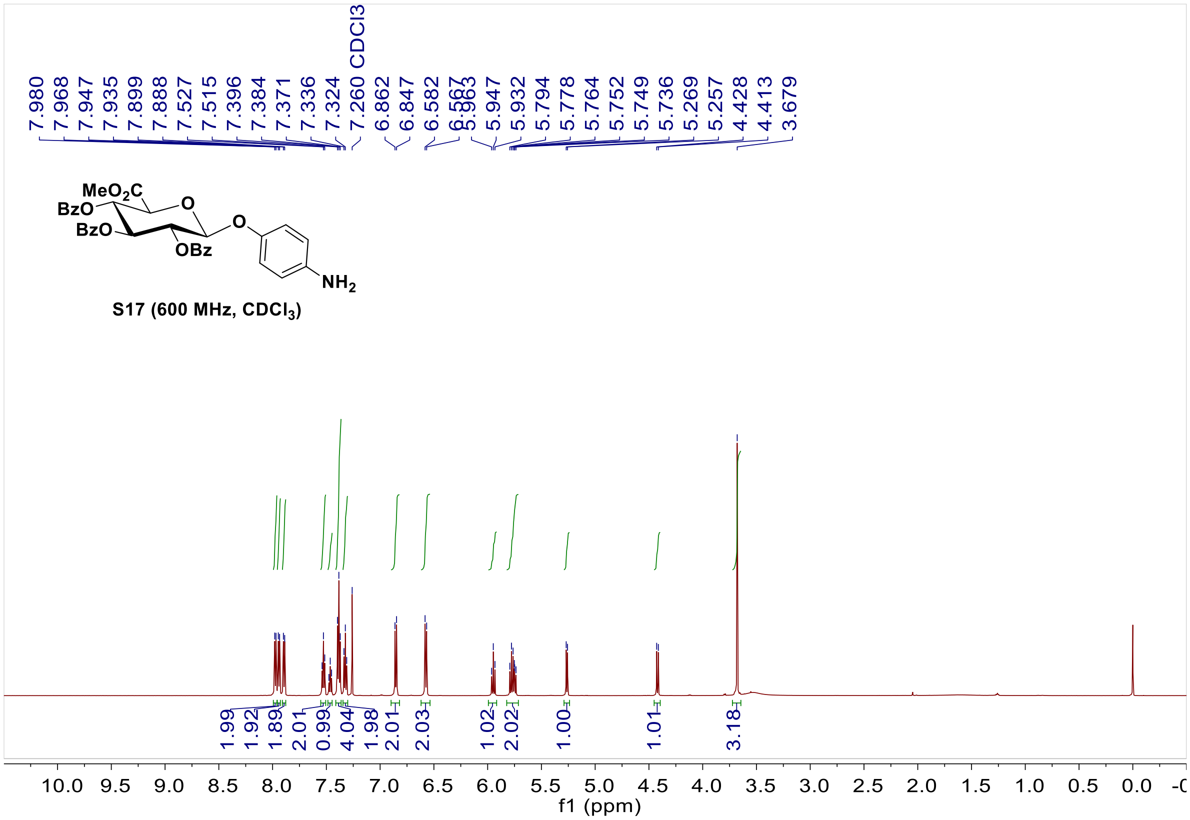
7.980
7.968
7.947
7.935
7.899
7.888
7.527
7.515
7.396
7.384
7.371
7.336
7.324
7.260 CDCl3
6.862
6.847
6.582
6.567
5.963
5.947
5.932
5.794
5.778
5.764
5.752
5.749
5.736
5.269
5.257
4.428
4.413
3.679
MeO2C
BzO
O
BzO
OBz
O
NH2
S17 (600 MHz, CDCl3)
1.99
1.92
1.89
2.01
0.99
4.04
1.98
2.01
2.03
1.02
2.02
1.00
1.01
3.18
10.0 9.5 9.0 8.5 8.0 7.5 7.0 6.5 6.0 5.5 5.0 4.5 4.0 3.5 3.0 2.5 2.0 1.5 1.0 0.5 0.0
f1 (ppm)

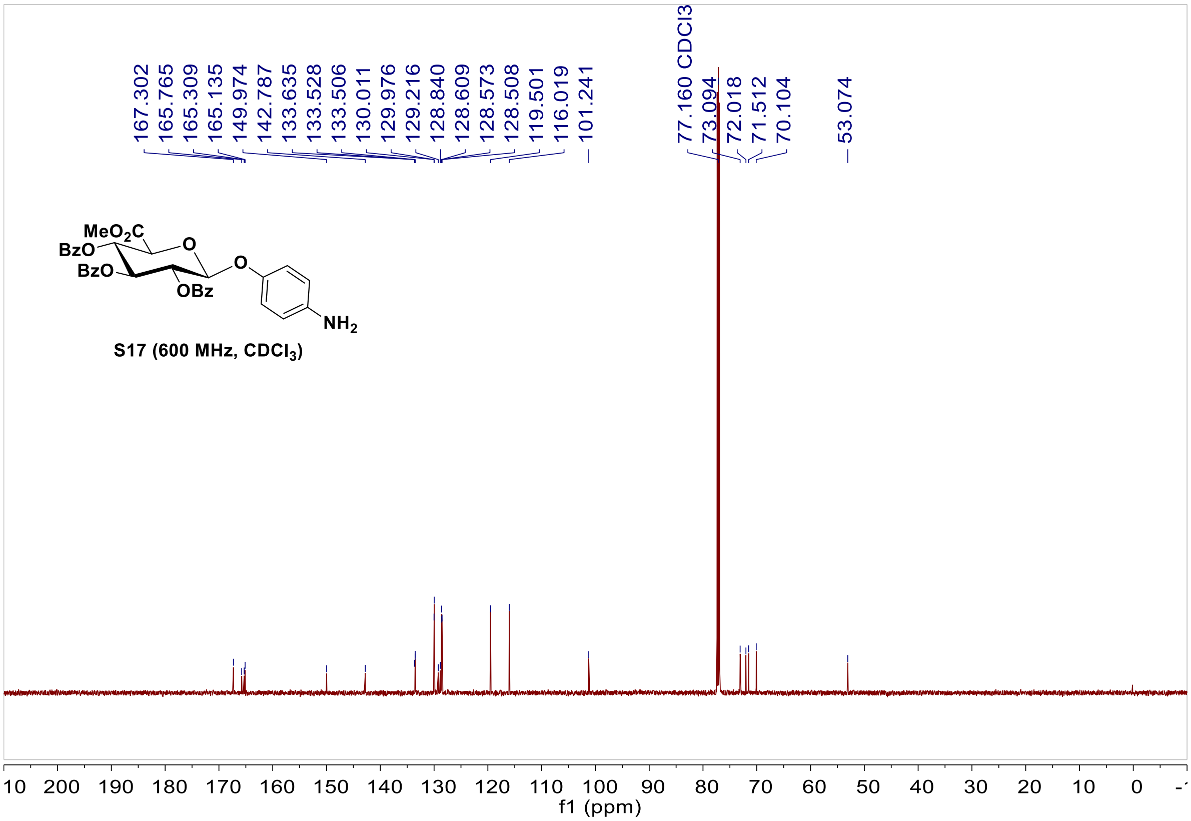
167.302
165.765
165.309
165.135
149.974
142.787
133.635
133.528
133.506
130.011
129.976
129.216
128.840
128.609
128.573
128.508
119.501
116.019
101.241
77.160 CDCl3
73.094
72.018
71.512
70.104
53.074
MeO2C
BzO
O
BzO
OBz
O
NH2
S17 (600 MHz, CDCl3)
200 190 180 170 160 150 140 130 120 110 100 90 80 70 60 50 40 30 20 10 0
f1 (ppm)

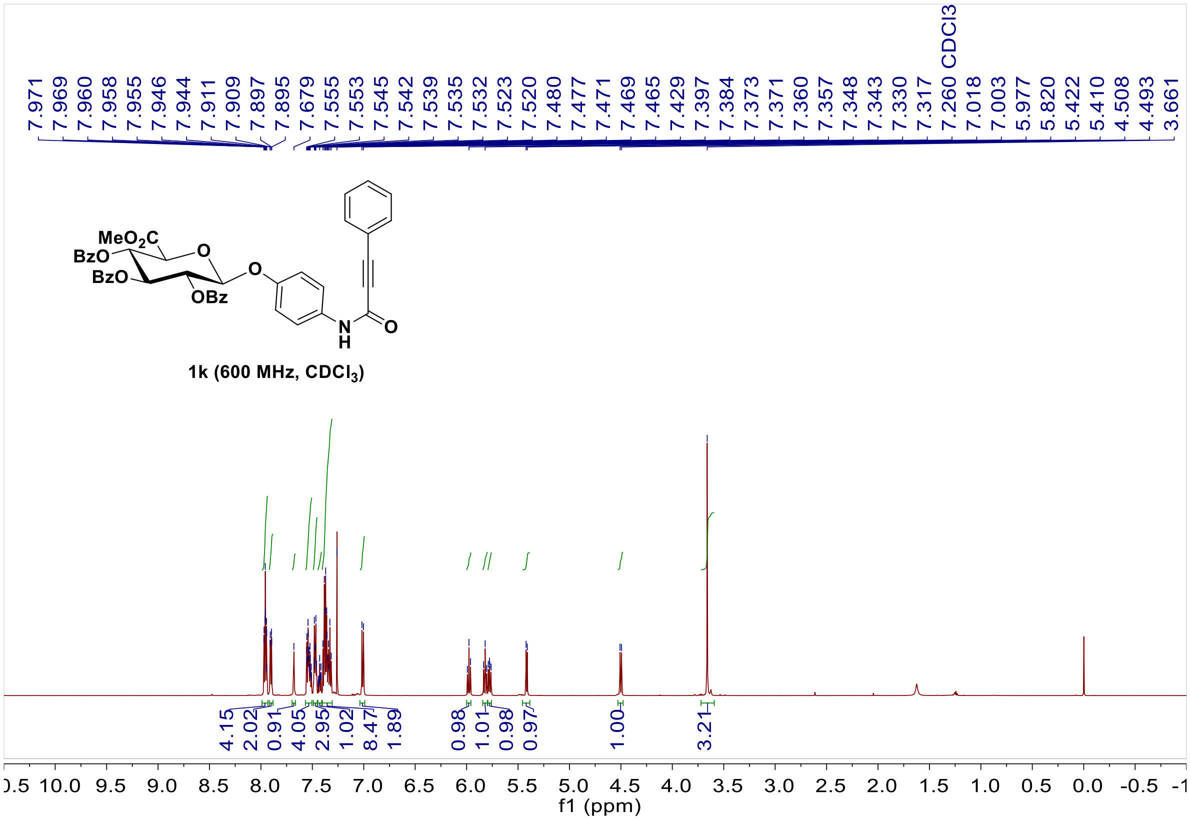
7.971 7.969 7.960 7.958 7.955 7.946 7.944 7.911 7.909 7.897 7.895 7.679 7.555 7.553 7.545 7.542 7.539 7.535 7.532 7.523 7.520 7.480 7.477 7.471 7.469 7.465 7.429 7.397 7.384 7.373 7.371 7.360 7.357 7.348 7.343 7.330 7.317 7.260 CDCl3 7.018 7.003 5.977 5.820 5.422 5.410 4.508 4.493 3.661
MeO2C
BzO
BzO
OBz
O
O
N
H
O
1k (600 MHz, CDCl3)
4.15 2.02 0.91 4.05 2.95 1.02 8.47 1.89 0.98 1.01 0.98 0.97 1.00 3.21
f1 (ppm)

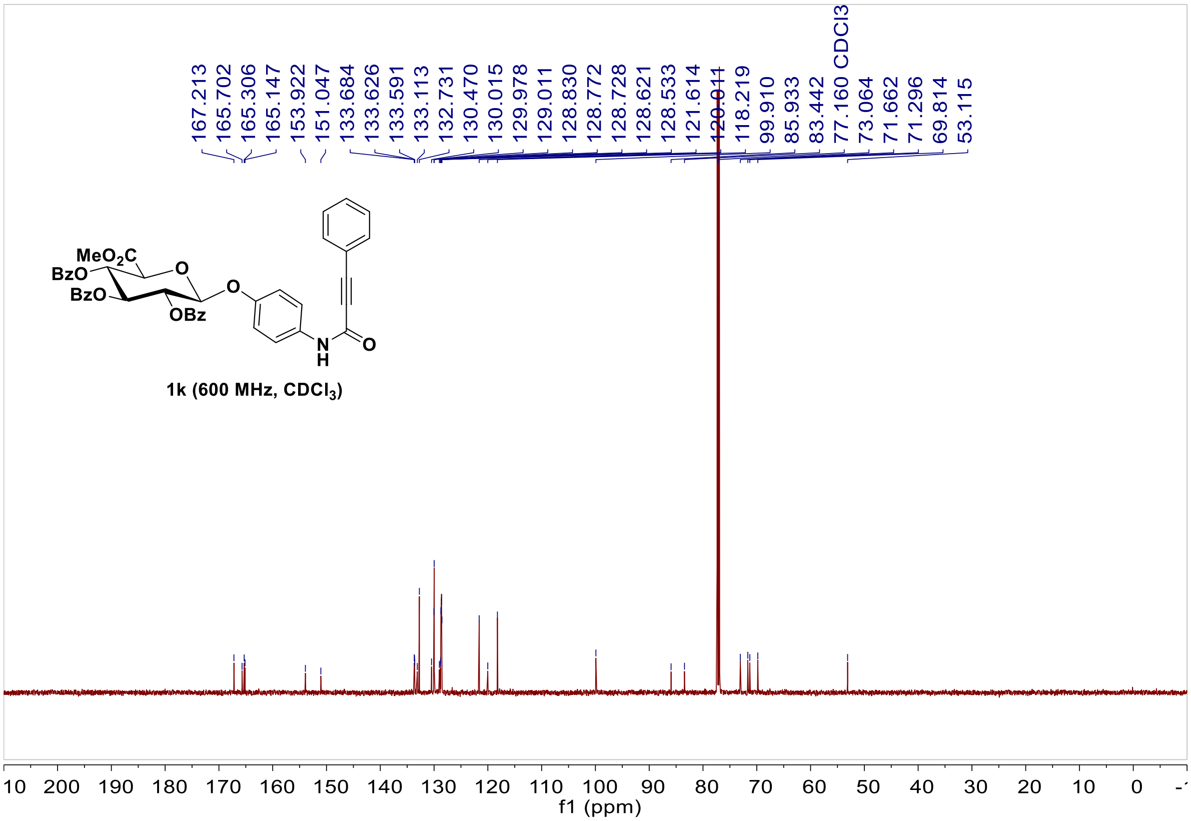
167.213 165.702 165.306 165.147 153.922 151.047 133.684 133.626 133.591 133.113 132.731 130.470 130.015 129.978 129.011 128.830 128.772 128.728 128.621 128.533 121.614 120.011 118.219 99.910 85.933 83.442 77.160 CDCl3 73.064 71.662 71.296 69.814 53.115
MeO2C
BzO
BzO
OBz
O
O
N
H
O
1k (600 MHz, CDCl3)
f1 (ppm)

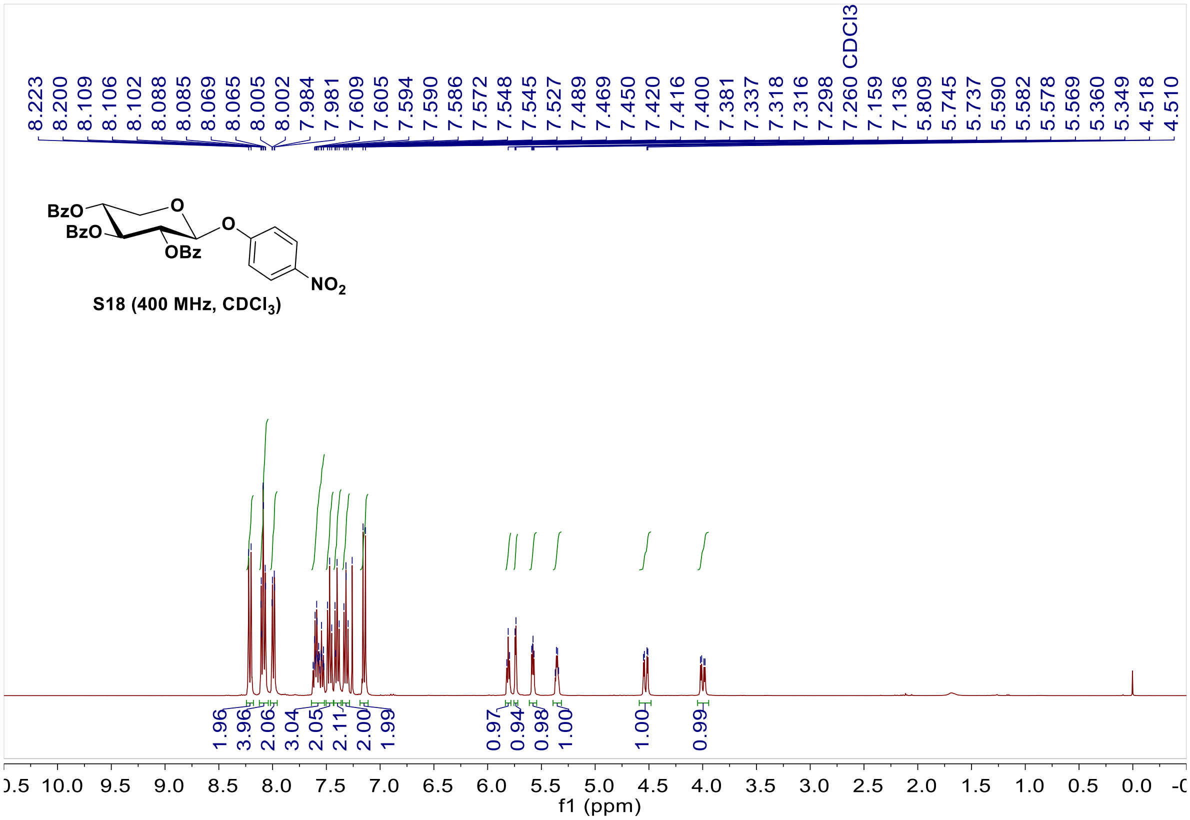

8.223
8.200
8.109
8.106
8.102
8.088
8.085
8.069
8.065
8.005
8.002
7.984
7.981
7.609
7.605
7.594
7.590
7.586
7.572
7.548
7.545
7.527
7.489
7.469
7.450
7.420
7.416
7.400
7.381
7.337
7.318
7.316
7.298
7.260 CDCl3
7.159
7.136
5.809
5.745
5.737
5.590
5.582
5.578
5.569
5.360
5.349
4.518
4.510
BzO
BzO
O
O
OBz
NO_2
S18 (400 MHz, $CDCl_3$)
1.96
3.96
2.06
3.04
2.05
2.11
2.00
1.99
0.97
0.94
0.98
1.00
1.00
0.99
f1 (ppm)

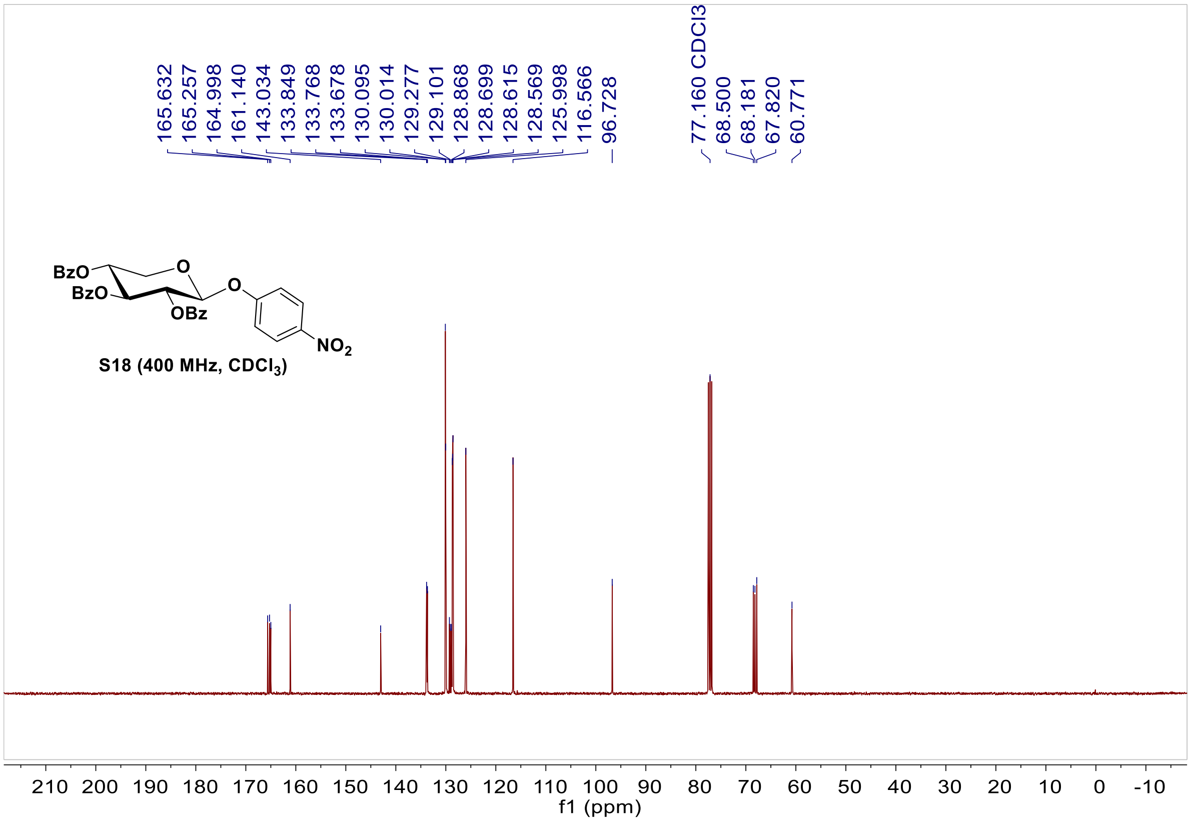

165.632
165.257
164.998
161.140
143.034
133.849
133.768
133.678
130.095
130.014
129.277
129.101
128.868
128.699
128.615
128.569
125.998
116.566
96.728
77.160 CDCl3
68.500
68.181
67.820
60.771
BzO
BzO
O
O
OBz
NO_2
S18 (400 MHz, $CDCl_3$)
f1 (ppm)

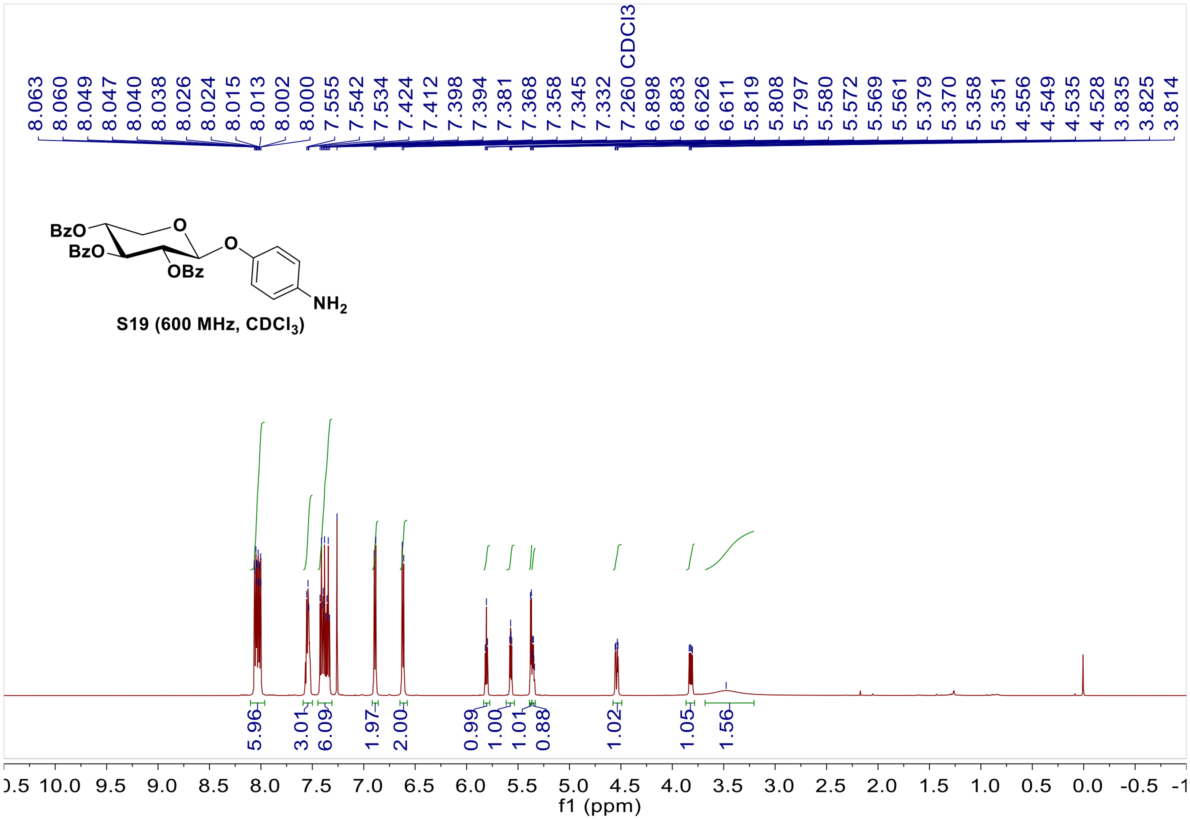
8.063 8.060 8.049 8.047 8.040 8.038 8.026 8.024 8.015 8.013 8.002 8.000 7.555 7.542 7.534 7.424 7.412 7.398 7.394 7.381 7.368 7.358 7.345 7.332 7.260 CDCl3 6.898 6.883 6.626 6.611 5.819 5.808 5.797 5.580 5.572 5.569 5.561 5.379 5.370 5.358 5.351 4.556 4.549 4.535 4.528 3.835 3.825 3.814
BzO
BzO
O
OBz
O
NH2
S19 (600 MHz, CDCl3)
5.96 3.01 6.09 1.97 2.00 0.99 1.00 1.01 0.88 1.02 1.05 1.56
f1 (ppm)

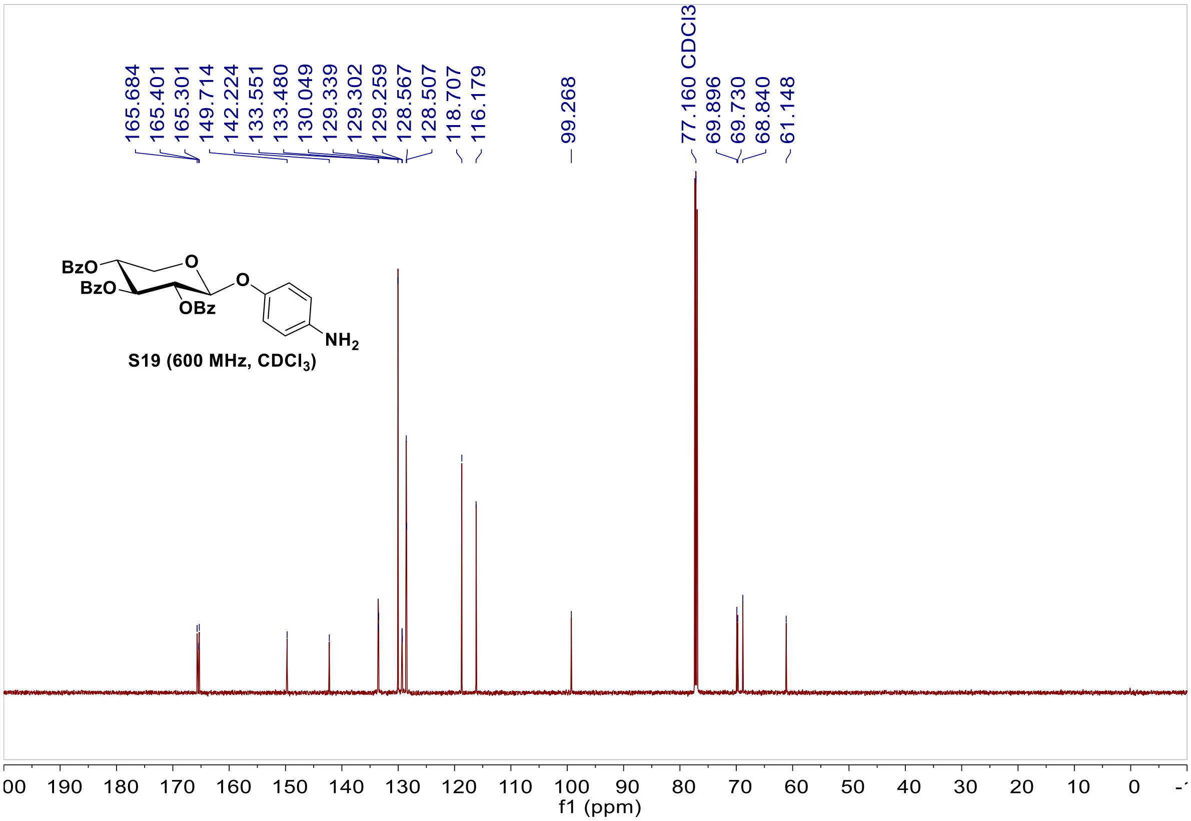
165.684 165.401 165.301 149.714 142.224 133.551 133.480 130.049 129.339 129.302 129.259 128.567 128.507 118.707 116.179 99.268 77.160 CDCl3 69.896 69.730 68.840 61.148
BzO
BzO
O
OBz
O
NH2
S19 (600 MHz, CDCl3)
f1 (ppm)

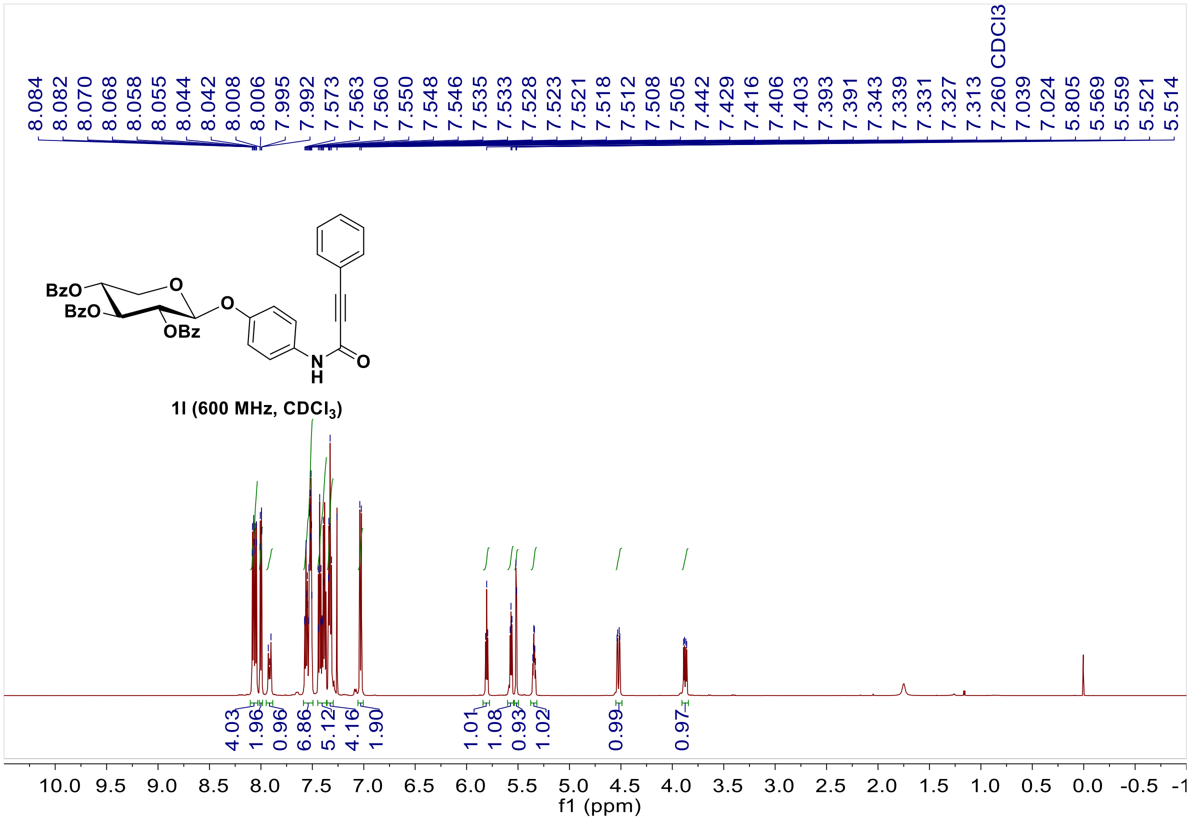
8.084 8.082 8.070 8.068 8.058 8.055 8.044 8.042 8.008 8.006 7.995 7.992 7.573 7.563 7.560 7.550 7.548 7.546 7.535 7.533 7.528 7.523 7.521 7.518 7.512 7.508 7.505 7.442 7.429 7.416 7.406 7.403 7.393 7.391 7.343 7.339 7.331 7.327 7.313 7.260 CDCl3 7.039 7.024 5.805 5.569 5.559 5.521 5.514
BzO
BzO
O
OBz
O
N
H
O
1l (600 MHz, CDCl3)
4.03 1.96 0.96 6.86 5.12 4.16 1.90 1.01 1.08 0.93 1.02 0.99 0.97
10.0 9.5 9.0 8.5 8.0 7.5 7.0 6.5 6.0 5.5 5.0 4.5 4.0 3.5 3.0 2.5 2.0 1.5 1.0 0.5 0.0 -0.5 -1
f1 (ppm)

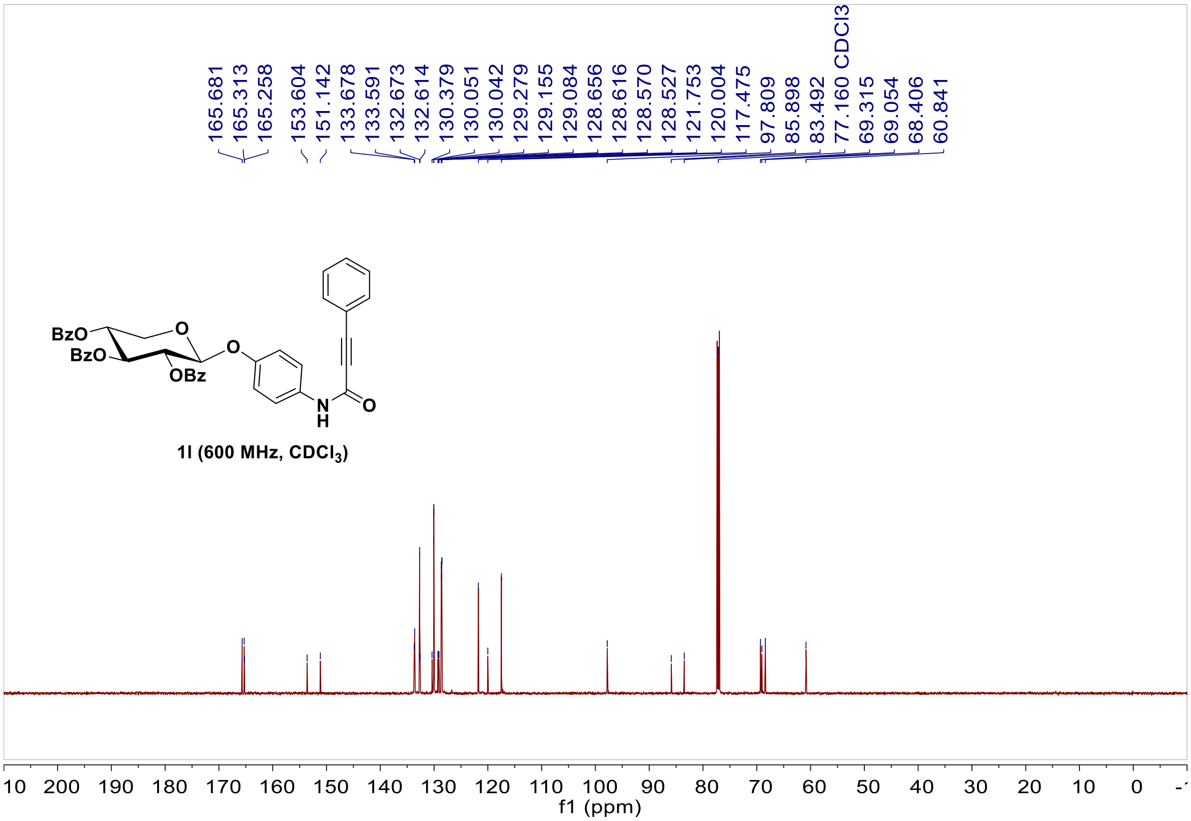
165.681 165.313 165.258 153.604 151.142 133.678 133.591 132.673 132.614 130.379 130.051 130.042 129.279 129.155 129.084 128.656 128.616 128.570 128.527 121.753 120.004 117.475 97.809 85.898 83.492 77.160 CDCl3 69.315 69.054 68.406 60.841
BzO
BzO
O
OBz
O
N
H
O
1l (600 MHz, CDCl3)
10 200 190 180 170 160 150 140 130 120 110 100 90 80 70 60 50 40 30 20 10 0
f1 (ppm)

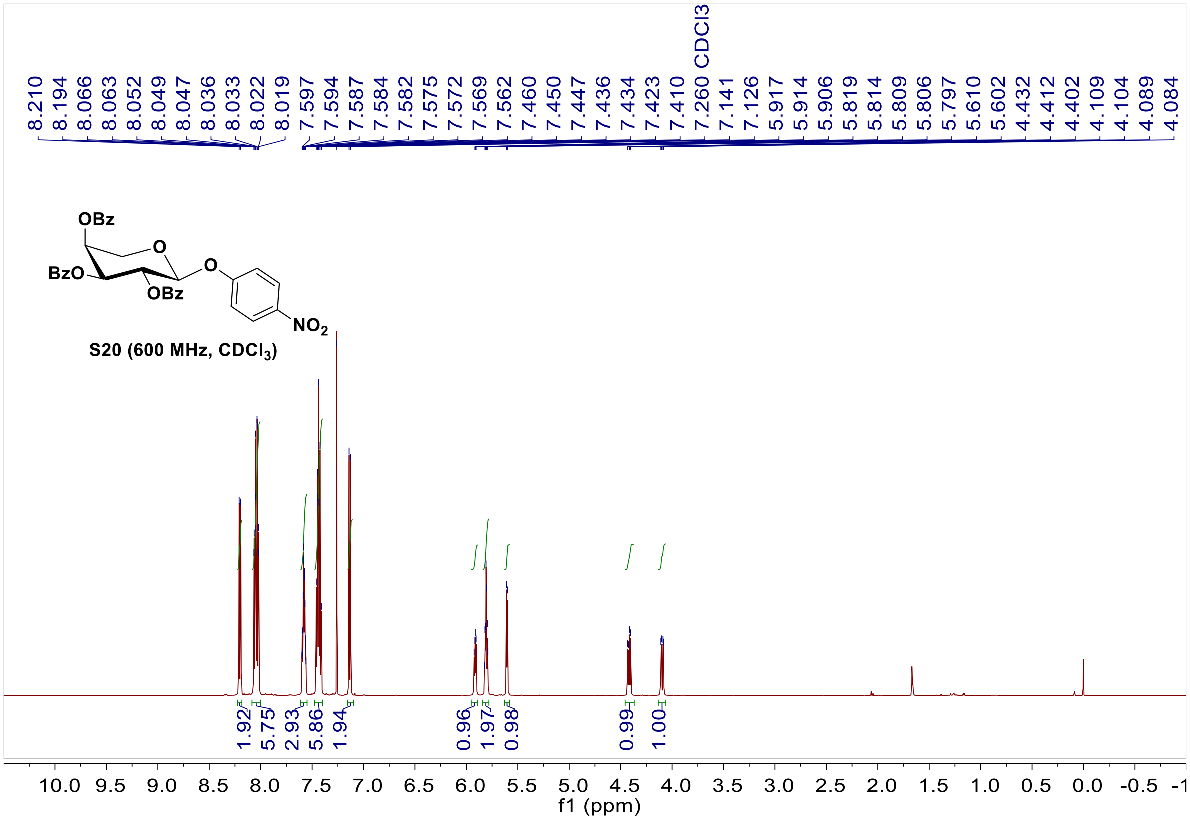
8.210
8.194
8.066
8.063
8.052
8.049
8.047
8.036
8.033
8.022
8.019
7.597
7.594
7.587
7.584
7.582
7.575
7.572
7.569
7.562
7.460
7.450
7.447
7.436
7.434
7.423
7.410
7.260 CDCl3
7.141
7.126
5.917
5.914
5.906
5.819
5.814
5.809
5.806
5.797
5.610
5.602
4.432
4.412
4.402
4.109
4.104
4.089
4.084
OBz
O
BzO
OBz
O
NO2
S20 (600 MHz, CDCl3)
1.92
5.75
2.93
5.86
1.94
0.96
1.97
0.98
0.99
1.00
10.0 9.5 9.0 8.5 8.0 7.5 7.0 6.5 6.0 5.5 5.0 4.5 4.0 3.5 3.0 2.5 2.0 1.5 1.0 0.5 0.0 -0.5 -1
f1 (ppm)

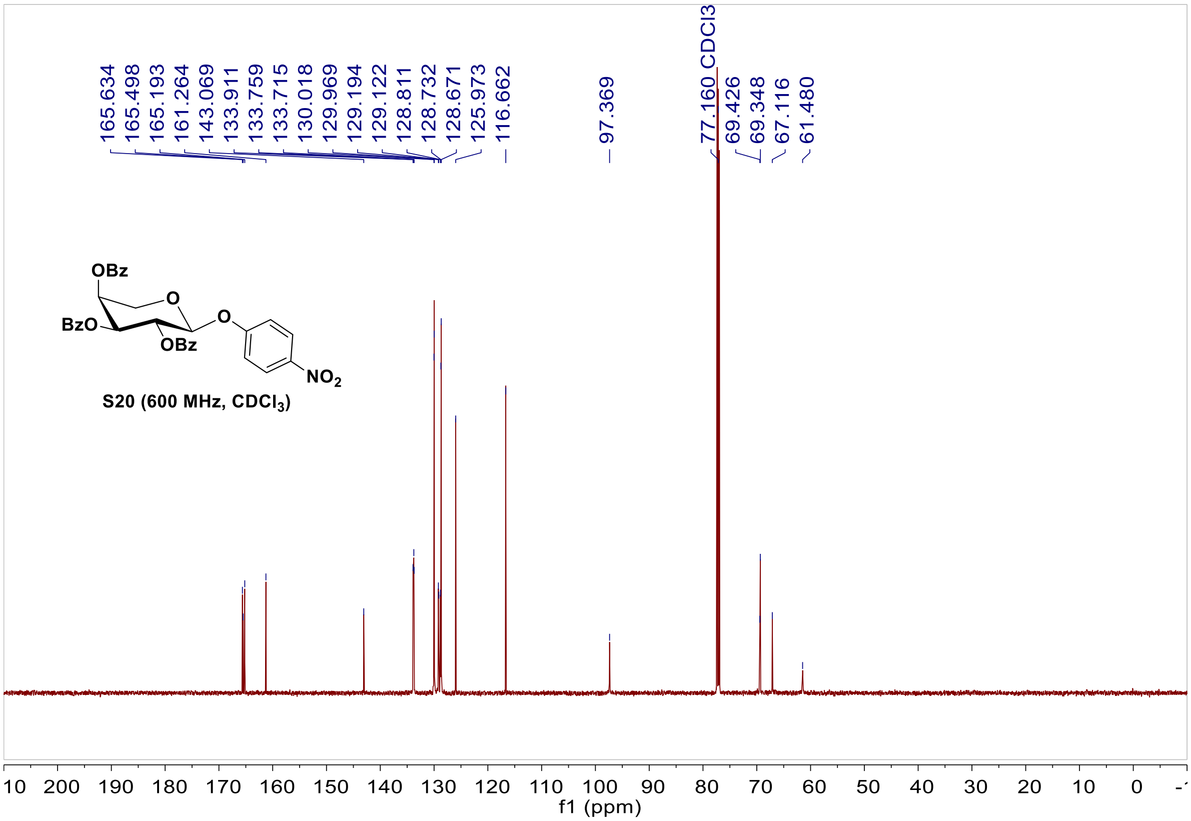
165.634
165.498
165.193
161.264
143.069
133.911
133.759
133.715
130.018
129.969
129.194
129.122
128.811
128.732
128.671
125.973
116.662
97.369
77.160 CDCl3
69.426
69.348
67.116
61.480
OBz
O
BzO
OBz
O
NO2
S20 (600 MHz, CDCl3)
10 200 190 180 170 160 150 140 130 120 110 100 90 80 70 60 50 40 30 20 10 0
f1 (ppm)

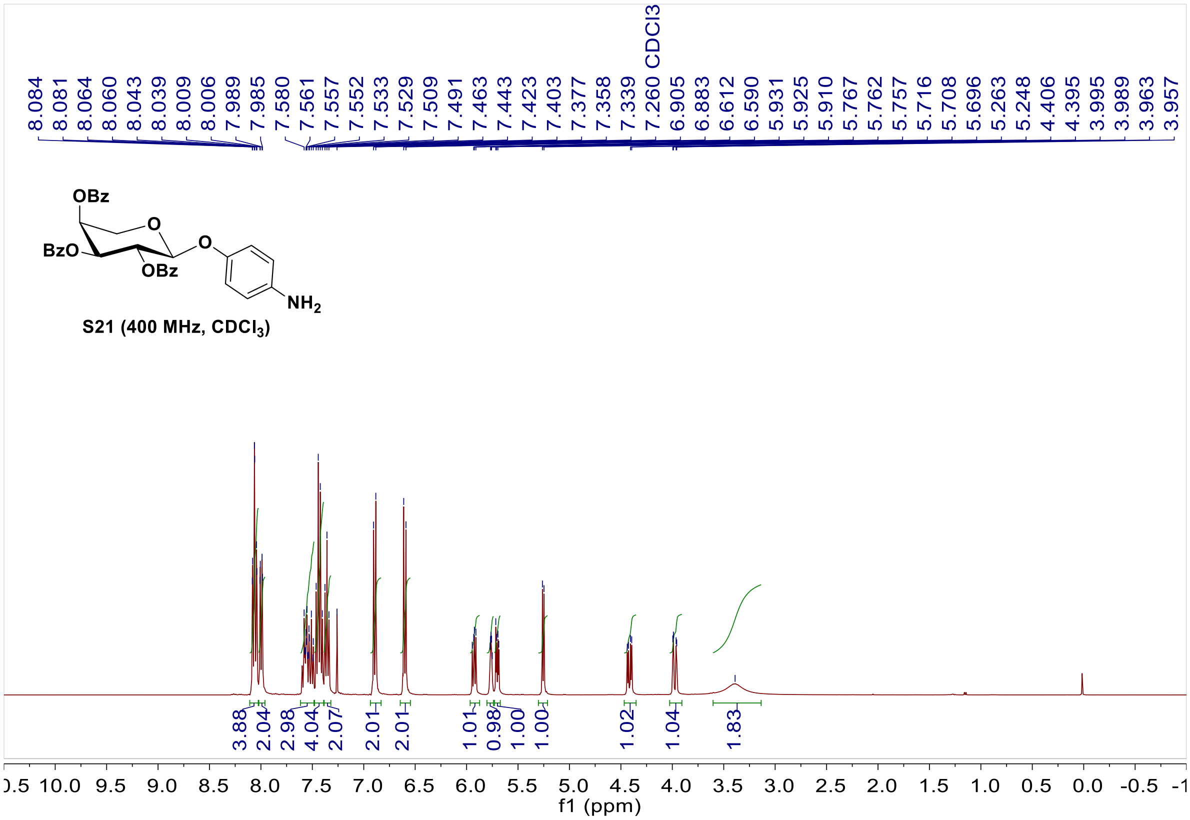
8.084 8.081 8.064 8.060 8.043 8.039 8.009 8.006 7.989 7.985 7.580 7.561 7.557 7.552 7.533 7.529 7.509 7.491 7.463 7.443 7.423 7.403 7.377 7.358 7.339 7.260 CDCl3 6.905 6.883 6.612 6.590 5.931 5.925 5.910 5.767 5.762 5.757 5.716 5.708 5.696 5.263 5.248 4.406 4.395 3.995 3.989 3.963 3.957
OBz
BzO
O
OBz
O
NH2
S21 (400 MHz, CDCl3)
3.88 2.04 2.98 4.04 2.07 2.01 2.01 1.01 0.98 1.00 1.00 1.02 1.04 1.83
f1 (ppm)

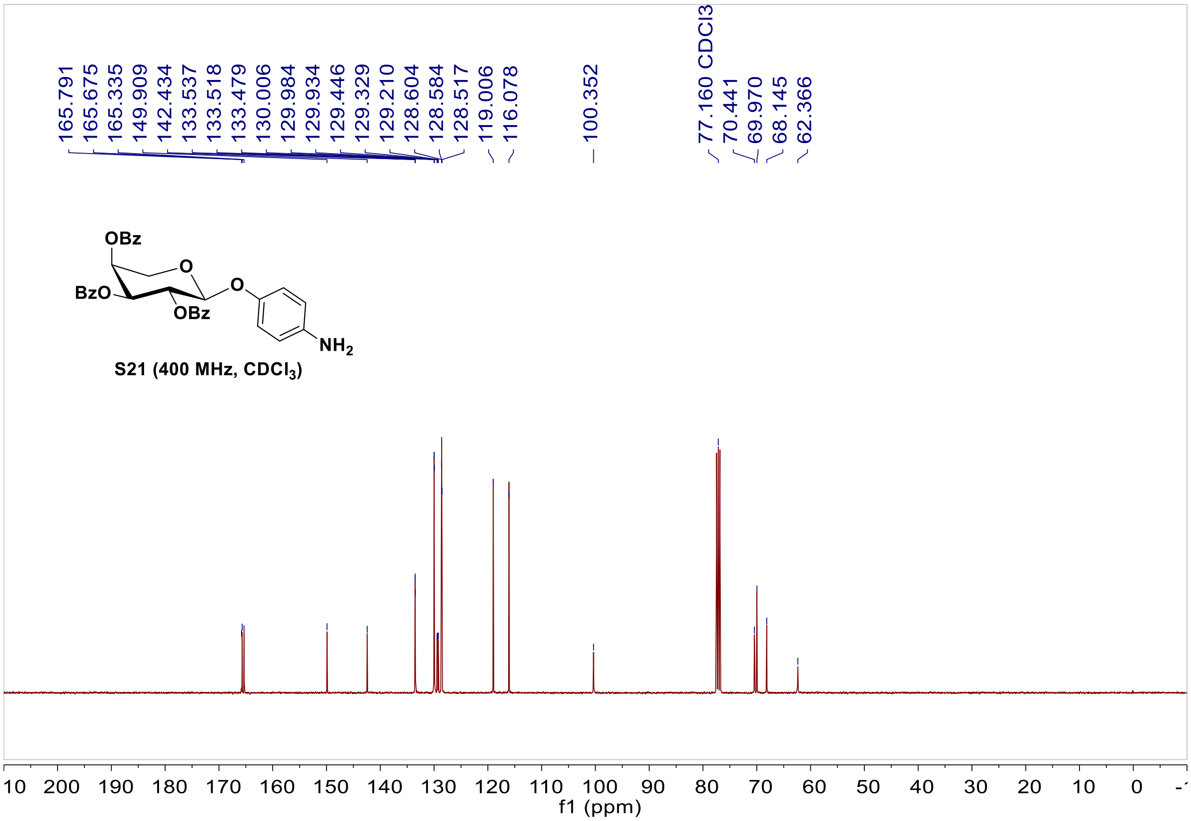
165.791 165.675 165.335 149.909 142.434 133.537 133.518 133.479 130.006 129.984 129.934 129.446 129.329 129.210 128.604 128.584 128.517 119.006 116.078
100.352
77.160 CDCl3 70.441 69.970 68.145 62.366
OBz
BzO
O
OBz
O
NH2
S21 (400 MHz, CDCl3)
f1 (ppm)

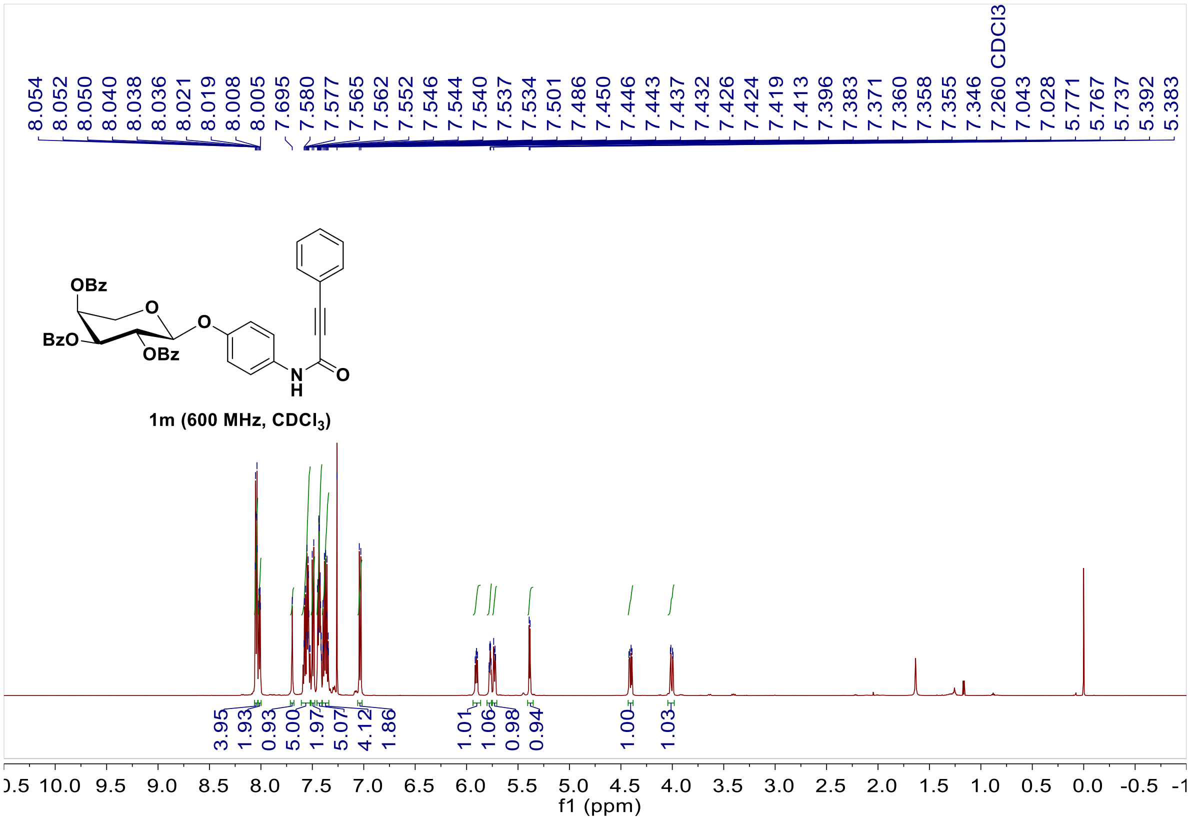
8.054 8.052 8.050 8.040 8.038 8.036 8.021 8.019 8.008 8.005 7.695 7.580 7.577 7.565 7.562 7.552 7.546 7.544 7.540 7.537 7.534 7.501 7.486 7.450 7.446 7.443 7.437 7.432 7.426 7.424 7.419 7.413 7.396 7.383 7.371 7.360 7.358 7.355 7.346 7.260 CDCl3 7.043 7.028 5.771 5.767 5.737 5.392 5.383
OBz
BzO
OBz
O
O
N
H
O
1m (600 MHz, CDCl3)
3.95 1.93 0.93 5.00 1.97 5.07 4.12 1.86 1.01 1.06 0.98 0.94 1.00 1.03
f1 (ppm)

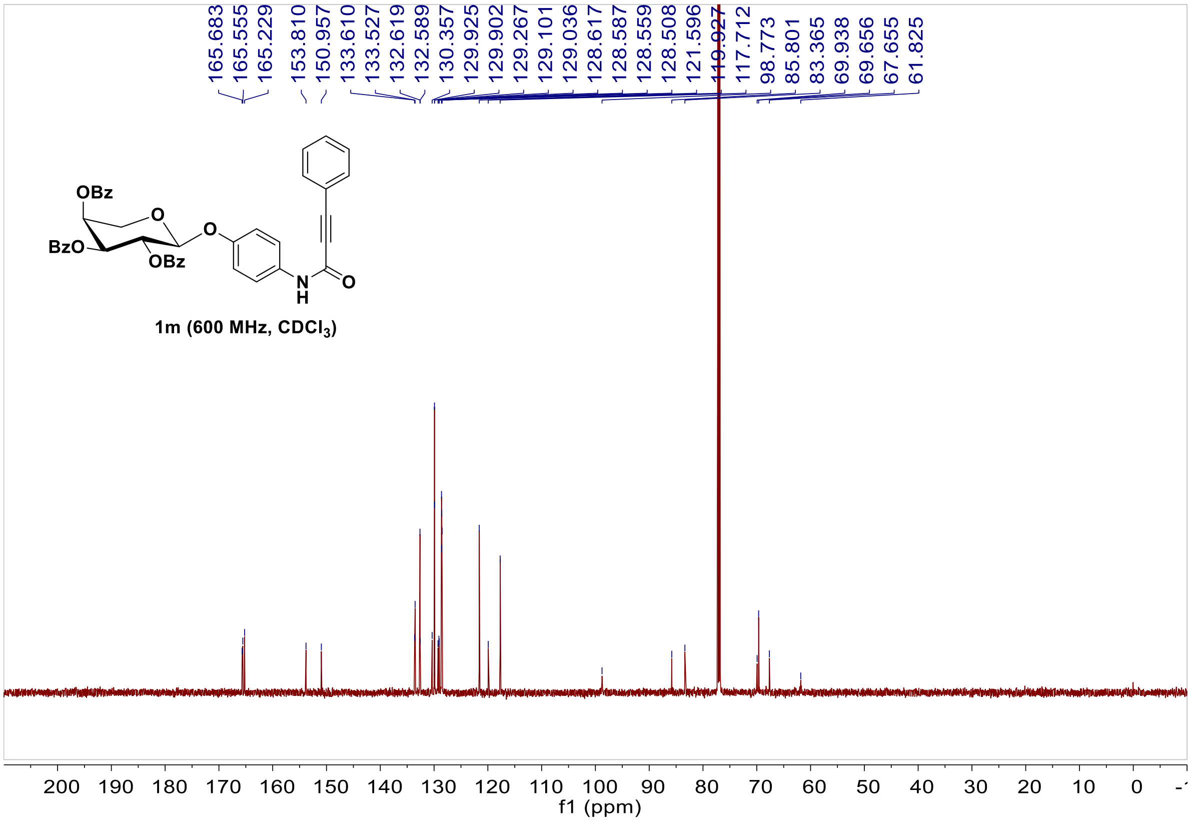
165.683 165.555 165.229 153.810 150.957 133.610 133.527 132.619 132.589 130.357 129.925 129.902 129.267 129.101 129.036 128.617 128.587 128.559 128.508 121.596 119.927 117.712 98.773 85.801 83.365 69.938 69.656 67.655 61.825
OBz
BzO
OBz
O
O
N
H
O
1m (600 MHz, CDCl3)
f1 (ppm)

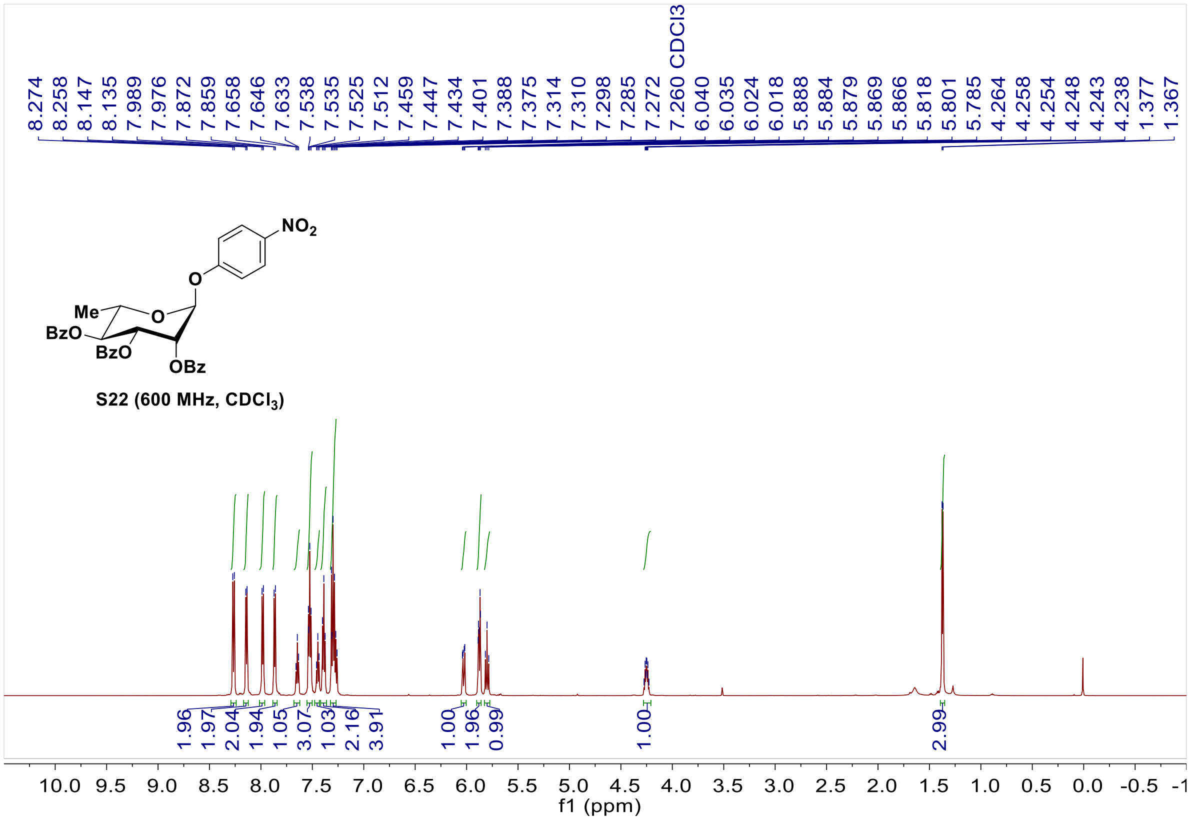
8.274
8.258
8.147
8.135
7.989
7.976
7.872
7.859
7.658
7.646
7.633
7.538
7.535
7.525
7.512
7.459
7.447
7.434
7.401
7.388
7.375
7.314
7.310
7.298
7.285
7.272
7.260 CDCl3
6.040
6.035
6.024
6.018
5.888
5.884
5.879
5.869
5.866
5.818
5.801
5.785
4.264
4.258
4.254
4.248
4.243
4.238
1.377
1.367
NO2
O
Me
O
BzO
BzO
OBz
S22 (600 MHz, CDCl3)
1.96
1.97
2.04
1.94
1.05
3.07
1.03
2.16
3.91
1.00
1.96
0.99
1.00
2.99
10.0 9.5 9.0 8.5 8.0 7.5 7.0 6.5 6.0 5.5 5.0 4.5 4.0 3.5 3.0 2.5 2.0 1.5 1.0 0.5 0.0 -0.5 -1
f1 (ppm)

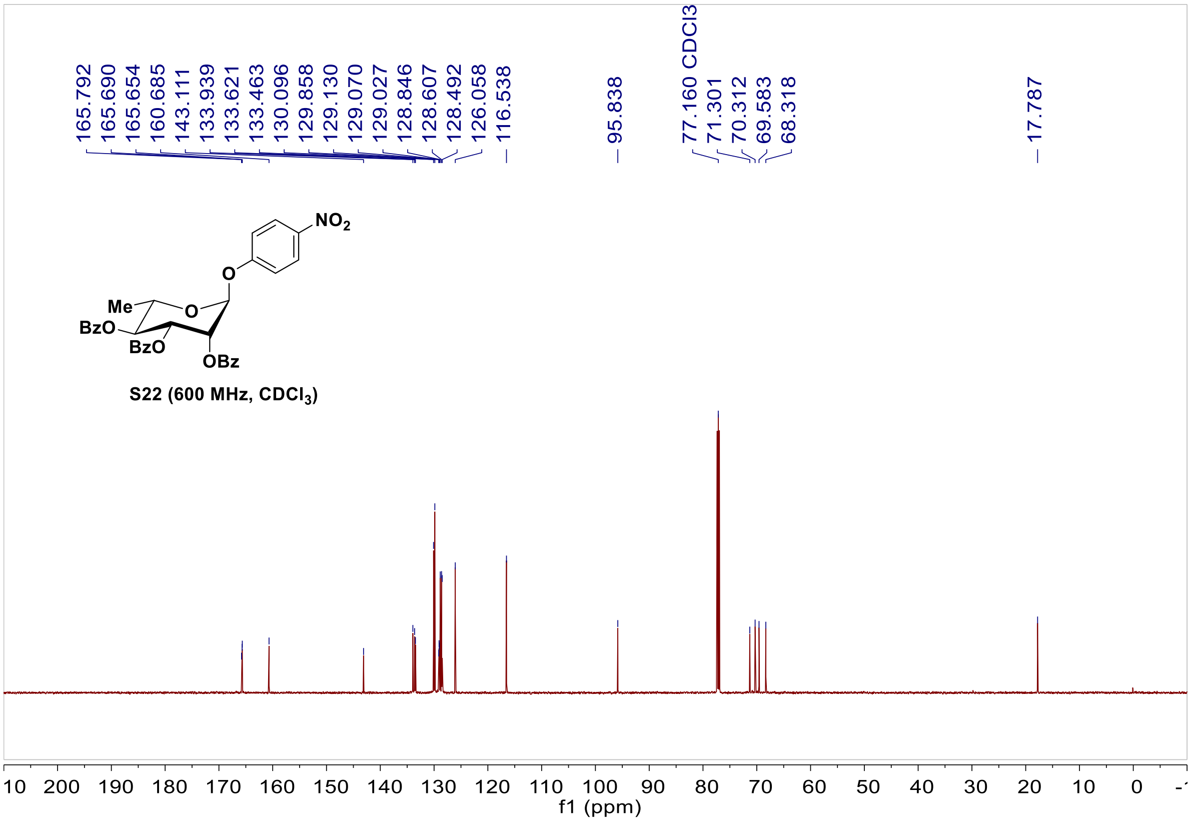
165.792
165.690
165.654
160.685
143.111
133.939
133.621
133.463
130.096
129.858
129.130
129.070
129.027
128.846
128.607
128.492
126.058
116.538
95.838
77.160 CDCl3
71.301
70.312
69.583
68.318
17.787
NO2
O
Me
O
BzO
BzO
OBz
S22 (600 MHz, CDCl3)
10 200 190 180 170 160 150 140 130 120 110 100 90 80 70 60 50 40 30 20 10 0
f1 (ppm)

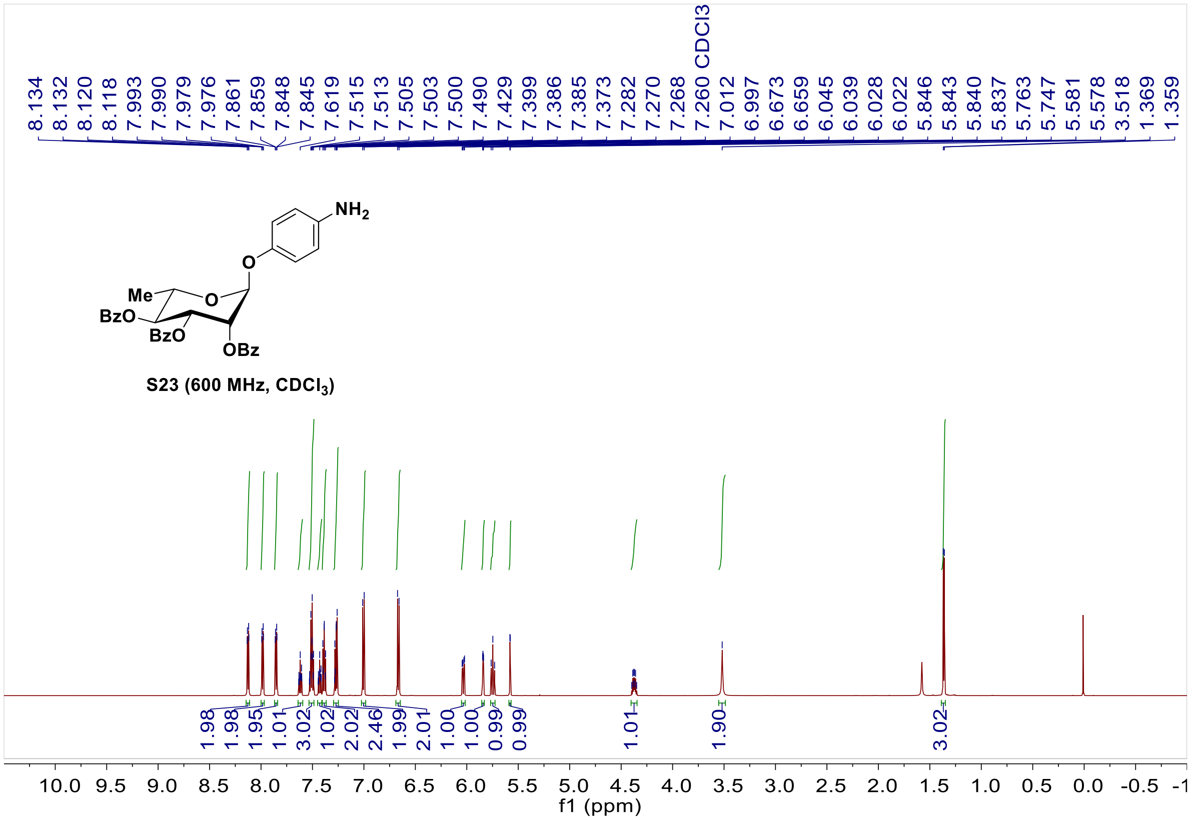
8.134 8.132 8.120 8.118 7.993 7.990 7.979 7.976 7.861 7.859 7.848 7.845 7.619 7.515 7.513 7.505 7.503 7.500 7.490 7.429 7.399 7.386 7.385 7.373 7.282 7.270 7.268 7.260 CDCl3 7.012 6.997 6.673 6.659 6.045 6.039 6.028 6.022 5.846 5.843 5.840 5.837 5.763 5.747 5.581 5.578 3.518 1.369 1.359
NH2
O
Me
O
BzO
BzO
OBz
S23 (600 MHz, CDCl3)
1.98 1.98 1.95 1.01 3.02 1.02 2.02 2.46 1.99 2.01 1.00 1.00 0.99 0.99 1.01 1.90 3.02
10.0 9.5 9.0 8.5 8.0 7.5 7.0 6.5 6.0 5.5 5.0 4.5 4.0 3.5 3.0 2.5 2.0 1.5 1.0 0.5 0.0 -0.5 -1
f1 (ppm)

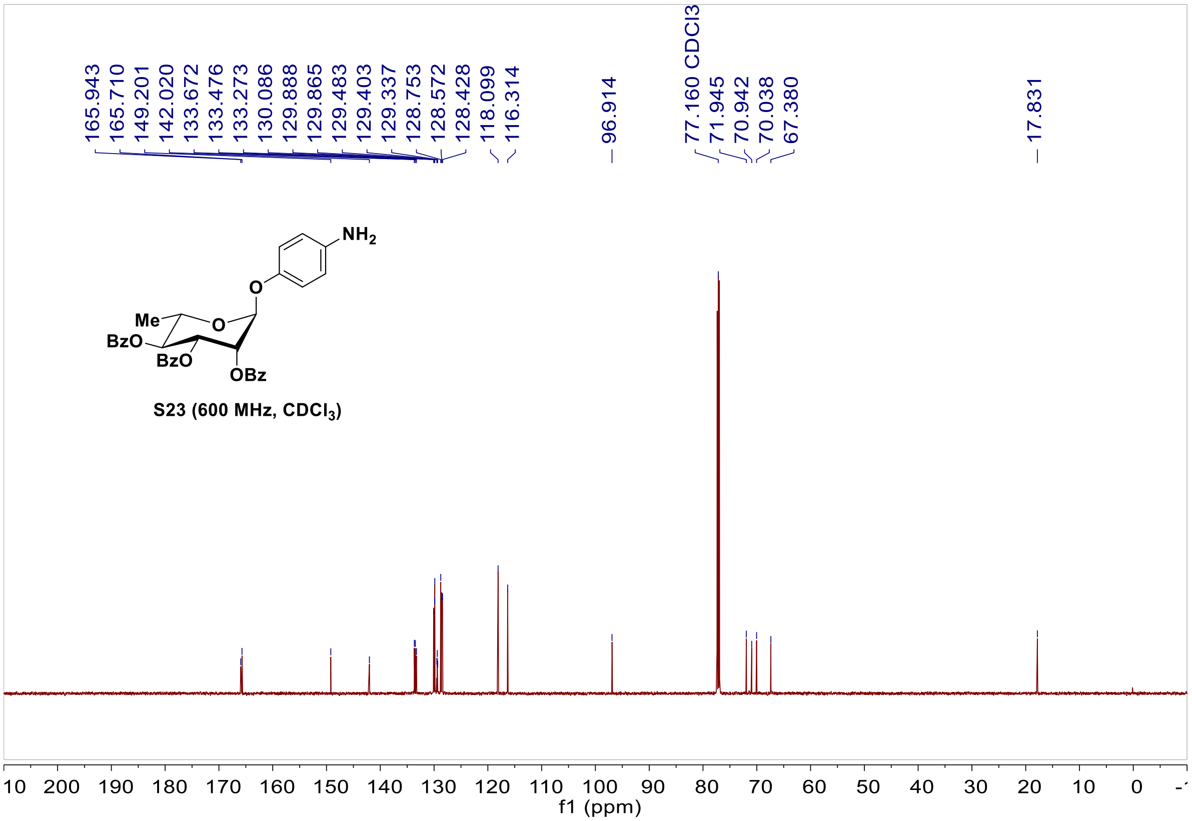
165.943 165.710 149.201 142.020 133.672 133.476 133.273 130.086 129.888 129.865 129.483 129.403 129.337 128.753 128.572 128.428 118.099 116.314 96.914 77.160 CDCl3 71.945 70.942 70.038 67.380 17.831
NH2
O
Me
O
BzO
BzO
OBz
S23 (600 MHz, CDCl3)
10 200 190 180 170 160 150 140 130 120 110 100 90 80 70 60 50 40 30 20 10 0
f1 (ppm)

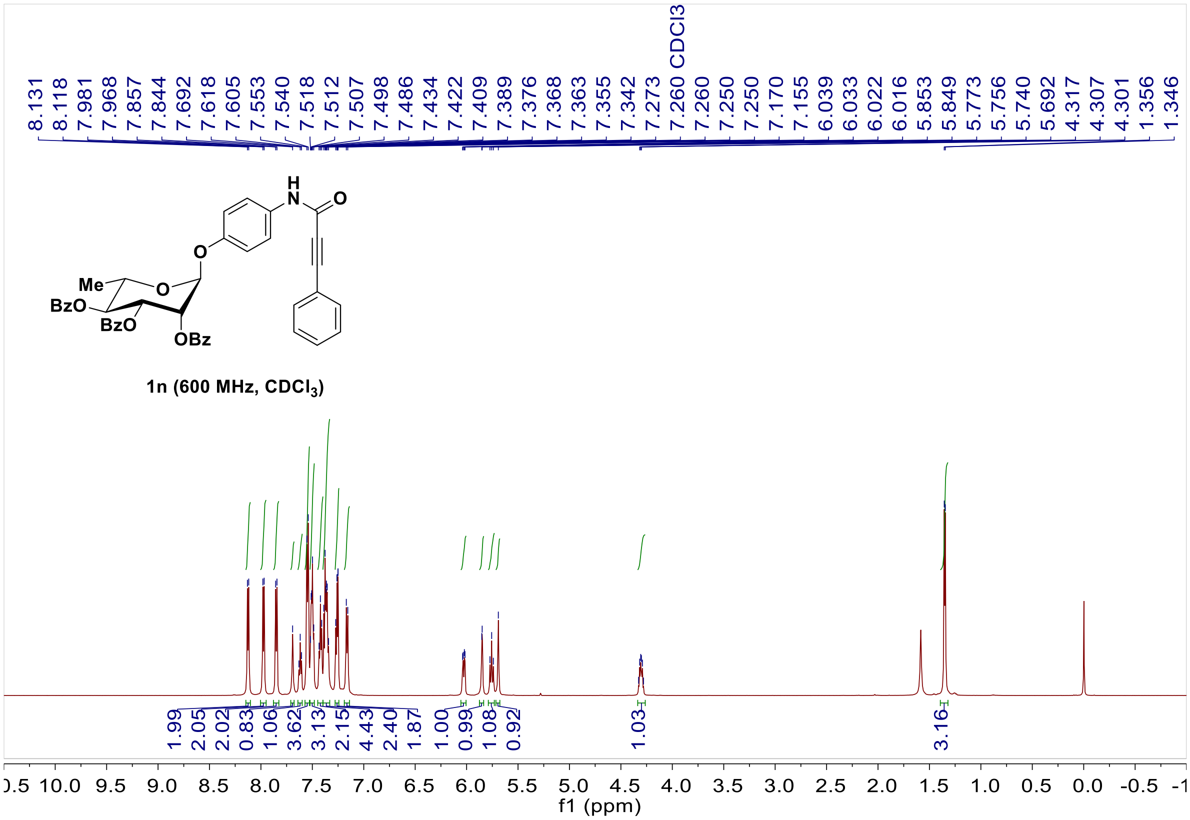
8.131 8.118 7.981 7.968 7.857 7.844 7.692 7.618 7.605 7.553 7.540 7.518 7.512 7.507 7.498 7.486 7.434 7.422 7.409 7.389 7.376 7.368 7.363 7.355 7.342 7.273 7.260 CDCl3 7.260 7.250 7.250 7.170 7.155 6.039 6.033 6.022 6.016 5.853 5.849 5.773 5.756 5.740 5.692 4.317 4.307 4.301 1.356 1.346
Me
O
BzO
BzO
OBz
H
N
O
1n (600 MHz, CDCl3)
1.99 2.05 2.02 0.83 1.06 3.62 3.13 2.15 4.43 2.40 1.87 1.00 0.99 1.08 0.92 1.03 3.16
0.5 10.0 9.5 9.0 8.5 8.0 7.5 7.0 6.5 6.0 5.5 5.0 4.5 4.0 3.5 3.0 2.5 2.0 1.5 1.0 0.5 0.0 -0.5 -1
f1 (ppm)
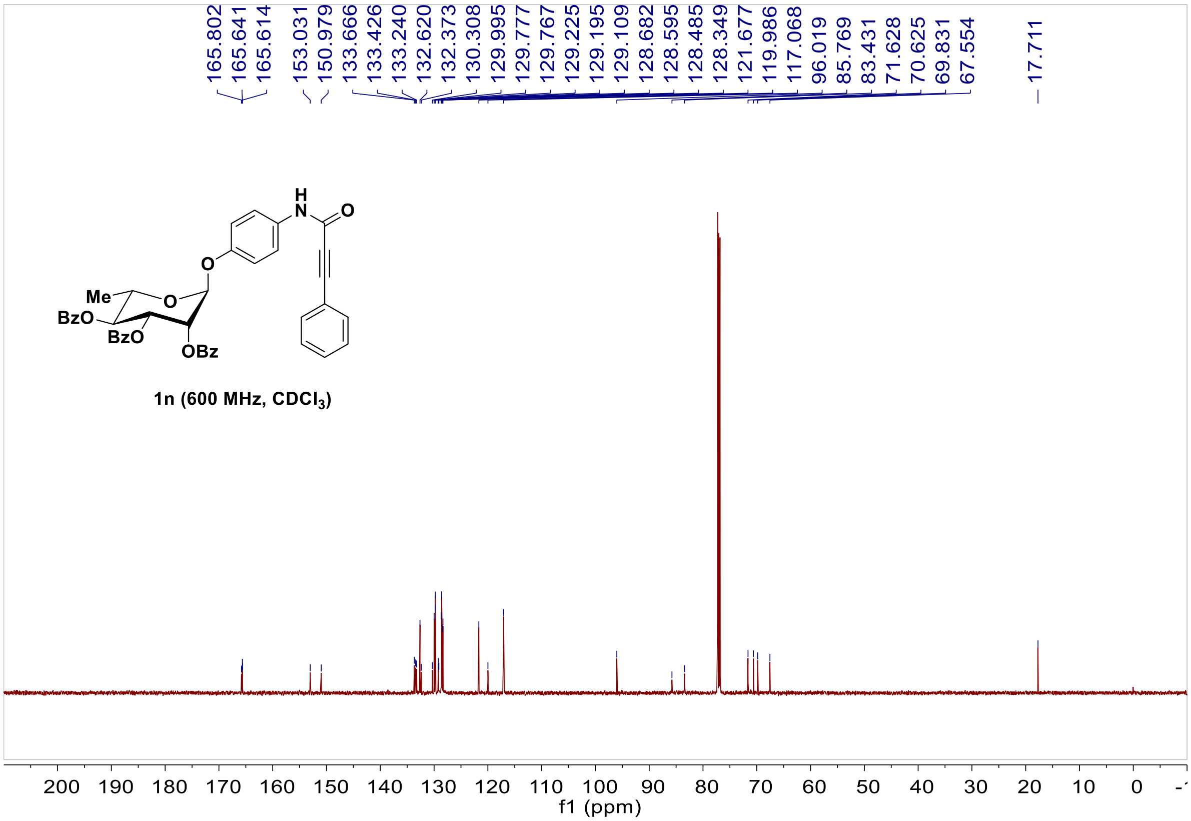
165.802 165.641 165.614 153.031 150.979 133.666 133.426 133.240 132.620 132.373 130.308 129.995 129.777 129.767 129.225 129.195 129.109 128.682 128.595 128.485 128.349 121.677 119.986 117.068 96.019 85.769 83.431 71.628 70.625 69.831 67.554 17.711
Me
O
BzO
BzO
OBz
H
N
O
1n (600 MHz, CDCl3)
200 190 180 170 160 150 140 130 120 110 100 90 80 70 60 50 40 30 20 10 0
f1 (ppm)

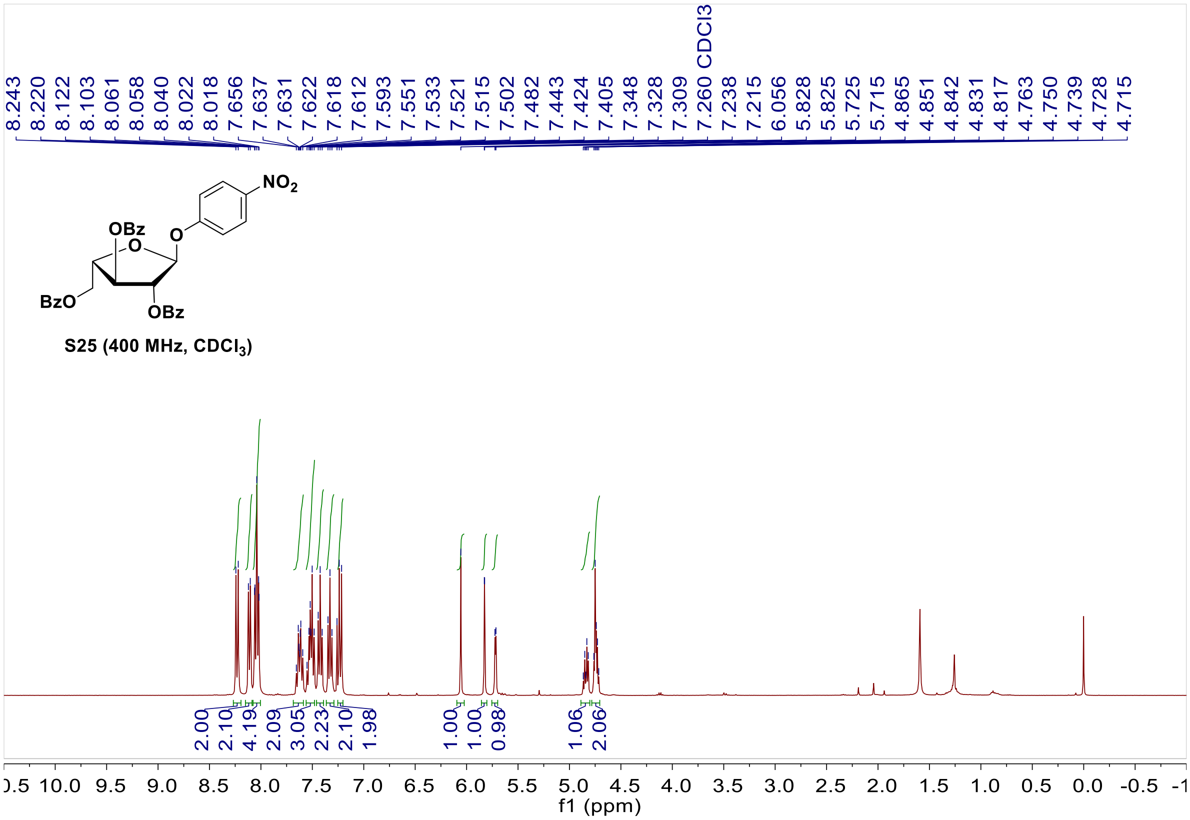
8.243 8.220 8.122 8.103 8.061 8.058 8.040 8.022 8.018 7.656 7.637 7.631 7.622 7.618 7.612 7.593 7.551 7.533 7.521 7.515 7.502 7.482 7.443 7.424 7.405 7.348 7.328 7.309 7.260 CDCl3 7.238 7.215 6.056 5.828 5.825 5.725 5.715 4.865 4.851 4.842 4.831 4.817 4.763 4.750 4.739 4.728 4.715
NO_2
OBz
O
O
BzO
OBz
S25 (400 MHz, $CDCl_3$)
2.00 2.10 4.19 2.09 3.05 2.23 2.10 1.98 1.00 1.00 0.98 1.06 2.06
f1 (ppm)

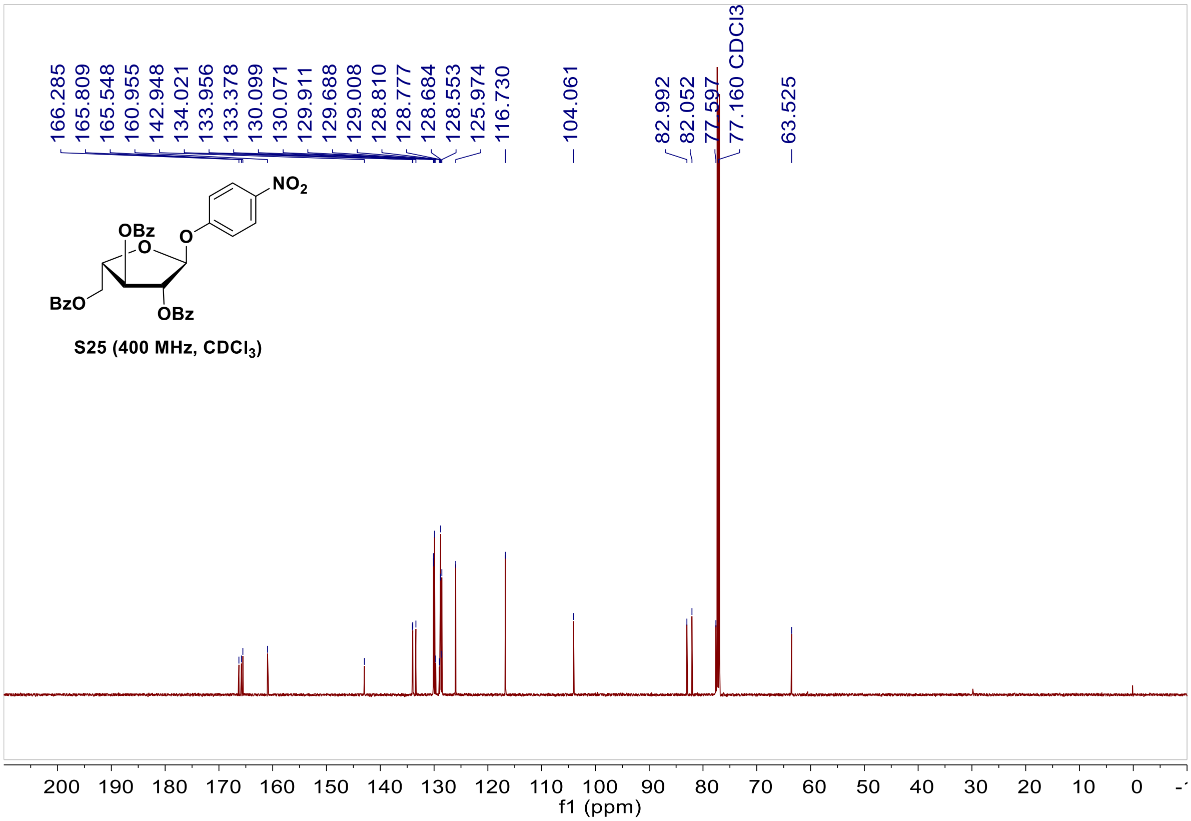
166.285 165.809 165.548 160.955 142.948 134.021 133.956 133.378 130.099 130.071 129.911 129.688 129.008 128.810 128.777 128.684 128.553 125.974 116.730 104.061 82.992 82.052 77.597 77.160 CDCl3 63.525
NO_2
OBz
O
O
BzO
OBz
S25 (400 MHz, $CDCl_3$)
f1 (ppm)

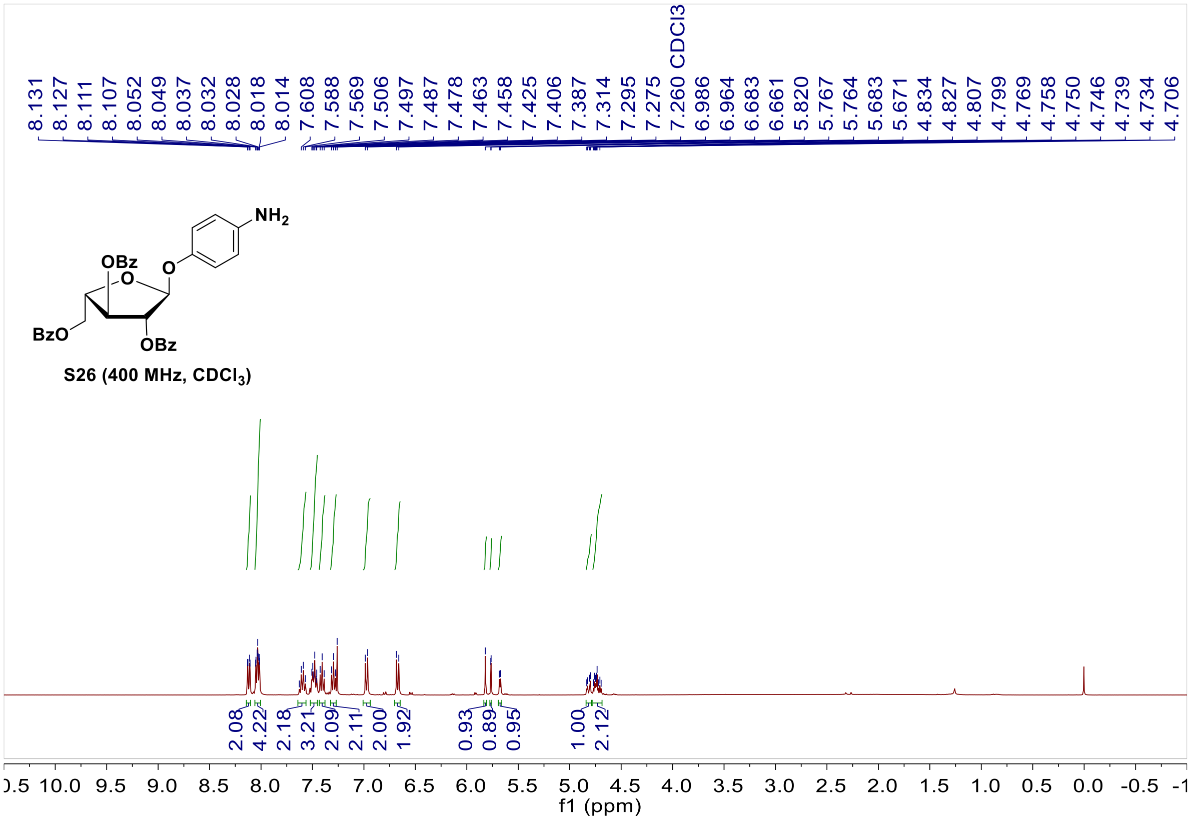
8.131
8.127
8.111
8.107
8.052
8.049
8.037
8.032
8.028
8.018
8.014
7.608
7.588
7.569
7.506
7.497
7.487
7.478
7.463
7.458
7.425
7.406
7.387
7.314
7.295
7.275
7.260 CDCl3
6.986
6.964
6.683
6.661
5.820
5.767
5.764
5.683
5.671
4.834
4.827
4.807
4.799
4.769
4.758
4.750
4.746
4.739
4.734
4.706
NH2
OBz
O
O
BzO
OBz
S26 (400 MHz, CDCl3)
2.08
4.22
2.18
3.21
2.09
2.11
2.00
1.92
0.93
0.89
0.95
1.00
2.12
f1 (ppm)

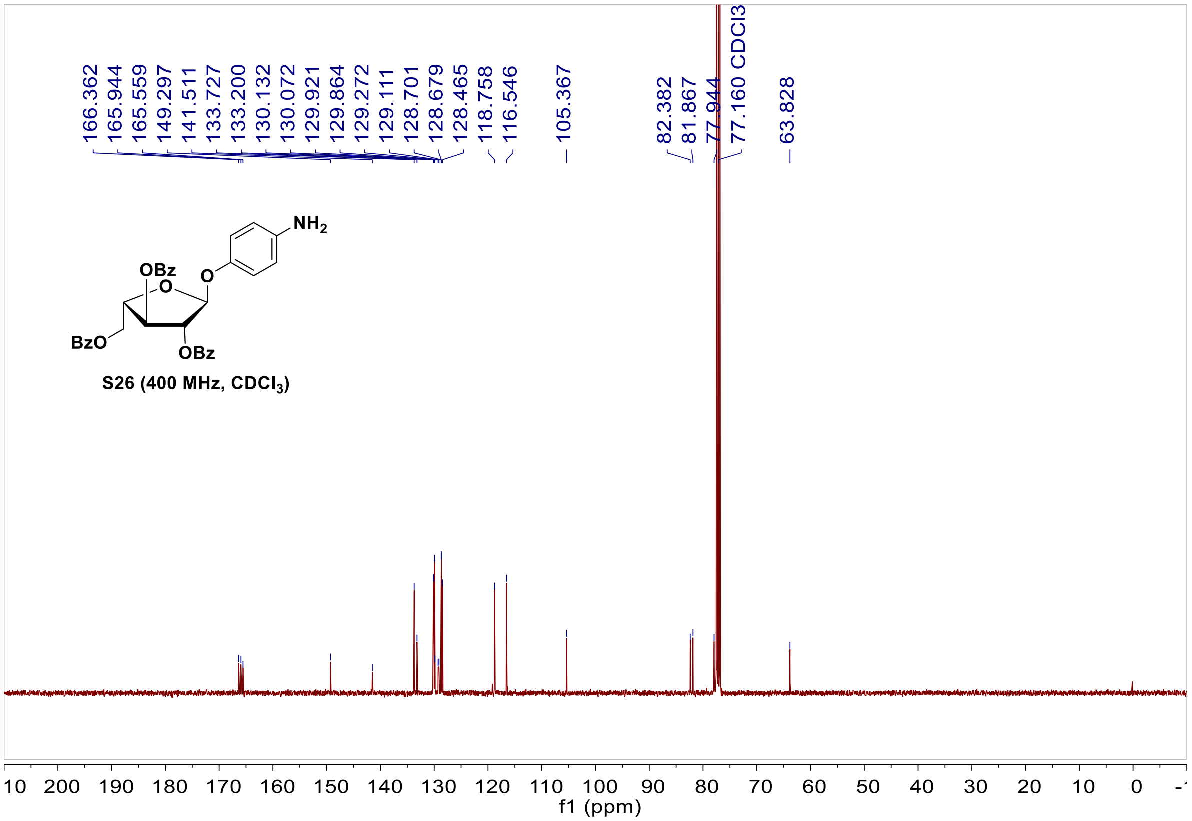
166.362
165.944
165.559
149.297
141.511
133.727
133.200
130.132
130.072
129.921
129.864
129.272
129.111
128.701
128.679
128.465
118.758
116.546
105.367
82.382
81.867
77.944
77.160 CDCl3
63.828
NH2
OBz
O
O
BzO
OBz
S26 (400 MHz, CDCl3)
f1 (ppm)

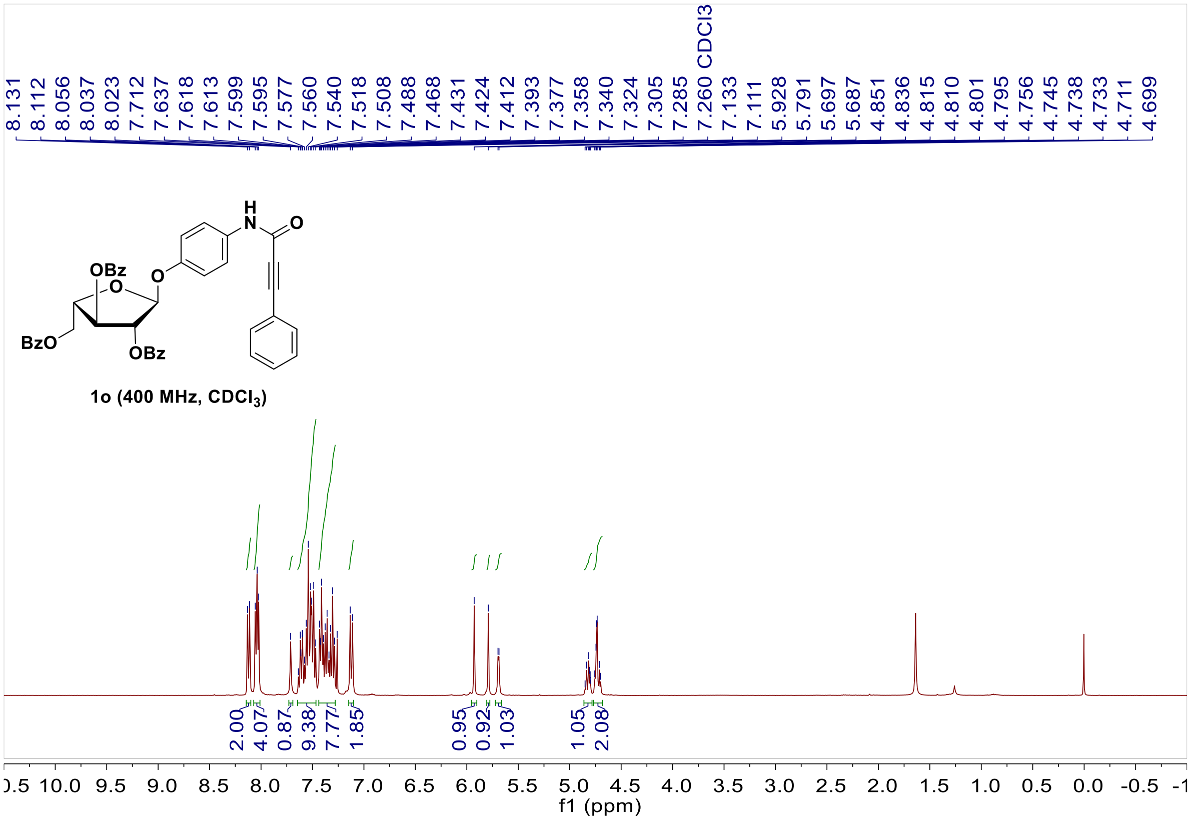
8.131 8.112 8.056 8.037 8.023 7.712 7.637 7.618 7.613 7.599 7.595 7.577 7.560 7.540 7.518 7.508 7.488 7.468 7.431 7.424 7.412 7.393 7.377 7.358 7.340 7.324 7.305 7.285 7.260 CDCl3 7.133 7.111 5.928 5.791 5.697 5.687 4.851 4.836 4.815 4.810 4.801 4.795 4.756 4.745 4.738 4.733 4.711 4.699
H
N
O
OBz
O
O
BzO
OBz
1o (400 MHz, CDCl3)
2.00 4.07 0.87 9.38 7.77 1.85 0.95 0.92 1.03 1.05 2.08
f1 (ppm)

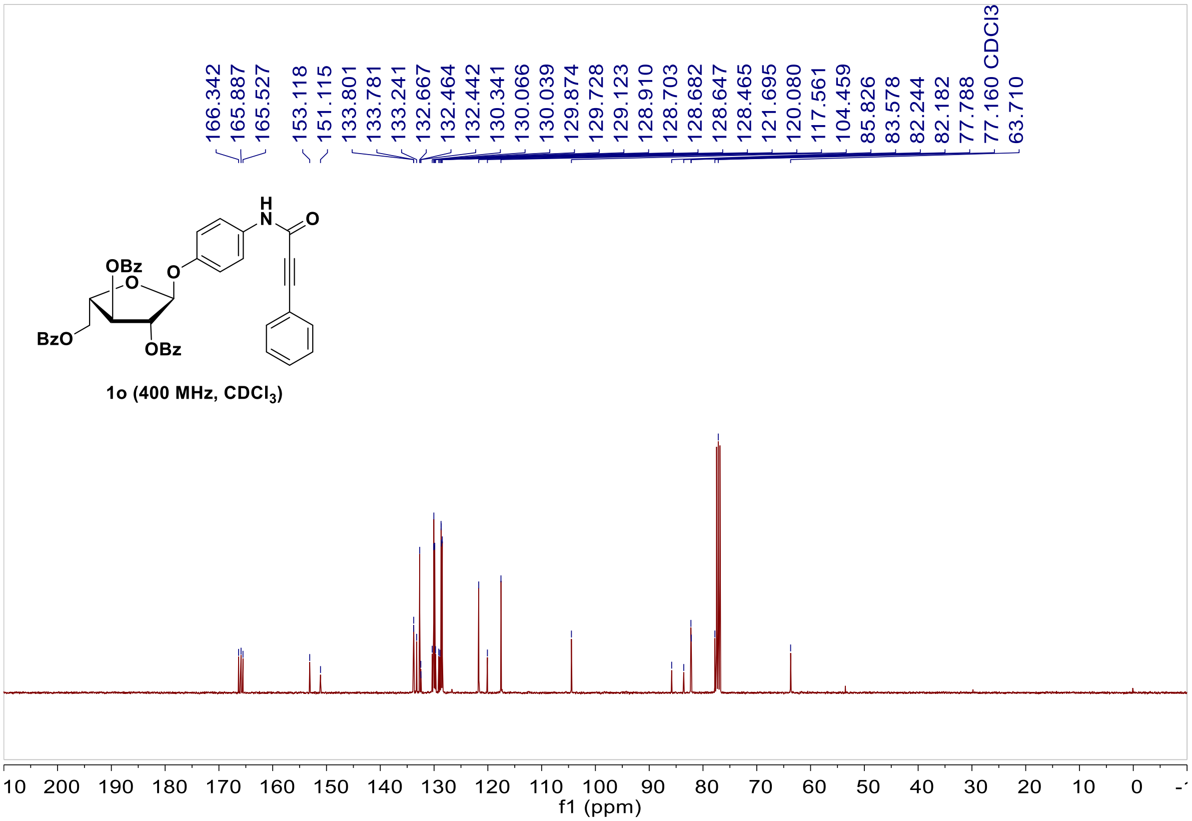
166.342 165.887 165.527 153.118 151.115 133.801 133.781 133.241 132.667 132.464 132.442 130.341 130.066 130.039 129.874 129.728 129.123 128.910 128.703 128.682 128.647 128.465 121.695 120.080 117.561 104.459 85.826 83.578 82.244 82.182 77.788 77.160 CDCl3 63.710
H
N
O
OBz
O
O
BzO
OBz
1o (400 MHz, CDCl3)
f1 (ppm)

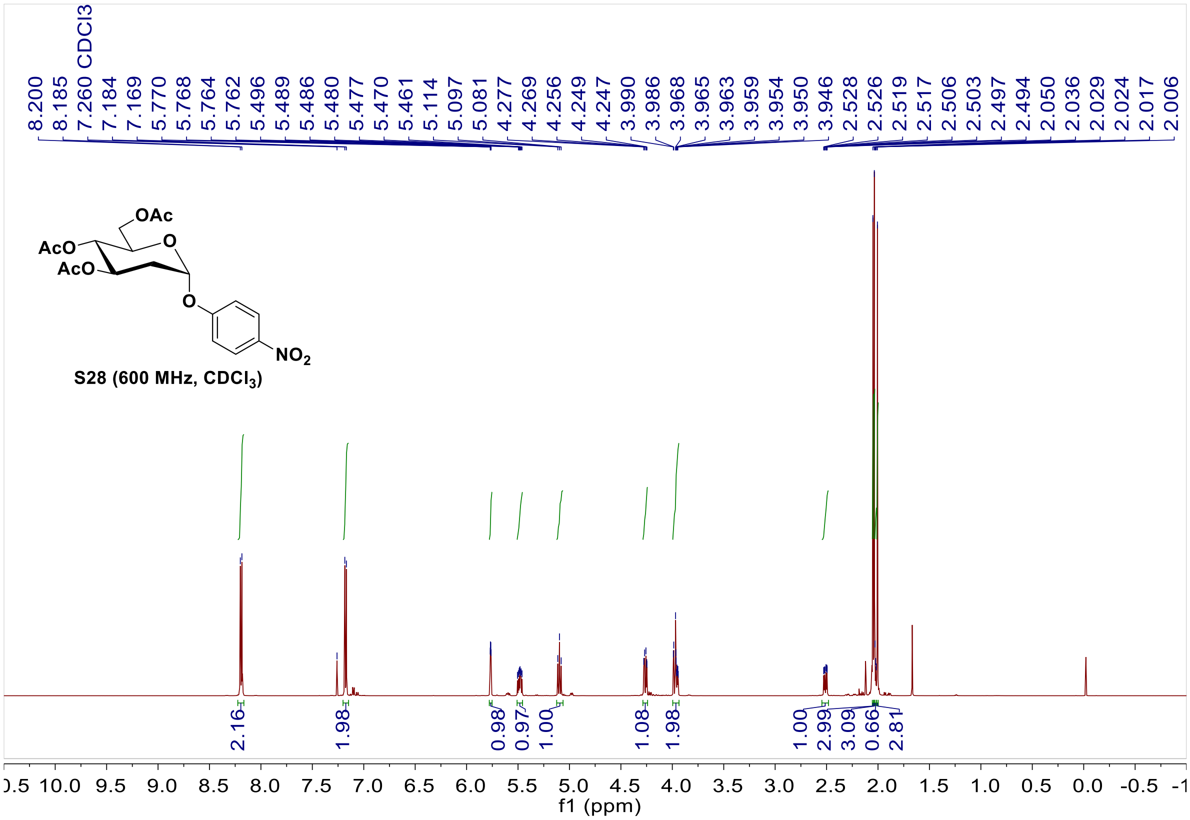
8.200
8.185
7.260 CDCl3
7.184
7.169
5.770
5.768
5.764
5.762
5.496
5.489
5.486
5.480
5.477
5.470
5.461
5.114
5.097
5.081
4.277
4.269
4.256
4.249
4.247
3.990
3.986
3.968
3.965
3.963
3.959
3.954
3.950
3.946
2.528
2.526
2.519
2.517
2.506
2.503
2.497
2.494
2.050
2.036
2.029
2.024
2.017
2.006
OAc
AcO
AcO
O
O
NO2
S28 (600 MHz, CDCl3)
2.16
1.98
0.98
0.97
1.00
1.08
1.98
1.00
2.99
3.09
0.66
2.81
f1 (ppm)

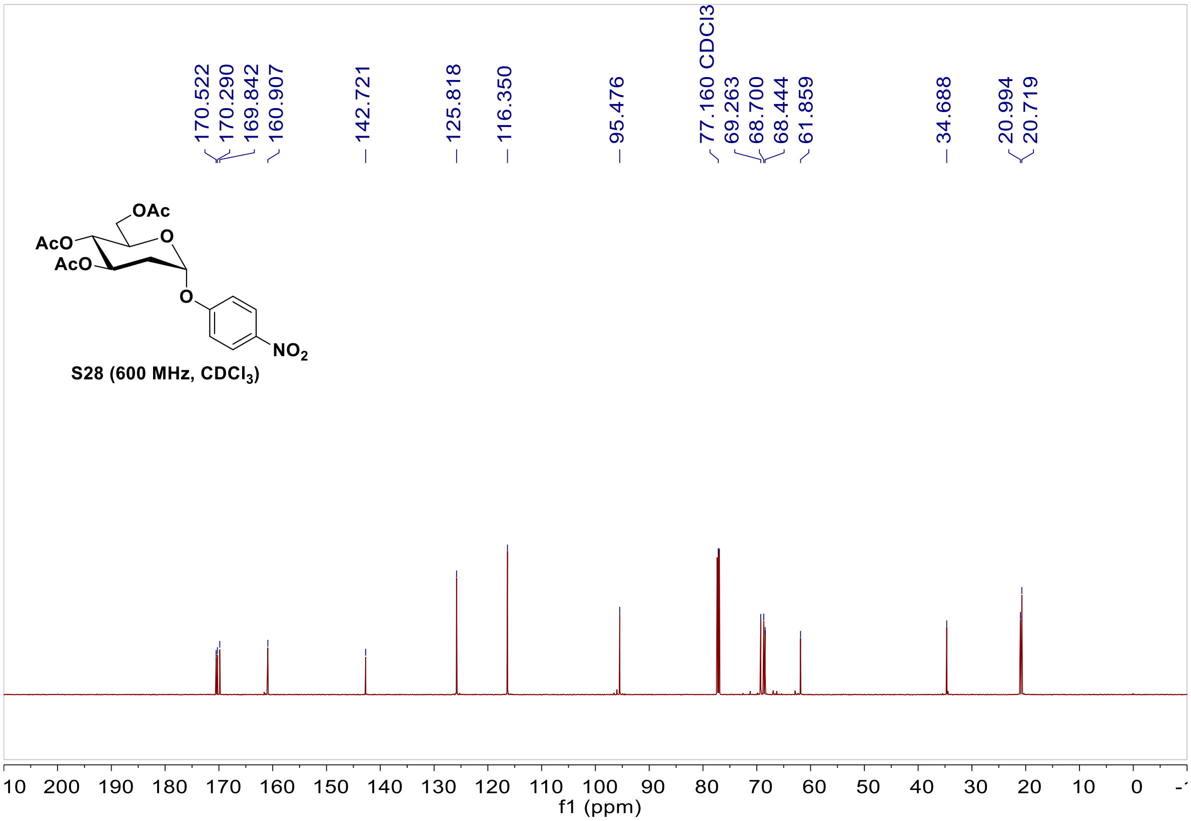
170.522
170.290
169.842
160.907
142.721
125.818
116.350
95.476
77.160 CDCl3
69.263
68.700
68.444
61.859
34.688
20.994
20.719
OAc
AcO
AcO
O
O
NO2
S28 (600 MHz, CDCl3)
f1 (ppm)

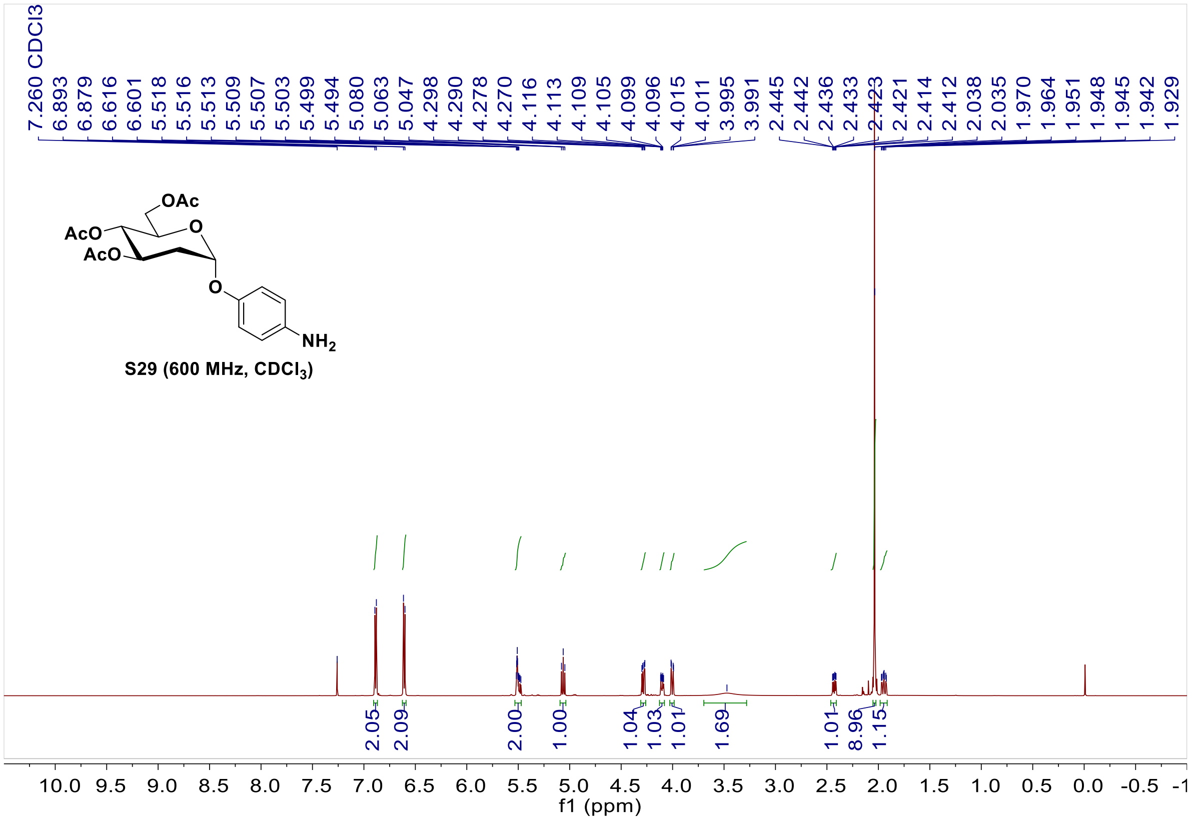
7.260 CDCl3
6.893
6.879
6.616
6.601
5.518
5.516
5.513
5.509
5.507
5.503
5.499
5.494
5.080
5.063
5.047
4.298
4.290
4.278
4.270
4.116
4.113
4.109
4.105
4.099
4.096
4.015
4.011
3.995
3.991
2.445
2.442
2.436
2.433
2.423
2.421
2.414
2.412
2.038
2.035
1.970
1.964
1.951
1.948
1.945
1.942
1.929
OAc
AcO
AcO
O
O
NH2
S29 (600 MHz, CDCl3)
2.05
2.09
2.00
1.00
1.04
1.03
1.01
1.69
1.01
8.96
1.15
10.0 9.5 9.0 8.5 8.0 7.5 7.0 6.5 6.0 5.5 5.0 4.5 4.0 3.5 3.0 2.5 2.0 1.5 1.0 0.5 0.0 -0.5 -1
f1 (ppm)

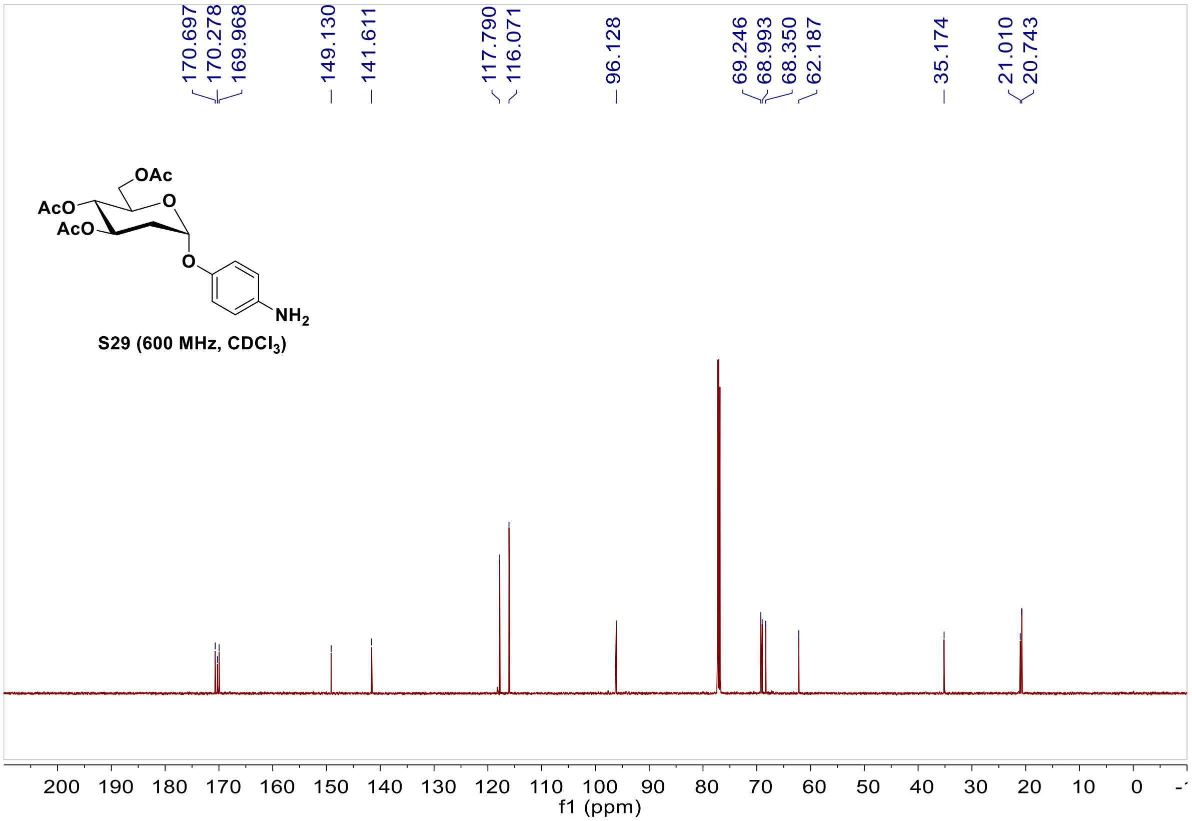
170.697
170.278
169.968
149.130
141.611
117.790
116.071
96.128
69.246
68.993
68.350
62.187
35.174
21.010
20.743
OAc
AcO
AcO
O
O
NH2
S29 (600 MHz, CDCl3)
200 190 180 170 160 150 140 130 120 110 100 90 80 70 60 50 40 30 20 10 0
f1 (ppm)

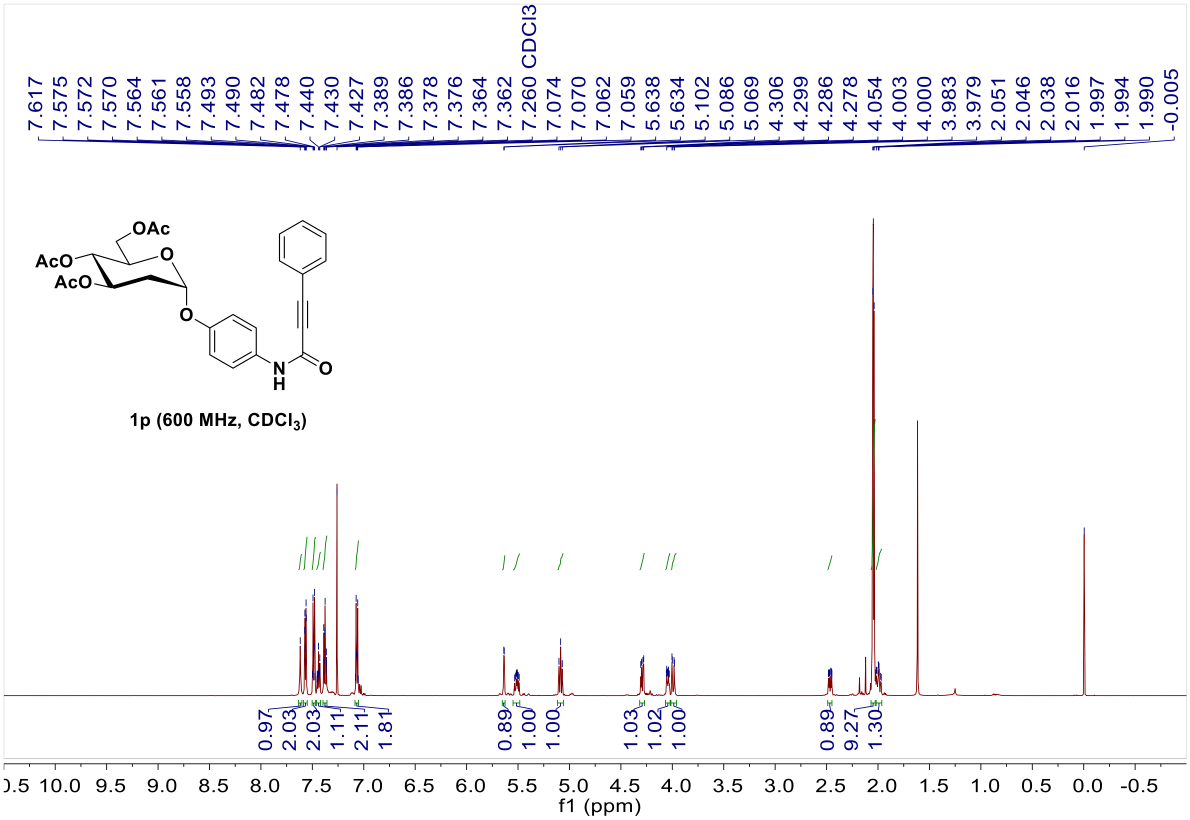
7.617
7.575
7.572
7.570
7.564
7.561
7.558
7.493
7.490
7.482
7.478
7.440
7.430
7.427
7.389
7.386
7.378
7.376
7.364
7.362
7.260 CDCl3
7.074
7.070
7.062
7.059
5.638
5.634
5.102
5.086
5.069
4.306
4.299
4.286
4.278
4.054
4.003
4.000
3.983
3.979
2.051
2.046
2.038
2.016
1.997
1.994
1.990
-0.005
OAc
AcO
AcO
O
O
N
H
O
1p (600 MHz, CDCl3)
0.97
2.03
2.03
1.11
2.11
1.81
0.89
1.00
1.00
1.03
1.02
1.00
0.89
9.27
1.30
f1 (ppm)

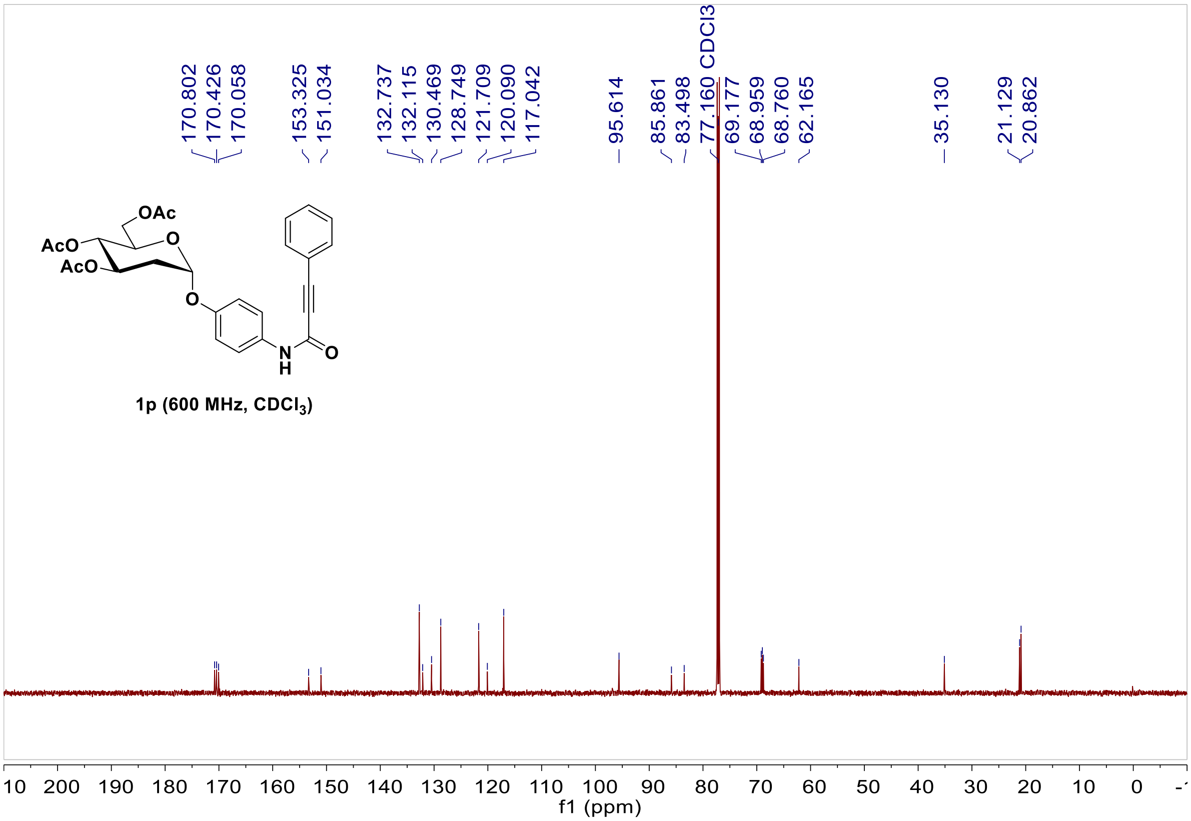
170.802
170.426
170.058
153.325
151.034
132.737
132.115
130.469
128.749
121.709
120.090
117.042
95.614
85.861
83.498
77.160 CDCl3
69.177
68.959
68.760
62.165
35.130
21.129
20.862
OAc
AcO
AcO
O
O
N
H
O
1p (600 MHz, CDCl3)
f1 (ppm)

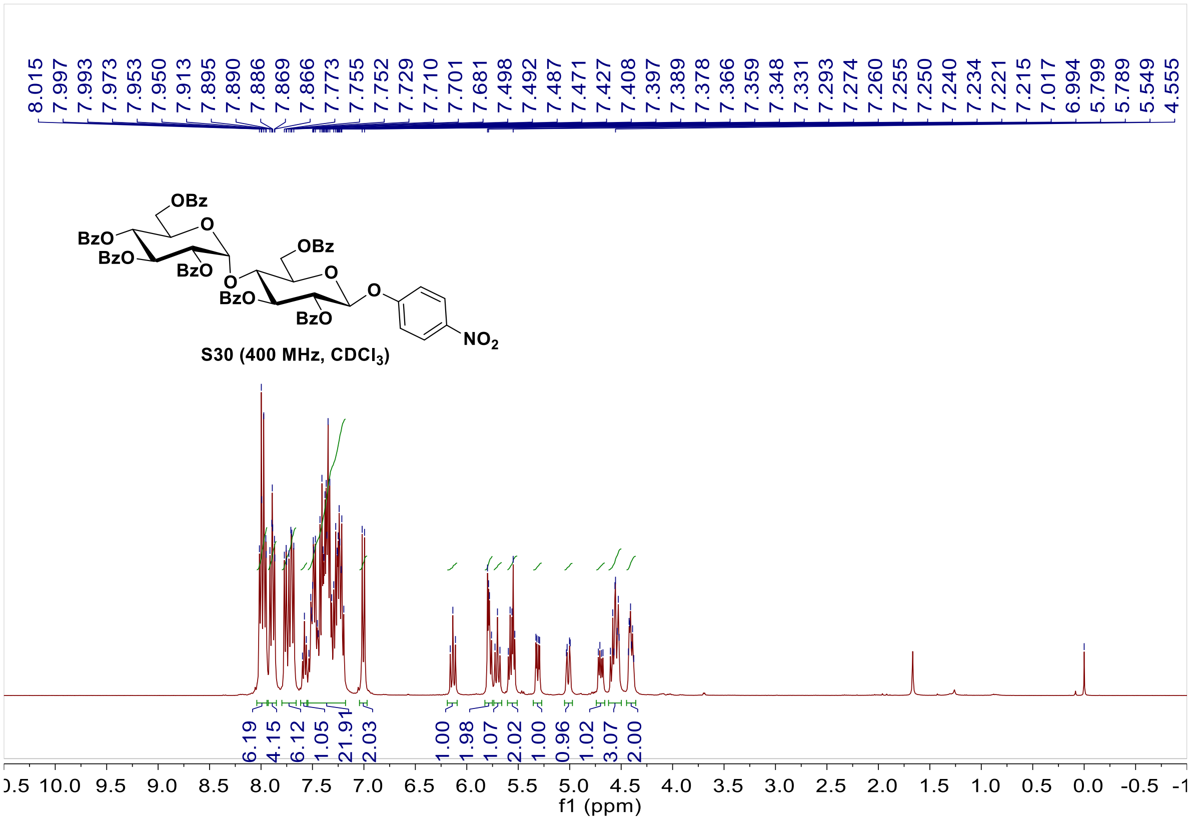
S30 (400 MHz, CDCl3)
f1 (ppm)

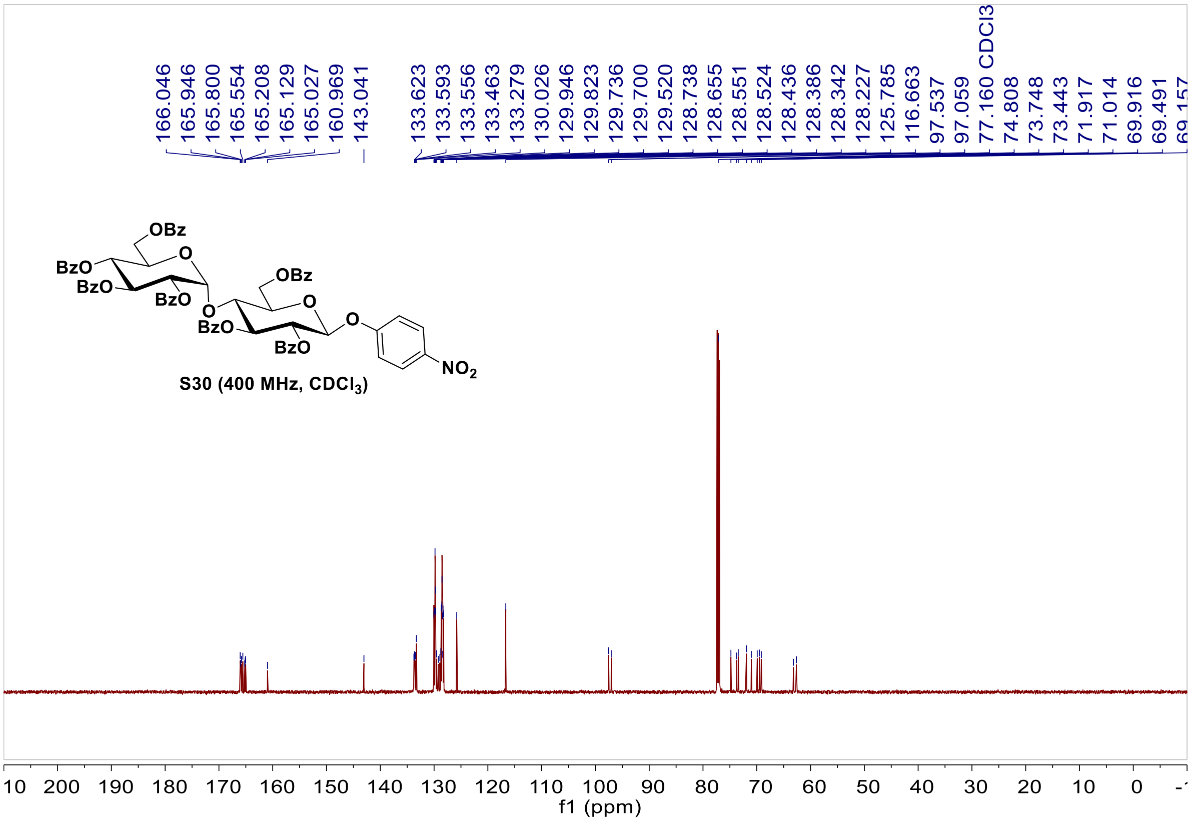
S30 (400 MHz, CDCl3)
77.160 CDCl3
f1 (ppm)

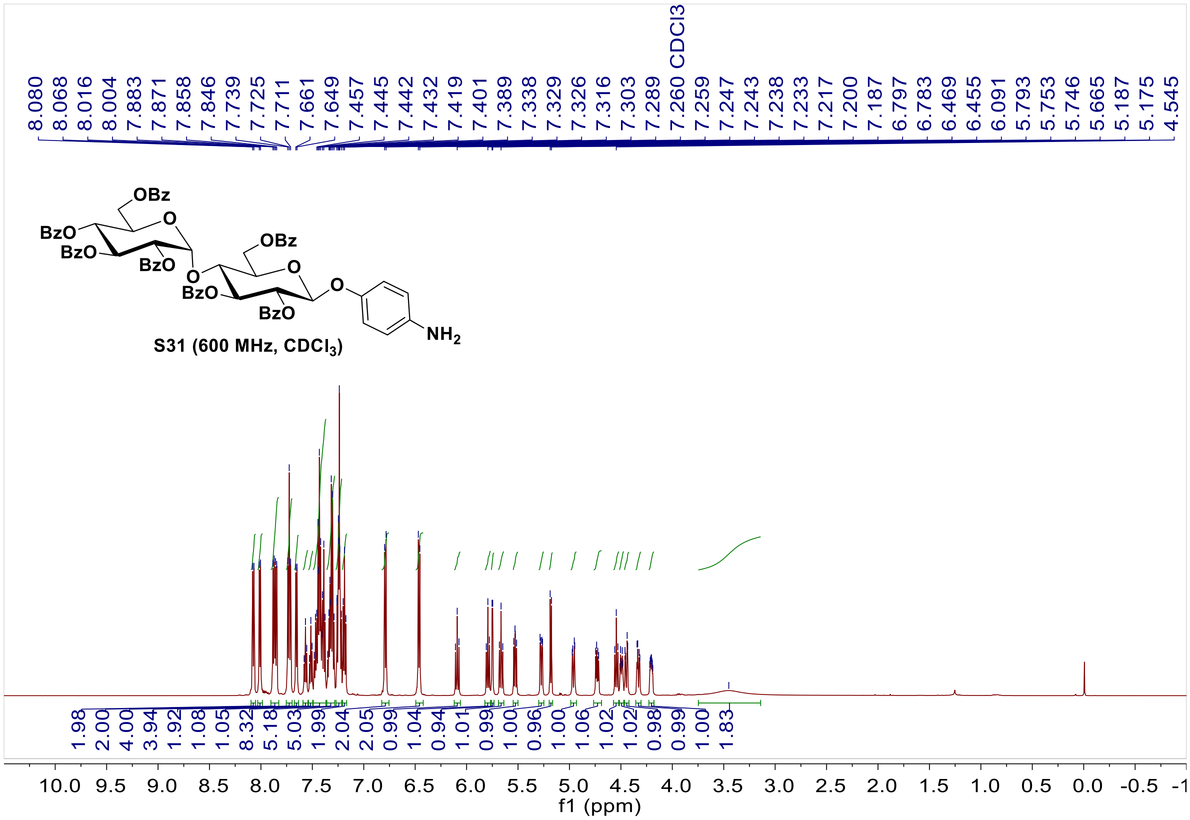

8.080 8.068 8.016 8.004 7.883 7.871 7.858 7.846 7.739 7.725 7.711 7.661 7.649 7.457 7.445 7.442 7.432 7.419 7.401 7.389 7.338 7.329 7.326 7.316 7.303 7.289 7.260 CDCl3 7.259 7.247 7.243 7.238 7.233 7.217 7.200 7.187 6.797 6.783 6.469 6.455 6.091 5.793 5.753 5.746 5.665 5.187 5.175 4.545
OBz
BzO
O
BzO
BzO
O
OBz
O
O
BzO
BzO
NH2
S31 (600 MHz, CDCl3)
1.98 2.00 4.00 3.94 1.92 1.08 1.05 8.32 5.18 5.03 1.99 2.04 2.05 0.99 1.04 0.94 1.01 0.99 1.00 0.96 1.00 1.06 1.02 1.02 0.98 0.99 1.00 1.83
10.0 9.5 9.0 8.5 8.0 7.5 7.0 6.5 6.0 5.5 5.0 4.5 4.0 3.5 3.0 2.5 2.0 1.5 1.0 0.5 0.0 -0.5 -1
f1 (ppm)

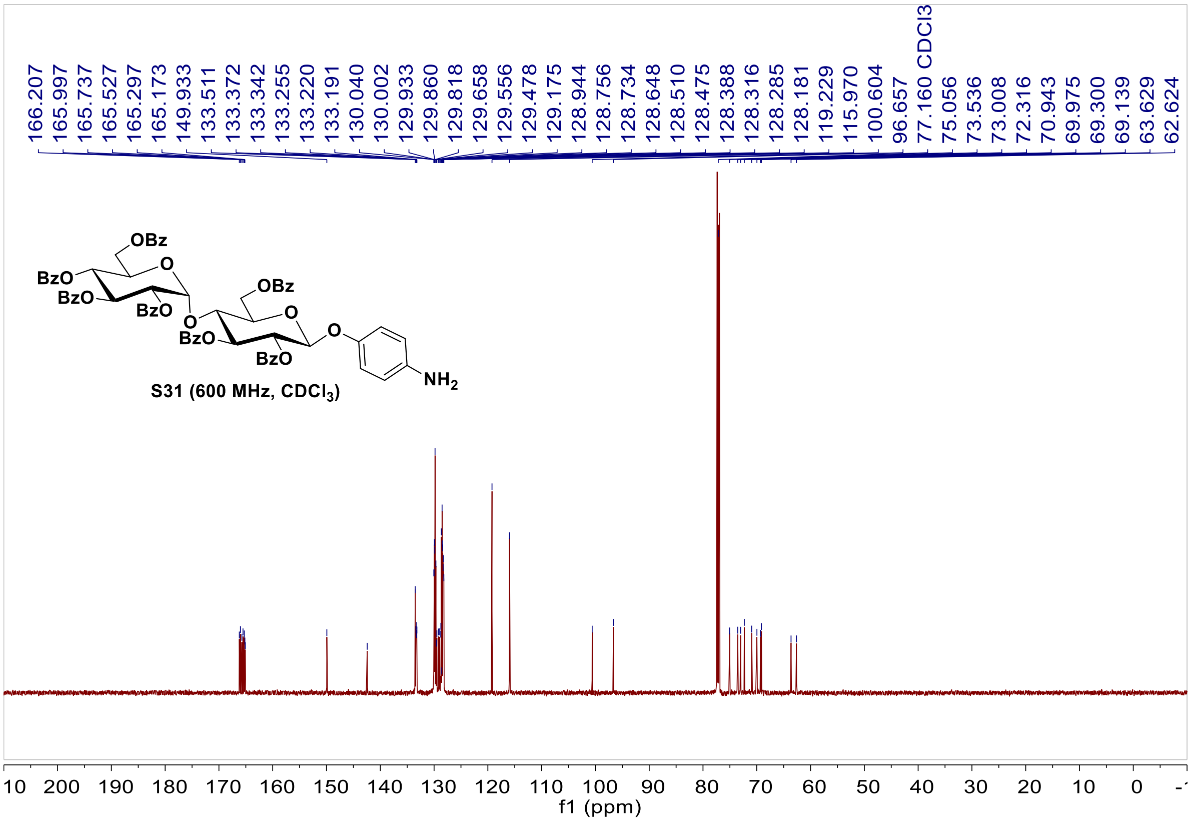

166.207 165.997 165.737 165.527 165.297 165.173 149.933 133.511 133.372 133.342 133.255 133.220 133.191 130.040 130.002 129.933 129.860 129.818 129.658 129.556 129.478 129.175 128.944 128.756 128.734 128.648 128.510 128.475 128.388 128.316 128.285 128.181 119.229 115.970 100.604 96.657 77.160 CDCl3 75.056 73.536 73.008 72.316 70.943 69.975 69.300 69.139 63.629 62.624
OBz
BzO
O
BzO
BzO
O
OBz
O
O
BzO
BzO
NH2
S31 (600 MHz, CDCl3)
10 200 190 180 170 160 150 140 130 120 110 100 90 80 70 60 50 40 30 20 10 0
f1 (ppm)

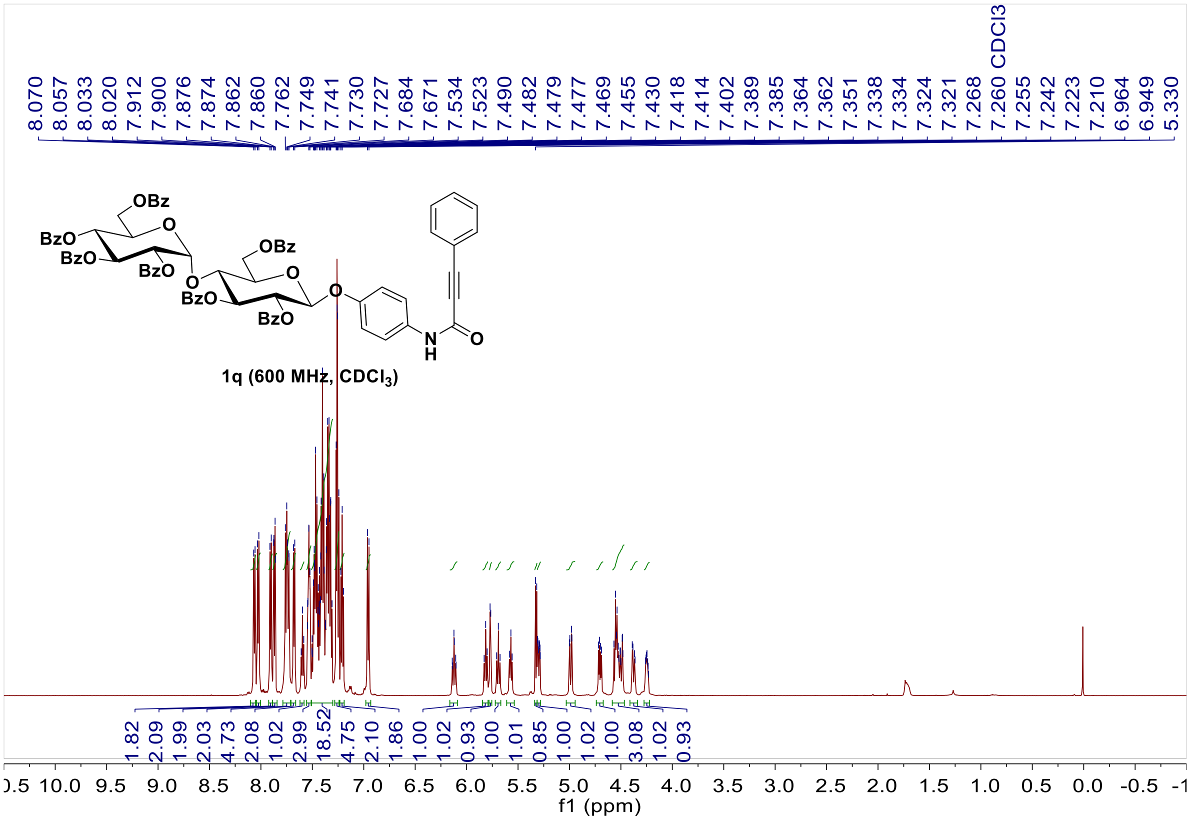
8.070 8.057 8.033 8.020 7.912 7.900 7.876 7.874 7.862 7.860 7.762 7.749 7.741 7.730 7.727 7.684 7.671 7.534 7.523 7.490 7.482 7.479 7.477 7.469 7.455 7.430 7.418 7.414 7.402 7.389 7.385 7.364 7.362 7.351 7.338 7.334 7.324 7.321 7.268 7.260 CDCl3 7.255 7.242 7.223 7.210 6.964 6.949 5.330
OBz
BzO
BzO
BzO
O
OBz
BzO
BzO
O
N
H
O
1q (600 MHz, CDCl$_3$)
1.82 2.09 1.99 2.03 4.73 2.08 1.02 2.99 18.52 4.75 2.10 1.86 1.00 1.02 0.93 1.00 1.01 0.85 1.00 1.02 1.00 3.08 1.02 0.93
f1 (ppm)

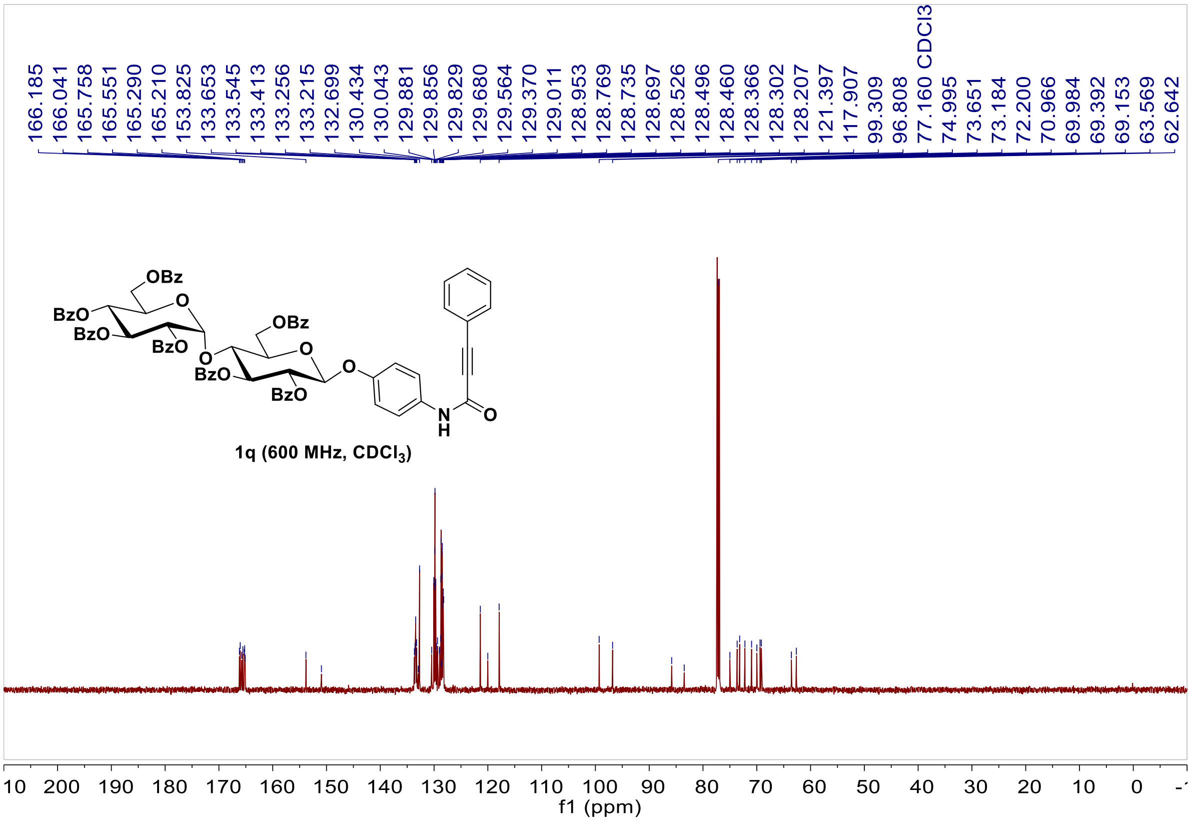
166.185 166.041 165.758 165.551 165.290 165.210 153.825 133.653 133.545 133.413 133.256 133.215 132.699 130.434 130.043 129.881 129.856 129.829 129.680 129.564 129.370 129.011 128.953 128.769 128.735 128.697 128.526 128.496 128.460 128.366 128.302 128.207 121.397 117.907 99.309 96.808 77.160 CDCl3 74.995 73.651 73.184 72.200 70.966 69.984 69.392 69.153 63.569 62.642
OBz
BzO
BzO
BzO
O
OBz
BzO
BzO
O
N
H
O
1q (600 MHz, CDCl$_3$)
f1 (ppm)

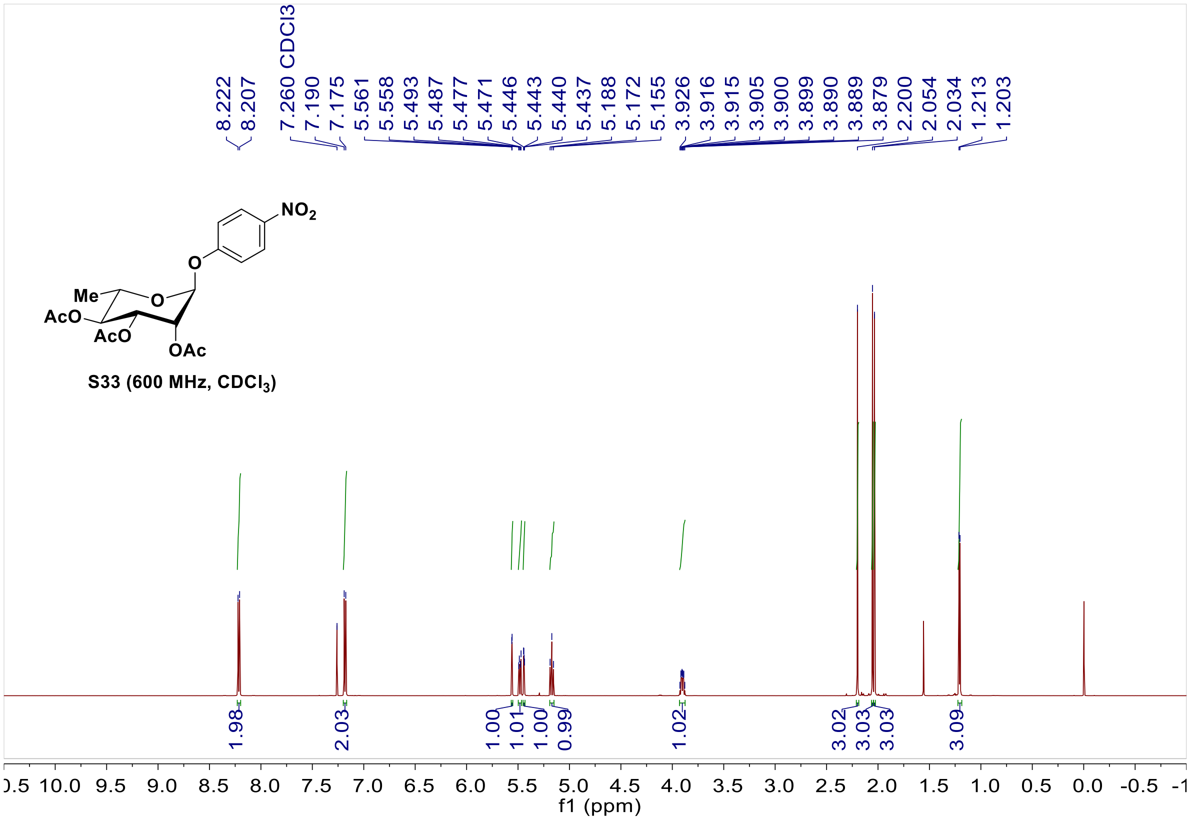
8.222
8.207
7.260 CDCl3
7.190
7.175
5.561
5.558
5.493
5.487
5.477
5.471
5.446
5.443
5.440
5.437
5.188
5.172
5.155
3.926
3.916
3.915
3.905
3.900
3.899
3.890
3.889
3.879
2.200
2.054
2.034
1.213
1.203
NO2
O
Me
O
AcO
AcO
OAc
S33 (600 MHz, CDCl3)
1.98
2.03
1.00
1.01
1.00
0.99
1.02
3.02
3.03
3.03
3.09
f1 (ppm)

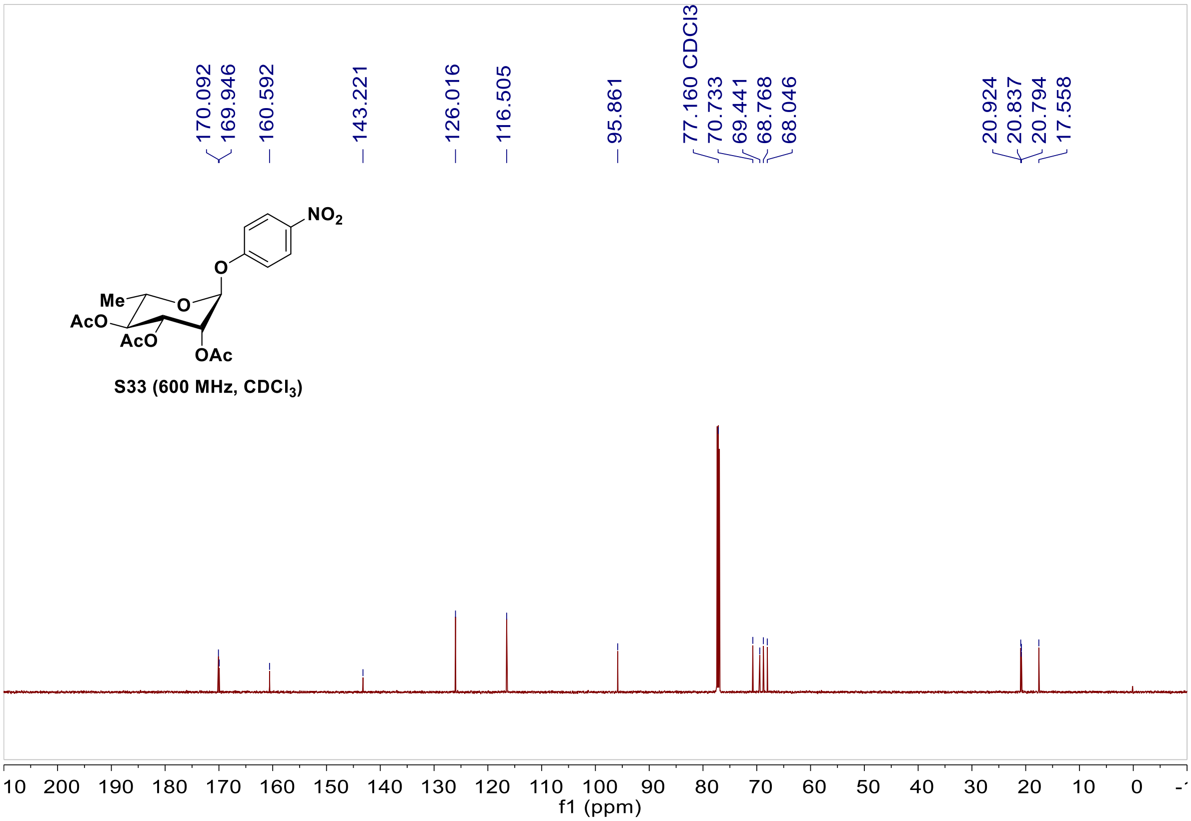
170.092
169.946
160.592
143.221
126.016
116.505
95.861
77.160 CDCl3
70.733
69.441
68.768
68.046
20.924
20.837
20.794
17.558
NO2
O
Me
O
AcO
AcO
OAc
S33 (600 MHz, CDCl3)
f1 (ppm)

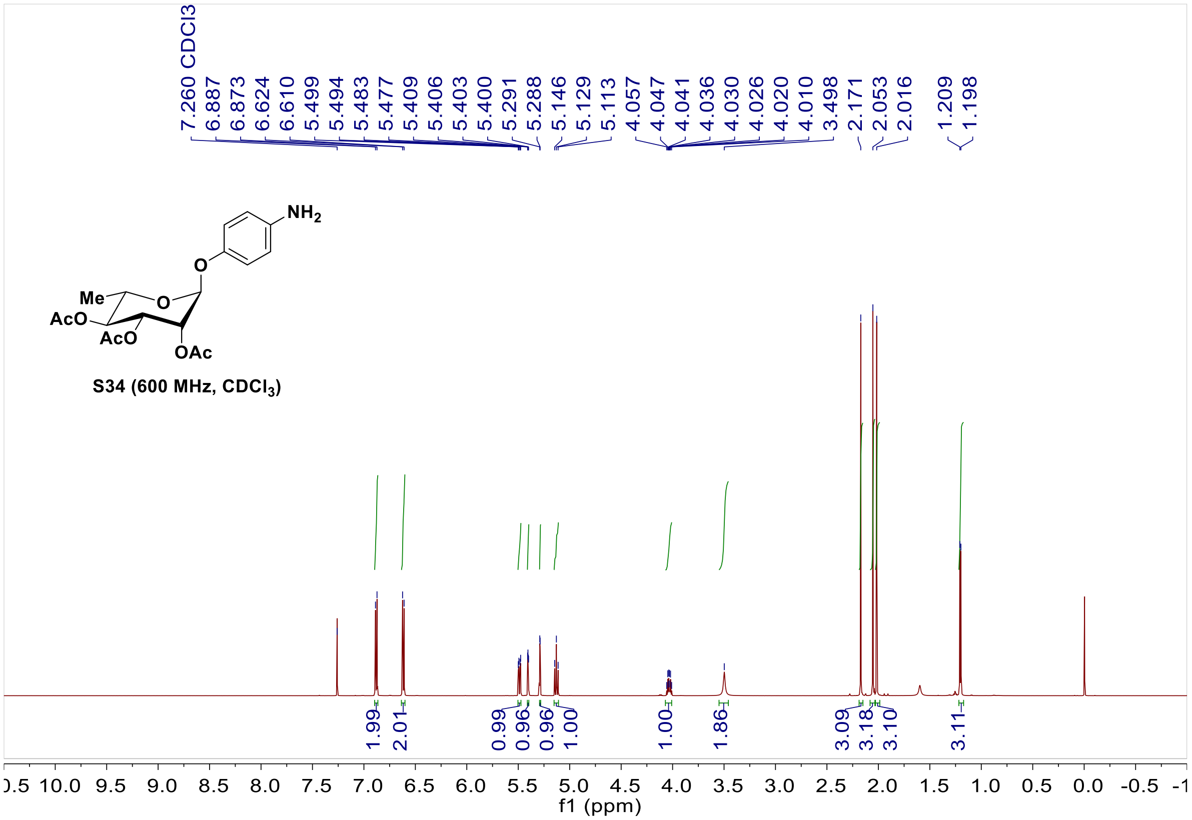
7.260 CDCl3
6.887
6.873
6.624
6.610
5.499
5.494
5.483
5.477
5.409
5.406
5.403
5.400
5.291
5.288
5.146
5.129
5.113
4.057
4.047
4.041
4.036
4.030
4.026
4.020
4.010
3.498
2.171
2.053
2.016
1.209
1.198
NH2
O
Me
O
AcO
AcO
OAc
S34 (600 MHz, CDCl3)
1.99
2.01
0.99
0.96
0.96
1.00
1.00
1.86
3.09
3.18
3.10
3.11
f1 (ppm)

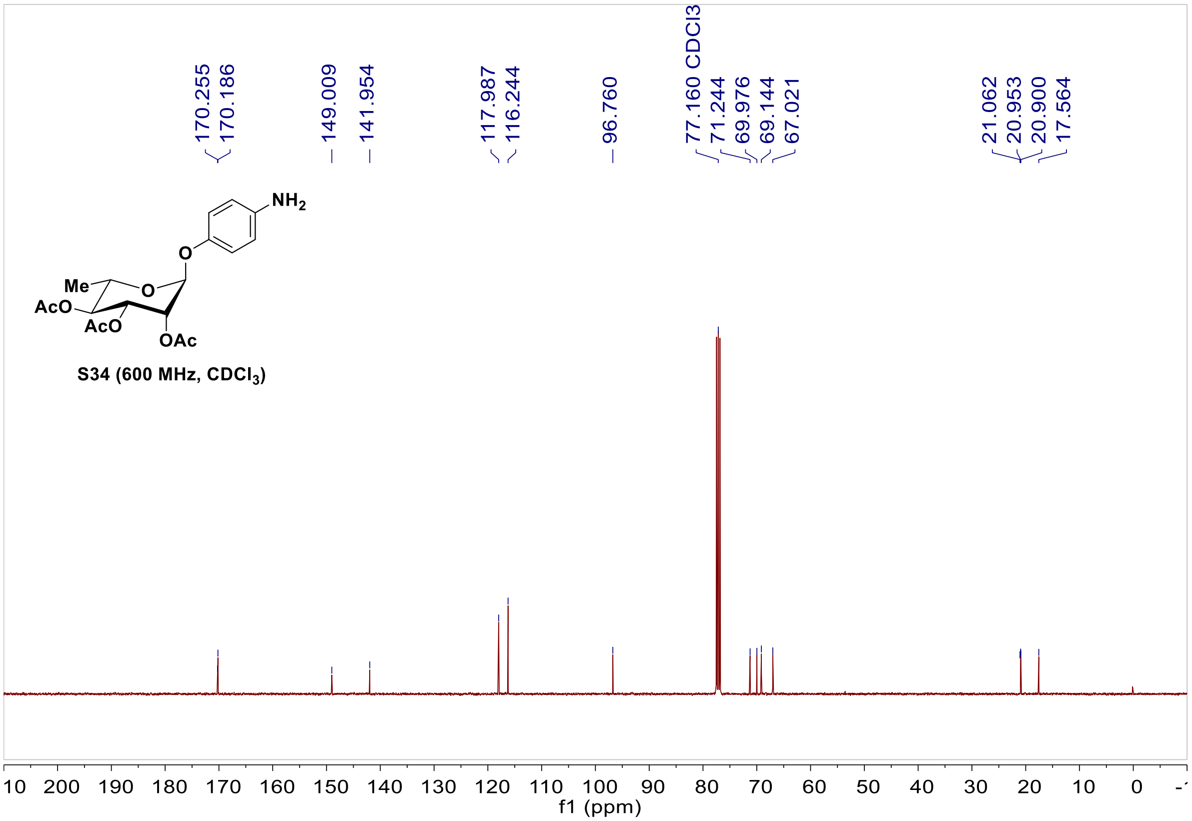
170.255
170.186
149.009
141.954
117.987
116.244
96.760
77.160 CDCl3
71.244
69.976
69.144
67.021
21.062
20.953
20.900
17.564
NH2
O
Me
O
AcO
AcO
OAc
S34 (600 MHz, CDCl3)
f1 (ppm)

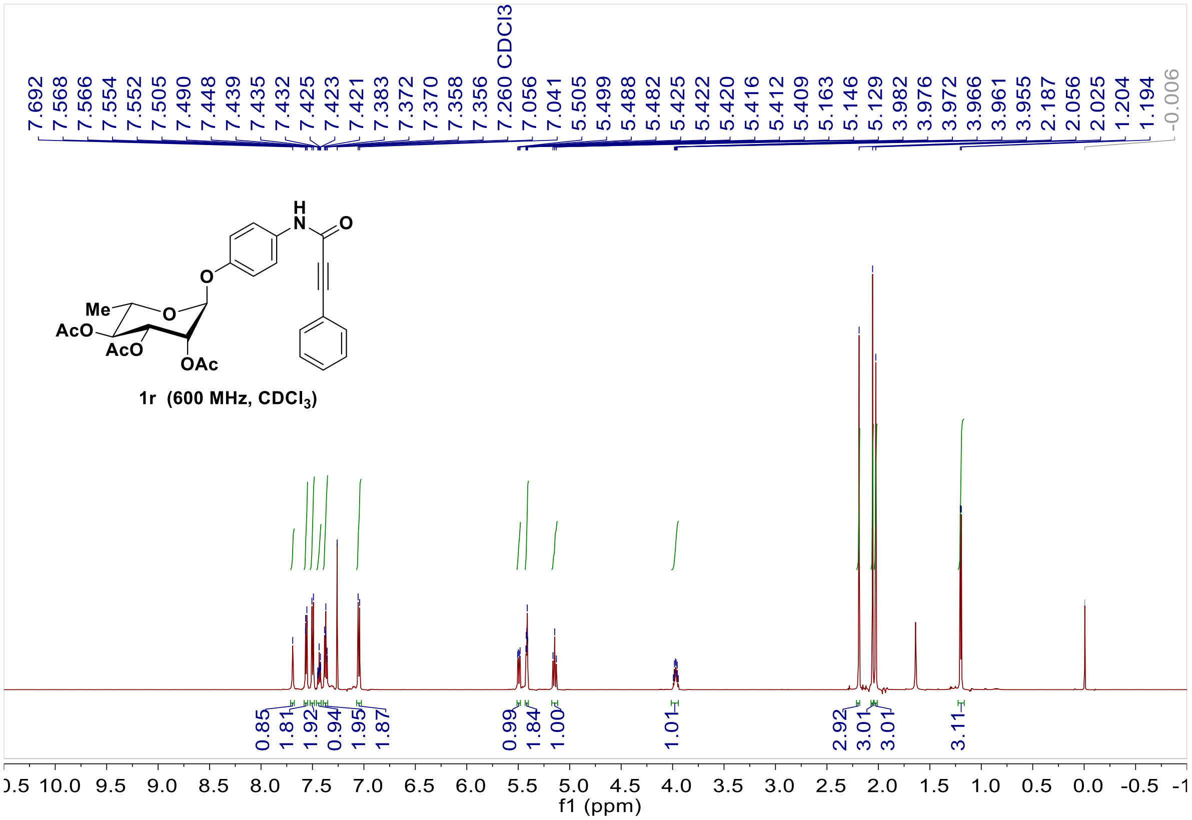
7.692 7.568 7.566 7.554 7.552 7.505 7.490 7.448 7.439 7.435 7.432 7.425 7.423 7.421 7.383 7.372 7.370 7.358 7.356 7.260 CDCl3 7.056 7.041 5.505 5.499 5.488 5.482 5.425 5.422 5.420 5.416 5.412 5.409 5.163 5.146 5.129 3.982 3.976 3.972 3.966 3.961 3.955 2.187 2.056 2.025 1.204 1.194 -0.006
H
N
O
O
Me
O
AcO
AcO
OAc
1r (600 MHz, $CDCl_3$)
0.85 1.81 1.92 0.94 1.95 1.87 0.99 1.84 1.00 1.01 2.92 3.01 3.01 3.11
10.5 10.0 9.5 9.0 8.5 8.0 7.5 7.0 6.5 6.0 5.5 5.0 4.5 4.0 3.5 3.0 2.5 2.0 1.5 1.0 0.5 0.0 -0.5 -1
f1 (ppm)
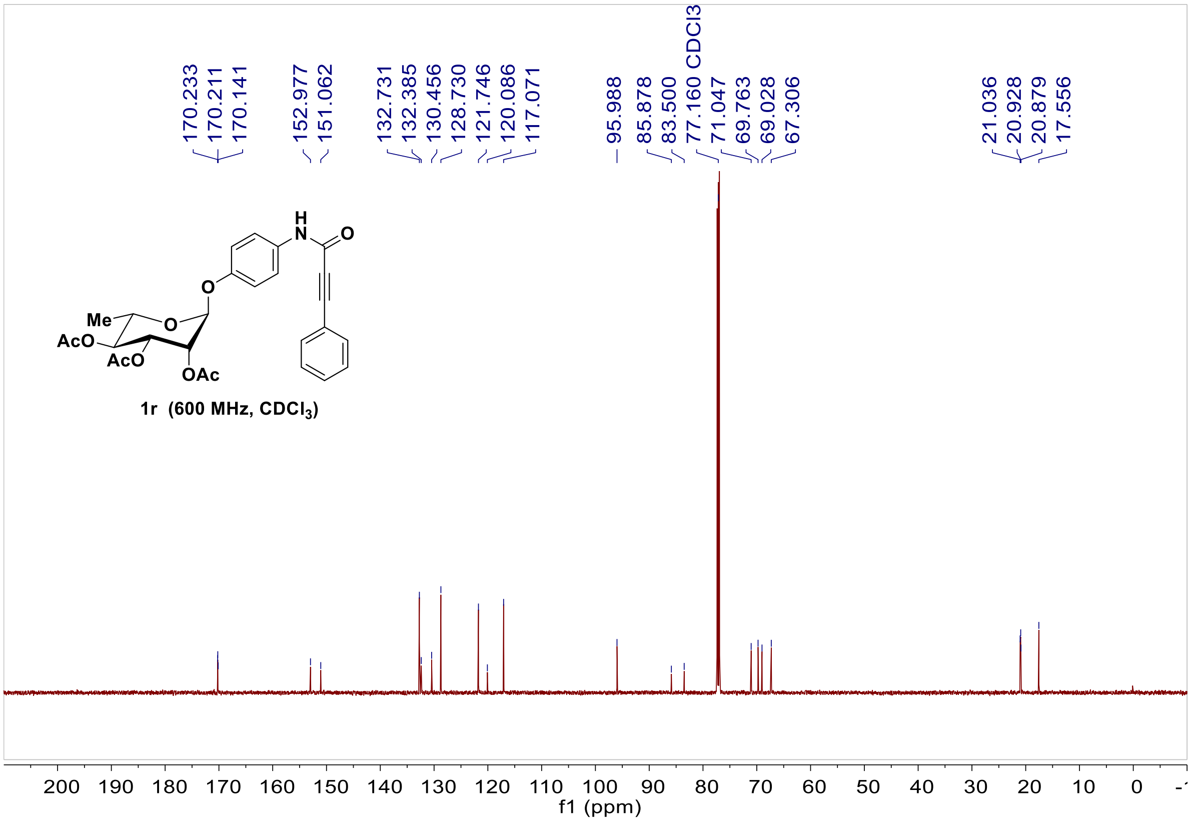
170.233 170.211 170.141 152.977 151.062 132.731 132.385 130.456 128.730 121.746 120.086 117.071 95.988 85.878 83.500 77.160 CDCl3 71.047 69.763 69.028 67.306 21.036 20.928 20.879 17.556
H
N
O
O
Me
O
AcO
AcO
OAc
1r (600 MHz, $CDCl_3$)
200 190 180 170 160 150 140 130 120 110 100 90 80 70 60 50 40 30 20 10 0
f1 (ppm)

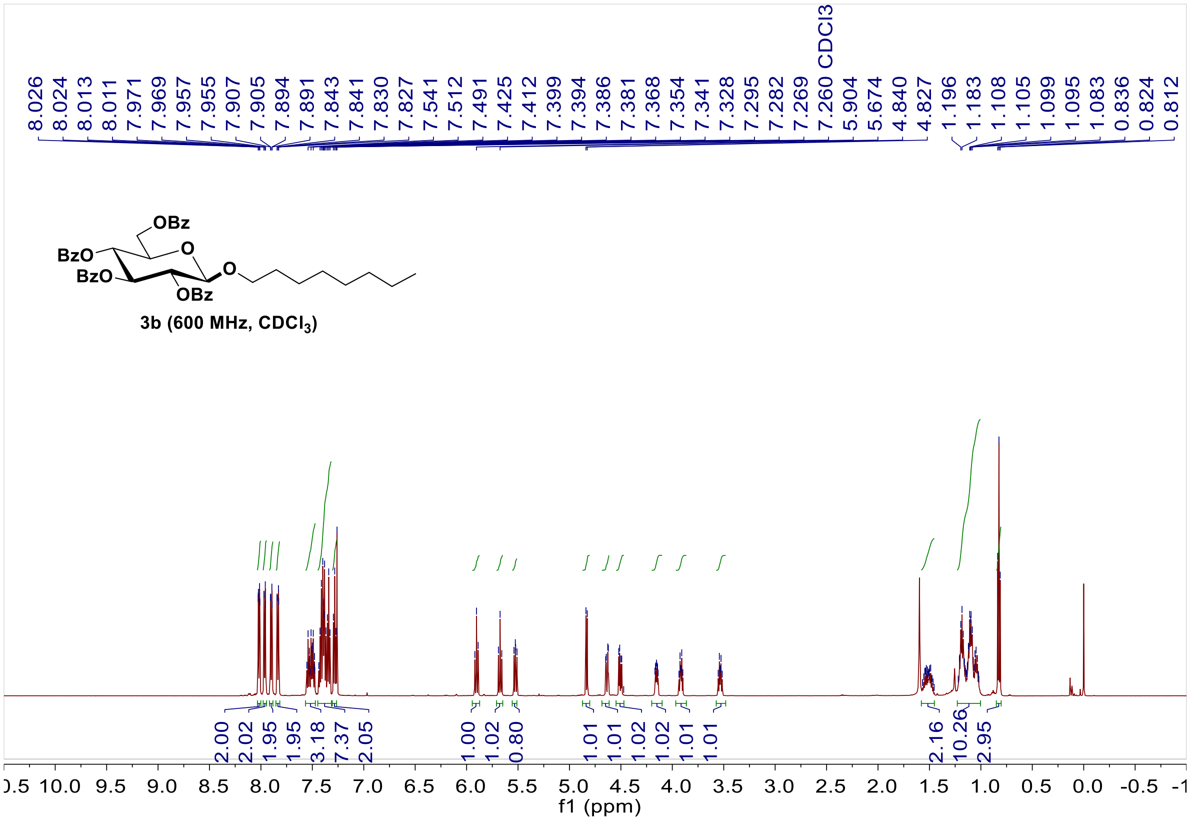

3b (600 MHz, $CDCl_3$)

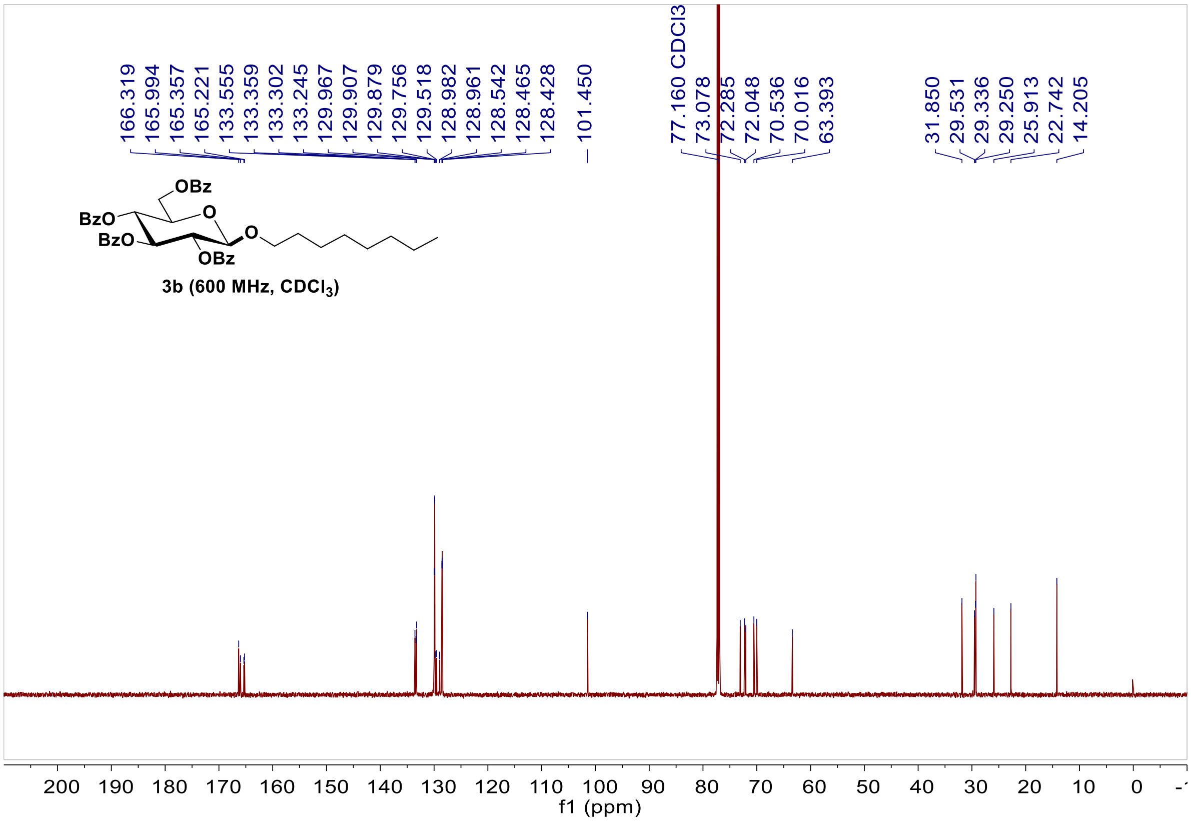

3b (600 MHz, $CDCl_3$)

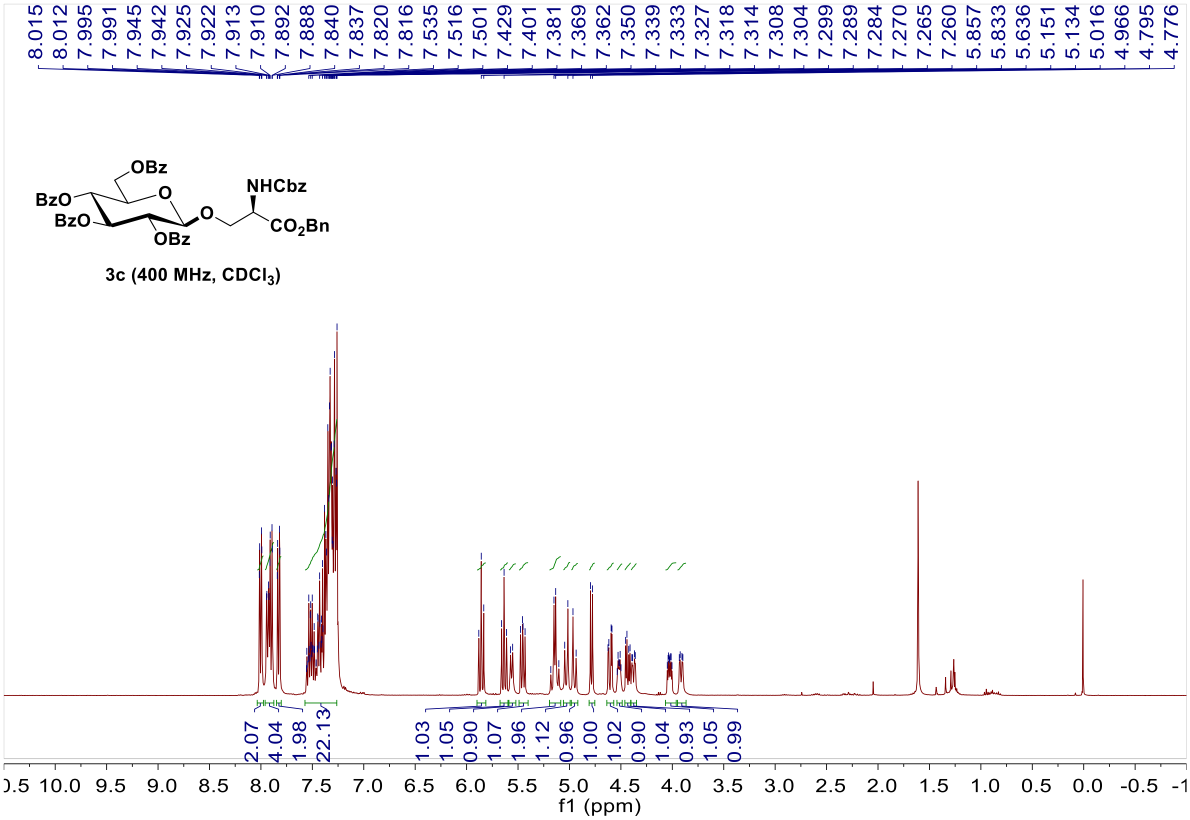

3c (400 MHz, CDCl3)
f1 (ppm)

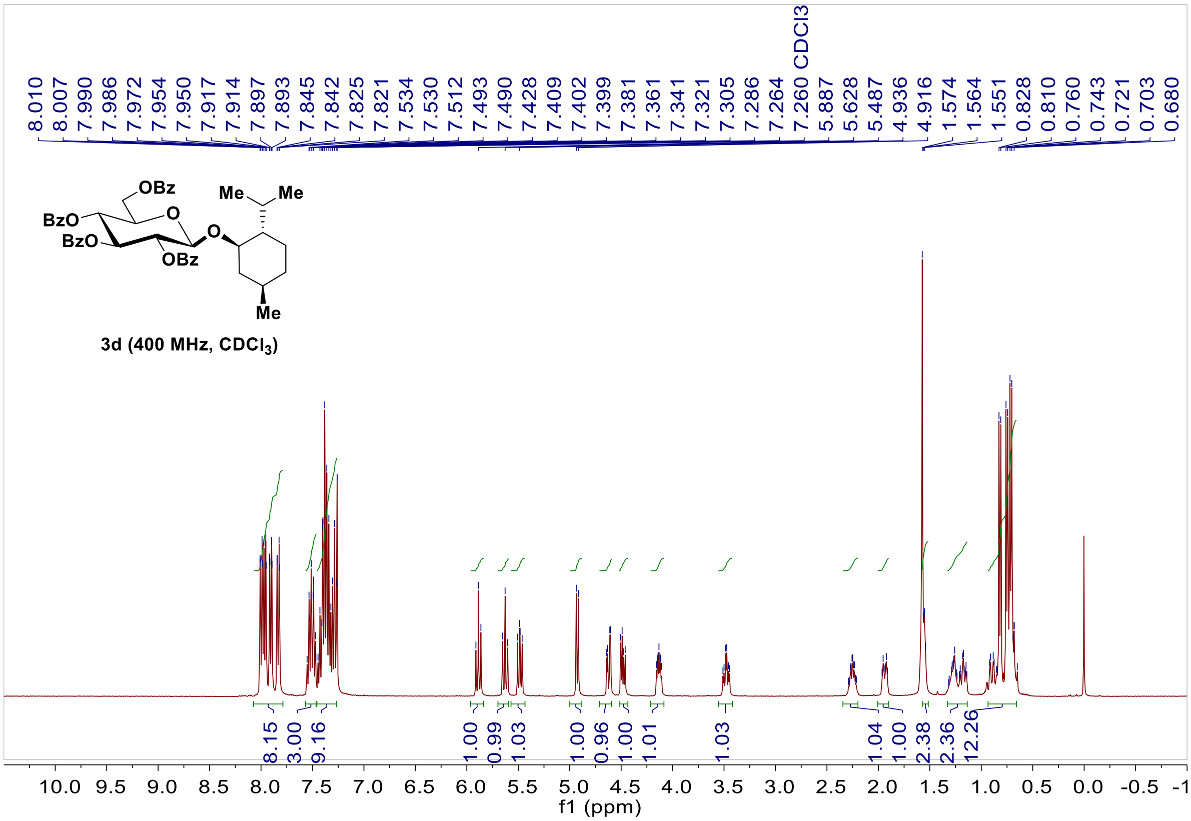

3d (400 MHz, CDCl3)
CDCl3
f1 (ppm)

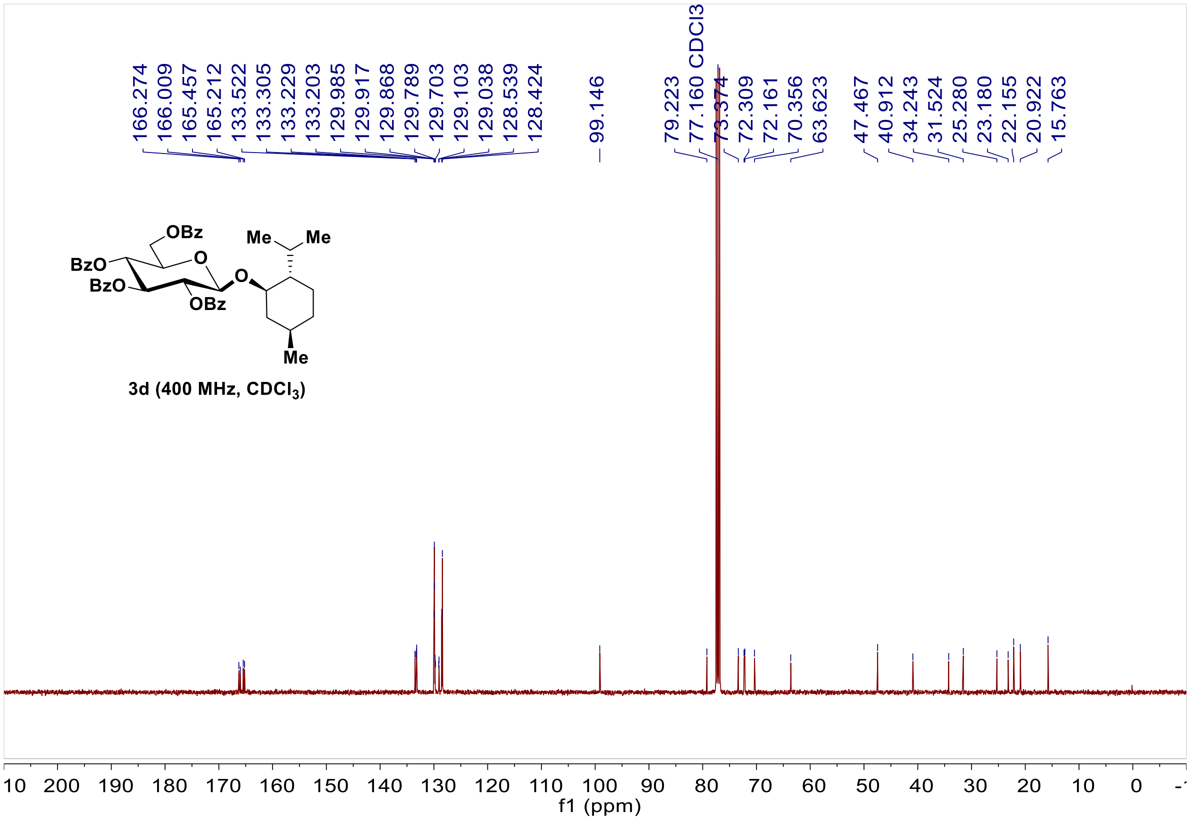
166.274
166.009
165.457
165.212
133.522
133.305
133.229
133.203
129.985
129.917
129.868
129.789
129.703
129.103
129.038
128.539
128.424
99.146
79.223
77.160 CDCl3
73.374
72.309
72.161
70.356
63.623
47.467
40.912
34.243
31.524
25.280
23.180
22.155
20.922
15.763
OBz
BzO
BzO
OBz
O
O
Me
Me
Me
3d (400 MHz, CDCl3)
10 200 190 180 170 160 150 140 130 120 110 100 90 80 70 60 50 40 30 20 10 0
f1 (ppm)

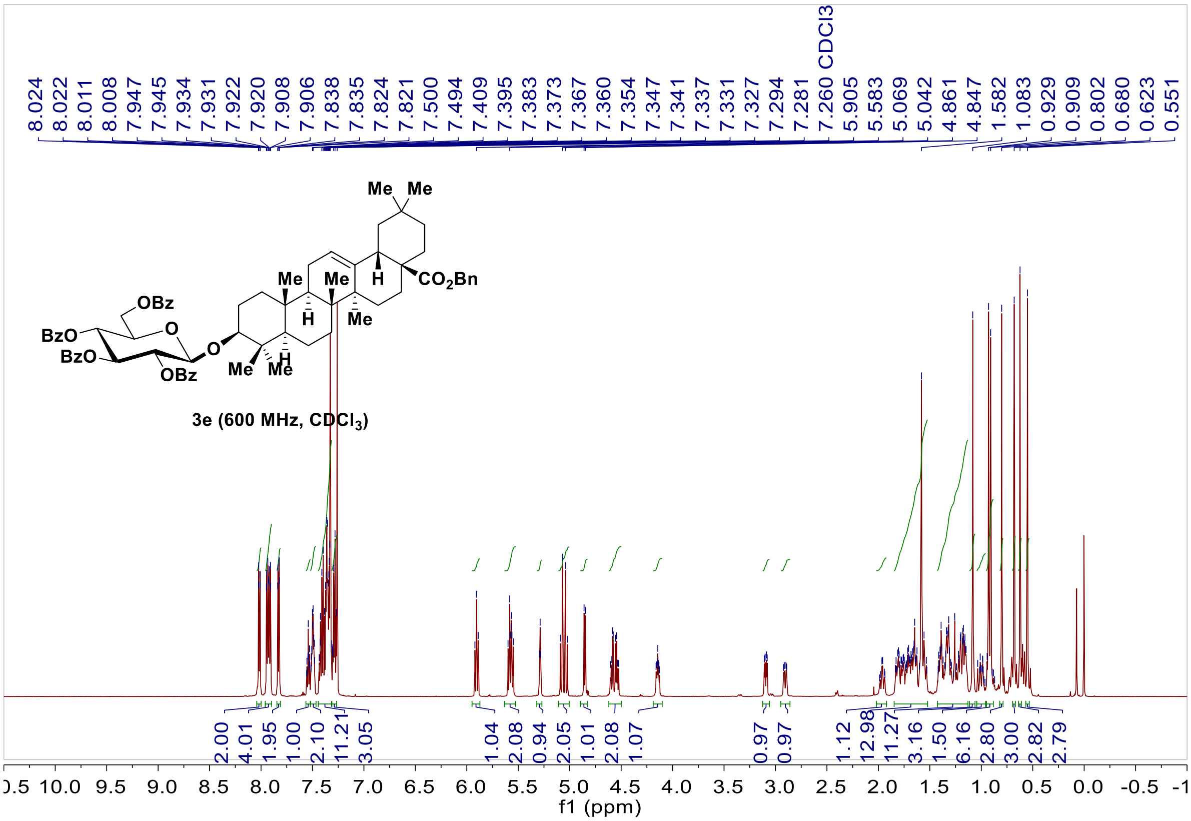
8.024
8.022
8.011
8.008
7.947
7.945
7.934
7.931
7.922
7.920
7.908
7.906
7.838
7.835
7.824
7.821
7.500
7.494
7.409
7.395
7.383
7.373
7.367
7.360
7.354
7.347
7.341
7.337
7.331
7.327
7.294
7.281
7.260 CDCl3
5.905
5.583
5.069
5.042
4.861
4.847
1.582
1.083
0.929
0.909
0.802
0.680
0.623
0.551
Me
Me
Me
Me
H
CO2Bn
H
Me
OBz
BzO
BzO
OBz
O
O
Me
Me
H
3e (600 MHz, CDCl3)
2.00
4.01
1.95
1.00
2.10
11.21
3.05
1.04
2.08
0.94
2.05
1.01
2.08
1.07
0.97
0.97
1.12
12.98
11.27
3.16
1.50
6.16
2.80
3.00
2.82
2.79
10.0 9.5 9.0 8.5 8.0 7.5 7.0 6.5 6.0 5.5 5.0 4.5 4.0 3.5 3.0 2.5 2.0 1.5 1.0 0.5 0.0 -0.5
f1 (ppm)

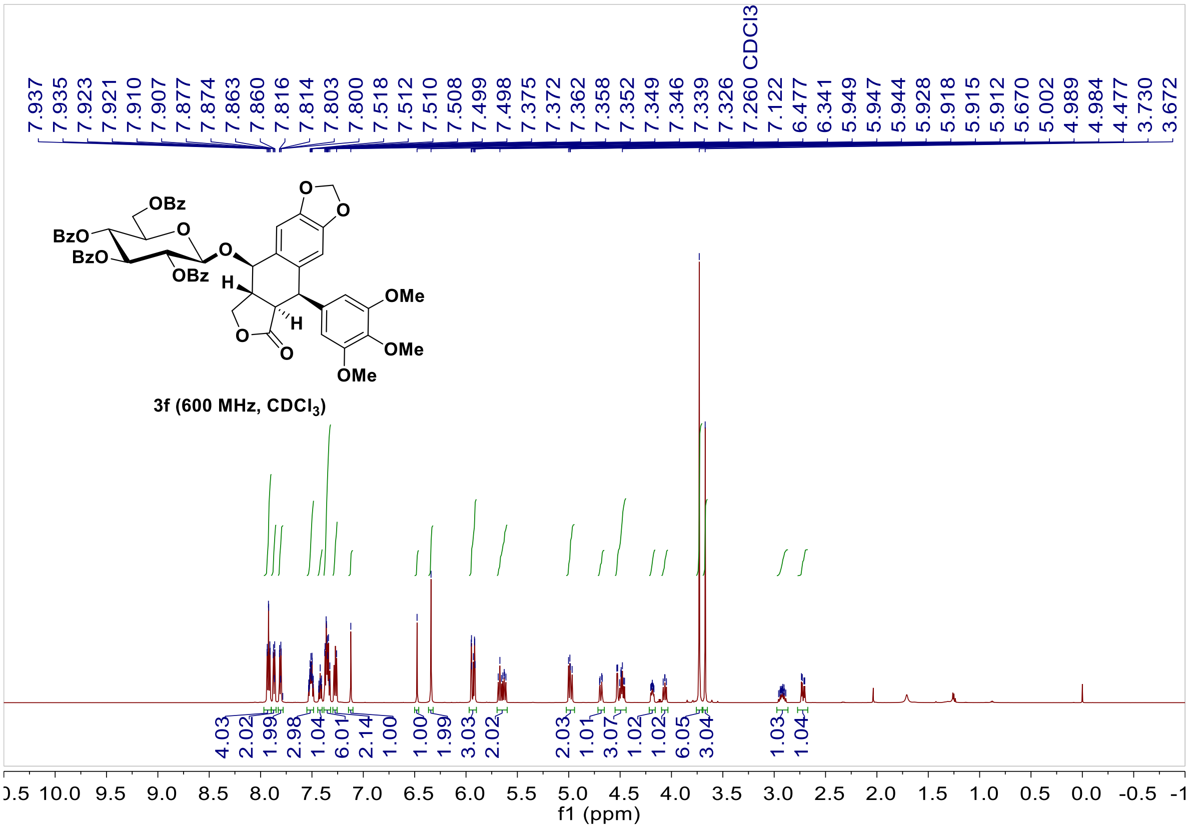
3f (600 MHz, CDCl3)
f1 (ppm)

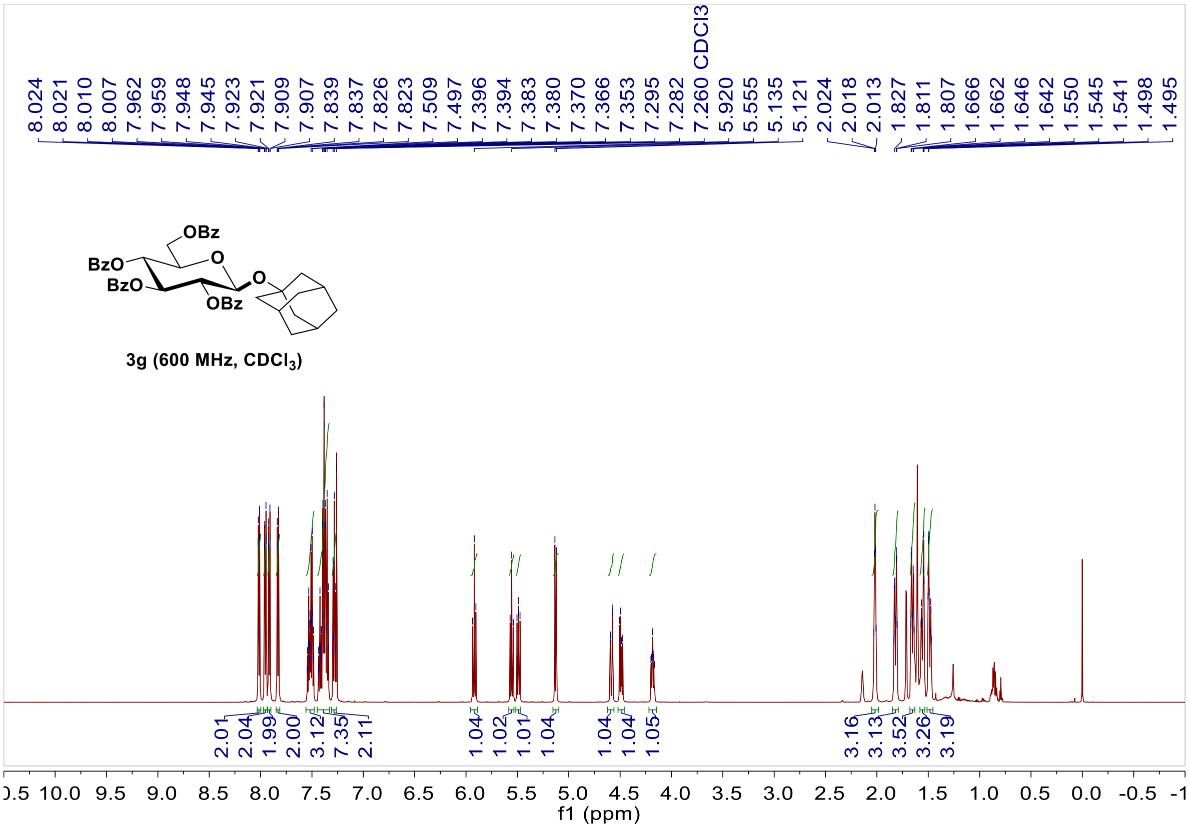
3g (600 MHz, CDCl3)
f1 (ppm)

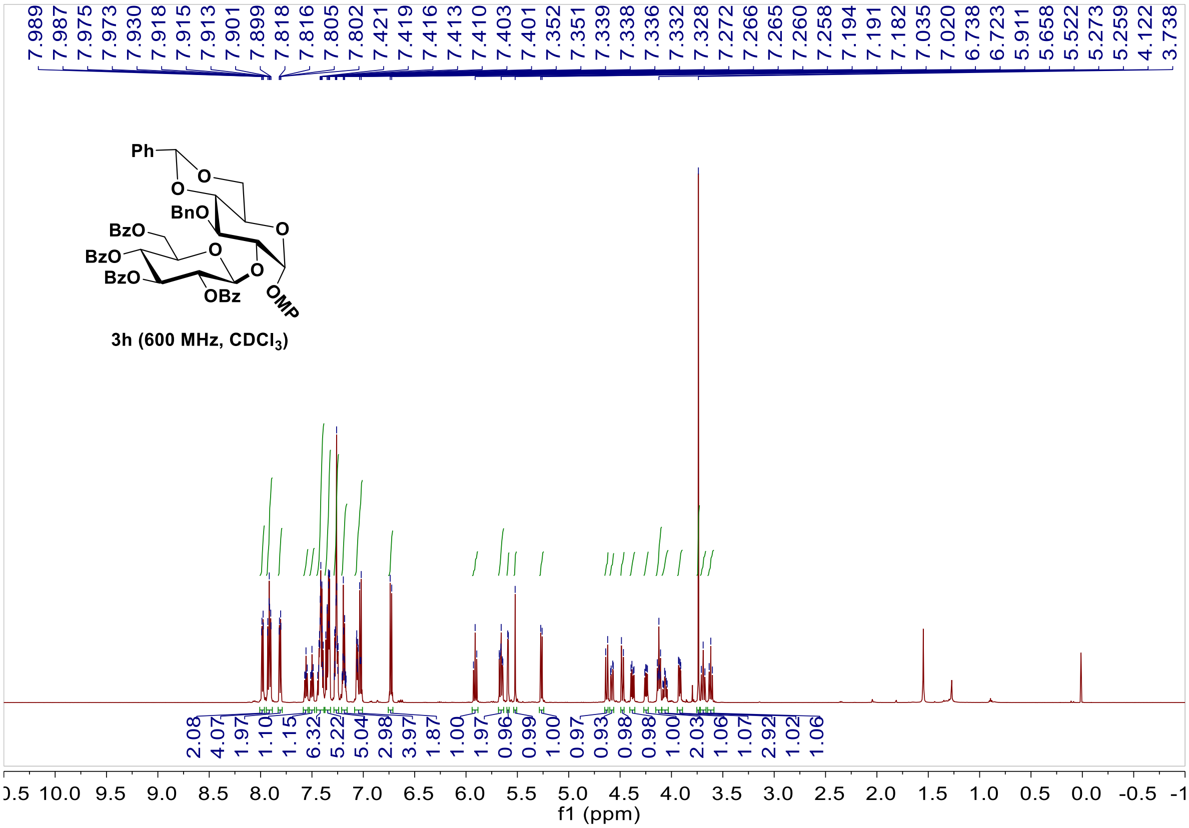
7.989 7.987 7.975 7.973 7.930 7.918 7.915 7.913 7.901 7.899 7.818 7.816 7.805 7.802 7.421 7.419 7.416 7.413 7.410 7.403 7.401 7.352 7.351 7.339 7.338 7.336 7.332 7.328 7.272 7.266 7.265 7.260 7.258 7.194 7.191 7.182 7.035 7.020 6.738 6.723 5.911 5.658 5.522 5.273 5.259 4.122 3.738
Ph
O
O
BnO
O
BzO
BzO
BzO
O
O
OBz
OMP
3h (600 MHz, CDCl$_3$)
2.08 4.07 1.97 1.10 1.15 6.32 5.22 5.04 2.98 3.97 1.87 1.00 1.97 0.96 0.99 1.00 0.97 0.93 0.98 0.98 1.00 2.03 1.06 1.07 2.92 1.02 1.06
f1 (ppm)

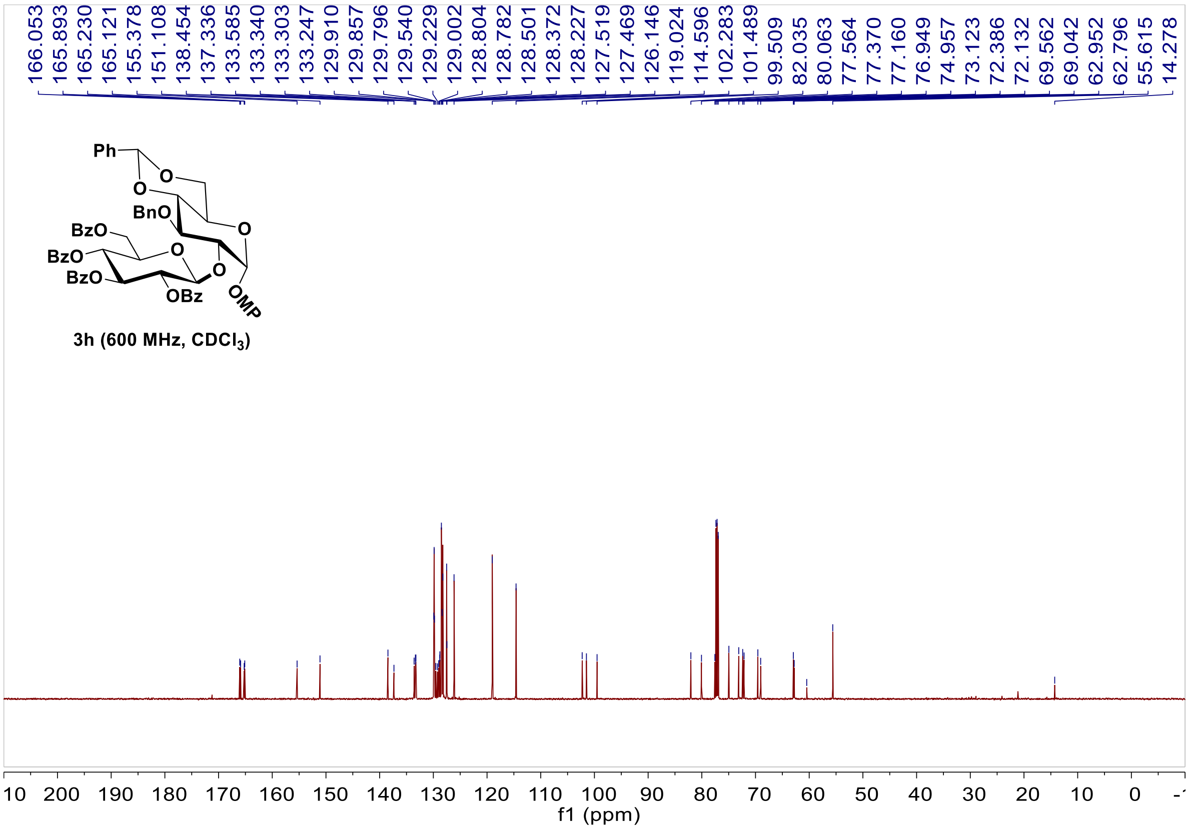
166.053 165.893 165.230 165.121 155.378 151.108 138.454 137.336 133.585 133.340 133.303 133.247 129.910 129.857 129.796 129.540 129.229 129.002 128.804 128.782 128.501 128.372 128.227 127.519 127.469 126.146 119.024 114.596 102.283 101.489 99.509 82.035 80.063 77.564 77.370 77.160 76.949 74.957 73.123 72.386 72.132 69.562 69.042 62.952 62.796 55.615 14.278
Ph
O
O
BnO
O
BzO
BzO
BzO
O
O
OBz
OMP
3h (600 MHz, CDCl$_3$)
f1 (ppm)

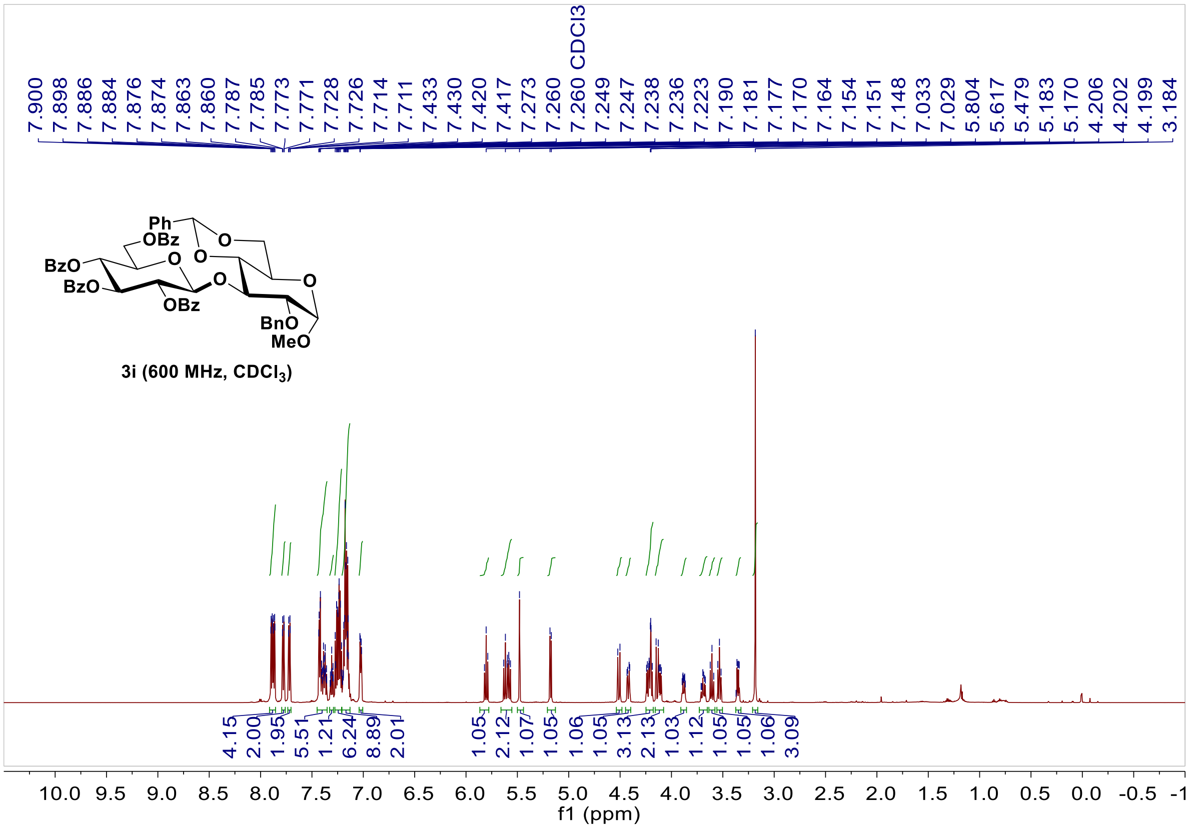
7.900 7.898 7.886 7.884 7.876 7.874 7.863 7.860 7.787 7.785 7.773 7.771 7.728 7.726 7.714 7.711 7.433 7.430 7.420 7.417 7.273 7.260 7.260 CDCl3 7.249 7.247 7.238 7.236 7.223 7.190 7.181 7.177 7.170 7.164 7.154 7.151 7.148 7.033 7.029 5.804 5.617 5.479 5.183 5.170 4.206 4.202 4.199 3.184
Ph
OBz
BzO
BzO
OBz
BnO
MeO
3i (600 MHz, CDCl$_3$)
4.15 2.00 1.95 5.51 1.21 6.24 8.89 2.01 1.05 2.12 1.07 1.05 1.06 1.05 3.13 2.13 1.03 1.12 1.05 1.05 1.06 3.09
10.0 9.5 9.0 8.5 8.0 7.5 7.0 6.5 6.0 5.5 5.0 4.5 4.0 3.5 3.0 2.5 2.0 1.5 1.0 0.5 0.0 -0.5 -1
f1 (ppm)

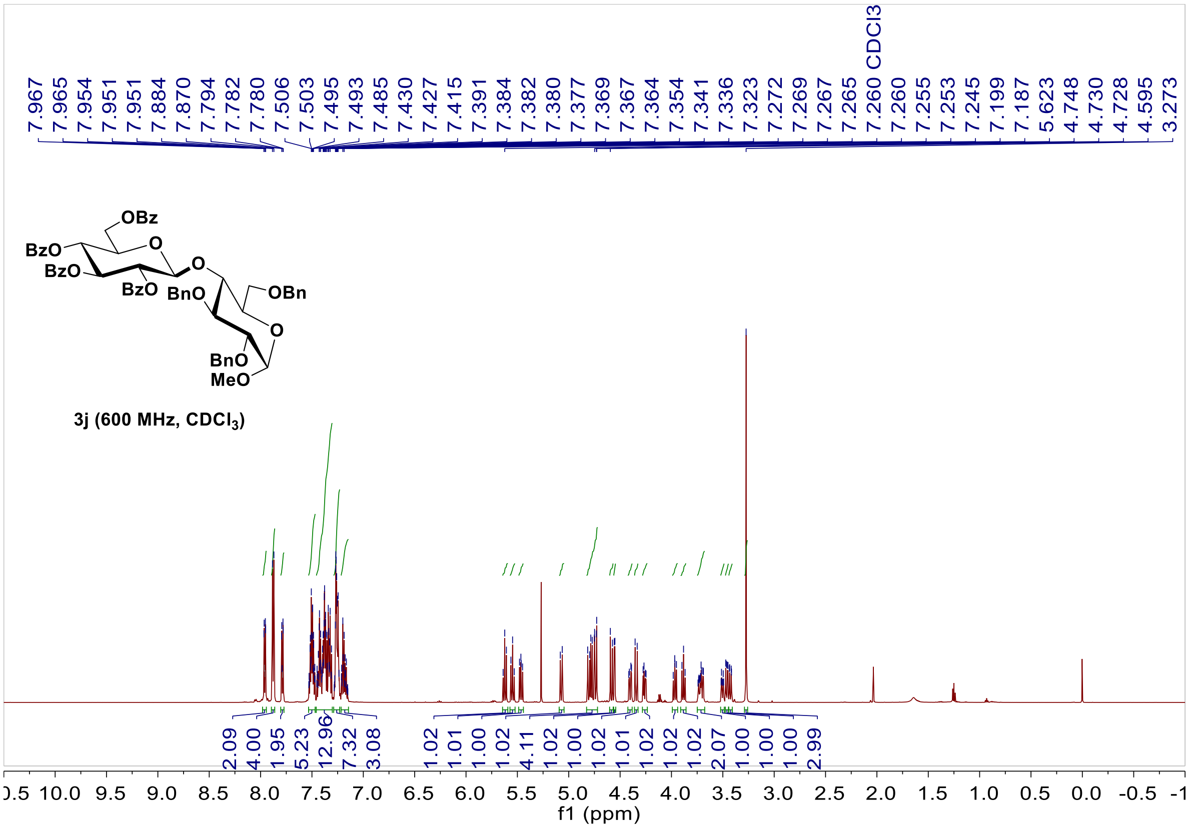
7.967 7.965 7.954 7.951 7.884 7.870 7.794 7.782 7.780 7.506 7.503 7.495 7.493 7.485 7.430 7.427 7.415 7.391 7.384 7.382 7.380 7.377 7.369 7.367 7.364 7.354 7.341 7.336 7.323 7.272 7.269 7.267 7.265 7.260 CDCl3 7.260 7.255 7.253 7.245 7.199 7.187 5.623 4.748 4.730 4.728 4.595 3.273
OBz
BzO
BzO
BzO
BnO
OBn
BnO
MeO
3j (600 MHz, CDCl$_3$)
2.09 4.00 1.95 5.23 12.96 7.32 3.08 1.02 1.01 1.00 1.02 4.11 1.02 1.00 1.02 1.01 1.02 1.02 1.02 2.07 1.00 1.00 1.00 2.99
10.0 9.5 9.0 8.5 8.0 7.5 7.0 6.5 6.0 5.5 5.0 4.5 4.0 3.5 3.0 2.5 2.0 1.5 1.0 0.5 0.0 -0.5 -1
f1 (ppm)

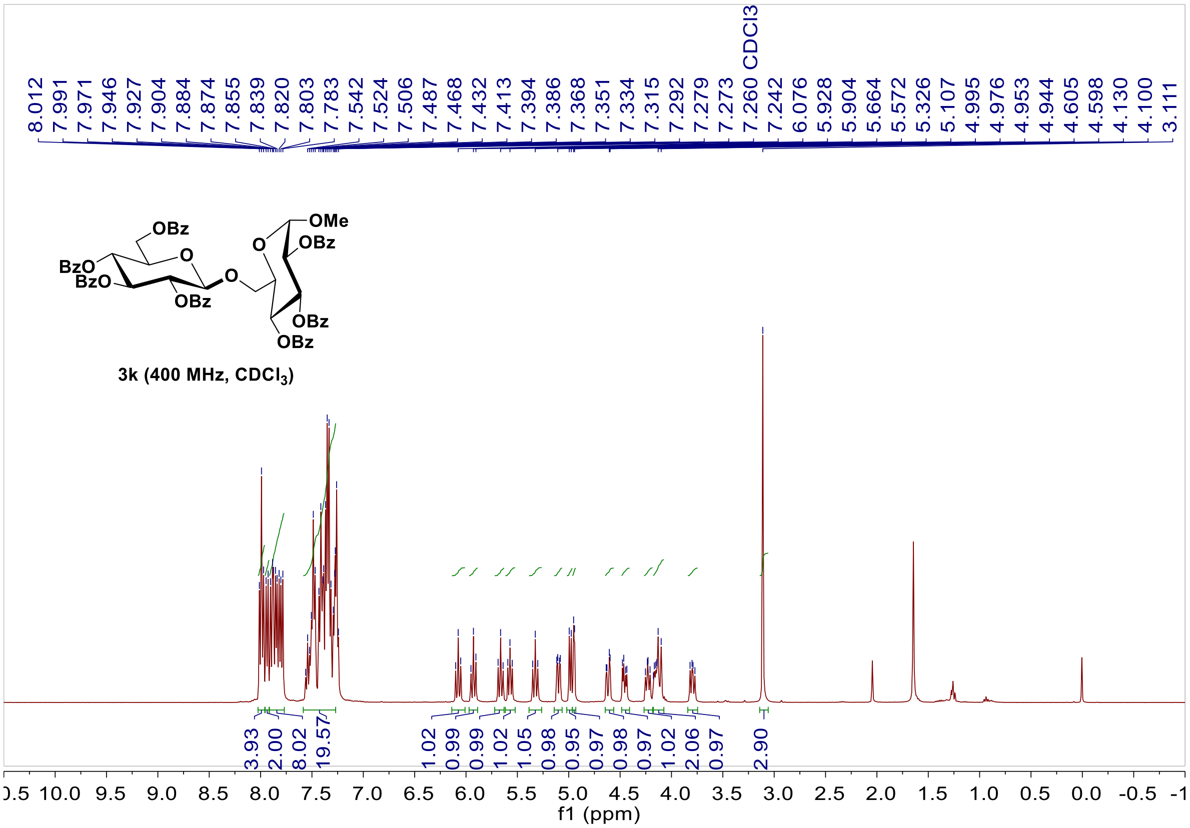
8.012 7.991 7.971 7.946 7.927 7.904 7.884 7.874 7.855 7.839 7.820 7.803 7.783 7.542 7.524 7.506 7.487 7.468 7.432 7.413 7.394 7.386 7.368 7.351 7.334 7.315 7.292 7.279 7.273 7.260 CDCl3 7.242 6.076 5.928 5.904 5.664 5.572 5.326 5.107 4.995 4.976 4.953 4.944 4.605 4.598 4.130 4.100 3.111
OBz
OMe
OBz
BzO
BzO
OBz
OBz
OBz
3k (400 MHz, $CDCl_3$)
3.93 2.00 8.02 19.57 1.02 0.99 0.99 1.02 1.05 0.98 0.95 0.97 0.98 0.97 1.02 2.06 0.97 2.90
f1 (ppm)

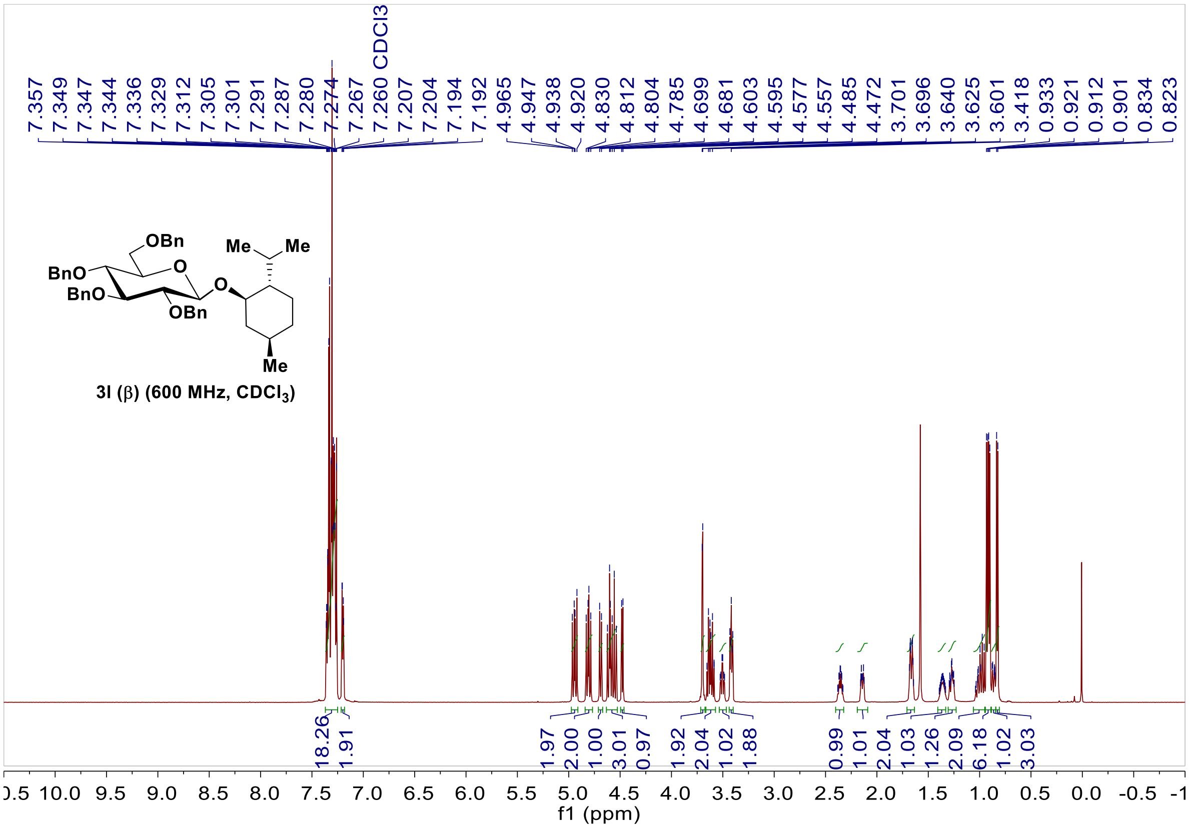
7.357 7.349 7.347 7.344 7.336 7.329 7.312 7.305 7.301 7.291 7.287 7.280 7.274 7.267 7.260 CDCl3 7.207 7.204 7.194 7.192 4.965 4.947 4.938 4.920 4.830 4.812 4.804 4.785 4.699 4.681 4.603 4.595 4.577 4.557 4.485 4.472 3.701 3.696 3.640 3.625 3.601 3.418 0.933 0.921 0.912 0.901 0.834 0.823
OBn
Me
Me
BnO
BnO
OBn
Me
3l (β) (600 MHz, $CDCl_3$)
18.26 1.91 1.97 2.00 1.00 3.01 0.97 1.92 2.04 1.02 1.88 0.99 1.01 2.04 1.03 1.26 2.09 6.18 1.02 3.03
f1 (ppm)

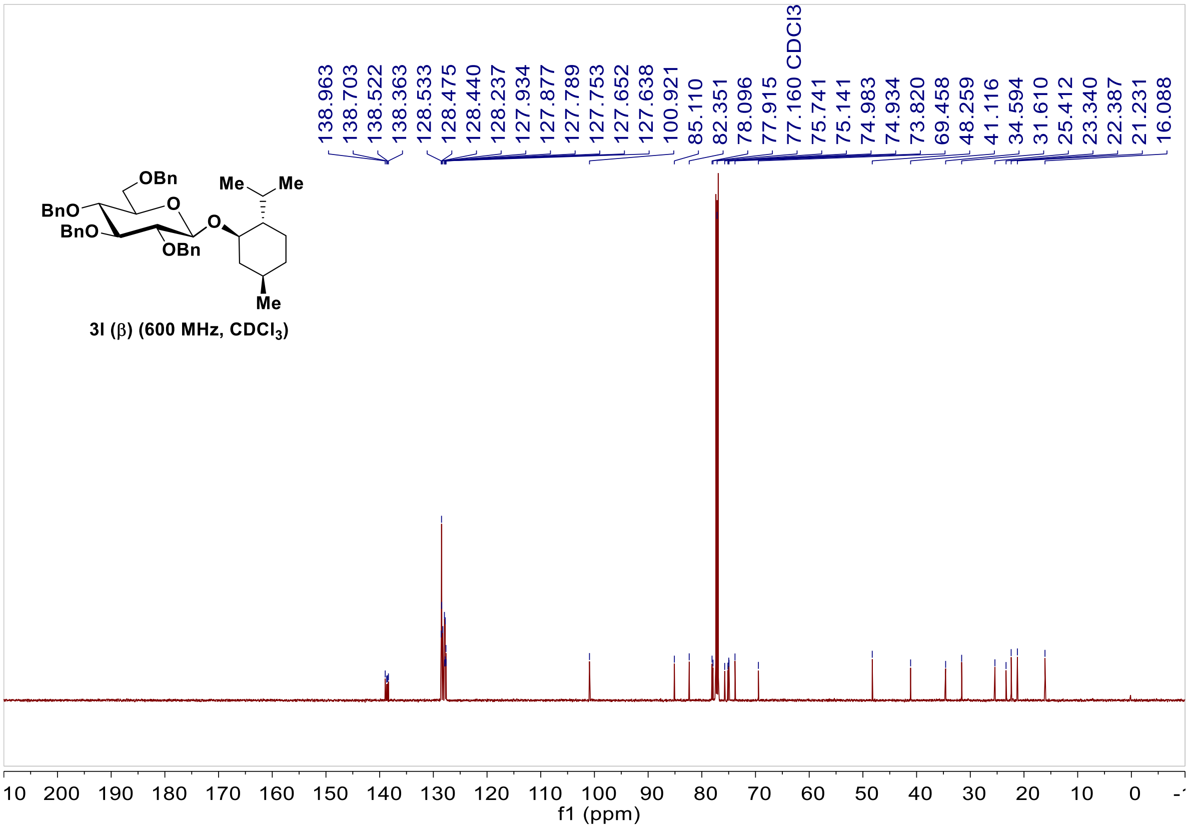
138.963
138.703
138.522
138.363
128.533
128.475
128.440
128.237
127.934
127.877
127.789
127.753
127.652
127.638
100.921
85.110
82.351
78.096
77.915
77.160 CDCl3
75.741
75.141
74.983
74.934
73.820
69.458
48.259
41.116
34.594
31.610
25.412
23.340
22.387
21.231
16.088
OBn
BnO
BnO
O
OBn
O
Me
Me
Me
3l (β) (600 MHz, CDCl3)
f1 (ppm)

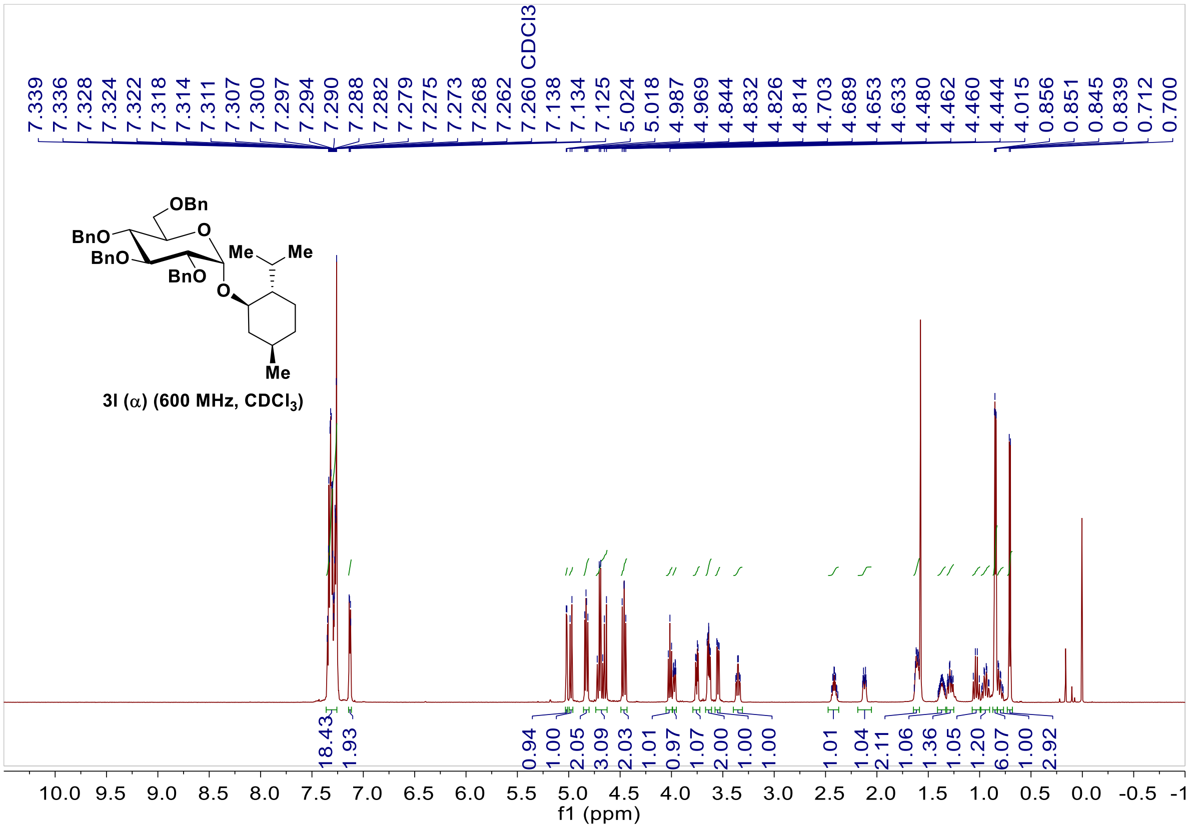
7.339
7.336
7.328
7.324
7.322
7.318
7.314
7.311
7.307
7.300
7.297
7.294
7.290
7.288
7.282
7.279
7.275
7.273
7.268
7.262
7.260 CDCl3
7.138
7.134
7.125
5.024
5.018
4.987
4.969
4.844
4.832
4.826
4.814
4.703
4.689
4.653
4.633
4.480
4.462
4.460
4.444
4.015
0.856
0.851
0.845
0.839
0.712
0.700
OBn
BnO
BnO
BnO
O
O
Me
Me
Me
3l (α) (600 MHz, CDCl3)
18.43
1.93
0.94
1.00
2.05
3.09
2.03
1.01
0.97
1.07
2.00
1.00
1.00
1.01
1.04
2.11
1.06
1.36
1.05
1.20
6.07
1.00
2.92
f1 (ppm)

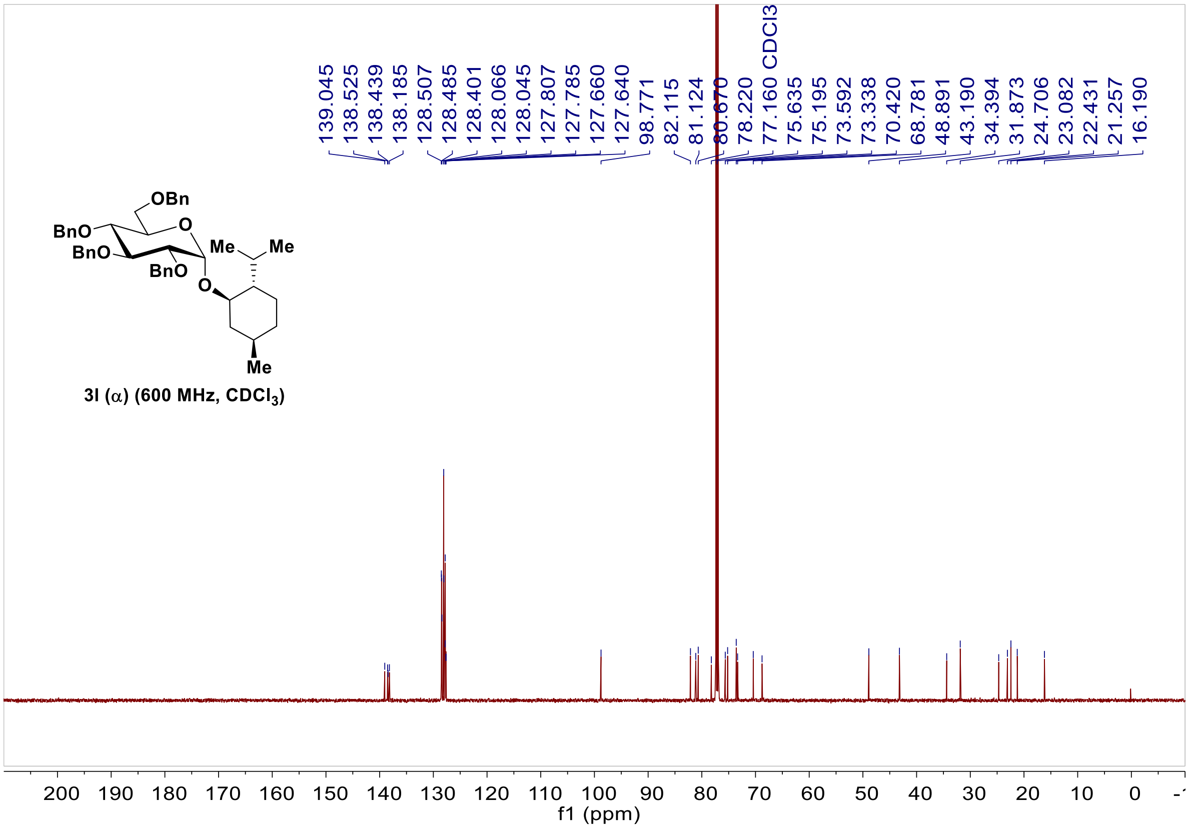

3l (α) (600 MHz, $CDCl_3$)

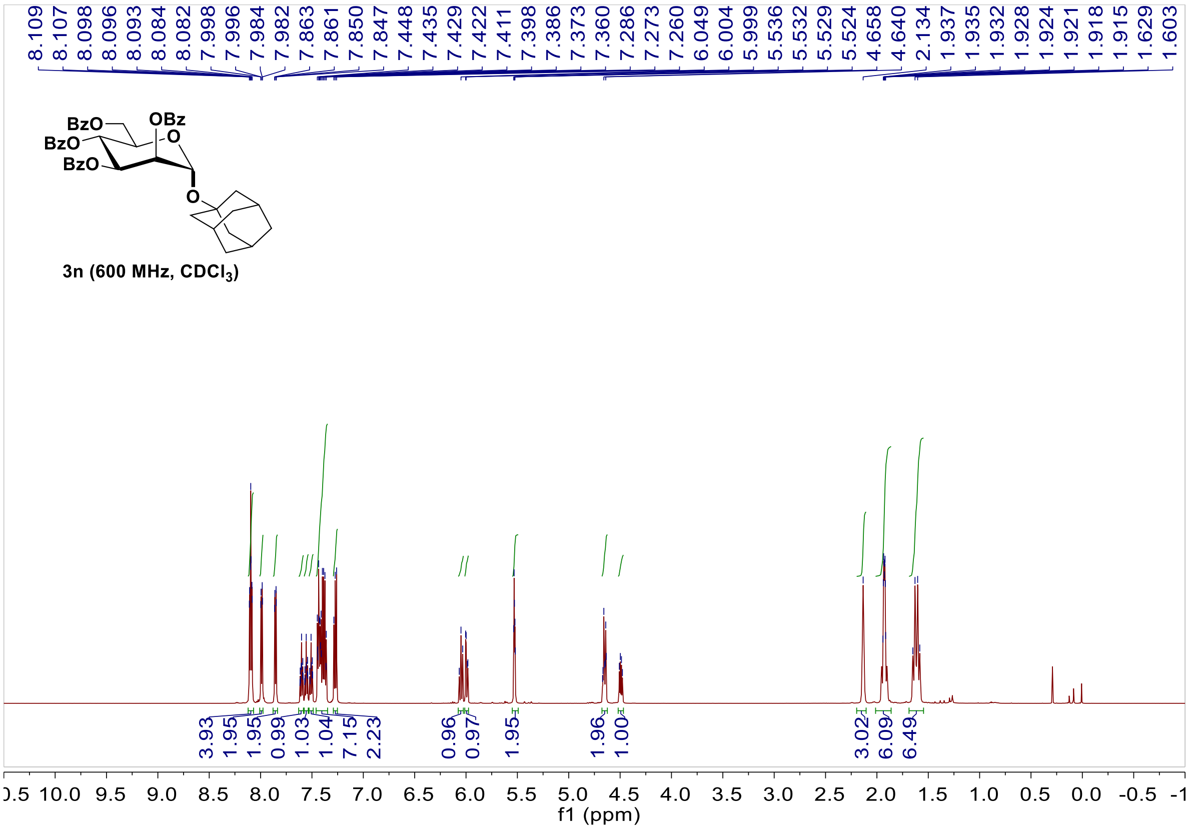

3n (600 MHz, $CDCl_3$)

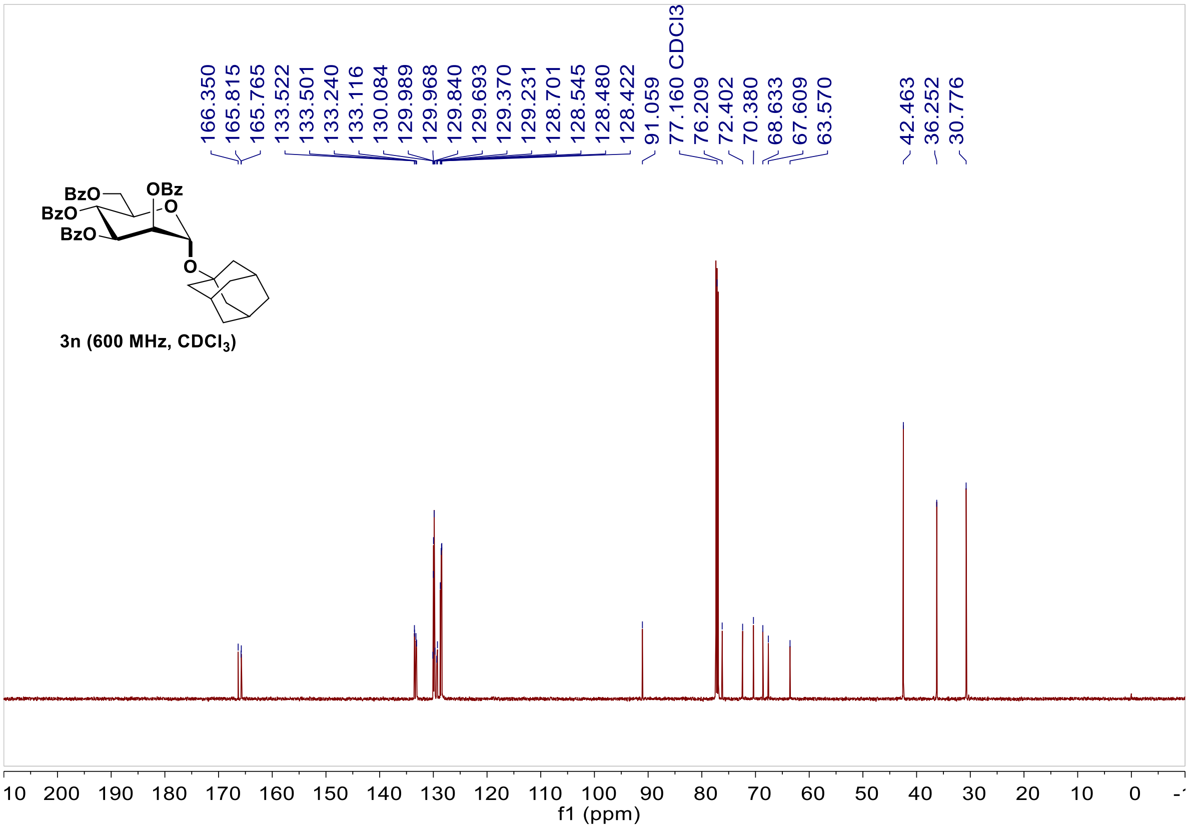
166.350
165.815
165.765
133.522
133.501
133.240
133.116
130.084
129.989
129.968
129.840
129.693
129.370
129.231
128.701
128.545
128.480
128.422
91.059
77.160 CDCl3
76.209
72.402
70.380
68.633
67.609
63.570
42.463
36.252
30.776
BzO
OBz
BzO
O
BzO
O
3n (600 MHz, CDCl3)
f1 (ppm)

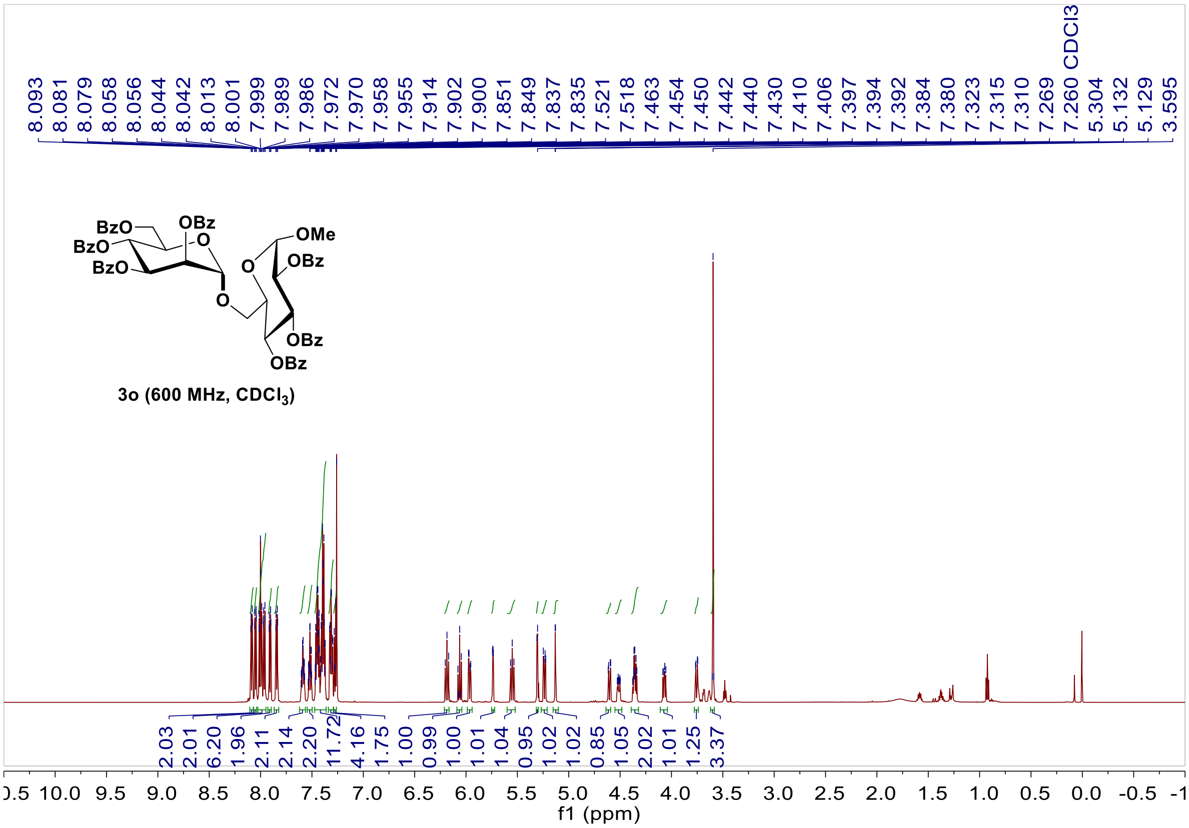
8.093
8.081
8.079
8.058
8.056
8.044
8.042
8.013
8.001
7.999
7.989
7.986
7.972
7.970
7.958
7.955
7.914
7.902
7.900
7.851
7.849
7.837
7.835
7.521
7.518
7.463
7.454
7.450
7.442
7.440
7.430
7.410
7.406
7.397
7.394
7.392
7.384
7.380
7.323
7.315
7.310
7.269
7.260 CDCl3
5.304
5.132
5.129
3.595
BzO
OBz
BzO
O
BzO
O
OMe
OBz
O
OBz
OBz
3o (600 MHz, CDCl3)
2.03
2.01
6.20
1.96
2.11
2.14
2.20
11.72
4.16
1.75
1.00
0.99
1.00
1.01
1.04
0.95
1.02
1.02
0.85
1.05
2.02
1.01
1.25
3.37
f1 (ppm)

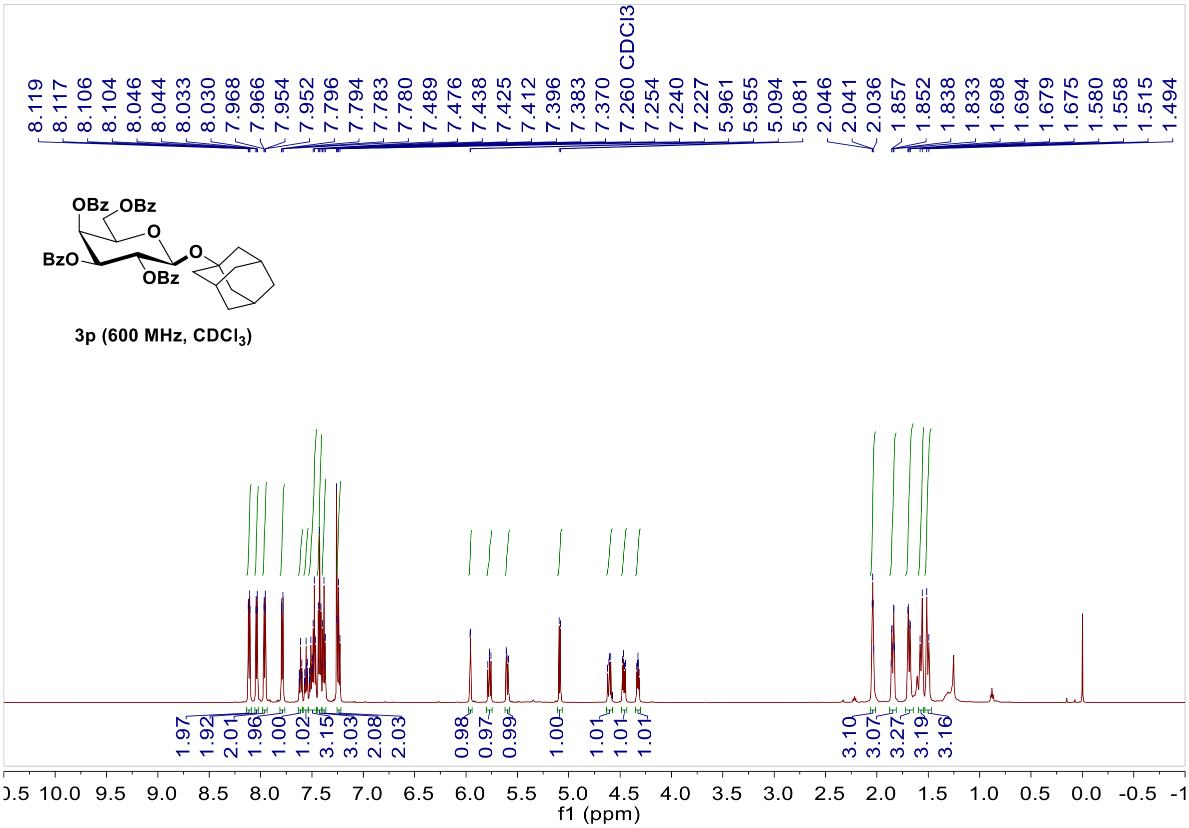
8.119 8.117 8.106 8.104 8.046 8.044 8.033 8.030 7.968 7.966 7.954 7.952 7.796 7.794 7.783 7.780 7.489 7.476 7.438 7.425 7.412 7.396 7.383 7.370 7.260 CDCl3 7.254 7.240 7.227 5.961 5.955 5.094 5.081 2.046 2.041 2.036 1.857 1.852 1.838 1.833 1.698 1.694 1.679 1.675 1.580 1.558 1.515 1.494
OBz OBz
O
BzO
OBz
O
3p (600 MHz, CDCl3)
1.97 1.92 2.01 1.96 1.00 1.02 3.15 3.03 2.08 2.03 0.98 0.97 0.99 1.00 1.01 1.01 1.01 3.10 3.07 3.27 3.19 3.16
f1 (ppm)

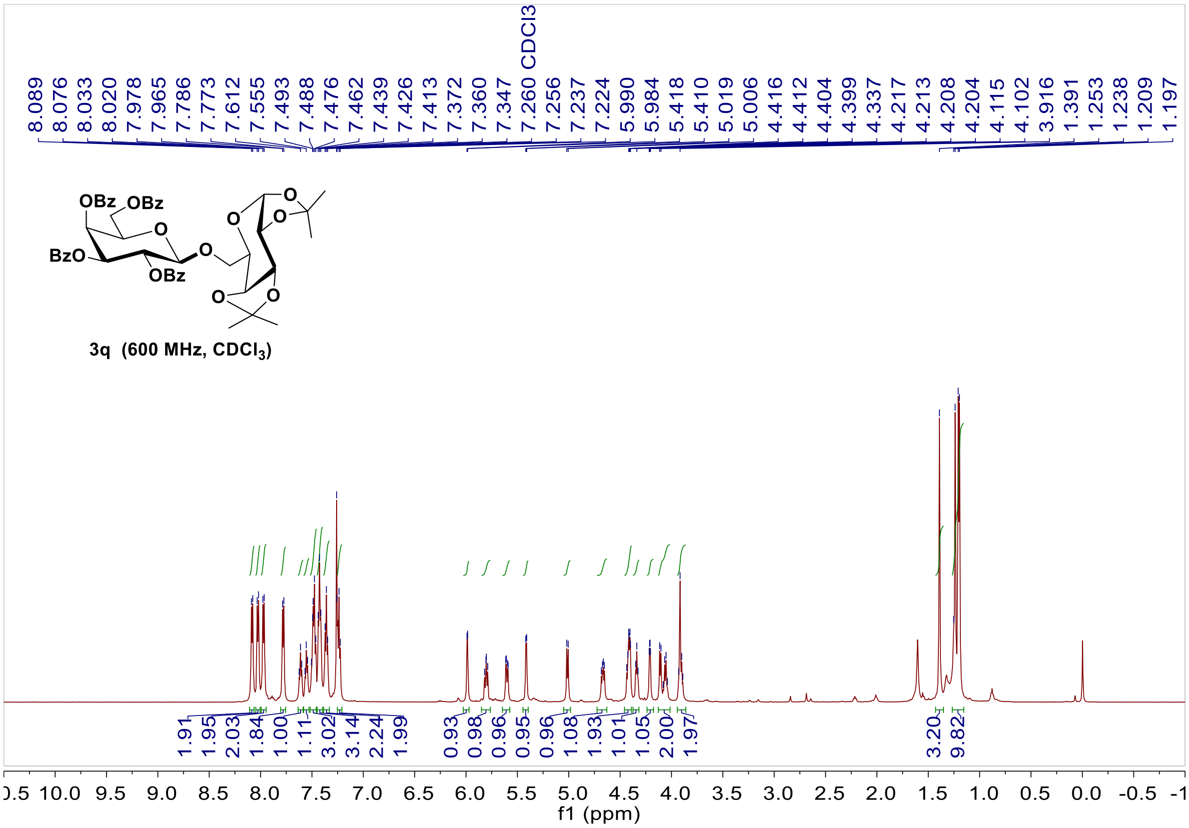
8.089 8.076 8.033 8.020 7.978 7.965 7.786 7.773 7.612 7.555 7.493 7.488 7.476 7.462 7.439 7.426 7.413 7.372 7.360 7.347 7.260 CDCl3 7.256 7.237 7.224 5.990 5.984 5.418 5.410 5.019 5.006 4.416 4.412 4.404 4.399 4.337 4.217 4.213 4.208 4.204 4.115 4.102 3.916 1.391 1.253 1.238 1.209 1.197
OBz OBz
O
BzO
OBz
3q (600 MHz, CDCl3)
1.91 1.95 2.03 1.84 1.00 1.11 3.02 3.14 2.24 1.99 0.93 0.98 0.96 0.95 0.96 1.08 1.93 1.01 1.05 2.00 1.97 3.20 9.82
f1 (ppm)

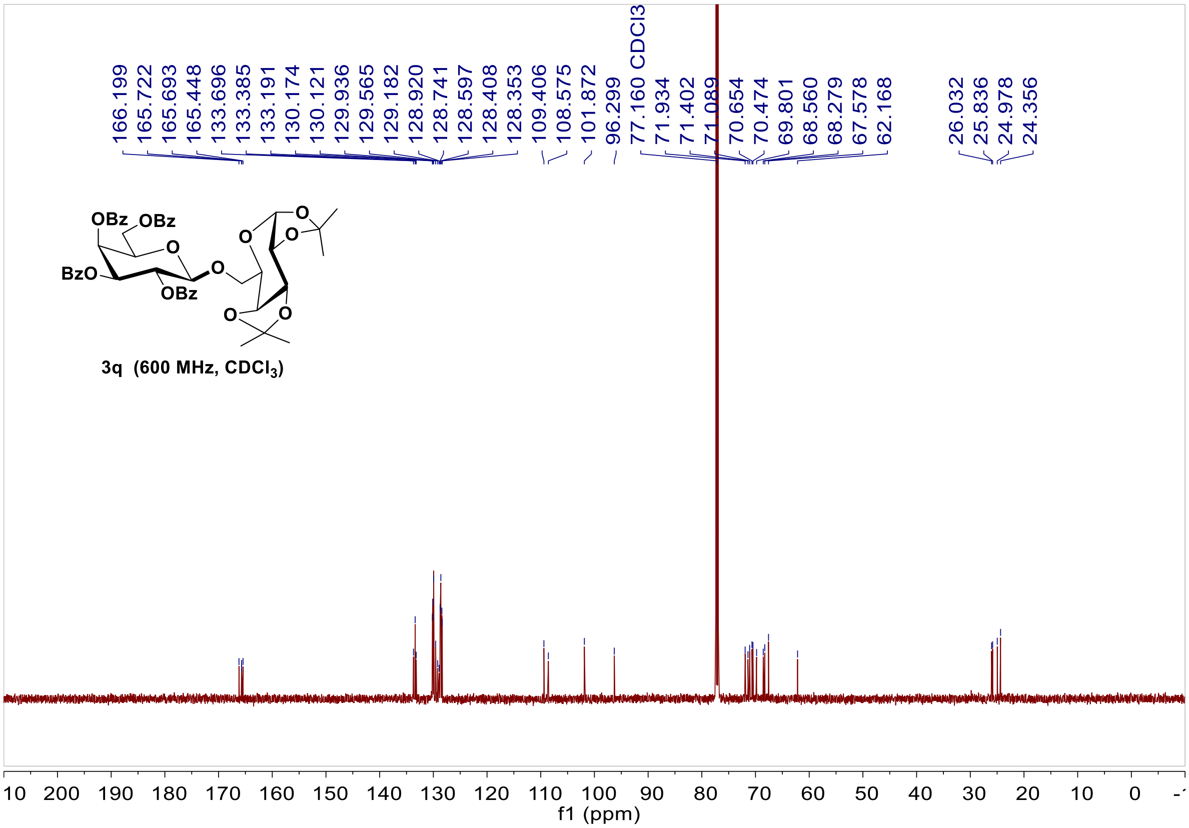
166.199
165.722
165.693
165.448
133.696
133.385
133.191
130.174
130.121
129.936
129.565
129.182
128.920
128.741
128.597
128.408
128.353
109.406
108.575
101.872
96.299
77.160 CDCl3
71.934
71.402
71.089
70.654
70.474
69.801
68.560
68.279
67.578
62.168
26.032
25.836
24.978
24.356
3q (600 MHz, CDCl3)
f1 (ppm)

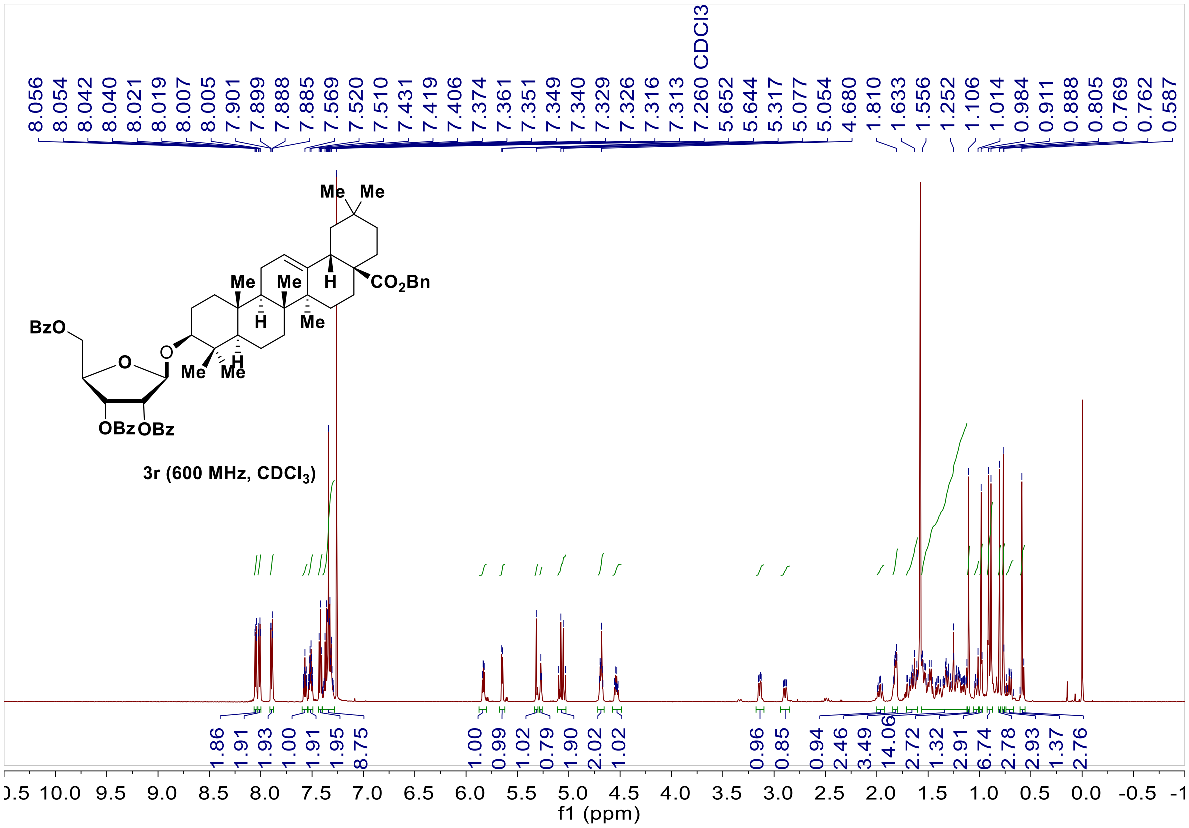
8.056
8.054
8.042
8.040
8.021
8.019
8.007
8.005
7.901
7.899
7.888
7.885
7.569
7.520
7.510
7.431
7.419
7.406
7.374
7.361
7.351
7.349
7.340
7.329
7.326
7.316
7.313
7.260 CDCl3
5.652
5.644
5.317
5.077
5.054
4.680
1.810
1.633
1.556
1.252
1.106
1.014
0.984
0.911
0.888
0.805
0.769
0.762
0.587
3r (600 MHz, CDCl3)
1.86
1.91
1.93
1.00
1.91
1.95
8.75
1.00
0.99
1.02
0.79
1.90
2.02
1.02
0.96
0.85
0.94
2.46
3.49
14.06
2.72
1.32
2.91
6.74
2.78
2.93
1.37
2.76
f1 (ppm)

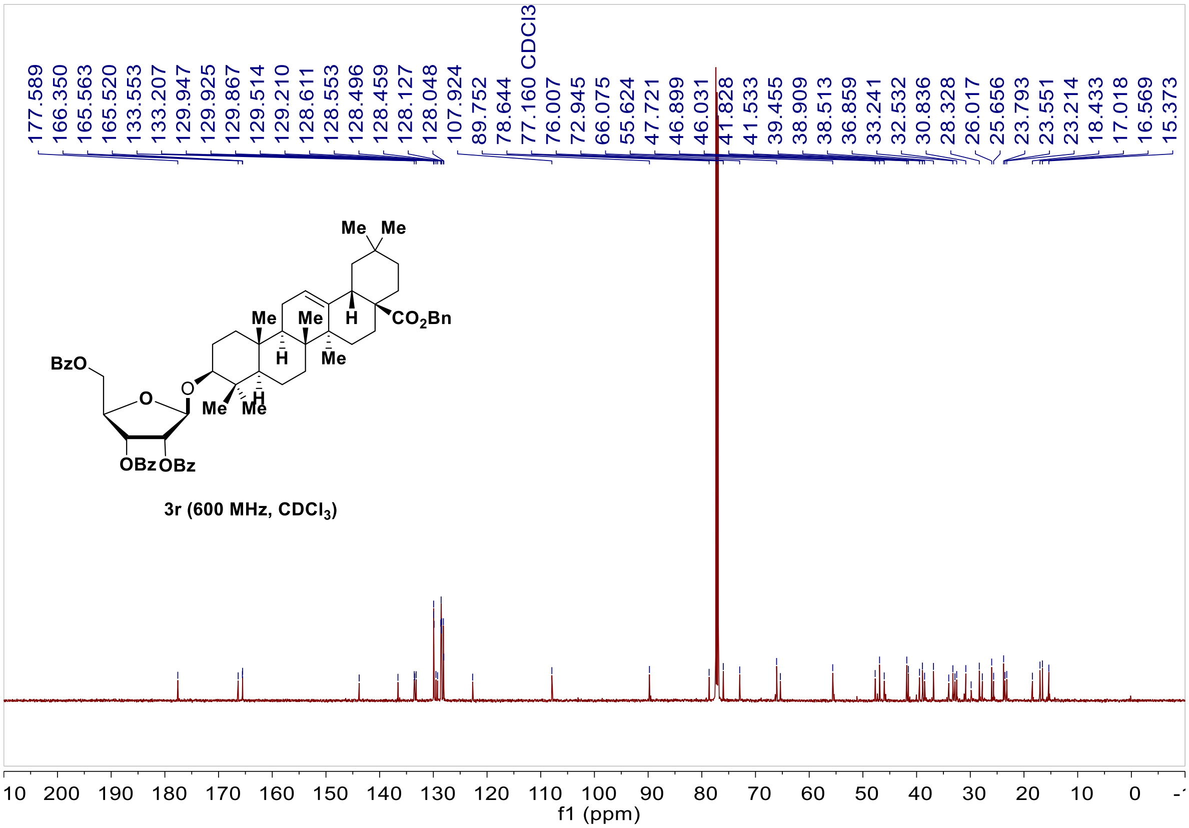
177.589 166.350 165.563 165.520 133.553 133.207 129.947 129.925 129.867 129.514 129.210 128.611 128.553 128.496 128.459 128.127 128.048 107.924 89.752 78.644 77.160 CDCl3 76.007 72.945 66.075 55.624 47.721 46.899 46.031 41.828 41.533 39.455 38.909 38.513 36.859 33.241 32.532 30.836 28.328 26.017 25.656 23.793 23.551 23.214 18.433 17.018 16.569 15.373
Me Me
Me
Me
H
CO2Bn
H
Me
BzO
O
O
Me
Me
H
OBz OBz
3r (600 MHz, CDCl3)
f1 (ppm)

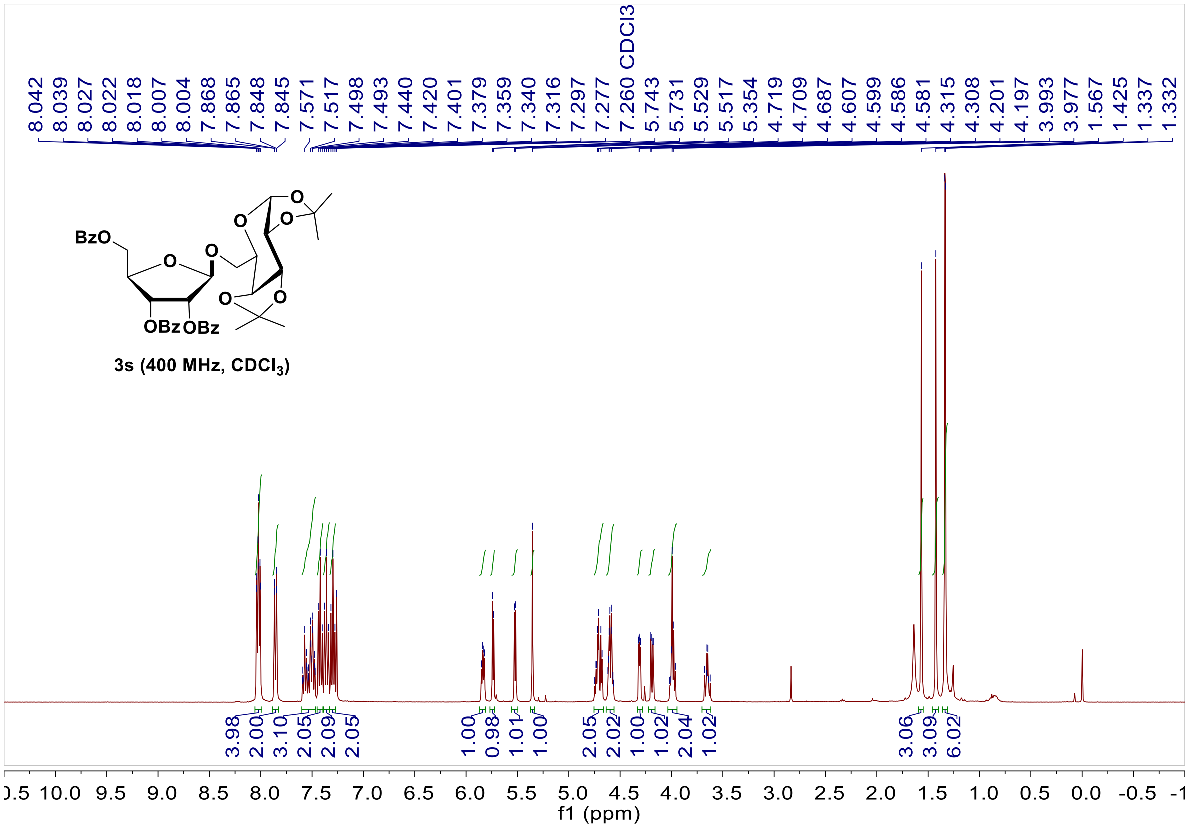
8.042 8.039 8.027 8.022 8.018 8.007 8.004 7.868 7.865 7.848 7.845 7.571 7.517 7.498 7.493 7.440 7.420 7.401 7.379 7.359 7.340 7.316 7.297 7.277 7.260 CDCl3 5.743 5.731 5.529 5.517 5.354 4.719 4.709 4.687 4.607 4.599 4.586 4.581 4.315 4.308 4.201 4.197 3.993 3.977 1.567 1.425 1.337 1.332
BzO
O
O
O
O
O
O
O
OBz OBz
3s (400 MHz, CDCl3)
3.98 2.00 3.10 2.05 2.09 2.05 1.00 0.98 1.01 1.00 2.05 2.02 1.00 1.02 2.04 1.02 3.06 3.09 6.02
f1 (ppm)

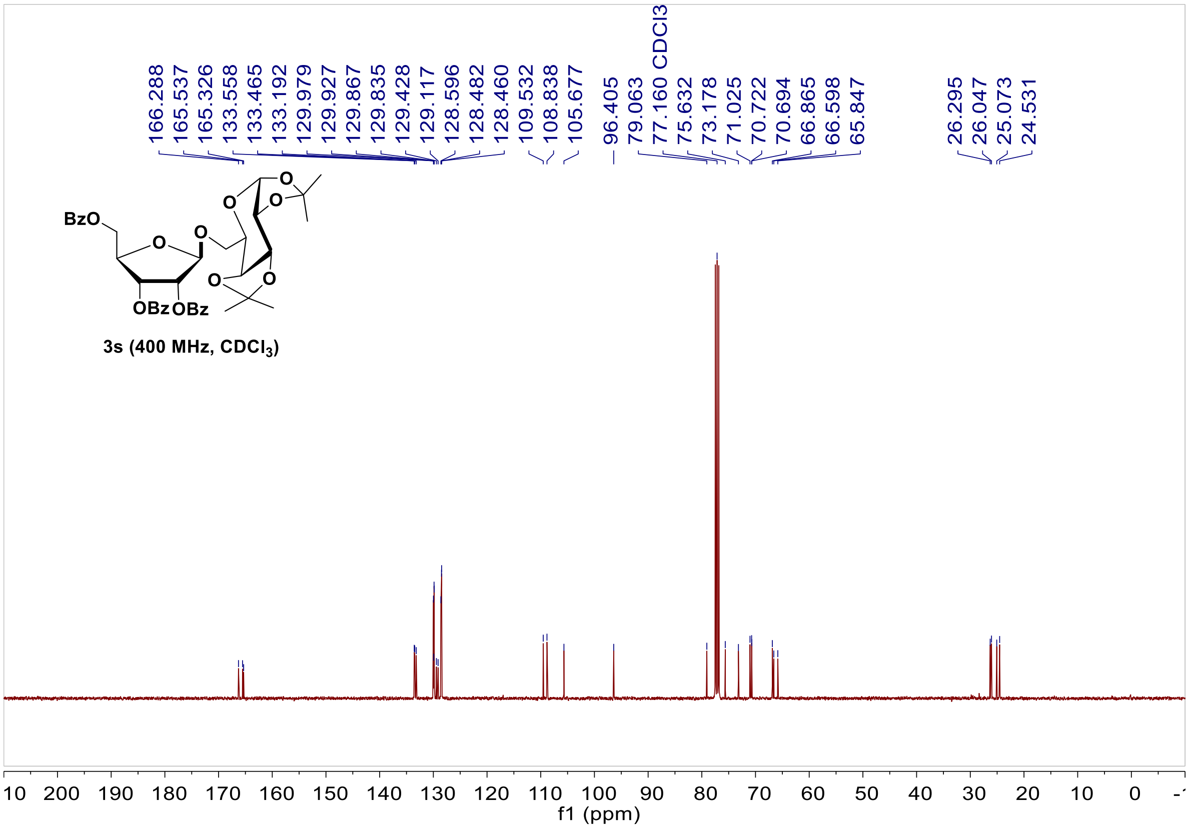

3s (400 MHz, $CDCl_3$)

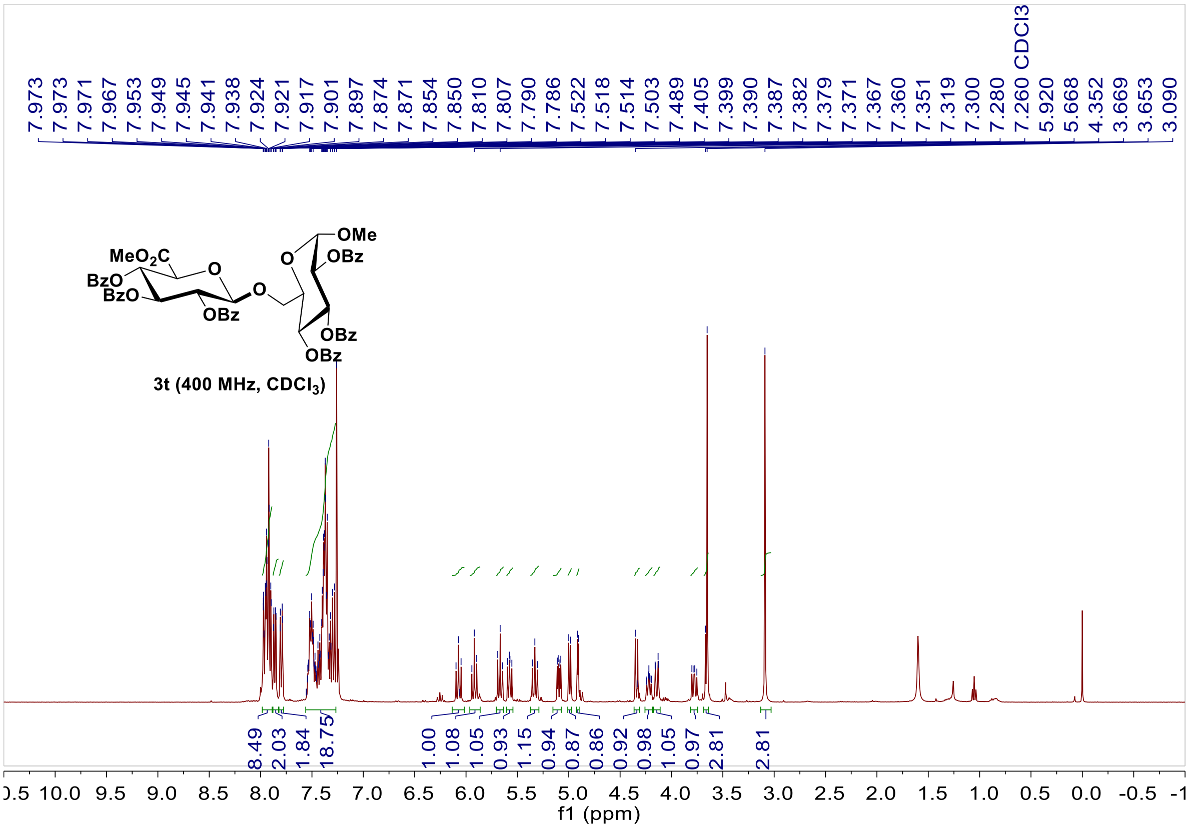

3t (400 MHz, $CDCl_3$)

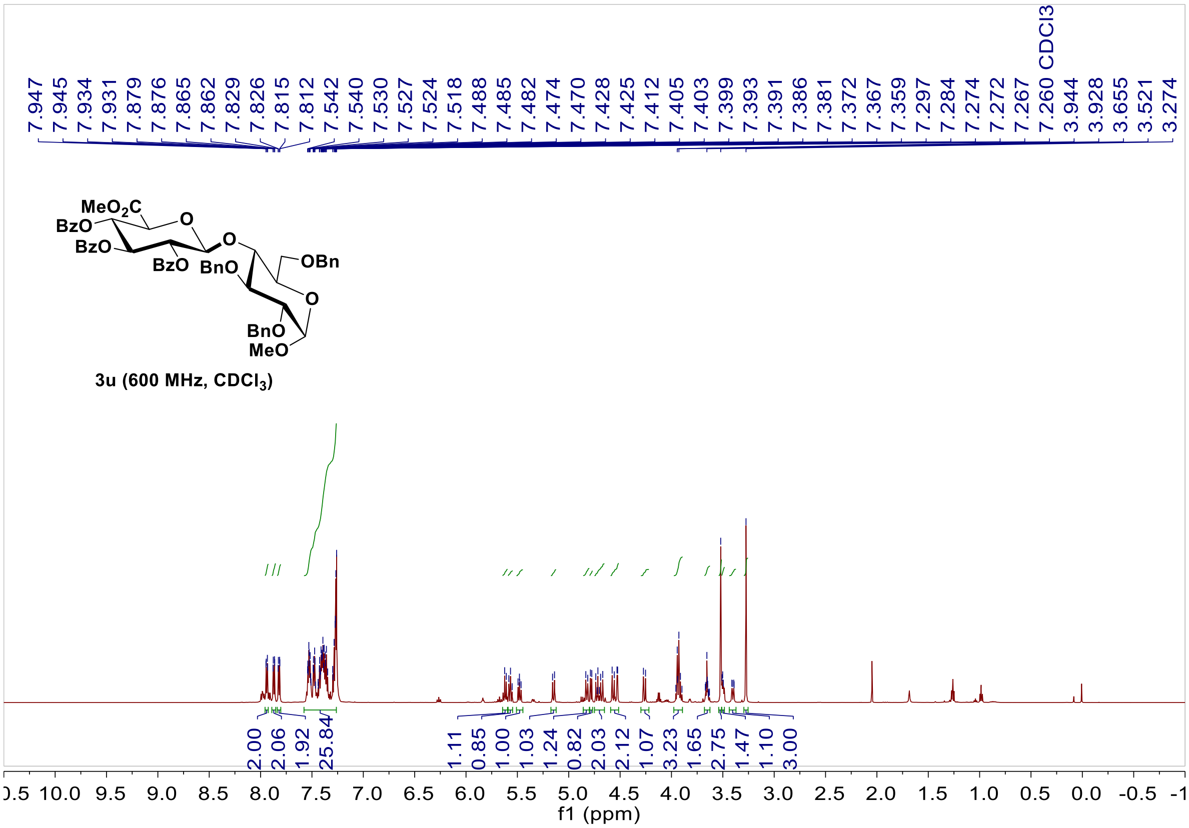
7.947 7.945 7.934 7.931 7.879 7.876 7.865 7.862 7.829 7.826 7.815 7.812 7.542 7.540 7.530 7.527 7.524 7.518 7.488 7.485 7.482 7.474 7.470 7.428 7.425 7.412 7.405 7.403 7.399 7.393 7.391 7.386 7.381 7.372 7.367 7.359 7.297 7.284 7.274 7.272 7.267 7.260 CDCl3 3.944 3.928 3.655 3.521 3.274
MeO2C
BzO
BzO
BzO
BnO
OBn
BnO
MeO
3u (600 MHz, CDCl3)
2.00 2.06 1.92 25.84 1.11 0.85 1.00 1.03 1.24 0.82 2.03 2.12 1.07 3.23 1.65 2.75 1.47 1.10 3.00
f1 (ppm)

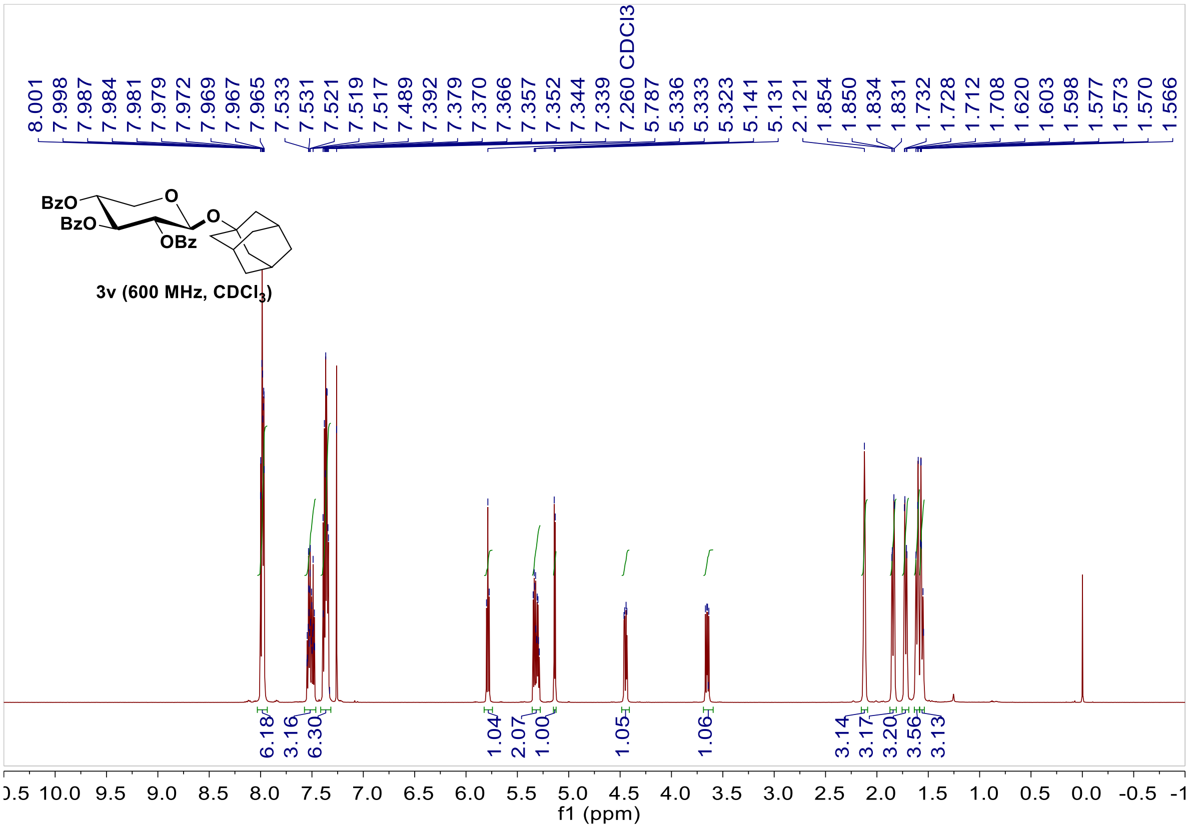
8.001 7.998 7.987 7.984 7.981 7.979 7.972 7.969 7.967 7.965 7.533 7.531 7.521 7.519 7.517 7.489 7.392 7.379 7.370 7.366 7.357 7.352 7.344 7.339 7.260 CDCl3 5.787 5.336 5.333 5.323 5.141 5.131 2.121 1.854 1.850 1.834 1.831 1.732 1.728 1.712 1.708 1.620 1.603 1.598 1.577 1.573 1.570 1.566
BzO
BzO
OBz
3v (600 MHz, CDCl3)
6.18 3.16 6.30 1.04 2.07 1.00 1.05 1.06 3.14 3.17 3.20 3.56 3.13
f1 (ppm)

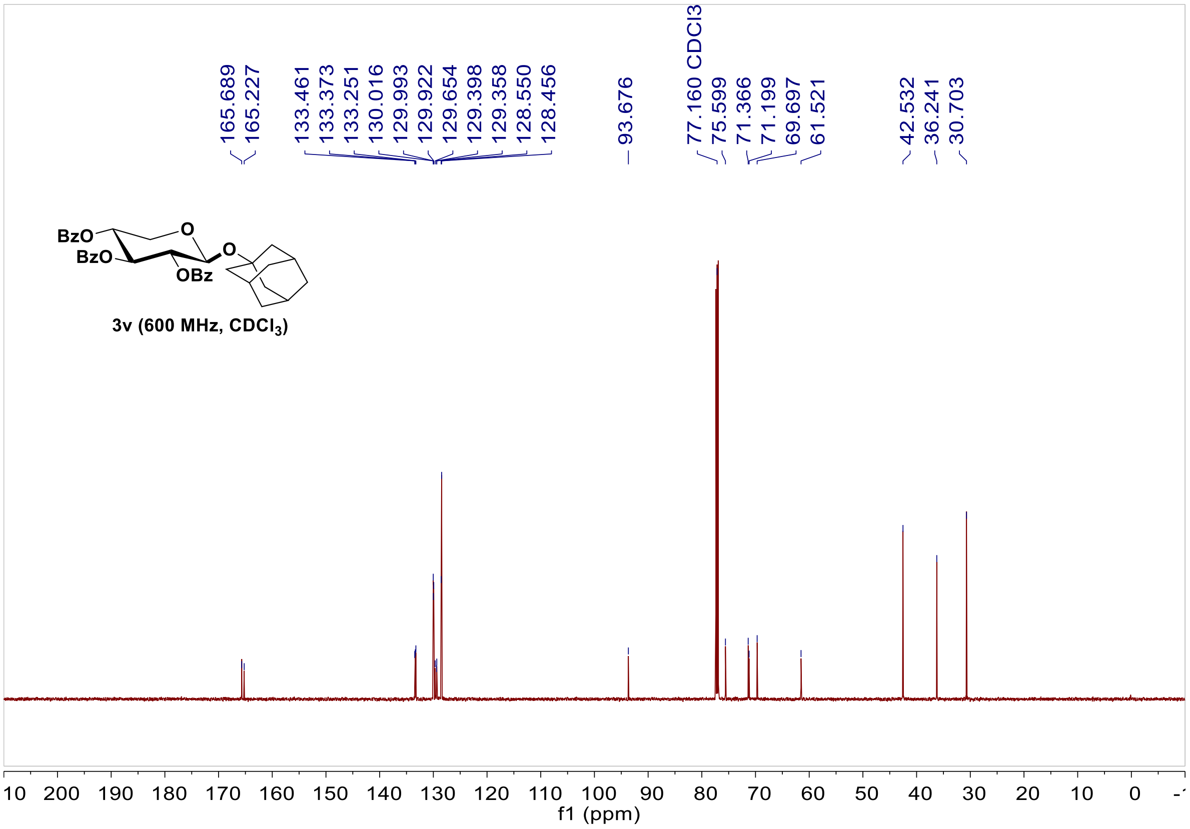
165.689
165.227
133.461
133.373
133.251
130.016
129.993
129.922
129.654
129.398
129.358
128.550
128.456
93.676
77.160 CDCl3
75.599
71.366
71.199
69.697
61.521
42.532
36.241
30.703
BzO
BzO
O
OBz
O
3v (600 MHz, CDCl3)
f1 (ppm)

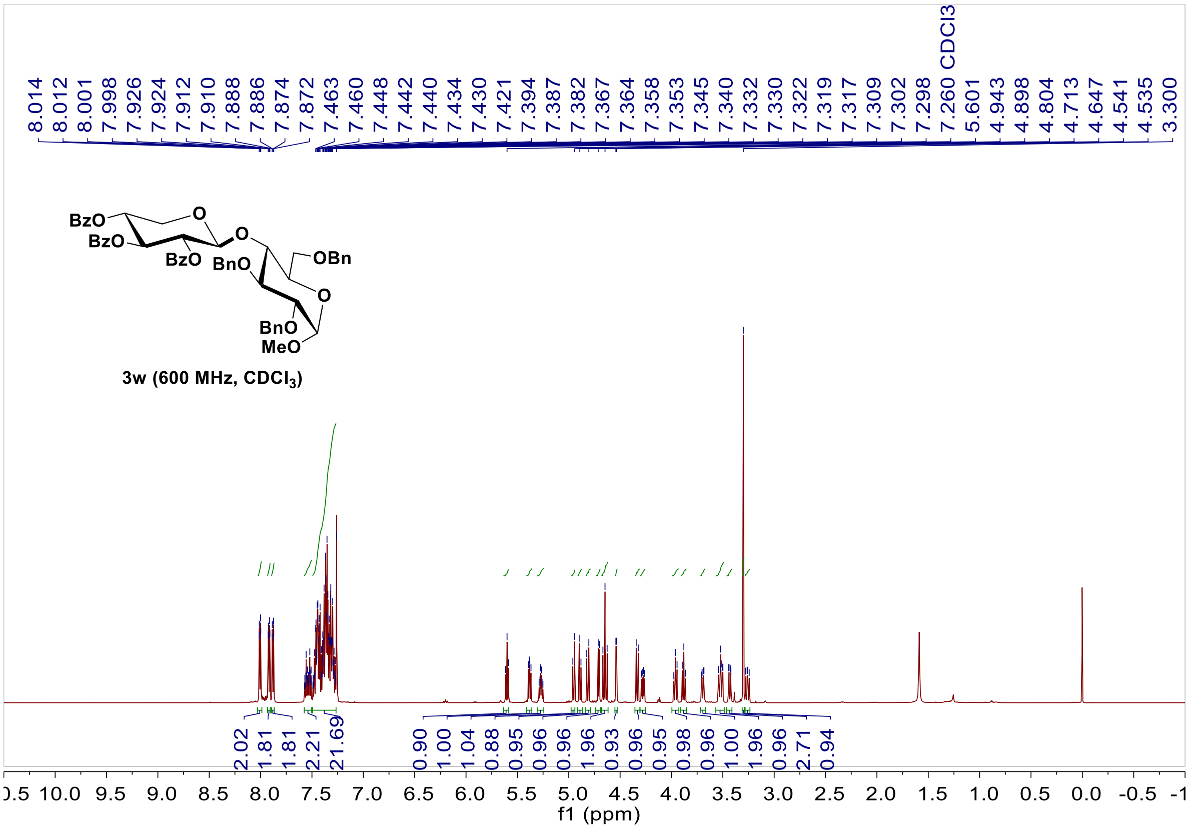
8.014
8.012
8.001
7.998
7.926
7.924
7.912
7.910
7.888
7.886
7.874
7.872
7.463
7.460
7.448
7.442
7.440
7.434
7.430
7.421
7.394
7.387
7.382
7.367
7.364
7.358
7.353
7.345
7.340
7.332
7.330
7.322
7.319
7.317
7.309
7.302
7.298
7.260 CDCl3
5.601
4.943
4.898
4.804
4.713
4.647
4.541
4.535
3.300
BzO
BzO
BzO
O
O
BnO
OBn
O
BnO
MeO
3w (600 MHz, CDCl3)
2.02
1.81
1.81
2.21
21.69
0.90
1.00
1.04
0.88
0.95
0.96
0.96
1.96
0.93
0.96
0.95
0.98
0.96
1.00
1.96
0.96
2.71
0.94
f1 (ppm)

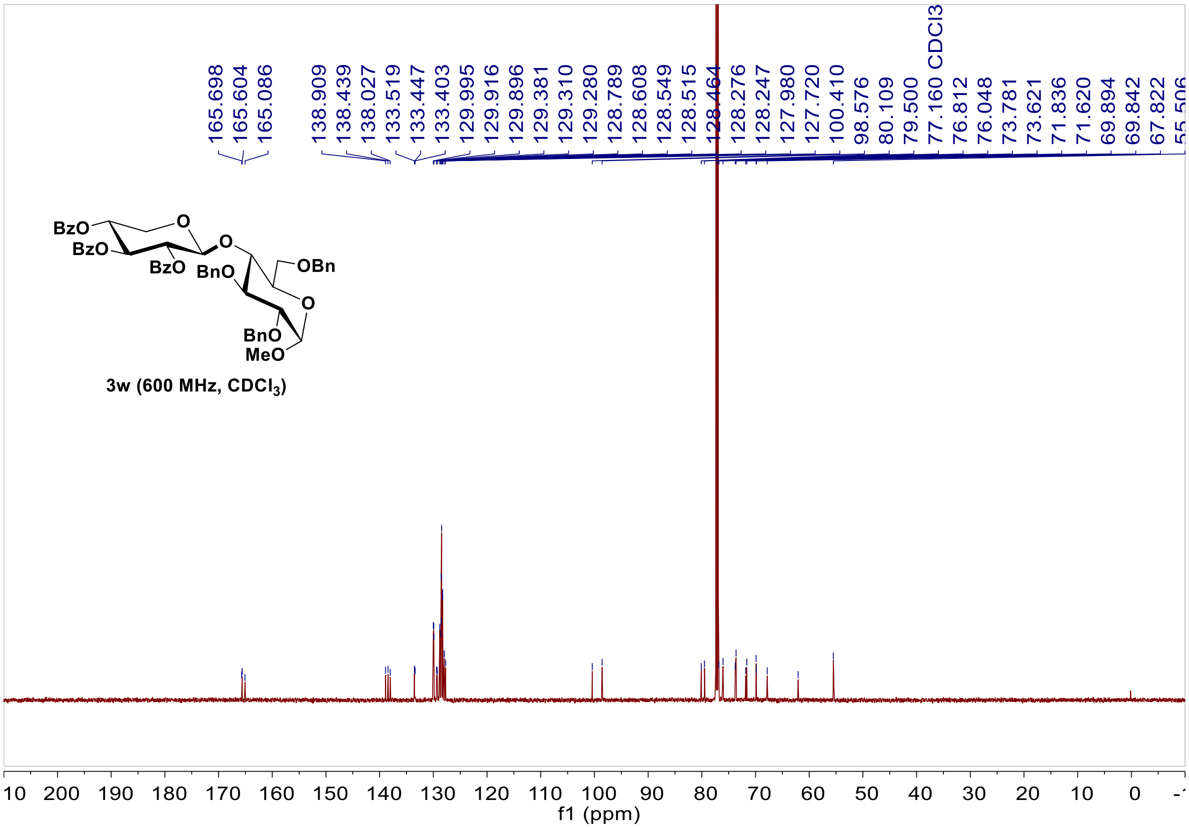
165.698
165.604
165.086
138.909
138.439
138.027
133.519
133.447
133.403
129.995
129.916
129.896
129.381
129.310
129.280
128.789
128.608
128.549
128.515
128.464
128.276
128.247
127.980
127.720
100.410
98.576
80.109
79.500
77.160 CDCl3
76.812
76.048
73.781
73.621
71.836
71.620
69.894
69.842
67.822
55.506
BzO
BzO
BzO
O
O
BnO
OBn
O
BnO
MeO
3w (600 MHz, CDCl$_3$)
f1 (ppm)

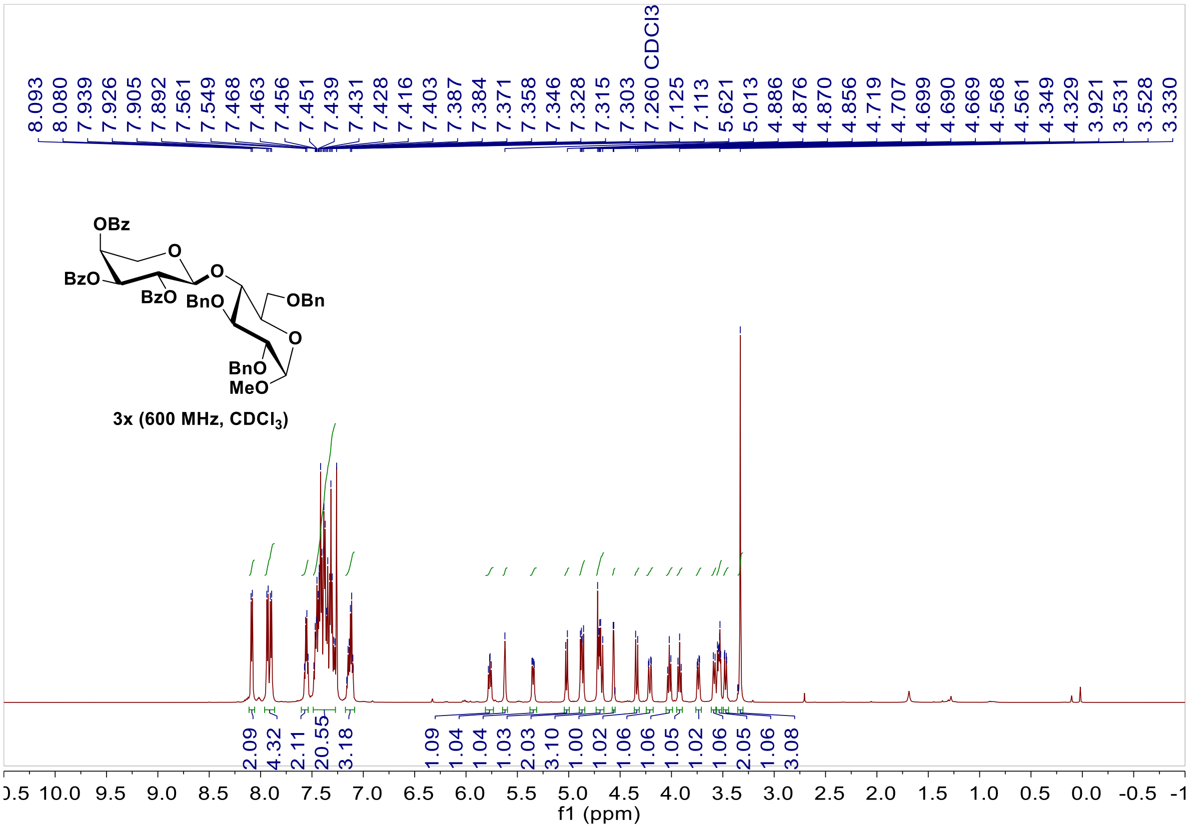
8.093
8.080
7.939
7.926
7.905
7.892
7.561
7.549
7.468
7.463
7.456
7.451
7.439
7.431
7.428
7.416
7.403
7.387
7.384
7.371
7.358
7.346
7.328
7.315
7.303
7.260 CDCl3
7.125
7.113
5.621
5.013
4.886
4.876
4.870
4.856
4.719
4.707
4.699
4.690
4.669
4.568
4.561
4.349
4.329
3.921
3.531
3.528
3.330
OBz
BzO
BzO
O
O
BnO
OBn
O
BnO
MeO
3x (600 MHz, CDCl$_3$)
2.09
4.32
2.11
20.55
3.18
1.09
1.04
1.04
1.03
2.03
3.10
1.00
1.02
1.06
1.06
1.05
1.02
1.06
2.05
1.06
3.08
f1 (ppm)

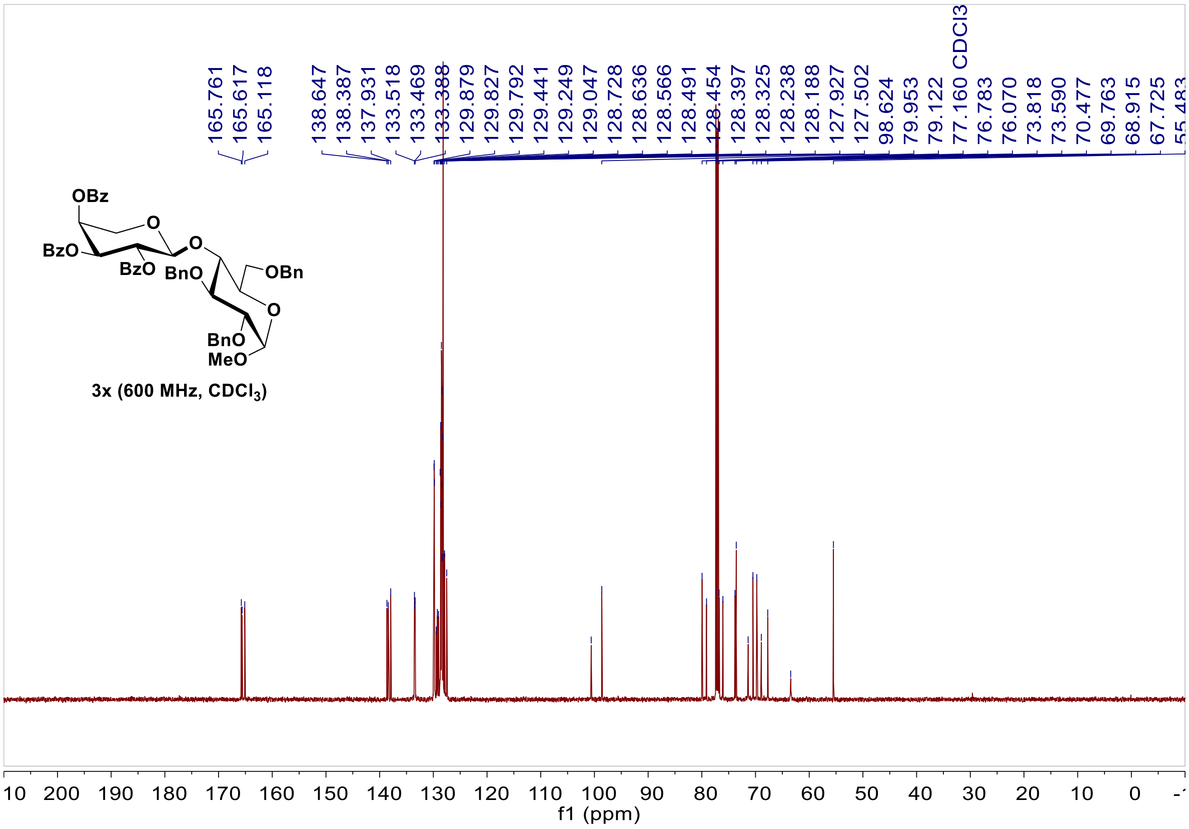

165.761
165.617
165.118
138.647
138.387
137.931
133.518
133.469
133.388
129.879
129.827
129.792
129.441
129.249
129.047
128.728
128.636
128.566
128.491
128.454
128.397
128.325
128.238
128.188
127.927
127.502
98.624
79.953
79.122
77.160 CDCl3
76.783
76.070
73.818
73.590
70.477
69.763
68.915
67.725
OBz
BzO
BzO
BnO
OBn
BnO
MeO
3x (600 MHz, CDCl3)
f1 (ppm)

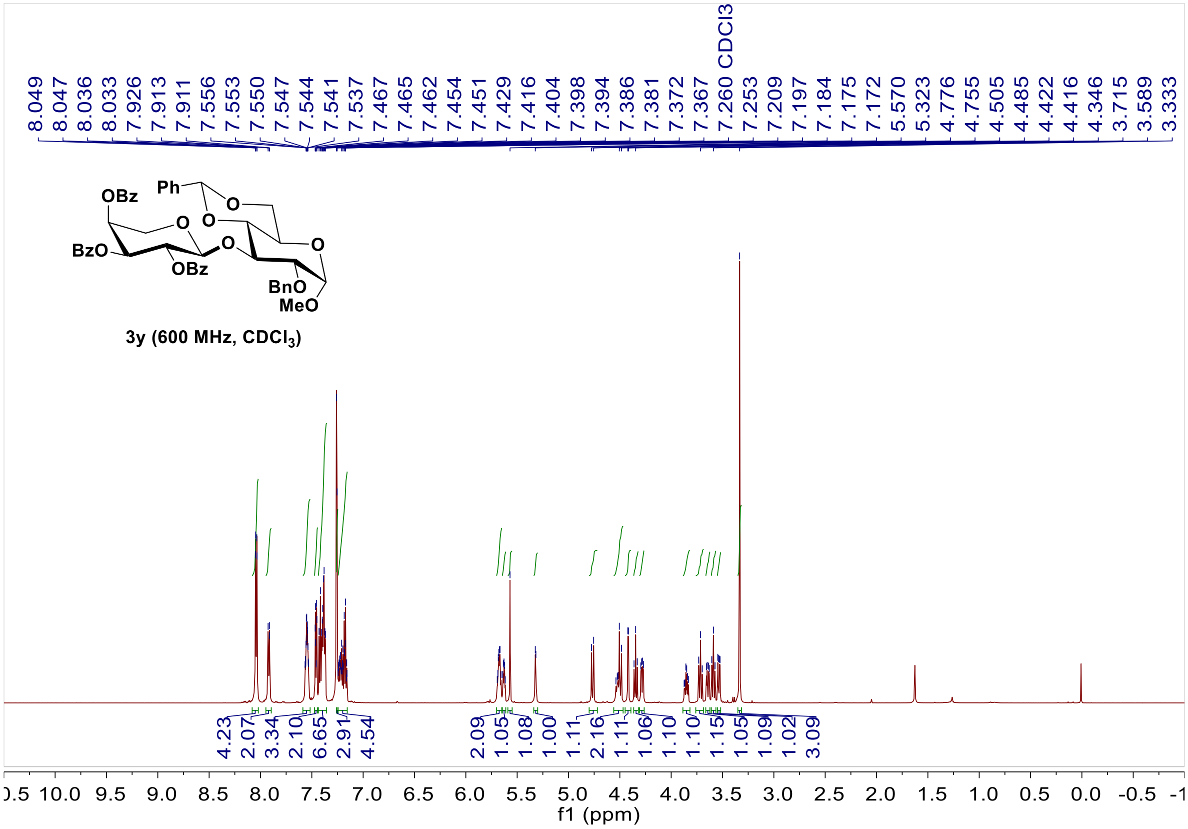

8.049
8.047
8.036
8.033
7.926
7.913
7.911
7.556
7.553
7.550
7.547
7.544
7.541
7.537
7.467
7.465
7.462
7.454
7.451
7.429
7.416
7.404
7.398
7.394
7.386
7.381
7.372
7.367
7.260 CDCl3
7.253
7.209
7.197
7.184
7.175
7.172
5.570
5.323
4.776
4.755
4.505
4.485
4.422
4.416
4.346
3.715
3.589
3.333
OBz
Ph
BzO
OBz
BnO
MeO
3y (600 MHz, CDCl3)
4.23
2.07
3.34
2.10
6.65
2.91
4.54
2.09
1.05
1.08
1.00
1.11
2.16
1.11
1.06
1.10
1.10
1.15
1.05
1.09
1.02
3.09
f1 (ppm)

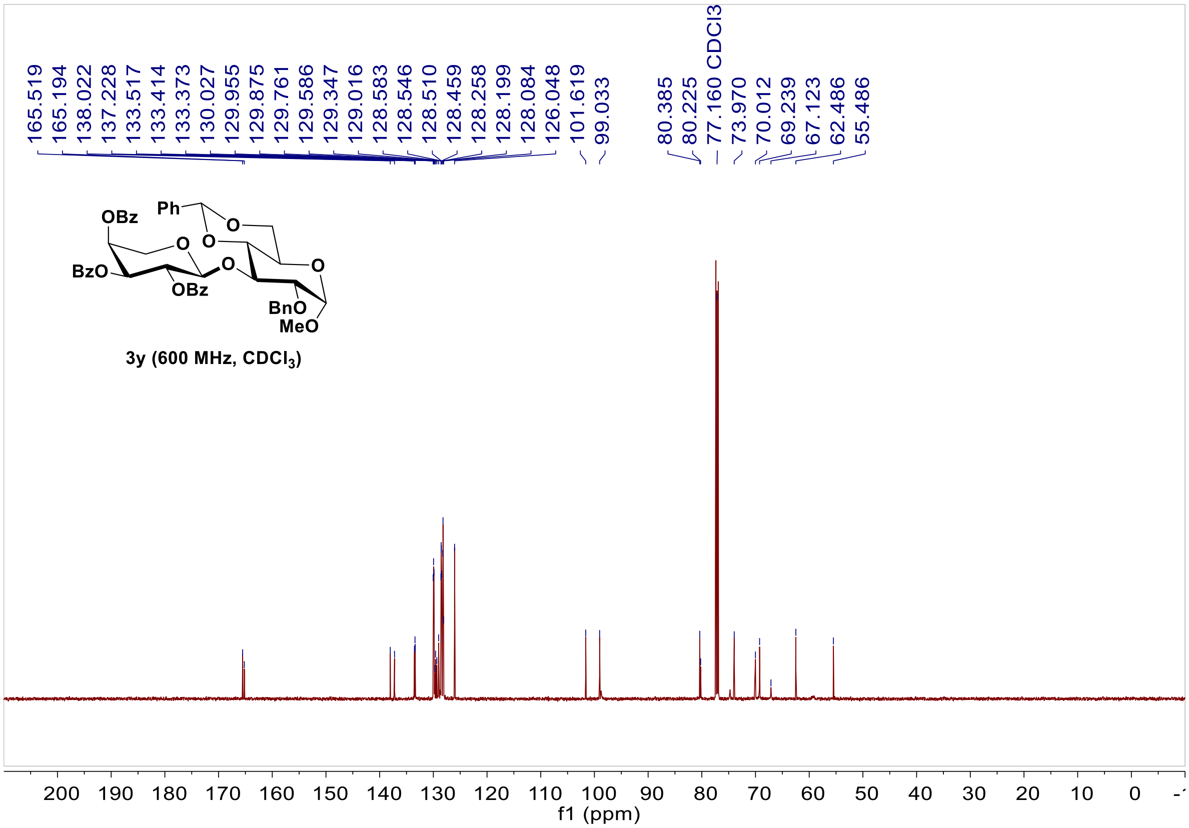
165.519
165.194
138.022
137.228
133.517
133.414
133.373
130.027
129.955
129.875
129.761
129.586
129.347
129.016
128.583
128.546
128.510
128.459
128.258
128.199
128.084
126.048
101.619
99.033
80.385
80.225
77.160 CDCl3
73.970
70.012
69.239
67.123
62.486
55.486
OBz
Ph
O
O
O
BzO
O
O
OBz
BnO
MeO
3y (600 MHz, CDCl$_3$)
200 190 180 170 160 150 140 130 120 110 100 90 80 70 60 50 40 30 20 10 0
f1 (ppm)

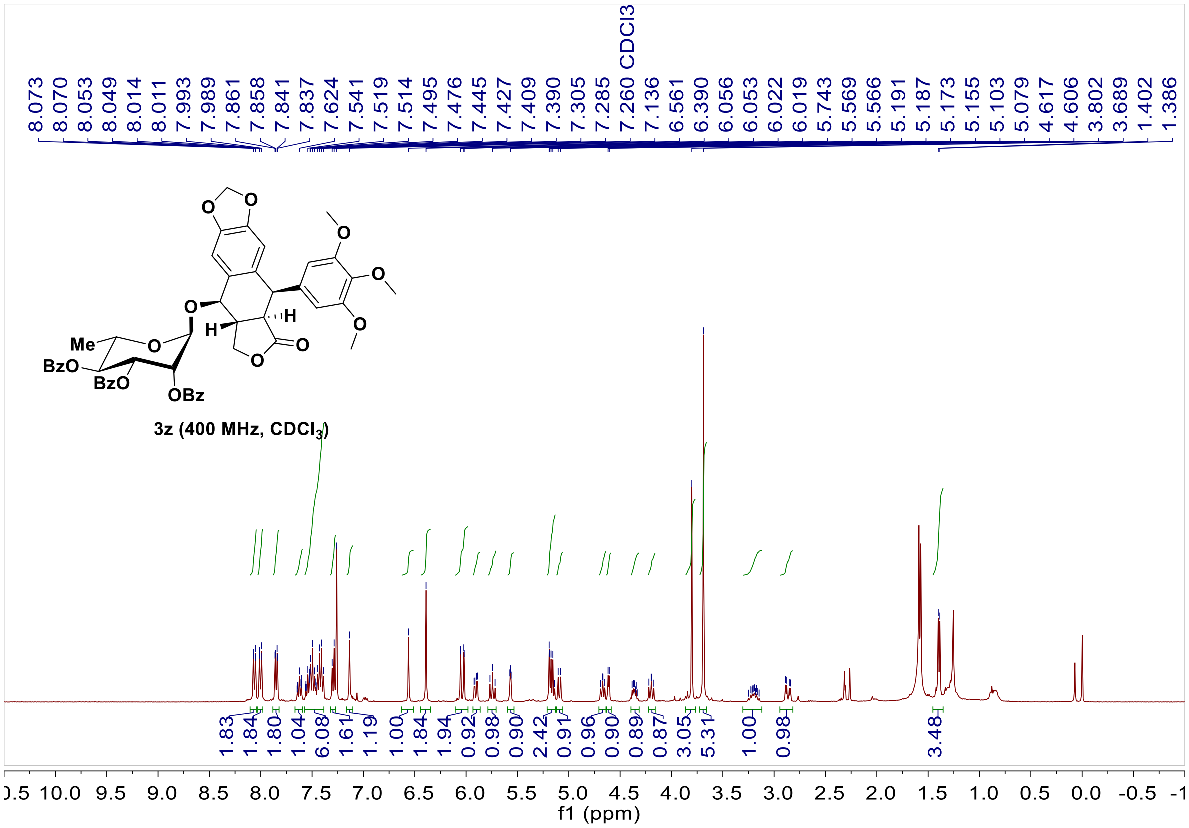
8.073
8.070
8.053
8.049
8.014
8.011
7.993
7.989
7.861
7.858
7.841
7.837
7.624
7.541
7.519
7.514
7.495
7.476
7.445
7.427
7.409
7.390
7.305
7.285
7.260 CDCl3
7.136
6.561
6.390
6.056
6.053
6.022
6.019
5.743
5.569
5.566
5.191
5.187
5.173
5.155
5.103
5.079
4.617
4.606
3.802
3.689
1.402
1.386
O
O
O
O
O
O
H
H
O
Me
BzO
O
O
BzO
OBz
3z (400 MHz, CDCl$_3$)
1.83
1.84
1.80
1.04
6.08
1.61
1.19
1.00
1.84
1.94
0.92
0.98
0.90
0.90
2.42
0.91
0.96
0.90
0.89
0.87
3.05
5.31
1.00
0.98
3.48
10.0 9.5 9.0 8.5 8.0 7.5 7.0 6.5 6.0 5.5 5.0 4.5 4.0 3.5 3.0 2.5 2.0 1.5 1.0 0.5 0.0 -0.5
f1 (ppm)

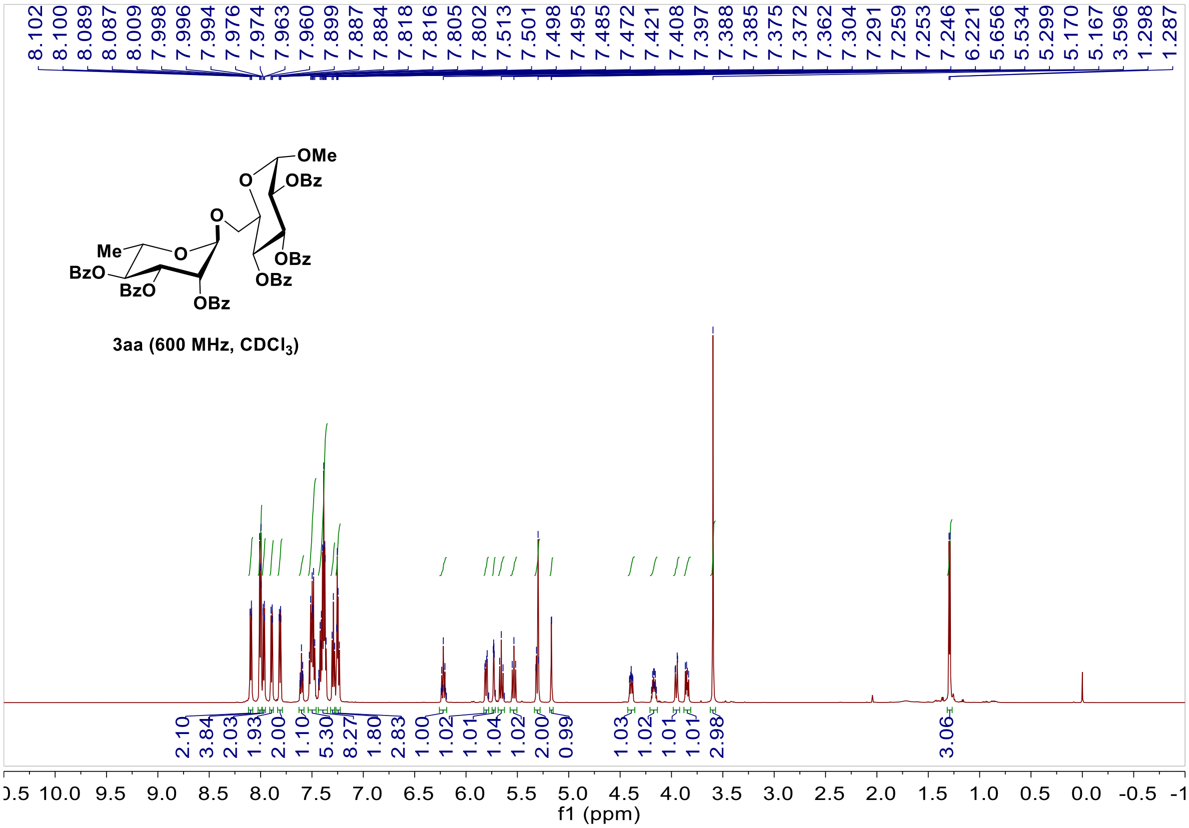
8.102
8.100
8.089
8.087
8.009
7.998
7.996
7.994
7.976
7.974
7.963
7.960
7.899
7.887
7.884
7.818
7.816
7.805
7.802
7.513
7.501
7.498
7.495
7.485
7.472
7.421
7.408
7.397
7.388
7.385
7.375
7.372
7.362
7.304
7.291
7.259
7.253
7.246
6.221
5.656
5.534
5.299
5.170
5.167
3.596
1.298
1.287
OMe
O
OBz
O
Me
O
BzO
BzO
OBz
OBz
OBz
3aa (600 MHz, $CDCl_3$)
2.10
3.84
2.03
1.93
2.00
1.10
5.30
8.27
1.80
2.83
1.00
1.02
1.01
1.04
1.02
2.00
0.99
1.03
1.02
1.01
1.01
2.98
3.06
f1 (ppm)
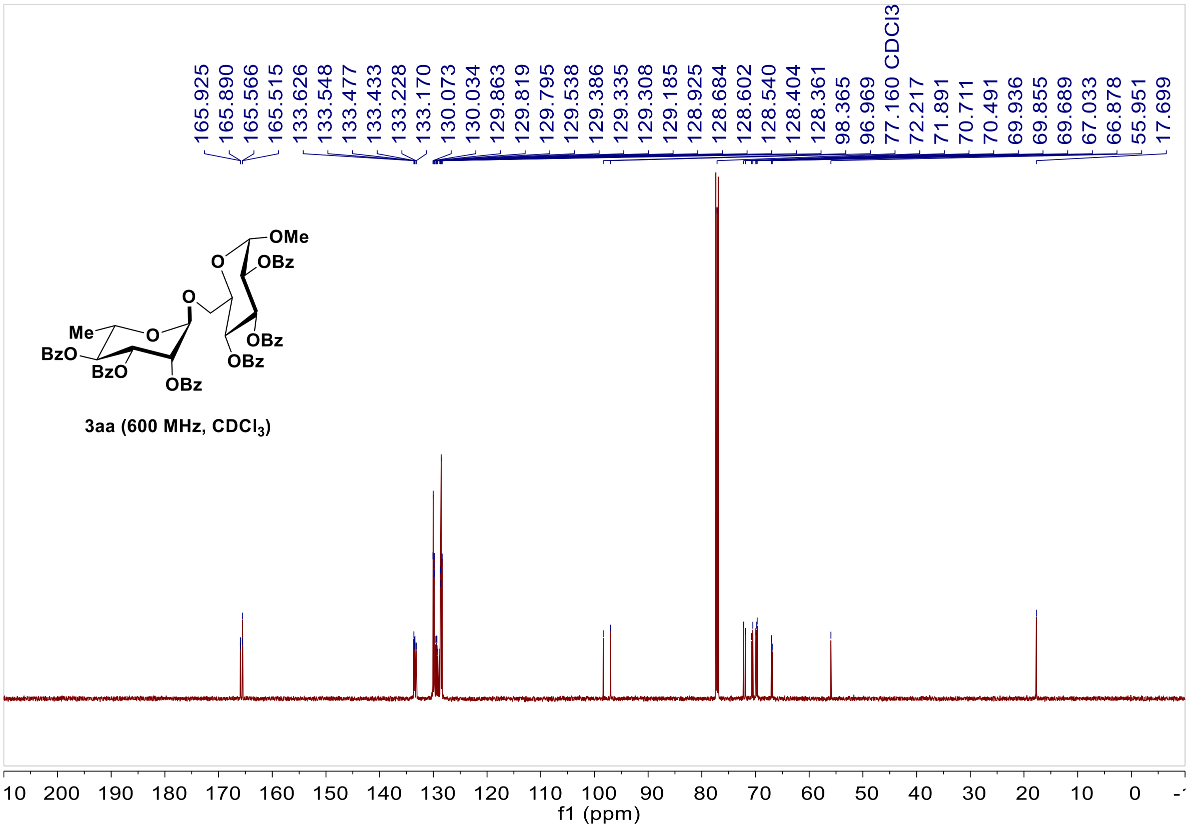
165.925
165.890
165.566
165.515
133.626
133.548
133.477
133.433
133.228
133.170
130.073
130.034
129.863
129.819
129.795
129.538
129.386
129.335
129.308
129.185
128.925
128.684
128.602
128.540
128.404
128.361
98.365
96.969
77.160 CDCl3
72.217
71.891
70.711
70.491
69.936
69.855
69.689
67.033
66.878
55.951
17.699
OMe
O
OBz
O
Me
O
BzO
BzO
OBz
OBz
OBz
3aa (600 MHz, $CDCl_3$)
f1 (ppm)

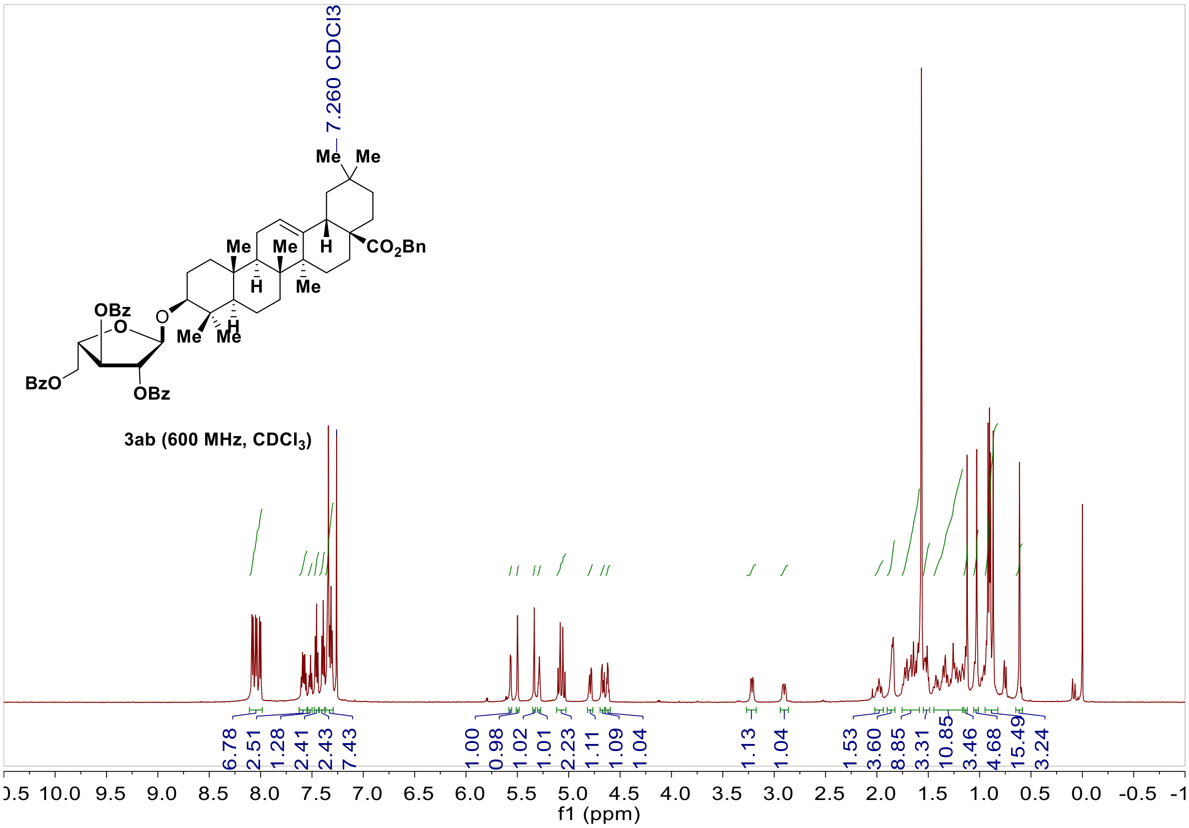
7.260 CDCl3
Me Me
Me Me H CO2Bn
H Me
OBz O
Me Me H
BzO OBz
3ab (600 MHz, CDCl3)
6.78
2.51
1.28
2.41
2.43
7.43
1.00
0.98
1.02
1.01
2.23
1.11
1.09
1.04
1.13
1.04
1.53
3.60
8.85
3.31
10.85
3.46
4.68
15.49
3.24
f1 (ppm)

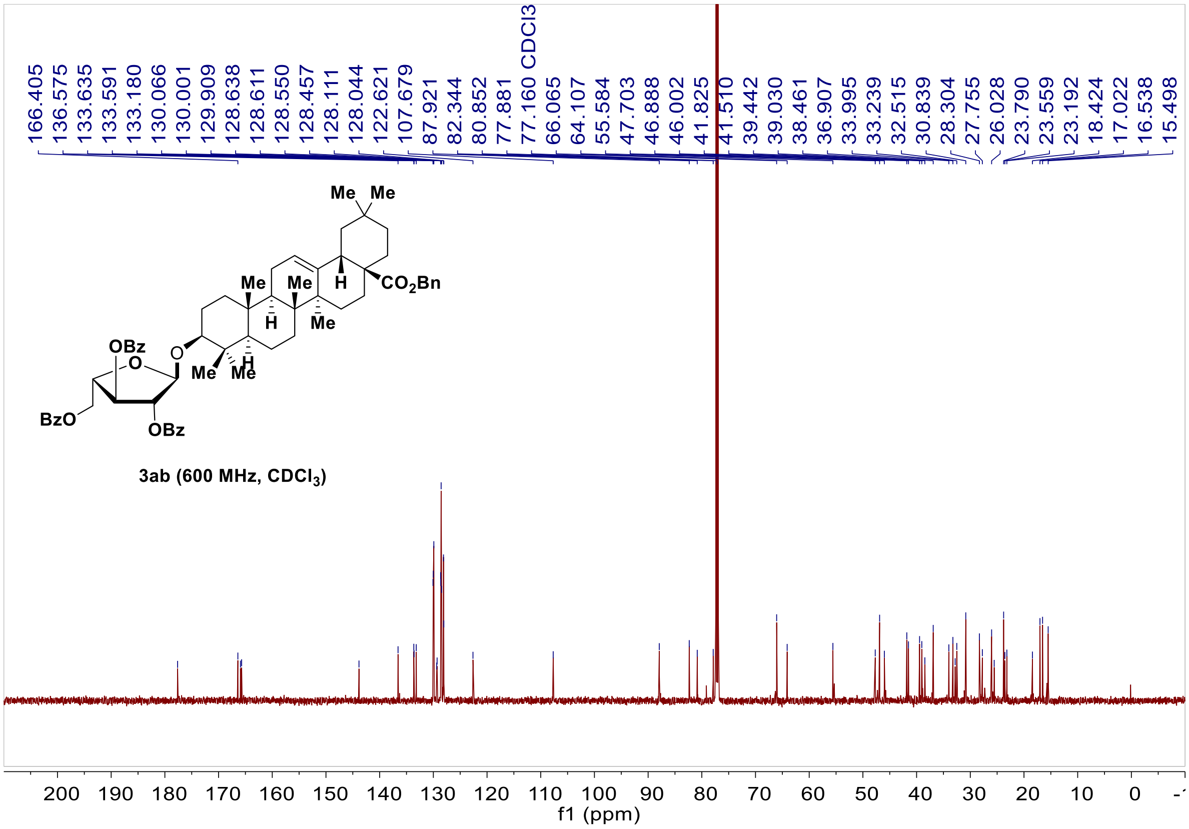
166.405
136.575
133.635
133.591
133.180
130.066
130.001
129.909
128.638
128.611
128.550
128.457
128.111
128.044
122.621
107.679
87.921
82.344
80.852
77.881
77.160 CDCl3
66.065
64.107
55.584
47.703
46.888
46.002
41.825
41.510
39.442
39.030
38.461
36.907
33.995
33.239
32.515
30.839
28.304
27.755
26.028
23.790
23.559
23.192
18.424
17.022
16.538
15.498
Me Me
Me Me H CO2Bn
H Me
OBz O
Me Me H
BzO OBz
3ab (600 MHz, CDCl3)
f1 (ppm)

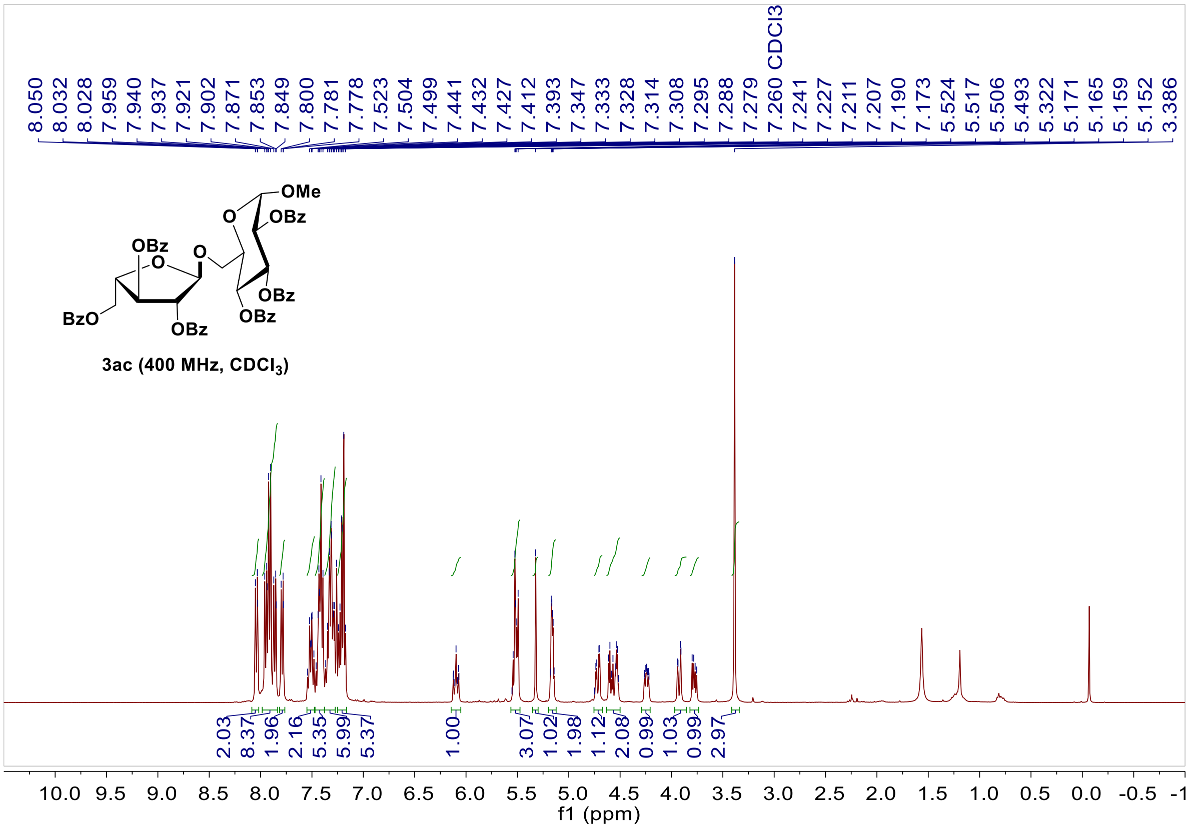
8.050 8.032 8.028 7.959 7.940 7.937 7.921 7.902 7.871 7.853 7.849 7.800 7.781 7.778 7.523 7.504 7.499 7.441 7.432 7.427 7.412 7.393 7.347 7.333 7.328 7.314 7.308 7.295 7.288 7.279 7.260 CDCl3 7.241 7.227 7.211 7.207 7.190 7.173 5.524 5.517 5.506 5.493 5.322 5.171 5.165 5.159 5.152 3.386
OMe
O
OBz
OBz
O
O
OBz
OBz
BzO
OBz
3ac (400 MHz, $CDCl_3$)
2.03 8.37 1.96 2.16 5.35 5.99 5.37 1.00 3.07 1.02 1.98 1.12 2.08 0.99 1.03 0.99 2.97
10.0 9.5 9.0 8.5 8.0 7.5 7.0 6.5 6.0 5.5 5.0 4.5 4.0 3.5 3.0 2.5 2.0 1.5 1.0 0.5 0.0 -0.5 -1
f1 (ppm)

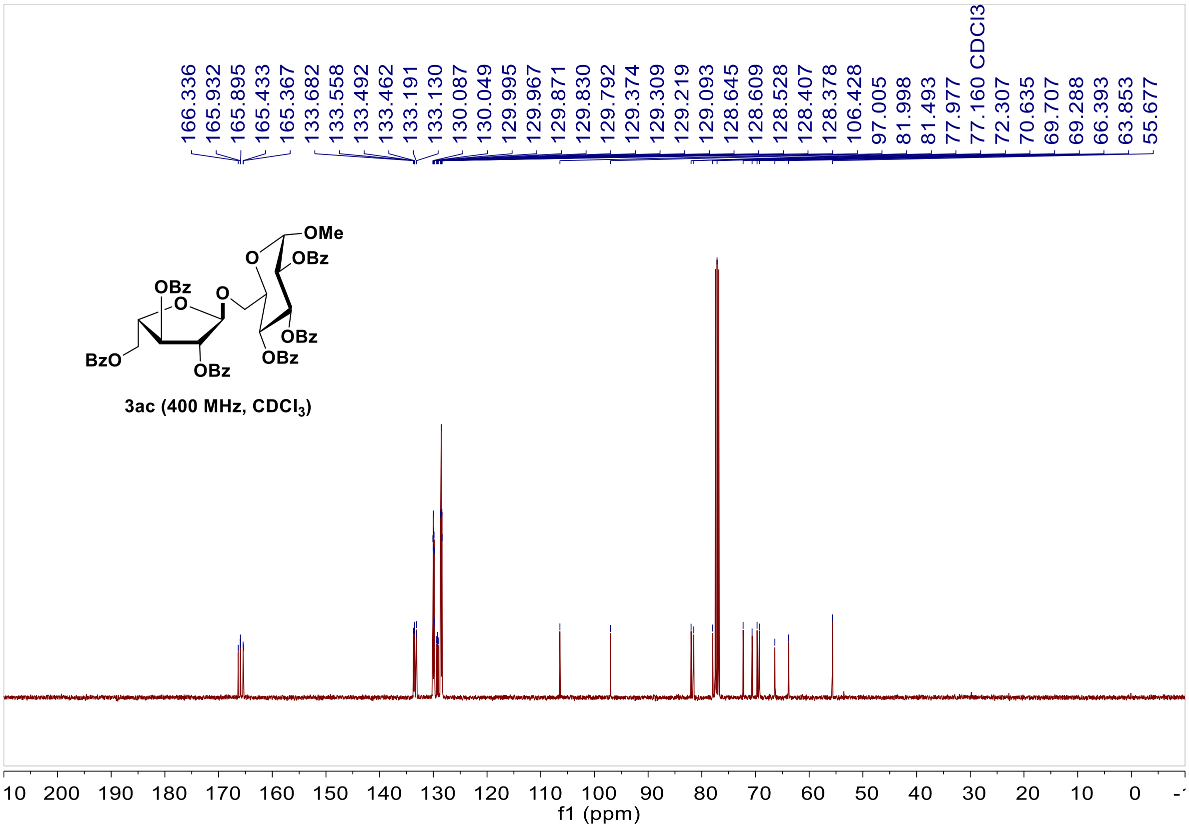
166.336 165.932 165.895 165.433 165.367 133.682 133.558 133.492 133.462 133.191 133.130 130.087 130.049 129.995 129.967 129.871 129.830 129.792 129.374 129.309 129.219 129.093 128.645 128.609 128.528 128.407 128.378 106.428 97.005 81.998 81.493 77.977 77.160 CDCl3 72.307 70.635 69.707 69.288 66.393 63.853 55.677
OMe
O
OBz
OBz
O
O
OBz
OBz
BzO
OBz
3ac (400 MHz, $CDCl_3$)
10 200 190 180 170 160 150 140 130 120 110 100 90 80 70 60 50 40 30 20 10 0
f1 (ppm)

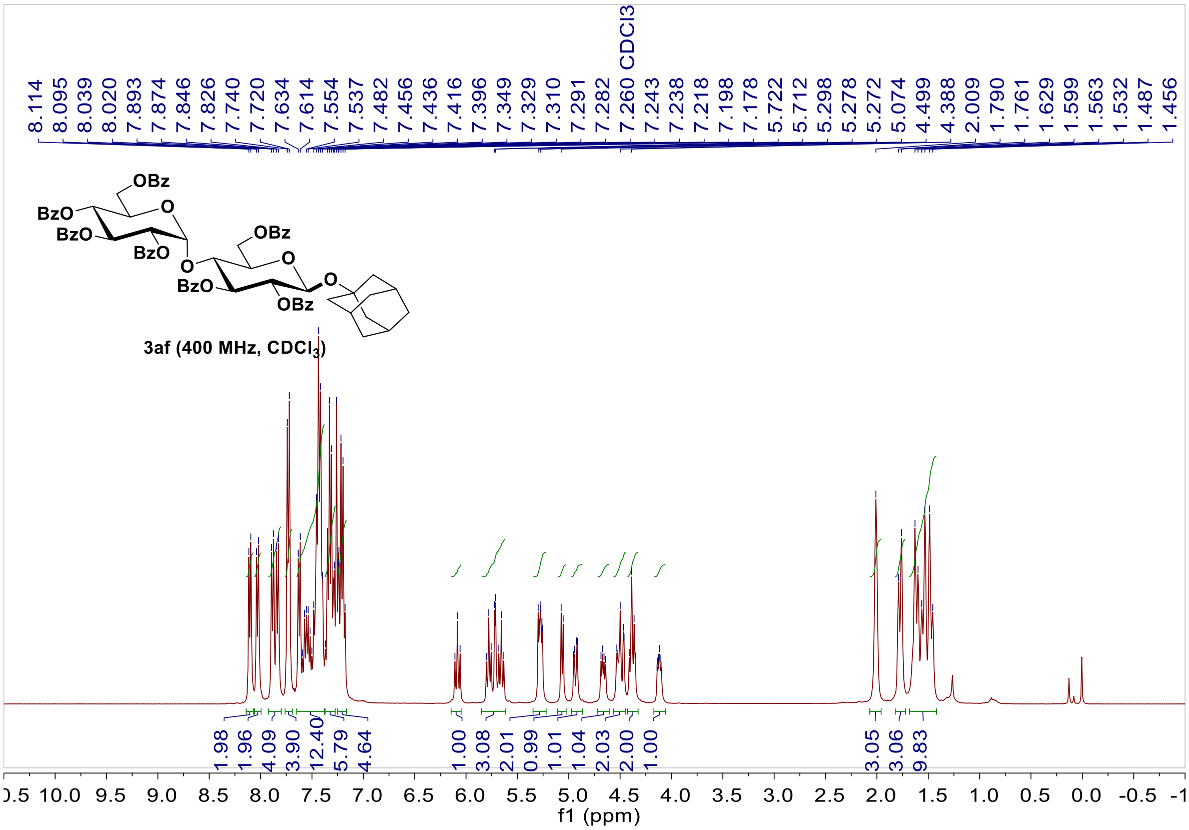
8.114 8.095 8.039 8.020 7.893 7.874 7.846 7.826 7.740 7.720 7.634 7.614 7.554 7.537 7.482 7.456 7.436 7.416 7.396 7.349 7.329 7.310 7.291 7.282 7.260 CDCl3 7.243 7.238 7.218 7.198 7.178 5.722 5.712 5.298 5.278 5.272 5.074 4.499 4.388 2.009 1.790 1.761 1.629 1.599 1.563 1.532 1.487 1.456
3af (400 MHz, CDCl$_3$)
1.98 1.96 4.09 3.90 12.40 5.79 4.64 1.00 3.08 2.01 0.99 1.01 1.04 2.03 2.00 1.00 3.05 3.06 9.83
f1 (ppm)

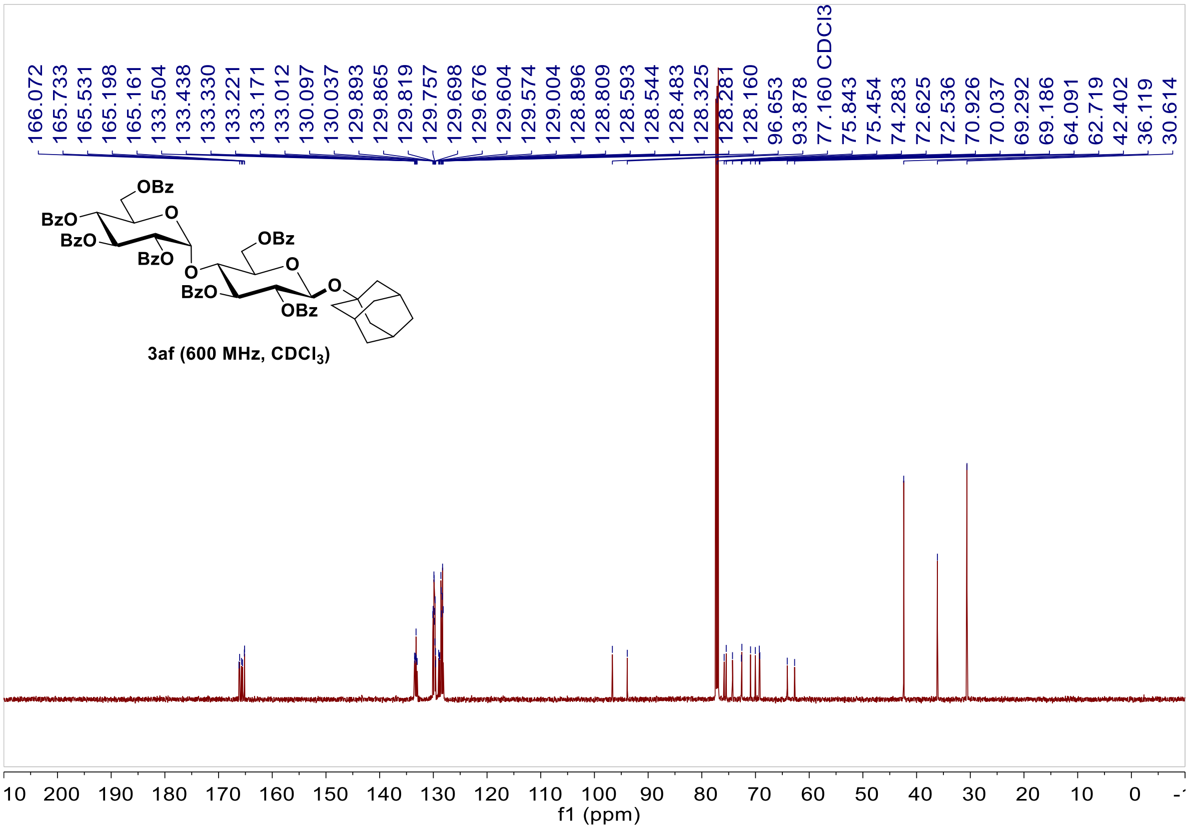
166.072 165.733 165.531 165.198 165.161 133.504 133.438 133.330 133.221 133.171 133.012 130.097 130.037 129.893 129.865 129.819 129.757 129.698 129.676 129.604 129.574 129.004 128.896 128.809 128.593 128.544 128.483 128.325 128.281 128.160 96.653 93.878 77.160 CDCl3 75.843 75.454 74.283 72.625 72.536 70.926 70.037 69.292 69.186 64.091 62.719 42.402 36.119 30.614
3af (600 MHz, CDCl$_3$)
f1 (ppm)

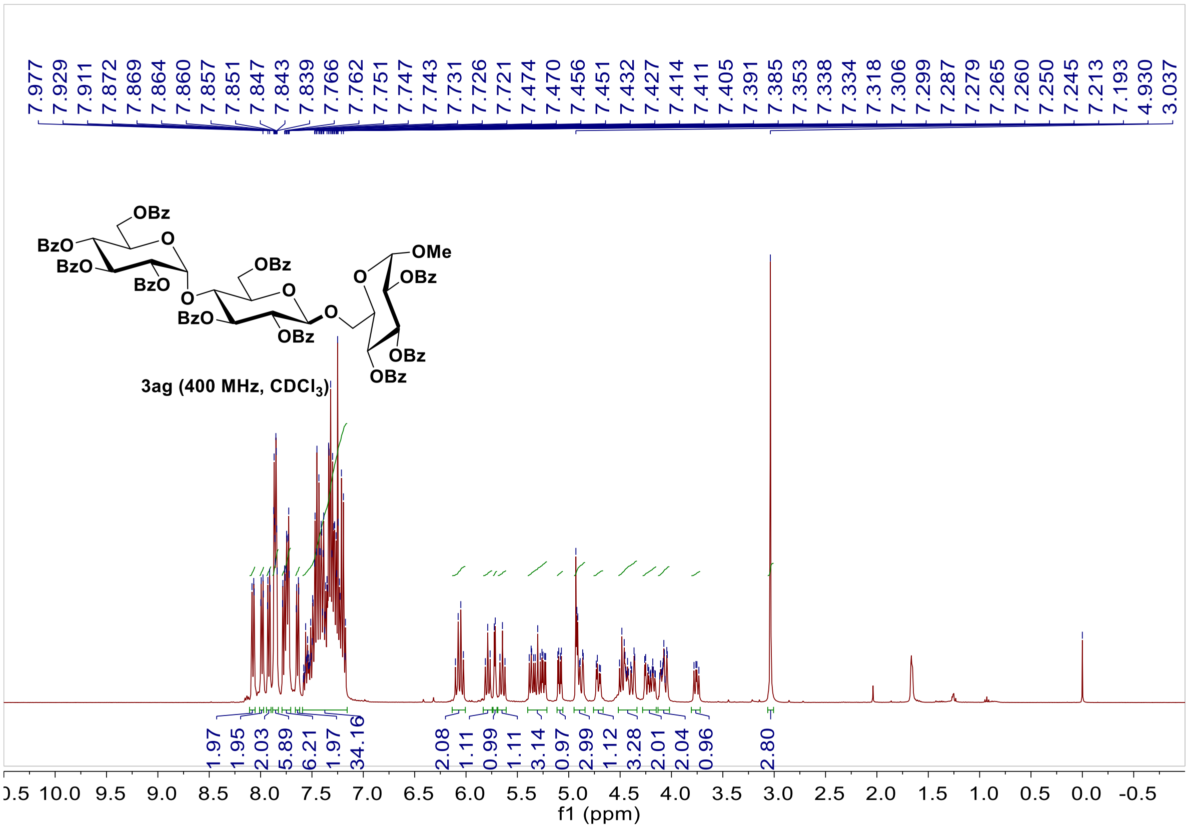
7.977 7.929 7.911 7.872 7.869 7.864 7.860 7.857 7.851 7.847 7.843 7.839 7.766 7.762 7.751 7.747 7.743 7.731 7.726 7.721 7.474 7.470 7.456 7.451 7.432 7.427 7.414 7.411 7.405 7.391 7.385 7.353 7.338 7.334 7.318 7.306 7.299 7.287 7.279 7.265 7.260 7.250 7.245 7.213 7.193 4.930 3.037
OBz
BzO
O
OMe
BzO
OBz
O
BzO
O
O
OBz
BzO
OBz
OBz
OBz
3ag (400 MHz, CDCl3)
1.97 1.95 2.03 5.89 6.21 1.97 34.16 2.08 1.11 0.99 1.11 3.14 0.97 2.99 1.12 3.28 2.01 2.04 0.96 2.80
10.5 10.0 9.5 9.0 8.5 8.0 7.5 7.0 6.5 6.0 5.5 5.0 4.5 4.0 3.5 3.0 2.5 2.0 1.5 1.0 0.5 0.0 -0.5
f1 (ppm)

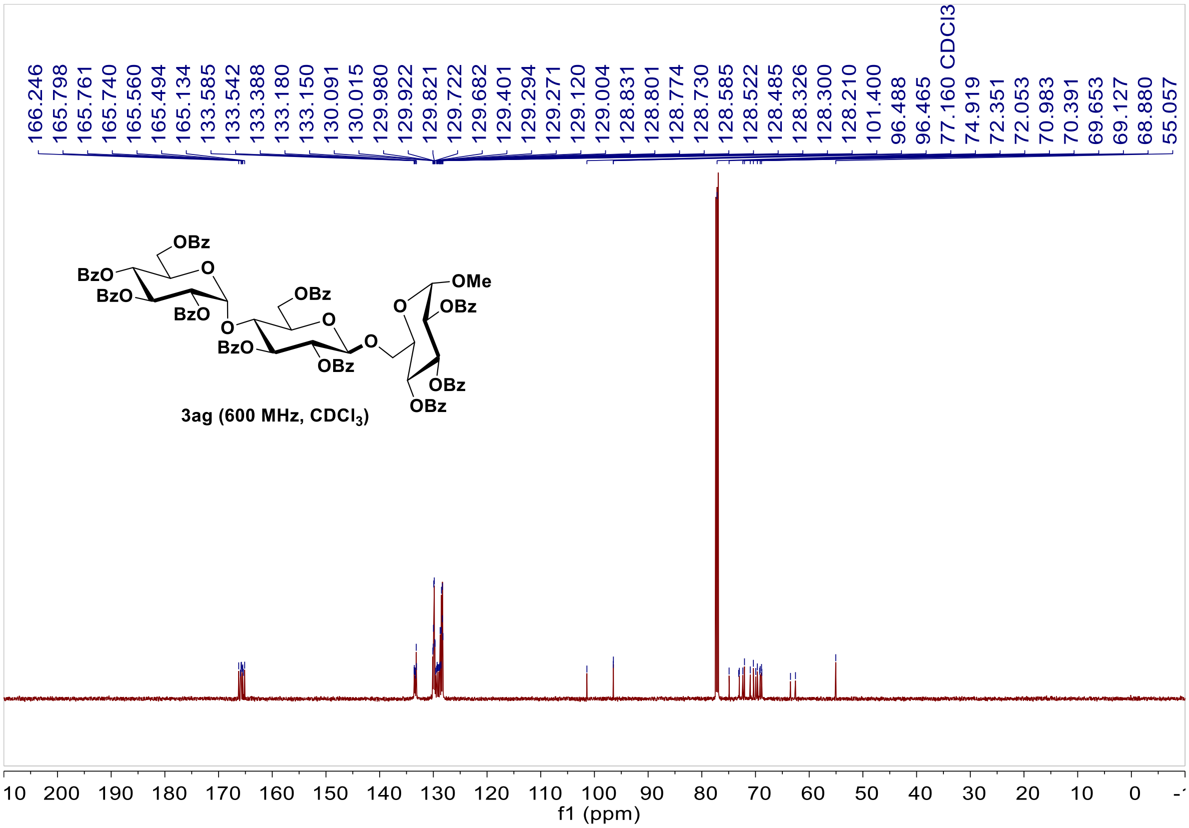
166.246 165.798 165.761 165.740 165.560 165.494 165.134 133.585 133.542 133.388 133.180 133.150 130.091 130.015 129.980 129.922 129.821 129.722 129.682 129.401 129.294 129.271 129.120 129.004 128.831 128.801 128.774 128.730 128.585 128.522 128.485 128.326 128.300 128.210 101.400 96.488 96.465 77.160 CDCl3 74.919 72.351 72.053 70.983 70.391 69.653 69.127 68.880 55.057
OBz
BzO
O
OMe
BzO
OBz
O
BzO
O
O
OBz
BzO
OBz
OBz
OBz
3ag (600 MHz, CDCl3)
210 200 190 180 170 160 150 140 130 120 110 100 90 80 70 60 50 40 30 20 10 0 -10
f1 (ppm)

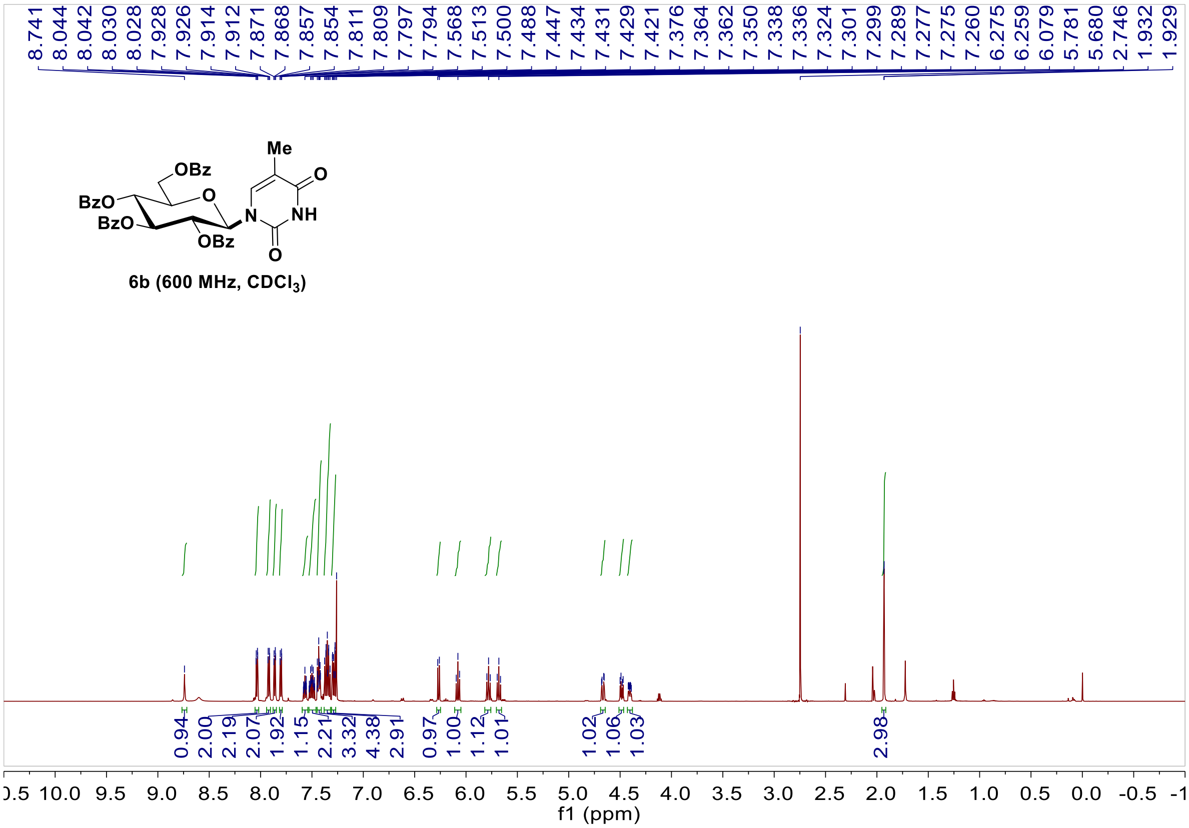
8.741 8.044 8.042 8.030 8.028 7.928 7.926 7.914 7.912 7.871 7.868 7.857 7.854 7.811 7.809 7.797 7.794 7.568 7.513 7.500 7.488 7.447 7.434 7.431 7.429 7.421 7.376 7.364 7.362 7.350 7.338 7.336 7.324 7.301 7.299 7.289 7.277 7.275 7.260 6.275 6.259 6.079 5.781 5.680 2.746 1.932 1.929
Me
OBz
O
BzO
BzO
OBz
N
NH
O
O
6b (600 MHz, CDCl3)
0.94 2.00 2.19 2.07 1.92 1.15 2.21 3.32 4.38 2.91 0.97 1.00 1.12 1.01 1.02 1.06 1.03 2.98
10.0 9.5 9.0 8.5 8.0 7.5 7.0 6.5 6.0 5.5 5.0 4.5 4.0 3.5 3.0 2.5 2.0 1.5 1.0 0.5 0.0 -0.5
f1 (ppm)

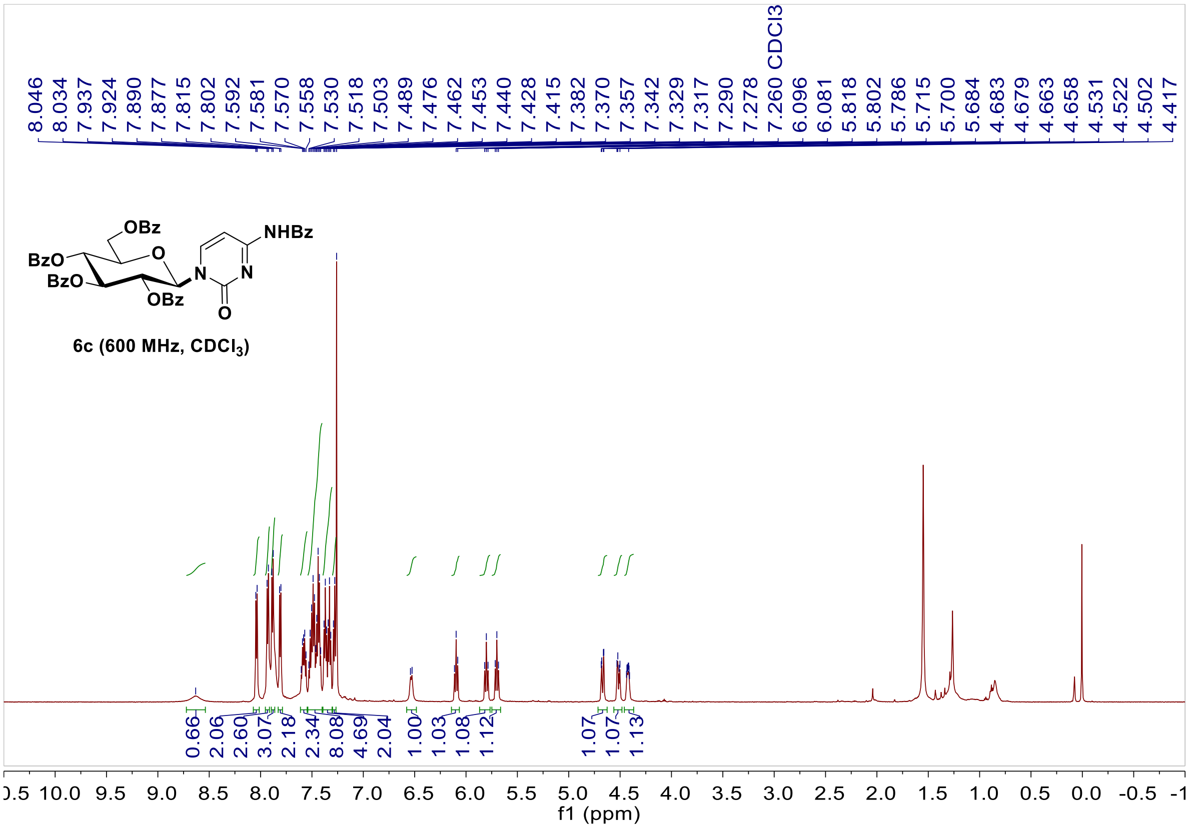
CDCl3
8.046 8.034 7.937 7.924 7.890 7.877 7.815 7.802 7.592 7.581 7.570 7.558 7.530 7.518 7.503 7.489 7.476 7.462 7.453 7.440 7.428 7.415 7.382 7.370 7.357 7.342 7.329 7.317 7.290 7.278 7.260 6.096 6.081 5.818 5.802 5.786 5.715 5.700 5.684 4.683 4.679 4.663 4.658 4.531 4.522 4.502 4.417
OBz
O
NHBz
BzO
BzO
OBz
N
N
O
6c (600 MHz, CDCl3)
0.66 2.06 2.60 3.07 2.18 2.34 8.08 4.69 2.04 1.00 1.03 1.08 1.12 1.07 1.07 1.13
10.0 9.5 9.0 8.5 8.0 7.5 7.0 6.5 6.0 5.5 5.0 4.5 4.0 3.5 3.0 2.5 2.0 1.5 1.0 0.5 0.0 -0.5
f1 (ppm)

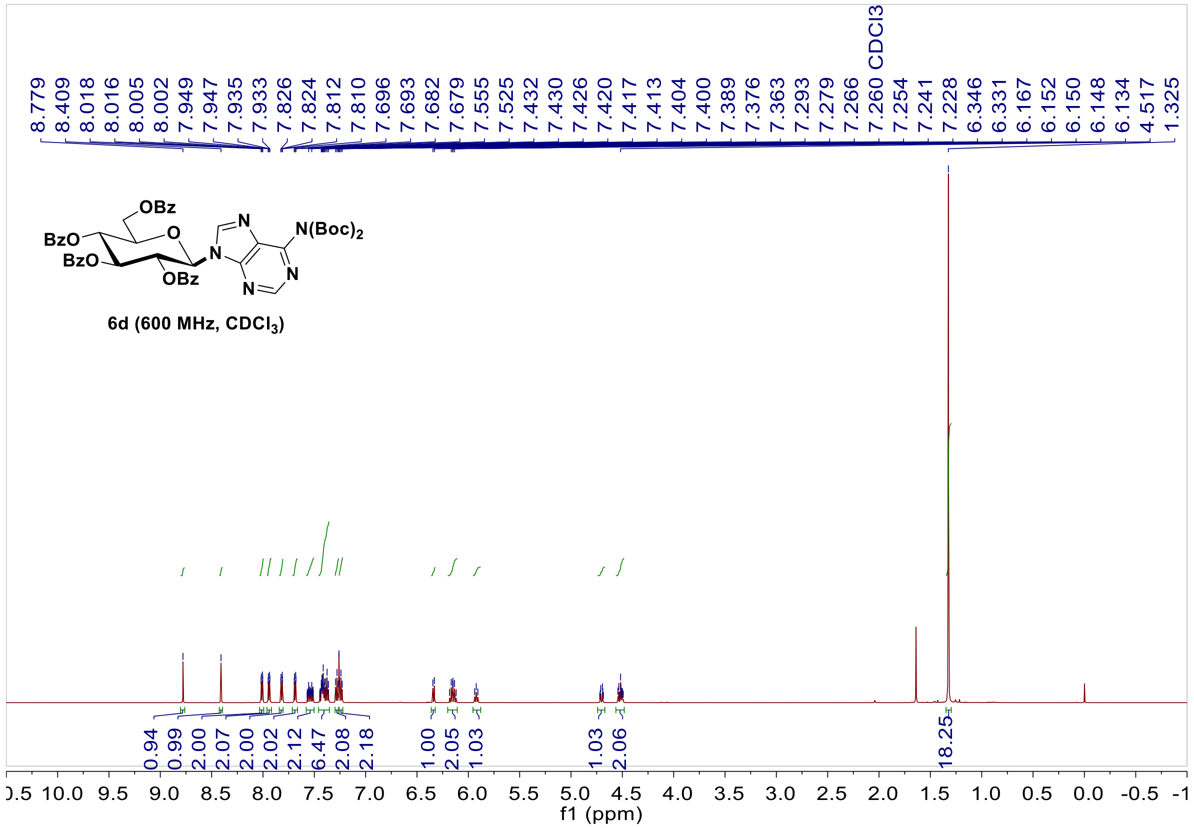
8.779 8.409 8.018 8.016 8.005 8.002 7.949 7.947 7.935 7.933 7.826 7.824 7.812 7.810 7.696 7.693 7.682 7.679 7.555 7.525 7.432 7.430 7.426 7.420 7.417 7.413 7.404 7.400 7.389 7.376 7.363 7.293 7.279 7.266 7.260 CDCl3 7.254 7.241 7.228 6.346 6.331 6.167 6.152 6.150 6.148 6.134 4.517 1.325
OBz
BzO
BzO
O
OBz
N
N
N(Boc)2
N
N
6d (600 MHz, CDCl3)
0.94 0.99 2.00 2.07 2.00 2.02 2.12 6.47 2.08 2.18 1.00 2.05 1.03 1.03 2.06 18.25
f1 (ppm)

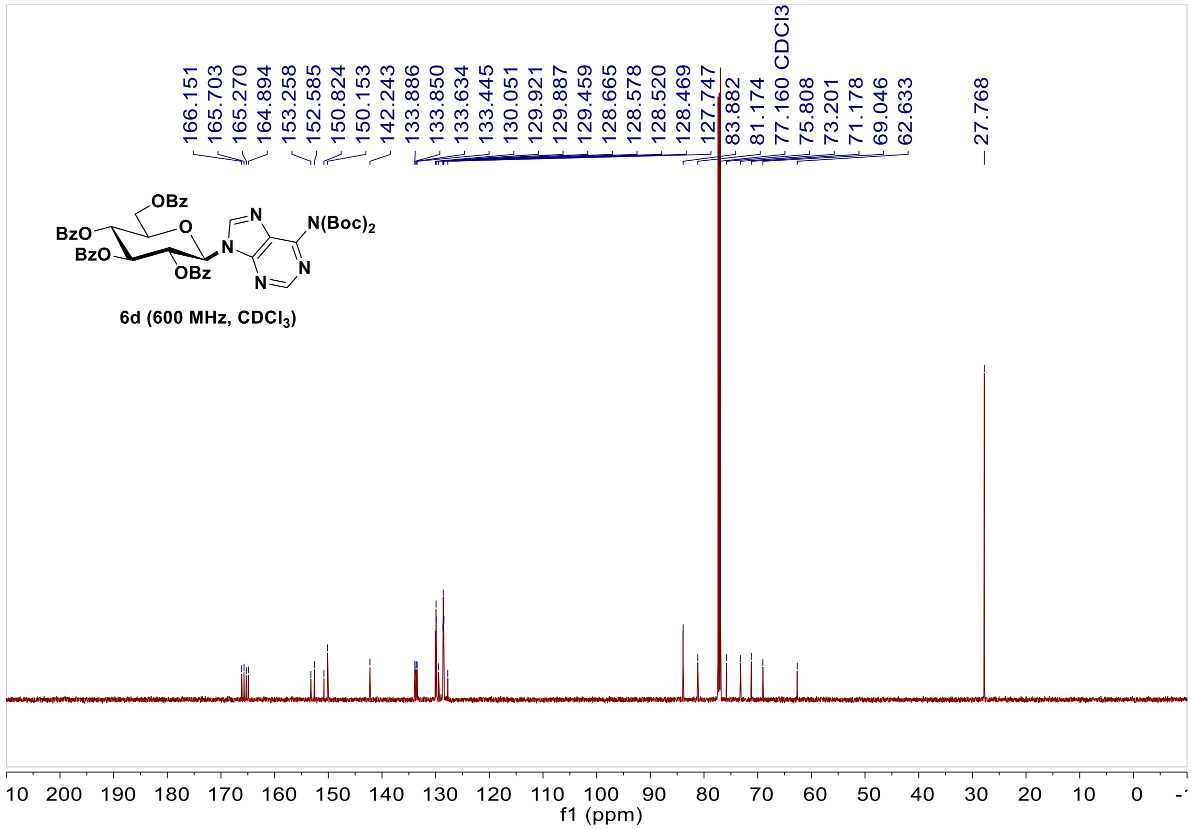
166.151 165.703 165.270 164.894 153.258 152.585 150.824 150.153 142.243 133.886 133.850 133.634 133.445 130.051 129.921 129.887 129.459 128.665 128.578 128.520 128.469 127.747 83.882 81.174 77.160 CDCl3 75.808 73.201 71.178 69.046 62.633 27.768
OBz
BzO
BzO
O
OBz
N
N
N(Boc)2
N
N
6d (600 MHz, CDCl3)
f1 (ppm)

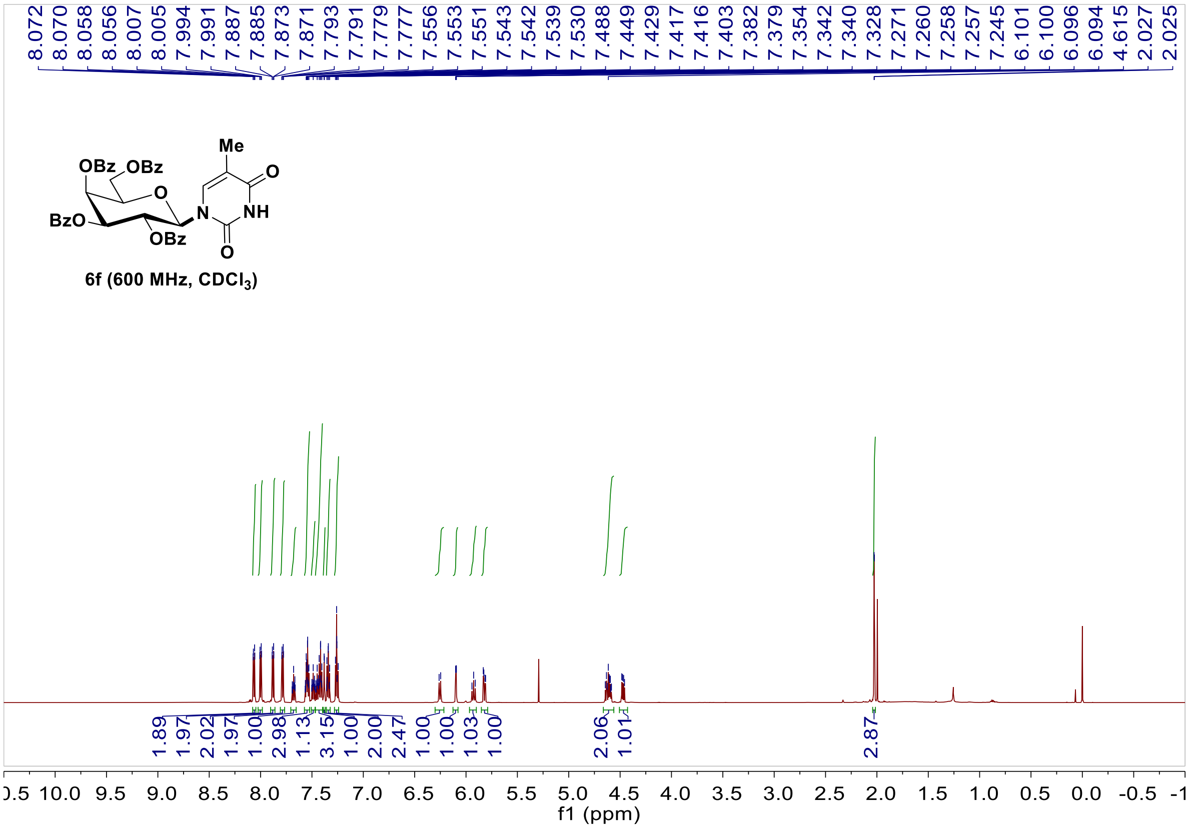
6f (600 MHz, CDCl3)
f1 (ppm)

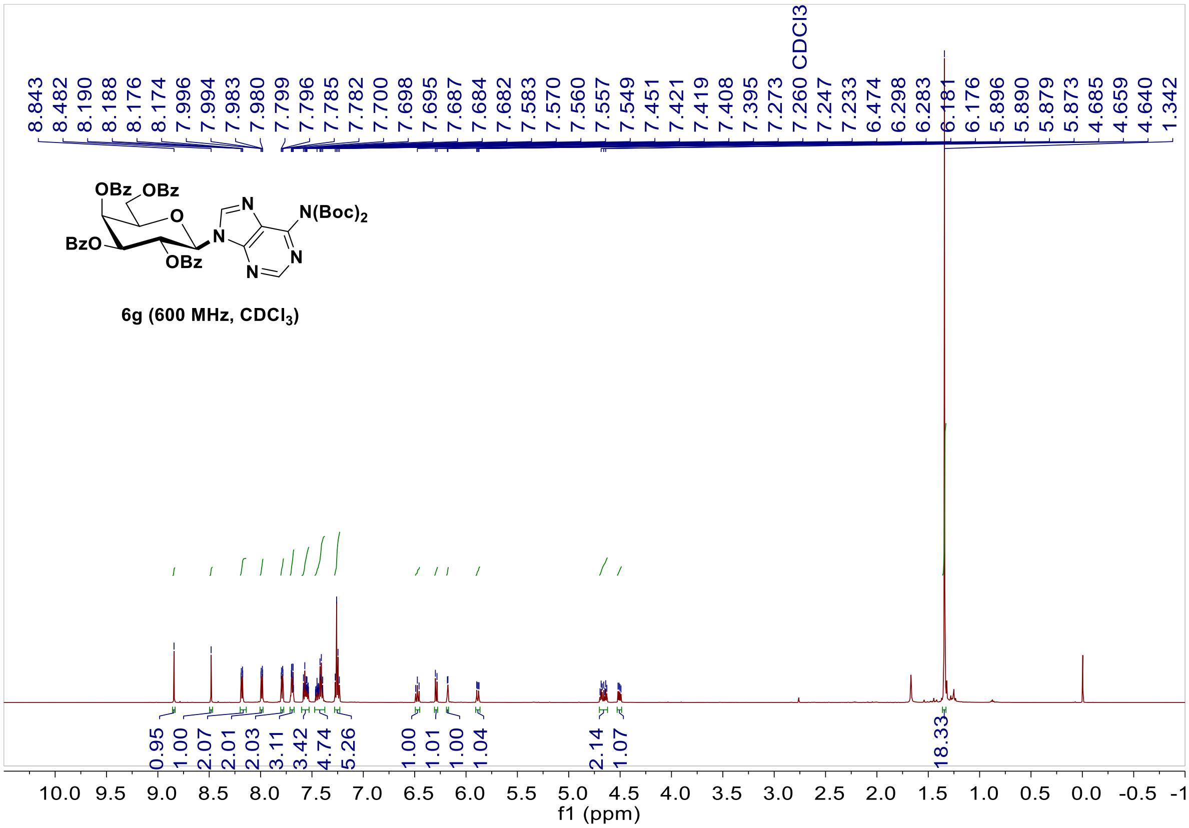
6g (600 MHz, CDCl3)
f1 (ppm)

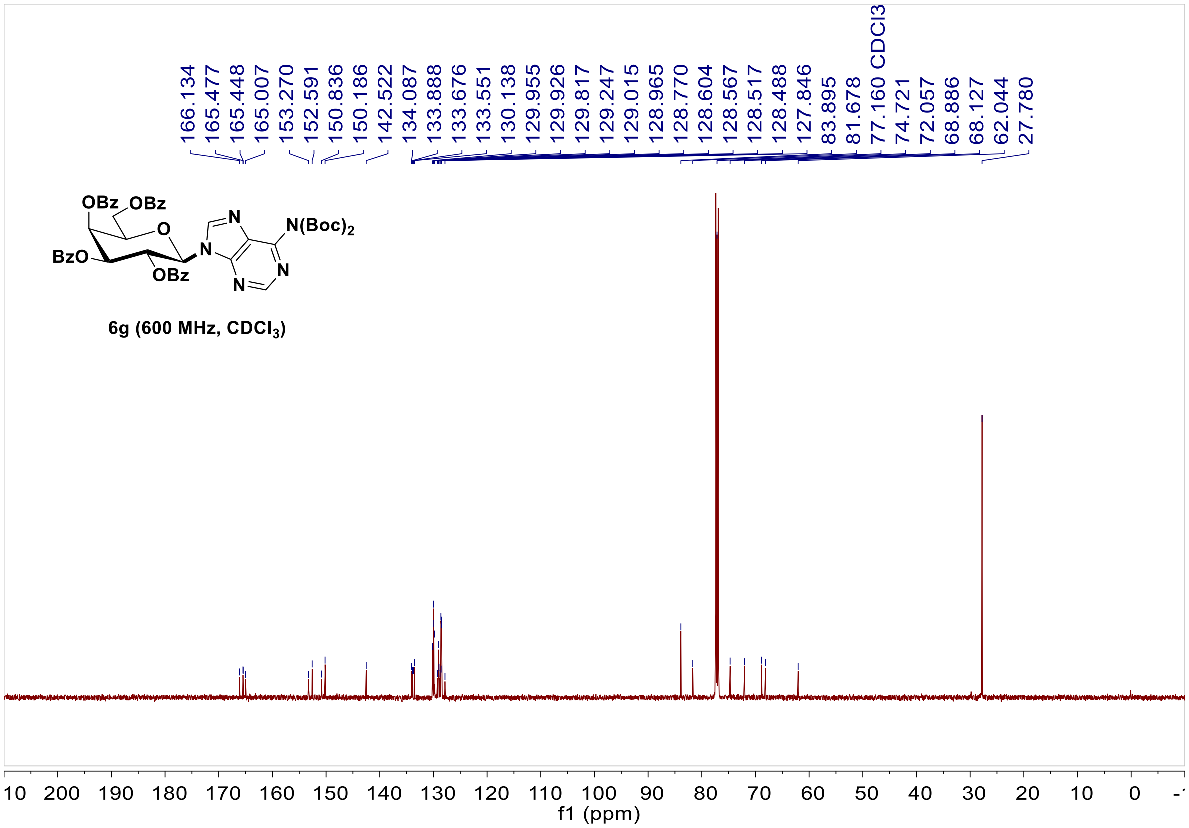

166.134
165.477
165.448
165.007
153.270
152.591
150.836
150.186
142.522
134.087
133.888
133.676
133.551
130.138
129.955
129.926
129.817
129.247
129.015
128.965
128.770
128.604
128.567
128.517
128.488
127.846
83.895
81.678
77.160 CDCl3
74.721
72.057
68.886
68.127
62.044
27.780
OBz
OBz
O
BzO
OBz
N
N(Boc)2
N
N
N
6g (600 MHz, CDCl3)
10 200 190 180 170 160 150 140 130 120 110 100 90 80 70 60 50 40 30 20 10 0
f1 (ppm)

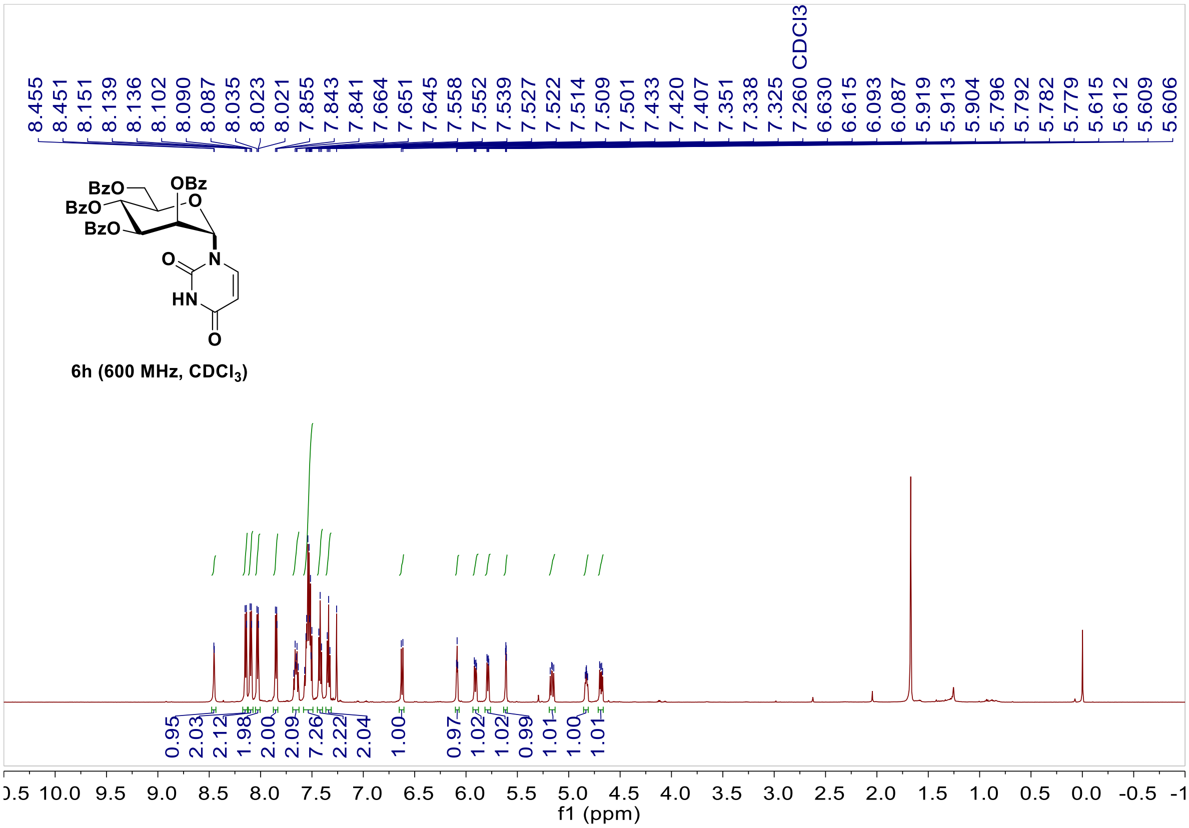

8.455
8.451
8.151
8.139
8.136
8.102
8.090
8.087
8.035
8.023
8.021
7.855
7.843
7.841
7.664
7.651
7.645
7.558
7.552
7.539
7.527
7.522
7.514
7.509
7.501
7.433
7.420
7.407
7.351
7.338
7.325
7.260 CDCl3
6.630
6.615
6.093
6.087
5.919
5.913
5.904
5.796
5.792
5.782
5.779
5.615
5.612
5.609
5.606
BzO
OBz
BzO
O
BzO
O
N
HN
O
6h (600 MHz, CDCl3)
0.95
2.03
2.12
1.98
2.00
2.09
7.26
2.22
2.04
1.00
0.97
1.02
1.02
0.99
1.01
1.00
1.01
10.0 9.5 9.0 8.5 8.0 7.5 7.0 6.5 6.0 5.5 5.0 4.5 4.0 3.5 3.0 2.5 2.0 1.5 1.0 0.5 0.0 -0.5
f1 (ppm)

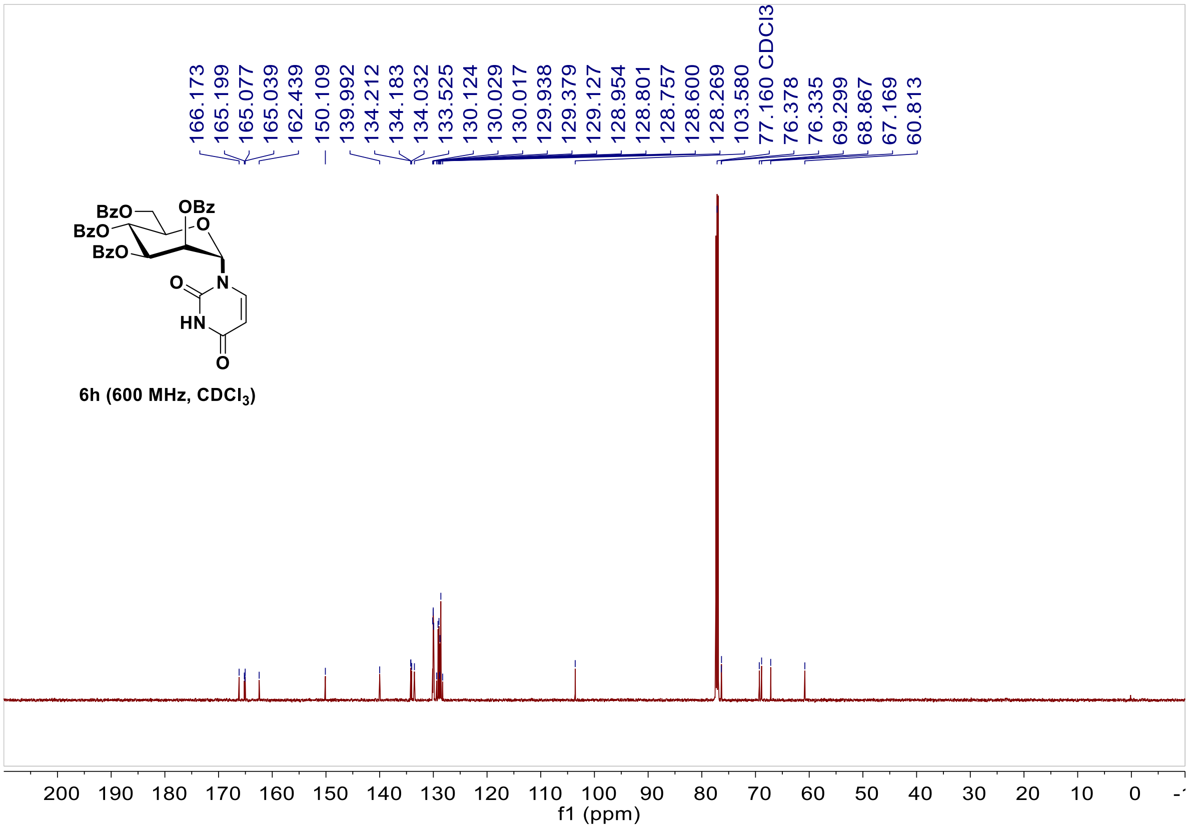
166.173
165.199
165.077
165.039
162.439
150.109
139.992
134.212
134.183
134.032
133.525
130.124
130.029
130.017
129.938
129.379
129.127
128.954
128.801
128.757
128.600
128.269
103.580
77.160 CDCl3
76.378
76.335
69.299
68.867
67.169
60.813
BzO
OBz
BzO
O
BzO
N
O
HN
O
6h (600 MHz, CDCl3)
200 190 180 170 160 150 140 130 120 110 100 90 80 70 60 50 40 30 20 10 0
f1 (ppm)

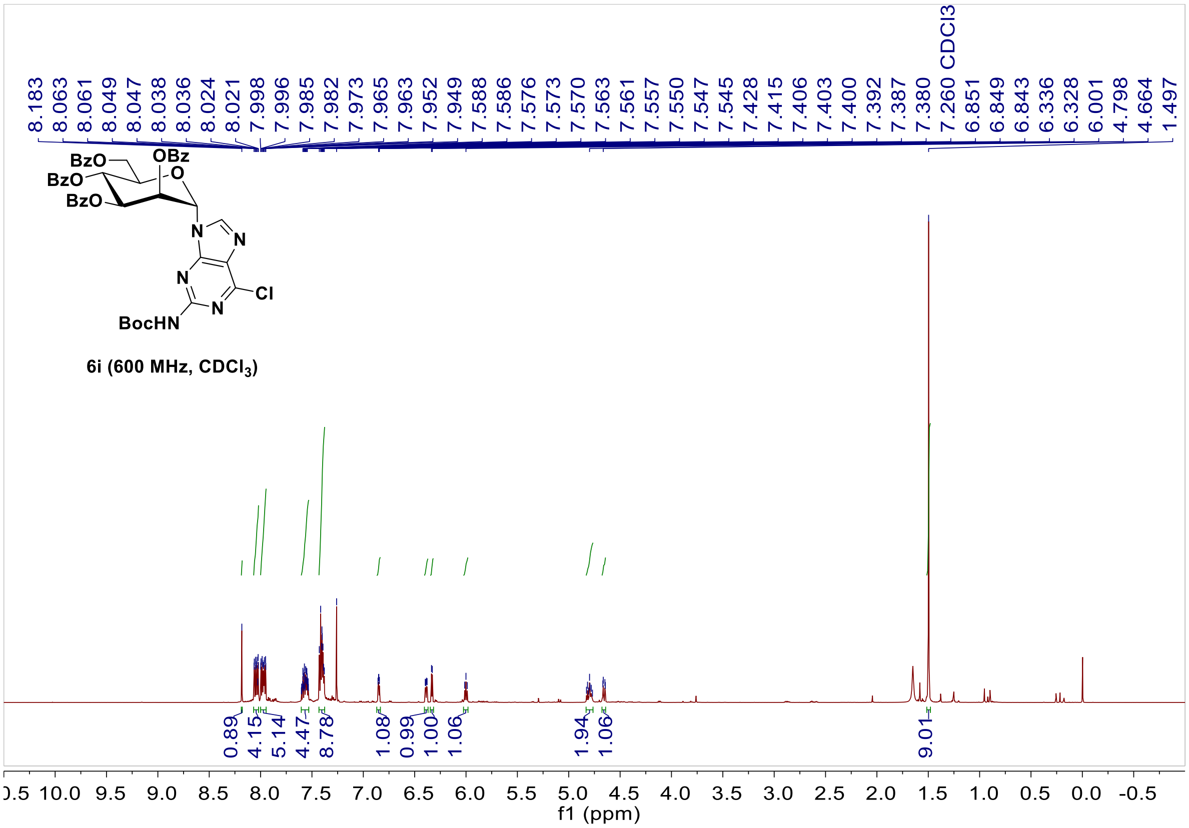
8.183
8.063
8.061
8.049
8.047
8.038
8.036
8.024
8.021
7.998
7.996
7.985
7.982
7.973
7.965
7.963
7.952
7.949
7.588
7.586
7.576
7.573
7.570
7.563
7.561
7.557
7.550
7.547
7.545
7.428
7.415
7.406
7.403
7.400
7.392
7.387
7.380
7.260 CDCl3
6.851
6.849
6.843
6.336
6.328
6.001
4.798
4.664
1.497
BzO
OBz
BzO
O
BzO
N
N
N
Cl
N
BocHN
6i (600 MHz, CDCl3)
0.89
4.15
5.14
4.47
8.78
1.08
0.99
1.00
1.06
1.94
1.06
9.01
10.0 9.5 9.0 8.5 8.0 7.5 7.0 6.5 6.0 5.5 5.0 4.5 4.0 3.5 3.0 2.5 2.0 1.5 1.0 0.5 0.0 -0.5
f1 (ppm)

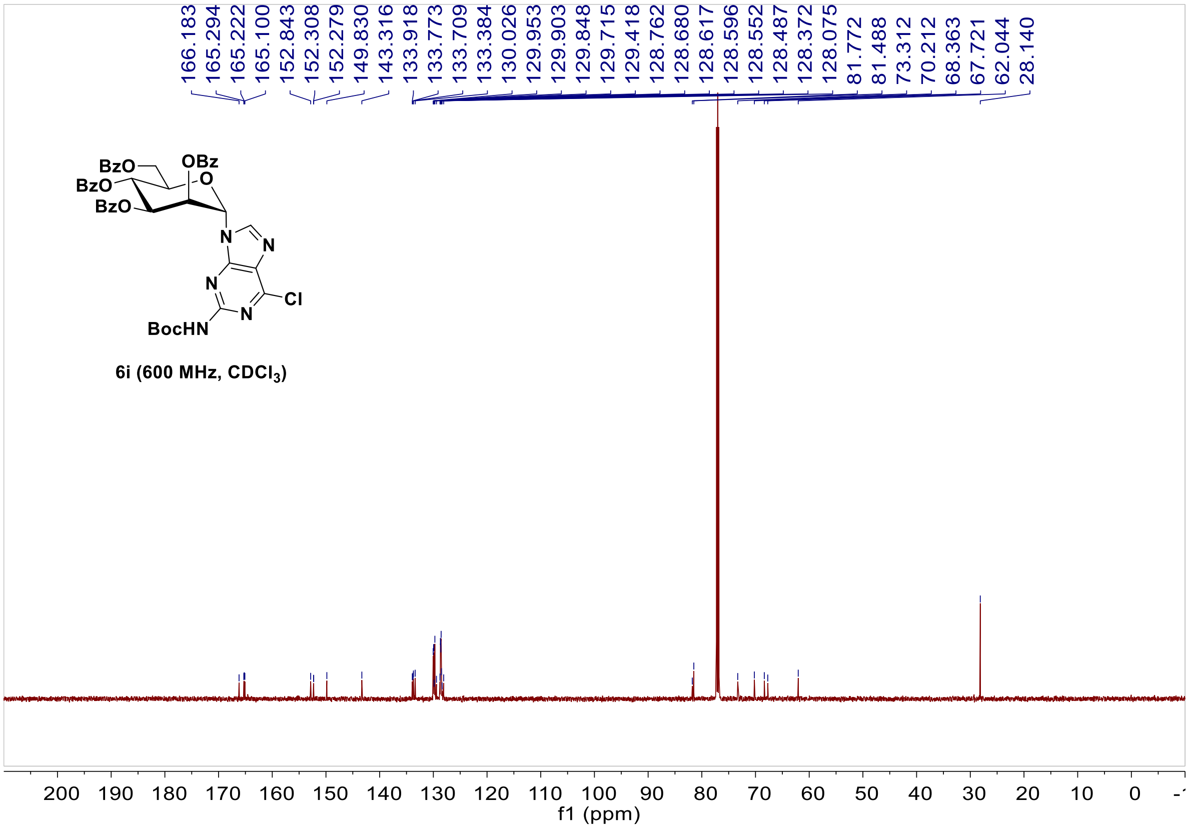
166.183
165.294
165.222
165.100
152.843
152.308
152.279
149.830
143.316
133.918
133.773
133.709
133.384
130.026
129.953
129.903
129.848
129.715
129.418
128.762
128.680
128.617
128.596
128.552
128.487
128.372
128.075
81.772
81.488
73.312
70.212
68.363
67.721
62.044
28.140
BzO
BzO
BzO
OBz
O
N
N
N
N
Cl
BocHN
6i (600 MHz, CDCl3)
200 190 180 170 160 150 140 130 120 110 100 90 80 70 60 50 40 30 20 10 0
f1 (ppm)

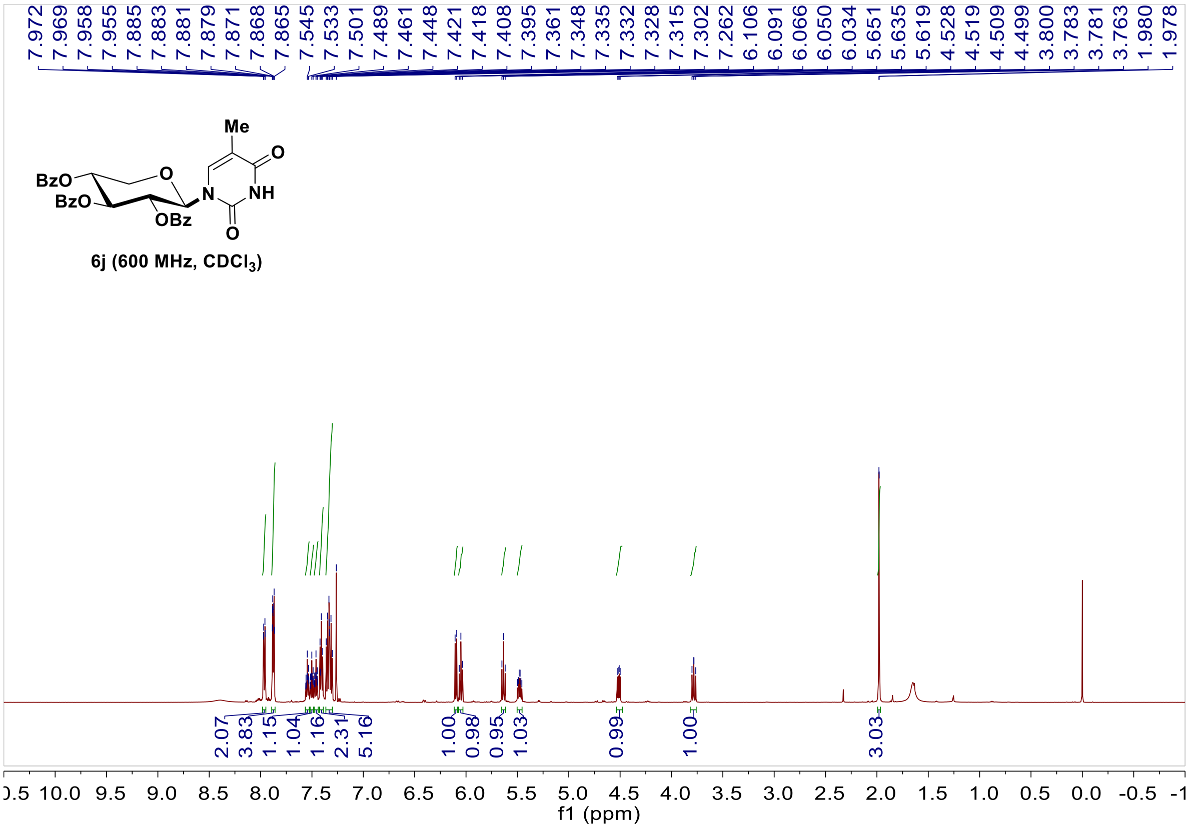
7.972
7.969
7.958
7.955
7.885
7.883
7.881
7.879
7.871
7.868
7.865
7.545
7.533
7.501
7.489
7.461
7.448
7.421
7.418
7.408
7.395
7.361
7.348
7.335
7.332
7.328
7.315
7.302
7.262
6.106
6.091
6.066
6.050
6.034
5.651
5.635
5.619
4.528
4.519
4.509
4.499
3.800
3.783
3.781
3.763
1.980
1.978
Me
O
NH
O
N
O
BzO
BzO
OBz
6j (600 MHz, CDCl3)
2.07
3.83
1.15
1.04
1.16
2.31
5.16
1.00
0.98
0.95
1.03
0.99
1.00
3.03
10.0 9.5 9.0 8.5 8.0 7.5 7.0 6.5 6.0 5.5 5.0 4.5 4.0 3.5 3.0 2.5 2.0 1.5 1.0 0.5 0.0 -0.5
f1 (ppm)

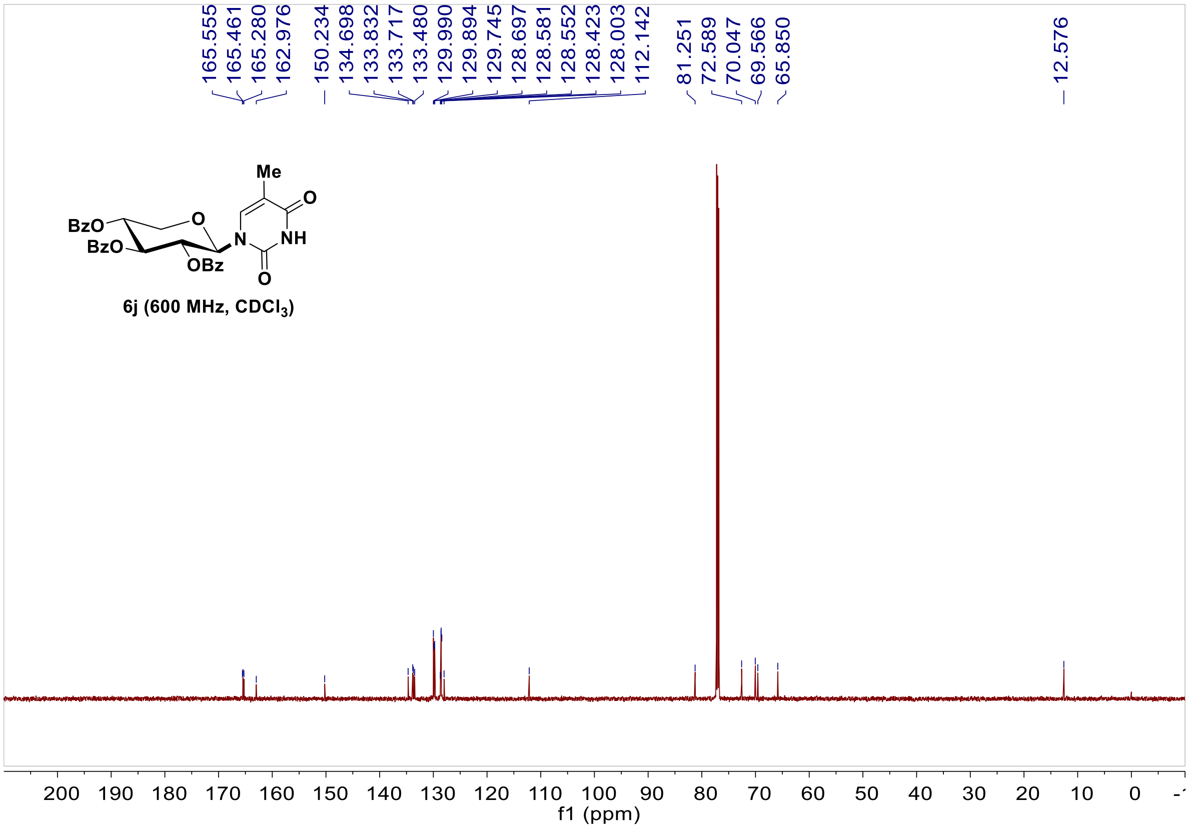
165.555
165.461
165.280
162.976
150.234
134.698
133.832
133.717
133.480
129.990
129.894
129.745
128.697
128.581
128.552
128.423
128.003
112.142
81.251
72.589
70.047
69.566
65.850
12.576
Me
O
BzO
O
N
NH
BzO
OBz
O
6j (600 MHz, CDCl3)
200 190 180 170 160 150 140 130 120 110 100 90 80 70 60 50 40 30 20 10 0
f1 (ppm)

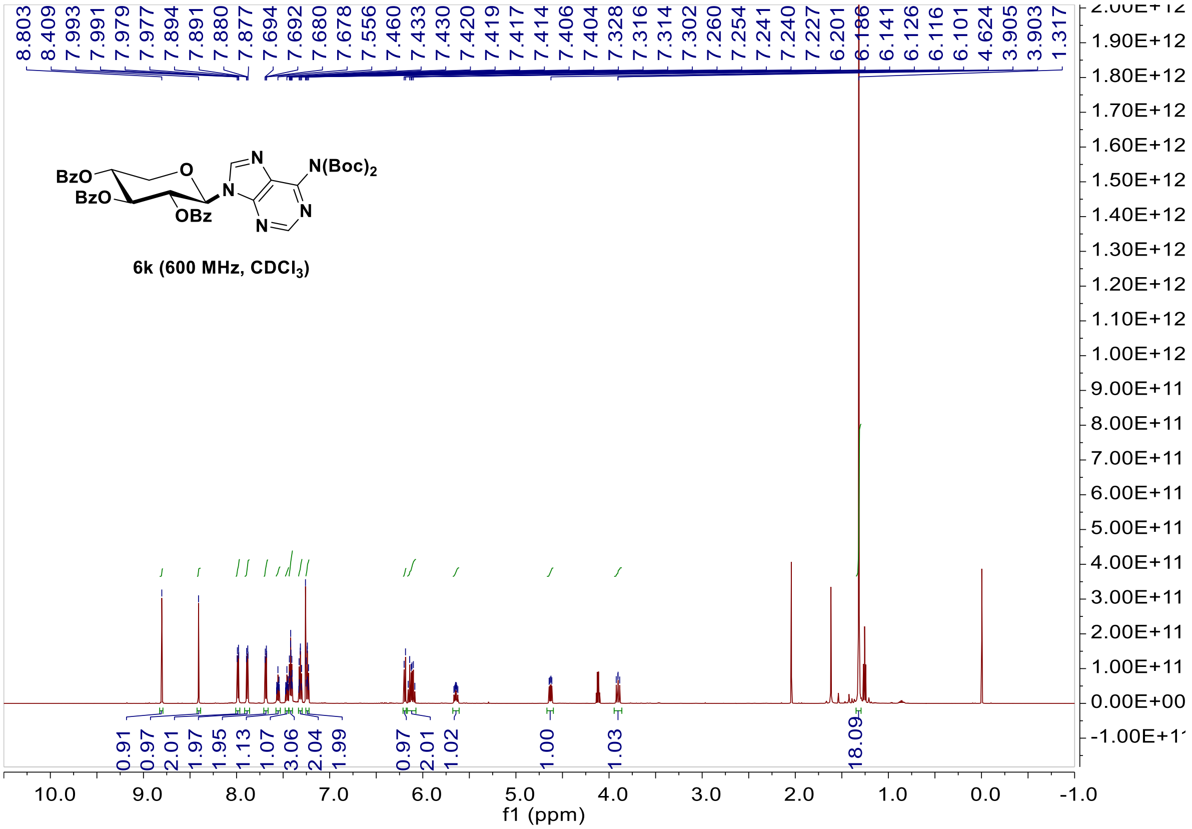
8.803
8.409
7.993
7.991
7.979
7.977
7.894
7.891
7.880
7.877
7.694
7.692
7.680
7.678
7.556
7.460
7.433
7.430
7.420
7.419
7.417
7.414
7.406
7.404
7.328
7.316
7.314
7.302
7.260
7.254
7.241
7.240
7.227
6.201
6.186
6.141
6.126
6.116
6.101
4.624
3.905
3.903
1.317
BzO
O
N
N(Boc)2
BzO
N
OBz
N
N
6k (600 MHz, CDCl3)
0.91
0.97
2.01
1.97
1.95
1.13
1.07
3.06
2.04
1.99
0.97
2.01
1.02
1.00
1.03
18.09
1.90E+12
1.80E+12
1.70E+12
1.60E+12
1.50E+12
1.40E+12
1.30E+12
1.20E+12
1.10E+12
1.00E+12
9.00E+11
8.00E+11
7.00E+11
6.00E+11
5.00E+11
4.00E+11
3.00E+11
2.00E+11
1.00E+11
0.00E+00
10.0 9.0 8.0 7.0 6.0 5.0 4.0 3.0 2.0 1.0 0.0 -1.0
f1 (ppm)

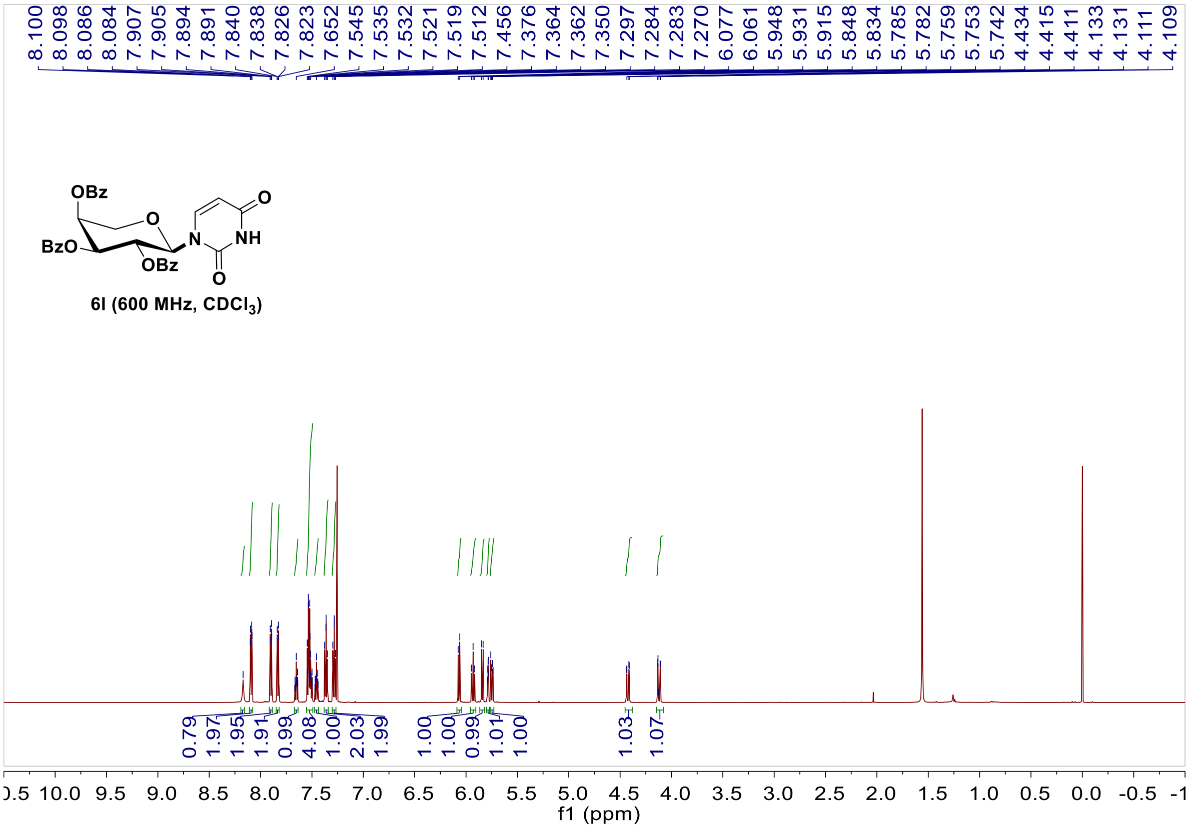
8.100 8.098 8.086 8.084 7.907 7.905 7.894 7.891 7.840 7.838 7.826 7.823 7.652 7.545 7.535 7.532 7.521 7.519 7.512 7.456 7.376 7.364 7.362 7.350 7.297 7.284 7.283 7.270 6.077 6.061 5.948 5.931 5.915 5.848 5.834 5.785 5.782 5.759 5.753 5.742 4.434 4.415 4.411 4.133 4.131 4.111 4.109
OBz
BzO
OBz
O
N
NH
O
O
6l (600 MHz, $CDCl_3$)
0.79 1.97 1.95 1.91 0.99 4.08 1.00 2.03 1.99 1.00 1.00 0.99 1.01 1.00 1.03 1.07
f1 (ppm)

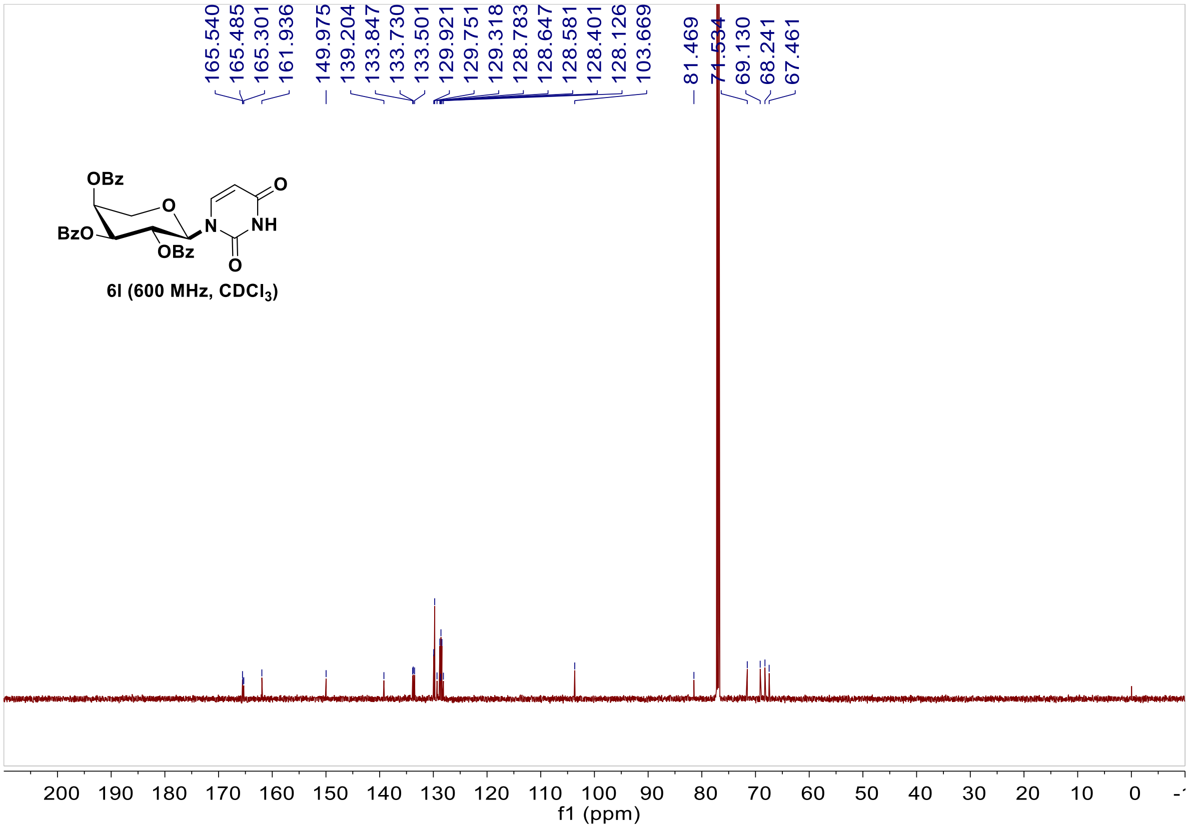
165.540 165.485 165.301 161.936 149.975 139.204 133.847 133.730 133.501 129.921 129.751 129.318 128.783 128.647 128.581 128.401 128.126 103.669 81.469 71.534 69.130 68.241 67.461
OBz
BzO
OBz
O
N
NH
O
O
6l (600 MHz, $CDCl_3$)
f1 (ppm)

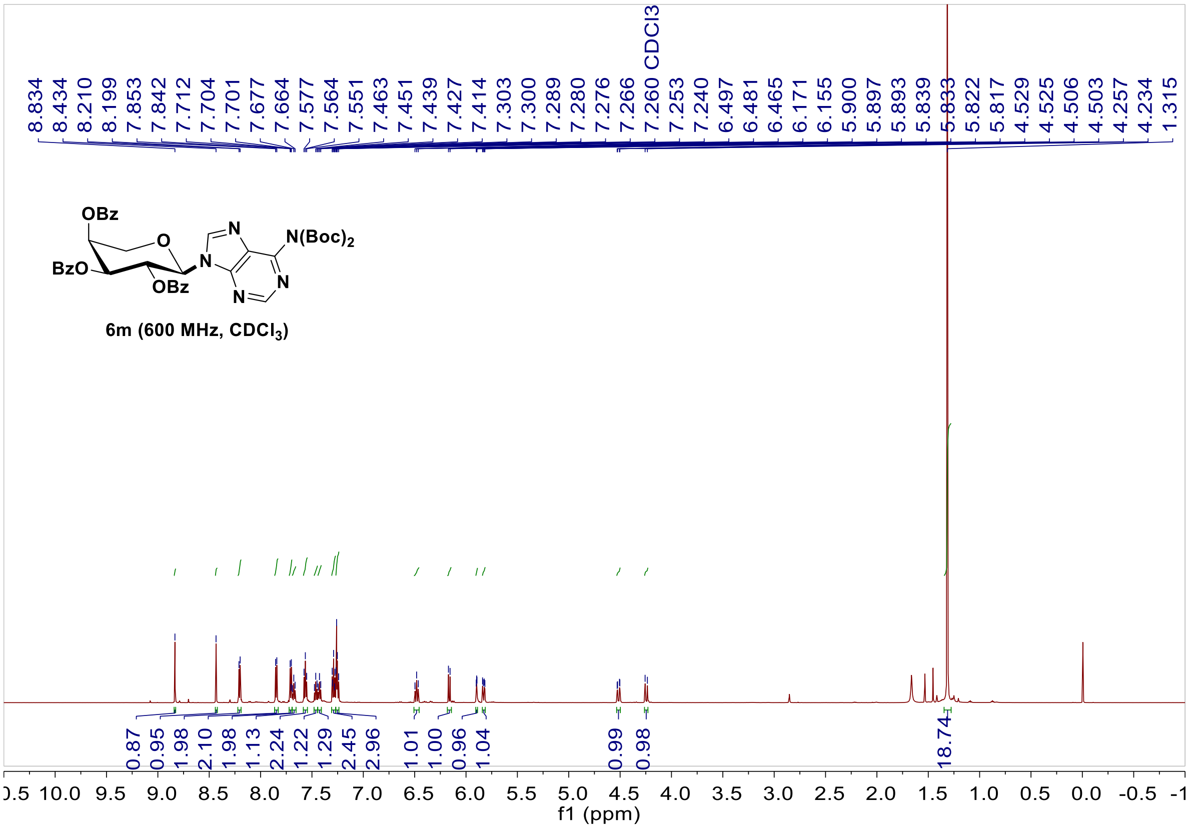
8.834 8.434 8.210 8.199 7.853 7.842 7.712 7.704 7.701 7.677 7.664 7.577 7.564 7.551 7.463 7.451 7.439 7.427 7.414 7.303 7.300 7.289 7.280 7.276 7.266 7.260 CDCl3 7.253 7.240 6.497 6.481 6.465 6.171 6.155 5.900 5.897 5.893 5.839 5.833 5.822 5.817 4.529 4.525 4.506 4.503 4.257 4.234 1.315
OBz
BzO
OBz
O
N
N
N
N
N(Boc)2
6m (600 MHz, CDCl3)
0.87 0.95 1.98 2.10 1.98 1.13 2.24 1.22 1.29 2.45 2.96 1.01 1.00 0.96 1.04 0.99 0.98 18.74
10.0 9.5 9.0 8.5 8.0 7.5 7.0 6.5 6.0 5.5 5.0 4.5 4.0 3.5 3.0 2.5 2.0 1.5 1.0 0.5 0.0 -0.5
f1 (ppm)

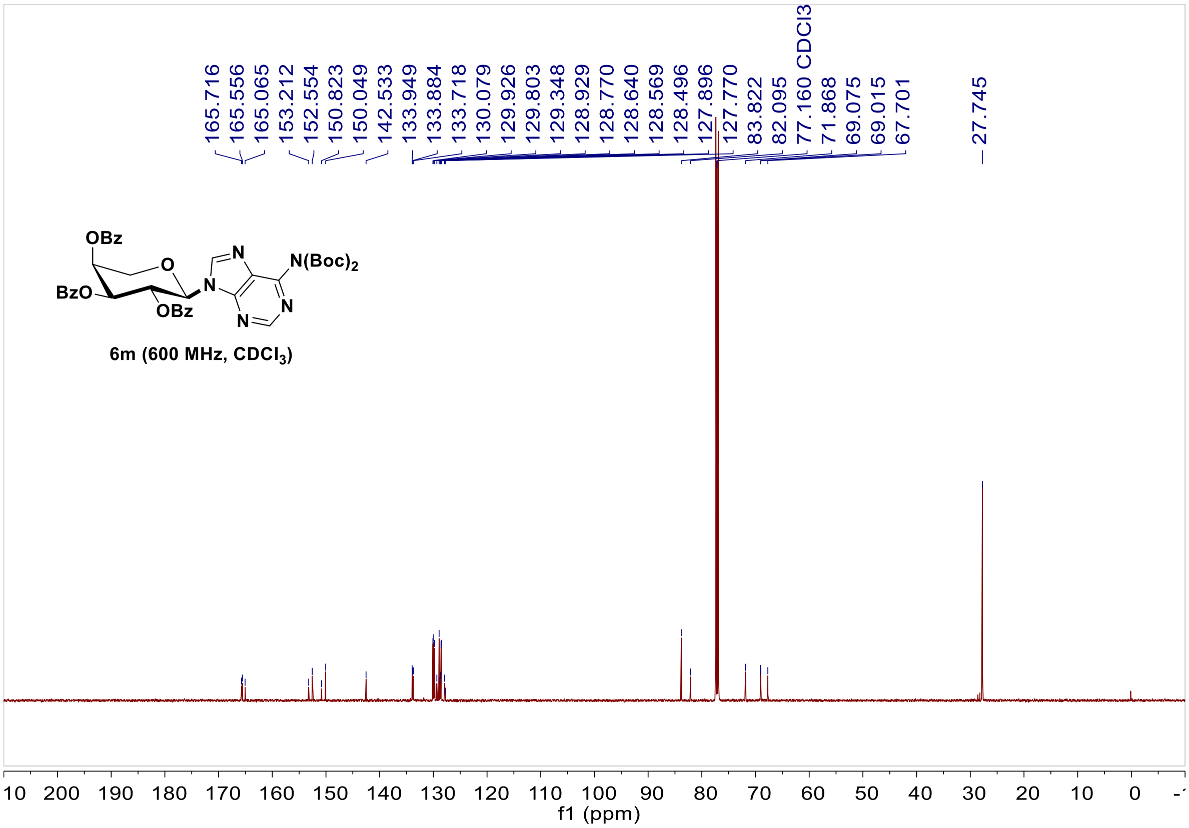
165.716 165.556 165.065 153.212 152.554 150.823 150.049 142.533 133.949 133.884 133.718 130.079 129.926 129.803 129.348 128.929 128.770 128.640 128.569 128.496 127.896 127.770 83.822 82.095 77.160 CDCl3 71.868 69.075 69.015 67.701 27.745
OBz
BzO
OBz
O
N
N
N
N
N(Boc)2
6m (600 MHz, CDCl3)
200 190 180 170 160 150 140 130 120 110 100 90 80 70 60 50 40 30 20 10 0
f1 (ppm)

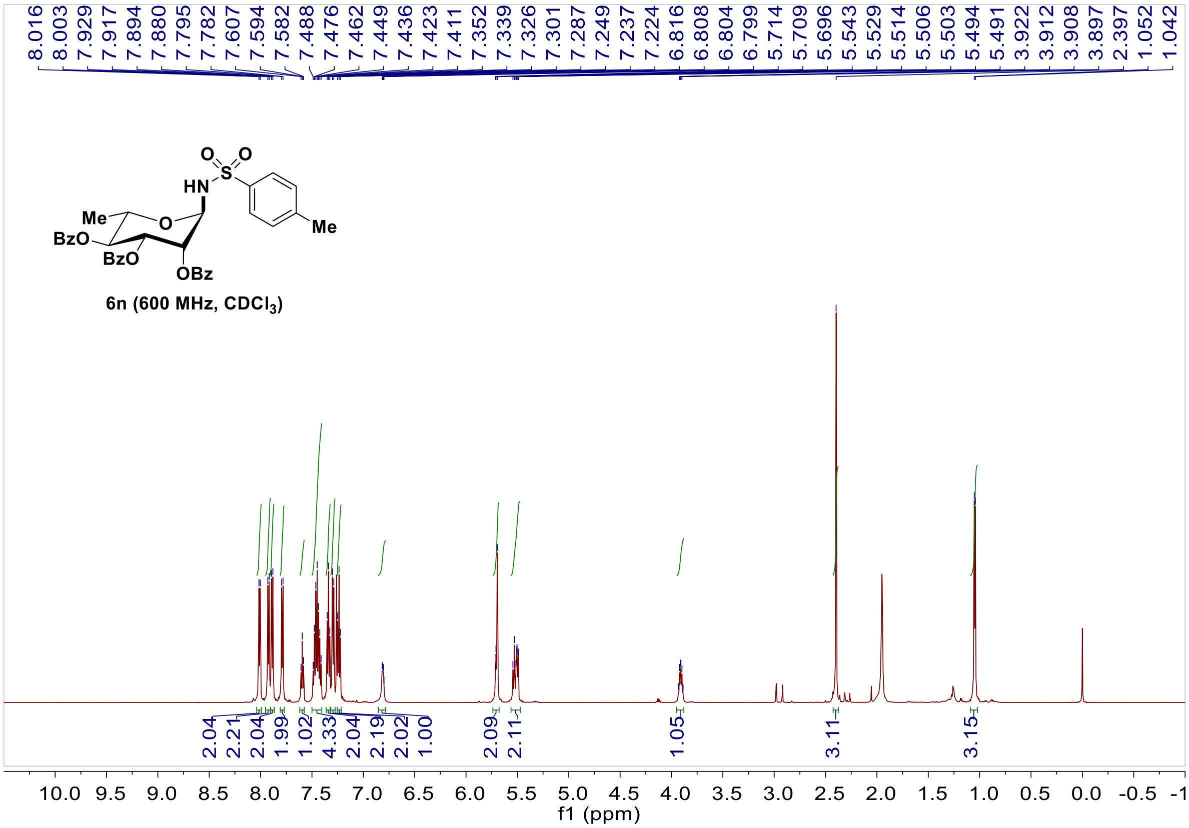
8.016 8.003 7.929 7.917 7.894 7.880 7.795 7.782 7.607 7.594 7.582 7.488 7.476 7.462 7.449 7.436 7.423 7.411 7.352 7.339 7.326 7.301 7.287 7.249 7.237 7.224 6.816 6.808 6.804 6.799 5.714 5.709 5.696 5.543 5.529 5.514 5.506 5.503 5.494 5.491 3.922 3.912 3.908 3.897 2.397 1.052 1.042
Me
BzO
BzO
OBz
HN
O
S
O O
Me
6n (600 MHz, CDCl3)
2.04 2.21 2.04 1.99 1.02 4.33 2.04 2.19 2.02 1.00 2.09 2.11 1.05 3.11 3.15
10.0 9.5 9.0 8.5 8.0 7.5 7.0 6.5 6.0 5.5 5.0 4.5 4.0 3.5 3.0 2.5 2.0 1.5 1.0 0.5 0.0 -0.5 -1
f1 (ppm)

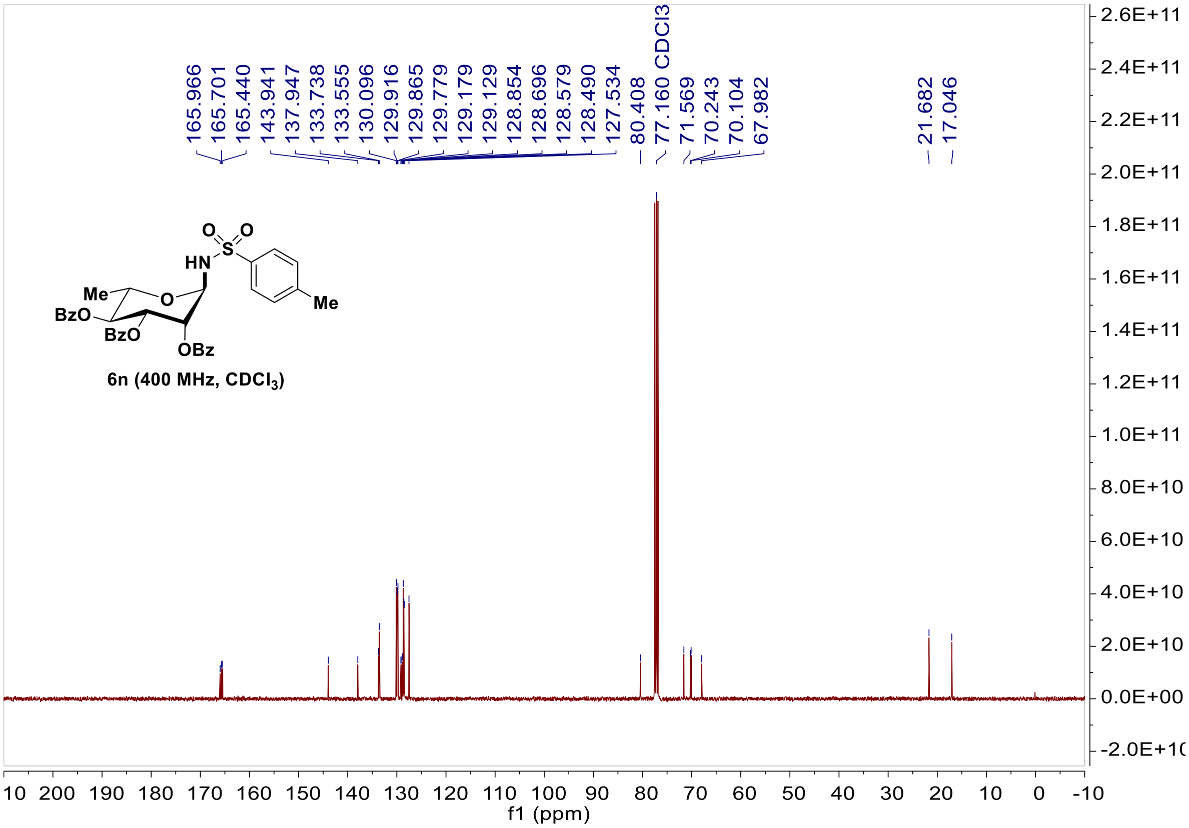
165.966 165.701 165.440 143.941 137.947 133.738 133.555 130.096 129.916 129.865 129.779 129.179 129.129 128.854 128.696 128.579 128.490 127.534 80.408 77.160 CDCl3 71.569 70.243 70.104 67.982 21.682 17.046
Me
BzO
BzO
OBz
HN
O
S
O O
Me
6n (400 MHz, CDCl3)
2.6E+11 2.4E+11 2.2E+11 2.0E+11 1.8E+11 1.6E+11 1.4E+11 1.2E+11 1.0E+11 8.0E+10 6.0E+10 4.0E+10 2.0E+10 0.0E+00 -2.0E+10
10 200 190 180 170 160 150 140 130 120 110 100 90 80 70 60 50 40 30 20 10 0 -10
f1 (ppm)

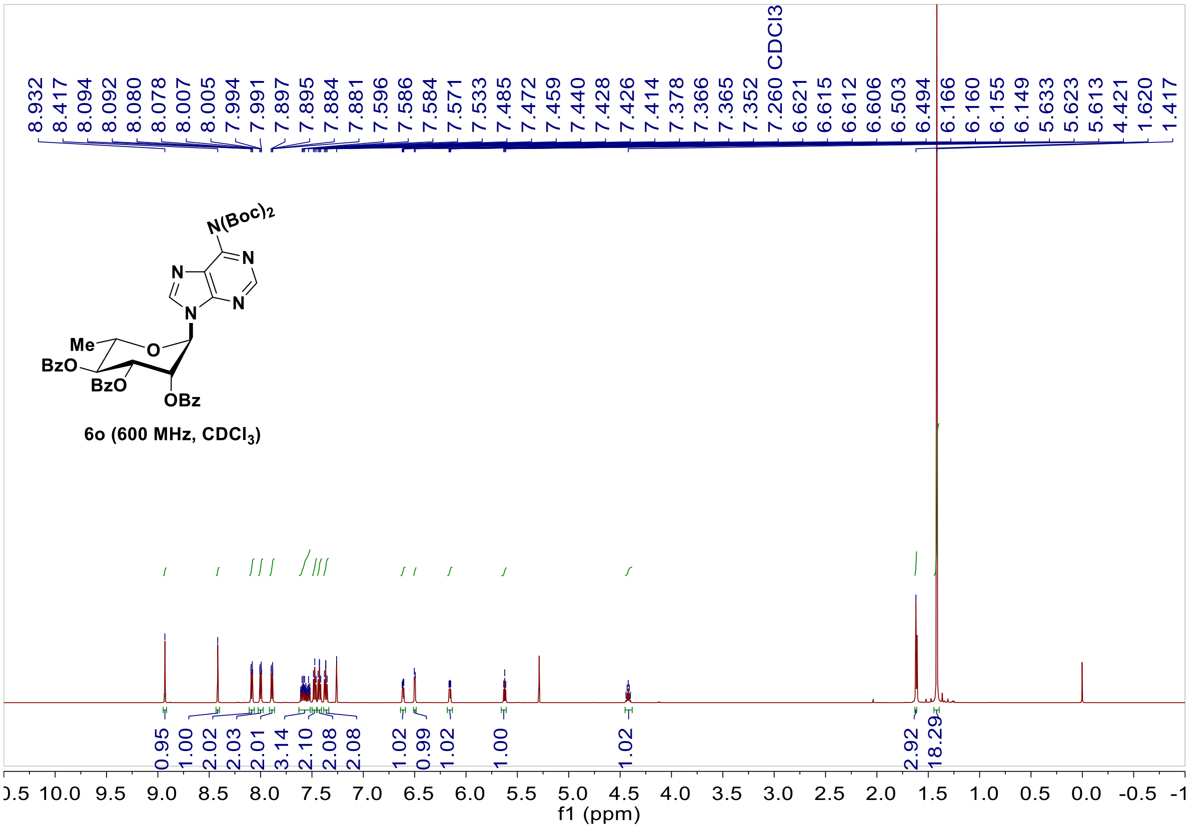
8.932 8.417 8.094 8.092 8.080 8.078 8.007 8.005 7.994 7.991 7.897 7.895 7.884 7.881 7.596 7.586 7.584 7.571 7.533 7.485 7.472 7.459 7.440 7.428 7.426 7.414 7.378 7.366 7.365 7.352 7.260 CDCl3 6.621 6.615 6.612 6.606 6.503 6.494 6.166 6.160 6.155 6.149 5.633 5.623 5.613 4.421 1.620 1.417
N(Boc)2
Me
BzO
BzO
OBz
6o (600 MHz, CDCl3)
0.95 1.00 2.02 2.03 2.01 3.14 2.10 2.08 2.08 1.02 0.99 1.02 1.00 1.02 2.92 18.29
f1 (ppm)

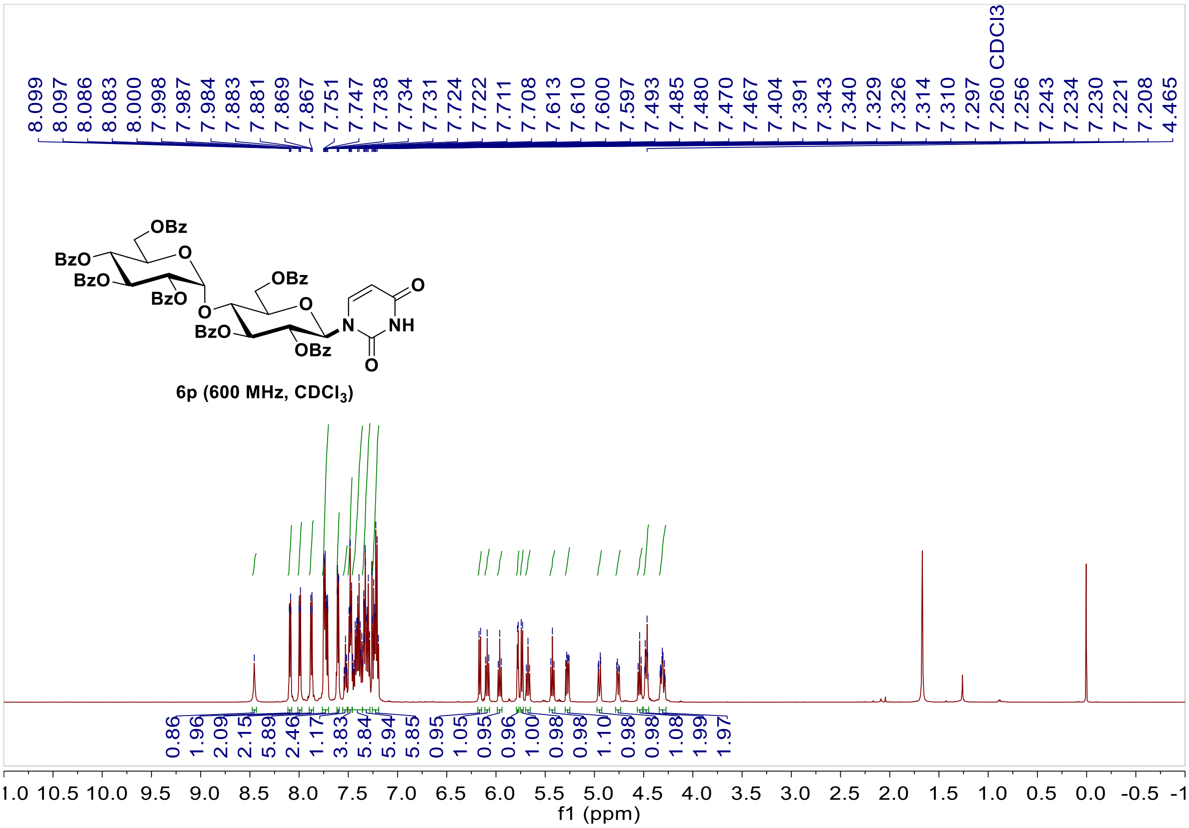
8.099 8.097 8.086 8.083 8.000 7.998 7.987 7.984 7.883 7.881 7.869 7.867 7.751 7.747 7.738 7.734 7.731 7.724 7.722 7.711 7.708 7.613 7.610 7.600 7.597 7.493 7.485 7.480 7.470 7.467 7.404 7.391 7.343 7.340 7.329 7.326 7.314 7.310 7.297 7.260 CDCl3 7.256 7.243 7.234 7.230 7.221 7.208 4.465
OBz
BzO
BzO
BzO
OBz
BzO
OBz
NH
6p (600 MHz, CDCl3)
0.86 1.96 2.09 2.15 5.89 2.46 1.17 3.83 5.84 5.94 5.85 0.95 1.05 0.95 0.96 1.00 0.98 0.98 1.10 0.98 0.98 1.08 1.99 1.97
f1 (ppm)

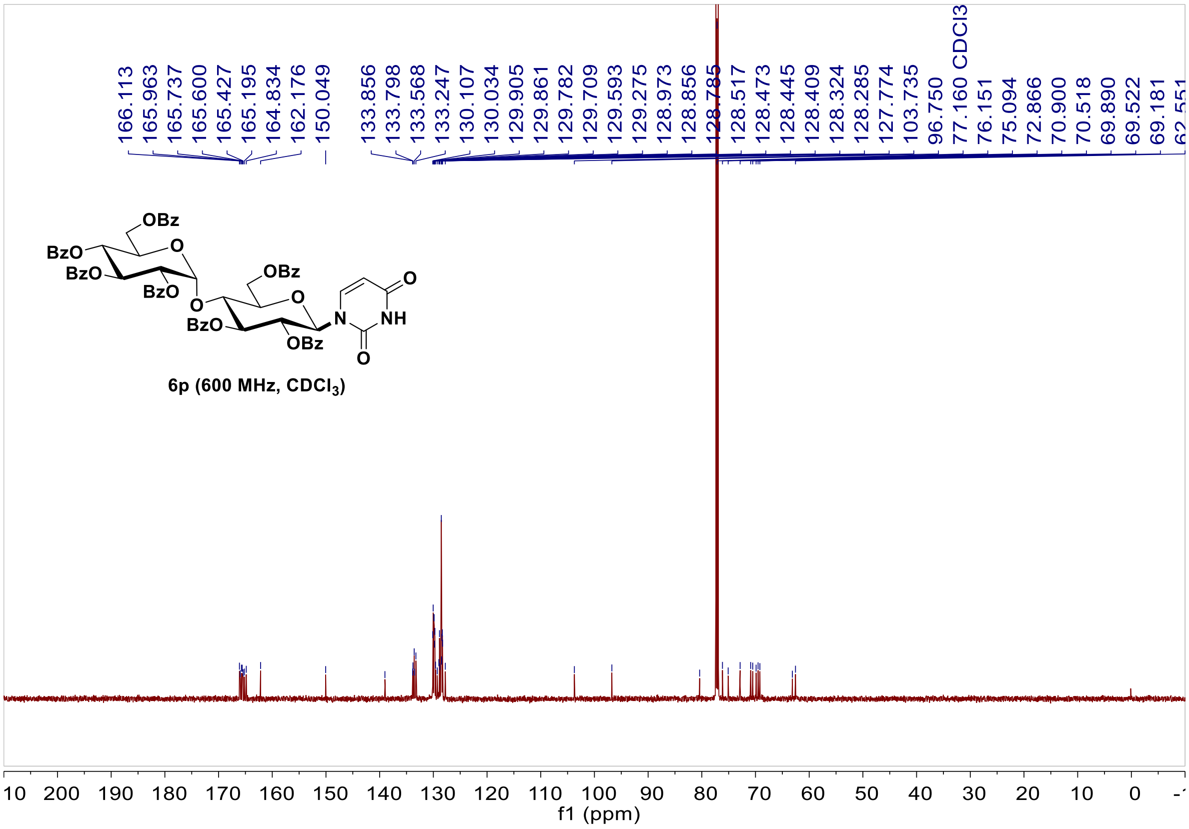
166.113
165.963
165.737
165.600
165.427
165.195
164.834
162.176
150.049
133.856
133.798
133.568
133.247
130.107
130.034
129.905
129.861
129.782
129.709
129.593
129.275
128.973
128.856
128.785
128.517
128.473
128.445
128.409
128.324
128.285
127.774
103.735
96.750
77.160 CDCl3
76.151
75.094
72.866
70.900
70.518
69.890
69.522
69.181
6p (600 MHz, $CDCl_3$)
f1 (ppm)

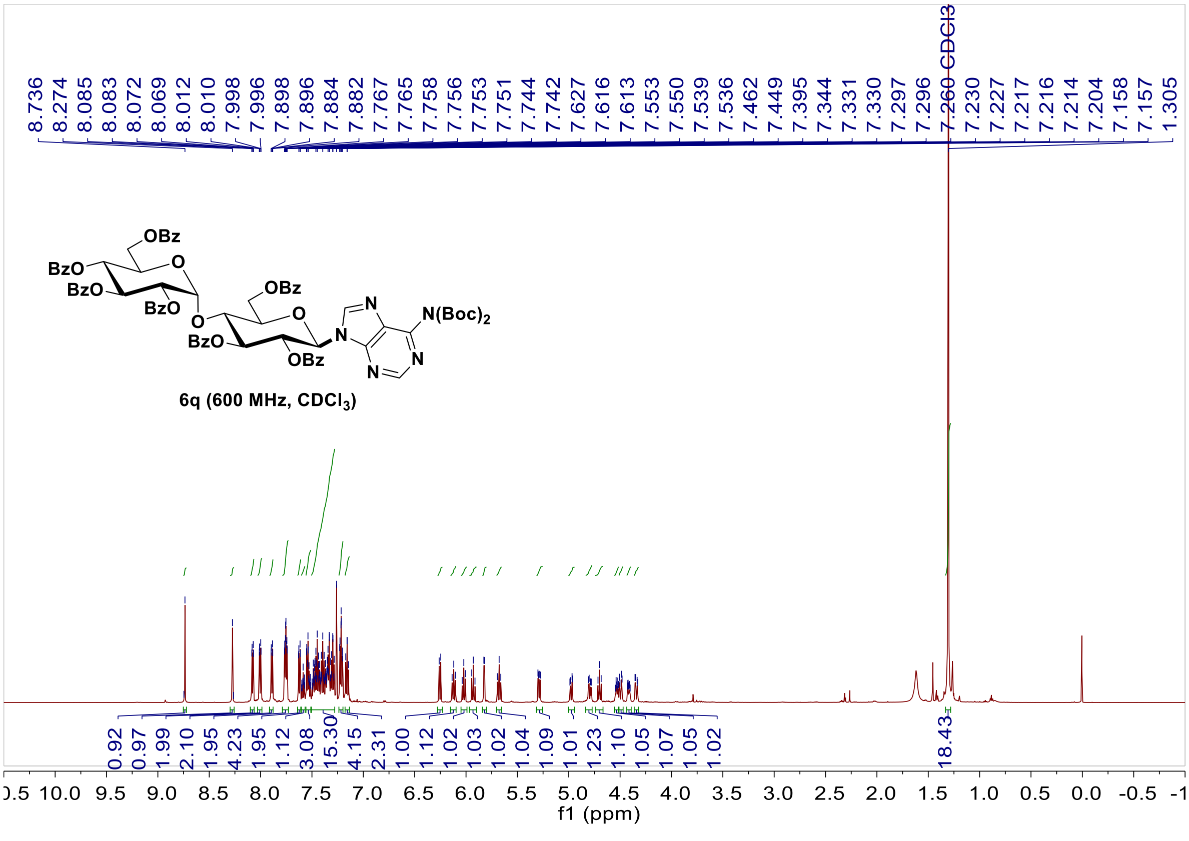
8.736
8.274
8.085
8.083
8.072
8.069
8.012
8.010
7.998
7.996
7.898
7.896
7.884
7.882
7.767
7.765
7.758
7.756
7.753
7.751
7.744
7.742
7.627
7.616
7.613
7.553
7.550
7.539
7.536
7.462
7.449
7.395
7.344
7.331
7.330
7.297
7.296
7.260 CDCl3
7.230
7.227
7.217
7.216
7.214
7.204
7.158
7.157
1.305
6q (600 MHz, $CDCl_3$)
0.92
0.97
1.99
2.10
1.95
4.23
1.95
1.12
3.08
15.30
4.15
2.31
1.00
1.12
1.02
1.03
1.02
1.04
1.09
1.01
1.23
1.10
1.05
1.07
1.05
1.02
18.43
f1 (ppm)

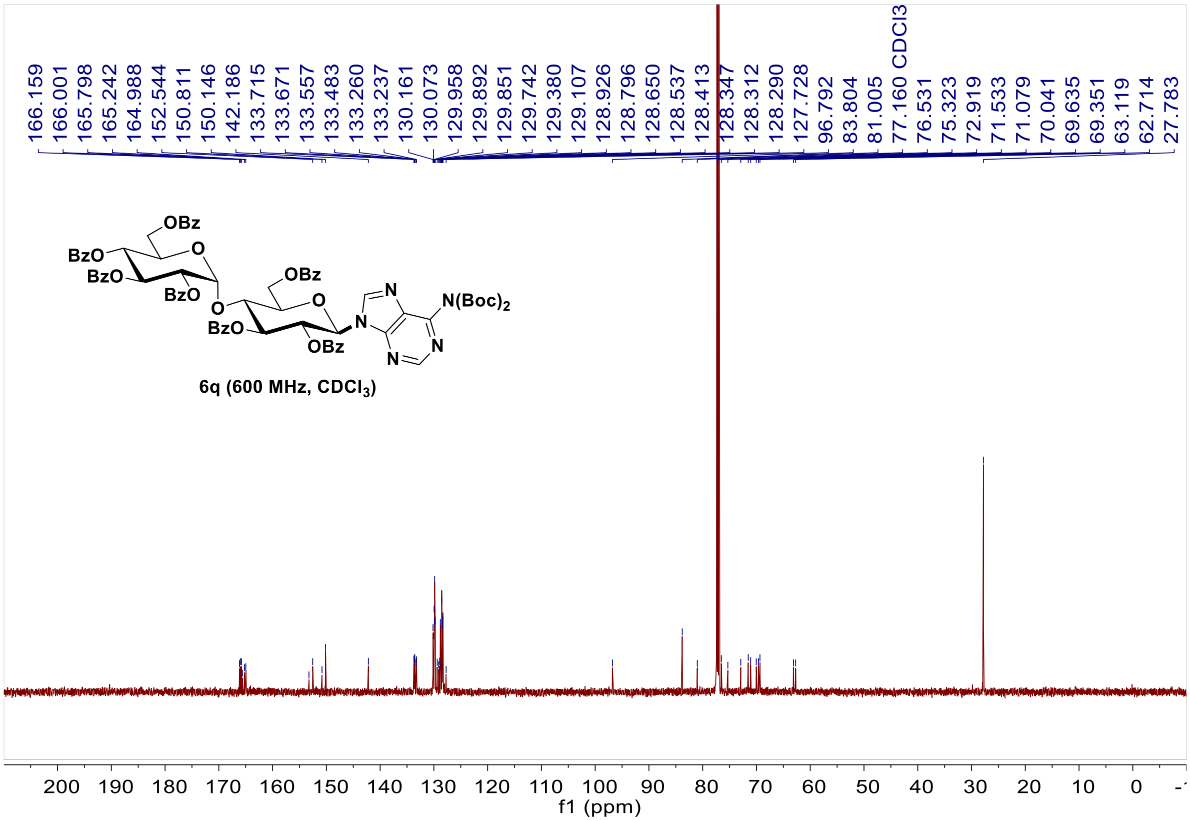
166.159
166.001
165.798
165.242
164.988
152.544
150.811
150.146
142.186
133.715
133.671
133.557
133.483
133.260
133.237
130.161
130.073
129.958
129.892
129.851
129.742
129.380
129.107
128.926
128.796
128.650
128.537
128.413
128.347
128.312
128.290
127.728
96.792
83.804
81.005
77.160 CDCl3
76.531
75.323
72.919
71.533
71.079
70.041
69.635
69.351
63.119
62.714
27.783
OBz
BzO
O
BzO
BzO
OBz
O
N
N(Boc)2
BzO
N
OBz
N
N
6q (600 MHz, CDCl3)
200 190 180 170 160 150 140 130 120 110 100 90 80 70 60 50 40 30 20 10 0
f1 (ppm)

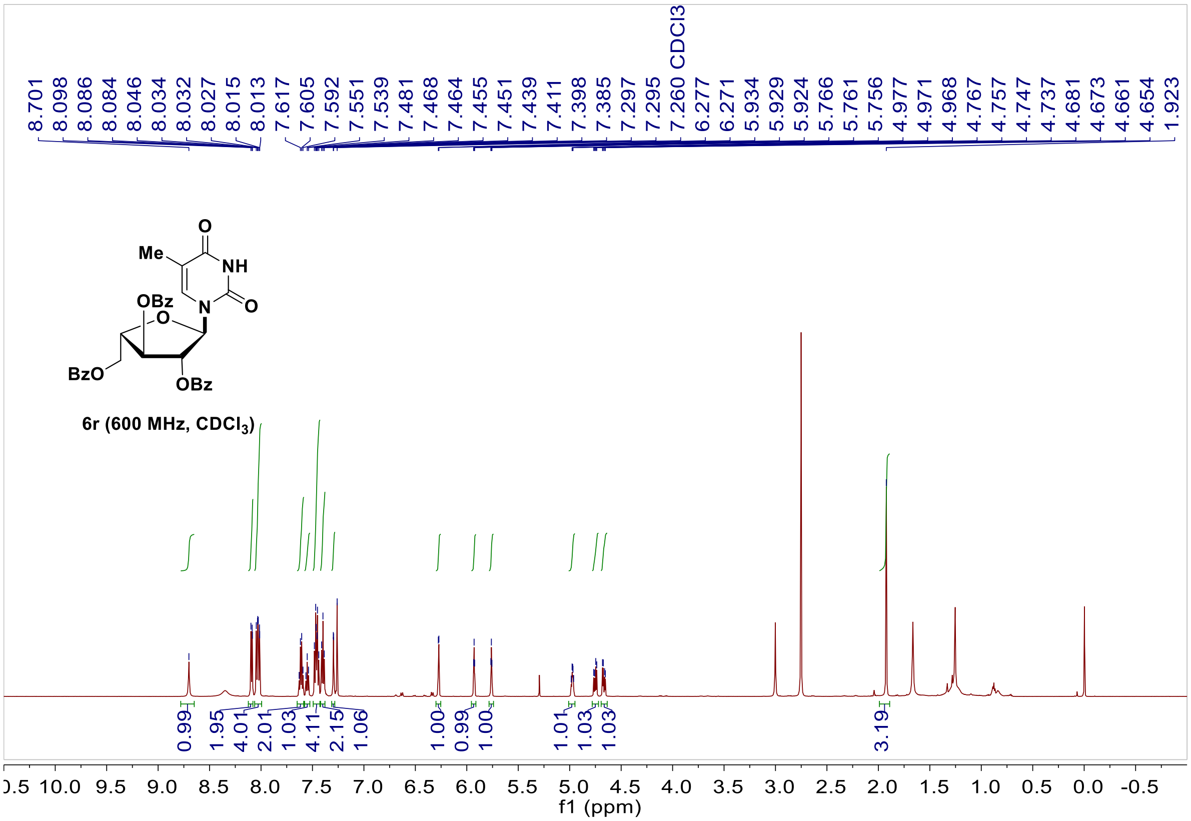
8.701
8.098
8.086
8.084
8.046
8.034
8.032
8.027
8.015
8.013
7.617
7.605
7.592
7.551
7.539
7.481
7.468
7.464
7.455
7.451
7.439
7.411
7.398
7.385
7.297
7.295
7.260 CDCl3
6.277
6.271
5.934
5.929
5.924
5.766
5.761
5.756
4.977
4.971
4.968
4.767
4.757
4.747
4.737
4.681
4.673
4.661
4.654
1.923
O
Me
NH
OBz
O
N
O
BzO
OBz
6r (600 MHz, CDCl3)
0.99
1.95
4.01
2.01
1.03
4.11
2.15
1.06
1.00
0.99
1.00
1.01
1.03
1.03
3.19
10.0 9.5 9.0 8.5 8.0 7.5 7.0 6.5 6.0 5.5 5.0 4.5 4.0 3.5 3.0 2.5 2.0 1.5 1.0 0.5 0.0 -0.5
f1 (ppm)

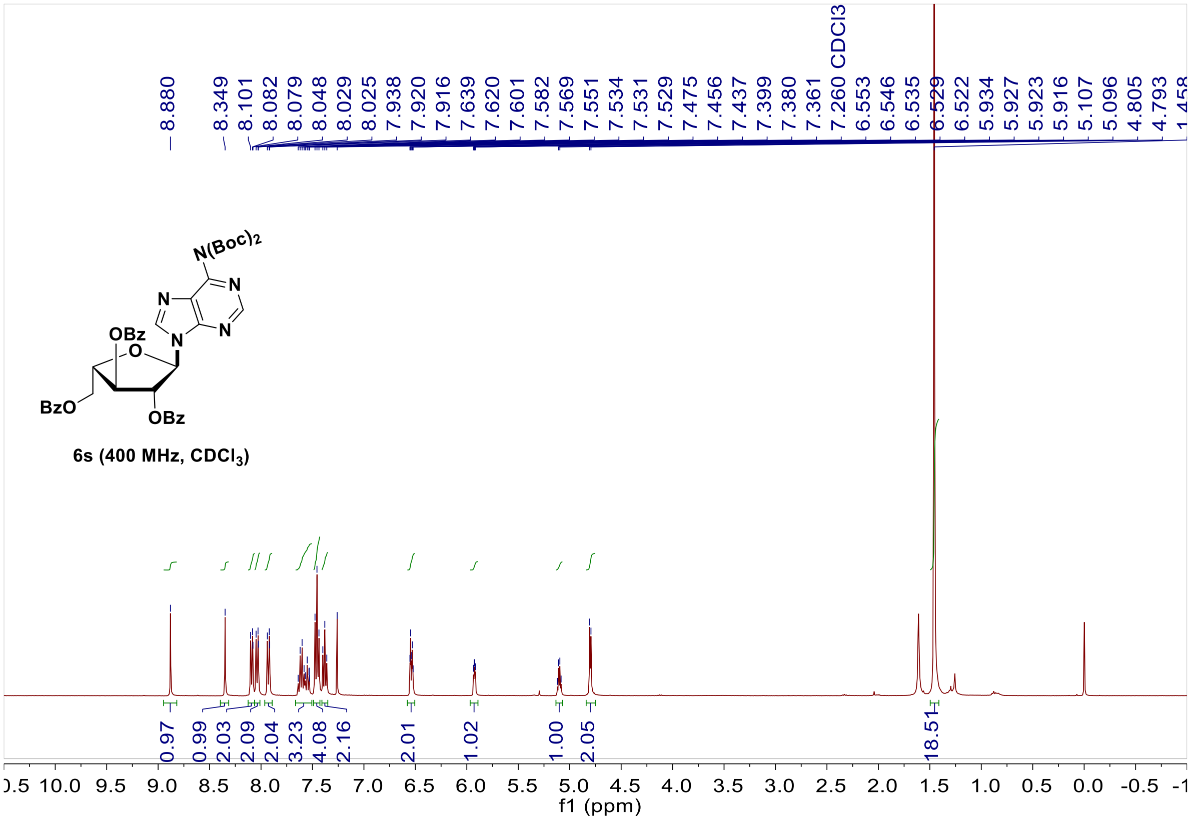
8.880
8.349
8.101
8.082
8.079
8.048
8.029
8.025
7.938
7.920
7.916
7.639
7.620
7.601
7.582
7.569
7.551
7.534
7.531
7.529
7.475
7.456
7.437
7.399
7.380
7.361
7.260 CDCl3
6.553
6.546
6.535
6.529
6.522
5.934
5.927
5.923
5.916
5.107
5.096
4.805
4.793
N(Boc)2
N
N
N
N
OBz
O
BzO
OBz
6s (400 MHz, CDCl3)
0.97
0.99
2.03
2.09
2.04
3.23
4.08
2.16
2.01
1.02
1.00
2.05
18.51
10.0 9.5 9.0 8.5 8.0 7.5 7.0 6.5 6.0 5.5 5.0 4.5 4.0 3.5 3.0 2.5 2.0 1.5 1.0 0.5 0.0 -0.5
f1 (ppm)

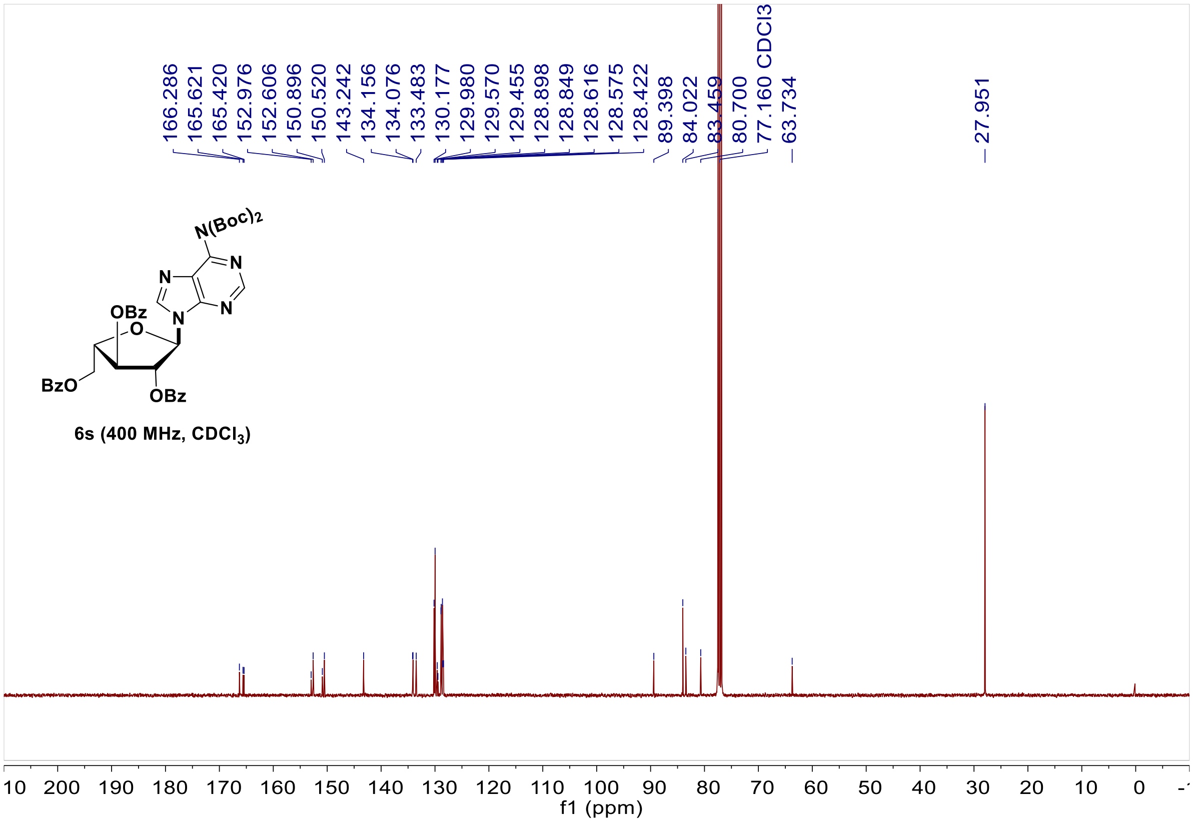
166.286
165.621
165.420
152.976
152.606
150.896
150.520
143.242
134.156
134.076
133.483
130.177
129.980
129.570
129.455
128.898
128.849
128.616
128.575
128.422
89.398
84.022
83.459
80.700
77.160 CDCl3
63.734
27.951
N(Boc)2
N
N
N
N
OBz
O
BzO
OBz
6s (400 MHz, CDCl3)
200 190 180 170 160 150 140 130 120 110 100 90 80 70 60 50 40 30 20 10 0
f1 (ppm)

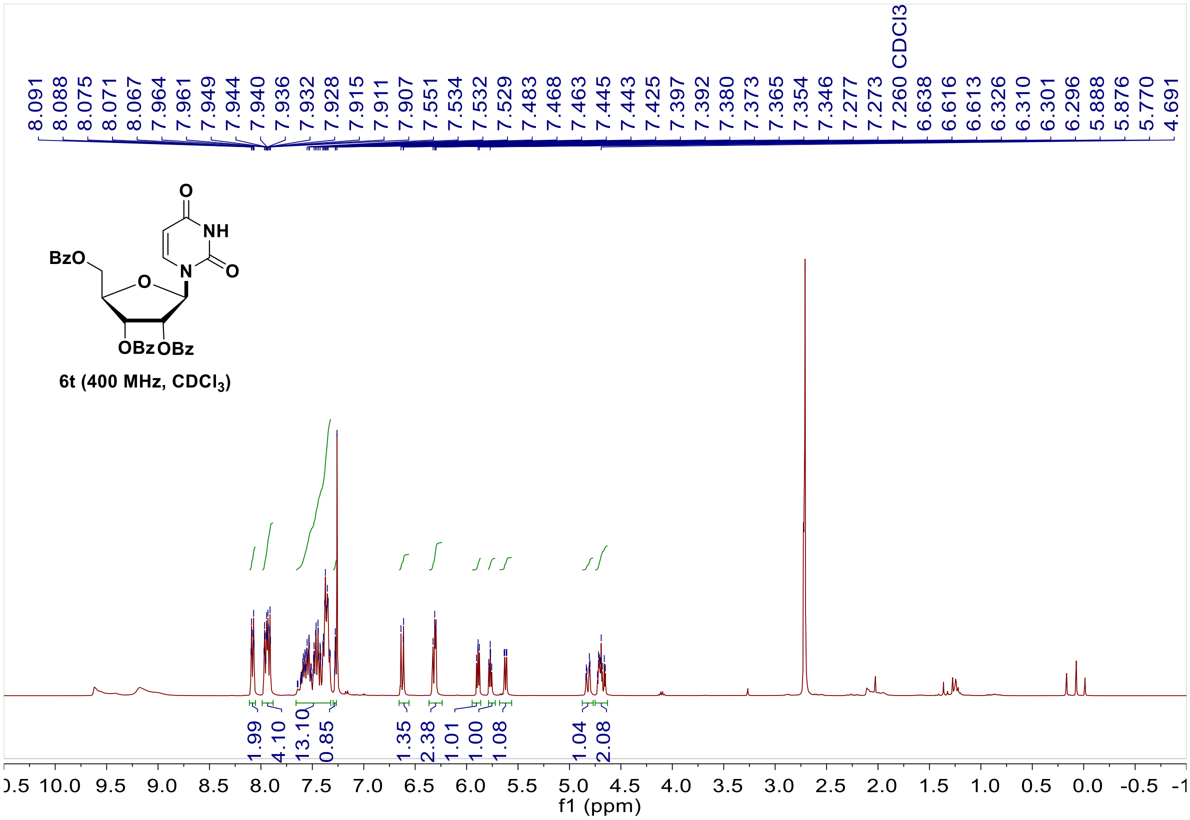
8.091 8.088 8.075 8.071 8.067 7.964 7.961 7.949 7.944 7.940 7.936 7.932 7.928 7.915 7.911 7.907 7.551 7.534 7.532 7.529 7.483 7.468 7.463 7.445 7.443 7.425 7.397 7.392 7.380 7.373 7.365 7.354 7.346 7.277 7.273 7.260 CDCl3 6.638 6.616 6.613 6.326 6.310 6.301 6.296 5.888 5.876 5.770 4.691
O
NH
O
N
BzO
O
OBz OBz
6t (400 MHz, CDCl3)
1.99 4.10 13.10 0.85 1.35 2.38 1.01 1.00 1.08 1.04 2.08
f1 (ppm)

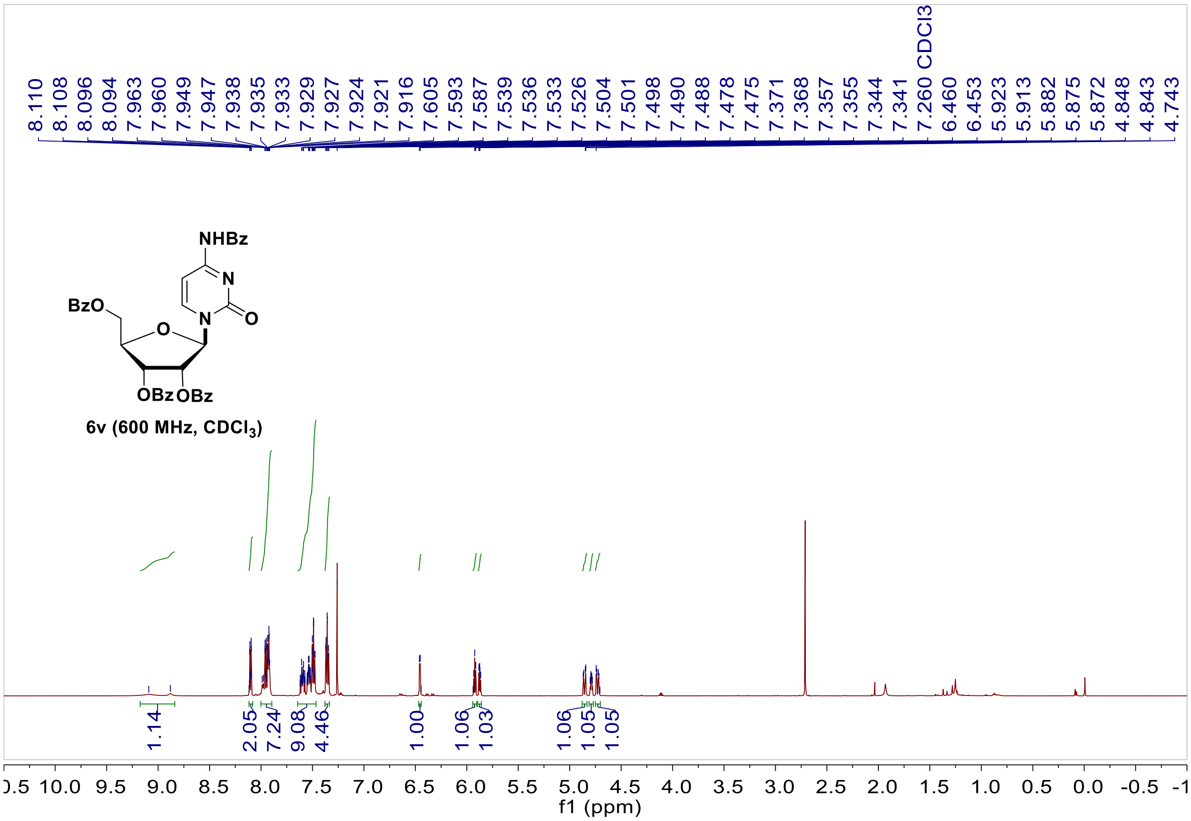
8.110 8.108 8.096 8.094 7.963 7.960 7.949 7.947 7.938 7.935 7.933 7.929 7.927 7.924 7.921 7.916 7.605 7.593 7.587 7.539 7.536 7.533 7.526 7.504 7.501 7.498 7.490 7.488 7.478 7.475 7.371 7.368 7.357 7.355 7.344 7.341 7.260 CDCl3 6.460 6.453 5.923 5.913 5.882 5.875 5.872 4.848 4.843 4.743
NHBz
N
O
N
BzO
O
OBz OBz
6v (600 MHz, CDCl3)
1.14 2.05 7.24 9.08 4.46 1.00 1.06 1.03 1.06 1.05 1.05
f1 (ppm)

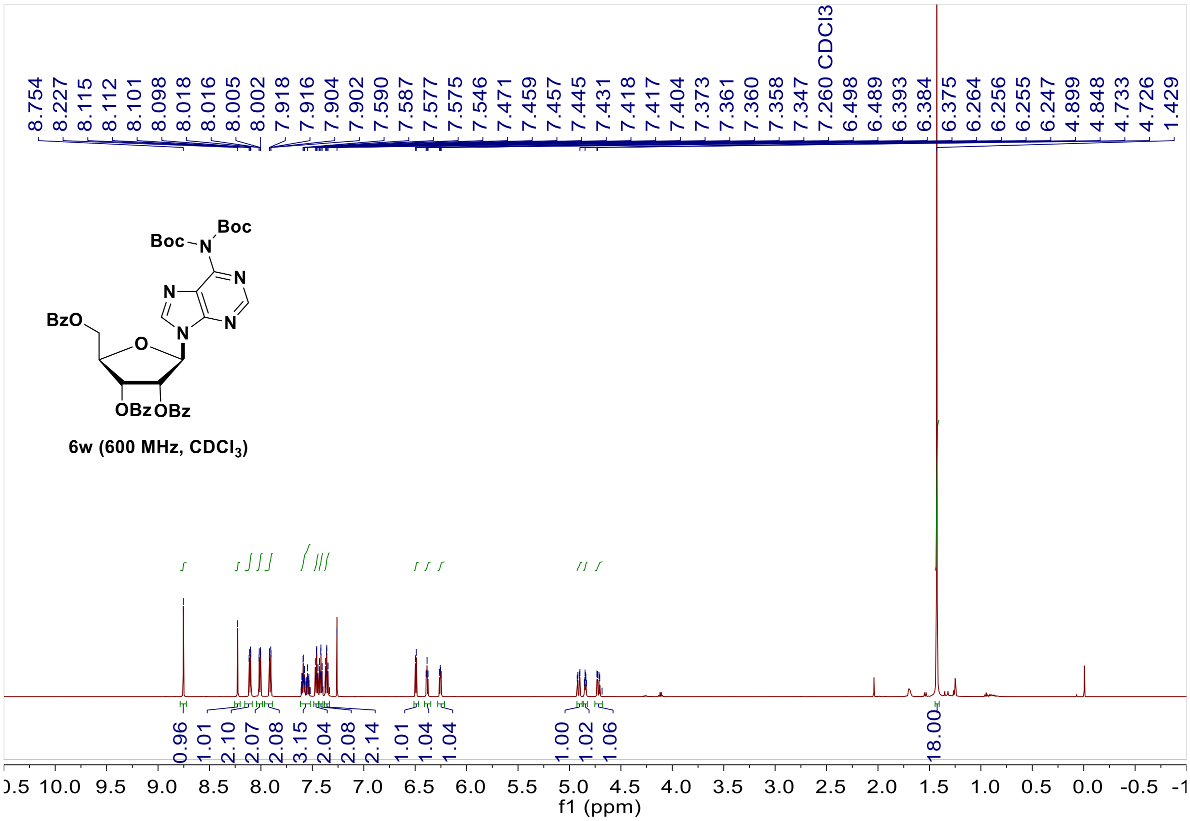
8.754 8.227 8.115 8.112 8.101 8.098 8.018 8.016 8.005 8.002 7.918 7.916 7.904 7.902 7.590 7.587 7.577 7.575 7.546 7.471 7.459 7.457 7.445 7.431 7.418 7.417 7.404 7.373 7.361 7.360 7.358 7.347 7.260 CDCl3 6.498 6.489 6.393 6.384 6.375 6.264 6.256 6.255 6.247 4.899 4.848 4.733 4.726 1.429
Boc
Boc
N
N
N
N
N
BzO
O
OBz OBz
6w (600 MHz, CDCl3)
0.96 1.01 2.10 2.07 2.08 3.15 2.04 2.08 2.14 1.01 1.04 1.04 1.00 1.02 1.06 18.00
f1 (ppm)
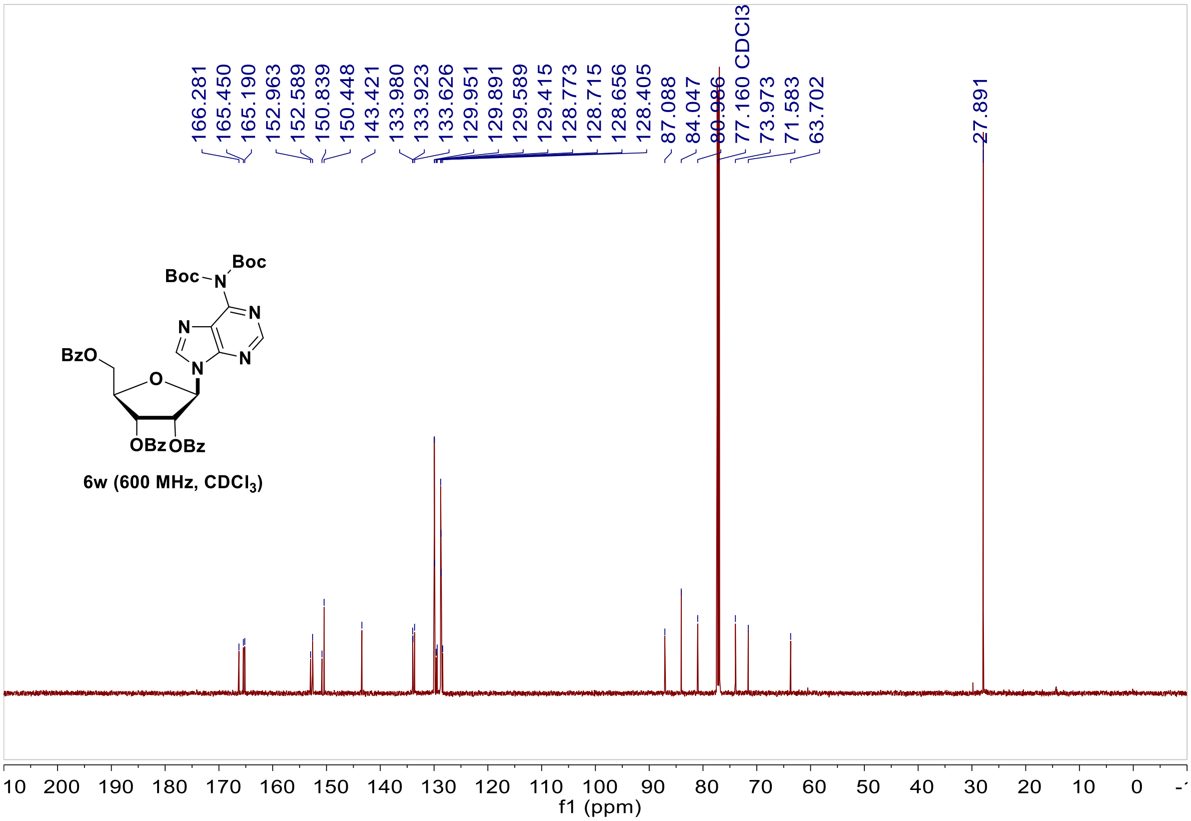
166.281 165.450 165.190 152.963 152.589 150.839 150.448 143.421 133.980 133.923 133.626 129.951 129.891 129.589 129.415 128.773 128.715 128.656 128.405 87.088 84.047 80.986 77.160 CDCl3 73.973 71.583 63.702 27.891
Boc
Boc
N
N
N
N
N
BzO
O
OBz OBz
6w (600 MHz, CDCl3)
f1 (ppm)

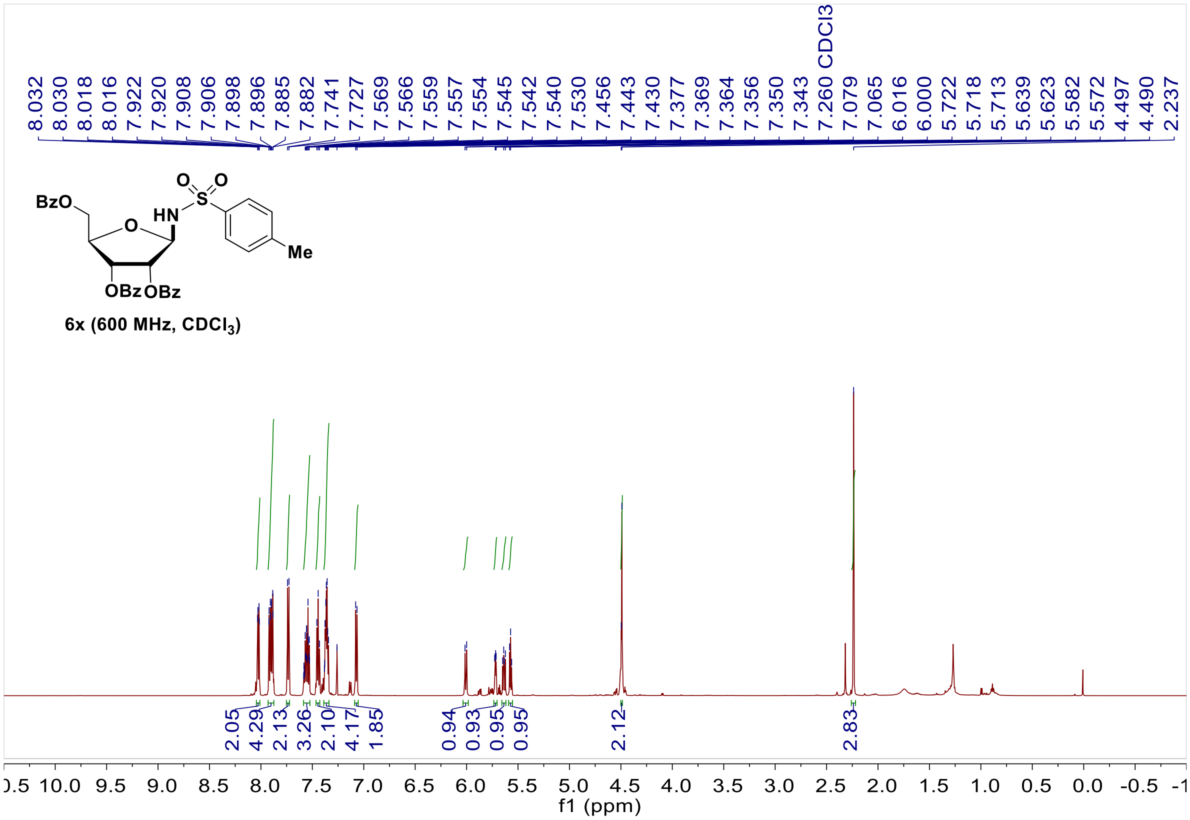

8.032
8.030
8.018
8.016
7.922
7.920
7.908
7.906
7.898
7.896
7.885
7.882
7.741
7.727
7.569
7.566
7.559
7.557
7.554
7.545
7.542
7.540
7.530
7.456
7.443
7.430
7.377
7.369
7.364
7.356
7.350
7.343
7.260 CDCl3
7.079
7.065
6.016
6.000
5.722
5.718
5.713
5.639
5.623
5.582
5.572
4.497
4.490
2.237
BzO
O
HN
S
O
O
Me
OBz
OBz
6x (600 MHz, CDCl3)
2.05
4.29
2.13
3.26
2.10
4.17
1.85
0.94
0.93
0.95
0.95
2.12
2.83
f1 (ppm)

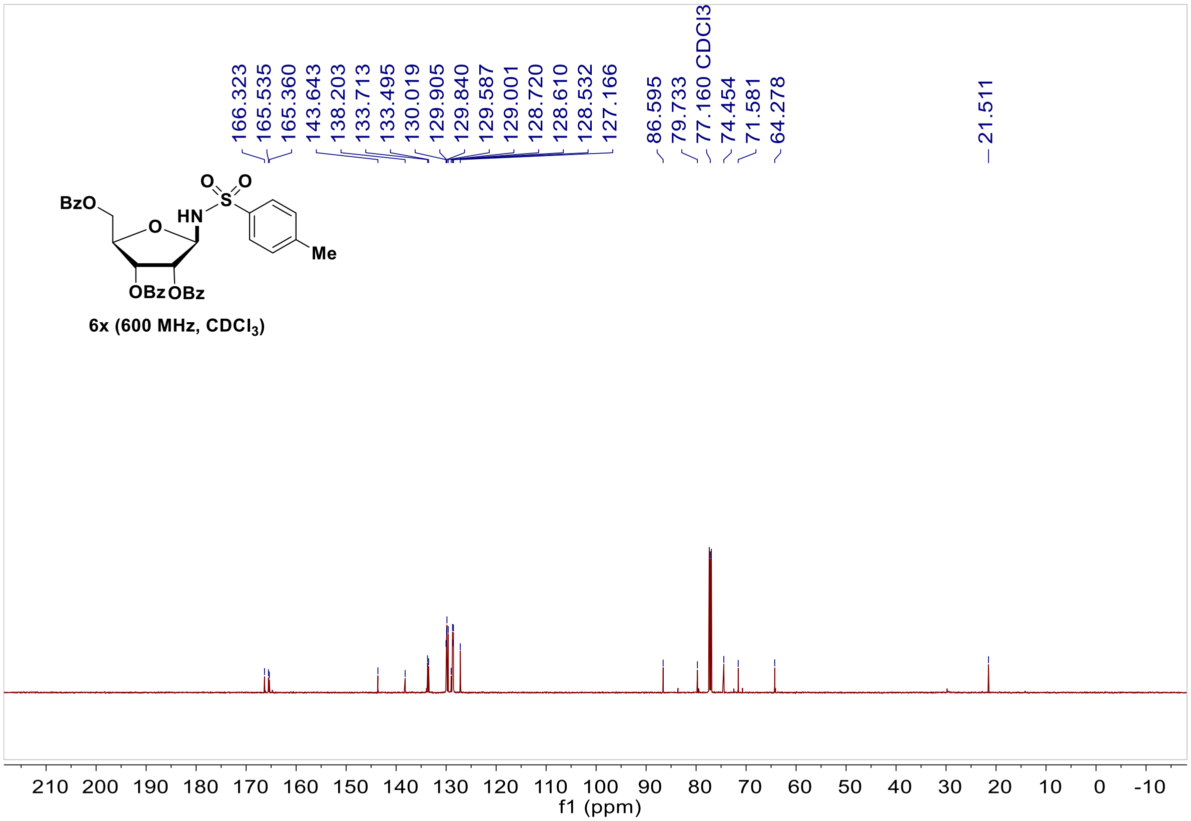

166.323
165.535
165.360
143.643
138.203
133.713
133.495
130.019
129.905
129.840
129.587
129.001
128.720
128.610
128.532
127.166
86.595
79.733
77.160 CDCl3
74.454
71.581
64.278
21.511
BzO
O
HN
S
O
O
Me
OBz
OBz
6x (600 MHz, CDCl3)
f1 (ppm)

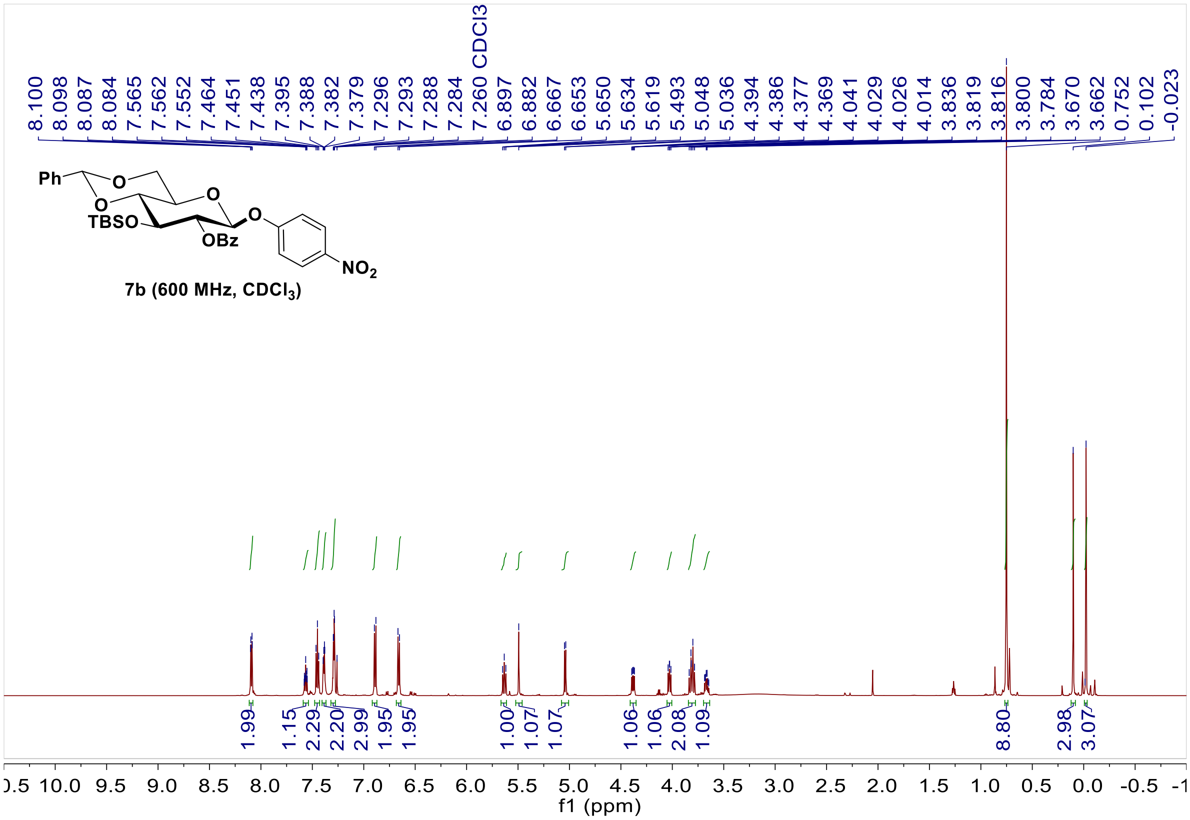
8.100 8.098 8.087 8.084 7.565 7.562 7.552 7.464 7.451 7.438 7.395 7.388 7.382 7.379 7.296 7.293 7.288 7.284 7.260 CDCl3 6.897 6.882 6.667 6.653 5.650 5.634 5.619 5.493 5.048 5.036 4.394 4.386 4.377 4.369 4.041 4.029 4.026 4.014 3.836 3.819 3.816 3.800 3.784 3.670 3.662 0.752 0.102 -0.023
Ph
O
O
O
TBSO
OBz
O
NO2
7b (600 MHz, CDCl3)
1.99 1.15 2.29 2.20 2.99 1.95 1.95 1.00 1.07 1.07 1.06 1.06 2.08 1.09 8.80 2.98 3.07
f1 (ppm)

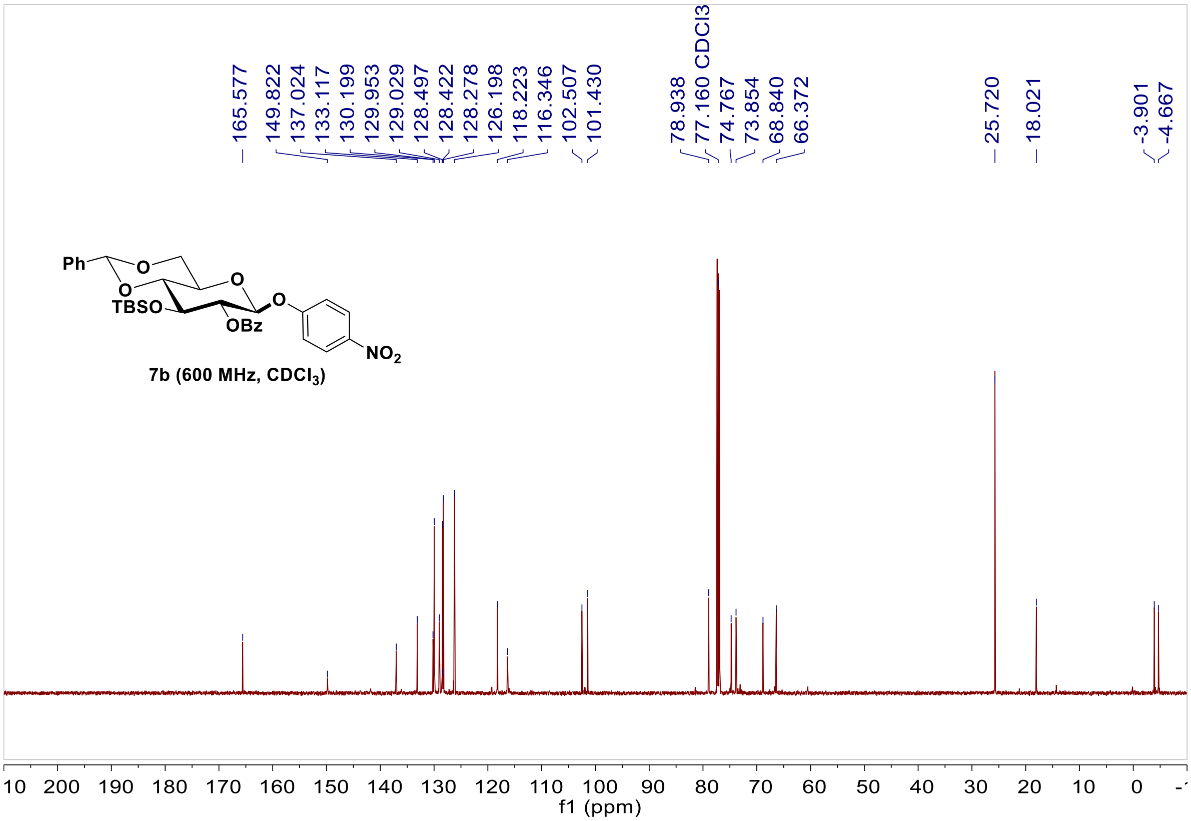
165.577 149.822 137.024 133.117 130.199 129.953 129.029 128.497 128.422 128.278 126.198 118.223 116.346 102.507 101.430 78.938 77.160 CDCl3 74.767 73.854 68.840 66.372 25.720 18.021 -3.901 -4.667
Ph
O
O
O
TBSO
OBz
O
NO2
7b (600 MHz, CDCl3)
f1 (ppm)

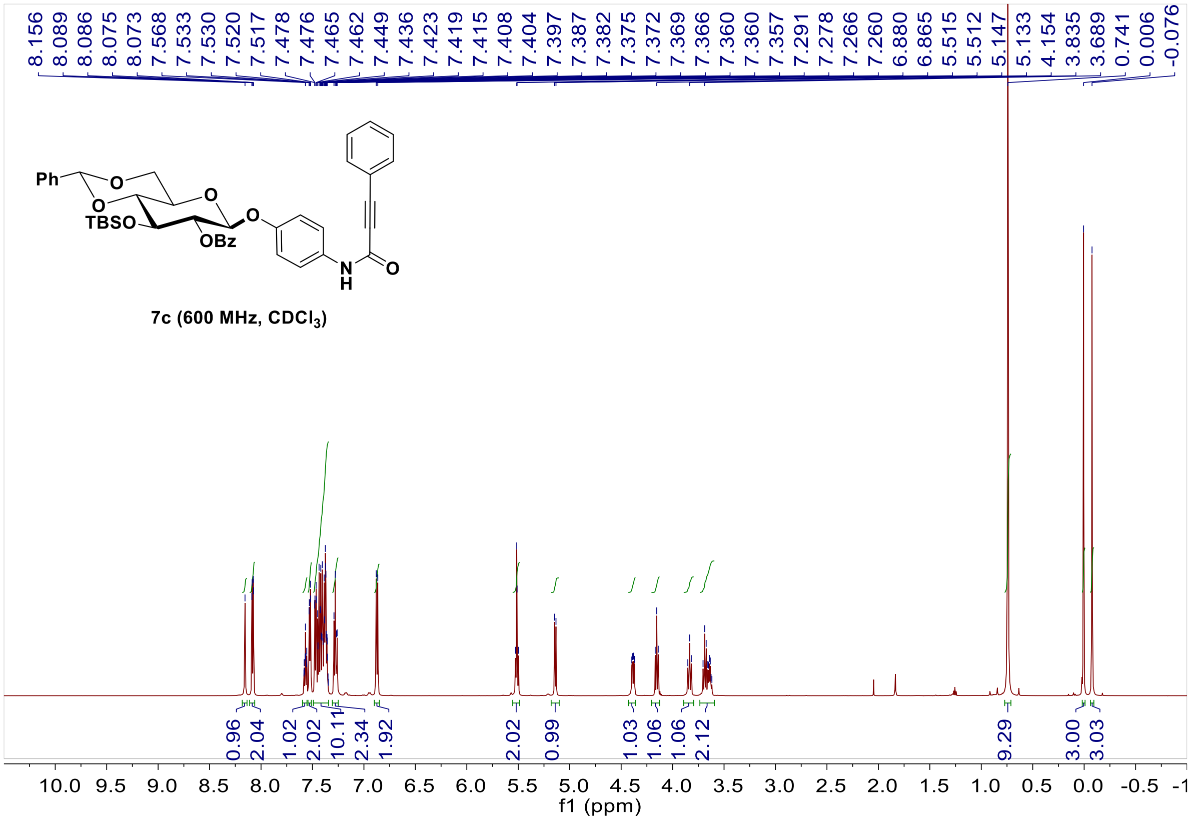
8.156 8.089 8.086 8.075 8.073 7.568 7.533 7.530 7.520 7.517 7.478 7.476 7.465 7.462 7.449 7.436 7.423 7.419 7.415 7.408 7.404 7.397 7.387 7.382 7.375 7.372 7.369 7.366 7.360 7.360 7.357 7.291 7.278 7.266 7.260 6.880 6.865 5.515 5.512 5.147 5.133 4.154 3.835 3.689 0.741 0.006 -0.076
Ph
O
O
TBSO
O
O
OBz
N
H
O
7c (600 MHz, CDCl$_3$)
0.96 2.04 1.02 2.02 10.11 2.34 1.92 2.02 0.99 1.03 1.06 1.06 2.12 9.29 3.00 3.03
10.0 9.5 9.0 8.5 8.0 7.5 7.0 6.5 6.0 5.5 5.0 4.5 4.0 3.5 3.0 2.5 2.0 1.5 1.0 0.5 0.0 -0.5 -1
f1 (ppm)

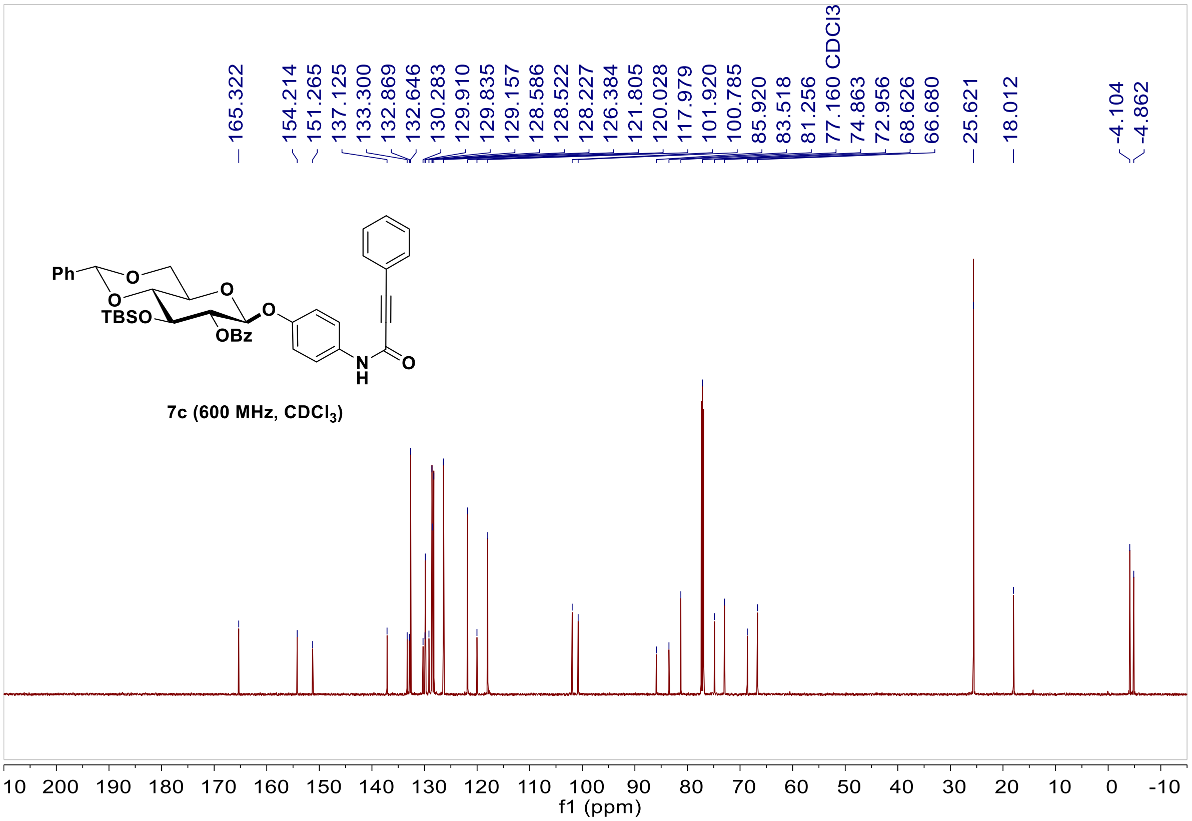
165.322 154.214 151.265 137.125 133.300 132.869 132.646 130.283 129.910 129.835 129.157 128.586 128.522 128.227 126.384 121.805 120.028 117.979 101.920 100.785 85.920 83.518 81.256 77.160 CDCl3 74.863 72.956 68.626 66.680 25.621 18.012 -4.104 -4.862
Ph
O
O
TBSO
O
O
OBz
N
H
O
7c (600 MHz, CDCl$_3$)
10 200 190 180 170 160 150 140 130 120 110 100 90 80 70 60 50 40 30 20 10 0 -10
f1 (ppm)

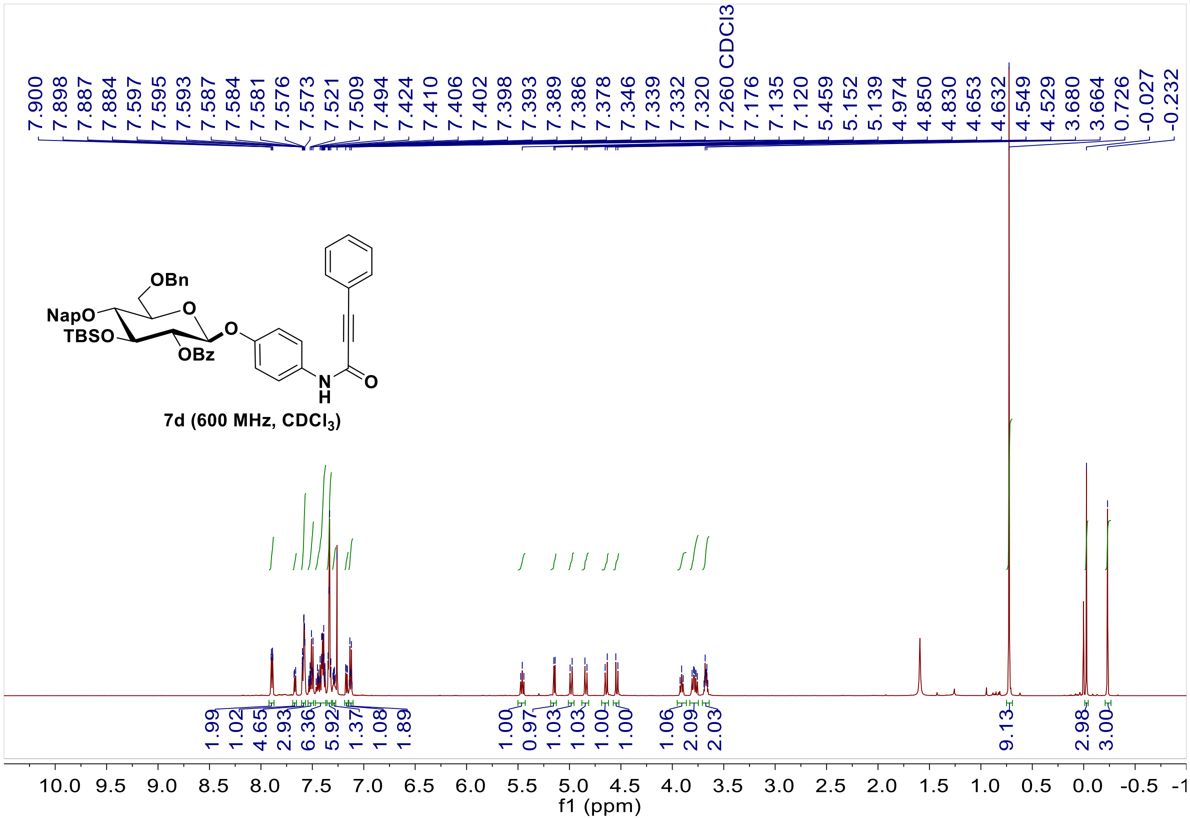
7.900
7.898
7.887
7.884
7.597
7.595
7.593
7.587
7.584
7.581
7.576
7.573
7.521
7.509
7.494
7.424
7.410
7.406
7.402
7.398
7.393
7.389
7.386
7.378
7.346
7.339
7.332
7.320
7.260 CDCl3
7.176
7.135
7.120
5.459
5.152
5.139
4.974
4.850
4.830
4.653
4.632
4.549
4.529
3.680
3.664
0.726
-0.027
-0.232
OBn
NapO
TBSO
OBz
O
N
H
O
7d (600 MHz, CDCl3)
1.99
1.02
4.65
2.93
6.36
5.92
1.37
1.08
1.89
1.00
0.97
1.03
1.03
1.00
1.00
1.06
2.09
2.03
9.13
2.98
3.00
f1 (ppm)

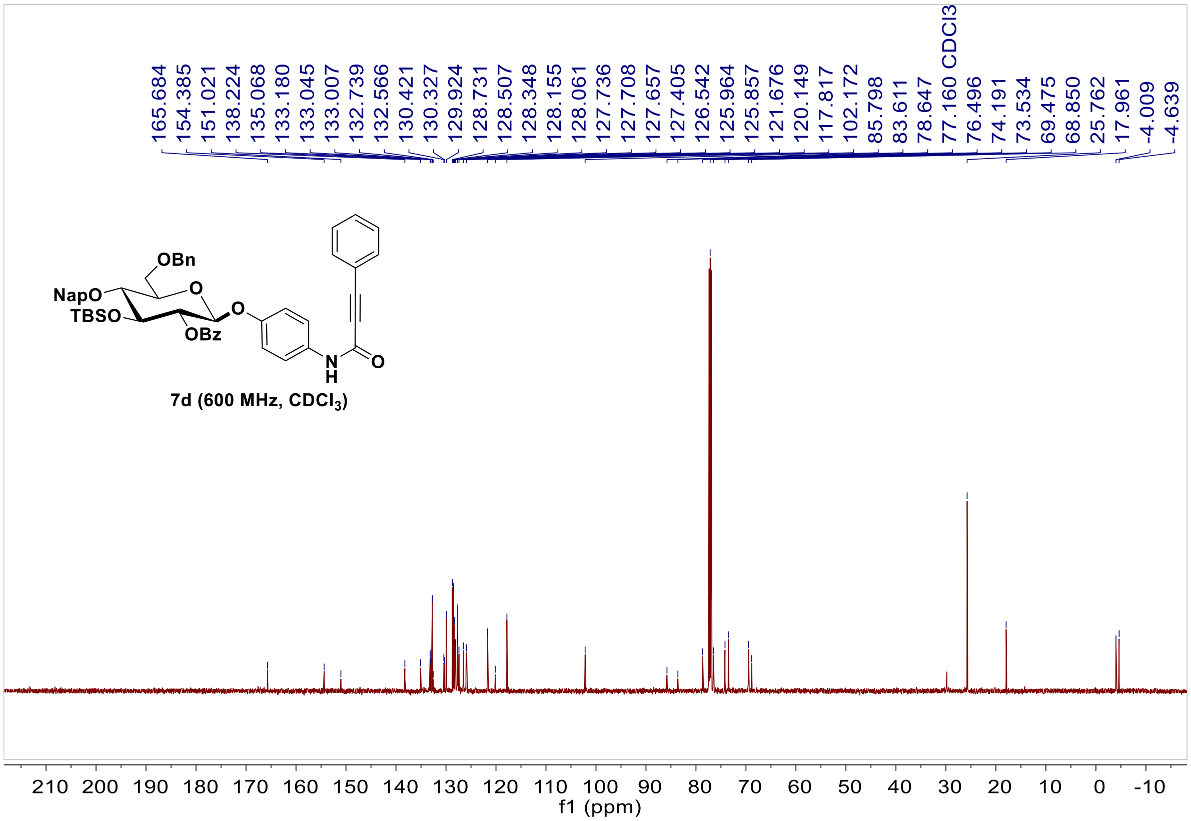
165.684
154.385
151.021
138.224
135.068
133.180
133.045
133.007
132.739
132.566
130.421
130.327
129.924
128.731
128.507
128.348
128.155
128.061
127.736
127.708
127.657
127.405
126.542
125.964
125.857
121.676
120.149
117.817
102.172
85.798
83.611
78.647
77.160 CDCl3
76.496
74.191
73.534
69.475
68.850
25.762
17.961
-4.009
-4.639
OBn
NapO
TBSO
OBz
O
N
H
O
7d (600 MHz, CDCl3)
f1 (ppm)

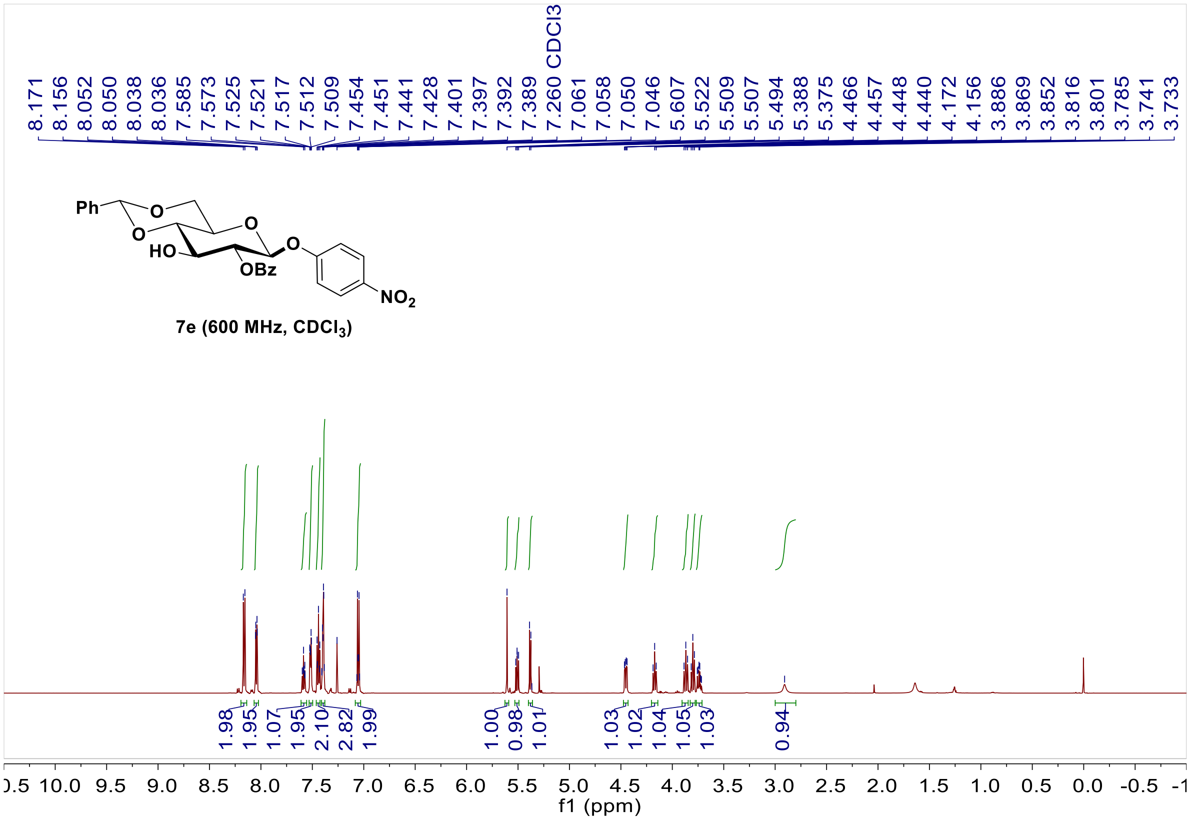
8.171 8.156 8.052 8.050 8.038 8.036 7.585 7.573 7.525 7.521 7.517 7.512 7.509 7.454 7.451 7.441 7.428 7.401 7.397 7.392 7.389 7.260 CDCl3 7.061 7.058 7.050 7.046 5.607 5.522 5.509 5.507 5.494 5.388 5.375 4.466 4.457 4.448 4.440 4.172 4.156 3.886 3.869 3.852 3.816 3.801 3.785 3.741 3.733
Ph
O
O
O
HO
OBz
O
NO2
7e (600 MHz, CDCl3)
1.98 1.95 1.07 1.95 2.10 2.82 1.99 1.00 0.98 1.01 1.03 1.02 1.04 1.05 1.03 0.94
f1 (ppm)

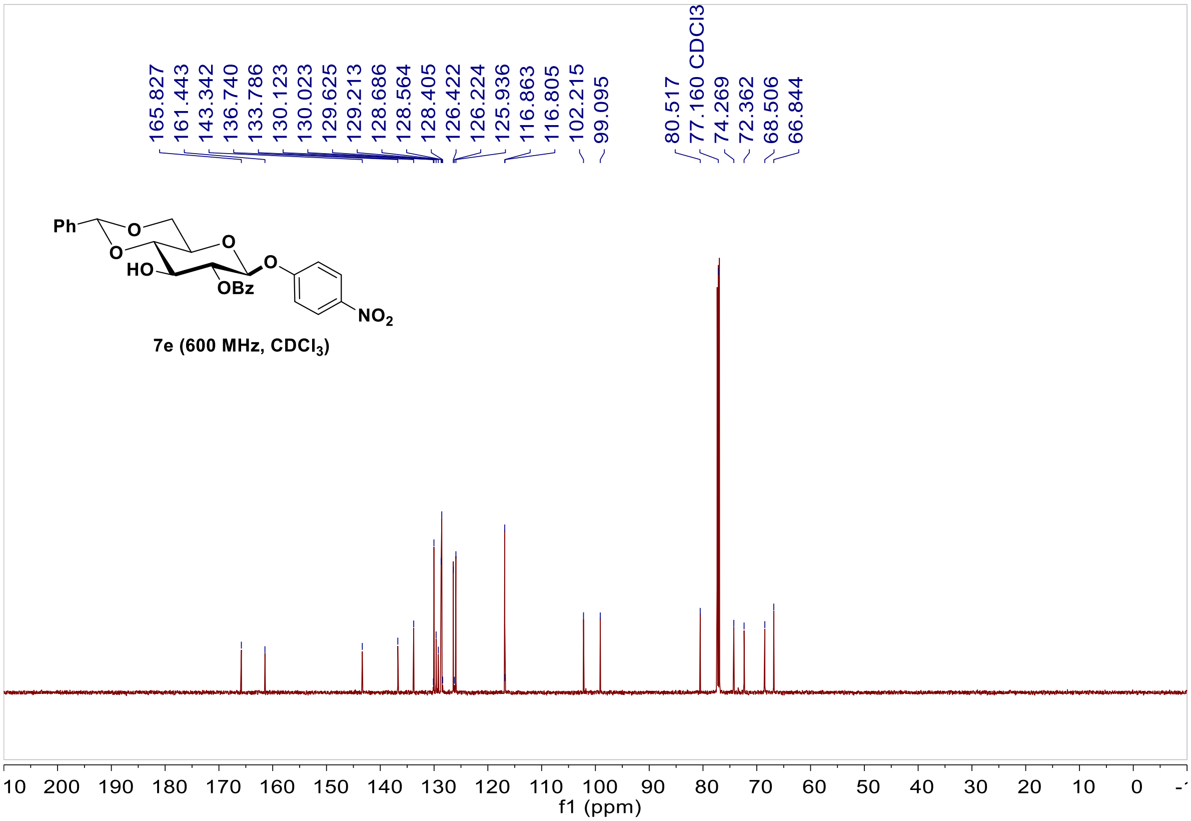
165.827 161.443 143.342 136.740 133.786 130.123 130.023 129.625 129.213 128.686 128.564 128.405 126.422 126.224 125.936 116.863 116.805 102.215 99.095 80.517 77.160 CDCl3 74.269 72.362 68.506 66.844
Ph
O
O
O
HO
OBz
O
NO2
7e (600 MHz, CDCl3)
f1 (ppm)

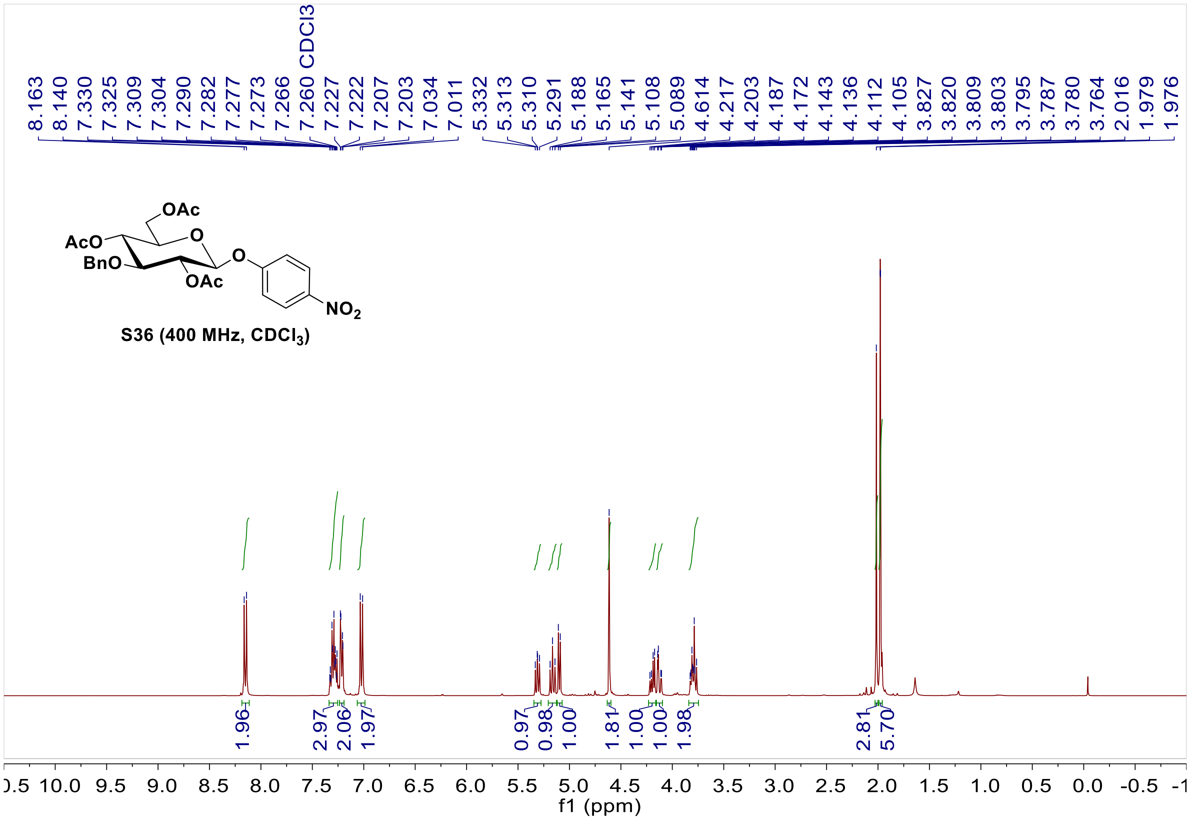
8.163
8.140
7.330
7.325
7.309
7.304
7.290
7.282
7.277
7.273
7.266
7.260 CDCl3
7.227
7.222
7.207
7.203
7.034
7.011
5.332
5.313
5.310
5.291
5.188
5.165
5.141
5.108
5.089
4.614
4.217
4.203
4.187
4.172
4.143
4.136
4.112
4.105
3.827
3.820
3.809
3.803
3.795
3.787
3.780
3.764
2.016
1.979
1.976
OAc
AcO
O
BnO
O
OAc
NO2
S36 (400 MHz, CDCl3)
1.96
2.97
2.06
1.97
0.97
0.98
1.00
1.81
1.00
1.00
1.98
2.81
5.70
f1 (ppm)

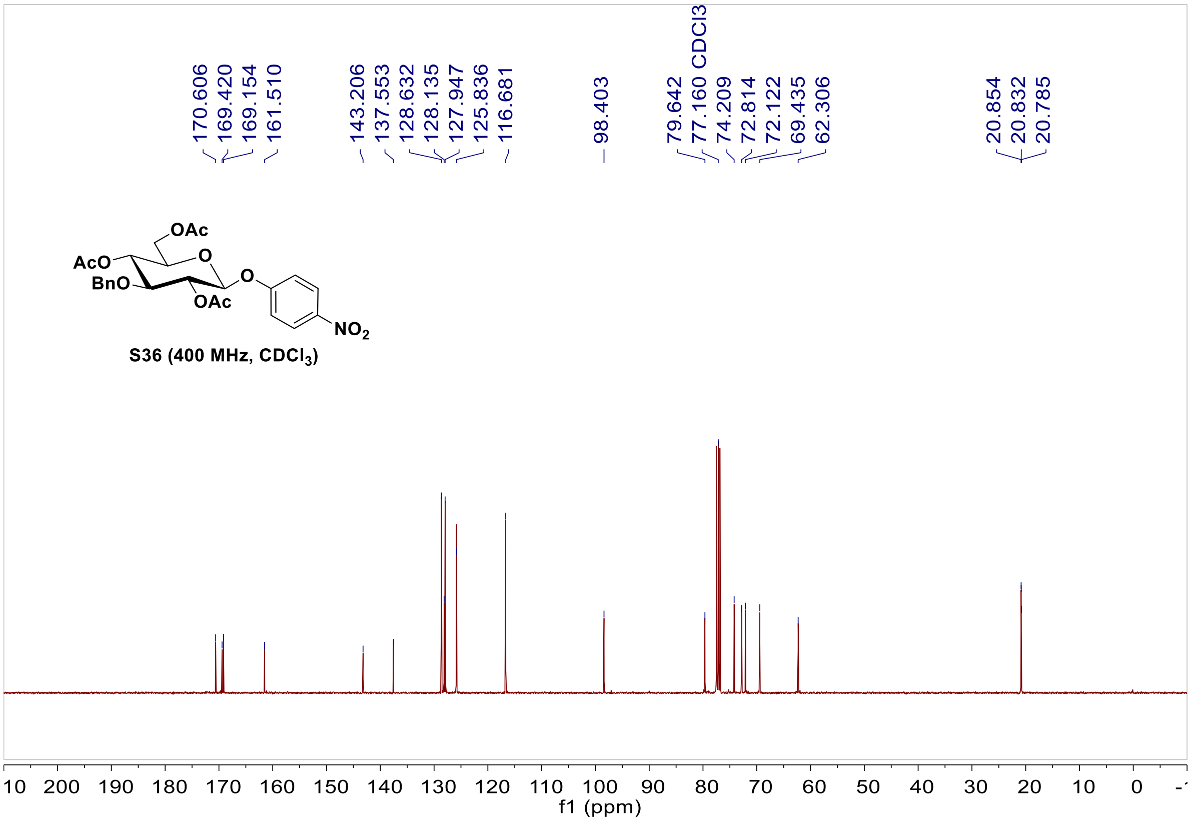
170.606
169.420
169.154
161.510
143.206
137.553
128.632
128.135
127.947
125.836
116.681
98.403
79.642
77.160 CDCl3
74.209
72.814
72.122
69.435
62.306
20.854
20.832
20.785
OAc
AcO
O
BnO
O
OAc
NO2
S36 (400 MHz, CDCl3)
f1 (ppm)

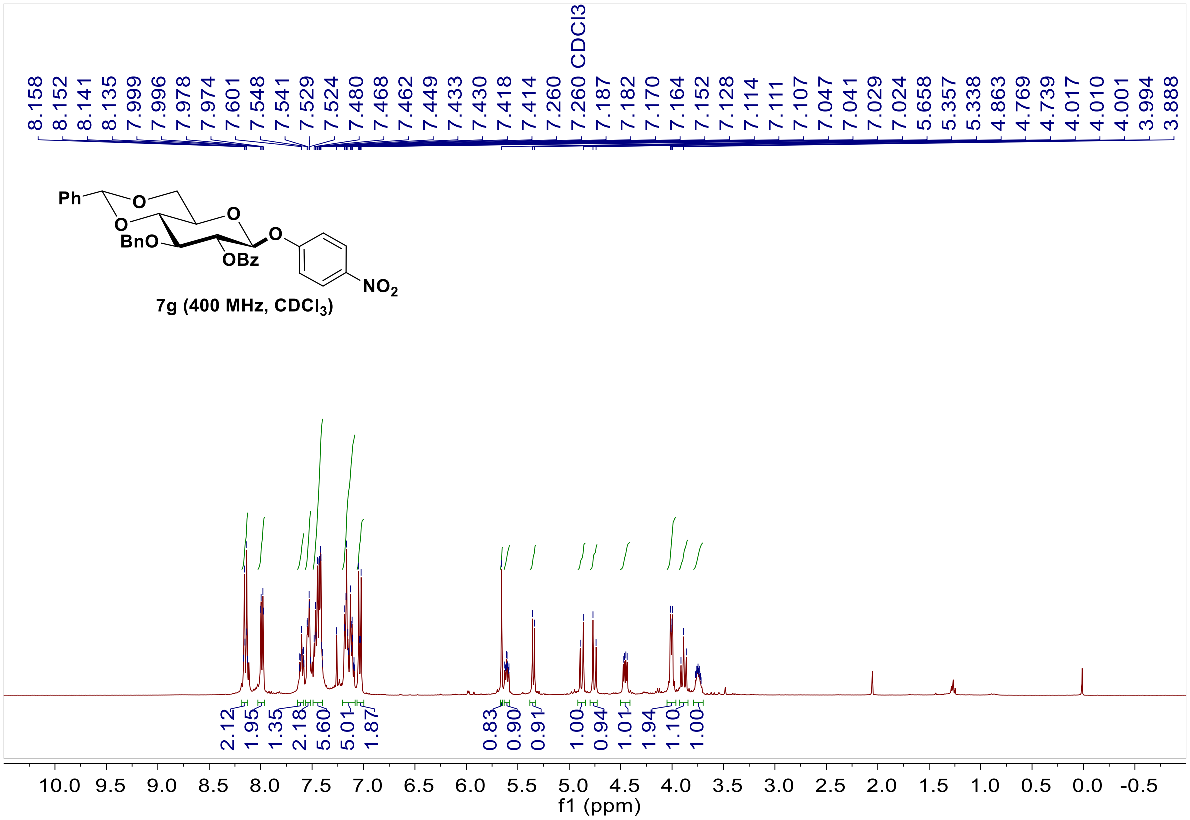
8.158 8.152 8.141 8.135 7.999 7.996 7.978 7.974 7.601 7.548 7.541 7.529 7.524 7.480 7.468 7.462 7.449 7.433 7.430 7.418 7.414 7.260 7.260 CDCl3 7.187 7.182 7.170 7.164 7.152 7.128 7.114 7.111 7.107 7.047 7.041 7.029 7.024 5.658 5.357 5.338 4.863 4.769 4.739 4.017 4.010 4.001 3.994 3.888
Ph
O
O
O
O
BnO
OBz
NO2
7g (400 MHz, CDCl3)
2.12 1.95 1.35 2.18 5.60 5.01 1.87 0.83 0.90 0.91 1.00 0.94 1.01 1.94 1.10 1.00
10.0 9.5 9.0 8.5 8.0 7.5 7.0 6.5 6.0 5.5 5.0 4.5 4.0 3.5 3.0 2.5 2.0 1.5 1.0 0.5 0.0 -0.5
f1 (ppm)

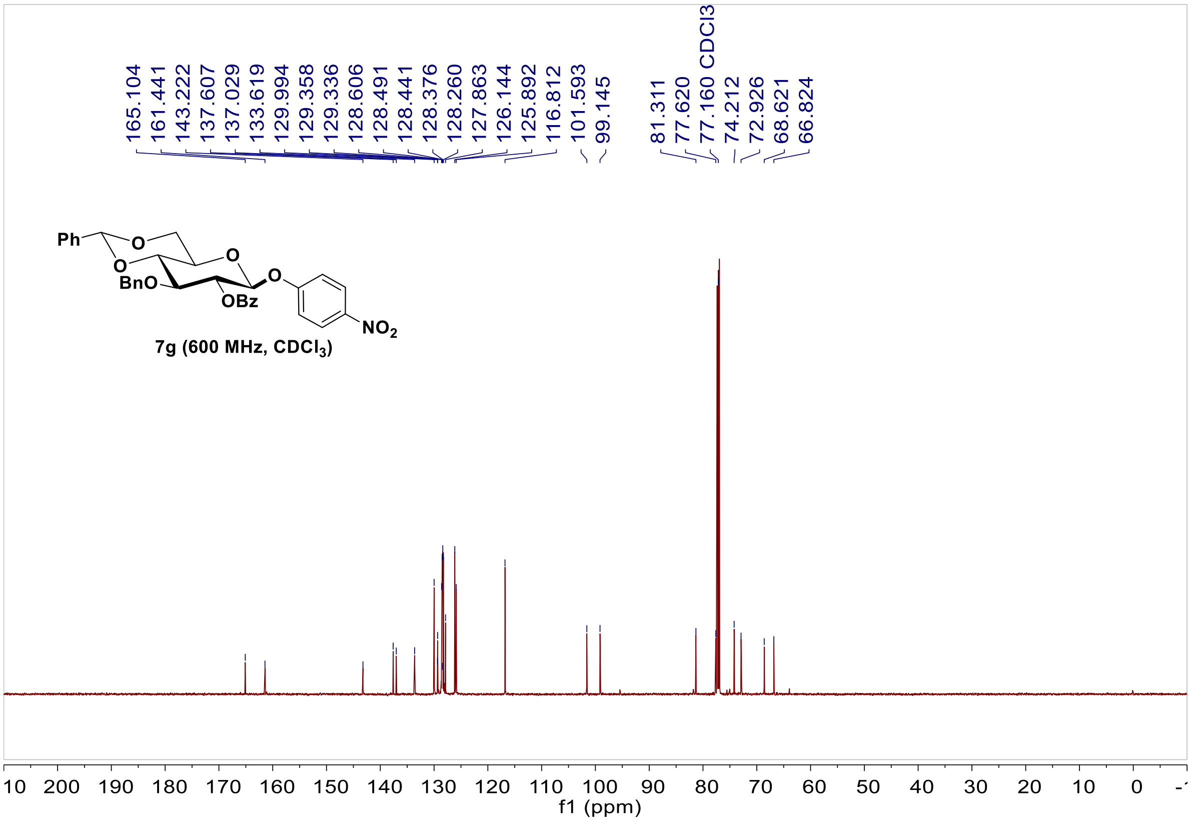
165.104 161.441 143.222 137.607 137.029 133.619 129.994 129.358 129.336 128.606 128.491 128.441 128.376 128.260 127.863 126.144 125.892 116.812 101.593 99.145 81.311 77.620 77.160 CDCl3 74.212 72.926 68.621 66.824
Ph
O
O
O
O
BnO
OBz
NO2
7g (600 MHz, CDCl3)
10 200 190 180 170 160 150 140 130 120 110 100 90 80 70 60 50 40 30 20 10 0
f1 (ppm)

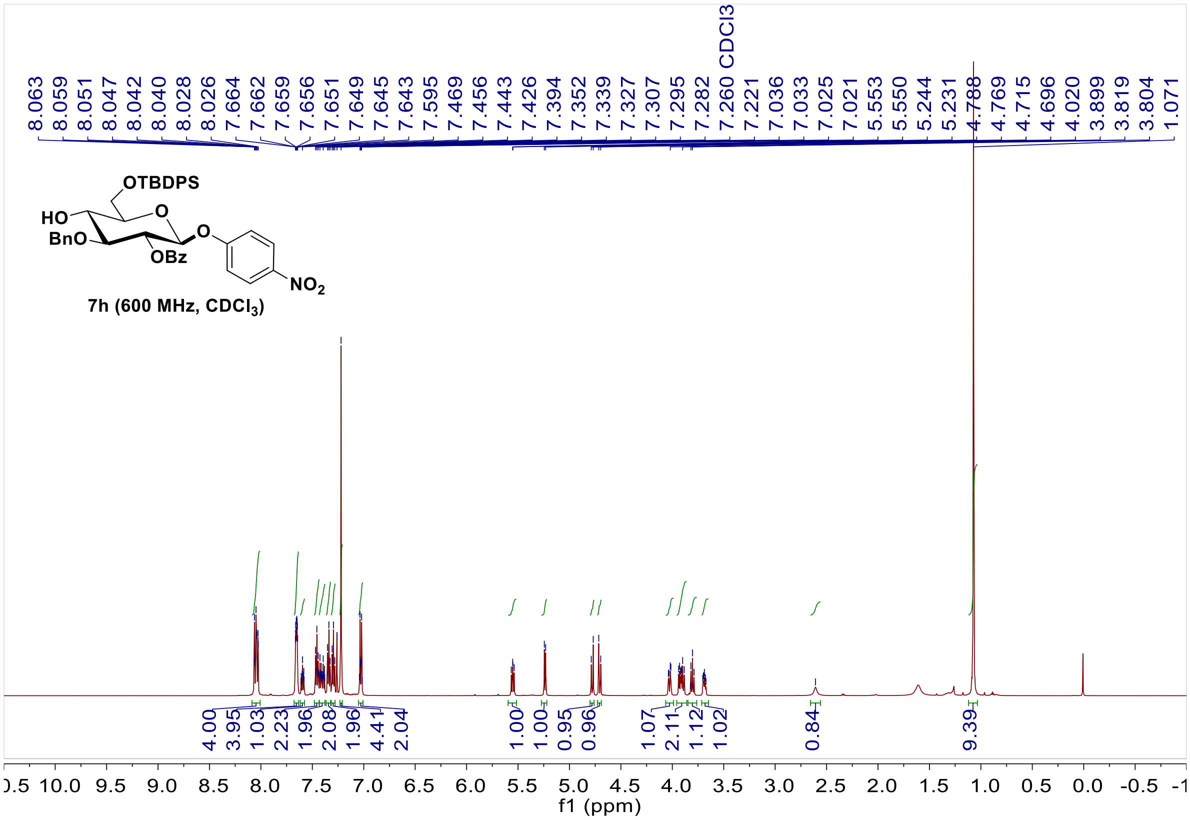
8.063 8.059 8.051 8.047 8.042 8.040 8.028 8.026 7.664 7.662 7.659 7.656 7.651 7.649 7.645 7.643 7.595 7.469 7.456 7.443 7.426 7.394 7.352 7.339 7.327 7.307 7.295 7.282 7.260 CDCl3 7.221 7.036 7.033 7.025 7.021 5.553 5.550 5.244 5.231 4.788 4.769 4.715 4.696 4.020 3.899 3.819 3.804 1.071
OTBDPS
HO
BnO
O
O
OBz
NO2
7h (600 MHz, CDCl3)
4.00 3.95 1.03 2.23 1.96 2.08 1.96 4.41 2.04 1.00 1.00 0.95 0.96 1.07 2.11 1.12 1.02 0.84 9.39
f1 (ppm)

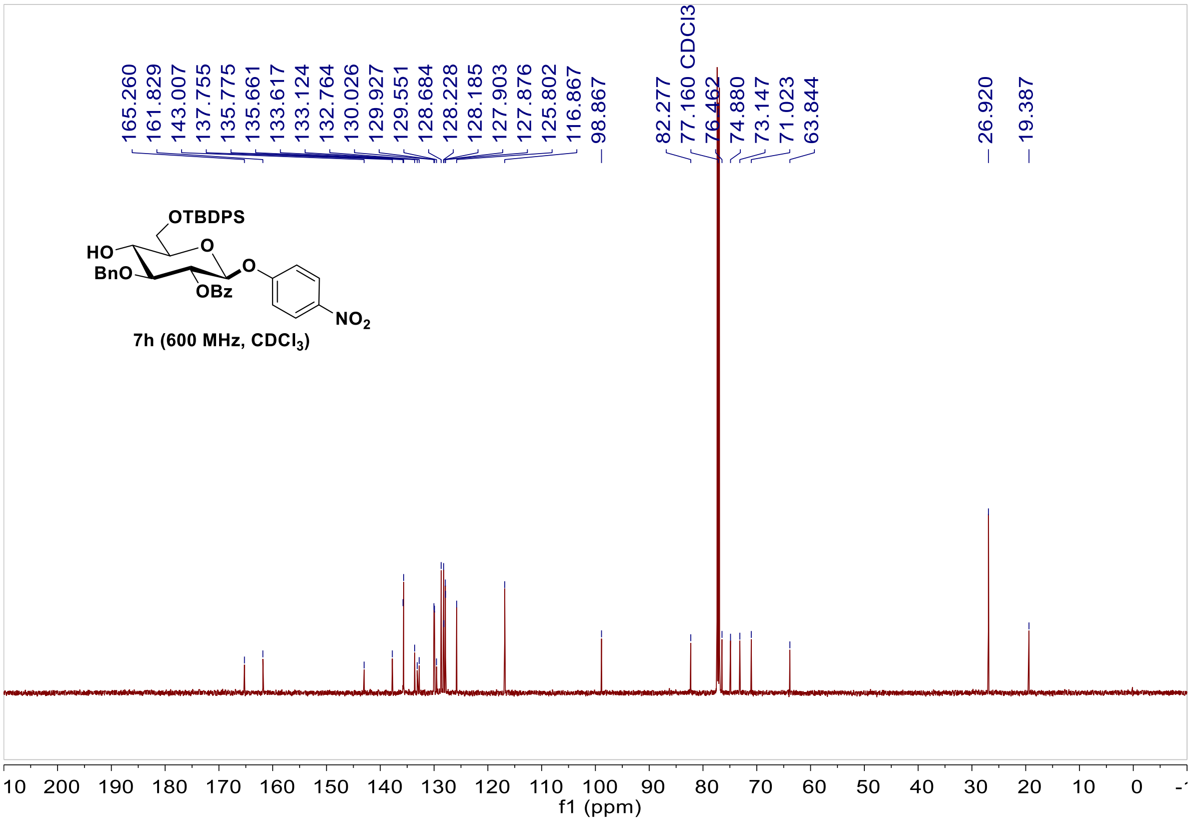
165.260 161.829 143.007 137.755 135.775 135.661 133.617 133.124 132.764 130.026 129.927 129.551 128.684 128.228 128.185 127.903 127.876 125.802 116.867 98.867 82.277 77.160 CDCl3 76.462 74.880 73.147 71.023 63.844 26.920 19.387
OTBDPS
HO
BnO
O
O
OBz
NO2
7h (600 MHz, CDCl3)
f1 (ppm)

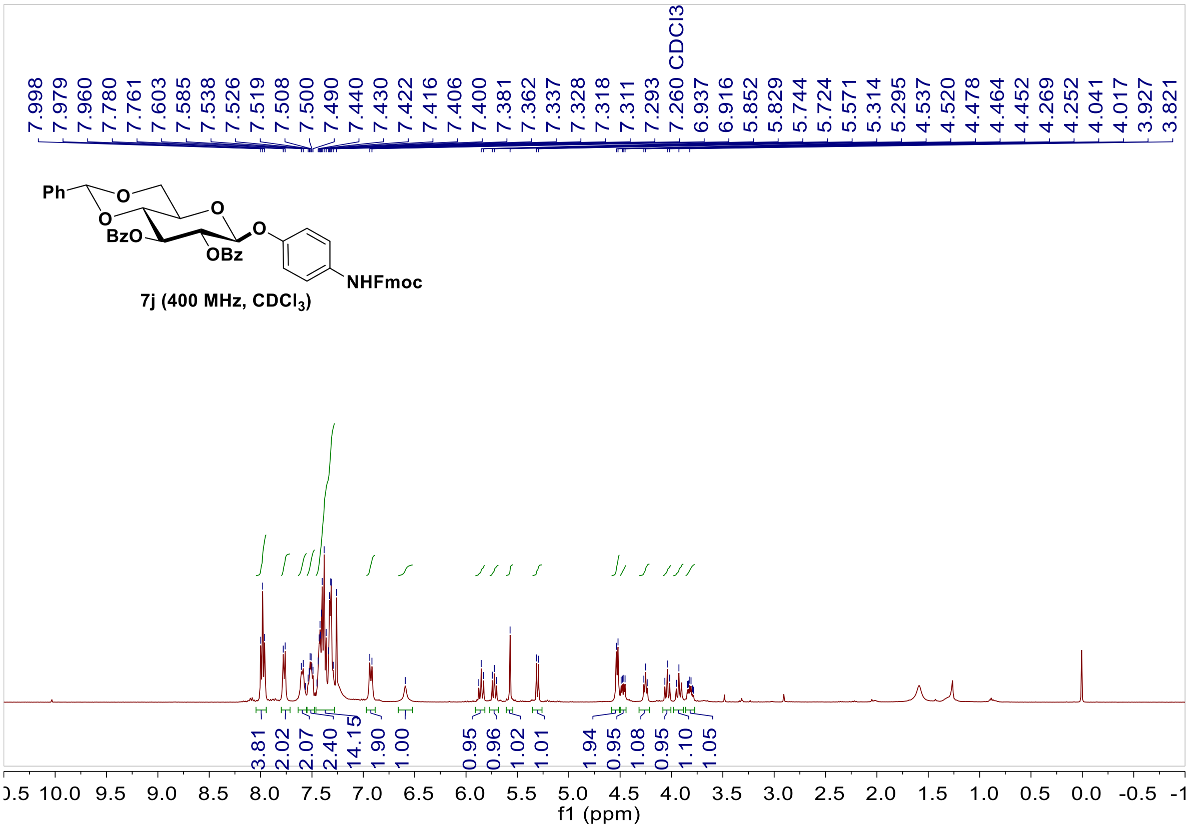
7.998
7.979
7.960
7.780
7.761
7.603
7.585
7.538
7.526
7.519
7.508
7.500
7.490
7.440
7.430
7.422
7.416
7.406
7.400
7.381
7.362
7.337
7.328
7.318
7.311
7.293
7.260 CDCl3
6.937
6.916
5.852
5.829
5.744
5.724
5.571
5.314
5.295
4.537
4.520
4.478
4.464
4.452
4.269
4.252
4.041
4.017
3.927
3.821
Ph
O
O
O
BzO
OBz
O
NHFmoc
7j (400 MHz, CDCl3)
3.81
2.02
2.07
2.40
14.15
1.90
1.00
0.95
0.96
1.02
1.01
1.94
0.95
1.08
0.95
1.10
1.05
f1 (ppm)

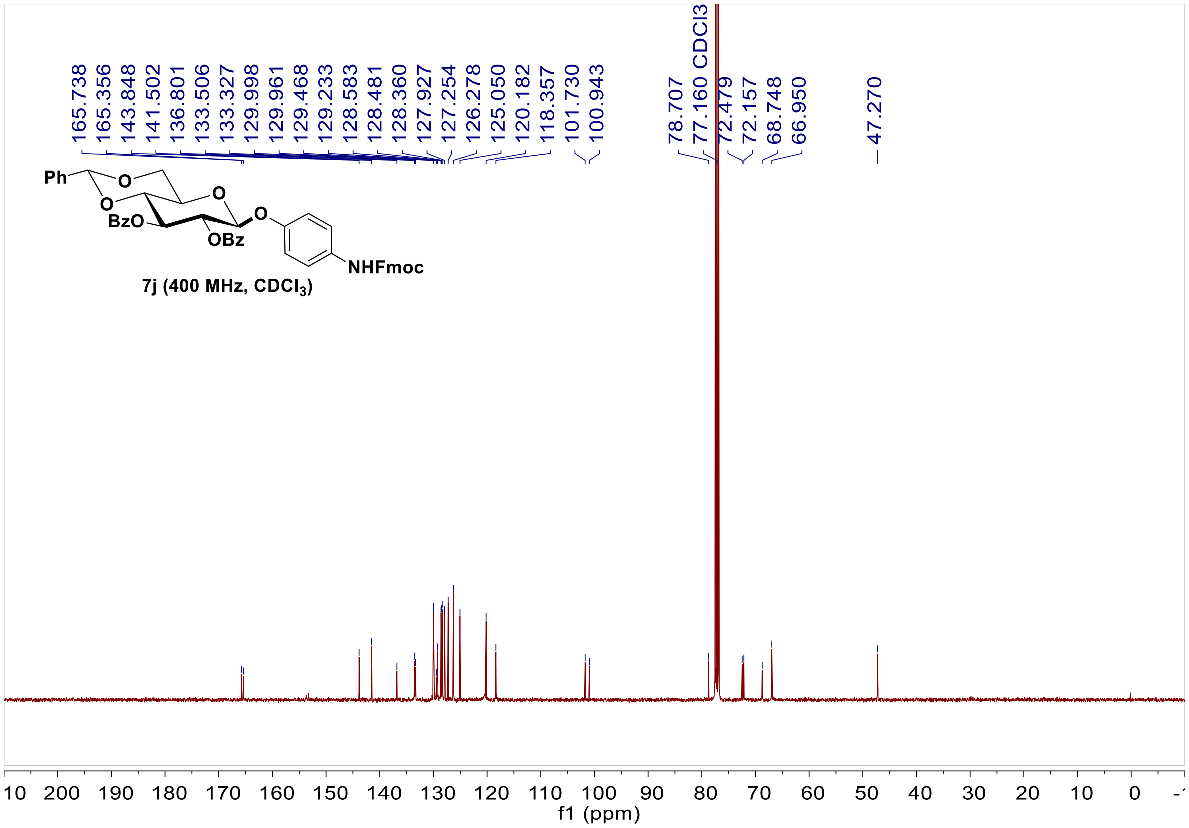
165.738
165.356
143.848
141.502
136.801
133.506
133.327
129.998
129.961
129.468
129.233
128.583
128.481
128.360
127.927
127.254
126.278
125.050
120.182
118.357
101.730
100.943
78.707
77.160 CDCl3
72.479
72.157
68.748
66.950
47.270
Ph
O
O
O
BzO
OBz
O
NHFmoc
7j (400 MHz, CDCl3)
f1 (ppm)

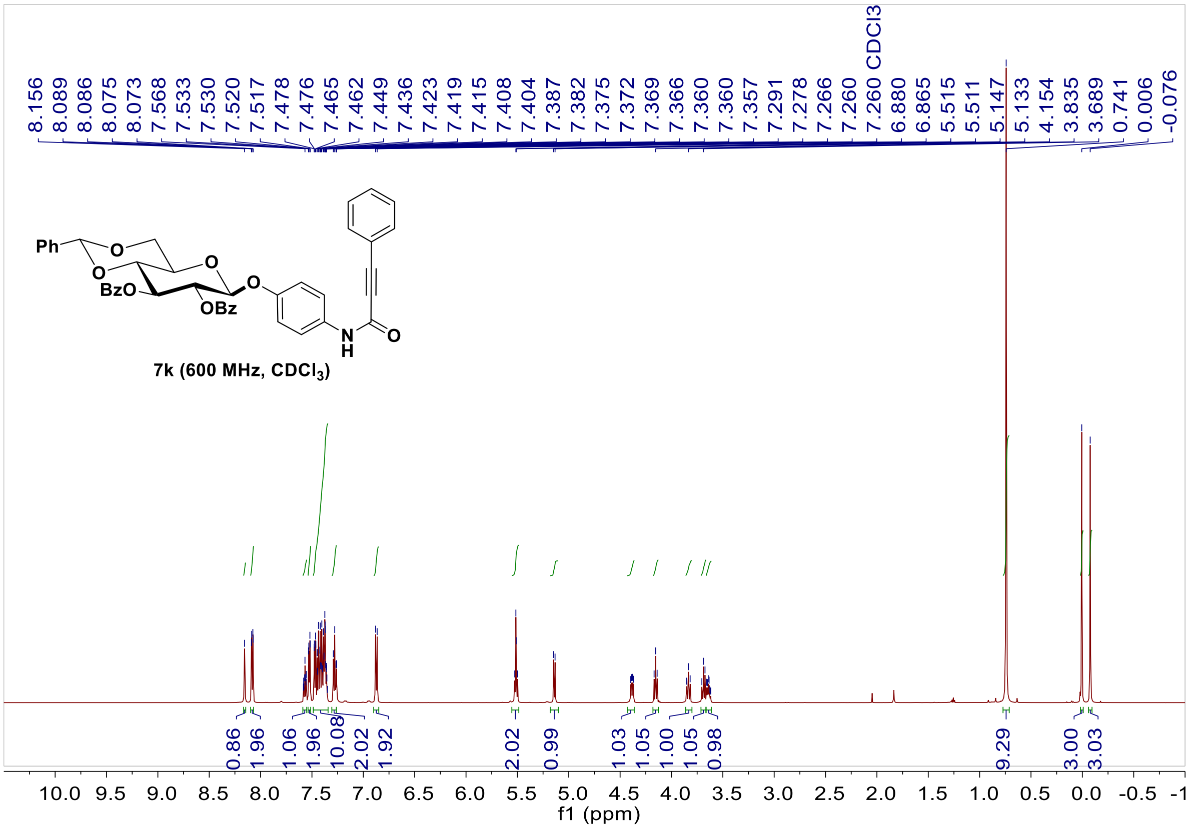
8.156 8.089 8.086 8.075 8.073 7.568 7.533 7.530 7.520 7.517 7.478 7.476 7.465 7.462 7.449 7.436 7.423 7.419 7.415 7.408 7.404 7.387 7.382 7.375 7.372 7.369 7.366 7.360 7.360 7.357 7.291 7.278 7.266 7.260 7.260 CDCl3 6.880 6.865 5.515 5.511 5.147 5.133 4.154 3.835 3.689 0.741 0.006 -0.076
7k (600 MHz, CDCl3)
0.86 1.96 1.06 1.96 10.08 2.02 1.92 2.02 0.99 1.03 1.05 1.00 1.05 0.98 9.29 3.00 3.03
10.0 9.5 9.0 8.5 8.0 7.5 7.0 6.5 6.0 5.5 5.0 4.5 4.0 3.5 3.0 2.5 2.0 1.5 1.0 0.5 0.0 -0.5 -1
f1 (ppm)

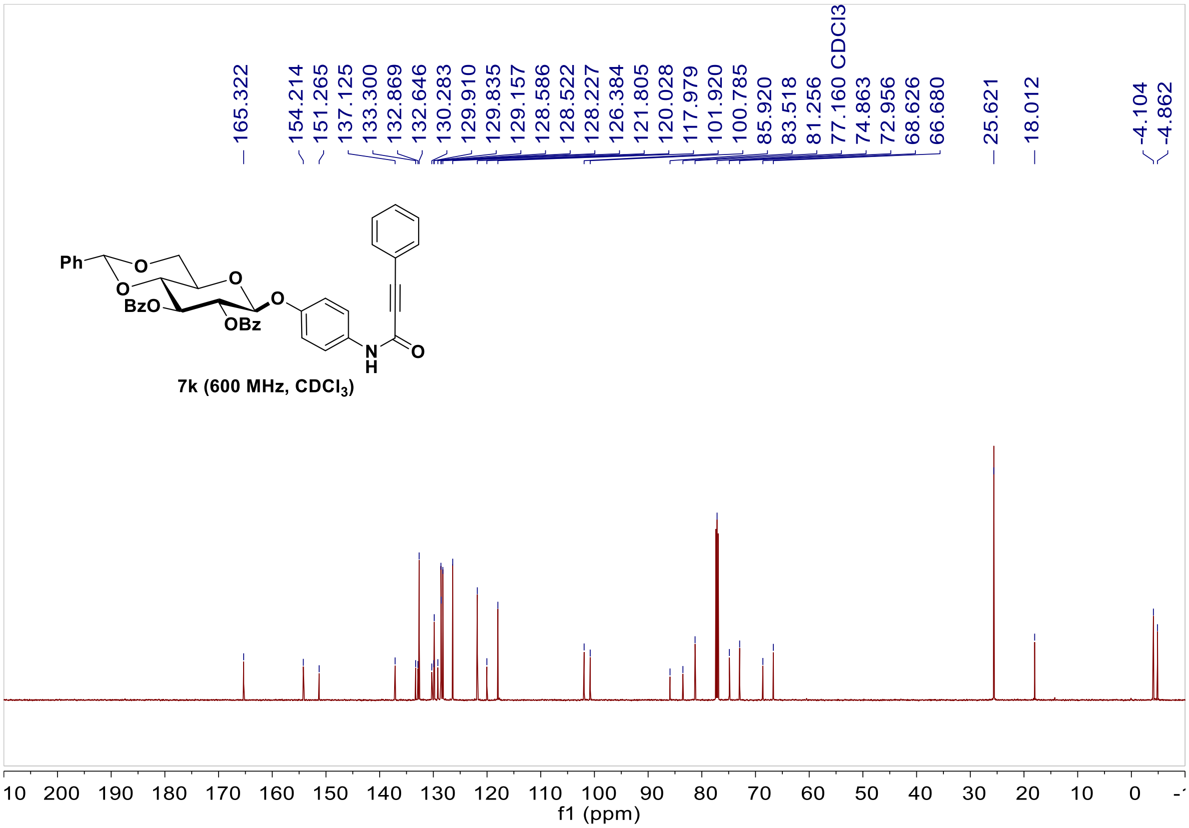
165.322 154.214 151.265 137.125 133.300 132.869 132.646 130.283 129.910 129.835 129.157 128.586 128.522 128.227 126.384 121.805 120.028 117.979 101.920 100.785 85.920 83.518 81.256 77.160 CDCl3 74.863 72.956 68.626 66.680 25.621 18.012 -4.104 -4.862
7k (600 MHz, CDCl3)
10 200 190 180 170 160 150 140 130 120 110 100 90 80 70 60 50 40 30 20 10 0
f1 (ppm)

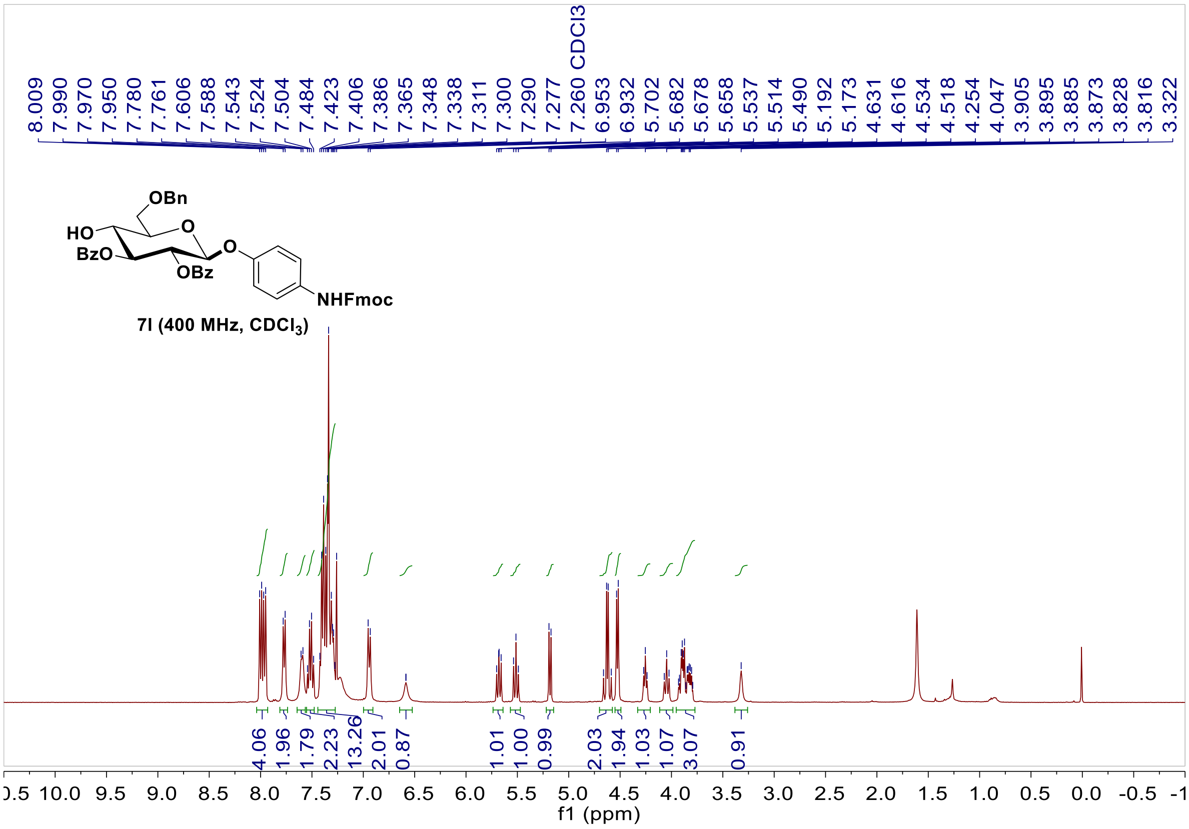
8.009 7.990 7.970 7.950 7.780 7.761 7.606 7.588 7.543 7.524 7.504 7.484 7.423 7.406 7.386 7.365 7.348 7.338 7.311 7.300 7.290 7.277 7.260 CDCl3 6.953 6.932 5.702 5.682 5.678 5.658 5.537 5.514 5.490 5.192 5.173 4.631 4.616 4.534 4.518 4.254 4.047 3.905 3.895 3.885 3.873 3.828 3.816 3.322
OBn
HO
BzO
OBz
O
NHFmoc
7l (400 MHz, CDCl3)
4.06 1.96 1.79 2.23 13.26 2.01 0.87 1.01 1.00 0.99 2.03 1.94 1.03 1.07 3.07 0.91
f1 (ppm)

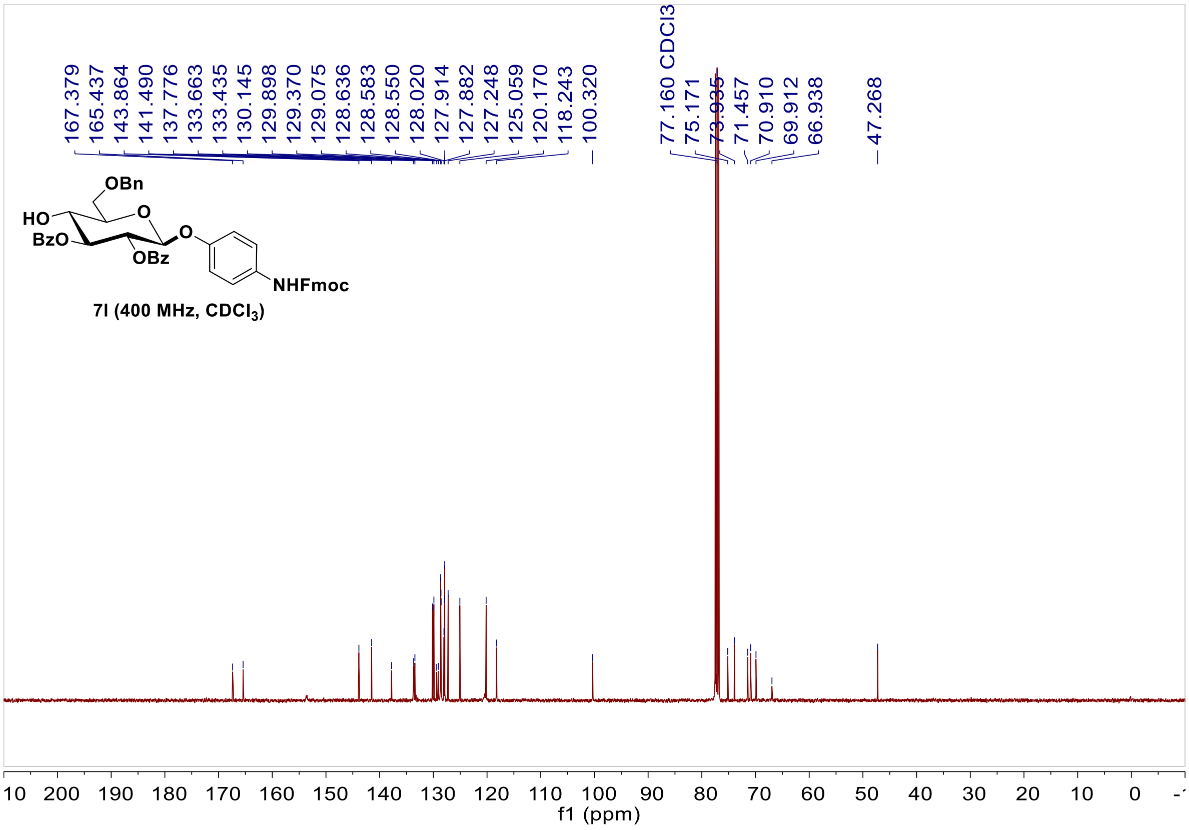
167.379 165.437 143.864 141.490 137.776 133.663 133.435 130.145 129.898 129.370 129.075 128.636 128.583 128.550 128.020 127.914 127.882 127.248 125.059 120.170 118.243 100.320
77.160 CDCl3 75.171 73.935 71.457 70.910 69.912 66.938 47.268
OBn
HO
BzO
OBz
O
NHFmoc
7l (400 MHz, CDCl3)
f1 (ppm)

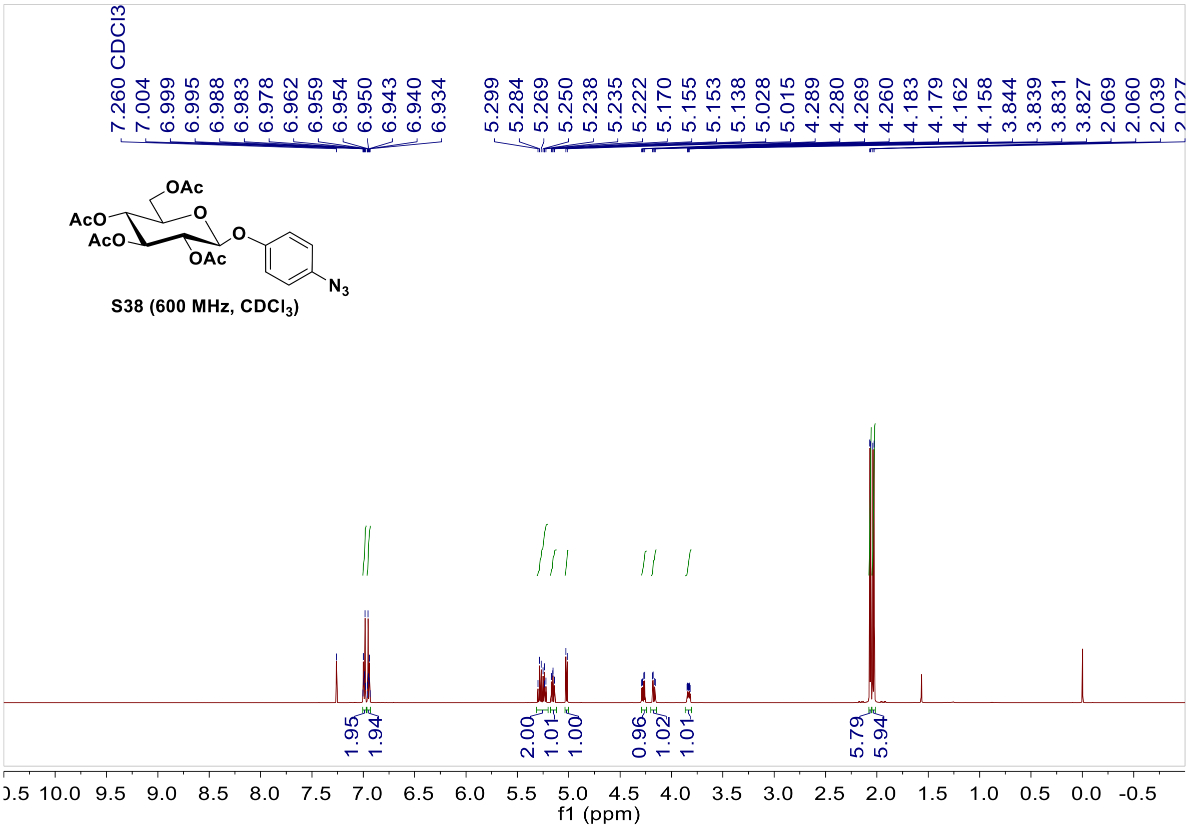
7.260 CDCl3
7.004
6.999
6.995
6.988
6.983
6.978
6.962
6.959
6.954
6.950
6.943
6.940
6.934
5.299
5.284
5.269
5.250
5.238
5.235
5.222
5.170
5.155
5.153
5.138
5.028
5.015
4.289
4.280
4.269
4.260
4.183
4.179
4.162
4.158
3.844
3.839
3.831
3.827
2.069
2.060
2.039
2.027
OAc
AcO
O
AcO
O
OAc
N3
S38 (600 MHz, CDCl3)
1.95
1.94
2.00
1.01
1.00
0.96
1.02
1.01
5.79
5.94
f1 (ppm)

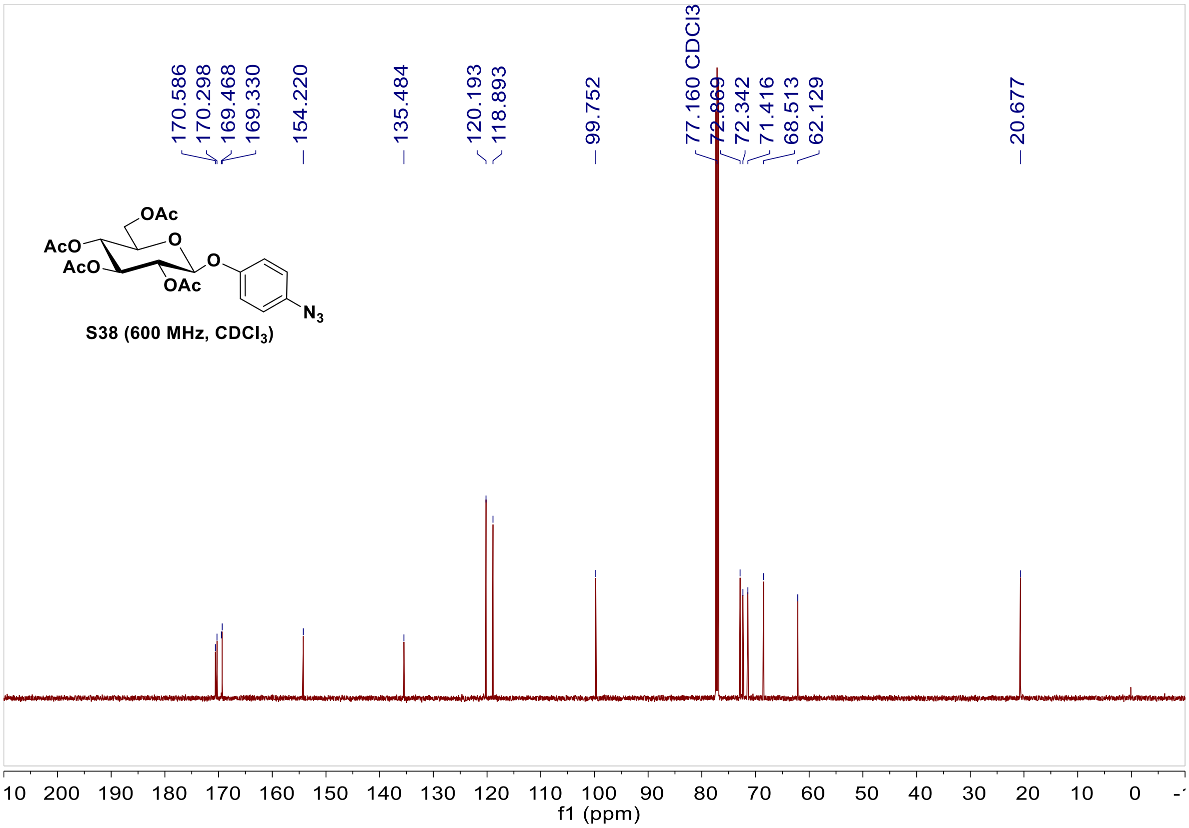
170.586
170.298
169.468
169.330
154.220
135.484
120.193
118.893
99.752
77.160 CDCl3
72.869
72.342
71.416
68.513
62.129
20.677
OAc
AcO
O
AcO
O
OAc
N3
S38 (600 MHz, CDCl3)
f1 (ppm)

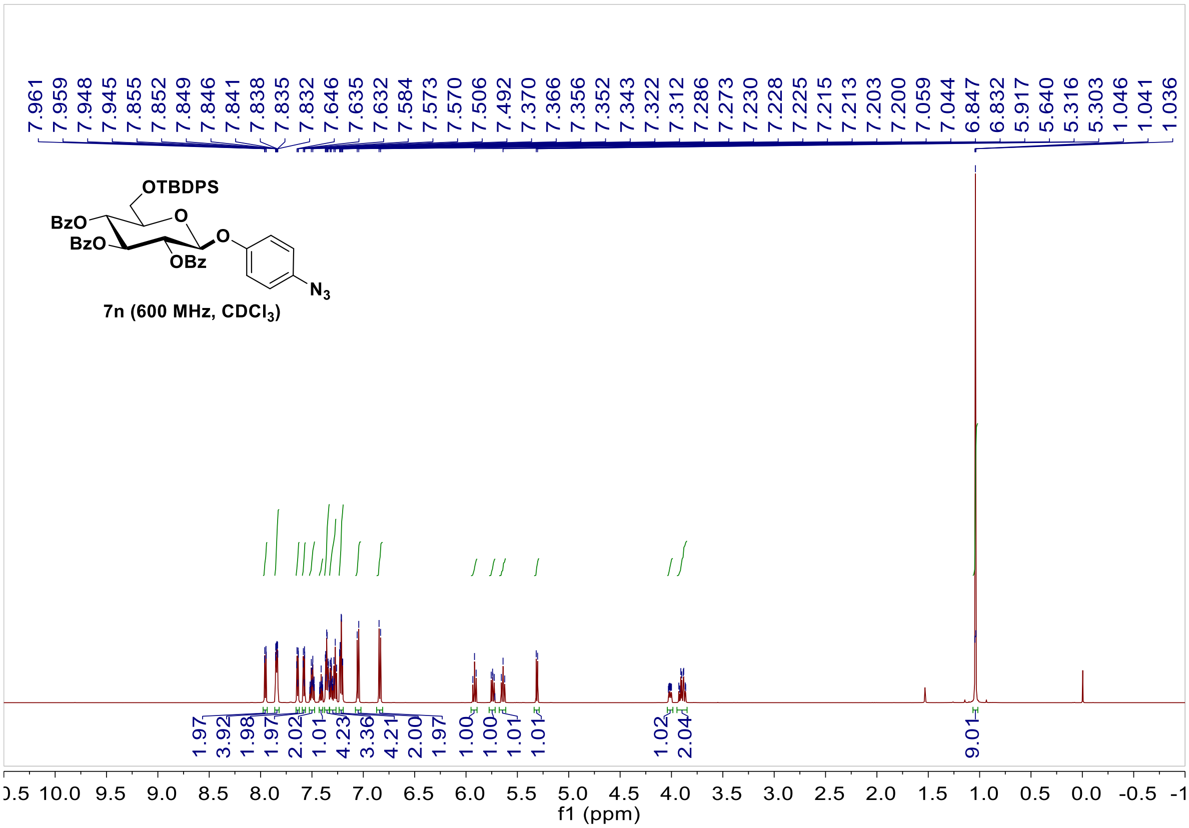
7.961 7.959 7.948 7.945 7.855 7.852 7.849 7.846 7.841 7.838 7.835 7.832 7.646 7.635 7.632 7.584 7.573 7.570 7.506 7.492 7.370 7.366 7.356 7.352 7.343 7.322 7.312 7.286 7.273 7.230 7.228 7.225 7.215 7.213 7.203 7.200 7.059 7.044 6.847 6.832 5.917 5.640 5.316 5.303 1.046 1.041 1.036
OTBDPS
BzO
BzO
O
O
OBz
N3
7n (600 MHz, CDCl3)
1.97 3.92 1.98 1.97 2.02 1.01 4.23 3.36 4.21 2.00 1.97 1.00 1.00 1.01 1.01 1.02 2.04 9.01
f1 (ppm)

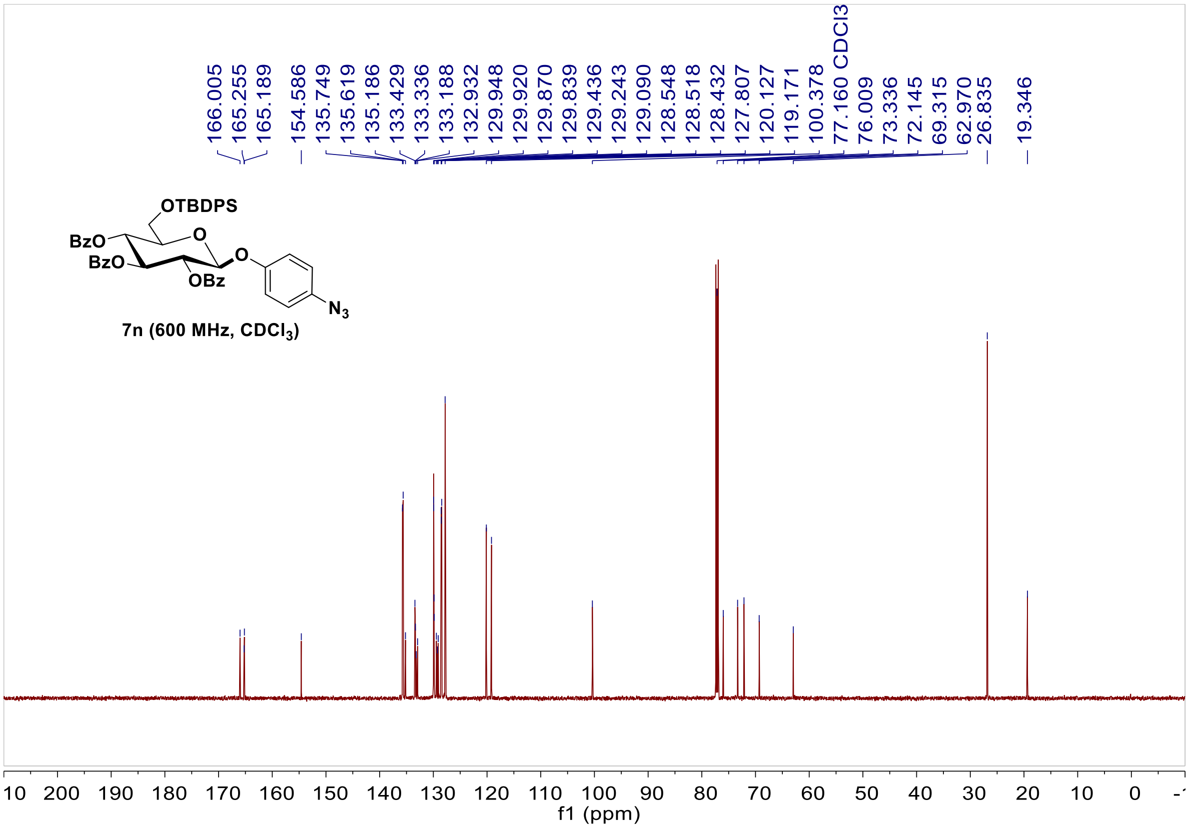
166.005 165.255 165.189 154.586 135.749 135.619 135.186 133.429 133.336 133.188 132.932 129.948 129.920 129.870 129.839 129.436 129.243 129.090 128.548 128.518 128.432 127.807 120.127 119.171 100.378 77.160 CDCl3 76.009 73.336 72.145 69.315 62.970 26.835 19.346
OTBDPS
BzO
BzO
O
O
OBz
N3
7n (600 MHz, CDCl3)
f1 (ppm)

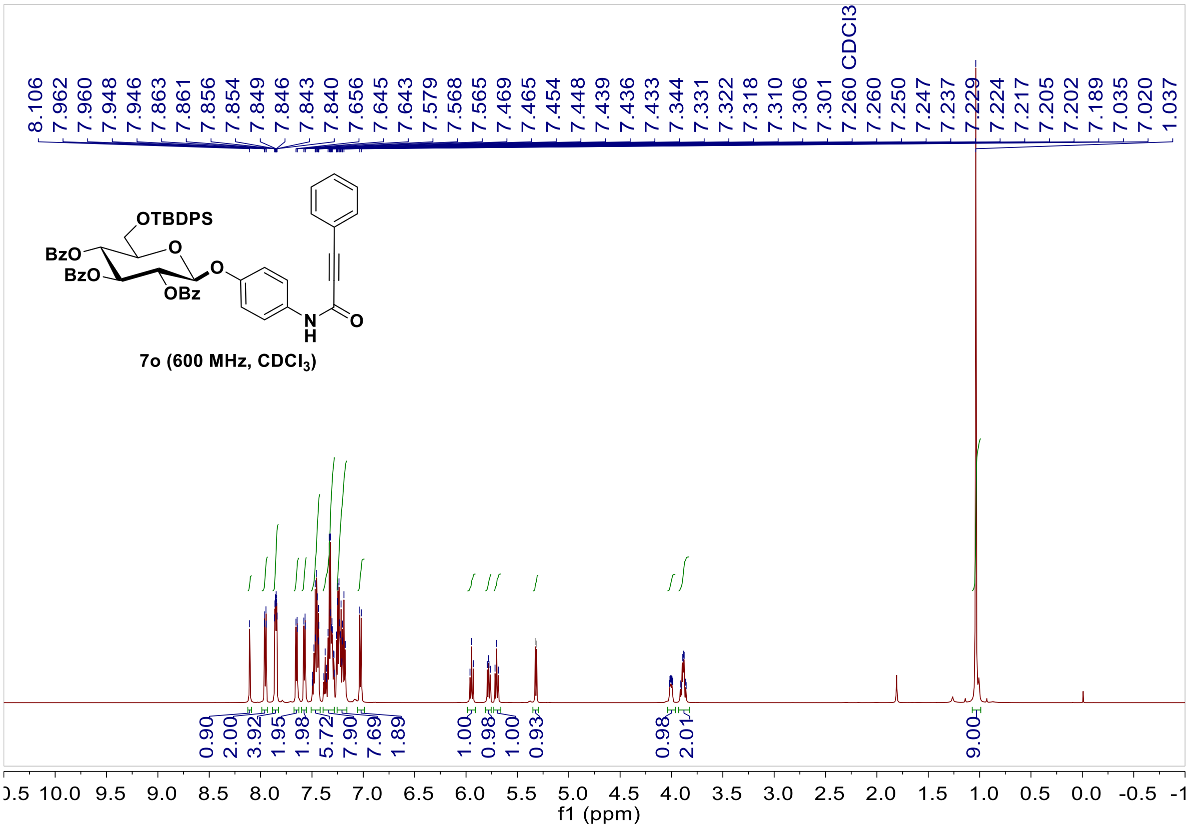

8.106
7.962
7.960
7.948
7.946
7.863
7.861
7.856
7.854
7.849
7.846
7.843
7.840
7.656
7.645
7.643
7.579
7.568
7.565
7.469
7.465
7.454
7.448
7.439
7.436
7.433
7.344
7.331
7.322
7.318
7.310
7.306
7.301
7.260 CDCl3
7.260
7.250
7.247
7.237
7.229
7.224
7.217
7.205
7.202
7.189
7.035
7.020
1.037
OTBDPS
BzO
BzO
O
O
OBz
N
H
O
7o (600 MHz, CDCl$_3$)
0.90
2.00
3.92
1.95
1.98
5.72
7.90
7.69
1.89
1.00
0.98
1.00
0.93
0.98
2.01
9.00
f1 (ppm)

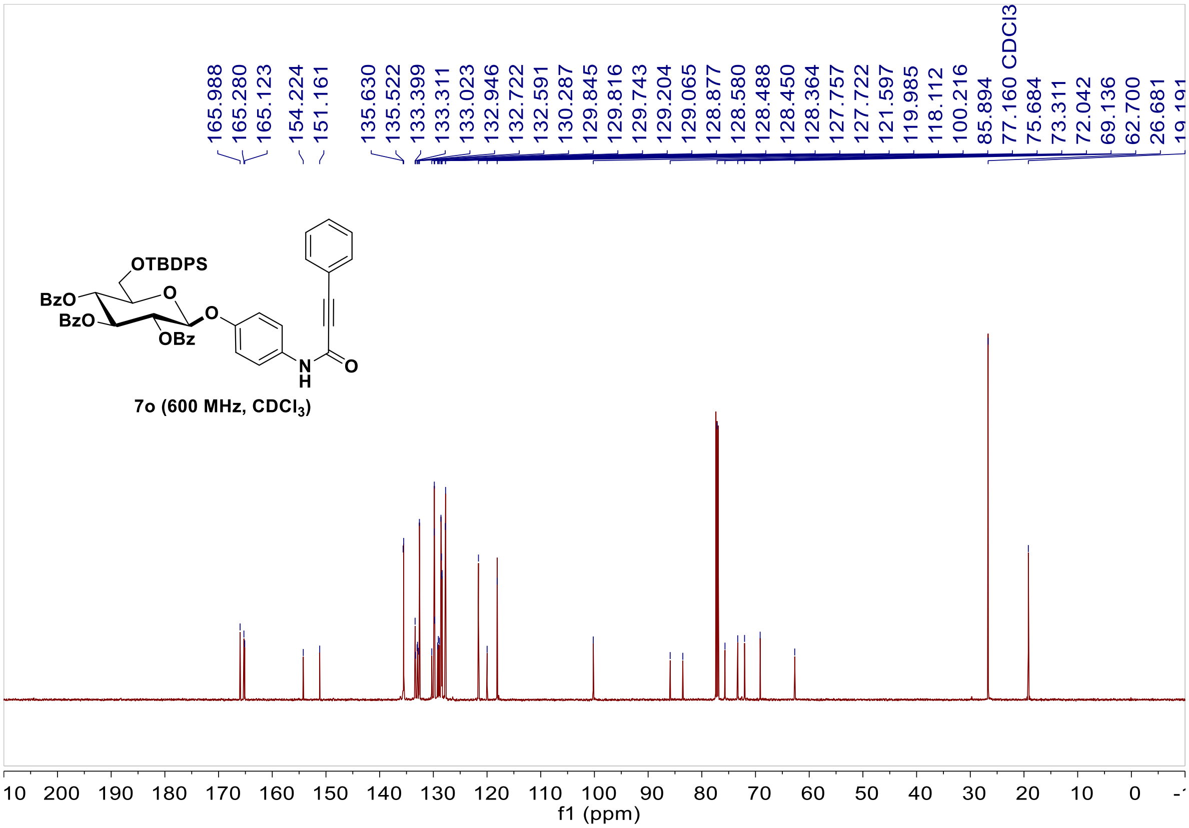

165.988
165.280
165.123
154.224
151.161
135.630
135.522
133.399
133.311
133.023
132.946
132.722
132.591
130.287
129.845
129.816
129.743
129.204
129.065
128.877
128.580
128.488
128.450
128.364
127.757
127.722
121.597
119.985
118.112
100.216
85.894
77.160 CDCl3
75.684
73.311
72.042
69.136
62.700
26.681
OTBDPS
BzO
BzO
O
O
OBz
N
H
O
7o (600 MHz, CDCl$_3$)
f1 (ppm)

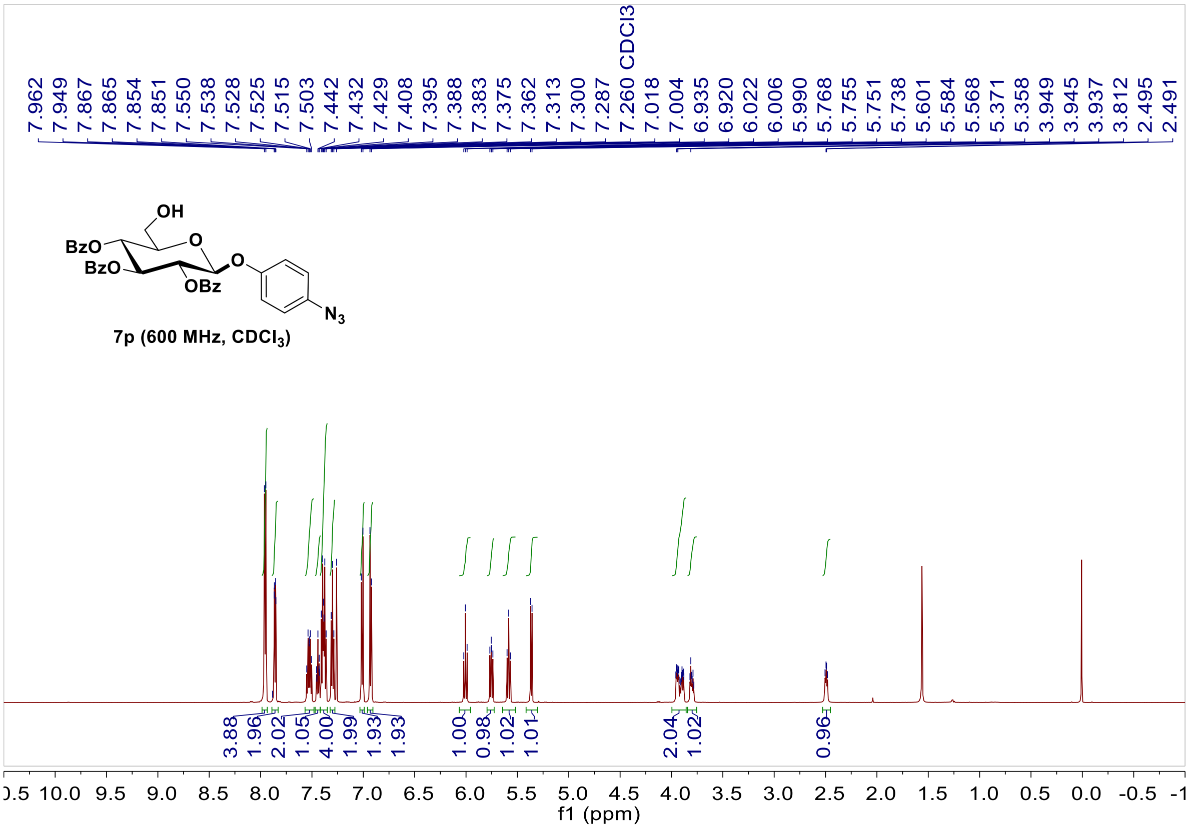
7p (600 MHz, CDCl3)
f1 (ppm)

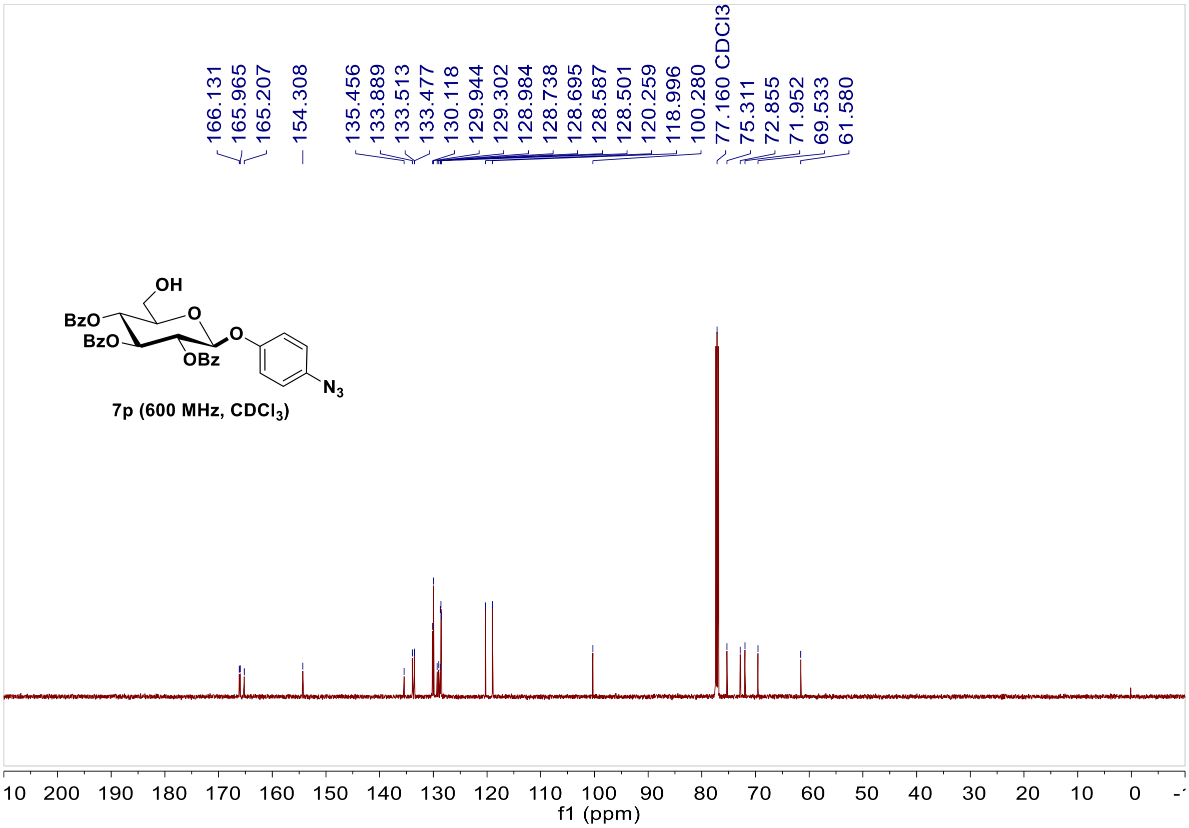
7p (600 MHz, CDCl3)
f1 (ppm)

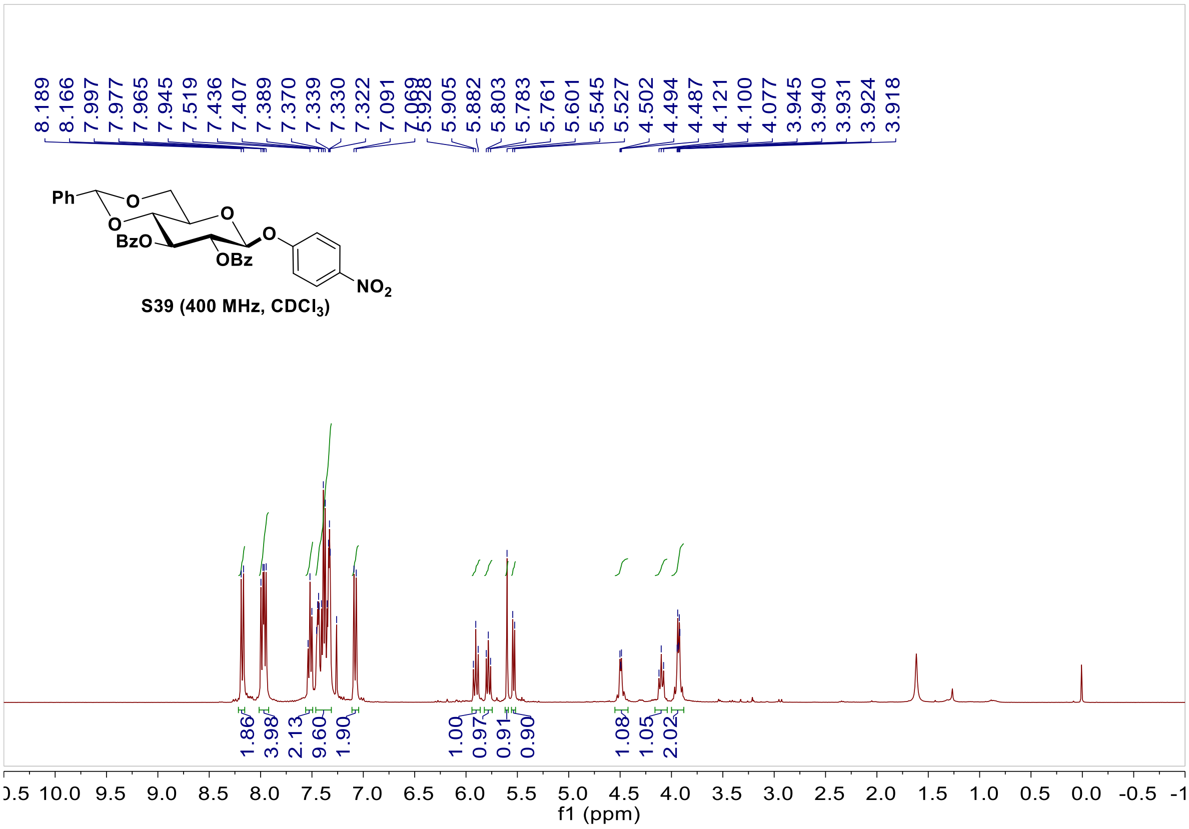
8.189
8.166
7.997
7.977
7.965
7.945
7.519
7.436
7.407
7.389
7.370
7.339
7.330
7.322
7.091
7.069
5.928
5.905
5.882
5.803
5.783
5.761
5.601
5.545
5.527
4.502
4.494
4.487
4.121
4.100
4.077
3.945
3.940
3.931
3.924
3.918
Ph
O
O
O
O
BzO
OBz
NO2
S39 (400 MHz, CDCl3)
1.86
3.98
2.13
9.60
1.90
1.00
0.97
0.91
0.90
1.08
1.05
2.02
f1 (ppm)

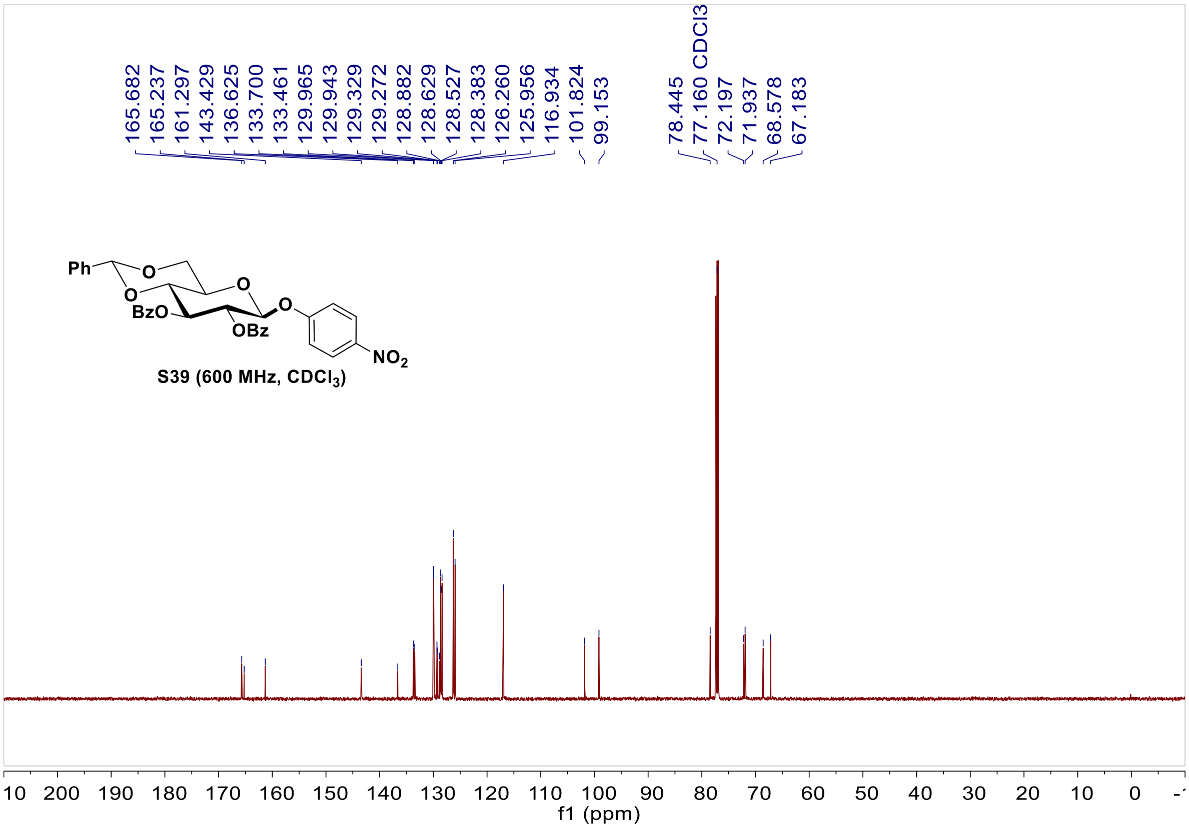
165.682
165.237
161.297
143.429
136.625
133.700
133.461
129.965
129.943
129.329
129.272
128.882
128.629
128.527
128.383
126.260
125.956
116.934
101.824
99.153
78.445
77.160 CDCl3
72.197
71.937
68.578
67.183
Ph
O
O
O
O
BzO
OBz
NO2
S39 (600 MHz, CDCl3)
f1 (ppm)

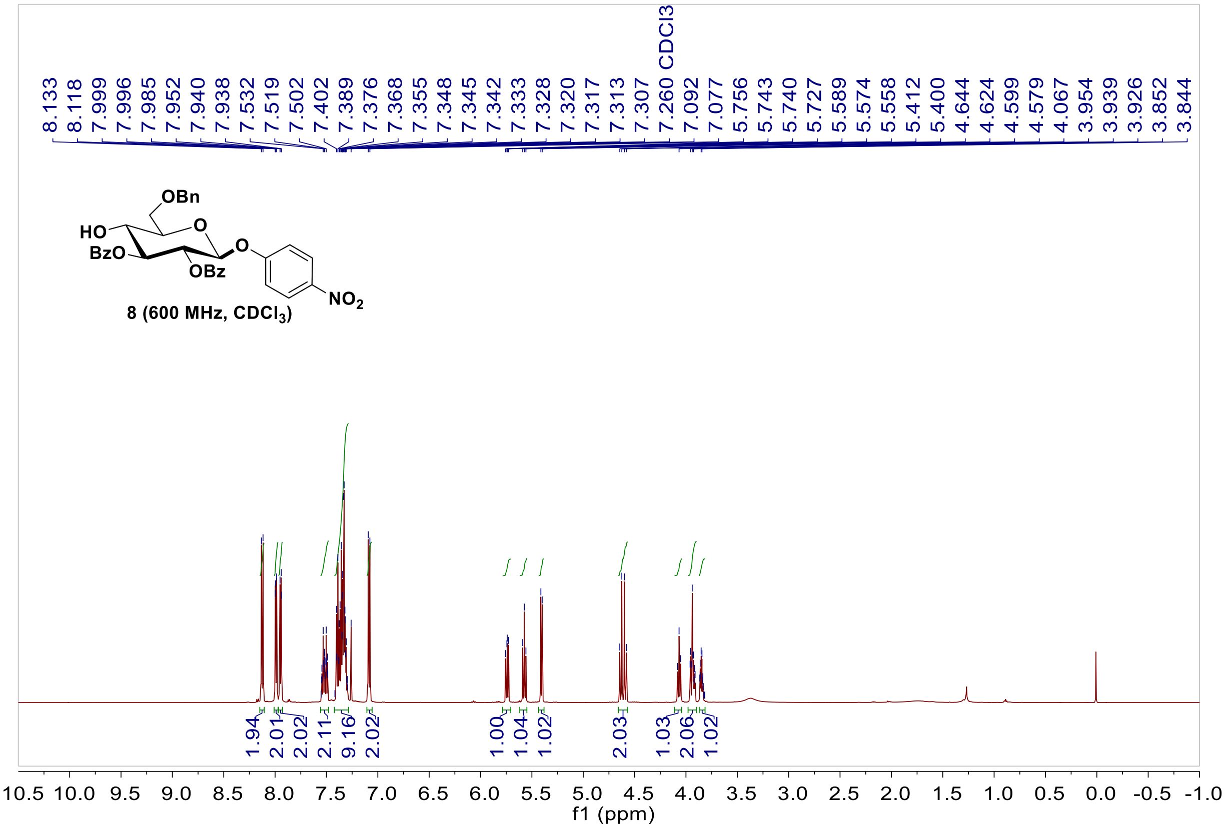
8.133
8.118
7.999
7.996
7.985
7.952
7.940
7.938
7.532
7.519
7.502
7.402
7.389
7.376
7.368
7.355
7.348
7.345
7.342
7.333
7.328
7.320
7.317
7.313
7.307
7.260 CDCl3
7.092
7.077
5.756
5.743
5.740
5.727
5.589
5.574
5.558
5.412
5.400
4.644
4.624
4.599
4.579
4.067
3.954
3.939
3.926
3.852
3.844
OBn
HO
BzO
O
OBz
O
NO2
8 (600 MHz, CDCl3)
1.94
2.01
2.02
2.11
9.16
2.02
1.00
1.04
1.02
2.03
1.03
2.06
1.02
10.5 10.0 9.5 9.0 8.5 8.0 7.5 7.0 6.5 6.0 5.5 5.0 4.5 4.0 3.5 3.0 2.5 2.0 1.5 1.0 0.5 0.0 -0.5 -1.0
f1 (ppm)

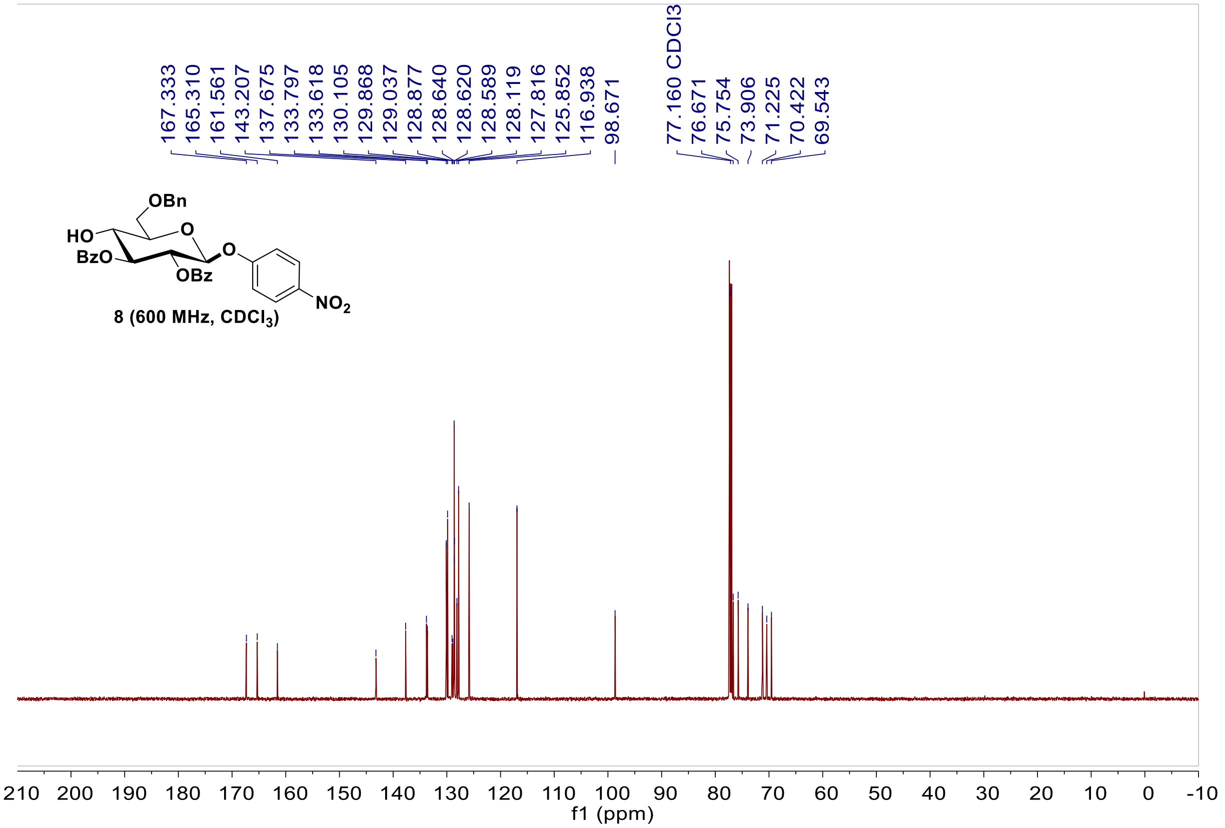
167.333
165.310
161.561
143.207
137.675
133.797
133.618
130.105
129.868
129.037
128.877
128.640
128.620
128.589
128.119
127.816
125.852
116.938
98.671
77.160 CDCl3
76.671
75.754
73.906
71.225
70.422
69.543
OBn
HO
BzO
O
OBz
O
NO2
8 (600 MHz, CDCl3)
210 200 190 180 170 160 150 140 130 120 110 100 90 80 70 60 50 40 30 20 10 0 -10
f1 (ppm)

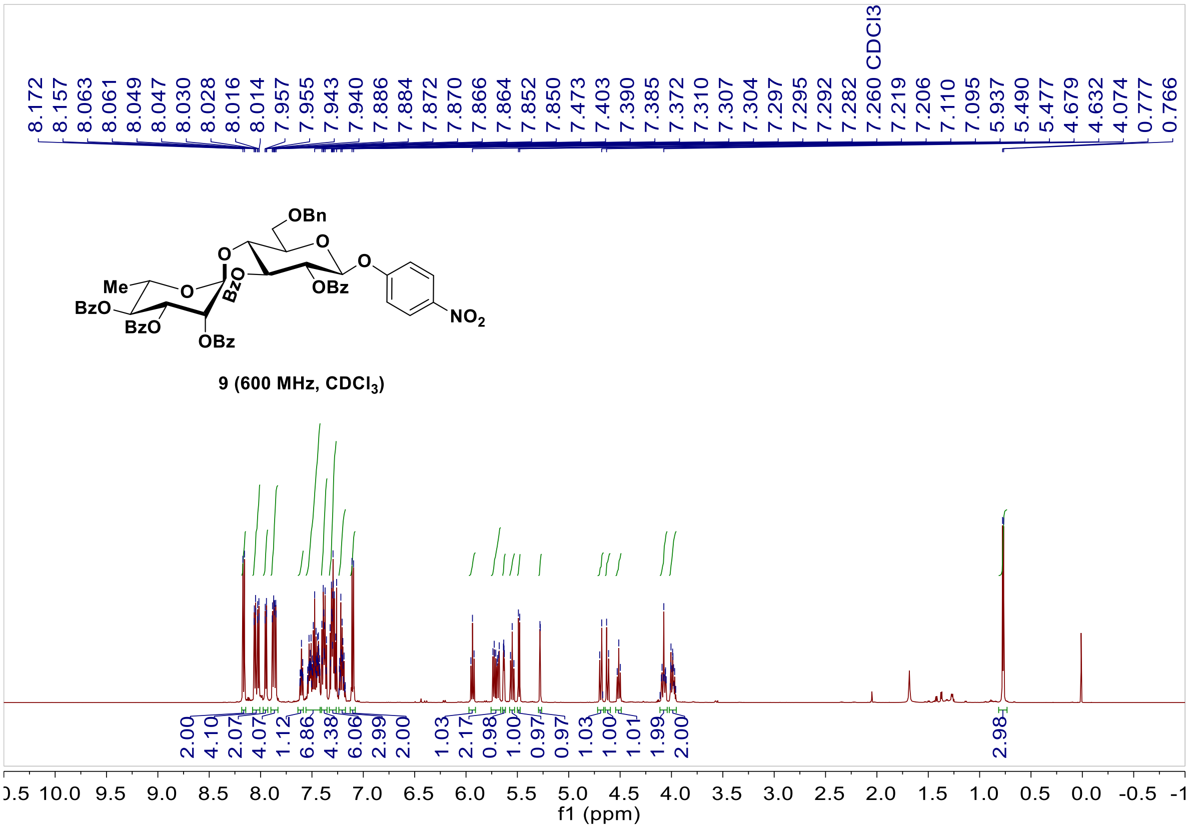
8.172 8.157 8.063 8.061 8.049 8.047 8.030 8.028 8.016 8.014 7.957 7.955 7.943 7.940 7.886 7.884 7.872 7.870 7.866 7.864 7.852 7.850 7.473 7.403 7.390 7.385 7.372 7.310 7.307 7.304 7.297 7.295 7.292 7.282 7.260 CDCl3 7.219 7.206 7.110 7.095 5.937 5.490 5.477 4.679 4.632 4.074 0.777 0.766
OBn
Me
BzO
BzO
OBz
BzO
OBz
NO2
9 (600 MHz, CDCl3)
2.00 4.10 2.07 4.07 1.12 6.86 4.38 6.06 2.99 2.00 1.03 2.17 0.98 1.00 0.97 0.97 1.03 1.00 1.01 1.99 2.00 2.98
f1 (ppm)
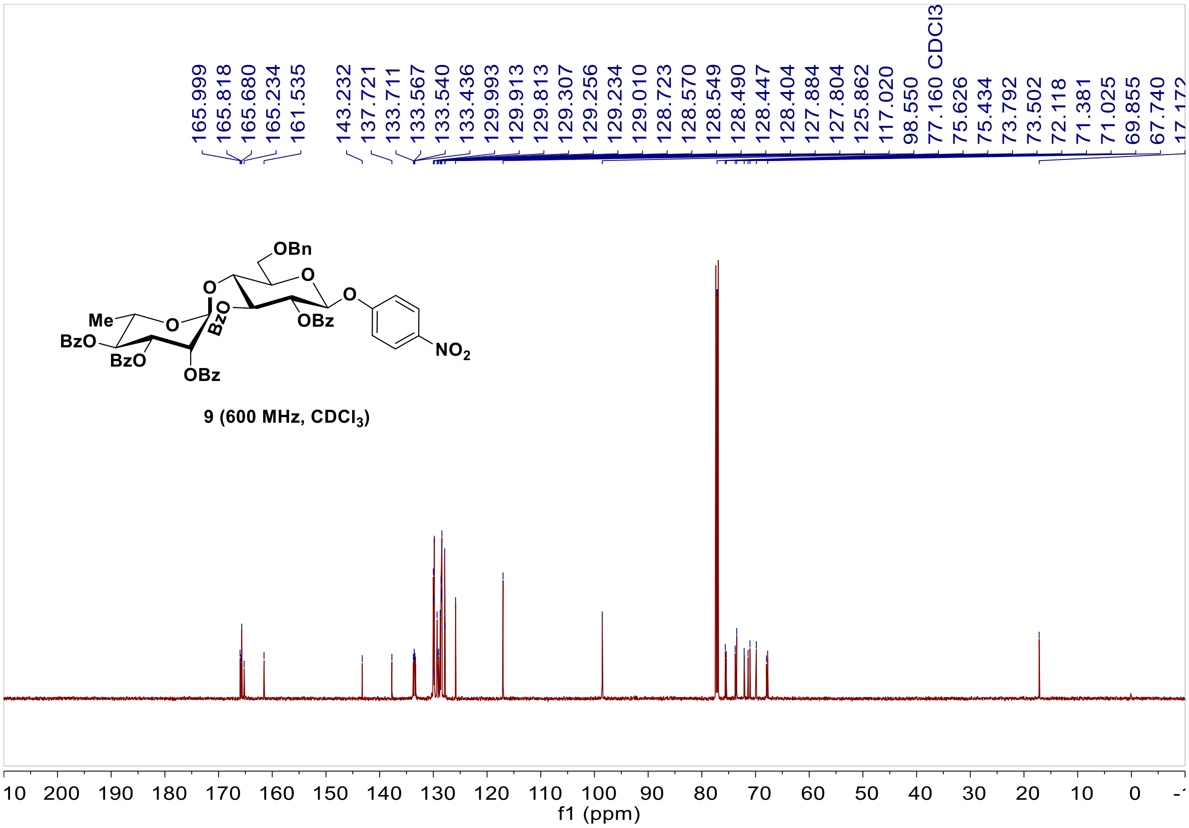
165.999 165.818 165.680 165.234 161.535 143.232 137.721 133.711 133.567 133.540 133.436 129.993 129.913 129.813 129.307 129.256 129.234 129.010 128.723 128.570 128.549 128.490 128.447 128.404 127.884 127.804 125.862 117.020 98.550 77.160 CDCl3 75.626 75.434 73.792 73.502 72.118 71.381 71.025 69.855 67.740
OBn
Me
BzO
BzO
OBz
BzO
OBz
NO2
9 (600 MHz, CDCl3)
f1 (ppm)

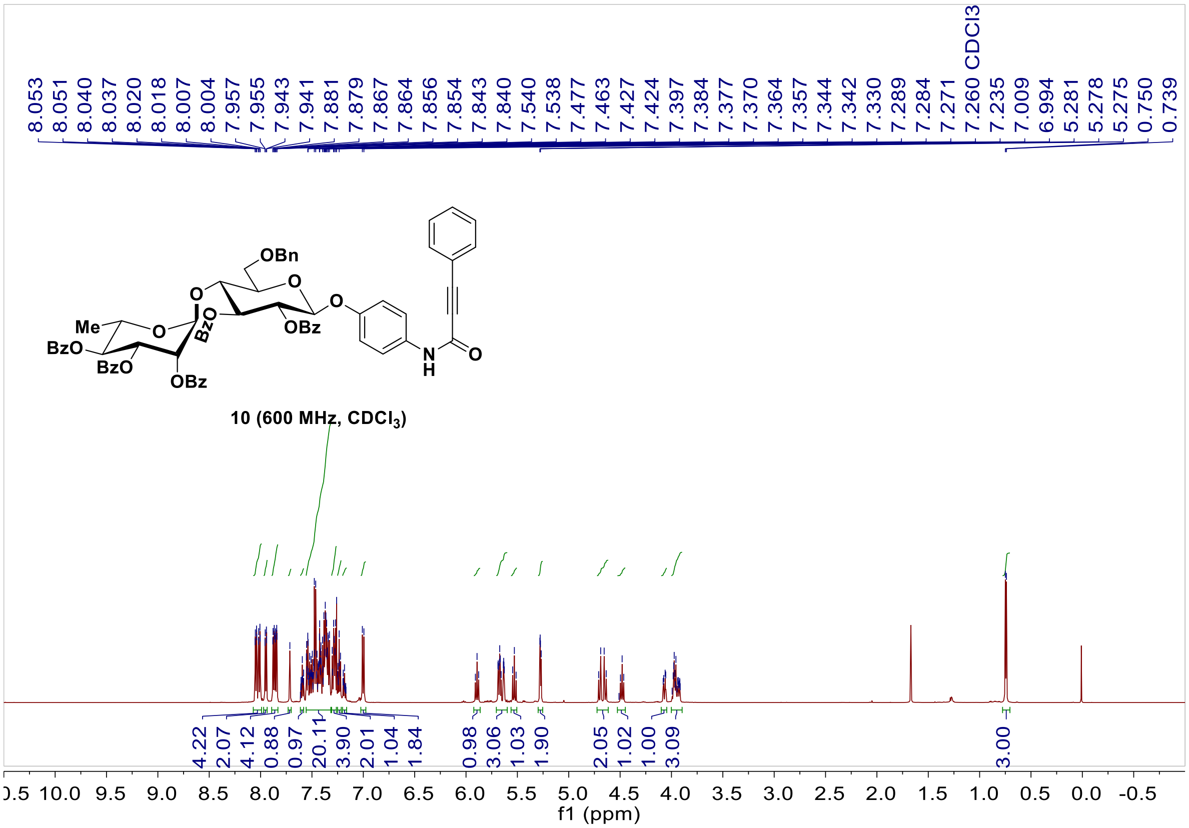
8.053
8.051
8.040
8.037
8.020
8.018
8.007
8.004
7.957
7.955
7.943
7.941
7.881
7.879
7.867
7.864
7.856
7.854
7.843
7.840
7.540
7.538
7.477
7.463
7.427
7.424
7.397
7.384
7.377
7.370
7.364
7.357
7.344
7.342
7.330
7.289
7.284
7.271
7.260 CDCl3
7.235
7.009
6.994
5.281
5.278
5.275
0.750
0.739
OBn
O
O
O
Me
O
BzO
BzO
OBz
BzO
OBz
O
N
H
O
10 (600 MHz, CDCl3)
4.22
2.07
4.12
0.88
0.97
20.11
3.90
2.01
1.04
1.84
0.98
3.06
1.03
1.90
2.05
1.02
1.00
3.09
3.00
10.5 10.0 9.5 9.0 8.5 8.0 7.5 7.0 6.5 6.0 5.5 5.0 4.5 4.0 3.5 3.0 2.5 2.0 1.5 1.0 0.5 0.0 -0.5
f1 (ppm)

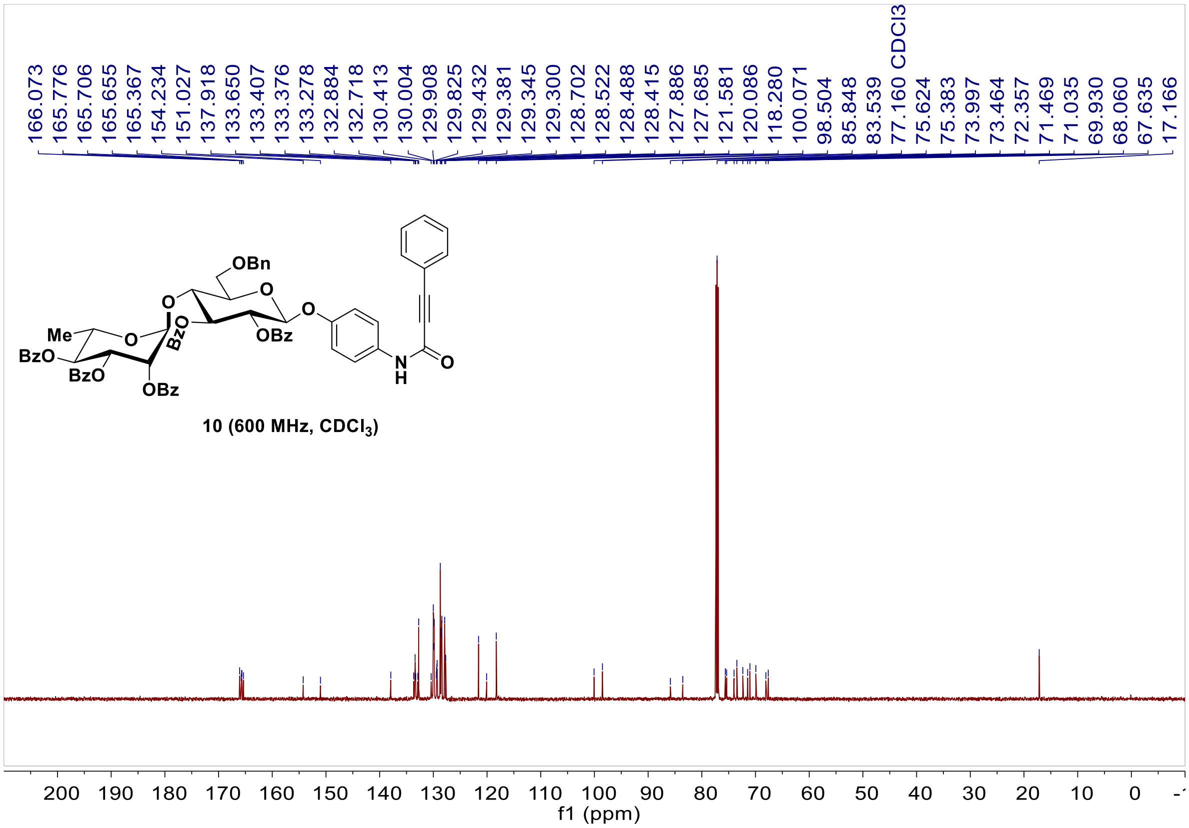
166.073
165.776
165.706
165.655
165.367
154.234
151.027
137.918
133.650
133.407
133.376
133.278
132.884
132.718
130.413
130.004
129.908
129.825
129.432
129.381
129.345
129.300
128.702
128.522
128.488
128.415
127.886
127.685
121.581
120.086
118.280
100.071
98.504
85.848
83.539
77.160 CDCl3
75.624
75.383
73.997
73.464
72.357
71.469
71.035
69.930
68.060
67.635
17.166
OBn
O
O
O
Me
O
BzO
BzO
OBz
BzO
OBz
O
N
H
O
10 (600 MHz, CDCl3)
200 190 180 170 160 150 140 130 120 110 100 90 80 70 60 50 40 30 20 10 0
f1 (ppm)

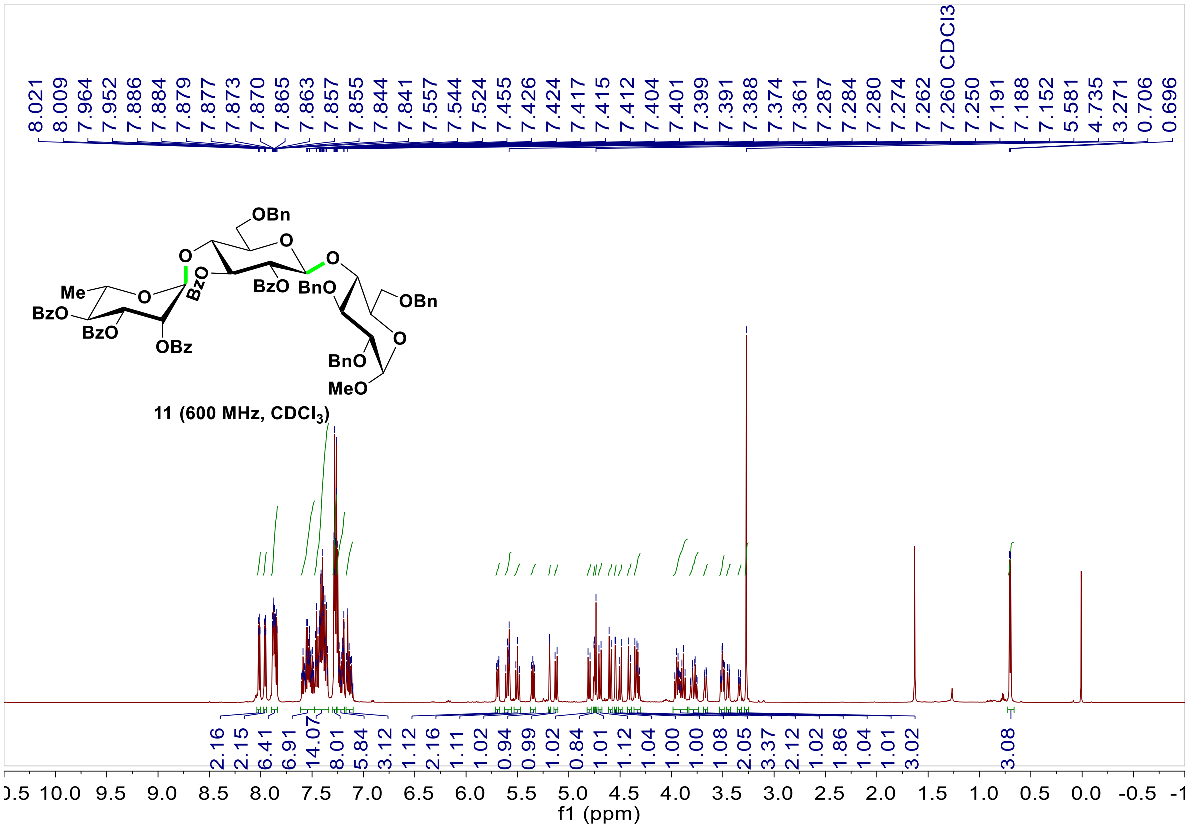

8.021
8.009
7.964
7.952
7.886
7.884
7.879
7.877
7.873
7.870
7.865
7.863
7.857
7.855
7.844
7.841
7.557
7.544
7.524
7.455
7.426
7.424
7.417
7.415
7.412
7.404
7.401
7.399
7.391
7.388
7.374
7.361
7.287
7.284
7.280
7.274
7.262
7.260 CDCl3
7.250
7.191
7.188
7.152
5.581
4.735
3.271
0.706
0.696
OBn
O
O
BzO
O
O
Me
O
BzO
BzO
BnO
OBn
BzO
BzO
OBz
BnO
O
MeO
11 (600 MHz, CDCl3)
2.16
2.15
6.41
6.91
14.07
8.01
5.84
3.12
1.12
2.16
1.11
1.02
0.94
0.99
1.02
0.84
1.01
1.12
1.04
1.00
1.00
1.08
2.05
3.37
2.12
1.02
1.86
1.04
1.01
3.02
3.08
0.5 10.0 9.5 9.0 8.5 8.0 7.5 7.0 6.5 6.0 5.5 5.0 4.5 4.0 3.5 3.0 2.5 2.0 1.5 1.0 0.5 0.0 -0.5 -1
f1 (ppm)

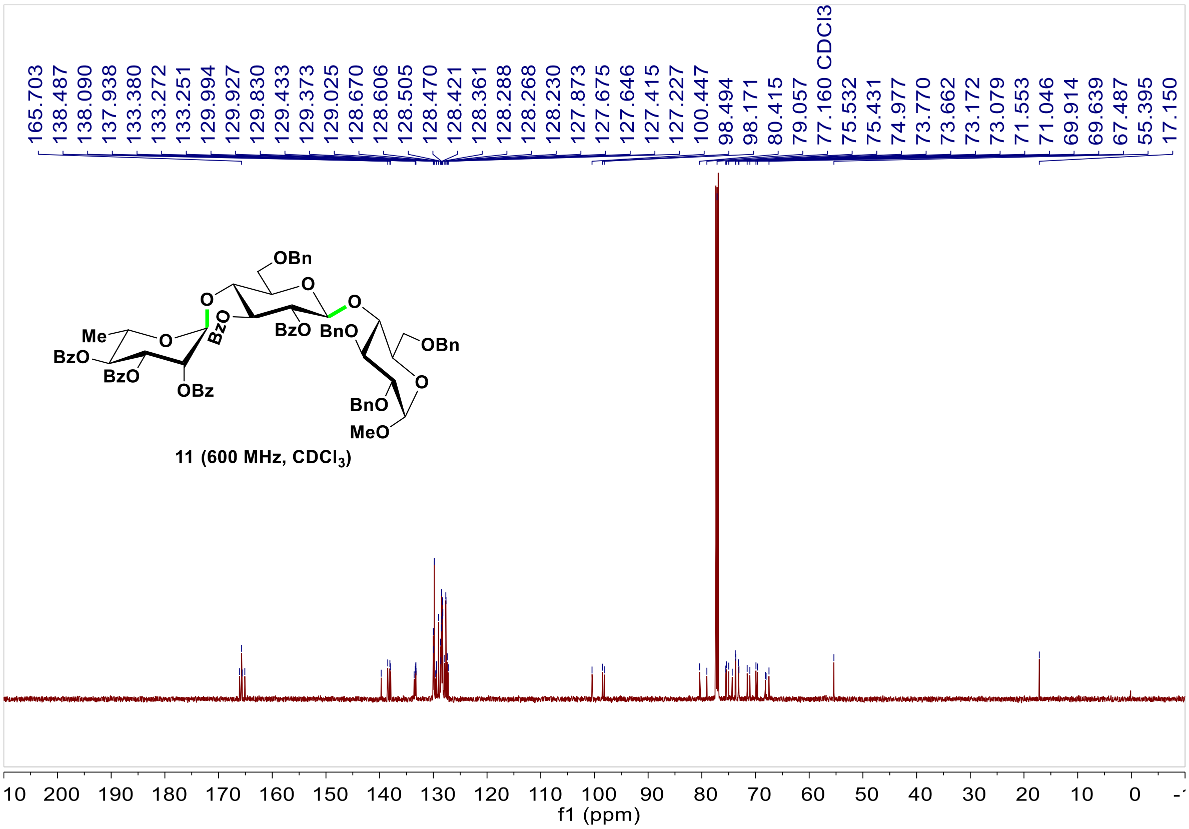

165.703
138.487
138.090
137.938
133.380
133.272
133.251
129.994
129.927
129.830
129.433
129.373
129.025
128.670
128.606
128.505
128.470
128.421
128.361
128.288
128.268
128.230
127.873
127.675
127.646
127.415
127.227
100.447
98.494
98.171
80.415
79.057
77.160 CDCl3
75.532
75.431
74.977
73.770
73.662
73.172
73.079
71.553
71.046
69.914
69.639
67.487
55.395
17.150
11 (600 MHz, CDCl3)
10 200 190 180 170 160 150 140 130 120 110 100 90 80 70 60 50 40 30 20 10 0
f1 (ppm)

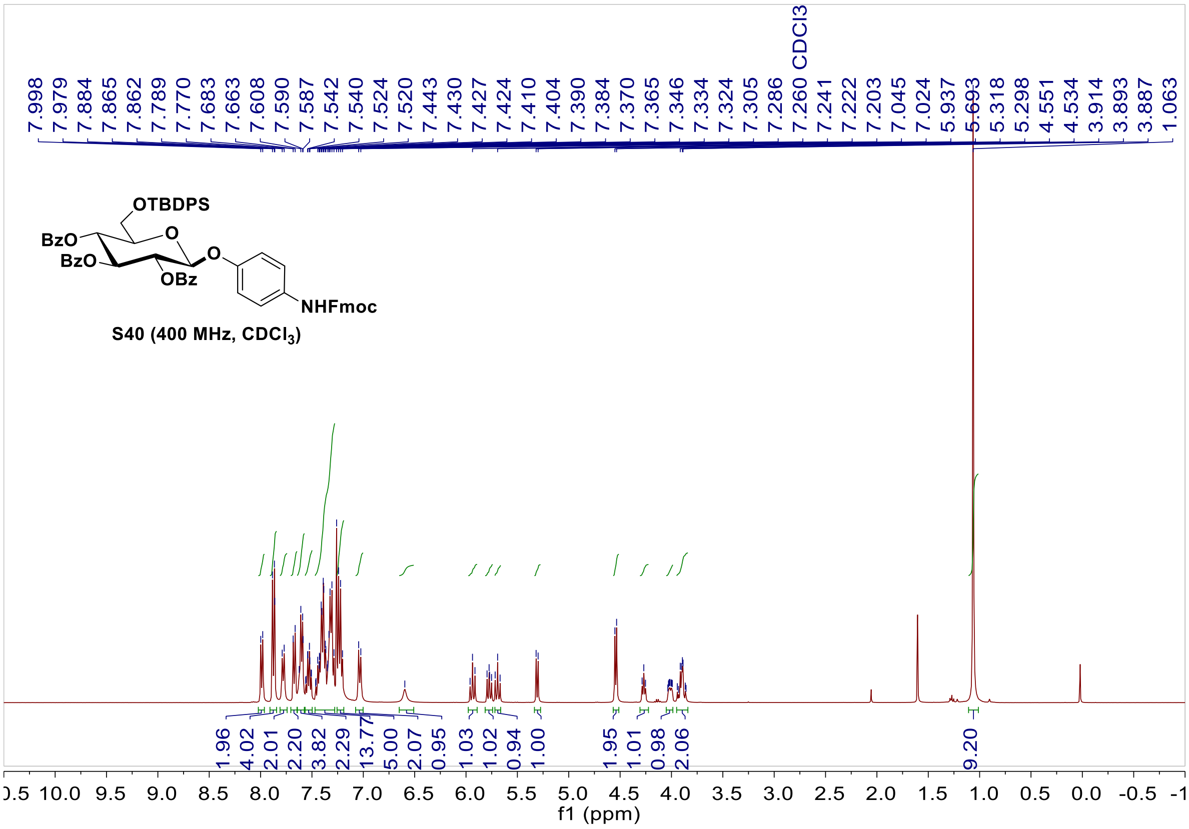
7.998
7.979
7.884
7.865
7.862
7.789
7.770
7.683
7.663
7.608
7.590
7.587
7.542
7.540
7.524
7.520
7.443
7.430
7.427
7.424
7.410
7.404
7.390
7.384
7.370
7.365
7.346
7.334
7.324
7.305
7.286
7.260 CDCl3
7.241
7.222
7.203
7.045
7.024
5.937
5.693
5.318
5.298
4.551
4.534
3.914
3.893
3.887
1.063
OTBDPS
BzO
BzO
O
O
OBz
NHFmoc
S40 (400 MHz, CDCl3)
1.96
4.02
2.01
2.20
3.82
2.29
13.77
5.00
2.07
0.95
1.03
1.02
0.94
1.00
1.95
1.01
0.98
2.06
9.20
f1 (ppm)

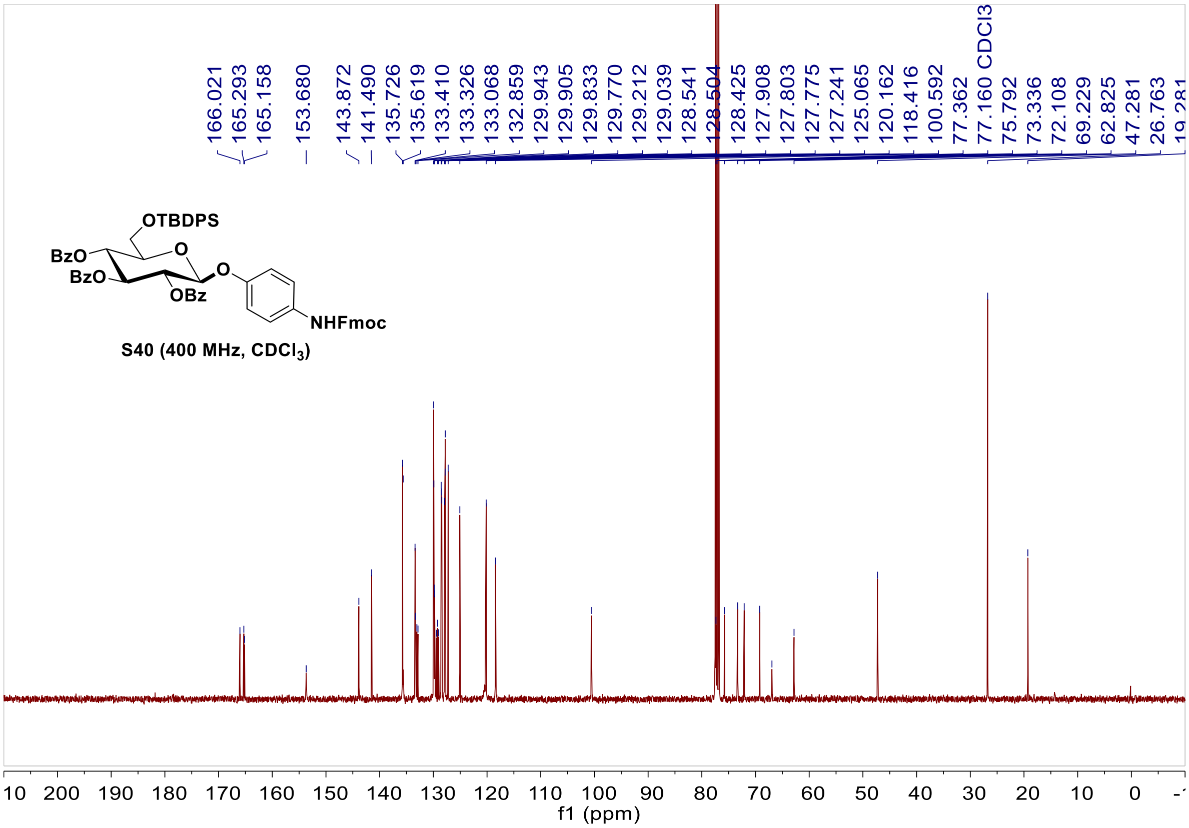
166.021
165.293
165.158
153.680
143.872
141.490
135.726
135.619
133.410
133.326
133.068
132.859
129.943
129.905
129.833
129.770
129.212
129.039
128.541
128.504
128.425
127.908
127.803
127.775
127.241
125.065
120.162
118.416
100.592
77.362
77.160 CDCl3
75.792
73.336
72.108
69.229
62.825
47.281
26.763
OTBDPS
BzO
BzO
O
O
OBz
NHFmoc
S40 (400 MHz, CDCl3)
f1 (ppm)

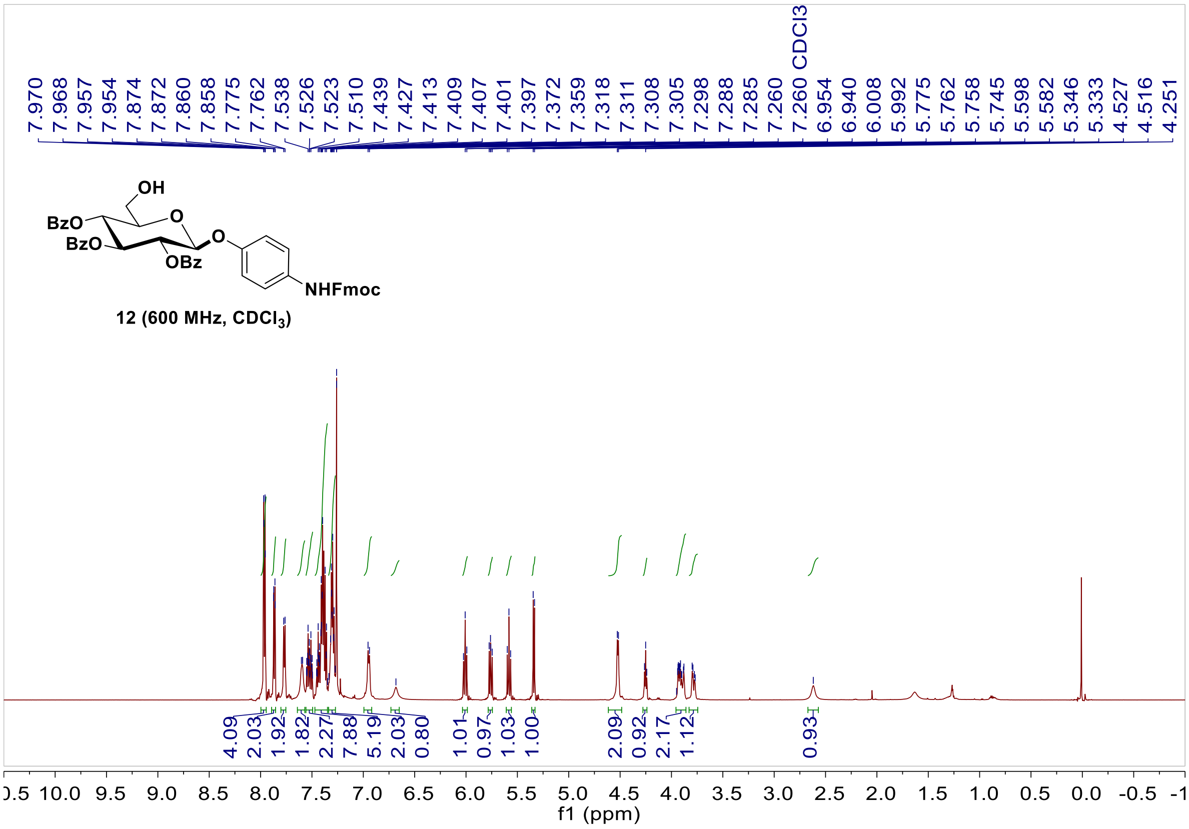
7.970
7.968
7.957
7.954
7.874
7.872
7.860
7.858
7.775
7.762
7.538
7.526
7.523
7.510
7.439
7.427
7.413
7.409
7.407
7.401
7.397
7.372
7.359
7.318
7.311
7.308
7.305
7.298
7.288
7.285
7.260
7.260 CDCl3
6.954
6.940
6.008
5.992
5.775
5.762
5.758
5.745
5.598
5.582
5.346
5.333
4.527
4.516
4.251
BzO
BzO
OBz
OH
O
O
NHFmoc
12 (600 MHz, CDCl3)
4.09
2.03
1.92
1.82
2.27
7.88
5.19
2.03
0.80
1.01
0.97
1.03
1.00
2.09
0.92
2.17
1.12
0.93
f1 (ppm)

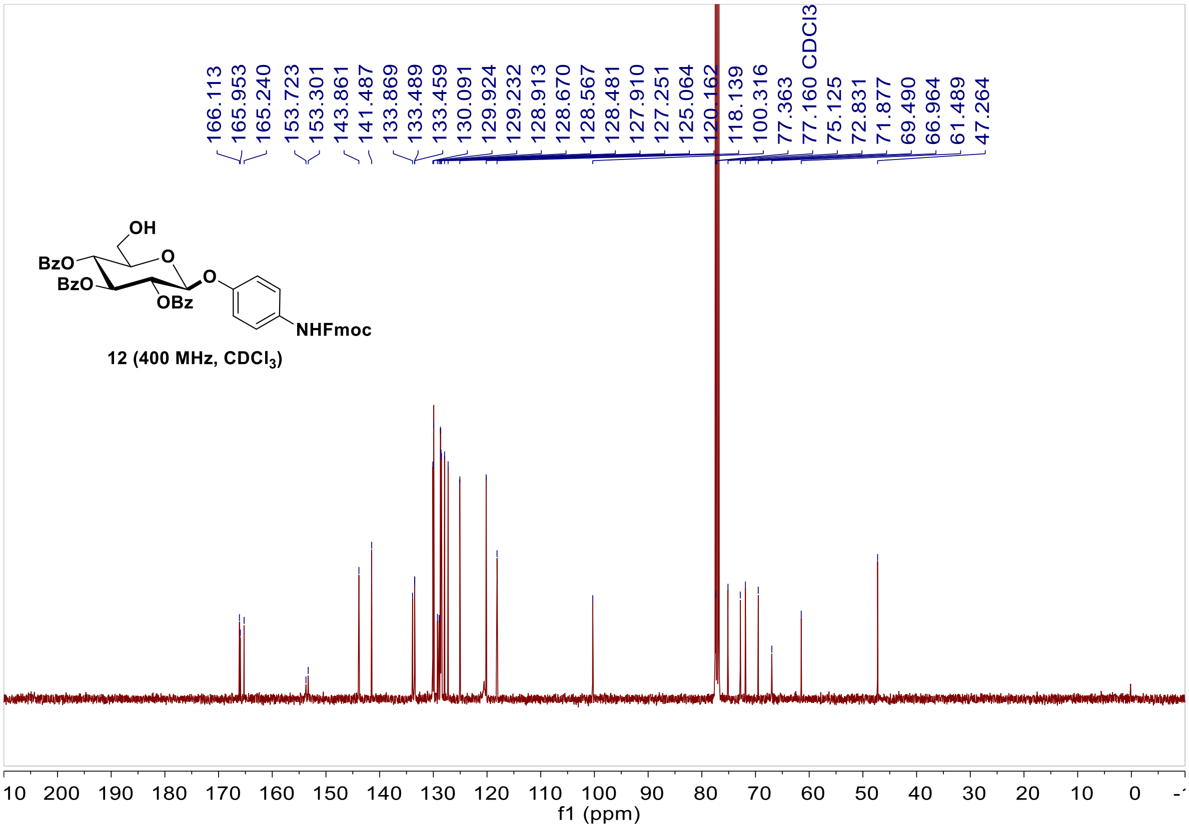
166.113
165.953
165.240
153.723
153.301
143.861
141.487
133.869
133.489
133.459
130.091
129.924
129.232
128.913
128.670
128.567
128.481
127.910
127.251
125.064
120.162
118.139
100.316
77.363
77.160 CDCl3
75.125
72.831
71.877
69.490
66.964
61.489
47.264
BzO
BzO
OBz
OH
O
O
NHFmoc
12 (400 MHz, CDCl3)
f1 (ppm)

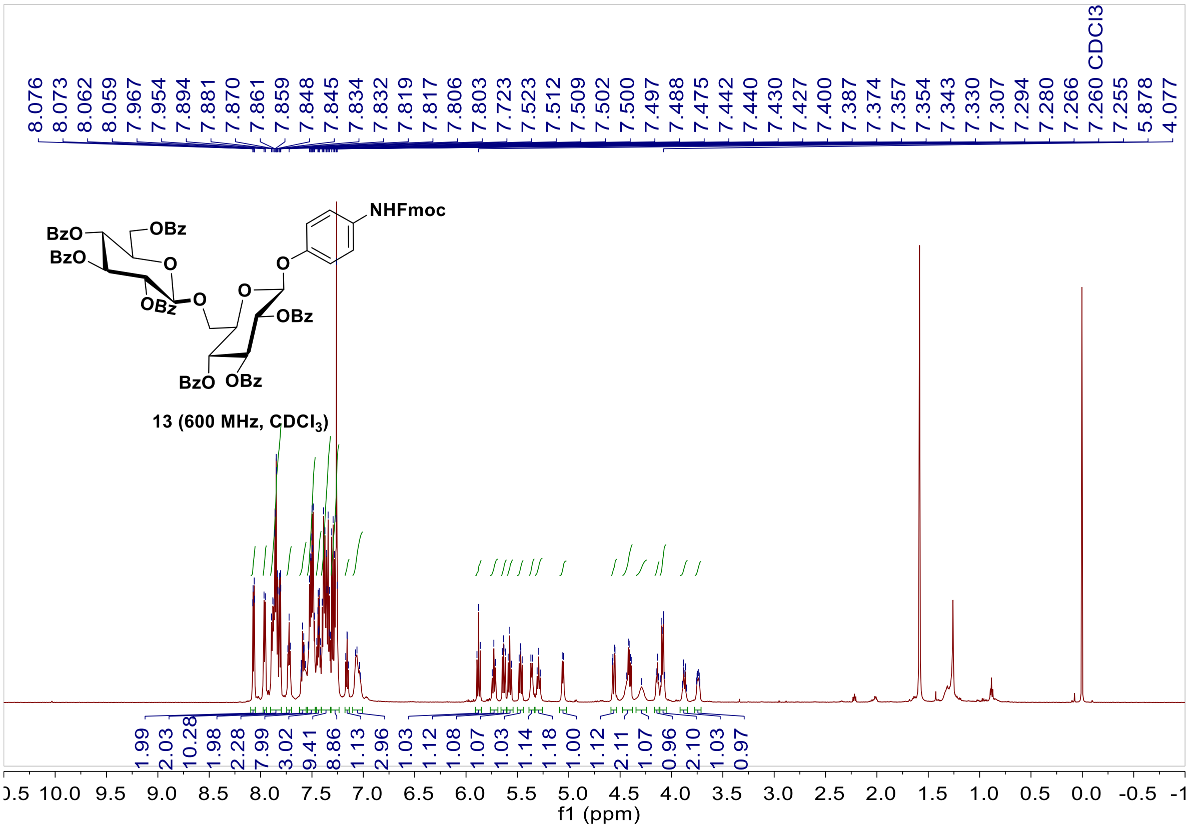
8.076
8.073
8.062
8.059
7.967
7.954
7.894
7.881
7.870
7.861
7.859
7.848
7.845
7.834
7.832
7.819
7.817
7.806
7.803
7.723
7.523
7.512
7.509
7.502
7.500
7.497
7.488
7.475
7.442
7.440
7.430
7.427
7.400
7.387
7.374
7.357
7.354
7.343
7.330
7.307
7.294
7.280
7.266
7.260 CDCl3
7.255
5.878
4.077
NHFmoc
BzO
OBz
BzO
O
O
OBz
O
O
OBz
BzO OBz
13 (600 MHz, CDCl3)
1.99
2.03
10.28
1.98
2.28
7.99
3.02
9.41
8.86
1.13
2.96
1.03
1.12
1.08
1.07
1.03
1.14
1.18
1.00
1.12
2.11
1.07
0.96
2.10
1.03
0.97
f1 (ppm)

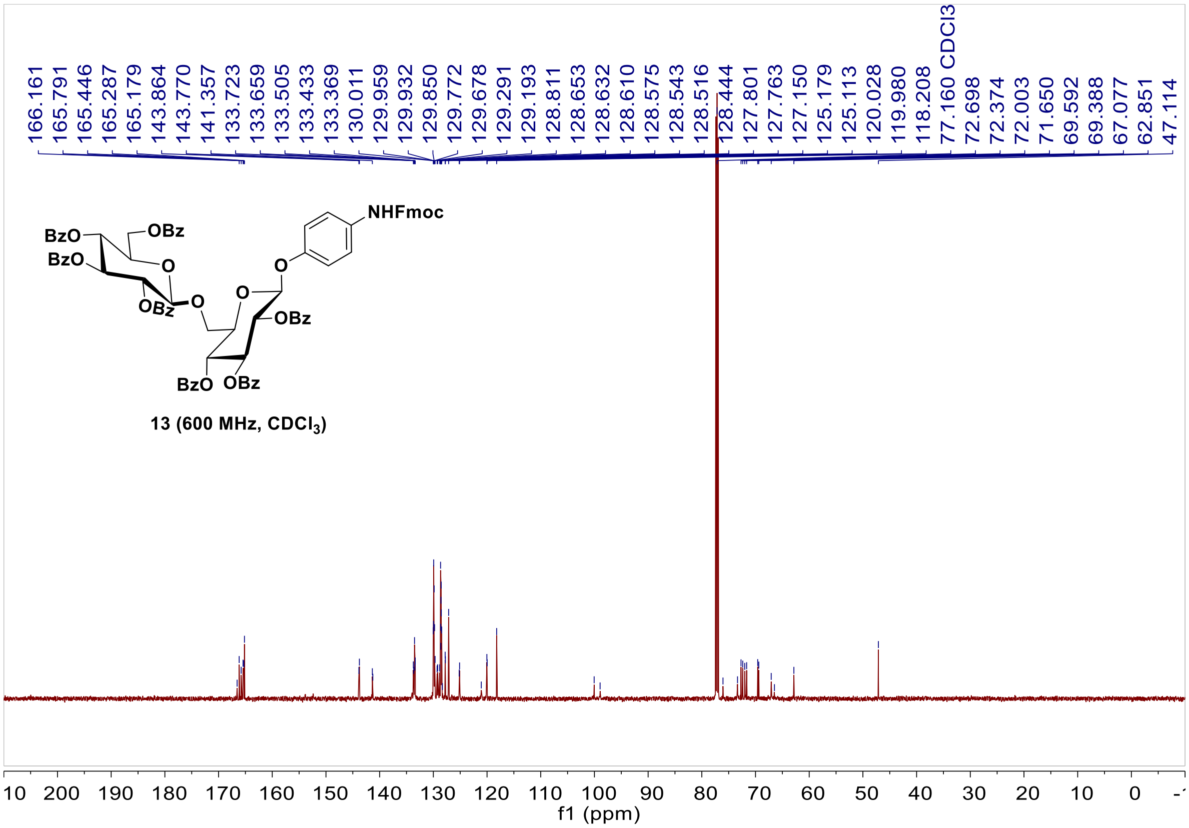
166.161
165.791
165.446
165.287
165.179
143.864
143.770
141.357
133.723
133.659
133.505
133.433
133.369
130.011
129.959
129.932
129.850
129.772
129.678
129.291
129.193
128.811
128.653
128.632
128.610
128.575
128.543
128.516
128.444
127.801
127.763
127.150
125.179
125.113
120.028
119.980
118.208
77.160 CDCl3
72.698
72.374
72.003
71.650
69.592
69.388
67.077
62.851
47.114
NHFmoc
BzO
OBz
BzO
O
O
OBz
O
O
OBz
BzO OBz
13 (600 MHz, CDCl3)
f1 (ppm)

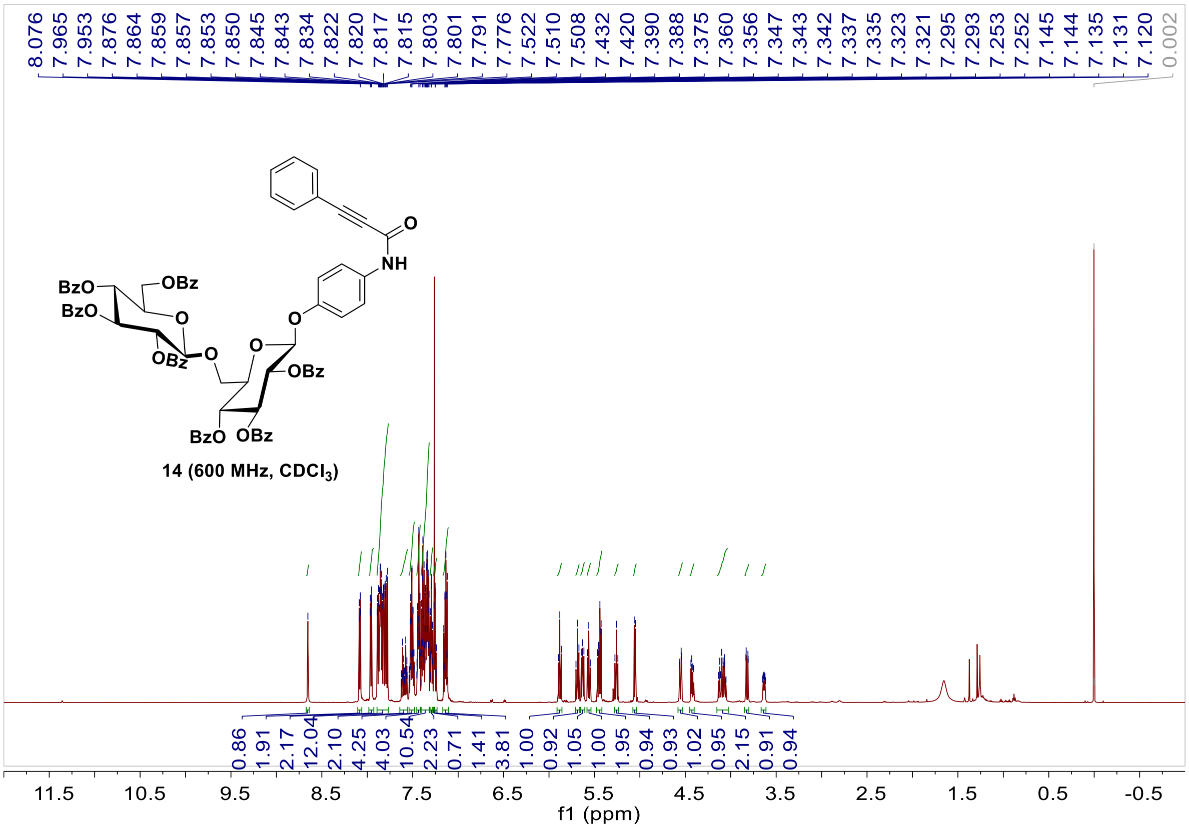
8.076 7.965 7.953 7.876 7.864 7.859 7.857 7.853 7.850 7.845 7.843 7.834 7.822 7.820 7.817 7.815 7.803 7.801 7.791 7.776 7.522 7.510 7.508 7.432 7.420 7.390 7.388 7.375 7.360 7.356 7.347 7.343 7.342 7.337 7.335 7.323 7.321 7.295 7.293 7.253 7.252 7.145 7.144 7.135 7.131 7.120 0.002
BzO OBz O BzO OBz O O OBz BzO OBz O NH O
14 (600 MHz, CDCl3)
0.86 1.91 2.17 12.04 2.10 4.25 4.03 10.54 2.23 0.71 1.41 3.81 1.00 0.92 1.05 1.00 1.95 0.94 0.93 1.02 0.95 2.15 0.91 0.94
11.5 10.5 9.5 8.5 7.5 6.5 5.5 4.5 3.5 2.5 1.5 0.5 -0.5
f1 (ppm)

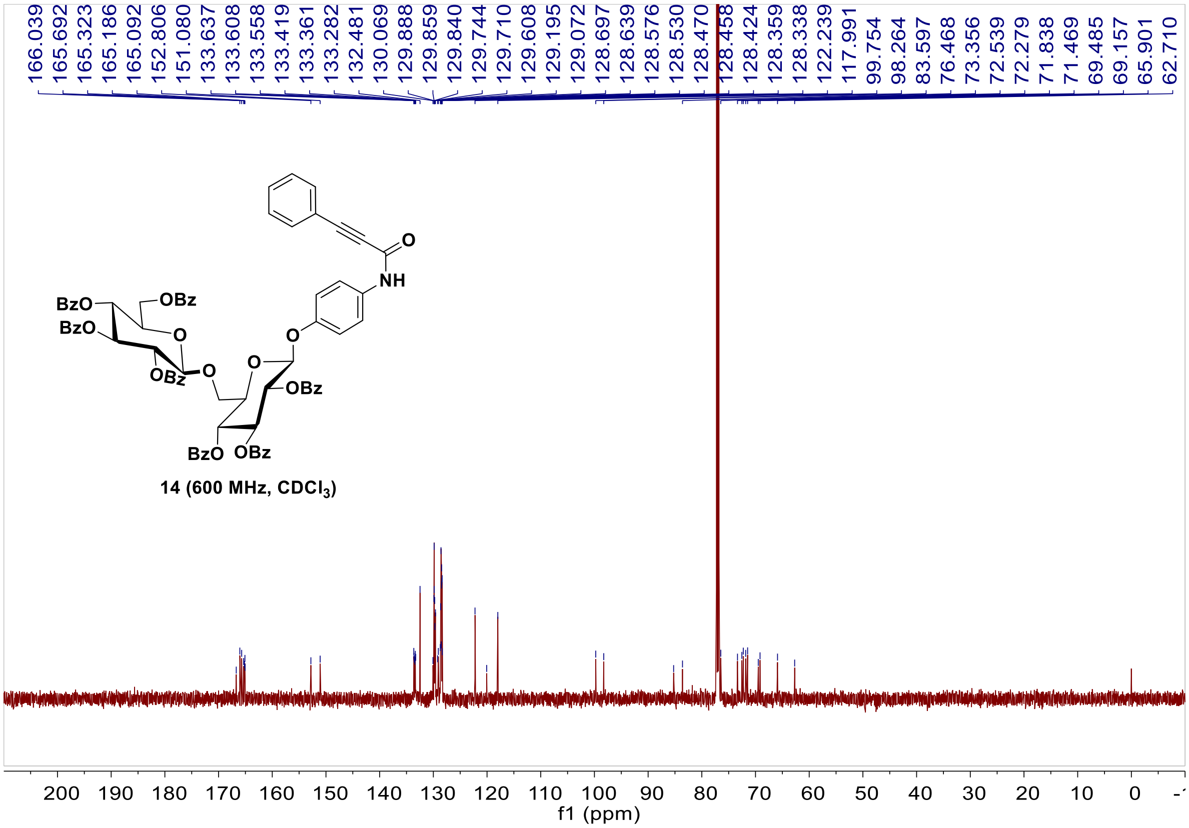
166.039 165.692 165.323 165.186 165.092 152.806 151.080 133.637 133.608 133.558 133.419 133.361 133.282 132.481 130.069 129.888 129.859 129.840 129.744 129.710 129.608 129.195 129.072 128.697 128.639 128.576 128.530 128.470 128.458 128.424 128.359 128.338 122.239 117.991 99.754 98.264 83.597 76.468 73.356 72.539 72.279 71.838 71.469 69.485 69.157 65.901 62.710
BzO OBz O BzO OBz O O OBz BzO OBz O NH O
14 (600 MHz, CDCl3)
200 190 180 170 160 150 140 130 120 110 100 90 80 70 60 50 40 30 20 10 0
f1 (ppm)

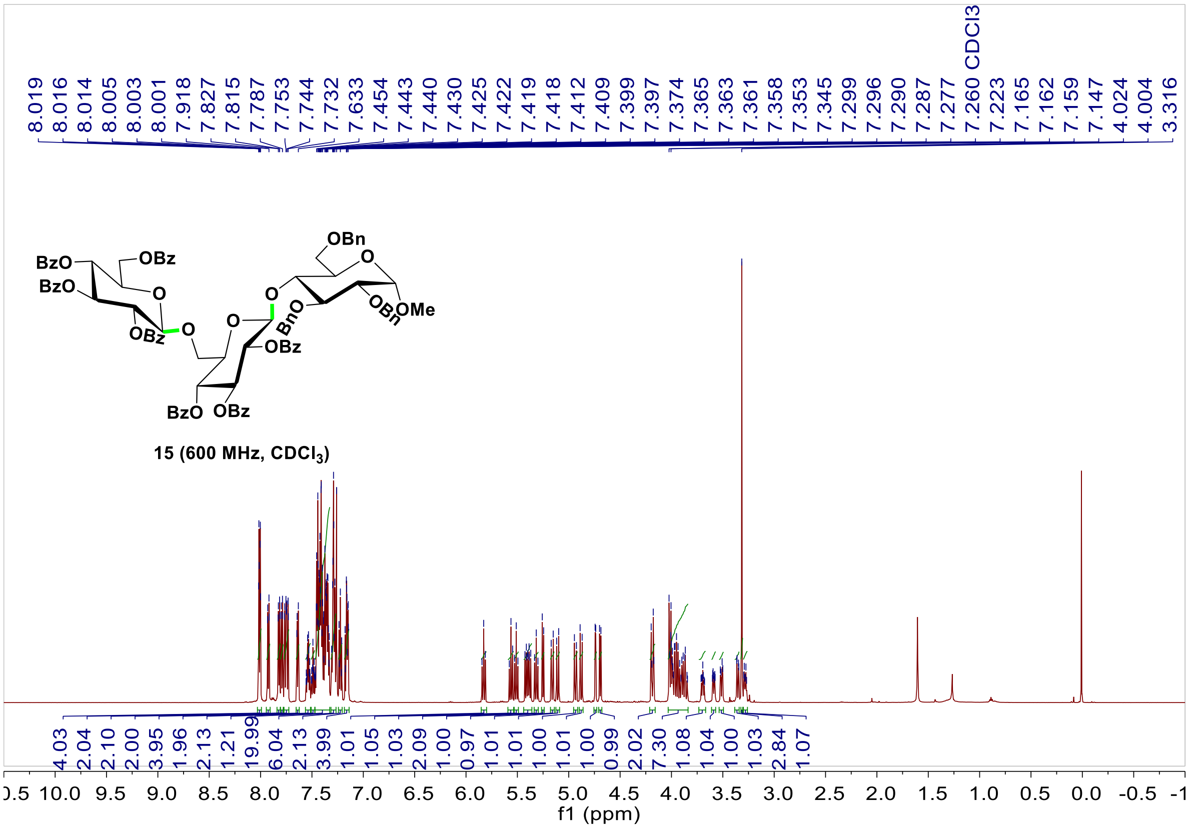
8.019 8.016 8.014 8.005 8.003 8.001 7.918 7.827 7.815 7.787 7.753 7.744 7.732 7.633 7.454 7.443 7.440 7.430 7.425 7.422 7.419 7.418 7.412 7.409 7.399 7.397 7.374 7.365 7.363 7.361 7.358 7.353 7.345 7.299 7.296 7.290 7.287 7.277 7.260 CDCl3 7.223 7.165 7.162 7.159 7.147 4.024 4.004 3.316
BzO
BzO
OBz
OBz
O
O
O
BzO
OBz
OBz
OBn
O
O
BnO
OBn
OMe
15 (600 MHz, CDCl$_3$)
4.03 2.04 2.10 2.00 3.95 1.96 2.13 1.21 19.99 6.04 2.13 3.99 1.01 1.05 1.03 2.09 1.00 0.97 1.01 1.01 1.00 1.01 1.00 0.99 2.02 7.30 1.08 1.04 1.00 1.03 2.84 1.07
f1 (ppm)

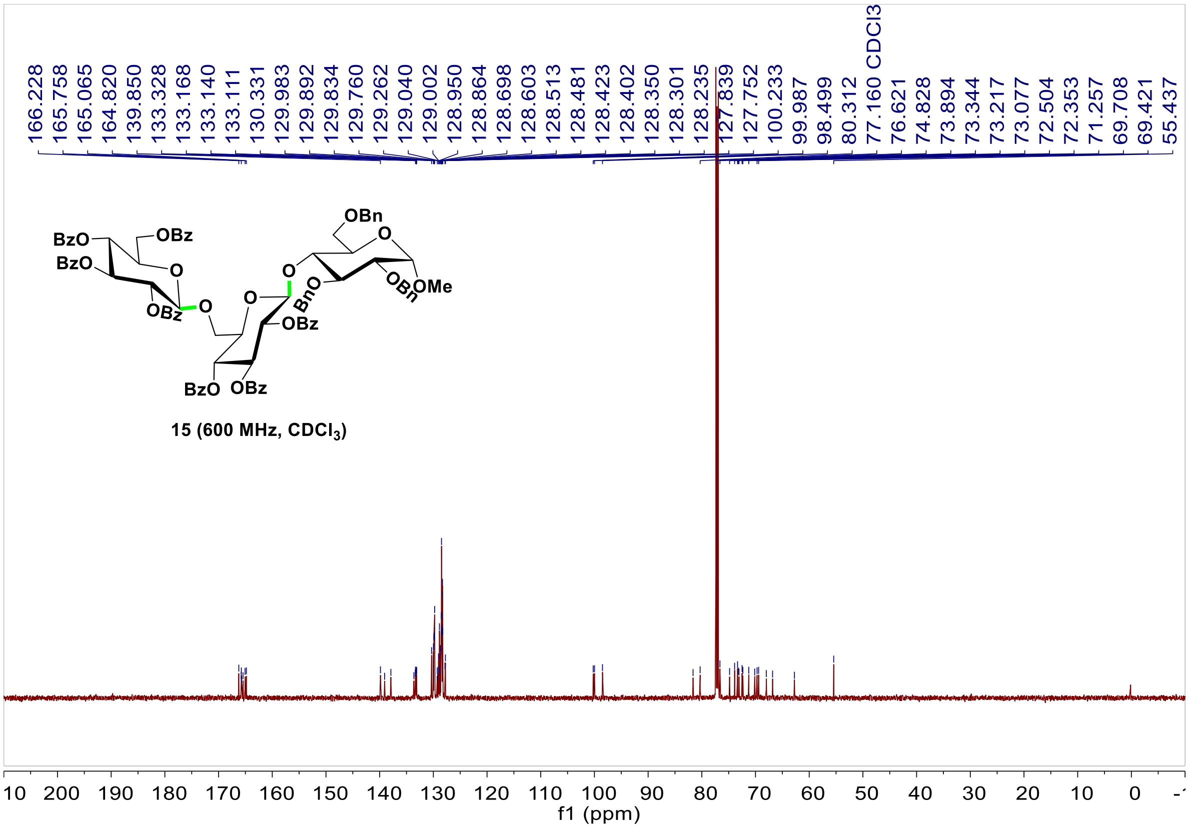
166.228 165.758 165.065 164.820 139.850 133.328 133.168 133.140 133.111 130.331 129.983 129.892 129.834 129.760 129.262 129.040 129.002 128.950 128.864 128.698 128.603 128.513 128.481 128.423 128.402 128.350 128.301 128.235 127.839 127.752 100.233 99.987 98.499 80.312 77.160 CDCl3 76.621 74.828 73.894 73.344 73.217 73.077 72.504 72.353 71.257 69.708 69.421 55.437
BzO
BzO
OBz
OBz
O
O
O
BzO
OBz
OBz
OBn
O
O
BnO
OBn
OMe
15 (600 MHz, CDCl$_3$)
f1 (ppm)

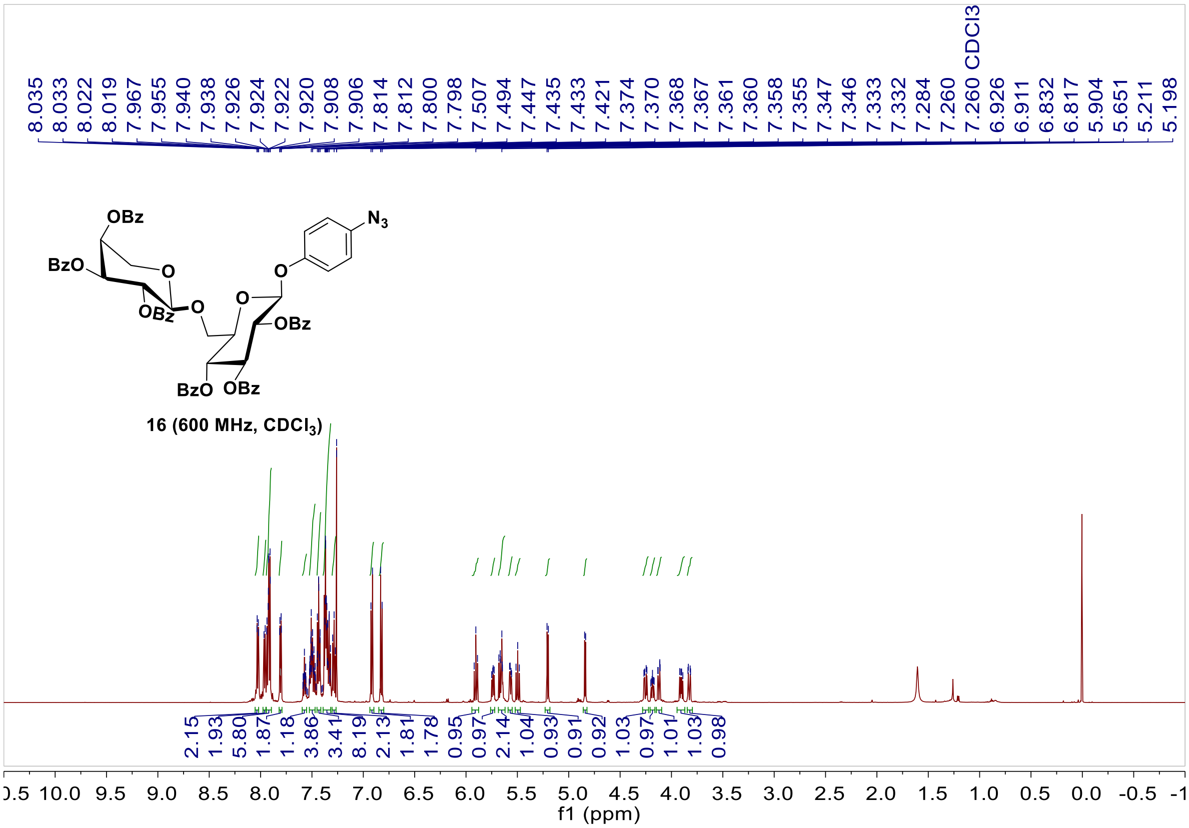
8.035 8.033 8.022 8.019 7.967 7.955 7.940 7.938 7.926 7.924 7.922 7.920 7.908 7.906 7.814 7.812 7.800 7.798 7.507 7.494 7.447 7.435 7.433 7.421 7.374 7.370 7.368 7.367 7.361 7.360 7.358 7.355 7.347 7.346 7.333 7.332 7.284 7.260 7.260 CDCl3 6.926 6.911 6.832 6.817 5.904 5.651 5.211 5.198
OBz
BzO
O
OBz
O
O
OBz
BzO OBz
O
N3
16 (600 MHz, CDCl3)
2.15 1.93 5.80 1.87 1.18 3.86 3.41 8.19 2.13 1.81 1.78 0.95 0.97 2.14 1.04 0.93 0.91 0.92 1.03 0.97 1.01 1.03 0.98
10.0 9.5 9.0 8.5 8.0 7.5 7.0 6.5 6.0 5.5 5.0 4.5 4.0 3.5 3.0 2.5 2.0 1.5 1.0 0.5 0.0 -0.5
f1 (ppm)

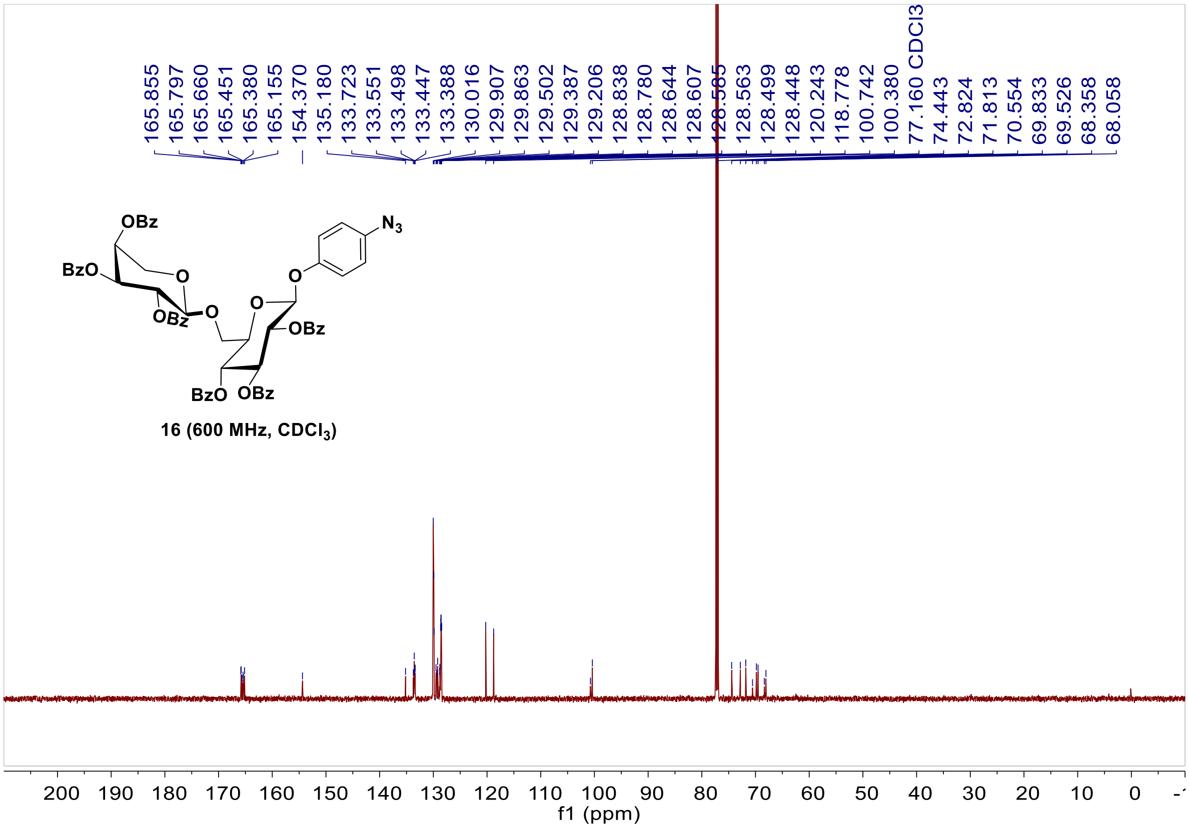
165.855 165.797 165.660 165.451 165.380 165.155 154.370 135.180 133.723 133.551 133.498 133.447 133.388 130.016 129.907 129.863 129.502 129.387 129.206 128.838 128.780 128.644 128.607 128.565 128.563 128.499 128.448 120.243 118.778 100.742 100.380 77.160 CDCl3 74.443 72.824 71.813 70.554 69.833 69.526 68.358 68.058
OBz
BzO
O
OBz
O
O
OBz
BzO OBz
O
N3
16 (600 MHz, CDCl3)
200 190 180 170 160 150 140 130 120 110 100 90 80 70 60 50 40 30 20 10 0
f1 (ppm)

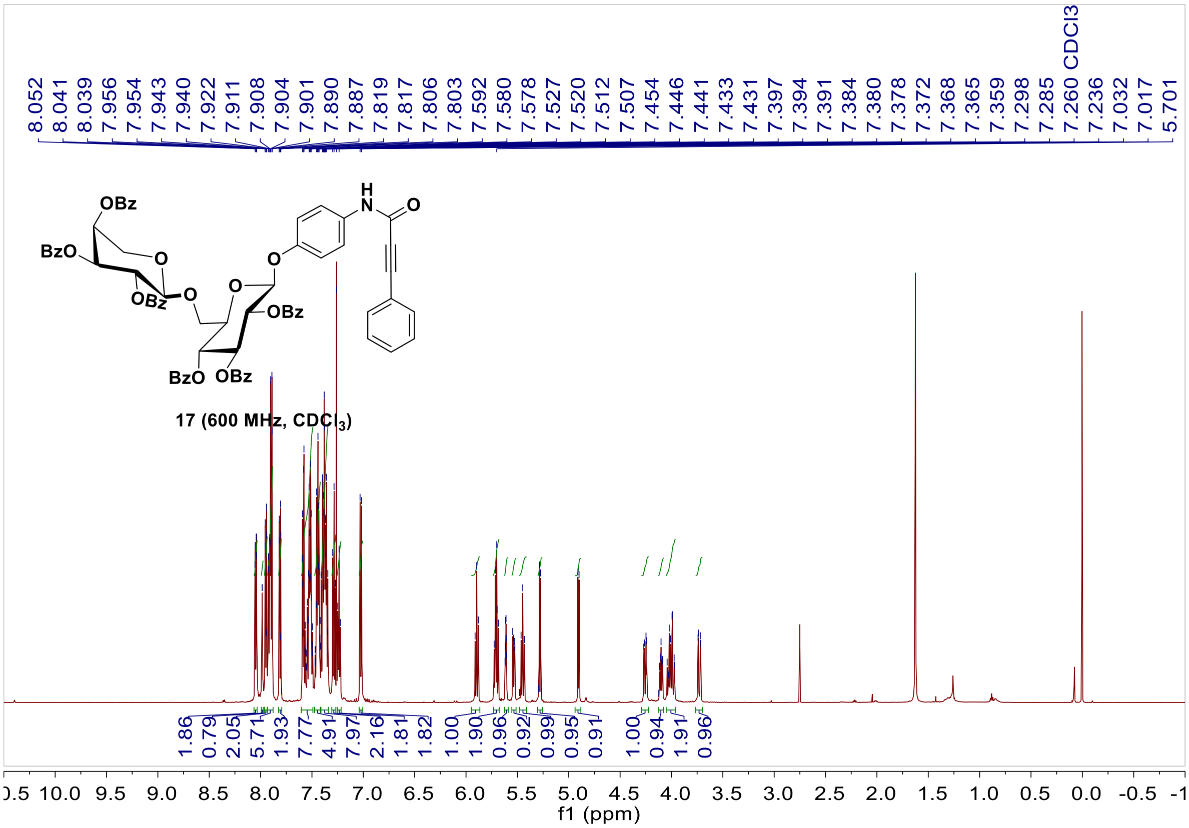
8.052 8.041 8.039 7.956 7.954 7.943 7.940 7.922 7.911 7.908 7.904 7.901 7.890 7.887 7.819 7.817 7.806 7.803 7.592 7.580 7.578 7.527 7.520 7.512 7.507 7.454 7.446 7.441 7.433 7.431 7.397 7.394 7.391 7.384 7.380 7.378 7.372 7.368 7.365 7.359 7.298 7.285 7.260 CDCl3 7.236 7.032 7.017 5.701
OBz
BzO
O
OBz
O
O
OBz
BzO OBz
O
H
N
O
17 (600 MHz, CDCl3)
1.86 0.79 2.05 5.71 1.93 7.77 4.91 7.97 2.16 1.81 1.82 1.00 1.90 0.96 0.92 0.99 0.95 0.91 1.00 0.94 1.91 0.96
f1 (ppm)

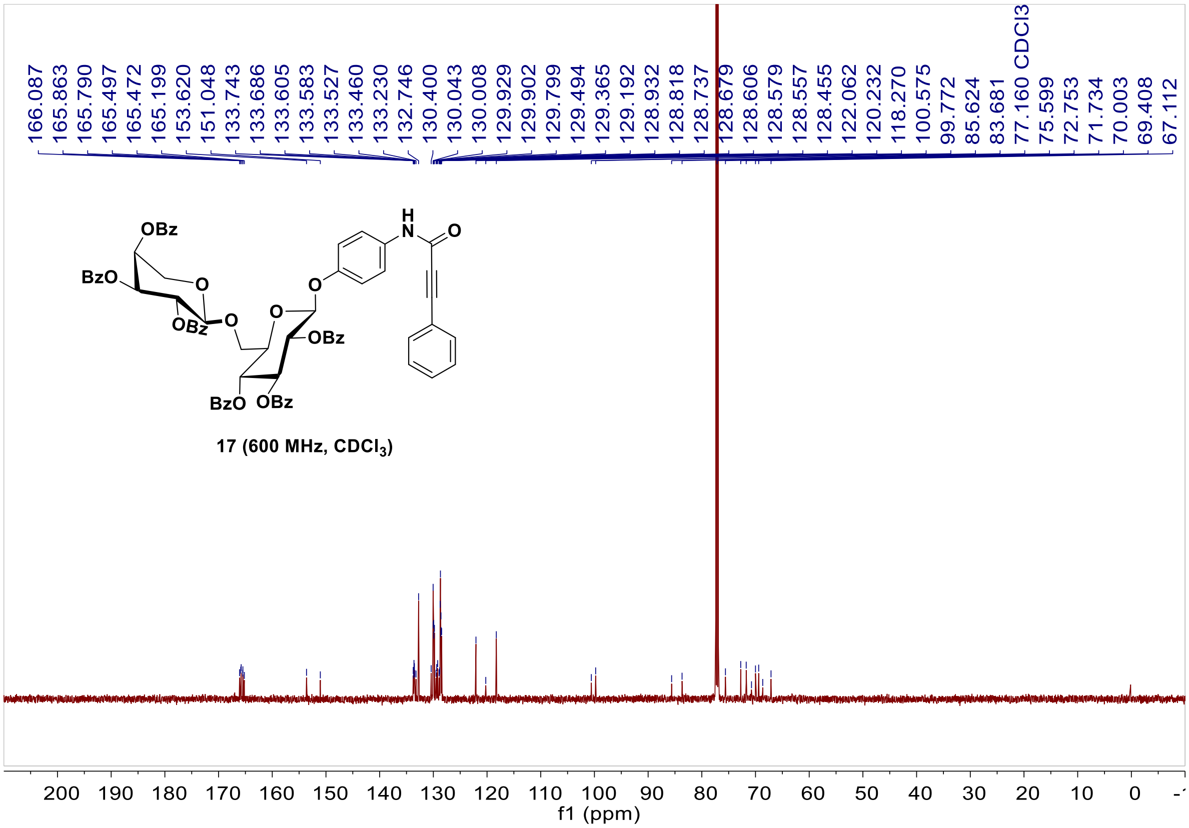
166.087 165.863 165.790 165.497 165.472 165.199 153.620 151.048 133.743 133.686 133.605 133.583 133.527 133.460 133.230 132.746 130.400 130.043 130.008 129.929 129.902 129.799 129.494 129.365 129.192 128.932 128.818 128.737 128.679 128.606 128.579 128.557 128.455 122.062 120.232 118.270 100.575 99.772 85.624 83.681 77.160 CDCl3 75.599 72.753 71.734 70.003 69.408 67.112
OBz
BzO
O
OBz
O
O
OBz
BzO OBz
O
H
N
O
17 (600 MHz, CDCl3)
f1 (ppm)

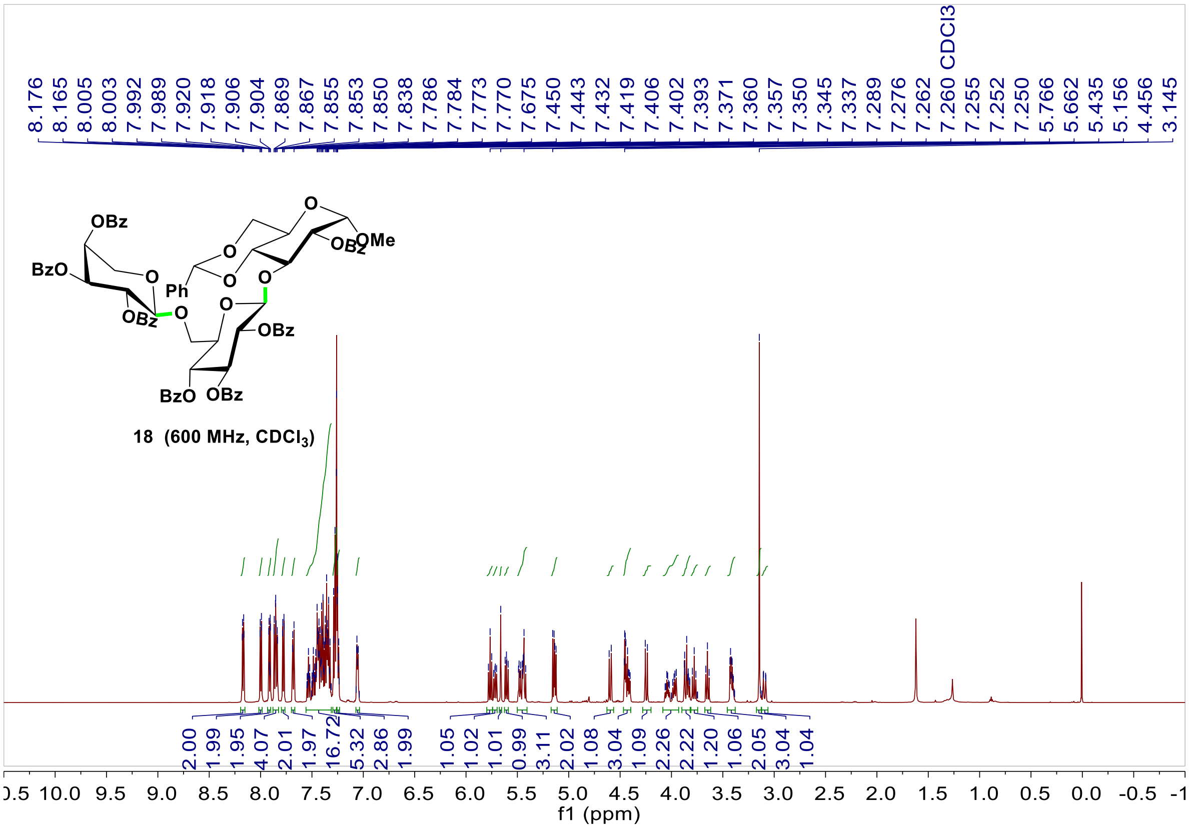
8.176 8.165 8.005 8.003 7.992 7.989 7.920 7.918 7.906 7.904 7.869 7.867 7.855 7.853 7.850 7.838 7.786 7.784 7.773 7.770 7.675 7.450 7.443 7.432 7.419 7.406 7.402 7.393 7.371 7.360 7.357 7.350 7.345 7.337 7.289 7.276 7.262 7.260 CDCl3 7.255 7.252 7.250 5.766 5.662 5.435 5.156 4.456 3.145
OBz
BzO
O
Ph
O
O
O
O
OBz
OMe
OBz
O
O
OBz
BzO OBz
18 (600 MHz, CDCl3)
2.00 1.99 1.95 4.07 2.01 1.97 16.72 5.32 2.86 1.99 1.05 1.02 1.01 0.99 3.11 2.02 1.08 3.04 1.09 2.26 2.22 1.20 1.06 2.05 3.04 1.04
0.5 10.0 9.5 9.0 8.5 8.0 7.5 7.0 6.5 6.0 5.5 5.0 4.5 4.0 3.5 3.0 2.5 2.0 1.5 1.0 0.5 0.0 -0.5 -1
f1 (ppm)

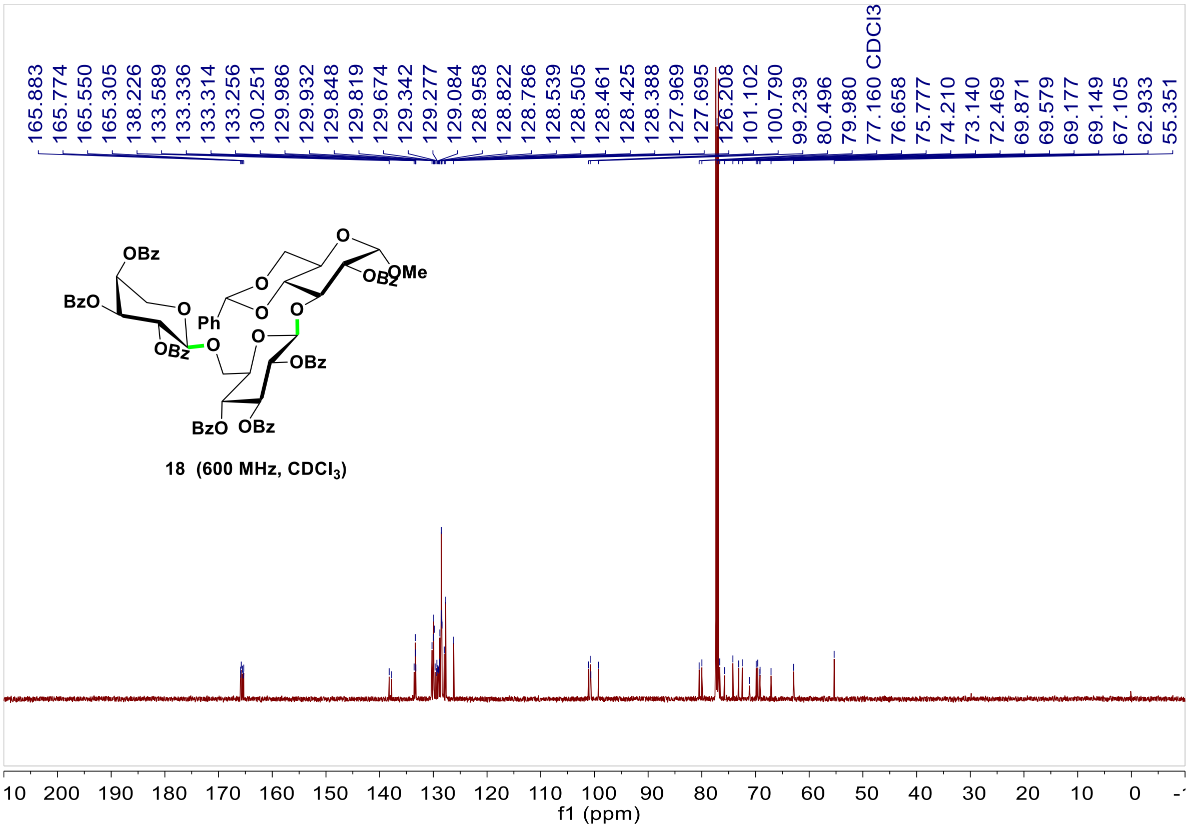
165.883 165.774 165.550 165.305 138.226 133.589 133.336 133.314 133.256 130.251 129.986 129.932 129.848 129.819 129.674 129.342 129.277 129.084 128.958 128.822 128.786 128.539 128.505 128.461 128.425 128.388 127.969 127.695 126.208 101.102 100.790 99.239 80.496 79.980 77.160 CDCl3 76.658 75.777 74.210 73.140 72.469 69.871 69.579 69.177 69.149 67.105 62.933 55.351
OBz
BzO
O
Ph
O
O
O
O
OBz
OMe
OBz
O
O
OBz
BzO OBz
18 (600 MHz, CDCl3)
10 200 190 180 170 160 150 140 130 120 110 100 90 80 70 60 50 40 30 20 10 0
f1 (ppm)

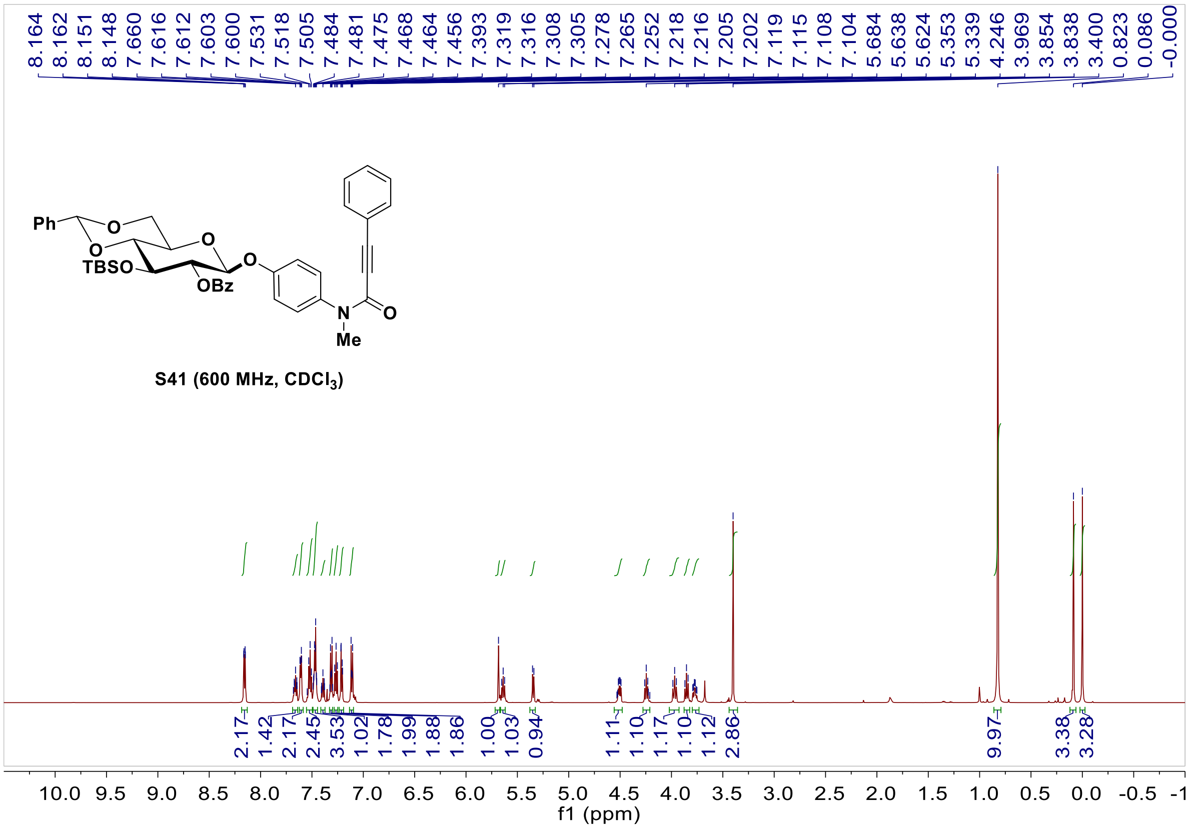

8.164 8.162 8.151 8.148 7.660 7.616 7.612 7.603 7.600 7.531 7.518 7.505 7.484 7.481 7.475 7.468 7.464 7.456 7.393 7.319 7.316 7.308 7.305 7.278 7.265 7.252 7.218 7.216 7.205 7.202 7.119 7.115 7.108 7.104 5.684 5.638 5.624 5.353 5.339 4.246 3.969 3.854 3.838 3.400 0.823 0.086 -0.000
Ph
O
O
O
TBSO
OBz
O
N
O
Me
S41 (600 MHz, CDCl$_3$)
2.17 1.42 2.17 2.45 3.53 1.02 1.78 1.99 1.88 1.86 1.00 1.03 0.94 1.11 1.10 1.17 1.10 1.12 2.86 9.97 3.38 3.28
10.0 9.5 9.0 8.5 8.0 7.5 7.0 6.5 6.0 5.5 5.0 4.5 4.0 3.5 3.0 2.5 2.0 1.5 1.0 0.5 0.0 -0.5 -1
f1 (ppm)

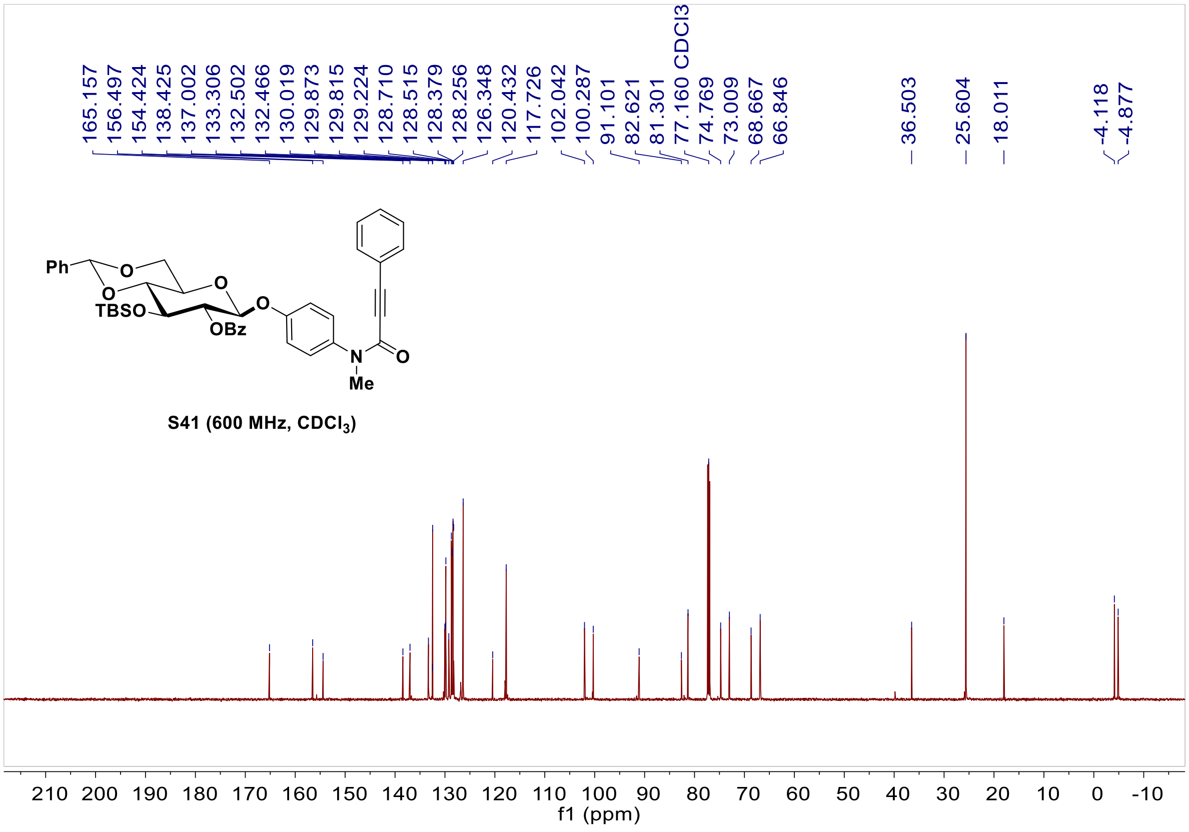

165.157 156.497 154.424 138.425 137.002 133.306 132.502 132.466 130.019 129.873 129.815 129.224 128.710 128.515 128.379 128.256 126.348 120.432 117.726 102.042 100.287 91.101 82.621 81.301 77.160 CDCl3 74.769 73.009 68.667 66.846 36.503 25.604 18.011 -4.118 -4.877
Ph
O
O
O
TBSO
OBz
O
N
O
Me
S41 (600 MHz, CDCl$_3$)
210 200 190 180 170 160 150 140 130 120 110 100 90 80 70 60 50 40 30 20 10 0 -10
f1 (ppm)

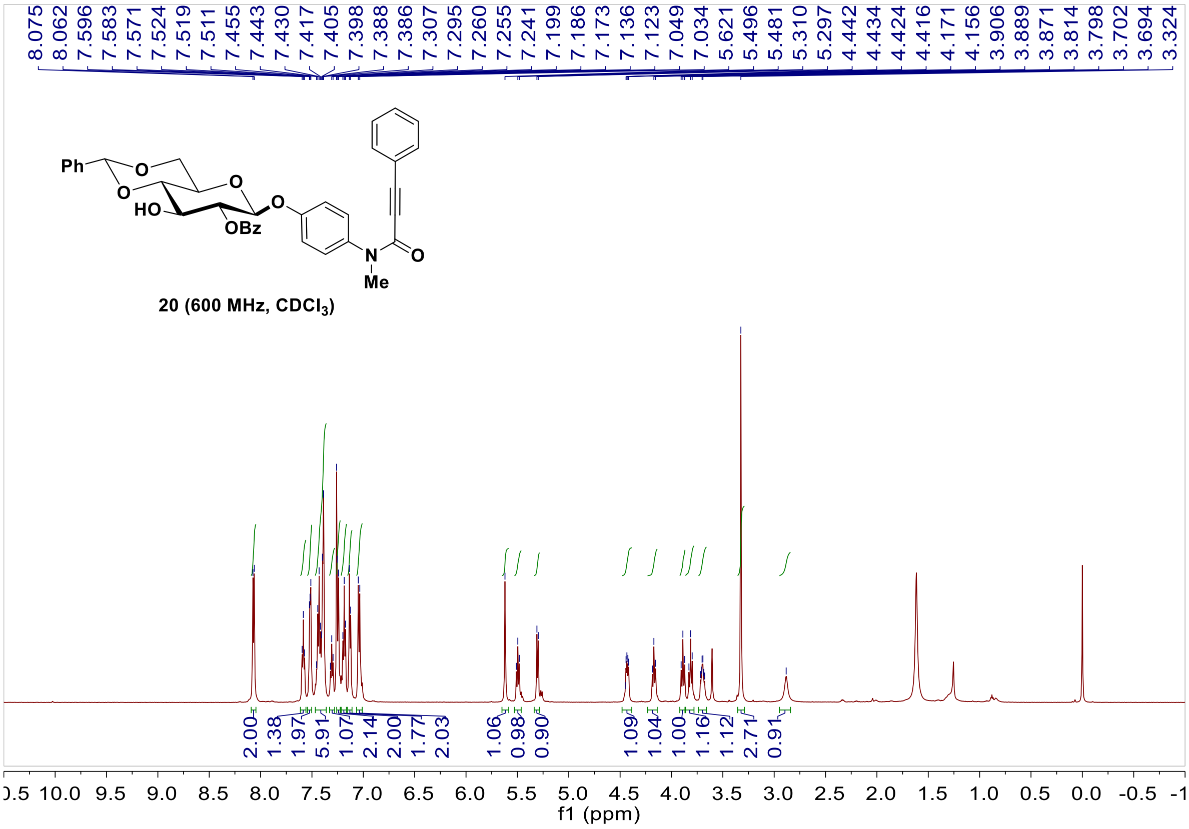
8.075 8.062 7.596 7.583 7.571 7.524 7.519 7.511 7.455 7.443 7.430 7.417 7.405 7.398 7.388 7.386 7.307 7.295 7.260 7.255 7.241 7.199 7.186 7.173 7.136 7.123 7.049 7.034 5.621 5.496 5.481 5.310 5.297 4.442 4.434 4.424 4.416 4.171 4.156 3.906 3.889 3.871 3.814 3.798 3.702 3.694 3.324
Ph
O
O
HO
OBz
O
O
N
O
Me
20 (600 MHz, CDCl3)
2.00 1.38 1.97 5.91 1.07 2.14 2.00 1.77 2.03 1.06 0.98 0.90 1.09 1.04 1.00 1.16 1.12 2.71 0.91
10.5 10.0 9.5 9.0 8.5 8.0 7.5 7.0 6.5 6.0 5.5 5.0 4.5 4.0 3.5 3.0 2.5 2.0 1.5 1.0 0.5 0.0 -0.5 -1
f1 (ppm)

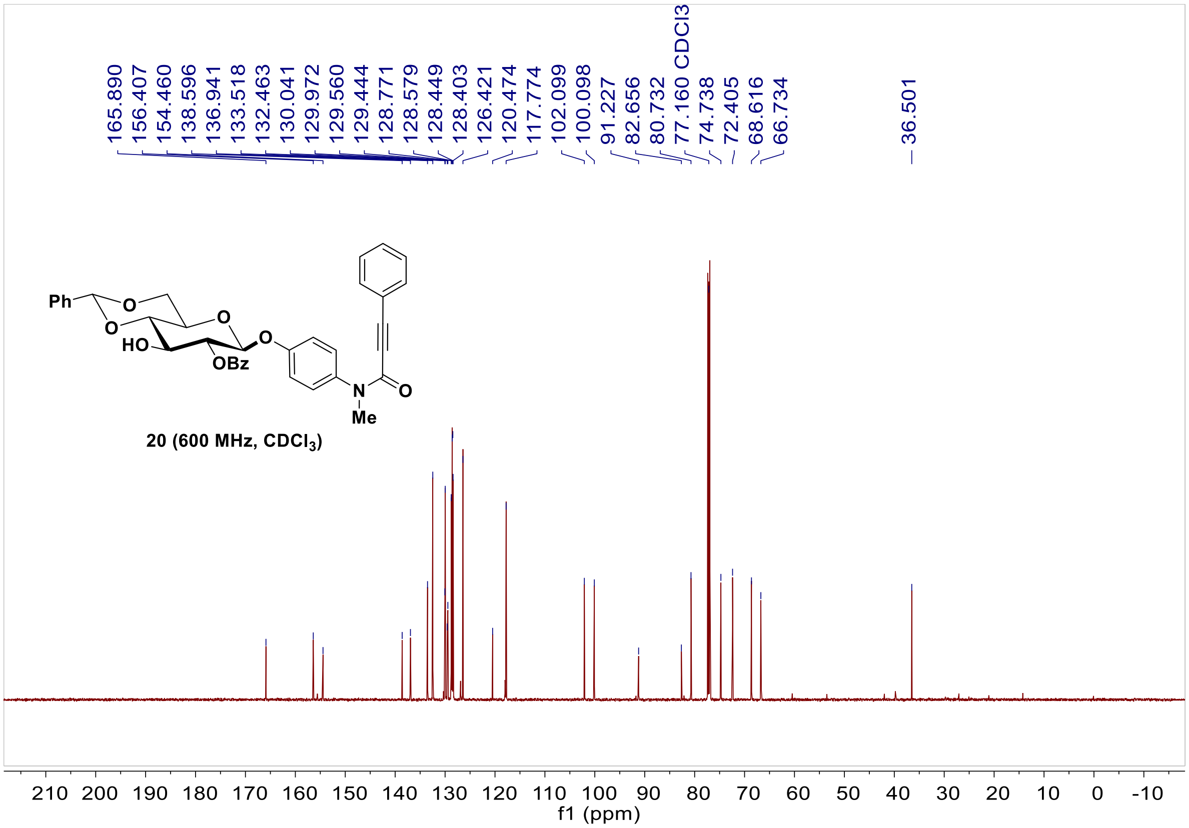
165.890 156.407 154.460 138.596 136.941 133.518 132.463 130.041 129.972 129.560 129.444 128.771 128.579 128.449 128.403 126.421 120.474 117.774 102.099 100.098 91.227 82.656 80.732 77.160 CDCl3 74.738 72.405 68.616 66.734
36.501
Ph
O
O
HO
OBz
O
O
N
O
Me
20 (600 MHz, CDCl3)
210 200 190 180 170 160 150 140 130 120 110 100 90 80 70 60 50 40 30 20 10 0 -10
f1 (ppm)

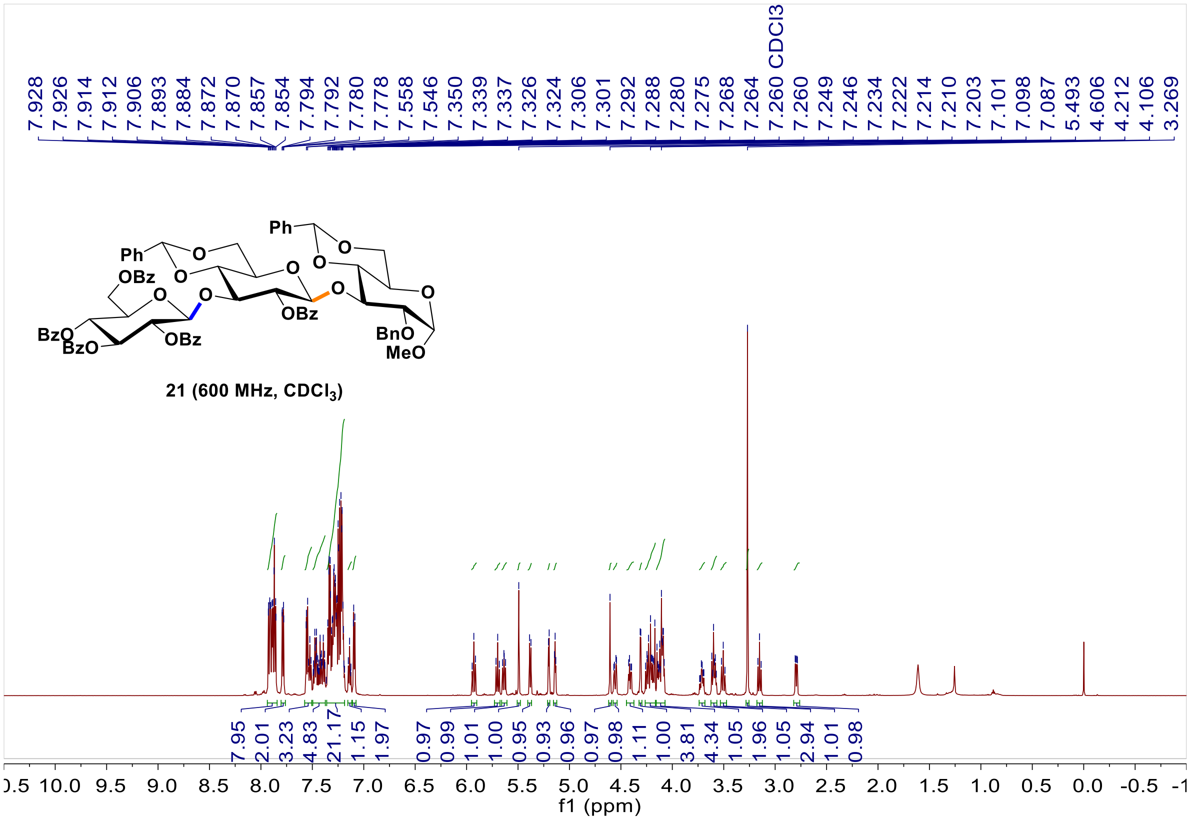

7.928
7.926
7.914
7.912
7.906
7.893
7.884
7.872
7.870
7.857
7.854
7.794
7.792
7.780
7.778
7.558
7.546
7.350
7.339
7.337
7.326
7.324
7.306
7.301
7.292
7.288
7.280
7.275
7.268
7.264
7.260 CDCl3
7.260
7.249
7.246
7.234
7.222
7.214
7.210
7.203
7.101
7.098
7.087
5.493
4.606
4.212
4.106
3.269
Ph
O
O
Ph
OBz
O
O
O
O
O
O
BzO
BzO
OBz
OBz
BnO
MeO
21 (600 MHz, CDCl3)
7.95
2.01
3.23
4.83
21.17
1.15
1.97
0.97
0.99
1.01
1.00
0.95
0.93
0.96
0.97
0.98
1.11
1.00
3.81
4.34
1.05
1.96
1.05
2.94
1.01
0.98
10.5 10.0 9.5 9.0 8.5 8.0 7.5 7.0 6.5 6.0 5.5 5.0 4.5 4.0 3.5 3.0 2.5 2.0 1.5 1.0 0.5 0.0 -0.5 -1
f1 (ppm)

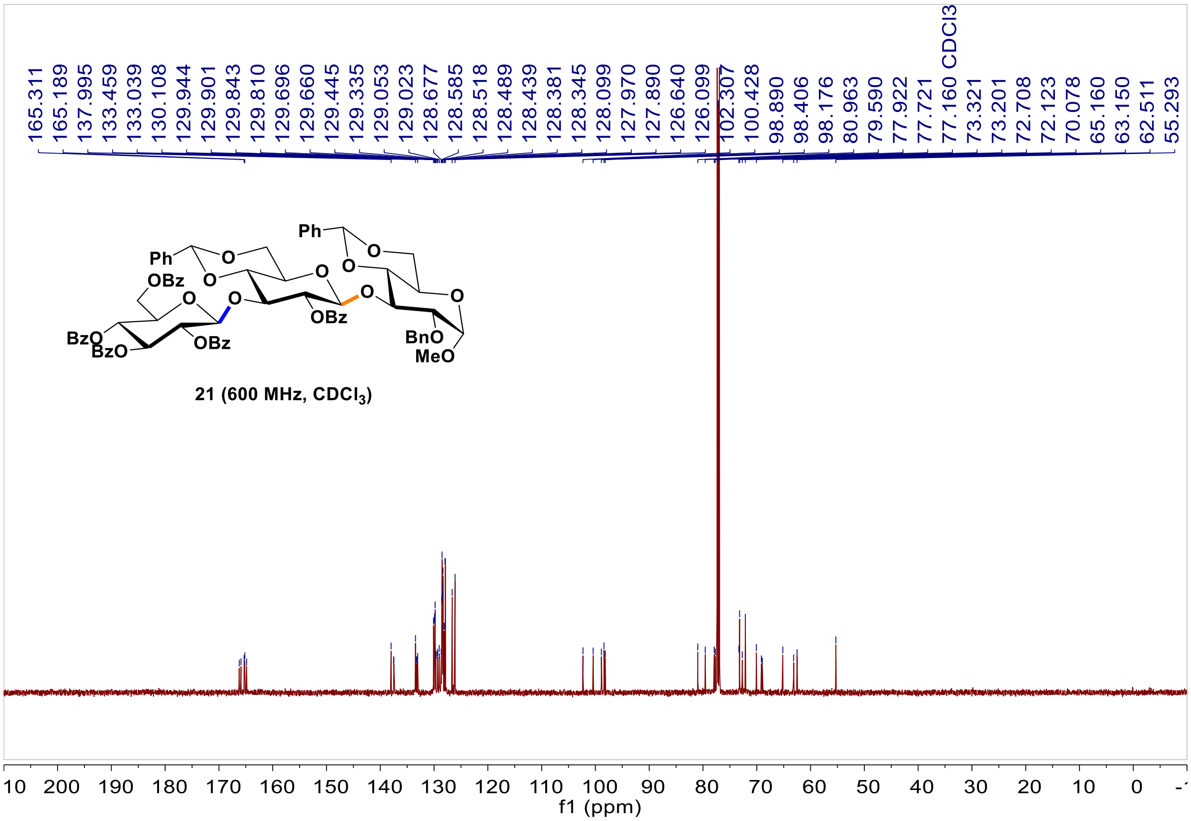

165.311
165.189
137.995
133.459
133.039
130.108
129.944
129.901
129.843
129.810
129.696
129.660
129.445
129.335
129.053
129.023
128.677
128.585
128.518
128.489
128.439
128.381
128.345
128.099
127.970
127.890
126.640
126.099
102.307
100.428
98.890
98.406
98.176
80.963
79.590
77.922
77.721
77.160 CDCl3
73.321
73.201
72.708
72.123
70.078
65.160
63.150
62.511
55.293
Ph
O
O
Ph
OBz
O
O
O
O
O
O
BzO
BzO
OBz
OBz
BnO
MeO
21 (600 MHz, CDCl3)
210 200 190 180 170 160 150 140 130 120 110 100 90 80 70 60 50 40 30 20 10 0 -10
f1 (ppm)

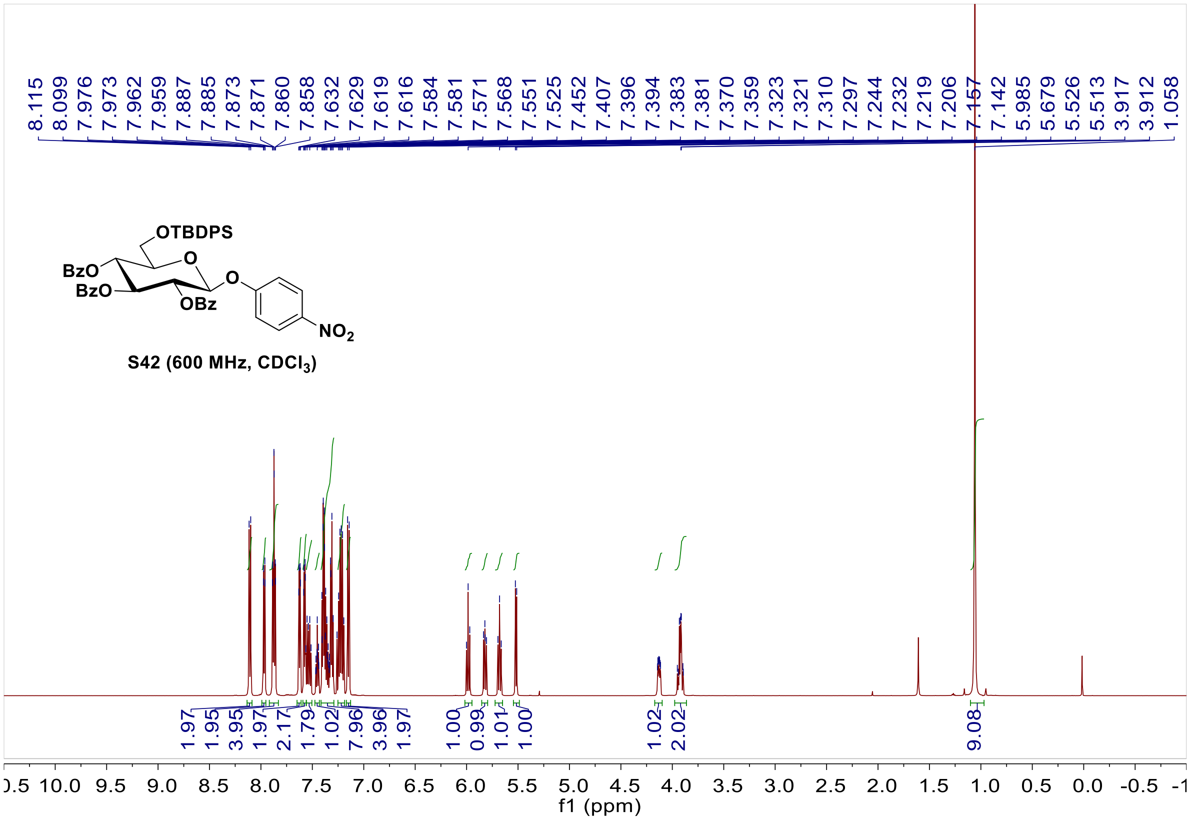
8.115 8.099 7.976 7.973 7.962 7.959 7.887 7.885 7.873 7.871 7.860 7.858 7.632 7.629 7.619 7.616 7.584 7.581 7.571 7.568 7.551 7.525 7.452 7.407 7.396 7.394 7.383 7.381 7.370 7.359 7.323 7.321 7.310 7.297 7.244 7.232 7.219 7.206 7.157 7.142 5.985 5.679 5.526 5.513 3.917 3.912 1.058
OTBDPS
BzO
BzO
O
O
OBz
NO2
S42 (600 MHz, CDCl3)
1.97 1.95 3.95 1.97 2.17 1.79 1.02 7.96 3.96 1.97 1.00 0.99 1.01 1.00 1.02 2.02 9.08
f1 (ppm)

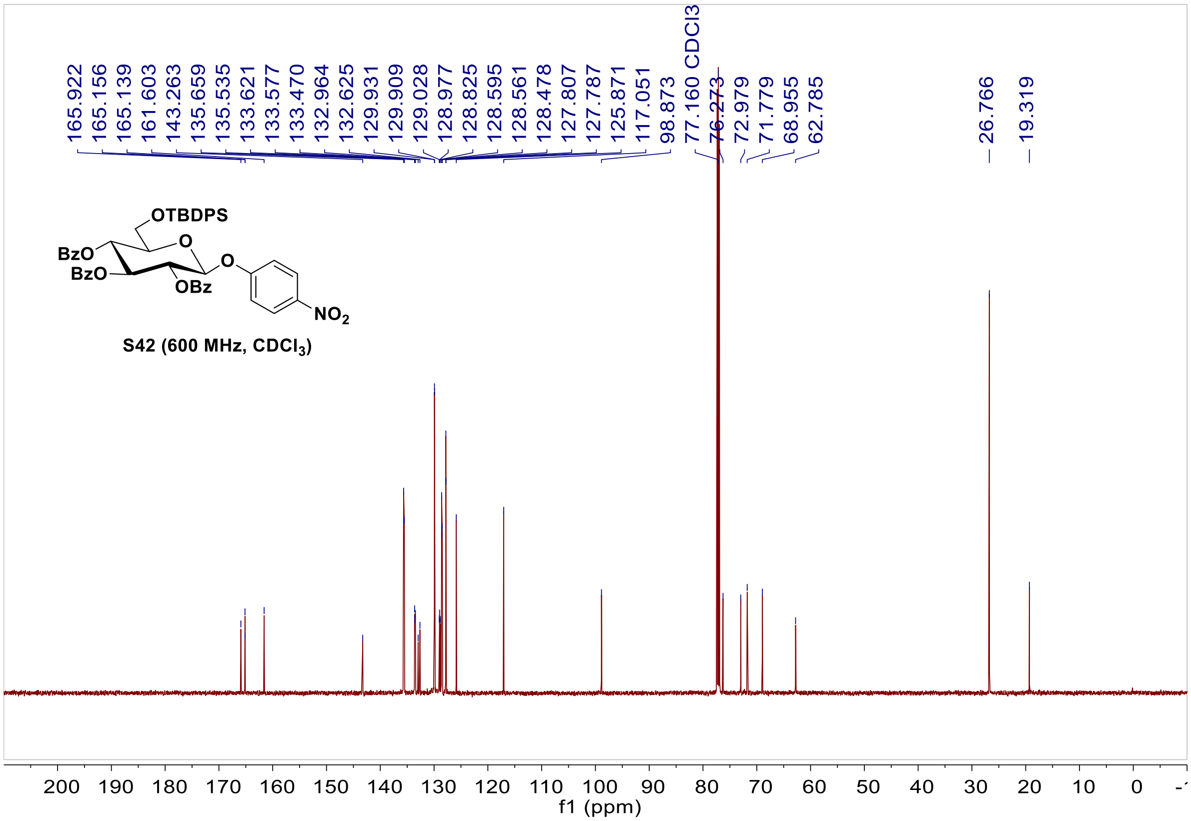
165.922 165.156 165.139 161.603 143.263 135.659 135.535 133.621 133.577 133.470 132.964 132.625 129.931 129.909 129.028 128.977 128.825 128.595 128.561 128.478 127.807 127.787 125.871 117.051 98.873 77.160 CDCl3 76.273 72.979 71.779 68.955 62.785 26.766 19.319
OTBDPS
BzO
BzO
O
O
OBz
NO2
S42 (600 MHz, CDCl3)
f1 (ppm)

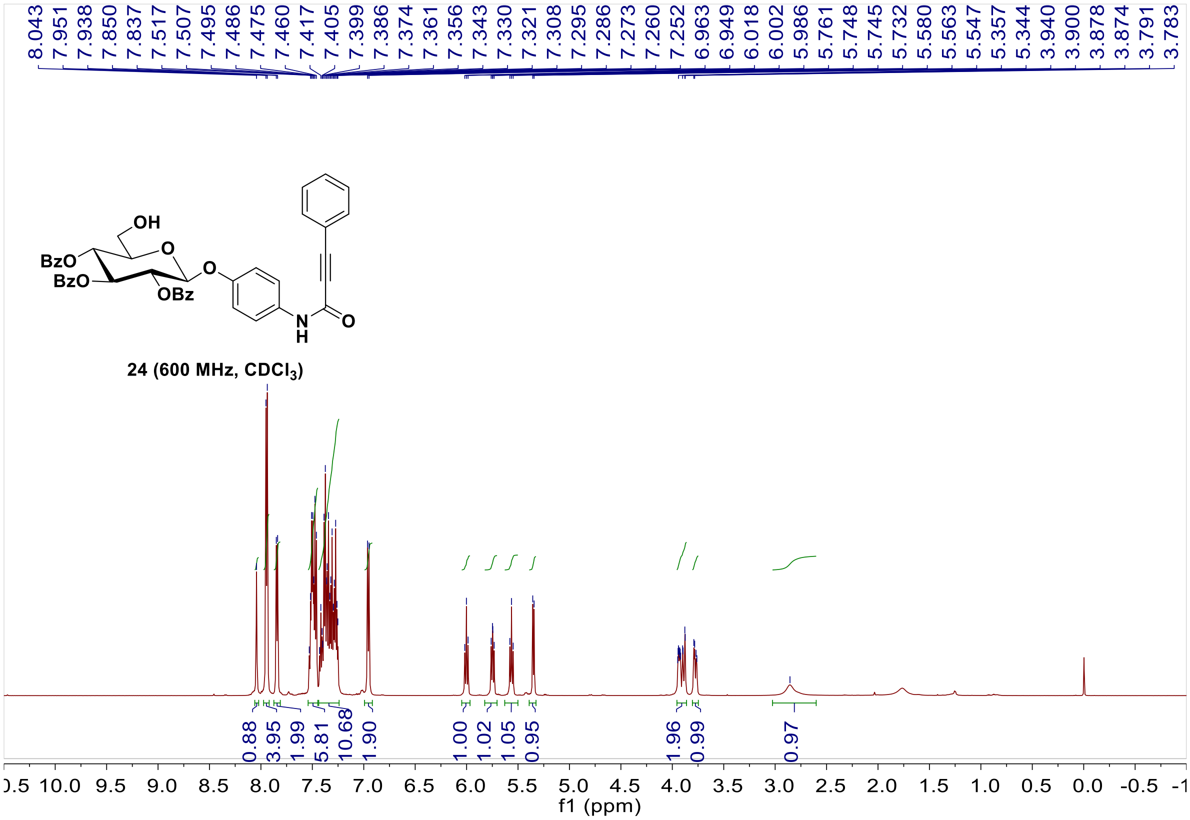
8.043 7.951 7.938 7.850 7.837 7.517 7.507 7.495 7.486 7.475 7.460 7.417 7.405 7.399 7.386 7.374 7.361 7.356 7.343 7.330 7.321 7.308 7.295 7.286 7.273 7.260 7.252 6.963 6.949 6.018 6.002 5.986 5.761 5.748 5.745 5.732 5.580 5.563 5.547 5.357 5.344 3.940 3.900 3.878 3.874 3.791 3.783
OH
O
BzO
BzO
OBz
O
N
H
O
24 (600 MHz, CDCl3)
0.88 3.95 1.99 5.81 10.68 1.90 1.00 1.02 1.05 0.95 1.96 0.99 0.97
f1 (ppm)

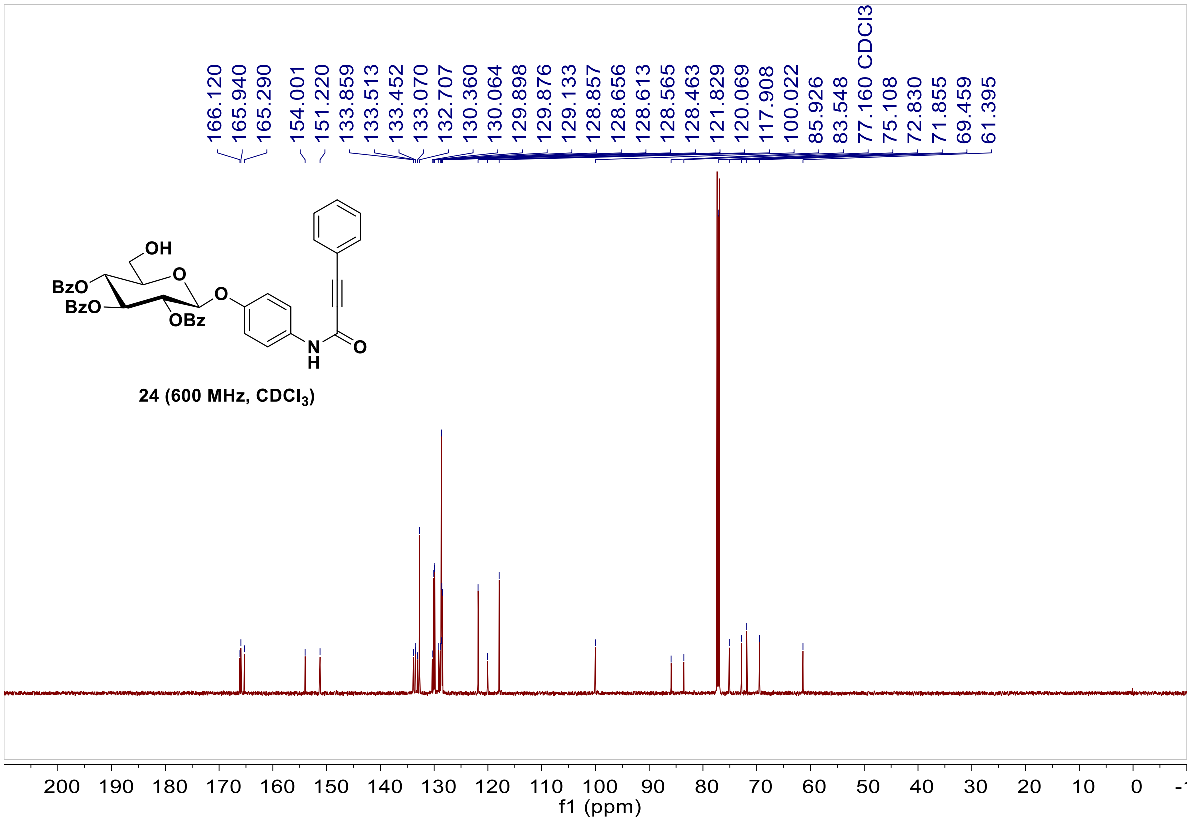
166.120 165.940 165.290 154.001 151.220 133.859 133.513 133.452 133.070 132.707 130.360 130.064 129.898 129.876 129.133 128.857 128.656 128.613 128.565 128.463 121.829 120.069 117.908 100.022 85.926 83.548 77.160 CDCl3 75.108 72.830 71.855 69.459 61.395
OH
O
BzO
BzO
OBz
O
N
H
O
24 (600 MHz, CDCl3)
f1 (ppm)

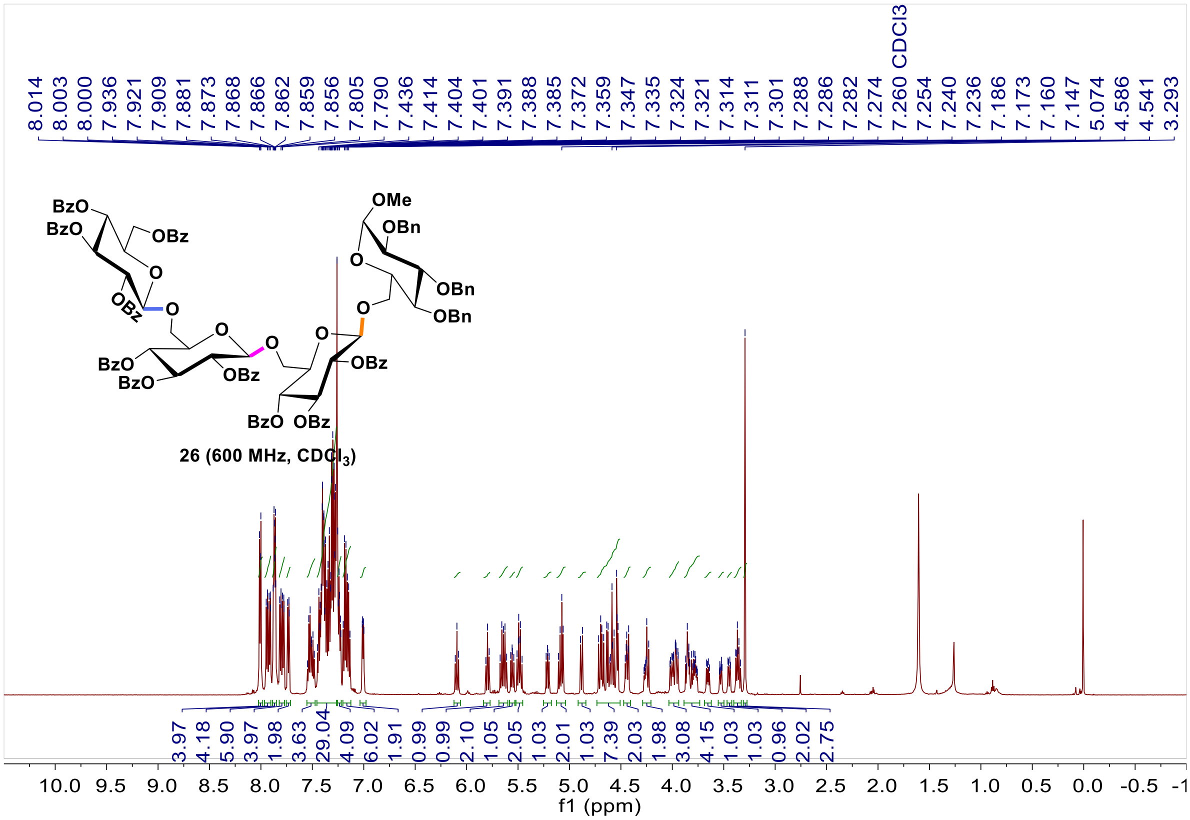
8.014
8.003
8.000
7.936
7.921
7.909
7.881
7.873
7.868
7.866
7.862
7.859
7.856
7.805
7.790
7.436
7.414
7.404
7.401
7.391
7.388
7.385
7.372
7.359
7.347
7.335
7.324
7.321
7.314
7.311
7.301
7.288
7.286
7.282
7.274
7.260 CDCl3
7.254
7.240
7.236
7.186
7.173
7.160
7.147
5.074
4.586
4.541
3.293
BzO
BzO
OBz
O
OBz
O
O
BzO
BzO
OBz
O
O
OBz
BzO
OBz
OMe
OBn
O
OBn
OBn
O
26 (600 MHz, CDCl3)
3.97
4.18
5.90
3.97
1.98
3.63
29.04
4.09
6.02
1.91
0.99
0.99
2.10
1.05
2.05
1.03
2.01
1.03
7.39
2.03
1.98
3.08
4.15
1.03
1.03
0.96
2.02
2.75
10.0 9.5 9.0 8.5 8.0 7.5 7.0 6.5 6.0 5.5 5.0 4.5 4.0 3.5 3.0 2.5 2.0 1.5 1.0 0.5 0.0 -0.5 -1
f1 (ppm)

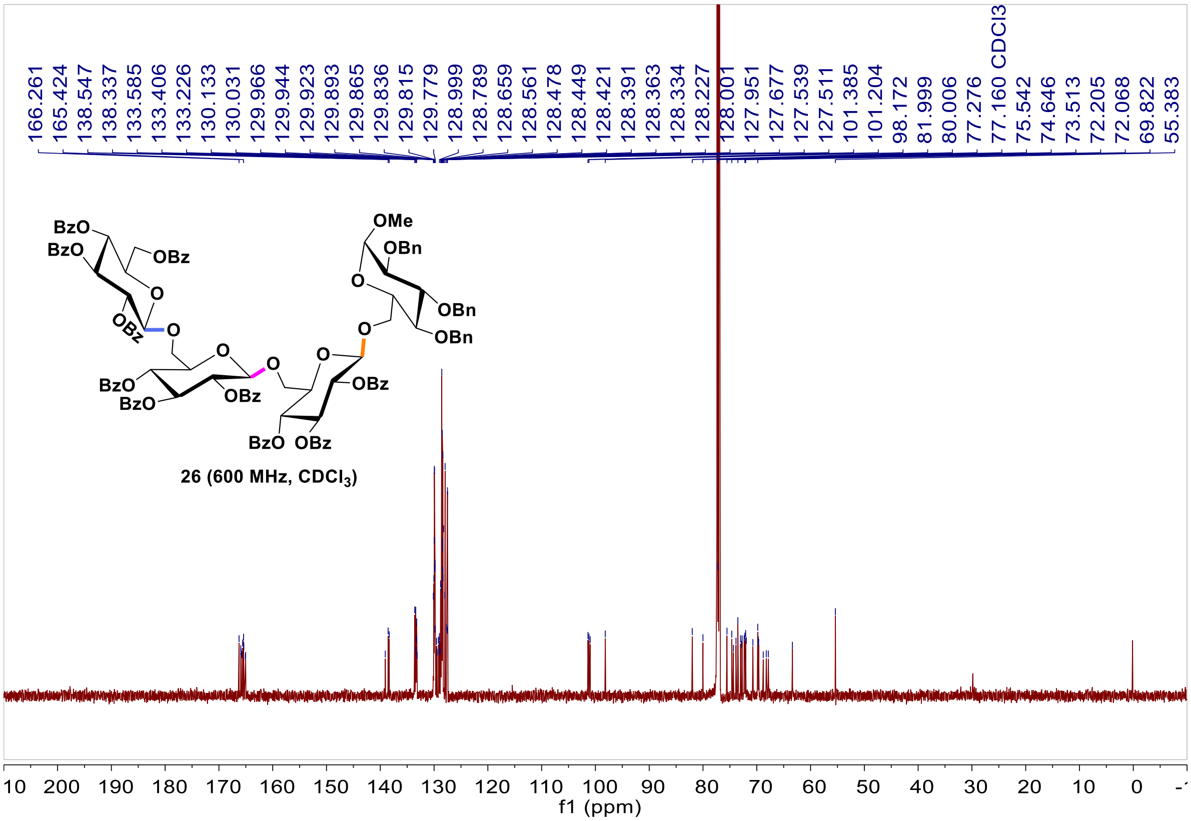
166.261
165.424
138.547
138.337
133.585
133.406
133.226
130.133
130.031
129.966
129.944
129.923
129.893
129.865
129.836
129.815
129.779
128.999
128.789
128.659
128.561
128.478
128.449
128.421
128.391
128.363
128.334
128.227
128.001
127.951
127.677
127.539
127.511
101.385
101.204
98.172
81.999
80.006
77.276
77.160 CDCl3
75.542
74.646
73.513
72.205
72.068
69.822
55.383
BzO
BzO
OBz
O
OBz
O
O
BzO
BzO
OBz
O
O
OBz
BzO
OBz
OMe
OBn
O
OBn
OBn
O
26 (600 MHz, CDCl3)
10 200 190 180 170 160 150 140 130 120 110 100 90 80 70 60 50 40 30 20 10 0
f1 (ppm)

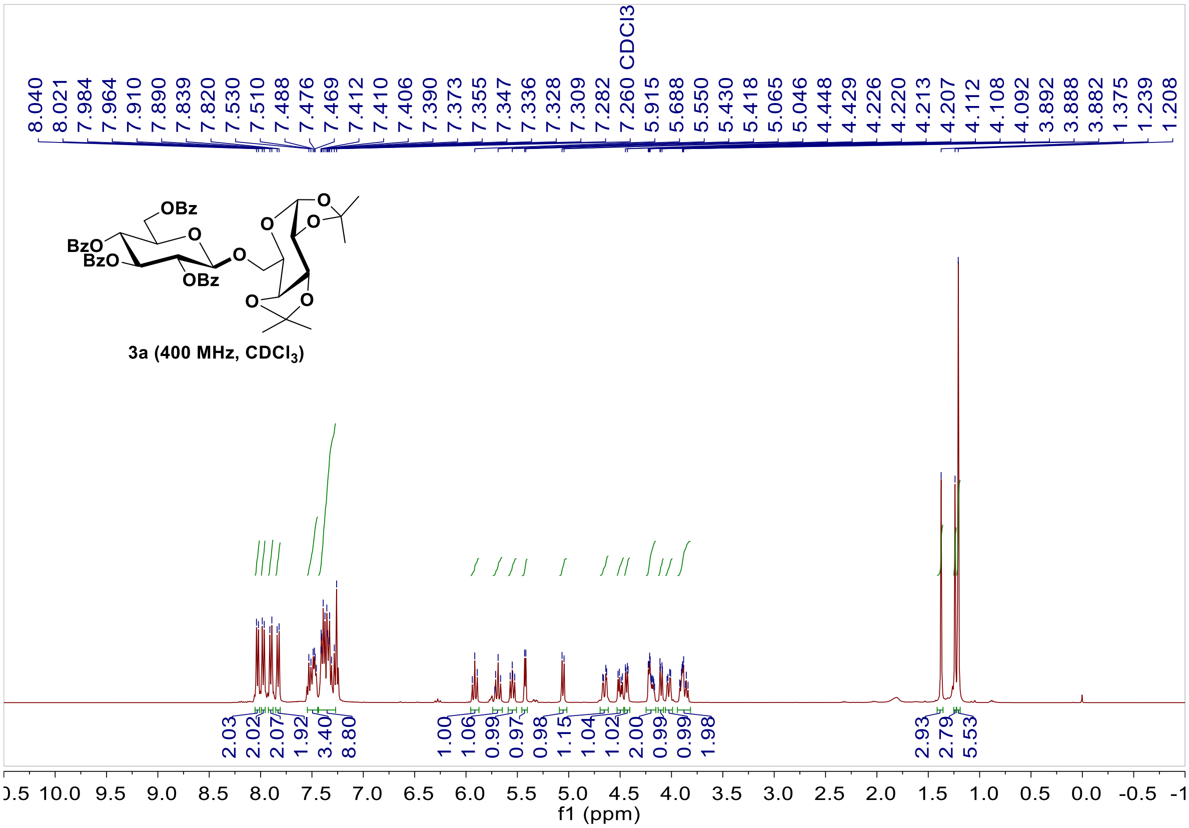
3a (400 MHz, CDCl3)
CDCl3
8.040 8.021 7.984 7.964 7.910 7.890 7.839 7.820 7.530 7.510 7.488 7.476 7.469 7.412 7.410 7.406 7.390 7.373 7.355 7.347 7.336 7.328 7.309 7.282 7.260 5.915 5.688 5.550 5.430 5.418 5.065 5.046 4.448 4.429 4.226 4.220 4.213 4.207 4.112 4.108 4.092 3.892 3.888 3.882 1.375 1.239 1.208
2.03 2.02 2.07 1.92 3.40 8.80 1.00 1.06 0.99 0.97 0.98 1.15 1.04 1.02 2.00 0.99 0.99 1.98 2.93 2.79 5.53
f1 (ppm)

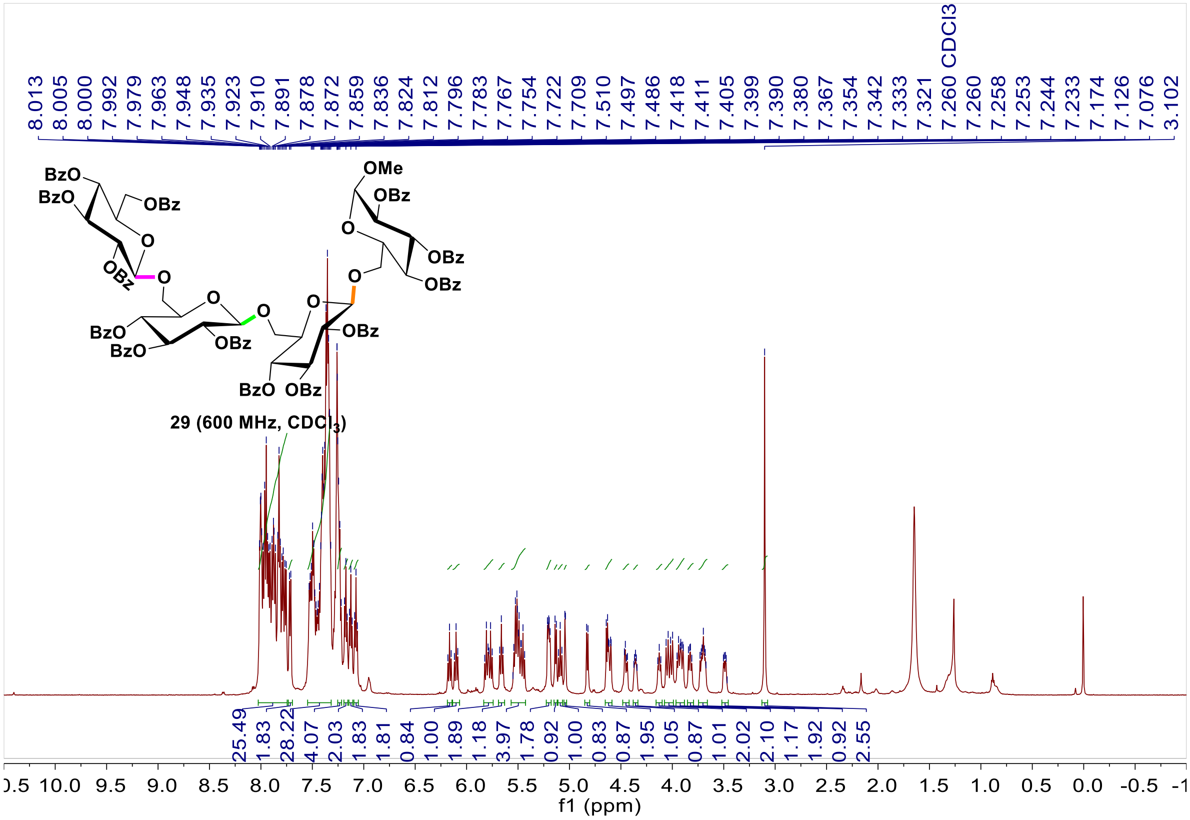
29 (600 MHz, CDCl3)
CDCl3
8.013 8.005 8.000 7.992 7.979 7.963 7.948 7.935 7.923 7.910 7.891 7.878 7.872 7.859 7.836 7.824 7.812 7.796 7.783 7.767 7.754 7.722 7.709 7.510 7.497 7.486 7.418 7.411 7.405 7.399 7.390 7.380 7.367 7.354 7.342 7.333 7.321 7.260 7.260 7.258 7.253 7.244 7.233 7.174 7.126 7.076 3.102
25.49 1.83 28.22 4.07 2.03 1.83 1.81 0.84 1.00 1.89 1.18 3.97 1.78 0.92 1.00 0.83 0.87 1.95 1.05 0.87 1.01 2.02 2.10 1.17 1.92 0.92 2.55
f1 (ppm)

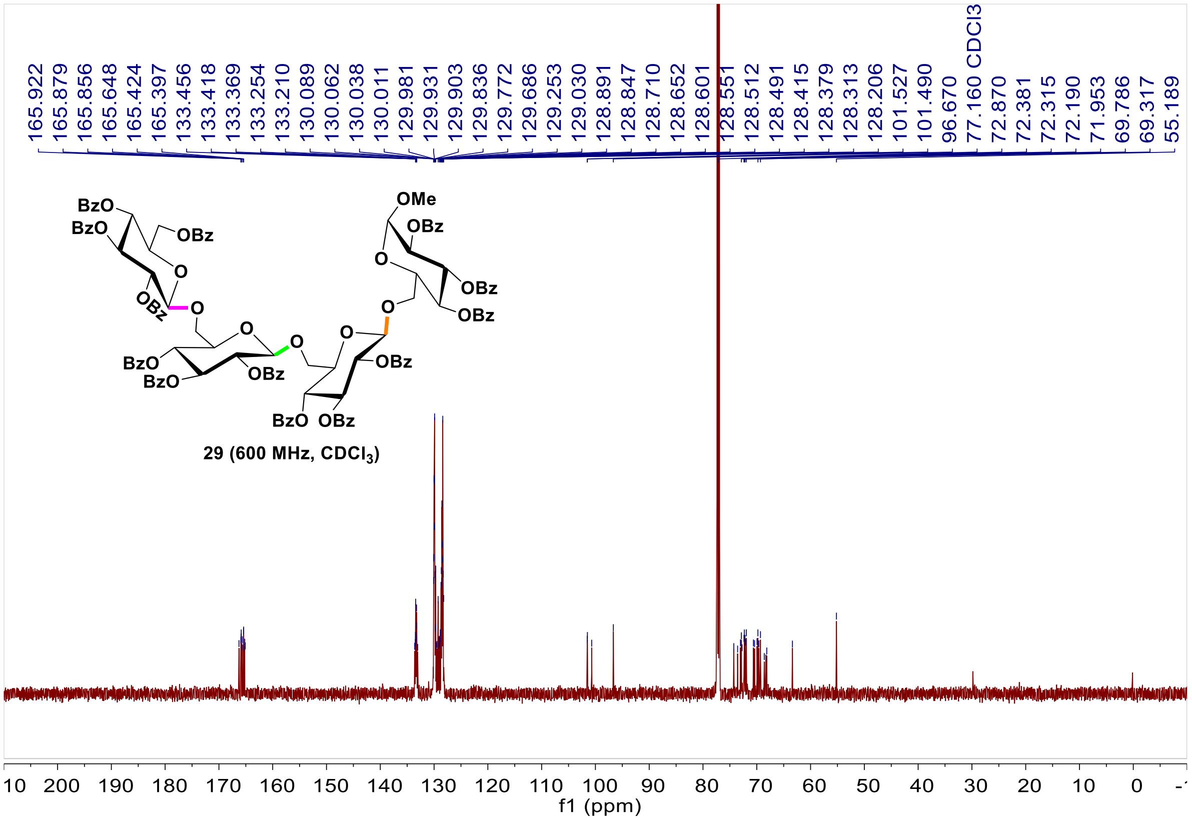
165.922 165.879 165.856 165.648 165.424 165.397 133.456 133.418 133.369 133.254 133.210 130.089 130.062 130.038 130.011 129.981 129.931 129.903 129.836 129.772 129.686 129.253 129.030 128.891 128.847 128.710 128.652 128.601 128.551 128.512 128.491 128.415 128.379 128.313 128.206 101.527 101.490 96.670 77.160 CDCl3 72.870 72.381 72.315 72.190 71.953 69.786 69.317 55.189
BzO
BzO
OBz
O
OBz
O
O
BzO
BzO
OBz
O
O
OMe
OBz
O
OBz
OBz
O
OBz
BzO OBz
29 (600 MHz, CDCl3)
10 200 190 180 170 160 150 140 130 120 110 100 90 80 70 60 50 40 30 20 10 0
f1 (ppm)

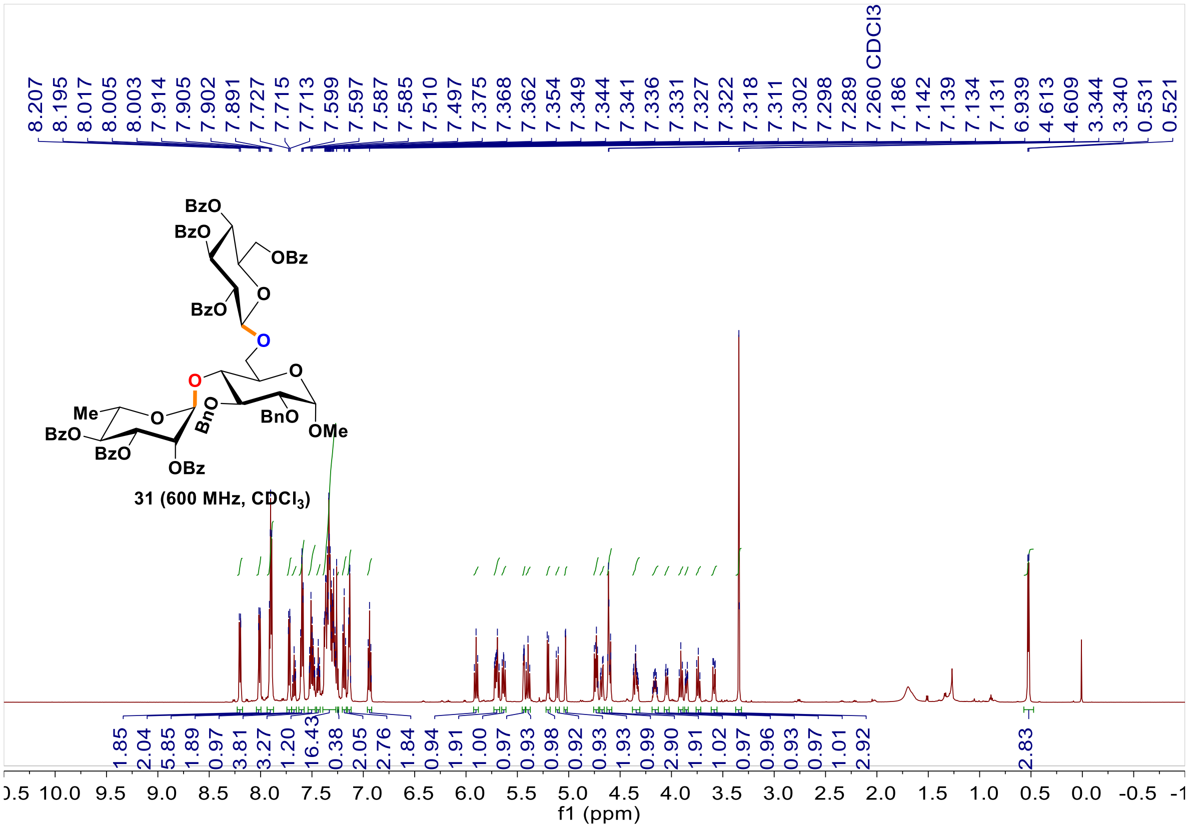
8.207 8.195 8.017 8.005 8.003 7.914 7.905 7.902 7.891 7.727 7.715 7.713 7.599 7.597 7.587 7.585 7.510 7.497 7.375 7.368 7.362 7.354 7.349 7.344 7.341 7.336 7.331 7.327 7.322 7.318 7.311 7.302 7.298 7.289 7.260 CDCl3 7.186 7.142 7.139 7.134 7.131 6.939 4.613 4.609 3.344 3.340 0.531 0.521
BzO
BzO
OBz
BzO
O
O
O
O
Me
BzO
BzO
OBz
O
BnO
BnO
OMe
31 (600 MHz, CDCl3)
1.85 2.04 5.85 1.89 0.97 3.81 3.27 1.20 16.43 0.38 2.05 2.76 1.84 0.94 1.91 1.00 0.97 0.93 0.98 0.92 0.93 1.93 0.99 2.90 1.91 1.02 0.97 0.96 0.93 0.97 1.01 2.92 2.83
0.5 10.0 9.5 9.0 8.5 8.0 7.5 7.0 6.5 6.0 5.5 5.0 4.5 4.0 3.5 3.0 2.5 2.0 1.5 1.0 0.5 0.0 -0.5 -1
f1 (ppm)

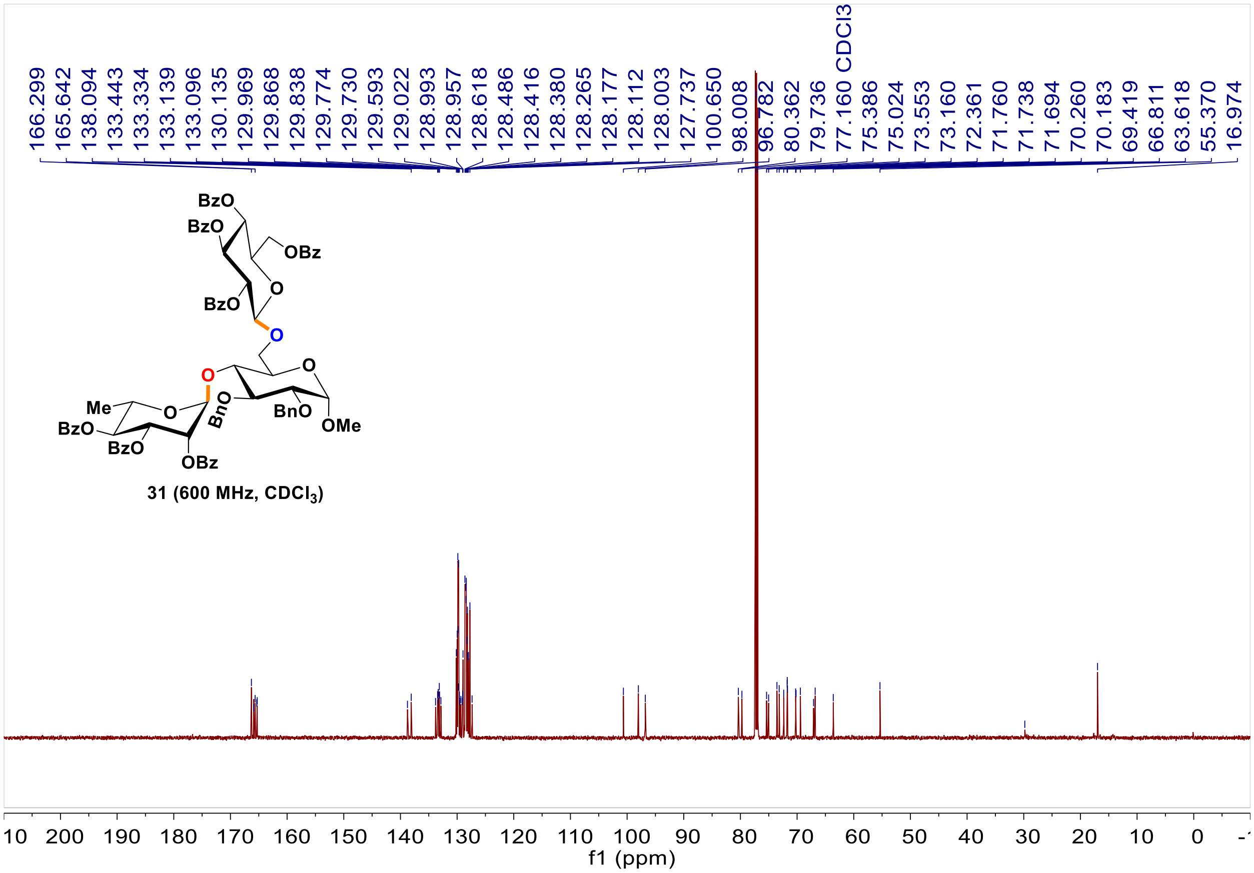
166.299
165.642
138.094
133.443
133.334
133.139
133.096
130.135
129.969
129.868
129.838
129.774
129.730
129.593
129.022
128.993
128.957
128.618
128.486
128.416
128.380
128.265
128.177
128.112
128.003
127.737
100.650
98.008
96.782
80.362
79.736
77.160 CDCl3
75.386
75.024
73.553
73.160
72.361
71.760
71.738
71.694
70.260
70.183
69.419
66.811
63.618
55.370
16.974
BzO
BzO
OBz
BzO
O
O
O
O
Me
O
BnO
BnO
OMe
BzO
BzO
OBz
31 (600 MHz, CDCl$_3$)
f1 (ppm)

REFERENCES AND NOTES

1. K. Arora, P. M. Sherilraj, K. A. Abutwaibe, B. Dhruw, S. L. Mudavath. Exploring glycans as vital biological macromolecules: A comprehensive review of advancements in biomedical frontiers. *Int. J. Biol. Macromol.* **268**, 131511 (2024).

2. A. Lakshminarayanan, M. Richard, B. G. Davis, Studying glycobiology at the single-molecule level. *Nat. Rev. Chem.* **2**, 148–159 (2018).

3. X. Cao, X. Du, H. Jiao, Q. An, R. Chen, P. Fang, J. Wang, B. Yu, Carbohydrate-based drugs launched during 2000-2021. *Acta Pharm. Sin. B* **12**, 3783–3821 (2022).

4. A. Jaiman, M. Thattai, Glycan biosynthesis: Structure, information, and heterogeneity. *Biophys. J.* **108**, 370a (2015).

5. C. J. Crawford, P. H. Seeberger, Advances in glycoside and oligosaccharide synthesis. *Chem. Soc. Rev.* **52**, 7773–7801 (2023).

6. V. W. Tai, B. Imperiali, Substrate specificity of the glycosyl donor for oligosaccharyl transferase. *J. Org. Chem.* **66**, 6217–28 (2001).

7. T. J. Boltje, T. Buskas, G. J. Boons, Opportunities and challenges in synthetic oligosaccharide and glycoconjugate research. *Nat. Chem.* **1**, 611–22 (2009).

8. W. Li, B. Yu, Gold-catalyzed glycosylation in the synthesis of complex carbohydrate-containing natural products. *Chem. Soc. Rev.* **47**, 7954–7984 (2018).

9. X. Zhu, R. R. Schmidt, New principles for glycoside-bond formation. *Angew. Chem. Int. Ed. Engl.* **48**, 1900–1934 (2009).

10. E. Fischer, Ueber die Glucoside der Alkohole. *Ber. Dtsch. Chem. Ges.* **26**, 2400–2412 (1893).

11. W. Koenigs, E. Knorr, Ueber einige Derivate des Traubenzuckers und der Galactose. *Ber. Dtsch. Chem. Ges.* **34**, 957–981 (1901).

12. K. Toshima, Glycosyl fluorides in glycosidations. *Carbohydr. Res.* **327**, 15–26 (2000).

13. R. R. Schmidt, New methods for the synthesis of glycosides and oligosaccharides—Are there alternatives to the Koenigs-Knorr method? *Angew. Chem. Int. Ed. Engl.* **25**, 212–235 (1986).

14. B. Yu, H. Tao, Glycosyl trifluoroacetimidates. Part 1: Preparation and application as new glycosyl donors. *Tetrahedron Lett.* **42**, 2405–2407 (2001).

15. G. Lian, X. Zhang, B. Yu, Thioglycosides in carbohydrate research. *Carbohydr. Res.* **403**, 13–22 (2015).

16. T. Tsuda, R. Arihara, S. Sato, M. Koshiba, S. Nakamura, S. Hashimoto, Direct and stereoselective synthesis of β-D-mannosides using 4,6-*O*-benzylidene-protected mannosyl diethyl phosphite as a donor. *Tetrahedron* **61**, 10719–10733 (2005).

17. P. Li, H. He, Y. Zhang, R. Yang, L. Xu, Z. Chen, Y. Huang, L. Bao, G. Xiao, Glycosyl *ortho*-(1-phenylvinyl)benzoates versatile glycosyl donors for highly efficient synthesis of both *O*-glycosides and nucleosides. *Nat. Commun.* **11**, 405 (2020).

18. Y. Li, Y. Yang, B. Yu, An efficient glycosylation protocol with glycosyl *ortho*-alkynylbenzoates as donors under the catalysis of $Ph_3PAuOTf$. *Tetrahedron Lett.* **49**, 3604–3608 (2008).

19. H. Ding, J. Lv, X. L. Zhang, Y. Xu, Y. H. Zhang, X. W. Liu, Efficient *O*- and *S*-glycosylation with *ortho*-2,2-dimethoxycarbonylcyclopropylbenzyl thioglycoside donors by catalytic strain-release. *Chem. Sci.* **15**, 3711–3720 (2024).

20. L. Song, M. Yang, M. Li, Y. Qiu, Y. Dai, J. Tian, X. Zheng, Y. Zhao, X. Yao, H. Tao, *N*-(*para*-methoxyphenylpropargyl) pyrrole-2-carboxylate (PPPC) glycosides as donors for glycosylation. *Org. Lett.* **27**, 396–401 (2025).

21. L. Yang, Y. Qiu, L. Pan, Z. Zhou, Y. Tan, M. Li, Y. Zhu, X. Yang, X. Zheng, W. Gu, Y. Ren, H. Tao, $In(OTf)_3$-only-catalyzed glycosylation via activation of an alkyne appended with an amide auxiliary group. *Org. Lett.* **27**, 1735–1740 (2025).

22. M. Li, L. Song, L. Yang, Y. Zhao, M. Yang, H. Tao, Development of glycosylation protocols using glycosyl *N*-phenylethynyl pyrrole-2-carboxylates as donors. *Org. Lett.* **26**, 9926–9930 (2024).

23. C. Cai, X. Sun, Y. Feng, Q. Zhang, Y. Chai, Insights into the activation of alkyne-installed glycosyl donors with dual acidic metal catalysts: Reaction pathway, influencing factors, and enlightenment for glycosylation. *Org. Lett.* **24**, 6266–6271 (2022).

24. B.-B. Zhan, K.-S. Xie, Q. Zhu, W. Zhou, D. Zhu, B. Yu, A $Cu(OTf)_2$-catalyzed glycosylation with glycosyl *ortho*-*N*-phthalimidoylpropynyl benzoates as donors. *Org. Lett.* **25**, 3841–3846 (2023).

25. S. Adhikari, K. N. Baryal, D. Zhu, X. Li, J. Zhu, Gold-catalyzed synthesis of 2-deoxy glycosides using *S*-but-3-ynyl thioglycoside donors. *ACS Catal.* **3**, 57–60 (2013).

26. J. Wang, X. Lan, S. Zhang, C. Cai, Q. Zhang, Y. Feng, Y. Chai, *S*-*o*-(p-methoxyphenylethynyl)benzyl (SMPEB) glycosides for catalytic glycosylation and their application in the synthesis of polyporus umbellatus polysaccharides. *Org. Lett.* **25**, 6116–6121 (2023).

27. F. Yang, Q. Wang, B. Yu, *Ortho*-alkynylphenyl thioglycosides as a new type of glycosylation donors under the catalysis of Au(I) complexes. *Tetrahedron Lett.* **53**, 5231–5234 (2012).

28. H. Liu, Z.-F. Liang, H.-J. Liu, J.-X. Liao, L.-J. Zhong, Y.-H. Tu, Q.-J. Zhang, B. Xiong, J.-S. Sun, *Ortho*-methoxycarbonylethynylphenyl thioglycosides (MCEPTs): Versatile glycosyl donors enabled by electron-withdrawing substituents and catalyzed by Gold(I) or Cu(II) complexes. *J. Am. Chem. Soc.* **145**, 3682–3695 (2023).

29. M. Li, X. Xiang, Z. Zhou, X. Zhou, M. Yang, Y. Zhao, X. Zheng, H. Tao, Copper-catalyzed glycosylation protocol based on 1,4-naphthoquinone-derived thioglycosides. *Org. Lett.* **27**, 2262–2267 (2025).

30. Z. Zheng, L. Zhang, Gold-catalyzed synthesis of α-D-glucosides using an *o*-ethynylphenyl β-D-1-thioglucoside donor. *Carbohydr. Res.* **471**, 56–63 (2019).

31. X. Chen, D. Shen, Q. Wang, Y. Yang, B. Yu, *Ortho*-(methyltosylaminoethynyl)benzyl glycosides as new glycosyl donors for latent-active glycosylation. *Chem. Commun.* **51**, 13957–13960 (2015).

32. Y. Hu, K. Yu, L.-L. Shi, L. Liu, J.-J. Sui, D.-Y. Liu, B. Xiong, J.-S. Sun, *O*-(*p*-methoxyphenylethynyl)phenyl glycosides: Versatile new glycosylation donors for the highly efficient construction of glycosidic linkages. *J. Am. Chem. Soc.* **139**, 12736–12744 (2017).

33. S. Y. Zhou, X. P. Hu, H. J. Liu, Q. J. Zhang, J. X. Liao, Y. H. Tu, J. S. Sun, 8-(methyltosylaminoethynyl)-1-naphthyl (MTAEN) glycosides: Potent donors in glycosides synthesis. *Org. Lett.* **24**, 653–657 (2022).

34. X. Lan, C. Cai, J. Wang, Q. Zhang, Y. Feng, Y. Chai, Tf_2O/TfOH catalytic glycosylation using *o*-(*p*-methoxyphenylethynyl)benzyl glycosides as donors and its application in synthesis of oligosaccharides. *Tetrahedron Lett.* **116**, 154342 (2023).

35. A. K. Kayastha, S. Hotha, Versatile gold catalyzed transglycosidation at ambient temperature. *Chem. Commun.* **48**, 7161–7163 (2012).

36. B. Hao, R. Li, P. Wang, Y. Wang, X. Li, P. Xu, Q. Zhang, X. Zhu, X. Zhang, Y. Zhu, Minimally protected and stereoselective *O*-glycosylation of carboxylic acid allows rapid access to α-1-*O*- and 2-*O*-acyl glycosides. *J. Am. Chem. Soc.* **147**, 13744–13753 (2025).

37. W. Liu, Z. Hu, P. Xu, B. Yu, Synthesis of anticoagulant pentasaccharide fondaparinux via 3,5-dimethyl-4-(2′-phenylethynylphenyl)phenyl glycosides. *Org. Lett.* **25**, 8506–8510 (2023).

38. Z. Hu, Y. Tang, B. Yu, Glycosylation with 3,5-dimethyl-4-(2′-phenylethynylphenyl)phenyl (EPP) glycosides via a dearomative activation mechanism. *J. Am. Chem. Soc.* **141**, 4806–4810 (2019).

39. W. Liu, Z. Wang, T. Gulzar, X. Zhang, G. Ding, P. Xu, B. Yu, 4-(2′-phenylethynylphenyl)phenyl glycosides as glycosylation donors. *Pure Appl. Chem.* **95**, 965–970 (2023).

40. Y. Zhou, X. Zhang, Y. Zhang, L. Ruan, J. Zhang, D. Zhang-Negrerie, Y. Du, Iodocyclization of *N*-arylpropynamides mediated by hypervalent iodine reagent: Divergent synthesis of iodinated quinolin-2-ones and spiro[4,5]trienones. *Org. Lett.* **19**, 150–153 (2017).

41. T. Okitsu, A. Horike, N. Shimazawa, A. Wada, A dearomative *ipso*-iodocyclization/desymmetrization sequence leading to optically active tricyclic piperazine scaffolds. *Org. Biomol. Chem.* **18**, 3501–3511 (2020).

42. Z. Qiao, C. Shao, Y. Gao, K. Liang, H. Yin, F.-X. Chen, An electrophilic thiocyanation/*ipso*-cyclization leading to spirocyclohexadienones. *Tetrahedron Lett.* **100**, 153875 (2022).

43. S. Hotha, S. Kashyap, Propargyl glycosides as stable glycosyl donors: Anomeric activation and glycoside syntheses. *J. Am. Chem. Soc.* **128**, 9620–9621 (2006).

44. B. Yu, J. Sun, Glycosylation with glycosyl *N*-phenyltrifluoroacetimidates (PTFAI) and a perspective of the future development of new glycosylation methods. *Chem. Commun.* **46**, 4668–4679 (2010).

45. X. Zhang, R. C. Larock, Synthesis of spiro[4.5]trienones by intramolecular *ipso*-halocyclization of 4-(*p*-methoxyaryl)-1-alkynes. *J. Am. Chem. Soc.* **127**, 12230–12231 (2005).

46. H. Vorbrueggen, Adventures in silicon-organic chemistry. *Acc. Chem. Res.* **28**, 509–520 (1995).

47. J. Liao, J. Sun, B. Yu, An improved procedure for nucleoside synthesis using glycosyl trifluoroacetimidates as donors. *Carbohydr. Res.* **344**, 1034–1038 (2009).

48. S. Dey, P. Garner, Synthesis of tert-butoxycarbonyl (Boc)-protected purines. *J. Org. Chem.* **65**, 7697–7699 (2000).

49. G.-J. Boons, S. Isles, Vinyl glycosides in oligosaccharide synthesis (part 1): A new latent-active glycosylation strategy. *Tetrahedron Lett.* **35**, 3593–3596 (1994).

50. T. C. Shiao, R. Roy, “Active-latent” thioglycosyl donors and acceptors in oligosaccharide syntheses. *Top. Curr. Chem.* **301**, 69–108 (2011).

51. K. M. Koeller, C. H. Wong, Synthesis of complex carbohydrates and glycoconjugates: Enzyme-based and programmable one-pot strategies. *Chem. Rev.* **100**, 4465–4494 (2000).

52. O. Kanie, Y. Ito, T. Ogawa, Orthogonal glycosylation strategy in oligosaccharide synthesis. *J. Am. Chem. Soc.* **116**, 12073–12074 (1994).

53. M. Liu, X. Qin, X. S. Ye, Glycan assembly strategy: From concept to application. *Chem. Rec.* **21**, 3256–3277 (2021).

54. C.-C. Wang, J.-C. Lee, S.-Y. Luo, S. S. Kulkarni, Y.-W. Huang, C.-C. Lee, K.-L. Chang, S.-C. Hung, Regioselective one-pot protection of carbohydrates. *Nature* **446**, 896–899 (2007).

55. R. Liu, Q. Hua, Q. Lou, J. Wang, X. Li, Z. Ma, Y. Yang, NIS/TMSOTf-promoted glycosidation of glycosyl *ortho*-hexynylbenzoates for versatile synthesis of *O*-glycosides and nucleosides. *J. Org. Chem.* **86**, 4763–4778 (2021).

56. P. R. Tentscher, J. S. Arey, Geometries and vibrational frequencies of small radicals: Performance of coupled cluster and more approximate methods. *J. Chem. Theory Comput.* **8**, 2165–2179 (2012).

57. T. Giroday, M. M. Montero-Campillo, N. Mora-Diez, Thermodynamic stability of PFOS: M06-2X and B3LYP comparison. *Comput. Theor. Chem.* **1046**, 81–92 (2014).

58. J.-J. Sui, D.-C. Xiong, X.-S. Ye, Copper-mediated *O*-arylation of lactols with aryl boronic acids. *Chin. Chem. Lett.* **30**, 1533–1537 (2019).

59. J. Park, E. Park, A. Kim, S.-A. Park, Y. Lee, K.-W. Chi, Y. H. Jung, I. S. Kim, Pd-catalyzed decarboxylative coupling of propiolic acids: One-pot synthesis of 1,4-disubstituted 1,3-diynes via sonogashira−homocoupling sequence. *J. Org. Chem.* **76**, 2214–2219 (2011).

60. T. Zenkoh, H. Tanaka, H. Setoi, T. Takahashi, Solid-phase synthesis of aryl *O*-glycoside using aqueous base and phase-transfer catalyst. *Synlett* **2002**, 0867–0870 (2002).

61. T. Dohi, D. Kato, R. Hyodo, D. Yamashita, M. Shiro, Y. Kita. Discovery of stabilized bisiodonium salts as intermediates in the carbon–carbon bond formation of alkynes. *Angew. Chem. Int. Ed. Engl.* **50**, 3784–3787 (2011).

62. Q. F. Yu, Y. H. Zhang, Q. Yin, B. X. Tang, R. Y. Tang, P. Zhong, J. H. Li, Electrophilic *ipso*-iodocyclization of *N*-(4-methylphenyl)propiolamides: Selective synthesis of 8-methyleneazaspiro[4,5]trienes. *J. Org. Chem.* **73**, 3658–3661 (2008).

63. T. Schmidt, R. Schmieder, W. M. Müller, B. Kiupel, F. Vögtle, Chiral amide rotaxanes with glucose stoppers–Synthesis, chiroptical properties and wheel-axle interactions. *Eur. J. Org. Chem.* **1998**, 2003–2007 (1998).

64. T. Ohyanagi, N. Nagahori, K. Shimawaki, H. Hinou, T. Yamashita, A. Sasaki, T. Jin, T. Iwanaga, M. Kinjo, S. Nishimura, Importance of sialic acid residues illuminated by live animal imaging using phosphorylcholine self-assembled monolayer-coated quantum dots. *J. Am. Chem. Soc.* **133**, 12507–12517 (2011).

65. T. Coyle, H. Brumer, K. A. Stubbs, An improved preparation of some aryl α-l-arabinofuranosides for use as chromogenic substrates for α-l-arabinofuranosidases. *Can. J. Chem.* **93**, 1176–1180 (2015).

66. Y. Zhang, Z. Chen, Y. Huang, S. He, X. Yang, Z. Wu, X. Wang, G. Xiao, Modular synthesis of nona-decasaccharide motif from psidium guajava polysaccharides: Orthogonal one-pot glycosylation strategy. *Angew. Chem. Int. Ed. Engl.* **59**, 7576–7584 (2020).

67. H. Zhao, M. Zhou, L. Duan, W. Wang, J. Zhang, D. Wang, X. Liang, Efficient synthesis and anti-fungal activity of oleanolic acid oxime esters. *Molecules* **18**, 3615–3629 (2013).

68. D. J. Cox, G. P. Singh, A. J. A. Watson, A. J. Fairbanks, Neighbouring group participation during glycosylation: Do 2-substituted ethyl ethers participate? *Eur. J. Org. Chem.* **2014**, 4624–4642 (2014).

69. J. Xue, Z. Guo, Synthetic studies on the carbohydrate moiety of amipurimycin. *J. Carbohyd. Chem.* **27**, 51–69 (2008).

70. S. van der Vorm, T. Hansen, H. S. Overkleeft, G. A. van der Marel, J. D. C. Codée, The influence of acceptor nucleophilicity on the glycosylation reaction mechanism. *Chem. Sci.* **8**, 1867–1875 (2017).

71. F. Zhang, W. Zhang, Y. Zhang, D. P. Curran, G. Liu, Synthesis and applications of a light-fluorous glycosyl donor. *J. Org. Chem.* **74**, 2594–2597 (2009).

72. C. S. Rye, S. G. Withers, Elucidation of the mechanism of polysaccharide cleavage by chondroitin AC lyase from flavobacterium heparinum. *J. Am. Chem. Soc.* **124**, 9756–9767 (2002).

73. S. Fletcher, V. M. Shahani, A. J. Lough, P. T. Gunning, Concise access to N9-mono-, N2-mono- and N2,N9-di-substituted guanines via efficient Mitsunobu reactions. *Tetrahedron* **66**, 4621–4632 (2010).

74. M. M. Matin, S. C. Bhattacharjee, P. Chakraborty, M. S. Alam, Synthesis, PASS predication, in vitro antimicrobial evaluation and pharmacokinetic study of novel n-octyl glucopyranoside esters. *Carbohyd. Res.* **485**, 107812 (2019).

75. H. Y. Wang, C. J. Simmons, S. A. Blaszczyk, P. G. Balzer, R. Luo, X. Duan, W. Tang, Isoquinoline-1-carboxylate as a traceless leaving group for chelation-assisted glycosylation under mild and neutral reaction conditions. *Angew. Chem. Int. Ed. Engl.* **56**, 15698–15702 (2017).

76. H. Liu, J.-X. Liao, Y. Hu, Y.-H. Tu, J.-S. Sun, A highly efficient approach to construct (epi)-podophyllotoxin-4-*O*-glycosidic linkages as well as its application in concise syntheses of etoposide and teniposide. *Org. Lett.* **18**, 1294–1297 (2016).

77. M. Adinolfi, G. Barone, A. Iadonisi, M. Schiattarella, Activation of glycoyl trihaloacetimidates with acid-washed molecular sieves in the glycosidation reaction. *Org. Lett.* **5**, 987–989 (2003).

78. B. Mishra, M. Neralkar, S. Hotha, Stable alkynyl glycosyl carbonates: Catalytic anomeric activation and synthesis of a tridecasaccharide reminiscent of mycobacterium tuberculosis cell wall lipoarabinomannan. *Angew. Chem. Int. Ed. Engl.* **55**, 7786–7791 (2016).

79. M. Heuckendorff, L. T. Poulsen, H. H. Jensen, Remote electronic effects by ether protecting groups fine-tune glycosyl donor reactivity. *J. Org. Chem.* **81**, 4988–5006 (2016).

80. A. Das, N. Jayaraman, Carbon tetrachloride-free allylic halogenation-mediated glycosylations of allyl glycosides. *Org. Biomol. Chem.* **19**, 9318–9325 (2021).

81. X. Liu, G.-E. Wen, J.-C. Liu, J.-X. Liao, J.-S. Sun, Total synthesis of scutellarin and apigenin 7-O-β-d-glucuronide. *Carbohyd. Res.* **475**, 69–73 (2019).

82. S. A. Thadke, B. Mishra, S. Hotha, Gold(III)-catalyzed glycosidations for 1,2-*trans* and 1,2-*cis* furanosides. *J. Org. Chem.* **79**, 7358–7371 (2014).

83. J. Park, T. J. Boltje, G. J. Boons, Direct and stereoselective synthesis of α-linked 2-deoxyglycosides. *Org. Lett.* **10**, 4367–4370 (2008).

84. Y. Li, X. Yang, Y. Liu, C. Zhu, Y. Yang, B. Yu, Gold(I)-catalyzed glycosylation with glycosyl *ortho*-alkynylbenzoates as donors: General scope and application in the synthesis of a cyclic triterpene saponin. *Chem. A Eur. J.* **16**, 1871–1882 (2010).

85. G. J. Liu, X. T. Zhang, G. W. Xing, A general method for *N*-glycosylation of nucleobases promoted by (p-Tol)$_2$SO/Tf$_2$O with thioglycoside as donor. *Chem. Commun.* **51**, 12803–12806 (2015).

86. B. V. Rao, S. Manmode, S. Hotha, Propargyl 1,2-orthoesters for a catalytic and stereoselective synthesis of pyrimidine nucleosides. *J. Org. Chem.* **80**, 1499–1505 (2015).

87. Q. Zhang, J. Sun, Y. Zhu, F. Zhang, B. Yu, An efficient approach to the synthesis of nucleosides: Gold(I)-catalyzed *N*-glycosylation of pyrimidines and purines with glycosyl *ortho*-alkynyl benzoates. *Angew. Chem. Int. Ed. Engl.* **50**, 4933–4936 (2011).

88. L. Cicco, F. M. Perna, A. Falcicchio, A. Altomare, F. Messa, A. Salomone, V. Capriati, P. Vitale, 1,3-Dipolar cycloaddition of alkanone enolates with azides in deep eutectic solvents for the metal-free regioselective synthesis of densely functionalized 1,2,3-triazoles. *Eur. J. Org. Chem.* **2022**, e202200843 (2022).

89. X. Huang, L. Huang, H. Wang, X.-S. Ye, Iterative one-pot synthesis of oligosaccharides. *Angew. Chem. Int. Ed. Engl.* **43**, 5221–5224 (2004).

90. A. Attouche, D. Urban, J.-M. Beau, A tin-free regioselective radical de-*O*-benzylation by an intramolecular hydrogen atom transfer on carbohydrate templates. *Angew. Chem. Int. Ed. Engl.* **52**, 9572–9575 (2013).
